# Expeditious chemical synthesis of xylomannans disproves the proposed antifreeze activities

**DOI:** 10.1093/nsr/nwae296

**Published:** 2024-08-23

**Authors:** Qian Zhu, Simone Nicolardi, Yuanguang Wang, Yasong Liu, Peng Xu, Jianjun Wang, Dapeng Zhu, Biao Yu

**Affiliations:** State Key Laboratory of Chemical Biology, Shanghai Institute of Organic Chemistry, University of Chinese Academy of Sciences, Chinese Academy of Sciences, Shanghai 200032, China; Center for Proteomics and Metabolomics, Leiden University Medical Center, Leiden 2333ZA, The Netherlands; Key Laboratory of Green Printing, Beijing National Laboratory for Molecular Science, Institute of Chemistry, Chinese Academy of Sciences, Beijing 100190, China; State Key Laboratory of Chemical Biology, Shanghai Institute of Organic Chemistry, University of Chinese Academy of Sciences, Chinese Academy of Sciences, Shanghai 200032, China; State Key Laboratory of Chemical Biology, Shanghai Institute of Organic Chemistry, University of Chinese Academy of Sciences, Chinese Academy of Sciences, Shanghai 200032, China; Key Laboratory of Green Printing, Beijing National Laboratory for Molecular Science, Institute of Chemistry, Chinese Academy of Sciences, Beijing 100190, China; Institute of Translational Medicine, Shanghai Jiao Tong University, Shanghai 200240, China; State Key Laboratory of Chemical Biology, Shanghai Institute of Organic Chemistry, University of Chinese Academy of Sciences, Chinese Academy of Sciences, Shanghai 200032, China

**Keywords:** xylomannan, glycosylation, polysaccharide, total synthesis, antifreeze

## Abstract

Cold-adapted species are able to generate cryoprotective proteins and glycoproteins to prevent freezing damage. The [→4)-*β*-D-Man*p*-(1→4)-*β*-D-Xyl*p*-(1→]*_n_* xylomannan from the Alaska beetle *Upis ceramboides* was disclosed by Walters and co-workers in 2009 as the first glycan-based antifreeze agent, which was later reported to be found in diverse taxa. Here, we report the rapid synthesis of four types of xylomannans, including the proposed antifreeze xylomannan up to a 64-mer (Type I), the regioisomeric [→3)-*β*-D-Man*p*-(1→4)-*β*-D-Xyl*p*-(1→]*_n_* 16-mer (Type II), the diastereomeric [→4)-*β*-L-Man*p*-(1→4)-*β*-D-Xyl*p*-(1→]*_n_* 16-mer (Type III) and the block-wise [→4)-*β*-D-Man*p*-(1→]*_m_*[→4)-*β*-D-Xyl*p*-(1→]*_n_* 32-mer (Type IV), by employing a strategic iterative exponential glycan growth (IEGG) process. The nuclear magnetic resonance spectral data of the alleged natural xylomannan are in accordance only to those of the block-wise Type IV glycan and none of these synthetic xylomannans has been found to be capable of inducing thermal hysteresis. These results disprove the previous reports about the natural occurrence of antifreeze xylomannans.

## Teaser

The alleged natural antifreeze xylomannan has been disproved via the chemical synthesis of four types of xylomannan glycans using the gold(I)-catalyzed strategic iterative exponential glycan growth (IEGG) process.

## INTRODUCTION

**Figure 1.**
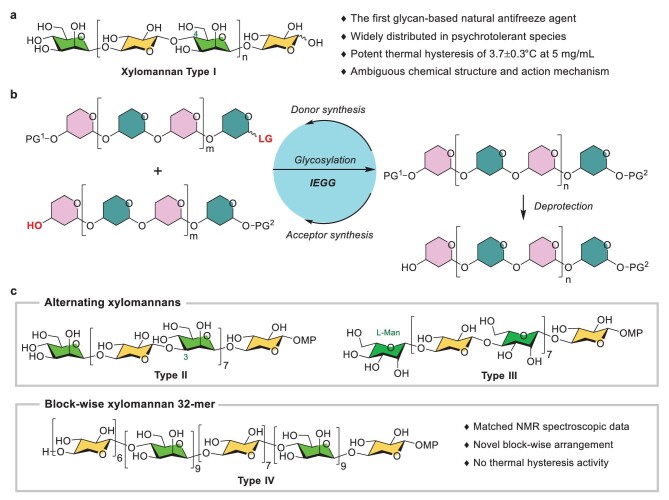
The originally proposed and presently synthesized antifreeze xylomannans and the general synthetic strategy. (a) The alleged antifreeze xylomannan (Type I). (b) Schematic illustration of the iterative exponential glycan growth (IEGG) strategy for glycan synthesis. (c) Synthetic xylomannans (Type I–IV) in this work. PG, protecting group; LG, leaving group; MP, 4-methoxyphenyl.

Subzero temperatures induce the formation of ice crystals in living organisms, causing cell membrane rupture, organelle damage and cell death. Therefore, a variety of psychrotolerant species have evolved with cryoprotective agents to survive freezing environments. The production of antifreeze proteins (AFPs) and glycoproteins (AFGPs) plays a pivotal role in the adaptation to subzero environments [1–3]. The AF(G)Ps are able to not only lower the freezing point of body fluid non-colligatively, but also inhibit the growth of small ice crystals into deleterious large ones [4]. The difference between the colligative melting and hysteretic freezing points, termed thermal hysteresis (TH), is diagnostic for the presence of macromolecular antifreeze substances. In 2009, Walters and co-workers identified an antifreeze xylomannan—the first non-protein TH-producing biomolecule—from the freeze-tolerant Alaskan beetle *Upis ceramboides* by means of ice-affinity purification [5]. This xylomannan exhibited a remarkable TH of 3.7 ± 0.3°C at 5 mg/mL, which is comparable to that of the most active insect AFPs, as well as potent inhibition of ice recrystallization [5,6]. Intriguingly, this type of TH-producing xylomannan was later found in various organisms across diverse taxa, including plants, frogs and insects, indicating its significance in cold tolerance although the exact function remains elusive (Fig. 1a) [6–8].

Cryopreservation of cells and biologics is essential in all biomedical research from routine sample storage to cell-based therapies, making the development of biocompatible cryoprotectants such as polysaccharides highly desirable [9]. Considerable efforts have been devoted to delineating the natural antifreeze xylomannan [10–13]. The chemical structure of the antifreeze xylomannan was initially characterized to be [→4)-*β*-D-Man*p*-(1→4)-*β*-D-Xyl*p*-(1→]*_n_* by Walters and co-workers on the basis of nuclear magnetic resonance (NMR) and mass spectrometry (MS) analysis combined with enzymatic degradative studies [5]. However, small possibilities of block-wise and/or branched arrangement of the Man*p* and Xyl*p* units were not excluded [5,10]. Although accompanying lipid components were detected in the ice-purified sample, they have not been proven to be covalently linked to the glycan. In 2011, the Crich group synthesized three [→4)-*β*-D-Man*p*-(1→4)-*β*-D-Xyl*p*-(1→]*_n_* xylomannan oligosaccharides (*n* = 2–4). Comparison of the NMR spectroscopic data supported the initially proposed xylomannan structure [10]. At around the same time, Ito and collaborators described the chemical synthesis of a similar xylomannan tetrasaccharide; NMR analysis and molecular modeling indicated a helical amphiphilic nature of the xylomannan, which could possibly confer the ice-binding capability [11]. In 2019, Serianni and co-workers synthesized five ^13^C-labelled xylomannan glycans; conformational analysis based on NMR experiments, density functional theory calculations, coupled with molecular dynamic simulations also suggested the helical amphiphilic topography [12].

Notwithstanding, the structure of the antifreeze xylomannan remained not conclusive in view of the suspicious discrepancies in the NMR data between the synthetic oligosaccharides and the authentic xylomannan, which were ascribed to the short length of the synthetic fragments. In addition, no TH property was detected for the short synthetic xylomannan [11]. As such, the synthesis of longer xylomannans has become necessary for the structural confirmation of the natural xylomannan as well as the verification of its antifreeze activity. In fact, the synthesis of glycans that are longer than 20-mer has only been performed sporadically, and most of those syntheses relied on linear or multiplicative assembly tactics [14]. In 2020, we developed an iterative exponential glycan growth (IEGG) strategy based on the gold(I)-catalysed glycosylation method for the synthesis of a 128-mer rhamnomannan relevant to the *O*-antigen of *Bacteroides vulgatus* [15]. This IEGG process entailed a parallel synthesis of glycosyl donor and acceptor from the same precursor glycan and subsequent glycosylation to produce the double-sized glycan, which can be invoked to the next cycle of assembly (Fig. 1b). By employing the IEGG process, we accomplished the rapid synthesis of the proposed [→4)-*β*-D-Man*p*-(1→4)-*β*-D-Xyl*p*-(1→]*_n_* glycans up to a 64-mer (Type I) and the regioisomeric [→3)-*β*-D-Man*p*-(1→4)-*β*-D-Xyl*p*-(1→]*_n_* 16-mer (Type II), the diastereomeric [→4)-*β*-L-Man*p*-(1→4)-*β*-D-Xyl*p*-(1→]*_n_* 16-mer (Type III) and the block-wise [→4)-*β*-D-Man*p*-(1→]*_m_*[→4)-*β*-D-Xyl*p*-(1→]*_n_* 32-mer (Type IV) (Fig. 1c). Structural analysis of these synthetic xylomannans and measurement of their TH-producing activities led us to disprove the structural assignment of the natural xylomannan as well as the natural occurrence of such antifreeze xylomannans.

## RESULTS

### Synthesis of the proposed natural xylomannans (Type I)

The initially proposed antifreeze xylomannan contains two types of glycosidic connections, i.e. Man-(1β→4)-Xyl and Xyl-(1β→4)-Man. Stereoselective formation of the β-mannopyranoside linkages is known to be difficult, whereas the β-xylopyranoside linkages can be robustly constructed with the assistance of neighboring group participation [16,17]. Therefore, we decided to fix the β-mannopyranoside linkage at a disaccharide level and to extend the sugar chain resorting to the β-specific xylopyranosylation.

**Figure 2.**
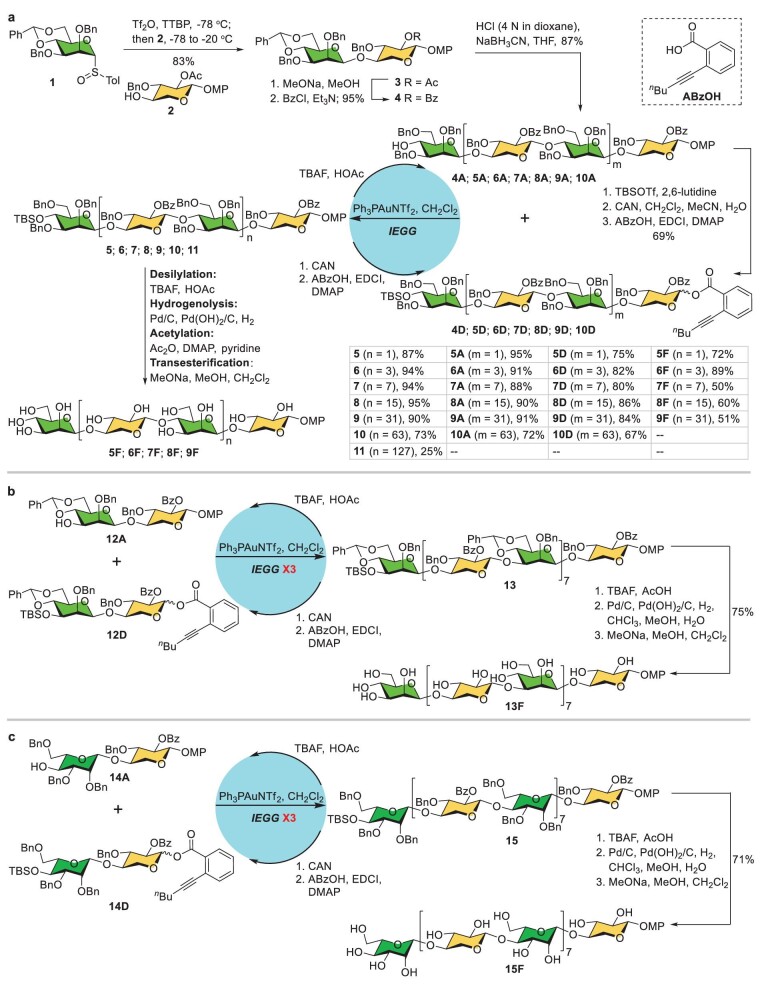
Synthesis of Type I–III xylomannans. (a) Preparation of H[→4)-*β*-D-Man*p*-(1→4)-*β*-D-Xyl*p*-(1→]*_n_*OMP glycans **5F**–**9F**. (b) Preparation of H[→3)-*β*-D-Man*p*-(1→4)-*β*-D-Xyl*p*-(1→]_8_OMP **13F**. (c) Preparation of H[→4)-*β*-L-Man*p*-(1→4)-*β*-D-Xyl*p*-(1→]_8_OMP **15F**. Tol, 4-methylphenyl; Tf, trifluoromethanesulfonyl; TTBP, 2,4,6-tri-*tert*-butylpyrimidine; TBS, *tert*-butyldimethylsilyl; CAN, cerium (IV) ammonium nitrate; EDCI, 1-(3-dimethylaminopropyl)-3-ethylcarbodiimide hydrochloride; DMAP, 4-dimethylaminopyridine; TBAF, tetrabutylammonium fluoride.

Our synthesis commenced with a large-scale preparation of the ManXyl disaccharide building blocks (Fig. 2a). Activation of mannopyranosyl sulfoxide donor **1** with trifluoromethanesulfonic anhydride in the presence of 2,4,6-tri-*tert*-butylpyrimidine followed by the addition of xylose-derived acceptor **2** effectively provided disaccharide **3** (83%, β/α > 19 : 1) [10,18]. The 2-*O*-acetyl group in **3** was changed to the benzoyl group to prevent the possible generation of orthoesters during the planned IEGG assembly [19]. Regioselective reductive opening of the benzylidene acetal with hydrogen chloride and sodium cyanoborohydride delivered disaccharide acceptor **4A** in 87% yield [10,20]. Capping of the free hydroxyl group in **4A** (TBSOTf, 2,6-lutidine) followed by oxidative cleavage of the anomeric 4-methoxyphenyl (MP) group with ceric ammonium nitrate (CAN) and subsequent esterification with *o*-hexynylbenzoic acid furnished disaccharide donor **4D** smoothly (69% over three steps). Coupling of disaccharide acceptor **4A** and donor **4D** under the Au(I)-catalysed conditions (0.1 equiv. Ph_3_PAuNTf_2_, 5 Å MS, CH_2_Cl_2_) proceeded well, leading to tetrasaccharide **5** in a high 87% yield [21]. Treatment of **5** with tetrabutylammonium fluoride (TBAF) and acetic acid gave rise to tetrasaccharide acceptor **5A** nearly quantitatively (95%). Oxidative cleavage of the anomeric MP group with CAN followed by condensation with *o*-hexynylbenzoic acid produced tetrasaccharide **5D** in a satisfactory 75% yield. These robust transformations laid the foundation for the subsequent IEGG assembly of the desired xylomannans, which involved the removal of *tert*-butyldimethylsilyl (TBS) ether with TBAF in the presence of acetic acid, cleavage of the anomeric MP group under the oxidation of CAN and subsequent condensation of the hemiacetal with *o*-hexynylbenzoic acid using 1-(3-dimethylaminopropyl)-3-ethylcarbodiimide hydrochloride and 4-dimethylaminopyridine, and Au(I)-catalysed glycosylation with the acceptor and donor. To our delight, this IEGG assembly cycle could be consecutively applied as many as six times, providing fully protected xylomannans up to a 128-mer (**6**–**10**) in 73%–95% yields. Further implementation of the IEGG cycle led to 256-mer **11** successfully, albeit in only 25% yield. Remarkably, the present IEGG strategy offered a pragmatic approach to polysaccharides, with 64-mer **9** and 128-mer **10** being prepared on scales of 1.1 and 0.6 g, respectively. It is noteworthy that gel permeation chromatography was proven to be viable to purify the long glycans such as 64-mer **9**, 128-mer **10** and 256-mer **11** [15].

Next, we turned to the global cleavage of the protecting groups. The deprotection process required three essential operations: (i) removal of the terminal silyl group with TBAF and acetic acid that had been carried out for the preparation of glycosyl acceptors; (ii) removal of the benzyl groups via hydrogenolysis catalysed by the combination of 10% Pd/C and 20% Pd(OH)_2_/C [22,23]; (iii) removal of the acyl groups via transesterification with methanol in the presence of NaOMe. A redundant acetylation step after hydrogenolysis was found to be necessary for the 8-mer and longer glycans, as the release of numerous hydroxyl groups significantly depressed their solubilities in organic solvents. In addition, repetitive hydrogenolysis was required for 16-mer and longer glycans, because a single hydrogenolysis step was not able to cleave all the benzyl C–O bonds. Thus, five free glycans (**5F**–**9F**) up to 64-mer were prepared in 50%–89% yields. We noticed that these synthetic xylomannans tended to aggregate in water as the sugar length increased. Particularly, 16-mer **7F** showed a poor solubility of ∼3.5 mg/mL in water, which was lower than that used for the assessment of the TH-producing activity for the natural xylomannan [5]. Unexpectedly, the NMR spectroscopic data of 16-mer **7F** differed apparently from those of the natural isolate (*vide infra*; for details, see Table S1).

### Synthesis of 16-mer xylomannans 13F and 15F corresponding to the possible regioisomeric (Type II) and diastereoisomeric (Type III) xylomannans

Considering that the *endo β*-(1→4)-xylosidase used in the original structural characterization might also be able to cleave 1,3-*β*-xylopyranosidic linkage [24], the regioisomeric [→3)-*β*-D-Man*p*-(1→4)-*β*-D-Xyl*p*-(1→]*_n_* could not be ruled out from being the authentic natural xylomannan. Indeed, Crich and co-workers once excluded this alternative Xyl-(1β→3)-Man linkage via NMR comparison with a synthetic tetrasaccharide [10]. The preparation of a longer glycan, such as 16-mer **13F**, for comparison with the natural and synthetic Type II xylomannan became imperative to solve the present structural puzzle (Fig. 2b). Thus, starting with the coupling of disaccharide donor **12D** and acceptor **12A**, three rounds of the IEGG process smoothly afforded the fully protected 16-mer **13** on a scale of 2.2 g, which was then transformed into free 16-mer **13F** in a good 75% yield by using the aforementioned deprotection procedure (for details, see Figs S15–S18).

Another possible structure for the natural xylomannan was the diastereomeric Type III in which L-mannose was incorporated instead of the previously assigned D-mannose. Although scarce, L-mannose has been found in natural glycans [25–27]. Thus, we also set out to synthesize the relevant 16-mer **15F** (Fig. 2c). Subjection of disaccharide donor **14D** and acceptor **14A** to three rounds of IEGG assembly provided the fully protected 16-mer **15** on a scale of 1.8 g. Subsequent removal of the TBS ether, benzyl groups and benzoyl groups furnished **15F** in 71% yield (for details, see Figs S19–S22).

The synthetic regioisomeric (Type II) and diastereoisomeric (Type III) xylomannan 16-mers **13F** and **15F** showed better water solubility than the Type I 16-mer **7F**. Unfortunately, discernible deviations were observed for their NMR spectroscopic data in comparison with those of the isolated sample, although their water solubility was enhanced (*vide infra*; for details, see Tables S2 and S3).

### Synthesis of block-wise xylomannan (Type IV) 32-mer **23F**

**Figure 3.**
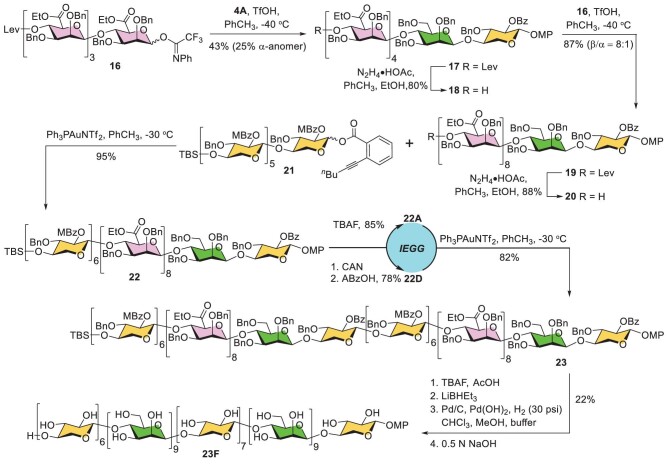
Synthesis of block-wise xylomannan (Type IV) 32-mer **23F**. Lev, levulinoyl; MBz, 4-methylbenzoyl.

Upon carefully examining the structural data of the natural xylomannan reported by Walters and co-workers [5], we found the sequential loss of a similar monosaccharide unit in the mass spectrum, implying the existence of oligomeric mannan and xylan segments. Thus, we devised a block-wise 32-mer **23F** with a Man/Xyl molar ratio of 9 : 7, so as to match the reported Man/Xyl ratio for the natural xylomannan. The synthesis of 32-mer **23F** is depicted in Fig. 3. The D-mannuronate derivative was selected as the precursor of the D-mannose unit due to its β-directing capacity in glycosylation [28–31], thereby enabling convergent construction of the embedded *β*-1,4-mannan substructure without resorting to 4,6-*O*-benzylidene protection. Thus, condensation of tetramannuronate imidate **16** with disaccharide acceptor **4A** under the action of triflic acid delivered hexasaccharide **17** in 43% yield, in addition to 25% of the α-anomer [31,32]. Removal of the terminal levulinoyl group with hydrazine acetate followed by glycosylation with **16** appended another tetramannuronate segment, giving decasaccharide **19** in a good 87% yield and satisfactory 8 : 1 β/α selectivity. Treatment of **19** with hydrazine acetate produced acceptor **20** smoothly. Next, **20** was coupled with *o*-hexynylbenzoate **21** under the promotion of Ph_3_PAuNTf_2_ to afford 16-mer **22** in an excellent 95% yield. Subjection of **22** to one round of the IEGG process doubled the length of the sugar chain effectively, furnishing the fully protected 32-mer **23** on a scale of 0.5 g. Finally, an array of steps including (i) cleavage of the TBS group with TBAF, (ii) reduction of the D-mannuronate residues to the corresponding D-mannose units with lithium triethylborohydride [30], (iii) hydrogenolysis under the catalysis of 10% Pd/C and 20% Pd(OH)_2_/C and (iv) saponification with 0.5 N NaOH solution led to the desired block-wise 32-mer **23F** in an appreciable 22% yield.

### Comparison of NMR spectroscopic data and TH-producing activities

**Figure 4.**
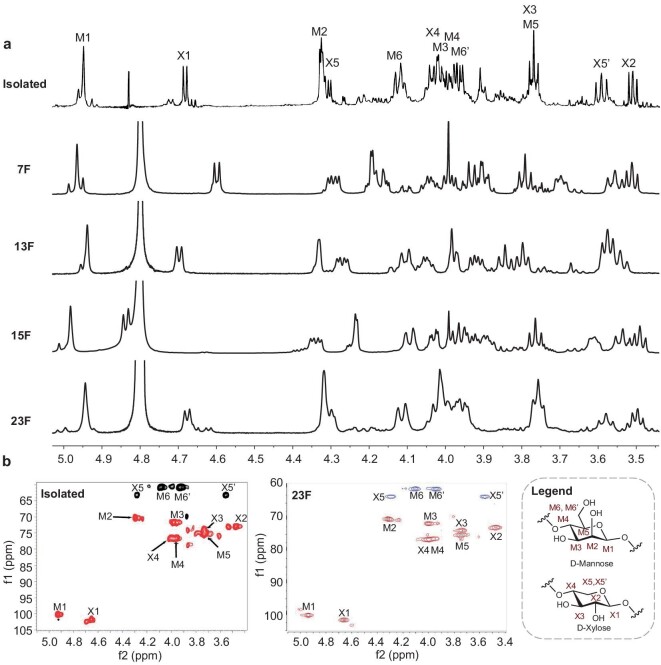
NMR spectral comparison of the natural xylomannan and synthetic xylomannans. (a) Overlaid ^1^H NMR spectra of the natural xylomannan and the synthetic xylomannans **7F, 13F, 15F** and **23F**. (b) ^1^H–^13^C HSQC spectra of the natural xylomannan and block-wise **23F**. The ^1^H NMR and HSQC spectra of the natural xylomannan was reproduced from [5].

With the synthetic xylomannans in hand, we were able to systematically compare their NMR spectroscopic data with those presented in the literature (Fig. 4; for details, see Figs S27–S30). Notably, the resonances of the internal monosaccharide residues were coincident and they dominated the spectra when the length of the sugar chain reached 16 mer, thus simplifying the interpretation of these signals. In line with the previous reports, the ^1^H and ^13^C spectra of 16-mer **7F** resembled those of the natural isolate, except the variances of 1.9 and 1.6 ppm for the anomeric carbons of the mannose and xylose residues, respectively [10,11]. The ^13^C NMR data of **13F** differed greatly from those of the natural xylomannan, possessing significant discrepancies of 9.0 and 10.9 ppm for the C3 and C4 of the mannose residue, respectively, therefore strongly ruling out the regioisomeric Xyl-(1β→3)-Man linkage. Not surprisingly, the ^1^H and ^13^C NMR spectroscopic data of **15F** did not agree with those of the natural xylomannan, indicating that the rare L-mannose was not the component. To our delight, signals that showed in the NMR spectra of **23F** were virtually identical to those reported by Walters and co-workers [5], with the deviations in the ^1^H and ^13^C chemical shifts not exceeding 0.03 and 0.2 ppm, respectively. It is of note that the small discrepancies in the ^1^H NMR spectrum between synthetic **23F** and natural isolate can be ascribed to the trace amounts of impurities in the natural isolate. Moreover, the ^1^H–^13^C hetereonuclear single quantum coherence (HSQC) spectrum of block-wise **23F** closely resembled that of the natural xylomannan (Fig. 4b). Taken together, we were confident that the natural xylomannan comprised *β*-1,4-xylan and *β*-1,4-mannan subunits, with the block-wise [→4)-*β*-D-Man*p*-(1→]*_m_*[→4)-*β*-D-Xyl*p*-(1→]*_n_*, such as **23F**, being a possible structure. However, we could not completely rule out alternative combination patterns of the *β*-1,4-xylan and *β*-1,4-mannan subunits, and the existence of sparse modifications in the natural xylomannan.

The antifreeze properties of the synthetic xylomannans were tested. Alternating xylomannans 16-mer **7F**, 32-mer **8F** and 64-mer **9F** exhibited only marginal TH ranging from 0.01 to 0.03°C at their saturated concentrations. Meanwhile, isomeric xylomannan 16-mer **13F** and **15F** were found to be not capable of inducing TH at a concentration of 10 mg/mL. The block-wise 32-mer **23F**, whose spectral data matched well with those of the natural isolate, did not produce any detectable TH at 10 mg/mL. Matrix-assisted laser-desorption ionization mass analysis of the natural xylomannan indicated an average molecular weight of 1.0–2.4 kDa [5], while the molecular weight of **23F** is 4.9 kDa. Considering that the superior ionization capacity of short glycans over longer ones usually led to underestimation of the average molecular weight of polydisperse polysaccharides [33,34], we believed that monodisperse **23F** could serve as a representative of the natural xylomannan. Therefore, the fact that block-wise 32-mer **23F** did not show any TH-producing activity was sufficient to negate the antifreeze property of the naturally occurring xylomannan. We surmised that the ice-purified substance that was studied by Walters and co-workers might have contained some hyperactive components that conferred the antifreeze activity [7,35,36].

## DISCUSSION

The chemical synthesis of four types of xylomannans has been realized expeditiously. Thus, rapid preparation of the originally proposed [→4)-*β*-D-Man*p*-(1→4)-*β*-D-Xyl*p*-(1→]*_n_* xylomannan (Type I), 16-mer **7F**, 32-mer **8F** and 64-mer **9F** became available, together with the regioisomeric [→3)-*β*-D-Man*p*-(1→4)-*β*-D-Xyl*p*-(1→]*_n_* (Type II) 16-mer **13F**, the diastereomeric [→4)-*β*-L-Man*p*-(1→4)-*β*-D-Xyl*p*-(1→]*_n_* (Type III) 16-mer **15F** and the block-wise [→4)-*β*-D-Man*p*-(1→]*_m_*[→4)-*β*-D-Xyl*p*-(1→]*_n_* (Type IV) 32-mer **23F**. The synthesis features an IEGG strategy that permits extremely rapid extension of the glycan sizes [15,37–39]. In fact, a fully protected Type I xylomannan 256-mer (**11**) has been obtained from disaccharide building blocks **4A** and **4D** through only seven rounds of assemblies. The successful synthesis also testifies to the robustness of the Au(I)-catalysed glycosylation with glycosyl *ortho*-alkynylbenzoates [21], which have been applied in coupling with high-molecular-weight glycan fragments, as showcased by the semi-gram-scale construction of 128-mer **10** via a [64-mer + 64-mer] glycosylation. It is noteworthy that a 100-mer linear mannan and a 1080-mer linear arabinan have recently been synthesized using stepwise solid-phase synthesis and multiplicative liquid-phase assembly, respectively [40–43]. The present IEGG strategy offers an expeditious and scalable solution to the synthesis of structurally complex polysaccharides and thus facilitates ensuing functional studies.

The availability of the homogeneous xylomannans via chemical synthesis has allowed us to examine carefully the structure and activity of the natural antifreeze xylomannan, which was reported to occur in diverse cold-adapted species. In contrast to the proposed alternating structure of [→4)-*β*-D-Man*p*-(1→4)-*β*-D-Xyl*p*-(1→]*_n_*, comparison of the NMR spectroscopic data with those of our synthetic xylomannans strongly supports a hybrid of *β*-1,4-mannan and *β*-1,4-xylan, which might result from occasional incorporation of xylose and mannose units during the enzymatic synthesis of mannan and xylan, respectively. All the synthetic xylomannans did not show any TH inducing activity, including the block-wise 32-mer xylomannan **23F**, which matched well with the reported antifreeze isolates in NMR data. Xylan and mannan are frequently found in plants and fungi but rarely in animals [44]. From the viewpoint of glycan biosynthesis, which requires specific glycosyltransferases, synthesis of a same type of xylomannan in diverse species, as reported in the literature [5–8], is questionable. Taken together, our study disproves the occurrence of TH-active xylomannans in nature. The non-protein antifreeze substance in the natural isolates and the mechanism of action await further investigation.

## Supplementary Material

nwae296_Supplemental_File

## References

[1] DeVries AL, Wohlschlag DE. Freezing resistance in some Antarctic fishes. Science 1969; 163: 1073–5. 10.1126/science.163.3871.1073 5764871

[2] Graether SP, Kuiper MJ, Gagné SM et al. β-helix structure and ice-binding properties of a hyperactive antifreeze protein from an insect. Nature 2000; 406: 325–8. 10.1038/35018610 10917537

[3] Sun T, Lin F-H, Campbell RL et al. An antifreeze protein folds with an interior network of more than 400 semi-clathrate waters. Science 2014; 343: 795–8. 10.1126/science.1247407 24531972

[4] Bar Dolev M, Braslavsky I, Davies PL. Ice-binding proteins and their function. Annu Rev Biochem 2016; 85: 515–42. 10.1146/annurev-biochem-060815-014546 27145844

[5] Walters KR, Serianni AS, Sformo T et al. A nonprotein thermal hysteresis-producing xylomannan antifreeze in the freeze-tolerant Alaskan beetle *Upis ceramboides*. Proc Natl Acad Sci USA 2009; 106: 20210–5. 10.1073/pnas.0909872106 19934038 PMC2787118

[6] Walters KR, Serianni AS, Voituron Y et al. A thermal hysteresis-producing xylomannan glycolipid antifreeze associated with cold tolerance is found in diverse taxa. J Comp Physiol B 2011; 181: 631–40. 10.1007/s00360-011-0552-8 21279720

[7] Larson DJ, Middle L, Vu H et al. Wood frog adaptations to overwintering in Alaska: new limits to freezing tolerance. J Exp Biol 2014; 217: 2193–200. 24737762 10.1242/jeb.101931

[8] Duman JG . Animal ice-binding (antifreeze) proteins and glycolipids: an overview with emphasis on physiological function. J Exp Biol 2015; 218: 1846–55. 10.1242/jeb.116905 26085662

[9] Murray KA, Gibson MI. Chemical approaches to cryopreservation. Nat Rev Chem 2022; 6: 579–93. 10.1038/s41570-022-00407-4 PMC9294745 35875681

[10] Crich D, Rahaman MY. Synthesis and structural verification of the xylomannan antifreeze substance from the freeze-tolerant Alaskan beetle *Upis ceramboides*. J Org Chem 2011; 76: 8611–20. 10.1021/jo201780e 21955117 PMC3204896

[11] Ishiwata A, Sakurai A, Nishimiya Y et al. Synthetic study and structural analysis of the antifreeze agent xylomannan from *Upis ceramboides*. J Am Chem Soc 2011; 133: 19524–35. 10.1021/ja208528c 22029271

[12] Zhang W, Meredith R, Yoon M-K et al. Synthesis and *O*-glycosidic linkage conformational analysis of ^13^C-labeled oligosaccharide fragments of an antifreeze glycolipid. J Org Chem 2019; 84: 1706–24. 10.1021/acs.joc.8b01411 30624062 PMC8224223

[13] Lim YJ . Studies undertaken towards the total synthesis of antifreeze compounds based on the xylomannan antifreeze from the Alaskan beetle Upis ceramboides. Ph.D. Thesis. University of Alberta, Edmonton, Canada, 2019.

[14] Wang S, Yang Y, Zhu Q et al. Chemical synthesis of polysaccharides. Curr Opin Chem Biol 2022; 69: 102154. 10.1016/j.cbpa.2022.102154 35526499

[15] Zhu Q, Shen Z, Chiodo F et al. Chemical synthesis of glycans up to a 128-mer relevant to the *O*-antigen of *Bacteroides vulgatus*. Nat Commun 2020; 11: 4142. 10.1038/s41467-020-17992-x 32811831 PMC7434892

[16] Boltje TJ, Buskas T, Boons G-J. Opportunities and challenges in synthetic oligosaccharide and glycoconjugate research. Nat Chem 2009; 1: 611–22. 10.1038/nchem.399 20161474 PMC2794050

[17] Nigudkar SS, Demchenko AV. Stereocontrolled 1,2-*cis* glycosylation as the driving force of progress in synthetic carbohydrate chemistry. Chem Sci 2015; 6: 2687–704. 10.1039/C5SC00280J 26078847 PMC4465199

[18] Crich D, Sun S. Direct synthesis of β-mannopyranosides by the sulfoxide method. J Org Chem 1997; 62: 1198–9. 10.1021/jo962345z

[19] Christensen HM, Oscarson S, Jensen HH. Common side reactions of the glycosyl donor in chemical glycosylation. Carbohydr Res 2015; 408: 51–95. 10.1016/j.carres.2015.02.007 25862946

[20] Garegg PJ, Hultberg H, Wallin S. A novel, reductive ring-opening of carbohydrate benzylidene acetals. Carbohydr Res 1982; 108: 97–101. 10.1016/S0008-6215(00)81894-7

[21] Yu B . Gold(I)-catalyzed glycosylation with glycosyl *o*-alkynylbenzoates as donors. Acc Chem Res 2018; 51: 507–16. 10.1021/acs.accounts.7b00573 29297680

[22] Li Y, Manickam G, Ghoshal A et al. More efficient palladium catalyst for hydrogenolysis of benzyl groups. Synth Commun 2006; 36: 925–8. 10.1080/00397910500466199

[23] Crawford C, Oscarson S. Optimized conditions for the palladium-catalyzed hydrogenolysis of benzyl and naphthylmethyl ethers: preventing saturation of aromatic protecting groups. Eur J Org Chem 2020; 2020: 3332–7. 10.1002/ejoc.202000401

[24] Biely P, VrŠAnskÁ M. Synthesis and hydrolysis of 1,3-β-xylosidic linkages by endo-1,4-β-xylanase of *Cryptococcus albidus*. Eur J Biochem 1983; 129: 645–51. 10.1111/j.1432-1033.1983.tb07098.x 6825681

[25] Bellich B, Jou IA, Buriola C et al. The biofilm of *Burkholderia cenocepacia* H111 contains an exopolysaccharide composed of L-rhamnose and L-mannose: structural characterization and molecular modelling. Carbohydr Res 2021; 499: 108231. 10.1016/j.carres.2020.108231 33440288 PMC9638112

[26] Jansson P-E, Savitri Kumar N, Lindberg B. Structural studies of a polysaccharide (S-88) elaborated by *Pseudomonas* ATCC 31554. Carbohydr Res 1986; 156: 165–72. 10.1016/S0008-6215(00)90108-3 3815405

[27] Chowdhury TA, Lindberg B, Lindquist U et al. Structural studies of an extracellular polysaccharide (S-198) elaborated by *Alcaligenes* ATCC 31853. Carbohydr Res 1987; 161: 127–32. 10.1016/0008-6215(87)84011-9 2790839

[28] van den Bos LJ, Dinkelaar J, Overkleeft HS et al. Stereocontrolled synthesis of β-D-mannuronic acid esters: synthesis of an alginate trisaccharide. J Am Chem Soc 2006; 128: 13066–7. 10.1021/ja064787q 17017782

[29] Codée JDC, van den Bos LJ, de Jong A-R et al. The stereodirecting effect of the glycosyl C5-carboxylate ester: stereoselective synthesis of β-mannuronic acid alginates. J Org Chem 2008; 74: 38–47. 10.1021/jo8020192 19035740

[30] Tang S-L, Pohl NLB. Automated solution-phase synthesis of β-1,4-mannuronate and β-1,4-mannan. Org Lett 2015; 17: 2642–5. 10.1021/acs.orglett.5b01013 25955886 PMC4460921

[31] Pan D, Zhang L, Hua Q et al. Highly convergent synthesis of a β-mannuronic acid alginate hexadecasaccharide. Org Biomol Chem 2019; 17: 6174–7. 10.1039/C9OB01254K 31168536

[32] Zhang L, Zhang Y, Hua Q et al. Promoter-controlled synthesis and antigenic evaluation of mannuronic acid alginate glycans of *Pseudomonas aeruginosa*. Org Lett 2022; 24: 8381–6. 10.1021/acs.orglett.2c03439 36346693

[33] Garrozzo D, Impallomeni G, Spina E et al. Matrix-assisted laser desorption/ionization mass spectrometry of polysaccharides. Rapid Commun Mass Spectrom 1995; 9: 937–41. 10.1002/rcm.1290091014

[34] Nicolardi S, Joseph AA, Zhu Q et al. Analysis of synthetic monodisperse polysaccharides by wide mass range ultrahigh-resolution MALDI mass spectrometry. Anal Chem 2021; 93: 4666–75. 10.1021/acs.analchem.1c00239 33667082 PMC8034773

[35] Duman JG, Serianni AS. The role of endogenous antifreeze protein enhancers in the hemolymph thermal hysteresis activity of the beetle *Dendroides canadensis*. J Insect Physiol 2002; 48: 103–11. 10.1016/S0022-1910(01)00150-0 12770137

[36] Liu Z, Zheng X, Wang J. Bioinspired ice-binding materials for tissue and organ cryopreservation. J Am Chem Soc 2022; 144: 5685–701. 10.1021/jacs.2c00203 35324185

[37] Wang Q, Qu Y, Tian H et al. Iterative binomial synthesis of monodisperse polyfluorenes up to 64-mers and their chain-length-dependent properties. Macromolecules 2011; 44: 1256–60. 10.1021/ma102954h

[38] Barnes JC, Ehrlich DJC, Gao AX et al. Iterative exponential growth of stereo- and sequence-controlled polymers. Nat Chem 2015; 7: 810–5. 10.1038/nchem.2346 26391080

[39] Yin J, Choi S, Pyle D et al. Backbone engineering of monodisperse conjugated polymers via integrated iterative binomial synthesis. J Am Chem Soc 2023; 145: 19120–8. 10.1021/jacs.3c08143 37603817 PMC10472507

[40] Joseph AA, Pardo-Vargas A, Seeberger PH. Total synthesis of polysaccharides by automated glycan assembly. J Am Chem Soc 2020; 142: 8561–4. 10.1021/jacs.0c00751 32338884 PMC7304863

[41] Yao W, Xiong D-C, Yang Y et al. Automated solution-phase multiplicative synthesis of complex glycans up to a 1,080-mer. Nat Synth 2022; 1: 854–63. 10.1038/s44160-022-00171-9

[42] Qin X, Xu C, Liu M et al. Synthesis of branched arabinogalactans up to a 140-mer from *Panax notoginseng* and their anti-pancreatic-cancer activity. Nat Synth 2024; 3: 245–55. 10.1038/s44160-023-00428-x

[43] Ma Y, Zhang Y, Huang Y et al. One-pot assembly of mannose-capped lipoarabinomannan motifs up to 101-mer from the *mycobacterium tuberculosis* cell wall. J Am Chem Soc 2024; 146: 4112–22. 10.1021/jacs.3c12815 38226918

[44] BeMiller JN . Occurrence and significance. In: Fraser-Reid BO, Tatsuta K, Thiem J (eds). Glycoscience: Chemistry and Chemical Biology. Berlin Heidelberg: Springer, 2008, 1435–44.

