# Supplementary material for: Expeditious chemical synthesis of xylomannans disproves the proposed antifreeze activities
Source: Natl Sci Rev. 2024 Aug 23;11(10):nwae296. doi: 10.1093/nsr/nwae296 (PMC11418650; doi:10.1093/nsr/nwae296)
Supplement: nwae296_Supplemental_File [file nwae296_supplemental_file.pdf]

Supporting information

Expeditious chemical synthesis of xylomannans disproves the proposed antifreeze activities

Qian Zhu,[1] Simone Nicolardi,[2] Yuanguang Wang,[3] Yasong Liu,[1] Peng Xu,[1] Jianjun Wang,[3] Dapeng Zhu,[4,*] Biao Yu[1,*]

Affiliations:

[1]State Key Laboratory of Chemical Biology, Shanghai Institute of Organic Chemistry, University of Chinese Academy of Sciences, Chinese Academy of Sciences, Shanghai 200032, China

[2]Center for Proteomics and Metabolomics, Leiden University Medical Center, Leiden 2333ZA, The Netherlands

[3]Key Laboratory of Green Printing, Beijing National Laboratory for Molecular Science, Institute of Chemistry, Chinese Academy of Sciences, Beijing 100190, China

[4]Institute of Translational Medicine, Shanghai Jiao Tong University, Shanghai 200240, China

Correspondence to: zhudapeng@sjtu.edu.cn; byu@sioc.ac.cn

Contents

1. General methods for synthesis and characterization 3
2. Synthesis of [→4)-β-D-Man*p*-(1→4)-β-D-Xyl*p*-(1→] xylomannans 4
2.1 Preparation of monosaccharide building blocks **1** and **2** 4
2.2 Preparation of disaccharide building blocks **4A** and **4D** 6
2.3 Synthesis of fully protected glycans **5-11** 9
General procedure for the Au(I)-catalyzed glycosylation (general procedure I) 10
General procedure for the preparation of glycosyl acceptors (general procedure II) 10
General procedure for the preparation of glycosyl *o*-hexynylbenzoate donors (general procedure III) 11
2.4 Details for the purification and characterization of fully protected glycans **5-11** 20
2.5 Preparation of free glycans **4F-9F** 26
2.6 Spectral comparison of synthetic 16-mer **7F** with previously reported xylomannans 31
3. Synthesis of [→3)-β-D-Man*p*-(1→4)-β-D-Xyl*p*-(1→] xylomannan **13F** 33
3.1 Preparation of disaccharide building blocks **12A** and **12D** 33
3.2 Synthesis of fully protected 16-mer **13** 35
3.3 Preparation of free 16-mer **13F** 40
3.4 Spectral comparison of synthetic 16-mer **13F** with the natural xylomannan 40
4. Synthesis of [→4)-β-L-Man*p*-(1→4)-β-D-Xyl*p*-(1→] xylomannan **15F** 41
4.1 Preparation of disaccharide building blocks **14A** and **14D** 41
4.2 Synthesis of fully protected 16-mer **15** 44
4.3 Preparation of free 16-mer **15F** 48
4.4 Spectral comparison of synthetic 16-mer **15F** with the natural xylomannan 48
5. Synthesis of block-wise xylomannan 32-mer **23F** 50
5.1 Preparation of xylan 6-mer donor **21** 51
5.2 Preparation of fully protected block-wise 32-mer **23** 57
5.3 Preparation of free block-wise 32-mer **23F** 64
5.4 Spectral comparison of synthetic block-wise 32-mer **23F** with the natural xylomannan 65
6. Analysis of the NMR and mass spectroscopic data of the synthetic glycans 66
7. Antifreeze activity measurement. 68
References 70
^{1}H NMR, ^{13}C NMR, 2D NMR, and Mass spectra 71

1. General methods for synthesis and characterization

Reactions were carried out in glassware, unless otherwise noted. Crushed 4Å or 5Å molecular sieves were activated through flame-drying under high vacuum immediately prior to use. All chemicals were purchased as reagent grade and used without further purification, unless otherwise noted. Analytical thin-layer chromatography was performed using Merck pre-coated silica gel 60 F-254 plates; compound spots were visualized by UV light (254 nm) or immersion into a solution of 5% H_2SO_4 in ethanol followed by hot air gun heating. Column chromatography was performed on silica gel (200-300 mesh). Gel filtration was performed on Sephadex LH-60, LH-20, or G25. Optical rotations were obtained on Anton Paar MCP 5500 polarimeter at 589 nm (sodium D-line). NMR spectra were recorded on Bruker AM 400, Varian 500, or Agilent 600 MHz NMR spectrometers. Chemical shifts (δ) and coupling constants are reported in ppm and Hz, respectively. ^{1}H NMR spectra were calibrated using Me_4Si (0 ppm, $CDCl_3$) or residual HDO (4.60 ppm at 40 °C; 4.79 ppm at 25 °C). Spectra recorded with 20 mM Na_3PO_4 buffered D_2O were calibrated using residual HDO (4.80 ppm at 40 °C, pH = 7.5). ^{13}C NMR spectra were referenced using Me_4Si (0.0 ppm, $CDCl_3$) or external 2,2-dimethyl-2-silapentane-5-sulfonic acid (DSS) (0.0 ppm, D_2O). Assignments of resonances in ^{1}H and ^{13}C NMR spectra were done using ^{1}H-^{1}H COSY, ^{1}H-^{13}C HSQC, and ^{1}H-^{13}C HMBC experiments. The anomeric configuration of glycosylation products was determined by homonuclear and heteronuclear coupling constants. Splitting patterns are indicated as s (singlet), d (doublet), t (triplet), q (quartet), and br s (broad singlet) for ^{1}H NMR data. HRMS experiments were performed on a Bruker maXis 4G spectrometer. Recycle preparative gel permeation chromatography (GPC) were performed on a JAI-9201 series HPLC (columns: 1H and 3H, UV detector: JACSO MD2010, eluting solvent: $CHCl_3$, flow rate: 3.5 mL/min, pressure: 21 MPa). SEM measurements were performed on a NOVA NANOSEM 450 (FEI) instrument operating with an accelerating voltage of 3.0 kV; the samples were prepared on clean glass and dried at room temperature for 48 h, and were then coated with a thin film of gold before measuring.

2. Synthesis of [→4)-β-D-Man*p*-(1→4)-β-D-Xyl*p*-(1→] xylomannans

2.1 Preparation of monosaccharide building blocks 1 and 2

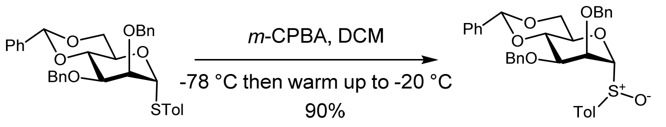

1

To a solution of 4,6-*O*-benzylidene-2,3-*O*-benzyl-1-*p*-methylphenylthio-α-D-mannopyranoside[1] (27.1 g, 48.9 mmol) in CH_2Cl_2 (400 mL) was added a solution of 3-chloroperoxybenzoic acid (*m*-CPBA) (77%, 8.86 g, 39.5 mmol) in CH_2Cl_2 (88 mL) dropwise at -78 °C, resulting in a white slurry. The mixture was stirred for 2 hours, during which time the temperature was allowed to warm up to -20 °C slowly. Another portion of *m*-CPBA (77%, 1.77 g, 7.9 mmol) in CH_2Cl_2 (20 mL) was added at -78 °C; and the reaction mixture was kept stirring for 0.5 hour. The reaction mixture was warmed up to -20 °C and was then quenched by pouring into an aqueous NaOH solution (7.0 g, 300 mL). The organic layer was separated, washed with saturated $NaHCO_3$ solution and brine, dried over anhydrous Na_2SO_4, and concentrated. The residue was purified by silica gel chromatography (petroleum ether/ethyl acetate, 1:4, R_f = 0.3) to give mannopyranosyl sulfoxide **1** as a white solid (25.2 g, 90%): $[\alpha]_D^{25}$ = -53.0 (*c* 1.0, $CHCl_3$); ^1H NMR (600 MHz, $CDCl_3$) δ 7.53–7.46 (m, 2 H), 7.45–7.36 (m, 5 H), 7.36–7.29 (m, 7 H), 7.29–7.27 (m, 1 H), 7.25–7.21 (m, 2 H), 5.63 (s, 1 H), 4.82 (d, J = 12.1 Hz, 1 H), 4.66 (d, J = 12.1 Hz, 1 H), 4.64–4.54 (m, 2 H), 4.47 (d, J = 1.3 Hz, 1 H), 4.41 (dd, J = 3.2, 1.4 Hz, 1 H), 4.35–4.24 (m, 2 H), 4.20 (dd, J = 10.3, 4.9 Hz, 1 H), 4.07 (td, J = 9.2, 4.7 Hz, 1 H), 3.74 (t, J = 10.1 Hz, 1 H), 2.45 (s, 3 H); ^{13}C NMR (151 MHz, $CDCl_3$) δ 142.4, 138.4, 138.3, 137.5, 137.4, 130.3, 129.1, 128.5, 128.42, 128.40, 128.38, 128.35, 128.1, 128.0, 127.9, 127.8, 126.2, 126.2, 124.6, 101.8, 97.7, 78.2, 77.4, 77.2, 77.0, 76.4, 73.6, 73.4, 73.0, 70.2, 68.4, 21.7; HRMS (ESI) calcd for $C_{34}H_{34}O_6SNa$ $[M + Na]^+$ 593.1974, found 593.1970.

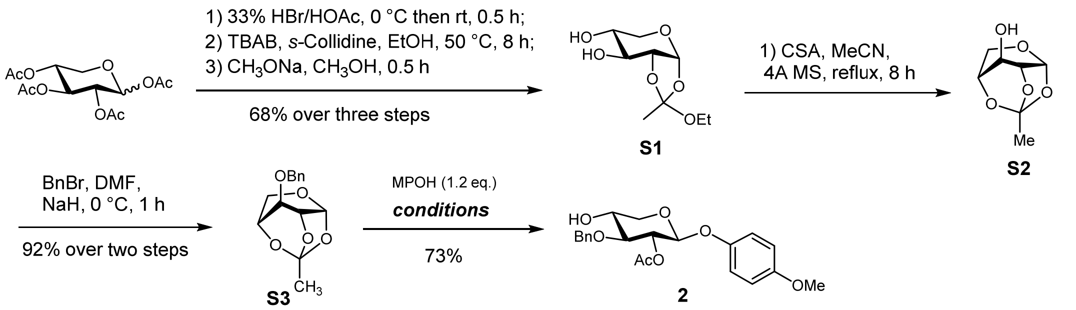

Entry	Conditions	Yield
1	$InBr_3$ (1.0), $PhCF_3$, 100 mM, 4A MS, rt, 1 h	50% [a]
2	$InBr_3$ (0.3), $PhCF_3$, 100 mM, 4A MS, rt, 8 h	51% [a]
3	$BF_3 \cdot Et_2O$ (0.2), DCM, 100 mM, 4A MS, 0 °C, 1 h	74% [b]
4	$BF_3 \cdot Et_2O$ (0.2), DCM, 100 mM, 4A MS, -30 °C, 1 h	75% [b]
5	$BF_3 \cdot Et_2O$ (0.2), DCM, 100 mM, 4A MS, -78 °C, 3 h	75% [b]
6	$BF_3 \cdot Et_2O$ (0.2), DCM, 220 mM, 4A MS, -30 °C, 1 h	73% [c]

[a] isolated yield
[b] Yield determined by ^1H NMR with mesitylene as an internal standard
[c] 17.5 g sacle

Figure S1. Preparation of xyloside building block **2**.

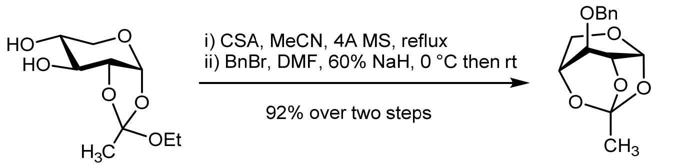

S3

To a mixture of 1,2-*O*-(1-ethoxylethylidene)-α-D-xylopyranoside[2] (9.20 g, 41.8 mmol) and 4 Å MS (15 g) in anhydrous MeCN (100 mL), was added CSA (291 mg, 1.25 mmol) at room temperature. The mixture was refluxed for 8 hours, to which NaOH (100 mg, 2.51 mmol) was added. The mixture was filtered through a pad of Celite. The filtrate was concentrated *in vacuo*. The resulting residue was azeotropically dried with toluene and used for the next step directly.

To a mixture of the above residue and benzyl bromide (13.8 mL, 116 mmol) in anhydrous *N,N*-dimethylformamide (100 mL) was added NaH (60% in grease, 4.63 g, 116 mmol) at 0 °C. The mixture was stirred at room temperature for 1 hour, and was then quenched with MeOH at 0 ºC carefully. The resulting mixture was concentrated *in vacuo*. The residue was purified by silica gel column chromatography (petroleum ether/ethyl acetate, 2:1, R_f = 0.6) to give orthoester **S3** as a colorless syrup (11.2 g, 92% over two steps): $[\alpha]_D^{25}$ = 38.2 (*c* 2.0, $CHCl_3$); ^1H NMR (500 MHz, $CDCl_3$) δ 7.40–7.30 (m, 5 H), 5.74 (d, *J* = 4.9 Hz, 1 H), 4.68 (d, *J* = 12.2 Hz, 1 H), 4.62 (d, *J* = 12.2 Hz, 1 H), 4.38 (dt, *J* = 5.0, 2.0 Hz, 1 H), 4.20–4.13 (m, 3 H), 3.94 (dd, *J* = 4.5, 2.0 Hz, 1 H), 1.63 (s, 3 H); ^{13}C NMR (126 MHz, $CDCl_3$) δ 137.6, 128.8, 128.3, 127.9, 118.4, 98.1, 77.4, 77.2, 76.9, 72.5, 72.4, 71.5, 70.0, 65.7, 20.3; HRMS (ESI) calcd for $C_{14}H_{16}O_5Na$ $[M + Na]^+$ 265.1076, found 265.1074. Data are in agreement with those reported in literature.[3]

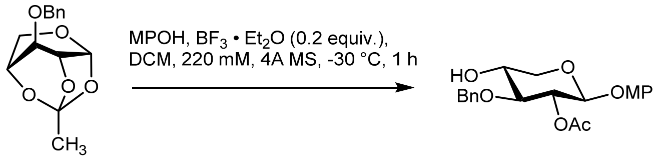

S3 **2**

To a solution of orthoester **S3** (17.5 g, 66.3 mmol) and 4-methoxyphenol (MPOH) (9.86 g, 79.6 mmol) in CH_2Cl_2 (300 mL) was added freshly activated 4 Å molecular sieves (30 g). The mixture was kept stirring at room temperature for 0.5 hour; $BF_3{\cdot}Et_2O$ (1.64 mL, 13.3 mmol) was added at -30 °C. The mixture was kept stirring for 1 hour; triethylamine (20 mL) was added to quench the reaction. The mixture was warmed up to room temperature, filtered through a pad of Celite, and washed with saturated $NaHCO_3$ solution. The filtrate was dried with anhydrous Na_2SO_4, filtered, and concentrated *in vacuo*. The residue was purified by silica gel column chromatography (petroleum ether/ethyl acetate, 1:2, R_f = 0.4) to give xylopyranoside **2** as a white solid (18.8 g, 73%): $[\alpha]_D^{25}$ = -28.2 (*c* 1.0, $CHCl_3$); ^1H NMR (500 MHz, $CDCl_3$) δ 7.38–7.27 (m, 5 H), 6.97–6.92 (m, 2 H), 6.84–6.77 (m, 2 H), 5.19 (dd, *J* = 7.5, 5.9 Hz, 1 H), 4.96 (d, *J* = 5.9 Hz, 1 H), 4.79 (d, *J* = 11.7 Hz, 1 H), 4.69 (d, *J* = 11.7 Hz, 1 H), 4.13 (dd, *J* = 11.7, 4.3 Hz, 1 H), 3.83 (tdd, *J* = 7.5, 5.6, 4.3 Hz, 1 H), 3.75 (s, 3 H), 3.57 (t, *J* = 7.3 Hz, 1 H), 3.40 (dd, *J* = 11.7, 7.9 Hz, 1 H), 2.53 (d, *J* = 5.7 Hz, 1 H), 2.07 (s, 3 H); ^{13}C NMR (126 MHz, $CDCl_3$) δ 169.6, 155.5, 151.1, 138.1, 128.7, 128.1, 127.9, 118.5, 114.7, 100.1, 80.2, 77.4, 77.2, 76.9, 73.8, 71.3, 68.8, 64.3, 55.8, 21.1; HRMS (ESI) calcd for $C_{21}H_{24}O_7Na$ $[M + Na]^+$ 411.1420, found 411.1420.

2.2 Preparation of disaccharide building blocks 4A and 4D

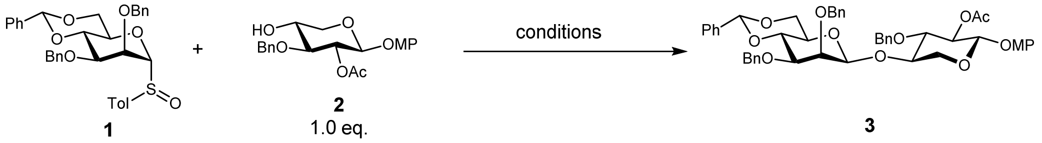

Entry	Donor (eq.)	Conditions	Yield[a]
1	**1** (1.20)	Tf_2O (1.2), TTBP (1.6), DCM, 48 mM, 4A MS, -78 °C → -20°C	67%
2	**1** (1.20)	Tf_2O (1.2), TTBP (1.6), DCM / 1-Octene, 125 mM, 4A MS, -78 °C → -20°C	86%
3	**1** (1.20)	Tf_2O (1.2), TTBP (1.6), DCM / 1-Octene, 105 mM, 4A MS, -78 °C → -20°C	83%[a]

[a] 25 grams scale

To a stirred solution of sulfoxide donor **1** (25.2 g, 44.2 mmol, azeotropically dried with toluene), 2,4,6-tri-*tert*-butylpyrimidine (17.5 g, 70.7 mmol), and 4 Å molecular sieves (30 g) in a mixed solvent of 1-octene/CH_2Cl_2 (71 mL/283 mL) was added Tf_2O (7.43 mL, 44.2 mmol) at -78 °C under argon atmosphere. After the reaction mixture was stirred at -78 °C for 30 minutes, a solution of acceptor **2** (14.3 g, 36.8 mmol, azeotropically dried with toluene) in CH_2Cl_2 (30 mL) was introduced by syringe over 15 minutes. Stirring was maintained for another 1 hour at -78 °C before the reaction temperature was allowed to warm to -20 °C slowly. Triethylamine (30 mL) was added to the reaction mixture, and the resultant mixture was filtered through a pad of Celite. The filtrate was washed with saturated $NaHCO_3$ solution and brine, dried over anhydrous Na_2SO_4, and concentrated *in vacuo*. The residue was purified by silica gel chromatography (petroleum ether/ethyl acetate, 1:3, Rf = 0.4) to give disaccharide **3** as a white foam (25.0 g, 83%): $[\alpha]_D^{25}$ = -64.5 (*c* 1.0, $CHCl_3$); ^{1}H NMR (500 MHz, $CDCl_3$) δ 7.51–7.44 (m, 4 H), 7.40–7.34 (m, 3 H), 7.33–7.25 (m, 14 H), 6.98–6.94 (m, 2 H), 6.85–6.80 (m, 2 H), 5.59 (s, 1 H), 5.15 (dd, J = 7.0, 5.4 Hz, 1 H), 5.05 (d, J = 5.4 Hz, 1 H), 4.96–4.90 (m, 2 H), 4.87 (d, J = 11.8 Hz, 1 H), 4.73 (d, J = 12.2 Hz, 2 H), 4.61 (d, J = 12.5 Hz, 1 H), 4.57 (d, J = 1.0 Hz, 1 H), 4.20 (dd, J = 10.4, 4.8 Hz, 1 H), 4.17 (t, J = 9.6 Hz, 1 H), 4.11 (dd, J = 12.2, 4.0 Hz, 1 H), 3.96 (td, J = 6.6, 4.0 Hz, 1 H), 3.90 (dd, J = 3.2, 1.0 Hz, 1 H), 3.80–3.74 (m, 5 H), 3.59 (dd, J = 9.9, 3.2 Hz, 1 H), 3.44 (dd, J = 12.2, 6.8 Hz, 1 H), 3.25 (td, J = 9.7, 4.9 Hz, 1 H), 1.99 (s, 3 H); ^{13}C NMR (126 MHz, $CDCl_3$) δ 169.7, 155.3, 150.9, 138.40, 138.38, 138.3, 137.5, 128.9, 128.5, 128.34, 128.27, 128.2, 127.7, 127.61, 127.59, 127.5, 126.1, 118.4, 114.6, 101.4, 100.9, 99.6, 78.6, 77.8, 77.2, 77.0, 76.8, 76.5, 75.5, 74.9, 73.2, 72.5, 70.3, 68.5, 67.6, 61.5, 55.7, 21.0; HRMS (ESI) calcd for $C_{48}H_{50}O_{12}Na$ $[M + Na]^+$ 841.3200, found 841.3198. The anomeric configuration was verified by 2D NOESY.

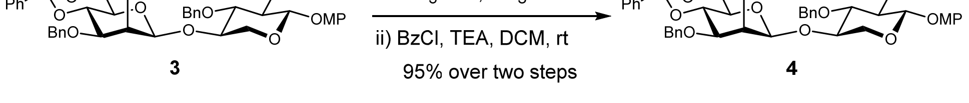

To a solution of disaccharide **3** (24.9 g, 30.4 mmol) in methanol (100 mL) was added CH_3ONa (164 mg, 3.04 mmol). The resulting mixture was refluxed for 30 minutes and was then concentrated *in vacuo*. The residue was diluted with CH_2Cl_2, and washed with H_2O two times. The organic layer was dried over anhydrous Na_2SO_4 and then concentrated *in vacuo* to give a residue (petroleum ether/ethyl acetate, 1:2, R_f = 0.6).

To a solution of the above residue, 4-dimethylaminopyridine (371 mg, 3.04 mmol), and triethylamine (42 mL, 304 mmol) in CH_2Cl_2 (200 mL) was added benzoyl chloride (10.5 mL, 91.2 mmol) at 0 °C. The mixture was stirred at room temperature for 2 hours, and was then quenched by

carful addition of methanol (5 mL) at 0 °C. The resultant mixture was poured into saturated $NaHCO_3$ solution and the aqueous layer was extracted with CH_2Cl_2 two times. The combined organic phase was dried over anhydrous $NaSO_4$, and concentrated *in vacuo*. The residue was purified by silica gel chromatography (petroleum ether/ethyl acetate, 3:1, R_f = 0.4) to give benzoate **4** as a white foam (26.8 g, 95% over two steps): $[\alpha]_D^{25}$ = -52.6 (*c* 1.0, $CHCl_3$); ^{1}H NMR (500 MHz, $CDCl_3$) δ 8.08–8.00 (m, 2 H), 7.55–7.49 (m, 1 H), 7.49–7.45 (m, 2 H), 7.44–7.41 (m, 2 H), 7.40–7.34 (m, 5 H), 7.32–7.23 (m, 10 H), 7.21–7.16 (m, 3 H), 7.00–6.93 (m, 2 H), 6.83–6.75 (m, 2 H), 5.48 (s, 1 H), 5.40 (dd, *J* = 6.4, 5.1 Hz, 1 H), 5.23 (d, *J* = 5.0 Hz, 1 H), 4.91–4.82 (m, 3 H), 4.80 (d, *J* = 11.8 Hz, 1 H), 4.73 (d, *J* = 12.5 Hz, 1 H), 4.62 (d, *J* = 12.5 Hz, 1 H), 4.56 (d, *J* = 1.0 Hz, 1 H), 4.19 (dd, *J* = 12.2, 3.7 Hz, 1 H), 4.11 (dd, *J* = 10.4, 4.8 Hz, 1 H), 4.06 (t, *J* = 9.5 Hz, 1 H), 4.02–3.92 (m, 2 H), 3.90 (dd, *J* = 3.2, 1.0 Hz, 1 H), 3.75 (s, 3 H), 3.60–3.46 (m, 3 H), 3.19 (td, *J* = 9.7, 4.8 Hz, 1 H); ^{13}C NMR (126 MHz, $CDCl_3$) δ 165.6, 155.5, 151.1, 138.6, 138.5, 138.3, 137.7, 133.2, 130.2, 123.0, 129.0, 128.6, 128.5, 128.4, 128.32, 128.28, 128.0, 127.74, 127.71, 127.69, 127.67, 126.2, 118.7, 114.7, 101.5, 101.3, 99.8, 78.6, 78.0, 77.4, 77.2, 76.9, 76.7, 76.1, 75.2, 73.3, 72.6, 70.8, 68.6, 67.7, 61.6, 55.8; HRMS (ESI) calcd for $C_{53}H_{52}O_{12}Na$ $[M + Na]^+$ 903.3356, found 903.3353.

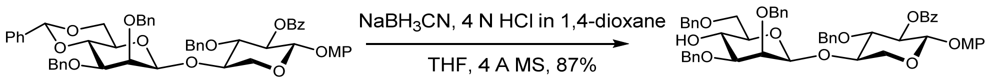

4 **4A**

To a solution of benzylidene acetal **4** (26.5 g, 30 mmol) in dry tetrahydrofuran (300 mL) were added 4 Å molecular sieves (30 g) and sodium cyanoborohydride (18.9 g, 300 mmol) at room temperature. The mixture was kept stirring for 10 minutes at room temperature. Subsequently, a solution of HCl in dioxane (4 N, 97.5 mL) was slowly added over a period of 20 minutes at 0 °C. After the reaction mixture was stirred at 0 °C for 30 minutes, solid $NaHCO_3$ (30 g), EtOAc (300 mL), and saturated $NaHCO_3$ solution (300 mL) were added successively. The resulting mixture was filtered through a pad of Celite. The filtrate was washed with saturated $NaHCO_3$ solution, dried over anhydrous Na_2SO_4, filtered, and concentrate *in vacuo*. The residue was purified by silica gel column chromatography (petroleum ether/ethyl acetate, 1:2, R_f = 0.4) to give alcohol **4A** as a white foam (22.9 g, 87%): $[\alpha]_D^{25}$ = -43.6 (*c* 1.0, $CHCl_3$); ^{1}H NMR (500 MHz, $CDCl_3$)) δ 8.10–8.04 (m, 2 H), 7.51 (ddt, *J* = 8.8, 7.3, 1.3 Hz, 1 H), 7.40–7.09 (m, 23 H), 7.00–6.94 (m, 2 H), 6.83–6.76 (m, 2 H), 5.44 (dd, *J* = 6.4, 5.0 Hz, 1 H), 5.25 (d, *J* = 5.1 Hz, 1 H), 4.88 (d, *J* = 12.1 Hz, 1 H), 4.79 (s, 2 H), 4.74 (d, *J* = 12.1 Hz, 1 H), 4.60–4.52 (m, 2 H), 4.48 (s, 2 H), 4.44 (d, *J* = 11.8 Hz, 1 H), 4.22 (dd, *J* = 12.3, 3.7 Hz, 1 H), 4.05 (td, *J* = 6.0, 3.7 Hz, 1 H), 4.01 (t, *J* = 6.2 Hz, 1 H), 3.95 (td, *J* = 9.4, 1.9 Hz, 1 H), 3.90 (d, *J* = 3.0 Hz, 1 H), 3.76 (s, 3 H), 3.67 (dd, *J* = 10.3, 4.1 Hz, 1 H), 3.63–3.53 (m, 2 H), 3.38 (ddd, *J* = 9.8, 5.8, 4.1 Hz, 1 H), 3.33 (dd, *J* = 9.4, 2.9 Hz, 1 H), 2.68 (d, *J* = 1.9 Hz, 1 H); ^{13}C NMR (126 MHz, $CDCl_3$) δ 165.6, 155.4, 151.1, 138.7, 138.4, 138.1, 138.0, 133.2, 130.2, 130.0, 128.6, 128.54, 128.52, 128.4, 128.3, 128.23, 128.0, 127.93, 127.89, 127.83, 127.77, 127.6, 127.5, 118.7, 114.6, 100.6, 99.8, 81.5, 77.4, 77.2, 77.0, 76.9, 75.4, 75.2, 74.51, 74.45, 73.9, 73.0, 71.5, 70.9, 70.7, 68.5, 61.5, 55.8; HRMS (ESI) calcd for $C_{53}H_{54}O_{12}Na$ $[M + Na]^+$ 905.3513, found 903.3514.

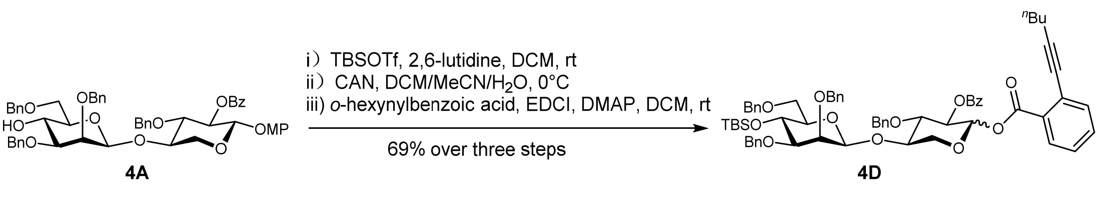

4A **4D**

To a solution of **4A** (3.24 g, 3.67 mmol) and 2,6-lutidine (2.14 mL, 18.4 mmol) in anhydrous CH_2Cl_2 (20 mL) was added *tert*-butyldimethylsilyl triflate (2.11 mL, 9.18 mmol) at 0 °C. The mixture was warmed up to room temperature and kept stirring for 1 hour; MeOH/Et_3N (1 mL/1 mL) was then added to quench the reaction. The mixture was washed with 1N HCl solution, saturated $NaHCO_3$ solution, and brine successively. The organic layer was dried over anhydrous Na_2SO_4, filtered, and concentrated *in vacuo*. The resulting residue was used in the next step directly.

To a solution of the above residue in CH_2Cl_2/CH_3CN/H_2O (20/40/0.4 mL) was added ceric ammonium nitrate (6.04 g, 11 mmol) at 0 °C. The mixture was stirred at 0 °C for 5 minutes, and was then poured into saturated $NaHCO_3$ solution. The aqueous layer was extracted with EtOAc three times. The combined organic layer was dried over anhydrous Na_2SO_4 and concentrated *in vacuo*. The residue was purified by silica gel chromatography (petroleum ether/ethyl acetate, 3:1, 0.5% Et_3N as additive, $R_f = 0.4$) to afford a brown foam, which was used in the next step directly.

To a mixture of the above foam (2.4 g, 2.70 mmol), *ortho*-hexynylbenzoic acid (817 mg, 4.04 mmol), 4-dimethylaminopyridine (823 mg, 6.74 mmol) in CH_2Cl_2 (20 mL) was added 1-(3-dimethylaminopropyl)-3-ethylcarbodiimide hydrochloride (EDCI) (1.29 g, 6.74 mmol) at room temperature. The mixture was stirred at room temperature for 8 hours, and was then poured into saturated $NaHCO_3$ solution. The aqueous layer was extracted with CH_2Cl_2 two times. The combined organic layer was dried over anhydrous Na_2SO_4, and concentrated *in vacuo*. The resulting residue was purified by silica gel chromatography (petroleum ether/ethyl acetate, 4:1, $R_f = 0.7$) to afford glycosyl donor **4D** as a white foam (2.73 g, 69% over three steps, α/β = 1:3.3): ^{1}H NMR (500 MHz, $CDCl_3$) δ 8.10–8.04 (m, 1.59 H), 7.97–7.92 (m, 0.38 H), 7.88 (dd, J = 8.0, 1.4 Hz, 1 H), 7.58–7.42 (m, 2.20 H), 7.39 (td, J = 7.6, 1.4 Hz, 0.71 H), 7.34 (m, 1H), 7.32–6.99 (m, 21 H), 6.61–6.53 (m, 0.23 H), 6.28 (d, J = 4.0 Hz, 0.76 H), 5.42 (t, J = 4.6 Hz, 0.77 H), 5.38 (dd, J = 9.5, 3.7 Hz, 0.23 H), 5.03 (d, J = 11.8 Hz, 0.24 H), 4.90–4.69 (m, 3 H), 4.65–4.45 (m, 4.22 H), 4.42 (dd, J = 11.9, 3.6 Hz, 1.50 H), 4.32–4.21 (m, 1 H), 4.18–4.10 (m, 1 H), 4.09–4.00 (m, 1 H), 3.94–3.67 (m, 4 H), 3.61 (dd, J = 11.1, 6.2 Hz, 0.26 H), 3.45–3.27 (m, 2.79 H), 2.46 (t, J = 7.1 Hz, 1.55 H), 2.37 (td, J = 7.1, 1.9 Hz, 0.45 H), 1.60 (dq, J = 8.7, 7.2 Hz, 1.53 H), 1.55–1.36 (m, 2.44 H), 0.93 (t, J = 7.3 Hz, 2.27 H), 0.88 (t, J = 7.3 Hz, 0.65 H), 0.01 (d, J = 2.3 Hz, 0.68 H), -0.01 (s, 0.79 H), -0.03 (s, 2.30 H), -0.05 (s, 2.28 H); ^{13}C NMR (126 MHz, $CDCl_3$) δ 165.60, 165.58, 164.3, 163.9, 139.00, 138.95, 138.8, 138.7, 138.6, 138.18, 138.15, 138.1, 134.9, 134.6, 133.2, 132.20, 132.18, 130.9, 130.8, 130.5, 130.33, 130.30, 130.0, 129.7, 129.6, 128.7, 128.41, 128.37, 128.35, 128.3, 128.22, 128.20, 128.1, 128.04, 127.95, 127.9, 127.8, 127.73, 127.71, 127.67, 127.61, 127.59, 127.56, 127.51, 127.48, 127.4, 127.29, 127.25, 127.23, 127.18, 125.7, 100.0, 99.5, 97.2, 97.1, 92.6, 90.9, 82.9, 82.4, 79.3, 79.2, 78.1, 77.6, 77.5, 77.4, 77.2, 76.9, 75.7, 75.1, 75.0, 74.9, 74.6, 74.4, 74.1, 73.8, 73.6, 73.5, 73.0, 71.5, 71.3, 71.2, 70.0, 69.8, 68.9, 68.2, 68.0, 62.1, 61.3, 30.8, 30.7, 26.1, 26.0, 22.2, 22.1, 19.68, 19.67, 18.24, 18.22, 13.80, 13.76, -3.67, -3.70, -4.77, -4.83; HRMS (ESI) calcd for $C_{65}H_{74}O_{12}SiNa$ $[M + Na]^+$ 1097.4847, found 1097.4847.

2.3 Synthesis of fully protected glycans 5-11

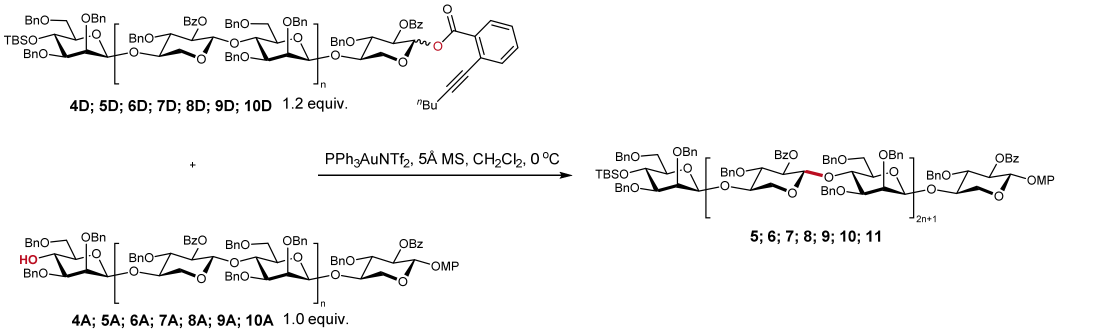

n	Substrates	PPh_3AuNTf_2 (eq.)[b]	DCM/5Å MS	*c*	time	Method for purification	Product
0	**4D** (10.5 g) + **4A** (8.62 g)	722 mg (0.10)	100 mL/10 g	100 mM	3 h	EA/PE = 1:3, R_f = 0.4	**5**, 15 g, 87%
1	**5D** (6.51 g) + **5A** (5.30 g)	477 mg (0.20)	51 mL/5 g	63 mM	3 h	EA/DCM/PE = 1:1:2, R_f = 0.4-0.5[a]	**6**, 10 g, 94%
3	**6D** (4.20 g) + **6A** (3.60 g)	168 mg (0.20)	30 mL/6 g	38 mM	2 h	EA/DCM/PE = 1:1:1.5, R_f = 0.55-0.45 [a]	**7**, 6.7 g, 94%
7	**7D** (2.85 g) + **7A** (2.30 g)	137 mg (0.50)	21 mL/10.3 g	18 mM	7 h	EA/DCM/PE = 3.5:2:5, R_f = 0.1-0.4 [a]	**8**, 4.5 g, 95%
15	**8D** (823 mg) + **8A** (600 mg)	52 mg (1.44)	6 mL/3 g	8.2 mM	11 h	Gel permeation chromatography	**9**, 1.1 g, 90%
31	**9D** (492 mg) + **9A** (407 mg)	62 mg (5.03)	8 mL/3 g	2.08 mM	16 h	Gel permeation chromatography	**10**, 594 mg, 73%
63	**10D** (229 mg) + **10A** (181 mg)	62 mg (23.0)	8 mL/3 g	0.46 mM	16 h	Gel permeation chromatography	**11**, 6 mg, 25%[c]

[a] TLC exhibited an elongated spot. [b]When the glycan length is extended, it was found necessary to increase the equivalent of PPh_3AuNTf_2.
[c] Due to the difficulty of purification using GPC, only 24 mg of the reaction mixture was purified, afford 6 mg 256-mer **11**.

Figure S2. Preparation of the [→4)-*β*-D-Man*p*-(1→4)-*β*-D-Xyl*p*-(1→]$_n$OMP glycans via the gold (I)-catalyzed [2^n+2^n] glycosylation. EA, ethyl acetate; PE, petroleum ether; DCM, dichloromethane; MS, molecular sieves.

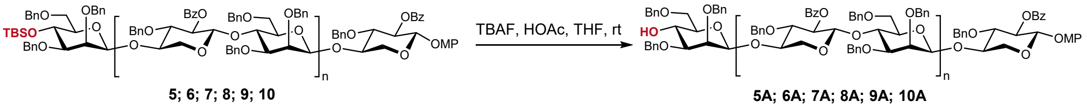

n	Substrates	**TBAF** (eq.)[a]; **HOAc** (eq.); THF	*c*	time	Method for purification	Product
1	**5** (6.14 g)	35 mL (10.0); 0.4 mL (2.0); 35 mL	100 mM	2.5 h	EA/PE = 1:1.5, R_f = 0.25	**5A**, 5.4 g, 95%
3	**6** (4.33 g)	13.2 mL (10.0); 151 μL (2.0); 30 mL	44 mM	8 h	EA/DCM/PE = 1:1:1.5, R_f = 0.25	**6A**, 3.8 g, 91%
7	**7** (2.90 g)	4.60 mL (10.0); 52.6 μL (2.0); 30 mL	15 mM	3 h	EA/DCM/PE = 4:2:5, R_f = 0.10 - 0.25 [a]	**7A**, 2.5 g, 88%
15	**8** (1.90 g)	1.53 mL (10.0); 17.6 μL (2.0); 30 mL	5.3 mM	4.5 h	Tol:Acetone = 4:1, R_f = 0.8 - 0.6 [a]	**8A**, 1.7 g, 90%[b]
31	**9** (500 mg)	0.133 mL (10.0); 1.5 μL (2.0); 5 mL	3.0 mM	24 h	Gel permeation chromatography	**9A**, 457 mg, 91%[b]
63	**10** (234 mg)	0.192 mL (40.0); 2.2 μL (8.0); 6 mL	0.82 mM	48 h	Gel permeation chromatography	**10A**, 168 mg, 72%[b]

[a] 1.0 M solution in THF. [b] Because of the poor solubility, the monitoring and purification of the 32-mer, 64-mer, and 128-mer on silica gel turned out to be problematic, leading to the product containing a small amount of impurities as indicated by NMR analysis.

Figure S3. Removal of the TBS group at the non-reducing end for the preparation of the [→4)-*β*-D-Man*p*-(1→4)-*β*-D-Xyl*p*-(1→]$_n$OMP acceptors. TBAF, tetrabutylammonium fluoride; THF, tetrahydrofuran; EA, ethyl acetate; PE, petroleum ether; DCM, dichloromethane; Tol, toluene.

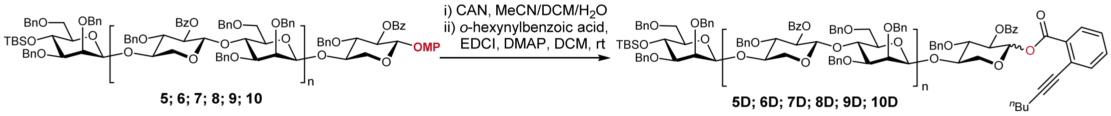

step i

n	Substrates	CAN (eq.); MeCN/DCM/H_2O	*c*	Time	Method for purification	Product
1	**5** (8.86 g)	8.30 g (3.0); 100/50/10 mL	34 mM	5 min	EA/PE = 1:2, R_f = 0.25	used directly
3	**6** (5.97 g)	4.00 g (4.0); 100/50/10 mL	12 mM	5 min	EA/DCM/PE = 1:1:1.5, R_f = 0.35-0.25	used directly
7	**7** (3.76 g)	1.31 g (4.0); 50/25/5 mL	8 mM	20 min	EA/DCM/PE = 3.5:2:5, R_f = 0.1-0.3 [a]	used directly
15	**8** (2.46 g)	872 mg (8.0); 50/25/5 mL	2.6 mM	20 min	Tol:Acetone = 4:1, R_f = 0.35-0.45 [a]	used directly
31	**9** (600 mg)	416 mg (31.0); 33/16.5/1.3 mL	0.5 mM	60 min	Monitored by ^{1}H NMR [a]	used directly
63	**10** (330 mg)	416 mg (112); 33/16.5/1.3 mL	0.13 mM	60 min	Monitored by ^{1}H NMR [a]	used directly

step ii

n	ABzOH (eq.); EDCI (eq.); DAMP (eq.); DCM	*c*	Time	Method for purification	Product (over 2 steps)
1	1.53 g (1.5); 2.41 g (2.5); 1.54 g (2.5), 30 mL	183 mM	8 h	EA/PE = 1:3, R_f = 0.5	**5D**, 6.91 g, 75%
3	533 mg (1.5); 871 mg (2.5); 557 mg (2.5), 30 mL	61 mM	9 h	EA/DCM/PE = 1:1:1.5, R_f = 0.45-0.35	**6D**, 4.98 g, 82%
7	181 mg (1.5); 285 mg (2.5); 182 mg (2.5), 20 mL	30 mM	8 h	EA/DCM/PE = 3:2:5, R_f = 0.2-0.5 [a]	**7D**, 3.05 g, 80%
15	181 mg (4.5); 285 mg (7.5); 182 mg (7.5), 20 mL	9.9 mM	10 h	Tol:Acetone = 5:1, R_f = 0.5-0.6 [a,b]	**8D**, 2.14 g, 86%
31	222 mg (45); 191 mg (75); 223 mg (75), 15 mL	1.5 mM	14 h	Short silica gel plug[a,b]	**9D**, 501 mg, 84%
63	222 mg (162); 191 mg (271); 223 mg (271), 15 mL	0.45 mM	12 h	Short silica gel plug[a,b]	**10D**, 221 mg, 67%

[a] TLC exhibited an elongated spot. [b] Because of the poor solubility, the monitoring and purification of the 32-mer, 64-mer, and 128-mer on silica gel turned out to be problematic, leading to the product containing a small number of impurities as indicated by NMR analysis.

Figure S4. Removal of the anomeric 4-methoxyphenyl (MP) group and subsequent condensation with *ortho*-hexynylbenzoic acid for the preparation of the [→4)-*β*-D-Man*p*-(1→4)-*β*-D-Xyl*p*-(1→]$_n$OMP donors. **Note**: the large excess of CAN is necessary for the fast and complete oxidation of MP group. CAN, ceric ammonium nitrate; EDCI, 1-(3-dimethylaminopropyl)-3-ethylcarbodiimide hydrochloride; DMAP, 4-dimethylaminopyridine.

General procedure for the Au(I)-catalyzed glycosylation (general procedure I)

A mixture of glycosyl donor (1.2 eq.) and acceptor (1.0 eq.) in a Schlenk flask equipped with a Teflon-coated magnetic stir bar was dried in a high vacuum at 40 °C for 8 hours, and then 5Å molecular sieves and anhydrous CH_2Cl_2 were added. The mixture was stirred at room temperature for 15 minutes and was then cooled to 0 °C, to which $Ph_3PAuNTf_2$ (0.1 or 0.2 eq.) was added. The resulting mixture was stirred at 0 °C and monitored by TLC for the disappearance of the acceptor, at which time Et_3N (2% (v/v) in CH_2Cl_2) was added to the reaction mixture. The resulting mixture was filtrated through a pad of Celite. The filtrate was concentrated *in vacuo* to give a residue, which was purified by silica gel column chromatography or by recycle gel permeation chromatography to give the product.

General procedure for the preparation of glycosyl acceptors (general procedure II)

To a solution of the fully protected glycan substrate in anhydrous tetrahydrofuran (THF) were added acetic acid and tetrabutylammonium fluoride (1 M in THF) successively. The reaction mixture was stirred at room temperature and monitored by TLC for the disappearance of the starting material, at which time the reaction mixture was poured into saturated NH_4Cl solution. The aqueous layer was extracted with EtOAc two times. The combined organic layer was dried over anhydrous $NaSO_4$, filtered, and concentrated *in vacuo*. The resulting residue was purified by silica gel column chromatography.

General procedure for the preparation of glycosyl *o*-hexynylbenzoate donors (general procedure III)

To a solution of the fully protected glycan substrate in a mixed solvent of $CH_3CN/CH_2Cl_2/H_2O$ was added ceric ammonium nitrate (CAN) at 0 °C. The reaction mixture was stirred at 0 °C and monitored by TLC for the disappearance of starting material, at which time the reaction mixture was poured into saturated $NaHCO_3$ solution. The aqueous layer was extracted with EtOAc two times, and the combined organic layer was dried over anhydrous Na_2SO_4 and concentrated *in vacuo*. The residue was purified by silica gel chromatography (0.5% Et_3N as additive) to afford a brown foam. The brown foam was used for the next step directly.

To a mixture of the above foam, *ortho*-hexynylbenzoic acid, and 4-dimethylaminopyridine in CH_2Cl_2 was added 1-(3-dimethylaminopropyl)-3-ethylcarbodiimide hydrochloride. The reaction mixture was stirred at room temperature and monitored by TLC for the disappearance of starting material, at which time the reaction mixture was poured into saturated $NaHCO_3$ solution. The aqueous layer was extracted with CH_2Cl_2 two times, and the combined organic layer was dried over anhydrous Na_2SO_4 and concentrated *in vacuo*. The resulting residue was purified by silica gel chromatography to afford the corresponding donor.

Fully protected 4-mer 5

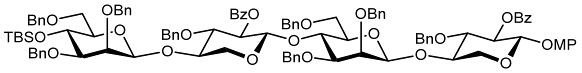

5

A white foam: $[\alpha]_D^{25}$ = -26.8 (*c* 1.0, $CHCl_3$); ^1H NMR (500 MHz, $CDCl_3$) δ 8.06–7.99 (m, 2 H), 7.97–7.92 (m, 2 H), 7.54 (ddt, J = 8.7, 7.3, 1.3 Hz, 1 H), 7.49 (ddt, J = 8.7, 7.3, 1.3 Hz, 1 H), 7.41–7.12 (m, 37 H), 7.09–6.89 (m, 13 H), 6.79–6.73 (m, 2 H), 5.39 (dd, J = 7.1, 5.5 Hz, 1 H), 5.19 (dd, J = 8.7, 7.1 Hz, 1 H), 5.14 (d, J = 5.5 Hz, 1 H), 4.91–4.78 (m, 4 H), 4.75–4.51 (m, 10 H), 4.49–4.39 (m, 5 H), 4.23 (t, J = 9.4 Hz, 1 H), 4.15–4.08 (m, 2 H), 4.04 (ddd, J = 8.8, 7.7, 5.0 Hz, 1 H), 4.01–3.95 (m, 2 H), 3.91 (dd, J = 12.2, 4.9 Hz, 1 H), 3.87 (t, J = 6.8 Hz, 1 H), 3.81 (dd, J = 7.4, 3.0 Hz, 2 H), 3.78 (dd, J = 11.0, 1.8 Hz, 1 H), 3.72 (s, 3 H), 3.64 (dd, J = 8.7, 7.7 Hz, 1 H), 3.58–3.51 (m, 2 H), 3.48–3.43 (m, 2 H), 3.40 (dd, J = 9.3, 3.1 Hz, 1 H), 3.38–3.34 (m, 1 H), 3.29 (dd, J = 9.1, 2.9 Hz, 1 H), 3.15 (ddd, J = 9.5, 4.5, 1.8 Hz, 1 H), 3.09 (dd, J = 12.2, 8.7 Hz, 1 H), 0.81 (s, 9 H), 0.01 (s, 3 H), -0.01 (s, 3 H); ^{13}C NMR (126 MHz, $CDCl_3$) δ 165.3, 164.9, 155.1, 150.7, 138.7, 138.6, 138.51, 138.45, 138.3, 138.2, 138.0, 137.9, 132.9, 132.8, 129.8, 129.7, 129.60, 129.55, 128.2, 128.10, 128.06, 128.02, 127.97, 127.93, 127.89, 127.86, 127.84, 127.80, 127.78, 127.76, 127.7, 127.61, 127.55, 127.49, 127.45, 127.44, 127.37, 127.34, 127.31, 127.29, 127.26, 127.2, 127.13, 127.10, 127.02, 127.01, 126.8, 118.4, 114.3, 100.7, 99.9, 99.6, 99.4, 82.2, 80.0, 79.0, 77.6, 75.7, 75.6, 75.1, 74.6, 74.1, 73.9, 73.8, 73.3, 73.1, 73.0, 72.6, 72.5, 72.2, 70.9, 70.7, 69.5, 68.2, 67.7, 62.3, 61.4, 55.4, 25.8, 17.9, -4.0, -5.1; HRMS (ESI) calcd for $C_{105}H_{114}O_{22}SiNa$ $[M + Na]^+$ 1777.7469, found 1777.7475.

4-Mer acceptor 5A

5A

A white foam: $[\alpha]_D^{25}$ = -30.7 (*c* 1.0, $CHCl_3$); ^{1}H NMR (500 MHz, $CDCl_3$) δ 8.05–7.97 (m, 2 H), 7.97–7.93 (m, 2 H), 7.54 (tt, *J* = 7.4, 1.3 Hz, 1 H), 7.53–7.47 (m, 1 H), 7.41–7.13 (m, 35 H), 7.09–6.96 (m, 10 H), 6.94–6.90 (m, 2 H), 6.81–6.73 (m, 2 H), 5.39 (dd, *J* = 7.1, 5.5 Hz, 1 H), 5.17 (dd, *J* = 8.6, 6.9 Hz, 1 H), 5.14 (d, *J* = 5.5 Hz, 1 H), 4.89 (d, *J* = 12.0 Hz, 1 H), 4.86–4.79 (m, 3 H), 4.77–4.71 (m, 3 H), 4.69 (d, *J* = 12.4 Hz, 2 H), 4.64–4.54 (m, 3 H), 4.50–4.39 (m, 6 H), 4.24 (t, *J* = 9.4 Hz, 1 H), 4.14 (d, *J* = 11.9 Hz, 1 H), 4.10 (dd, *J* = 12.2, 4.0 Hz, 1 H), 4.03–3.96 (m, 3 H), 3.91 (dd, *J* = 12.2, 4.9 Hz, 1 H), 3.86 (t, *J* = 6.8 Hz, 1 H), 3.83 (d, *J* = 3.0 Hz, 1 H), 3.81 (d, *J* = 3.1 Hz, 1 H), 3.75–3.71 (m, 4 H), 3.68–3.63 (m, 2 H), 3.55 (dd, *J* = 11.3, 4.5 Hz, 1 H), 3.49–3.43 (m, 2 H), 3.41 (dd, *J* = 9.3, 3.1 Hz, 1 H), 3.37 (ddd, *J* = 9.6, 5.5, 4.2 Hz, 1 H), 3.32 (dd, *J* = 9.4, 3.0 Hz, 1 H), 3.16 (ddd, *J* = 9.6, 4.5, 1.8 Hz, 1 H), 3.10 (dd, *J* = 12.2, 8.5 Hz, 1 H), 2.71 (d, *J* = 1.9 Hz, 1 H); ^{13}C NMR (126 MHz, $CDCl_3$) δ 165.6, 165.3, 155.4, 151.1, 138.9, 138.8, 138.7, 138.5, 138.4, 138.1, 138.0, 133.3, 133.2, 130.1, 130.0, 129.9, 128.6, 128.5, 128.40, 128.35, 128.3, 128.14, 128.12, 128.07, 128.03, 127.97, 127.94, 127.90, 127.86, 127.80, 127.79, 127.7, 127.64, 127.57, 127.55, 127.4, 127.34, 127.27, 118.7, 114.6, 100.9, 100.2, 100.1, 100.0, 81.5, 80.3, 79.2, 77.4, 77.2, 77.0, 76.3, 75.9, 75.42, 75.40, 75.3, 74.54, 74.49, 74.23, 74.15, 73.9, 73.4, 72.9, 72.8, 72.5, 71.6, 71.0, 70.9, 68.6, 68.5, 62.6, 61.7, 55.7; HRMS (ESI) calcd for $C_{99}H_{100}O_{22}Na$ $[M + Na]^+$ 1663.6604, found 1663.6625.

4-Mer donor 5D

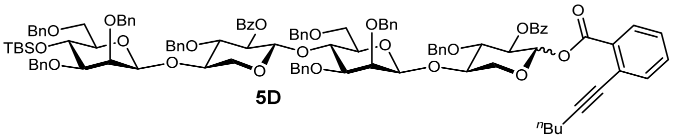

A white foam (α/β= 0.11:1): ^{1}H NMR (500 MHz, $CDCl_3$) δ 8.03–7.99 (m, 2 H), 7.97–7.92 (m, 2 H), 7.91 (dd, *J* = 8.4, 1.4 Hz, 0.19 H), 7.89–7.82 (m, 1 H), 7.52 (tt, *J* = 7.3, 1.3 Hz, 1 H), 7.49–7.43 (m, 2 H), 7.41–6.90 (m, 48 H), 6.54 (d, *J* = 3.7 Hz, 0.08 H), 6.20 (d, *J* = 4.4 Hz, 0.90 H), 5.38–5.34 (m, 0.93 H), 5.32 (dd, *J* = 9.2, 3.8 Hz, 0.10 H), 5.18 (dd, *J* = 8.7, 7.0 Hz, 1 H), 4.96 (d, *J* = 11.8 Hz, 0.11 H), 4.92–4.81 (m, 2 H), 4.78–4.72 (m, 2 H), 4.71–4.61 (m, 6 H), 4.55 (dd, *J* = 24.6, 12.4 Hz, 3 H), 4.48–4.43 (m, 4 H), 4.36 (d, *J* = 11.8 Hz, 1 H), 4.33–4.27 (m, 0.16 H), 4.23–4.08 (m, 3 H), 4.07–3.87 (m, 5 H), 3.84–3.73 (m, 4 H), 3.69–3.60 (m, 2 H), 3.59–3.52 (m, 1 H), 3.47 (dd, *J* = 11.1, 4.8 Hz, 1 H), 3.45–3.39 (m, 2 H), 3.38–3.32 (m, 1 H), 3.28 (dd, *J* = 9.1, 2.8 Hz, 1 H), 3.21–3.15 (m, 1 H), 3.07 (dd, *J* = 12.2, 8.6 Hz, 1 H), 2.44 (t, *J* = 7.2 Hz, 1.84 H), 2.34 (td, *J* = 7.3, 2.7 Hz, 0.25 H), 1.62–1.54 (m, 1.91 H), 1.51–1.33 (m, 2.27 H), 0.95–0.82 (m, 3 H), 0.80 (s, 8 H), 0.80 (d, *J* = 4.0 Hz, 1 H), 0.00 (s, 3 H), -0.02 (s, 3 H); ^{13}C NMR (126 MHz, $CDCl_3$) δ 165.59, 165.56, 165.2, 164.2, 163.9, 139.0, 138.9, 138.8, 138.7, 138.61, 138.55, 138.5, 138.2, 138.0, 134.9, 134.6, 133.22, 133.18, 132.2, 130.9, 130.4, 130.17, 130.15, 130.0, 129.92, 129.90, 129.6, 129.5, 128.4, 128.32, 128.30, 128.19, 128.16, 128.1, 128.00, 127.97, 127.92, 127.86, 127.83, 127.79, 127.7, 127.62, 127.59, 127.56, 127.51, 127.47, 127.44, 127.42, 127.33, 127.31, 127.2, 127.1, 125.7, 125.4, 101.0, 100.9, 100.3, 99.7, 99.6, 97.2, 97.1, 92.5, 90.7, 82.5, 82.4, 80.4, 80.3, 79.3, 79.24, 79.19, 79.1, 77.9, 77.4, 77.2, 77.0, 76.2, 76.02, 75.95, 75.84, 75.79, 75.4, 75.1, 74.9, 74.40, 74.36, 74.3, 74.2, 74.0, 73.6, 73.5, 73.33, 73.28, 73.2, 73.0, 72.81, 72.78, 72.4, 71.2, 69.8, 69.0, 68.6, 68.3, 68.0, 62.6, 61.9, 61.5, 30.8, 30.7, 26.1, 22.2, 22.1, 19.7, 19.6, 18.2, 13.79, 13.75, 0.1, -3.7, -4.8; HRMS (ESI) calcd for $C_{111}H_{120}O_{22}Na$ $[M + Na]^+$ 1855.7938, found 1855.7946.

Fully protected 8-mer 6

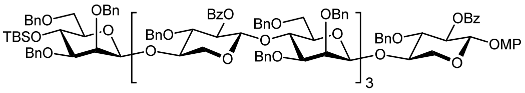

6

A white foam: $[\alpha]_D^{25}$ = -27.0 (*c* 1.0, $CHCl_3$); ^{1}H NMR (500 MHz, $CDCl_3$) δ 8.04–8.00 (m, 2 H), 7.96–7.93 (m, 2 H), 7.93–7.90 (m, 4 H), 7.55–7.47 (m, 4 H), 7.40–7.10 (m, 70 H), 7.08–6.90 (m, 18 H), 6.88–6.84 (m, 4 H), 6.79–6.74 (m, 2 H), 5.38 (dd, *J* = 7.1, 5.5 Hz, 1 H), 5.20 (dd, *J* = 8.8, 7.1 Hz, 1 H), 5.15–5.07 (m, 3 H), 4.89–4.76 (m, 10 H), 4.74–4.70 (m, 3 H), 4.70–4.62 (m, 8 H), 4.60 (d, *J* = 6.7 Hz, 1 H), 4.58–4.50 (m, 6 H), 4.49–4.44 (m, 5 H), 4.41–4.38 (m, 2 H), 4.31–4.08 (m, 9 H), 4.05 (td, *J* = 8.1, 4.9 Hz, 1 H), 4.01–3.94 (m, 2 H), 3.94–3.83 (m, 4 H), 3.82–3.77 (m, 5 H), 3.74 (t, *J* = 3.6 Hz, 2 H), 3.72 (s, 3 H), 3.66–3.33 (m, 16 H), 3.29 (dd, *J* = 9.1, 2.8 Hz, 1 H), 3.17–3.07 (m, 4 H), 3.02–2.94 (m, 2 H), 0.81 (s, 9 H), 0.00 (s, 3 H), -0.01 (s, 3 H); ^{13}C NMR (126 MHz, $CDCl_3$) δ 165.6, 165.28, 165.26, 155.4, 151.1, 139.02, 138.95, 138.91, 138.87, 138.85, 138.78, 138.75, 138.64, 138.56, 138.53, 138.50, 138.47, 138.3, 138.2, 133.23, 133.20, 133.16, 130.1, 130.0, 129.93, 129.91, 129.85, 128.5, 128.37, 128.35, 128.33, 128.31, 128.29, 128.20, 128.16, 128.12, 128.10, 128.07, 128.03, 127.99, 127.95, 127.91, 127.87, 127.83, 127.77, 127.71, 127.66, 127.64, 127.61, 127.58, 127.56, 127.53, 127.47, 127.43, 127.40, 127.34, 127.32, 127.30, 127.12, 127.09, 118.7, 114.6, 101.0, 100.9, 100.2, 100.0, 99.73, 99.71, 82.5, 80.3, 80.24, 80.21, 79.31, 79.26, 77.9, 77.4, 77.2, 77.0, 76.22, 76.20, 76.1, 76.04, 75.99, 75.8, 75.73, 75.67, 75.39, 75.35, 74.9, 74.4, 74.3, 74.2, 74.14, 74.05, 73.6, 73.49, 73.46, 73.39, 73.37, 73.34, 73.30, 72.91, 72.87, 72.6, 72.50, 72.45, 71.2, 71.0, 69.8, 68.5, 68.4, 68.0, 62.6, 62.5, 61.7, 55.7, 26.1, 18.3, 0.1, -3.7, -4.8; MALDI FT-ICR MS calcd for $C_{197}H_{206}O_{42}SiK$ $[M + K]^+$ 3310.338, found 3310.315.

8-Mer acceptor 6A

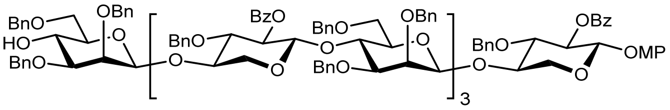

6A

A white foam: $[\alpha]_D^{25}$ = -18.9 (*c* 1.0, $CHCl_3$); ^{1}H NMR (600 MHz, $CDCl_3$) δ 8.04–8.00 (m, 2 H), 7.98–7.94 (m, 2 H), 7.93–7.89 (m, 4 H), 7.58–7.47 (m, 4 H), 7.40–7.32 (m, 23 H), 7.31–7.28 (m, 6 H), 7.28–7.11 (m, 39 H), 7.09–7.03 (m, 5 H), 7.02–6.95 (m, 7 H), 6.94–6.90 (m, 6 H), 6.89–6.84 (m, 4 H), 6.79–6.74 (m, 2 H), 5.38 (dd, *J* = 7.1, 5.5 Hz, 1 H), 5.18 (dd, *J* = 8.6, 6.9 Hz, 1 H), 5.15–5.07 (m, 3 H), 4.92–4.44 (m, 32 H), 4.42–4.37 (m, 2 H), 4.31–4.07 (m, 9 H), 4.03–3.71 (m, 17 H), 3.68–3.34 (m, 15 H), 3.32 (dd, *J* = 9.4, 2.9 Hz, 1 H), 3.16–3.08 (m, 4 H), 3.04–2.94 (m, 2 H), 2.72 (d, *J* = 1.9 Hz, 1 H); ^{13}C NMR (151 MHz, $CDCl_3$) δ 165.5, 165.18, 165.16, 165.1, 155.25, 150.93, 138.83, 138.80, 138.73, 138.66, 138.6, 138.44, 138.41, 138.37, 138.35, 138.2, 138.0, 137.89, 133.12, 133.09, 133.04, 129.96, 129.84, 129.81, 129.74, 129.72, 128.5, 128.4, 128.34, 128.25, 128.22, 128.20, 128.17, 128.1, 128.00, 127.97, 127.95, 127.93, 127.90, 127.83, 127.79, 127.76, 127.73, 127.70, 127.67, 127.64, 127.59, 127.51, 127.46, 127.44, 127.41, 127.36, 127.32, 127.30, 127.28, 127.22, 127.20, 127.1, 127.0, 118.6, 114.4, 100.81, 100.75, 100.1, 99.9, 99.8, 99.62, 99.59, 81.4, 80.2, 80.1, 79.21, 79.15, 79.1, 77.2, 77.0, 76.8, 76.12, 76.08, 75.93, 75.87, 75.7, 75.58, 75.56, 75.3, 75.2, 75.1, 74.39, 74.36, 74.2, 74.1, 74.0, 73.93, 73.90, 73.8, 73.38, 73.35, 73.32, 73.26, 73.2, 72.80, 72.78, 72.74, 72.67, 72.41, 72.38, 72.3, 71.4, 70.9, 70.7, 68.4, 68.34, 68.28, 68.2, 62.40, 62.37, 61.6, 55.6; MALDI FT-ICR MS calcd for $C_{191}H_{192}O_{42}K$ $[M + K]^+$ 3196.252, found 3196.227.

8-Mer donor 6D

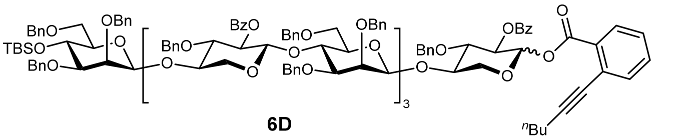

6D

A white foam (α/β= 0.52:1): ^{1}H NMR (600 MHz, CDCl$_3$) δ 8.03–7.99 (m, 1 H), 7.97–7.89 (m, 7 H), 7.87–7.81 (m, 1 H), 7.58–7.42 (m, 6 H), 7.41–6.89 (m, 93 H), 6.88–6.84 (m, 4 H), 6.54 (d, J = 3.7 Hz, 0.34 H), 6.20 (d, J = 4.4 Hz, 0.66 H), 5.36 (t, J = 5.0 Hz, 0.71 H), 5.31 (dd, J = 9.2, 3.8 Hz, 0.37 H), 5.20 (dd, J = 8.7, 7.0 Hz, 1 H), 5.12 (tdd, J = 10.7, 7.9, 4.3 Hz, 2 H), 4.95 (d, J = 11.8 Hz, 0.43 H), 4.91–4.40 (m, 37 H), 4.34 (d, J = 11.8 Hz, 0.74 H), 4.30–4.21 (m, 4.73 H), 4.20–4.02 (m, 7 H), 4.01–3.96 (m, 2 H), 3.95–3.86 (m, 4 H), 3.84–3.72 (m, 9 H), 3.68–3.33 (m, 16 H), 3.29 (dd, J = 9.1, 2.8 Hz, 1 H), 3.18–3.07 (m, 4.67 H), 3.02–2.92 (m, 2 H), 2.45 (t, J = 7.2 Hz, 1.40 H), 2.34 (td, J = 7.1, 2.7 Hz, 0.74 H), 1.63–1.36 (m, 4 H), 0.92 (t, J = 7.4 Hz, 2 H), 0.86 (t, J = 7.3 Hz, 1 H), 0.81 (s, 9 H), 0.01 (s, 3 H), -0.01 (s, 3 H); ^{13}C NMR (151 MHz, CDCl$_3$) δ 165.46, 165.44, 165.2, 165.11, 164.1, 163.7, 138.9, 138.83, 138.79, 138.76, 138.74, 138.68, 138.64, 138.59, 138.52, 138.45, 138.42, 138.37, 138.3, 138.1, 137.9, 134.7, 134.5, 133.10, 133.07, 132.1, 130.7, 130.6, 130.3, 130.04, 130.02, 129.84, 129.79, 129.74, 129.72, 129.5, 129.4, 128.32, 128.27, 128.22, 128.20, 128.18, 128.16, 128.1, 128.03, 127.96, 127.87, 127.85, 127.82, 127.76, 127.73, 127.71, 127.68, 127.6, 127.52, 127.50, 127.47, 127.44, 127.42, 127.38, 127.33, 127.29, 127.20, 127.18, 127.16, 127.11, 127.09, 127.02, 126.98, 126.96, 125.5, 125.2, 100.82, 100.81, 100.78, 100.75, 100.1, 99.59, 99.57, 99.5, 97.04, 96.95, 92.4, 90.6, 82.41, 80.35, 80.2, 80.14, 80.09, 79.2, 79.13, 79.07, 79.0, 77.8, 77.3, 77.0, 76.8, 76.1, 76.03, 76.00, 75.92, 75.88, 75.8, 75.7, 75.62, 75.59, 75.53, 75.51, 75.3, 74.9, 74.8, 74.2, 74.14, 74.09, 74.06, 73.92, 73.89, 73.5, 73.4, 73.3, 73.22, 73.19, 73.15, 72.84, 72.76, 72.74, 72.70, 72.6, 72.5, 72.4, 72.1, 71.12, 71.10, 69.7, 68.9, 68.5, 68.3, 68.2, 67.9, 62.5, 62.4, 62.3, 61.8, 61.4, 30.64, 30.56, 25.9, 22.1, 22.0, 19.54, 19.52, 18.1, 13.7, 13.6, 0.0, -3.8, -4.9; MALDI FT-ICR MS calcd for $C_{203}H_{212}O_{42}SiK$ [M + K]$^+$ 3388.385, found 3388.357.

Fully protected 16-mer 7

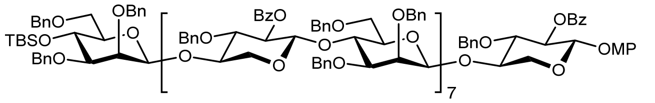

7

A white foam: $[\alpha]_D^{25}$ = -13.3 (c 1.0, CHCl$_3$); ^{1}H NMR (600 MHz, CDCl$_3$) δ 8.05–8.00 (m, 2 H), 7.97–7.93 (m, 2 H), 7.92–7.90 (m, 13 H), 7.56–7.47 (m, 8 H), 7.40–7.22 (m, 77 H), 7.21–7.10 (m, 72 H), 7.08–6.89 (m, 32 H), 6.88–6.83 (m, 12 H), 6.80–6.72 (m, 2 H), 5.37 (dd, J = 7.1, 5.5 Hz, 1 H), 5.19 (dd, J = 8.7, 7.1 Hz, 1 H), 5.15–5.07 (m, 7 H), 4.89–4.74 (m, 23 H), 4.74–4.70 (m, 3 H), 4.69–4.61 (m, 17 H), 4.61–4.50 (m, 16 H), 4.50–4.43 (m, 9 H), 4.41–4.37 (m, 2 H), 4.29–4.07 (m, 22 H), 4.04 (td, J = 8.1, 4.9 Hz, 1 H), 4.00–3.94 (m, 2 H), 3.93–3.83 (m, 8 H), 3.79 (ddd, J = 13.2, 7.3, 2.7 Hz, 10 H), 3.73 (d, J = 3.6 Hz, 9 H), 3.66–3.33 (m, 33 H), 3.28 (dd, J = 9.1, 2.8 Hz, 1 H), 3.16–3.07 (m, 9 H), 3.01–2.93 (m, 6 H), 0.80 (s, 9 H), -0.00 (s, 3 H), -0.02 (s, 3 H); ^{13}C NMR (151 MHz, CDCl$_3$) δ 165.5, 165.2, 165.1, 155.3, 150.9, 138.9, 138.84, 138.79, 138.75, 138.72, 138.66, 138.6, 138.5, 138.44, 138.41, 138.37, 138.3, 138.2, 138.1, 133.1, 133.04, 129.96, 129.9, 129.8, 129.7, 128.33, 128.25, 128.21, 128.19, 128.1, 128.04, 127.99, 127.97, 127.95, 127.9, 127.83, 127.79,

127.76, 127.7, 127.64, 127.58, 127.54, 127.52, 127.49, 127.46, 127.4, 127.32, 127.29, 127.22, 127.19, 127.00, 126.96, 118.6, 114.4, 100.83, 100.79, 100.1, 99.8, 99.61, 99.58, 82.4, 80.2, 80.11, 80.08, 79.2, 79.1, 77.8, 77.2, 77.0, 76.8, 76.1, 75.94, 75.92, 75.87, 75.7, 75.61, 75.55, 75.5, 75.3, 75.2, 74.8, 74.3, 74.2, 74.1, 74.0, 73.9, 73.5, 73.4, 73.34, 73.27, 73.24, 73.22, 73.18, 72.79, 72.76, 72.5, 72.4, 72.3, 71.1, 70.9, 69.7, 68.3, 68.2, 67.9, 62.5, 62.4, 61.6, 55.6, 25.9, 18.1, 0.0, -3.8, -4.9; MALDI FT-ICR MS calcd for $C_{381}H_{390}O_{82}SiK$ [M + K]$^+$ 6343.575, found 6343.582.

16-Mer acceptor 7A

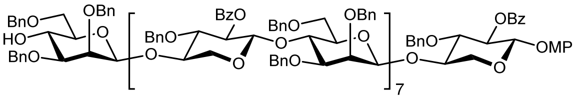

7A

A white foam: $[\alpha]_D^{25}$ = -13.0 (*c* 1.0, $CHCl_3$); ^{1}H NMR (600 MHz, $CDCl_3$) δ 8.03–8.00 (m, 2H), 7.96–7.94 (m, 2H), 7.93–7.89 (m, 11H), 7.56–7.48 (m, 8H), 7.40–7.22 (m, 76H), 7.21–7.10 (m, 62H), 7.08–7.03 (m, 5H), 7.03–6.94 (m, 10H), 6.94–6.89 (m, 13H), 6.88–6.83 (m, 11H), 6.78–6.74 (m, 2H), 5.37 (dd, *J* = 7.1, 5.5 Hz, 1H), 5.18 (dd, *J* = 8.6, 6.9 Hz, 1H), 5.15–5.08 (m, 7H), 4.91–4.70 (m, 25H), 4.69–4.61 (m, 14H), 4.60–4.51 (m, 14H), 4.50–4.43 (m, 9H), 4.41–4.37 (m, 2H), 4.31–4.06 (m, 22H), 4.03–3.70 (m, 30H), 3.68–3.34 (m, 32H), 3.32 (dd, *J* = 9.4, 2.9 Hz, 1H), 3.10 (d, *J* = 9.4 Hz, 8H), 2.97 (t, *J* = 9.8 Hz, 6H), 2.71 (d, *J* = 1.8 Hz, 1H); ^{13}C NMR (151 MHz, $CDCl_3$) δ 165.5, 165.18, 165.16, 165.1, 155.3, 150.9, 138.84, 138.79, 138.75, 138.73, 138.66, 138.58, 138.5, 138.42, 138.37, 138.35, 138.2, 138.0, 137.9, 133.09, 133.05, 130.0, 129.84, 129.81, 129.75, 129.7, 128.5, 128.4, 128.3, 128.2, 128.04, 128.00, 127.98, 127.95, 127.93, 127.91, 127.83, 127.79, 127.76, 127.73, 127.71, 127.67, 127.65, 127.6, 127.51, 127.46, 127.42, 127.35, 127.32, 127.29, 127.2, 127.1, 127.0, 118.6, 114.5, 100.80, 100.75, 100.1, 99.9, 99.8, 99.62, 99.59, 81.4, 80.2, 80.1, 79.2, 79.1, 77.2, 77.0, 76.8, 76.13, 76.09, 75.93, 75.87, 75.7, 75.6, 75.5, 75.3, 75.2, 75.1, 74.39, 74.36, 74.2, 74.1, 74.0, 73.93, 73.91, 73.8, 73.39, 73.35, 73.33, 73.27, 73.24, 73.22, 72.80, 72.77, 72.7, 72.41, 72.37, 72.3, 71.4, 70.9, 70.8, 68.4, 68.34, 68.25, 62.4, 61.6, 55.6; MALDI FT-ICR MS calcd for $C_{375}H_{376}O_{82}K$ [M + K]$^+$ 6229.488, found 6229.519.

16-Mer donor 7D

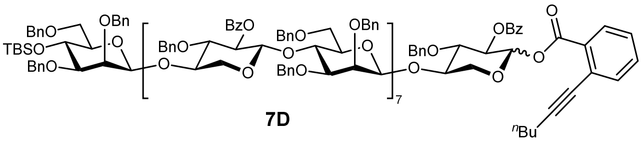

7D

A white foam (α/β= 0.39:1): ^{1}H NMR (600 MHz, $CDCl_3$) δ 8.02–7.99 (m, 1.52H), 7.96–7.89 (m, 15H), 7.86–7.82 (m, 1H), 7.55–7.44 (m, 9.32H), 7.40–7.22 (m, 76.55H), 7.22–7.10 (m, 71H), 7.09–6.90 (m, 29.70H), 6.88–6.83 (m, 12H), 6.53 (d, *J* = 3.7 Hz, 0.28H), 6.19 (d, *J* = 4.4 Hz, 0.72H), 5.35 (t, *J* = 5.0 Hz, 0.80H), 5.31 (dd, *J* = 9.1, 3.8 Hz, 0.28H), 5.19 (dd, *J* = 8.7, 7.0 Hz, 1H), 5.14–5.07 (m, 6.39H), 4.94 (d, *J* = 11.8 Hz, 0.31H), 4.89–4.41 (m, 68.63H), 4.34 (d, *J* = 11.8 Hz, 0.55H), 4.29–4.20 (m, 13H), 4.20–4.02 (m, 11H), 4.00–3.85 (m, 10H), 3.83–3.70 (m, 17H), 3.68–3.33 (m, 32H), 3.28 (dd, *J* = 9.1, 2.8 Hz, 1H), 3.19–3.06 (m, 8H), 3.02–2.90 (m, 6H), 2.45 (t, *J* = 7.2 Hz, 1.50H), 2.34 (td, *J* = 7.1, 2.6 Hz, 0.65H), 1.62–1.36 (m, 4H), 0.92 (t, *J* = 7.3 Hz, 2H), 0.86 (t, *J* = 7.3 Hz, 1H), 0.80 (s, 9H), -0.02 (s, 3H); ^{13}C NMR (151 MHz, $CDCl_3$) δ 165.47, 165.45, 165.2, 165.1, 163.8, 138.9, 138.84, 138.79, 138.76, 138.73, 138.68, 138.65, 138.64, 138.59, 138.52, 138.45, 138.42,

138.37, 138.3, 138.1, 137.9, 134.8, 134.5, 133.12, 133.08, 132.1, 130.8, 130.3, 130.0, 129.9, 129.8, 129.7, 129.5, 128.3, 128.23, 128.21, 128.19, 128.1, 128.03, 127.98, 127.88, 127.86, 127.83, 127.78, 127.75, 127.72, 127.70, 127.6, 127.54, 127.52, 127.49, 127.46, 127.43, 127.39, 127.34, 127.29, 127.22, 127.19, 127.1, 127.03, 126.99, 126.96, 125.6, 125.3, 100.83, 100.79, 100.1, 99.6, 99.5, 97.1, 92.4, 90.6, 82.4, 80.3, 80.2, 80.12, 80.09, 79.19, 79.15, 79.09, 79.0, 77.8, 77.2, 77.0, 76.8, 76.1, 75.93, 75.88, 75.6, 75.5, 75.3, 74.9, 74.8, 74.3, 74.2, 74.1, 73.9, 73.5, 73.4, 73.3, 73.23, 73.20, 73.17, 72.9, 72.8, 72.6, 72.5, 72.4, 72.2, 71.1, 69.7, 68.9, 68.5, 68.3, 67.9, 62.5, 62.4, 61.4, 30.7, 30.6, 25.9, 22.1, 22.0, 19.6, 19.5, 18.1, 13.7, 13.6, -3.8, -4.9; MALDI FT-ICR MS calcd for $C_{387}H_{396}O_{82}SiK$ $[M + K]^+$ 6421.622, found 6421.635.

Fully protected 32-mer 8

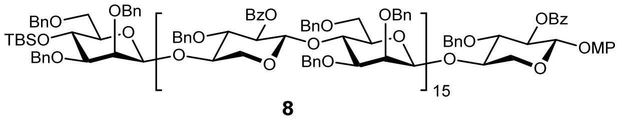

8

A white solid: $[\alpha]_D^{25}$ = -26.4 (*c* 1.0, $CHCl_3$); ^1H NMR (600 MHz, $CDCl_3$) δ 8.04–7.99 (m, 2H), 7.96–7.92 (m, 3H), 7.93–7.88 (m, 30H), 7.57–7.48 (m, 20H), 7.41–7.23 (m, 162H), 7.21–7.10 (m, 154H), 7.08–6.93 (m, 29H), 6.93–6.89 (m, 33H), 6.85 (t, *J* = 7.4 Hz, 30H), 6.79–6.73 (m, 2H), 5.37 (dd, *J* = 7.1, 5.6 Hz, 1H), 5.19 (dd, *J* = 8.7, 7.1 Hz, 1H), 5.11 (p, *J* = 5.7 Hz, 16H), 4.88–4.36 (m, 149H), 4.31–3.94 (m, 58H), 3.88 (q, *J* = 7.7 Hz, 20H), 3.82–3.68 (m, 39H), 3.66–3.32 (m, 74H), 3.28 (dd, *J* = 9.0, 2.8 Hz, 1H), 3.10 (d, *J* = 10.1 Hz, 18H), 2.96 (d, *J* = 10.5 Hz, 16H), 0.80 (s, 9H), -0.02 (s, 3H); ^{13}C NMR (151 MHz, $CDCl_3$) δ 165.5, 165.2, 155.3, 150.9, 138.9, 138.83, 138.78, 138.73, 138.70, 138.64, 138.60, 138.5, 138.39, 138.36, 138.2, 138.1, 133.1, 130.0, 129.9, 129.81, 129.78, 129.7, 128.3, 128.2, 128.09, 128.05, 128.0, 127.9, 127.84, 127.81, 127.78, 127.72, 127.65, 127.59, 127.54, 127.50, 127.46, 127.4, 127.30, 127.24, 127.21, 127.0, 118.6, 116.4, 114.5, 100.84, 100.79, 100.1, 99.8, 99.6, 82.4, 80.2, 80.10, 80.07, 79.2, 77.2, 77.0, 76.8, 76.1, 76.0, 75.90, 75.85, 75.7, 75.63, 75.55, 75.28, 75.25, 74.8, 74.3, 74.2, 74.1, 74.0, 73.9, 73.5, 73.4, 73.3, 73.2, 72.81, 72.76, 72.5, 72.4, 71.1, 70.9, 69.7, 68.2, 67.9, 62.5, 62.4, 61.6, 55.6, 29.7, 26.0, 18.1, -0.0, -3.8, -4.9; MALDI FT-ICR MS calcd for $C_{749}H_{758}O_{162}SiK$ $[M + K]^+$ 12412.054, found 12412.140.

32-Mer acceptor 8A

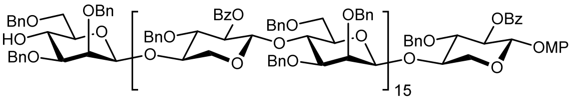

8A

A colorless amorphous solid: $[\alpha]_D^{25}$ = -9.5 (*c* 1.0, $CHCl_3$); ^1H NMR (600 MHz, $CDCl_3$) δ 8.04–8.00 (m, 2H), 7.98–7.93 (m, 2H), 7.94–7.89 (m, 29H), 7.56–7.47 (m, 15H), 7.40–7.20 (m, 140H), 7.22–7.10 (m, 141H), 7.09–7.03 (m, 5H), 7.02–6.93 (m, 19H), 6.94–6.89 (m, 31H), 6.89–6.80 (m, 29H), 6.79–6.73 (m, 2H), 5.37 (dd, *J* = 7.1, 5.6 Hz, 1H), 5.18 (dd, *J* = 8.6, 6.9 Hz, 1H), 5.15–5.07 (m, 16H), 4.91–4.74 (m, 50H), 4.73–4.61 (m, 35H), 4.60–4.51 (m, 32H), 4.50–4.44 (m, 19H), 4.42–4.37 (m, 3H), 4.32–4.06 (m, 46H), 4.02–3.83 (m, 24H), 3.82–3.76 (m, 11H), 3.75–3.70 (m, 22H), 3.66 (dd, *J* = 10.1, 5.9 Hz, 2H), 3.62–3.50 (m, 30H), 3.50–3.42 (m, 16H), 3.41–3.34 (m, 18H), 3.32 (dd, *J* = 9.5, 2.9 Hz, 1H), 3.17–3.06 (m, 17H), 2.97 (s, 15H), 2.70 (s, 1H); ^{13}C NMR (151 MHz, $CDCl_3$) δ 165.5, 165.18, 165.15, 155.3, 150.9, 138.84, 138.80, 138.77, 138.74, 138.68, 138.61,

138.59, 138.53, 138.46, 138.43, 138.38, 138.2, 138.0, 137.9, 133.1, 133.04, 129.97, 129.8, 129.7, 128.5, 128.41, 128.37, 128.34, 128.26, 128.22, 128.18, 128.03, 127.99, 127.98, 127.95, 127.93, 127.91, 127.86, 127.83, 127.78, 127.75, 127.73, 127.69, 127.67, 127.65, 127.58, 127.54, 127.51, 127.46, 127.4, 127.34, 127.29, 127.20, 127.18, 127.1, 127.0, 118.6, 114.5, 100.79, 100.75, 100.1, 99.9, 99.8, 99.6, 81.4, 80.2, 80.1, 79.2, 79.1, 77.2, 77.0, 76.8, 76.13, 76.08, 76.0, 75.9, 75.7, 75.6, 75.5, 75.3, 75.2, 75.1, 74.39, 74.36, 74.2, 74.1, 74.0, 73.9, 73.8, 73.4, 73.34, 73.32, 73.27, 73.2, 72.79, 72.77, 72.7, 72.41, 72.37, 72.3, 71.4, 70.9, 70.8, 68.5, 68.3, 62.4, 61.6, 55.6; MALDI FT-ICR MS calcd for $C_{743}H_{744}O_{162}K$ $[M + K]^+$ 12297.968, found 12298.065.

32-Mer donor 8D

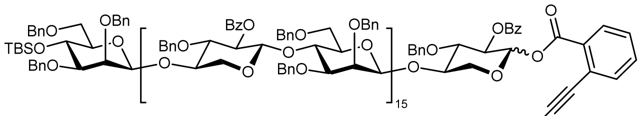

8D

A white foam (α/β= 0.54:1): ^{1}H NMR (600 MHz, $CDCl_3$) δ 8.03–7.98 (m, 1H), 7.97–7.92 (m, 2H), 7.93–7.89 (m, 31H), 7.87–7.82 (m, 1H), 7.57–7.42 (m, 18H), 7.41–7.21 (m, 174H), 7.23–7.09 (m, 149H), 7.10–6.92 (m, 27H), 6.93–6.89 (m, 32H), 6.88–6.82 (m, 30H), 6.53 (d, J = 3.7 Hz, 0.35H), 6.19 (d, J = 4.4 Hz, 0.65H), 5.35 (t, J = 5.0 Hz, 0.68H), 5.30 (dd, J = 9.0, 4.0 Hz, 0.38H), 5.22–5.16 (m, 1H), 5.16–5.06 (m, 15.5H), 4.94 (d, J = 11.9 Hz, 0.50H), 4.90–4.40 (m, 137H), 4.36–4.01 (m, 53H), 3.99–3.69 (m, 56H), 3.67–3.42 (m, 51H), 3.42–3.33 (m, 20H), 3.28 (dd, J = 9.1, 2.8 Hz, 1H), 3.15–3.07 (m, 17H), 3.02–2.92 (m, 14H), 2.45 (t, J = 7.2 Hz, 1.33H), 2.34 (td, J = 7.1, 2.6 Hz, 0.67H), 1.57–1.35 (m, 4H), 0.92 (t, J = 7.3 Hz, 2H), 0.86 (t, J = 7.3 Hz, 1H), 0.80 (s, 9H), -0.02 (s, 3H); ^{13}C NMR (151 MHz, $CDCl_3$) δ 166.7, 165.5, 165.2, 163.8, 138.9, 138.83, 138.78, 138.73, 138.70, 138.65, 138.60, 138.57, 138.5, 138.42, 138.39, 138.37, 138.34, 138.31, 138.1, 137.9, 134.8, 134.5, 133.1, 132.1, 130.8, 130.0, 129.8, 129.7, 129.5, 128.3, 128.24, 128.22, 128.20, 128.09, 128.05, 127.99, 127.89, 127.87, 127.84, 127.81, 127.78, 127.73, 127.67, 127.59, 127.55, 127.50, 127.47, 127.42, 127.37, 127.3, 127.24, 127.21, 127.1, 127.01, 126.97, 125.6, 125.3, 116.4, 100.8, 100.1, 99.6, 97.1, 92.4, 82.4, 80.1, 79.2, 77.7, 76.1, 76.0, 75.9, 75.63, 75.55, 75.0, 74.8, 74.3, 74.2, 74.1, 73.9, 73.5, 73.4, 73.3, 73.2, 72.9, 72.8, 72.5, 72.4, 72.2, 71.1, 69.7, 69.0, 68.2, 67.9, 62.4, 61.4, 30.7, 30.6, 29.7, 26.0, 22.1, 22.0, 19.6, 19.5, 18.1, 13.7, 13.6, -3.8, -4.9; MALDI FT-ICR MS calcd for $C_{755}H_{764}O_{162}Si$ $[M + K]^+$ 12490.101, found 12490.167.

Fully protected 64-mer 9

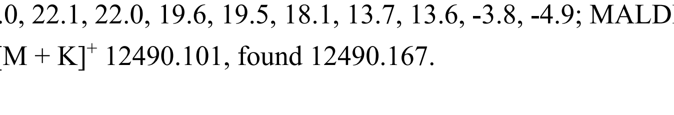

9

A pale-yellow solid: $[\alpha]_D^{25}$ = -9.3 (c 1.0, $CHCl_3$); ^{1}H NMR (600 MHz, $CDCl_3$) δ 8.03–7.99 (m, 2H), 7.97–7.88 (m, 64H), 7.56–7.47 (m, 32H), 7.40–7.29 (m, 205H), 7.28–7.22 (m, 87H), 7.21–7.10 (m, 295H), 7.08–6.94 (m, 45H), 6.93–6.89 (m, 64H), 6.88–6.81 (m, 62H), 6.78–6.75 (m, 2H), 5.40–5.35 (m, 1H), 5.22–5.17 (m, 1H), 5.16–5.07 (m, 34H), 4.89–4.43 (m, 271H), 4.38 (d, J = 8.5 Hz, 1H), 4.33–3.95 (m, 101H), 3.94–3.69 (m, 104H), 3.66–3.52 (m, 65H), 3.50–3.43 (m, 35H), 3.40–3.33 (m, 35H), 3.29–3.26 (m, 1H), 3.16–3.06 (m, 34H), 3.00–2.93 (m, 32H), 0.80 (s, 10H), -0.02 (s, 3H); ^{13}C NMR (151 MHz, $CDCl_3$) δ 165.5, 165.2, 155.3, 150.9, 138.9, 138.84, 138.80, 138.77,

138.74, 138.67, 138.64, 138.53, 138.46, 138.43, 138.38, 138.35, 138.2, 138.1, 133.1, 130.0, 129.8, 129.7, 128.5, 128.34, 128.26, 128.2, 128.1, 128.03, 127.99, 127.98, 127.95, 127.91, 127.87, 127.83, 127.79, 127.76, 127.70, 127.65, 127.59, 127.54, 127.52, 127.49, 127.46, 127.41, 127.34, 127.29, 127.2, 127.1, 127.0, 118.6, 116.4, 114.5, 100.8, 100.1, 99.8, 99.6, 82.4, 80.2, 80.1, 79.2, 77.8, 76.1, 75.9, 75.7, 75.5, 75.3, 75.2, 74.8, 74.3, 74.2, 74.1, 74.0, 73.9, 73.5, 73.4, 73.3, 73.24, 73.18, 72.79, 72.76, 72.5, 72.4, 72.3, 71.1, 70.9, 69.7, 68.3, 67.9, 62.4, 61.6, 55.6, 26.0, 18.1, -3.8, -4.9; MALDI FT-ICR MS calcd for $C_{1485}H_{1494}O_{322}SiK$ $[M + K]^+$ 24560.8, found 24558.9.

64-Mer acceptor 9A

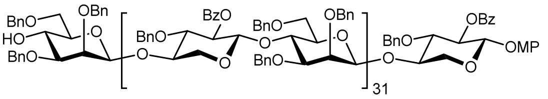

9A

A colorless amorphous solid: $[\alpha]_D^{25}$ = -12.2 (*c* 1.0, $CHCl_3$); ^{1}H NMR (500 MHz, $CDCl_3$) δ 8.05–8.00 (m, 2H), 7.99–7.87 (m, 64H), 7.57–7.48 (m, 33H), 7.40–7.30 (m, 208H), 7.28–7.24 (m, 86H), 7.21–7.11 (m, 293H), 7.09–7.04 (m, 3H), 7.02–7.00 (m, -2H), 6.99–6.95 (m, 36H), 6.94–6.90 (m, 65H), 6.89–6.83 (m, 60H), 6.79–6.74 (m, 2H), 5.38 (t, *J* = 6.2 Hz, 1H), 5.19 (t, *J* = 7.8 Hz, 1H), 5.16–5.05 (m, 32H), 4.94–4.36 (m, 290H), 4.31–4.07 (m, 113H), 3.94–3.69 (m, 109H), 3.65–3.52 (m, 68H), 3.50–3.45 (m, 31H), 3.40–3.31 (m, 37H), 3.17–3.08 (m, 32H), 3.02–2.94 (m, 34H), 2.70 (s, 1H), 0.81 (s, 0H); ^{13}C NMR (151 MHz, $CDCl_3$) δ 165.5, 165.2, 155.3, 151.0, 138.9, 138.8, 138.72, 138.67, 138.6, 138.44, 138.41, 138.35, 138.2, 138.0, 137.9, 133.2, 130.0, 129.9, 129.8, 129.7, 128.6, 128.5, 128.41, 128.39, 128.32, 128.26, 128.2, 128.11, 128.07, 128.03, 127.99, 127.96, 127.9, 127.84, 127.79, 127.74, 127.70, 127.64, 127.58, 127.5, 127.44, 127.36, 127.3, 127.2, 127.0, 118.6, 116.5, 114.5, 100.8, 100.1, 100.0, 99.9, 99.6, 81.4, 80.1, 79.3, 76.2, 75.9, 75.7, 75.6, 75.33, 75.26, 74.5, 74.4, 74.3, 74.2, 74.0, 73.8, 73.4, 73.3, 72.9, 72.8, 72.4, 71.4, 71.0, 70.7, 68.33, 68.27, 62.4, 61.7, 55.7; MALDI FT-ICR MS calcd for $C_{1479}H_{1480}O_{322}K$ $[M + K]^+$ 24446.5, found 24435.4.

64-Mer donor 9D

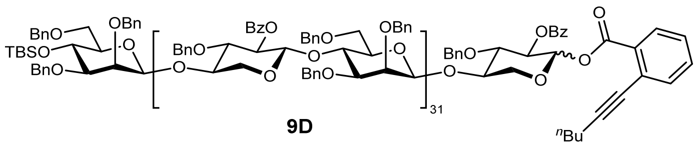

9D

A glassy solid (α/β= 0.58:1): ^{1}H NMR (600 MHz, $CDCl_3$) δ 8.03–7.82 (m, 73H), 7.57–7.43 (m, 40H), 7.40–7.28 (m, 217H), 7.29–7.23 (m, 82H), 7.21–7.10 (m, 315H), 6.99–6.93 (m, 35H), 6.93–6.89 (m, 66H), 6.89–6.82 (m, 66H), 6.53 (d, *J* = 3.7 Hz, 0.34H), 6.19 (d, *J* = 4.5 Hz, 0.59H), 5.22–5.17 (m, 1H), 5.15–5.07 (m, 35H), 4.90–4.68 (m, 112H), 4.70–4.62 (m, 65H), 4.60–4.51 (m, 71H), 4.49–4.43 (m, 36H), 4.30–4.20 (m, 67H), 4.17–4.12 (m, 34H), 3.91–3.85 (m, 36H), 3.83–3.75 (m, 37H), 3.74–3.70 (m, 35H), 3.61–3.51 (m, 70H), 3.49–3.43 (m, 33H), 3.38–3.32 (m, 35H), 3.13–3.08 (m, 36H), 3.01–2.90 (m, 31H), 2.49–2.31 (m, 2H), 1.65–1.33 (m, 5H), 0.93–0.83 (m, 4H), -0.02 (s, 3H); ^{13}C NMR (151 MHz, $CDCl_3$) δ 165.6, 165.3, 164.2, 163.9, 139.0, 138.9, 138.84, 138.77, 138.72, 138.66, 138.63, 138.58, 138.5, 138.43, 138.37, 138.1, 138.0, 134.8, 134.6, 133.2, 132.2, 130.8, 130.7, 130.1, 129.9, 129.8, 129.5, 128.4, 128.28, 128.27, 128.2, 128.13, 128.07, 128.04, 128.01, 127.98, 127.96, 127.92, 127.90, 127.87, 127.8, 127.7, 127.63, 127.55, 127.46, 127.4, 127.33, 127.30, 127.2, 127.1, 125.6, 125.3, 116.6, 100.9, 100.2, 99.7, 97.2, 92.5, 82.5, 80.2, 79.3, 77.8, 77.4, 77.2, 77.0, 76.2, 75.9, 75.6, 74.9, 74.4, 74.3, 74.0, 73.6, 73.43, 73.35, 73.29, 72.8, 72.6, 72.4, 72.2,

71.2, 69.8, 69.0, 68.3, 67.9, 62.4, 30.73, 30.65, 26.0, 22.14, 22.07, 19.63, 19.61, 18.2, 13.8, 13.7, -3.7, -4.8; MALDI FT-ICR MS calcd for $C_{1491}H_{1500}O_{322}SiK$ $[M + K]^+$ 24638.9, found 24631.7.

Fully protected 128-mer 10

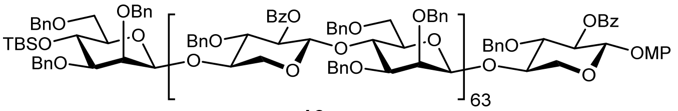

10

A glassy solid: $[\alpha]_D^{25}$ = -3.4 (*c* 0.5, $CHCl_3$); ^1H NMR (600 MHz, $CDCl_3$) δ 8.01–7.81 (m, 119H), 7.47–7.41 (m, 55H), 7.31–7.18 (m, 368H), 7.20–7.15 (m, 103H), 7.14–7.04 (m, 475H), 7.06–7.02 (m, 48H), 6.92–6.86 (m, 56H), 6.88–6.81 (m, 110H), 6.81–6.75 (m, 109H), 6.70–6.66 (m, 1H), 5.30 (t, *J* = 5.8 Hz, 1H), 5.17–5.01 (m, 64H), 4.78–4.73 (m, 86H), 4.72–4.67 (m, 76H), 4.61–4.56 (m, 111H), 4.52–4.43 (m, 116H), 4.42–4.36 (m, 58H), 4.21–4.12 (m, 108H), 4.09–4.04 (m, 52H), 3.83–3.80 (m, 65H), 3.73–3.70 (m, 48H), 3.67–3.64 (m, 60H), 3.52–3.45 (m, 109H), 3.41–3.36 (m, 48H), 3.31–3.26 (m, 66H), 3.05–3.00 (m, 57H), 2.91–2.88 (m, 58H), 0.73 (s, 9H), -0.08 (s, 6H); ^{13}C NMR (151 MHz, $CDCl_3$) δ 164.1, 149.9, 137.8, 137.7, 137.39, 137.35, 132.1, 128.8, 128.7, 127.3, 127.2, 127.0, 126.8, 126.74, 126.69, 126.6, 126.4, 126.33, 126.27, 126.2, 126.0, 117.5, 113.4, 99.8, 98.6, 79.1, 78.2, 76.2, 76.0, 75.8, 75.1, 74.9, 74.5, 73.2, 72.9, 72.3, 72.2, 71.7, 71.4, 67.2, 61.3, 54.6, 24.9, 0.0, -4.8, -6.0; MALDI FT-ICR MS calcd for $C_{2957}H_{2966}O_{642}SiK$ $[M + K]^+$ 48816, found 48813.

128-Mer acceptor 10A

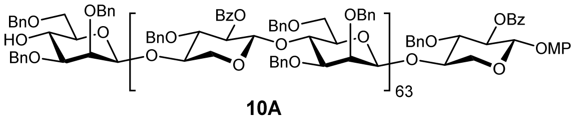

10A

A glassy solid: $[\alpha]_D^{25}$ = -8.6 (*c* 0.5, $CHCl_3$); ^1H NMR (500 MHz, $CDCl_3$) δ 8.10–7.80 (m, 125H), 7.64–7.47 (m, 64H), 7.41–7.29 (m, 383H), 7.27 (s, 31H), 7.25–7.24 (m, 33H), 7.22–7.09 (m, 551H), 6.99–6.94 (m, 67H), 6.93–6.88 (m, 119H), 6.80–6.73 (m, 2H), 5.37 (dd, *J* = 7.0, 5.5 Hz, 1H), 5.22–5.16 (m, 1H), 5.15–5.07 (m, 62H), 4.89–4.73 (m, 191H), 4.72–4.61 (m, 122H), 4.60–4.50 (m, 123H), 4.50–4.43 (m, 65H), 4.32–4.17 (m, 123H), 4.17–4.10 (m, 60H), 3.94–3.83 (m, 68H), 3.83–3.73 (m, 56H), 3.74–3.69 (m, 63H), 3.61–3.50 (m, 128H), 3.50–3.41 (m, 52H), 3.40–3.29 (m, 71H), 3.16–3.06 (m, 62H), 2.97 (s, 64H), 2.71 (d, *J* = 1.9 Hz, 1H); ^{13}C NMR (126 MHz, $CDCl_3$) δ 165.5, 165.2, 155.3, 151.0, 138.83, 138.77, 138.61, 138.59, 138.5, 138.4, 138.2, 138.0, 137.9, 133.1, 130.0, 129.84, 129.75, 128.5, 128.39, 128.36, 128.3, 128.2, 128.1, 128.0, 127.93, 127.85, 127.78, 127.7, 127.6, 127.53, 127.48, 127.4, 127.3, 127.2, 127.0, 118.6, 116.4, 114.5, 100.8, 100.1, 100.0, 99.9, 99.6, 81.4, 80.2, 79.2, 77.29, 77.25, 77.0, 76.8, 76.1, 75.9, 75.6, 75.3, 75.1, 74.4, 74.2, 74.0, 73.8, 73.4, 73.3, 72.8, 72.4, 71.4, 70.8, 68.3, 62.4, 61.6, 55.6; MALDI FT-ICR MS calcd for $C_{2591}H_{2952}O_{642}K$ $[M + K]^+$ 48702, found 48701.

128-Mer donor 10D

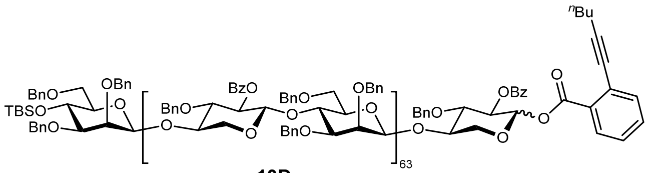

10D

A glassy solid (α/β= 0.58:1): ^{1}H NMR (500 MHz, $CDCl_3$) δ 7.93 (d, J = 7.6 Hz, 127H), 7.52 (d, J = 7.3 Hz, 66H), 7.42–7.29 (m, 397H), 7.31–7.22 (m, 147H), 7.23–7.10 (m, 572H), 6.98 (t, J = 7.4 Hz, 67H), 6.93 (d, J = 7.4 Hz, 119H), 6.90–6.83 (m, 113H), 6.55 (d, J = 3.7 Hz, 0.29H), 6.20 (d, J = 4.4 Hz, 0.50H), 5.37 (t, J = 5.0 Hz, 0.52H), 5.24–5.10 (m, 64H), 4.92–4.73 (m, 194H), 4.74–4.62 (m, 127H), 4.62–4.52 (m, 131H), 4.51–4.45 (m, 66H), 4.32–4.20 (m, 120H), 4.19–4.13 (m, 62H), 3.94–3.86 (m, 59H), 3.84–3.76 (m, 64H), 3.74 (s, 64H), 3.63–3.52 (m, 116H), 3.52–3.45 (m, 55H), 3.42–3.32 (m, 72H), 3.17–3.09 (m, 62H), 3.07–2.93 (m, 61H), 2.56–2.31 (m, 2H), 1.64–1.35 (m, 4H), 0.98–0.84 (m, 3H), 0.82 (s, 10H), -0.00 (s, 3H); ^{13}C NMR (151 MHz, $CDCl_3$) δ 165.52, 165.50, 165.2, 164.2, 163.8, 139.06, 139.03, 139.00, 138.97, 138.93, 138.90, 138.85, 138.79, 138.73, 138.70, 138.64, 138.60, 138.58, 138.55, 138.48, 138.42, 138.28, 138.24, 138.20, 138.18, 138.12, 137.97, 134.8, 134.5, 133.1, 132.2, 130.8, 130.7, 130.3, 130.08, 130.06, 129.90, 129.85, 129.77, 129.51, 129.45, 128.38, 128.2, 128.12, 128.08, 128.0, 127.9, 127.8, 127.7, 127.63, 127.56, 127.5, 127.4, 127.3, 127.23, 127.22, 127.0, 125.6, 125.3, 116.5, 100.8, 100.2, 99.6, 97.1, 97.0, 92.5, 90.7, 82.5, 80.2, 79.2, 79.1, 77.8, 77.33, 77.28, 77.1, 76.8, 76.1, 75.9, 75.64, 75.57, 75.0, 74.8, 74.3, 74.2, 74.1, 74.0, 73.6, 73.5, 73.4, 73.3, 73.2, 72.8, 72.6, 72.5, 72.4, 72.2, 71.6, 71.2, 69.8, 69.0, 68.3, 67.9, 62.4, 61.4, 30.7, 30.6, 26.0, 22.1, 22.0, 19.60, 19.58, 18.2, 13.8, 13.7, 13.7, -3.8, -4.9; MALDI FT-ICR MS calcd for $C_{2963}H_{2972}O_{642}SiK$ $[M + K]^+$ 48894, found 48893.

Fully protected 256-mer 11

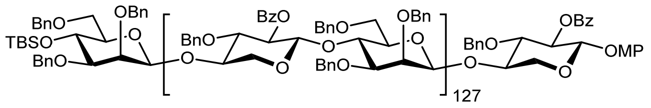

11

A glassy solid: $[\alpha]_D^{25}$ = -9.0 (c 0.5, $CHCl_3$); ^{1}H NMR (600 MHz, $CDCl_3$) δ 8.10–7.78 (m, 222H), 7.54–7.49 (m, 105H), 7.39–7.28 (m, 672H), 7.28–7.23 (m, 192H), 7.20–7.11 (m, 1000H), 6.99–6.93 (m, 111H), 6.93–6.89 (m, 213H), 6.88–6.82 (m, 208H), 5.37 (t, 1H), 5.23–5.02 (m, 128H), 4.89–4.72 (m, 335H), 4.70–4.62 (m, 209H), 4.60–4.50 (m, 213H), 4.50–4.41 (m, 107H), 4.34–4.18 (m, 202H), 4.18–4.12 (m, 105H), 3.88 (s, 101H), 3.78 (s, 117H), 3.72 (s, 109H), 3.60–3.51 (m, 226H), 3.46 (s, 81H), 3.40–3.30 (m, 128H), 3.16–3.06 (m, 105H), 2.97 (s, 123H), 0.81 (s, 9H), -0.10 (s, 1H); ^{13}C NMR (151 MHz, $CDCl_3$) δ 165.2, 139.0, 138.83, 138.79, 138.72, 138.70, 138.5, 138.4, 138.1, 133.1, 129.81, 129.78, 128.5, 128.3, 128.2, 128.0, 127.93, 127.90, 127.83, 127.74, 127.69, 127.59, 127.54, 127.51, 127.46, 127.32, 127.28, 127.2, 127.0, 118.6, 114.5, 100.8, 99.6, 82.5, 80.2, 79.2, 77.2, 77.0, 76.8, 76.1, 75.9, 75.6, 74.8, 74.2, 74.1, 74.0, 73.5, 73.4, 73.2, 72.8, 72.4, 71.1, 68.3, 67.9, 62.4, 55.6, 29.7, 26.0, 18.1, -3.8, -4.9; MALDI FT-ICR MS calcd for $C_{5901}H_{5910}O_{1282}SiK$ $[M + K]^+$ 97453, found 97313.

2.4 Details for the purification and characterization of fully protected glycans 5-11

Notably, 16-mer **7** and 32-mer **8** showed spot expansion of elution properties on silica gel thin-layer

chromatography (TLC). Purification of these two glycans was achieved via silica gel column chromatography by judicious choice of the eluents. Purification of longer glycans including 64-mer **9**, 128-mer **10**, and 256-mer **11** was carried out using gel permeation chromatography (GPC).

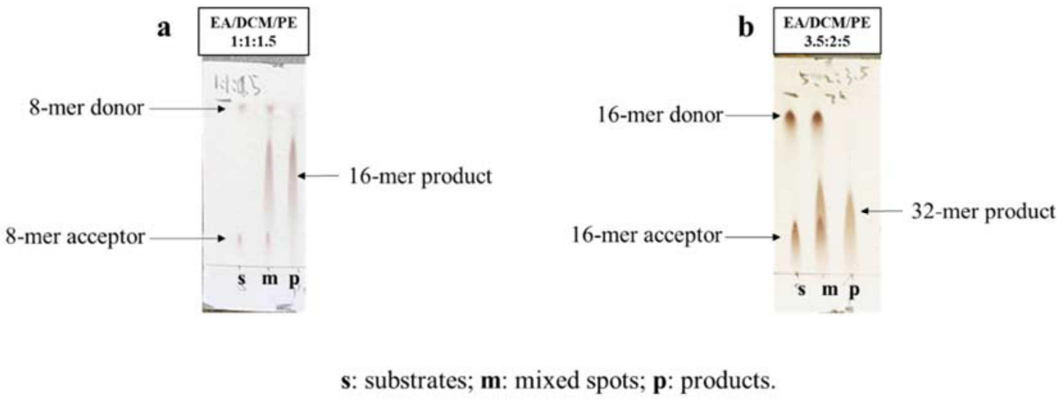

Figure S5. The TLC plates for the assemblies of 16-mer **7** and 32-mer **8**. **a**, TLC analysis of the [8+8] glycosylation for the preparation of 16-mer **7**. **b**, TLC analysis of the [16+16] glycosylation for the preparation of 32-mer **8**. EA, ethyl acetate; DCM, dichloromethane; PE, petroleum ether.

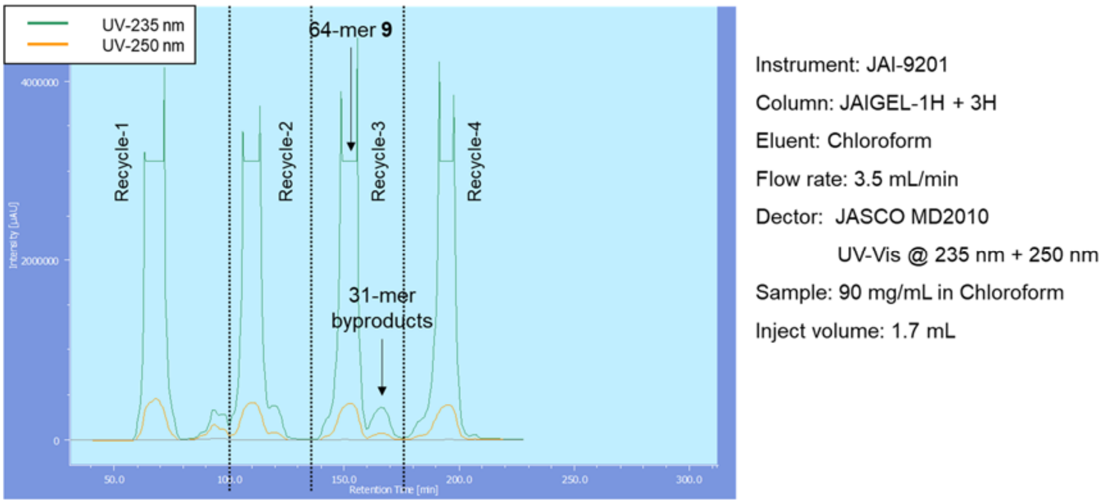

Figure S6. The preparative GPC traces of 64-mer **9** after [32+32] glycosylation.

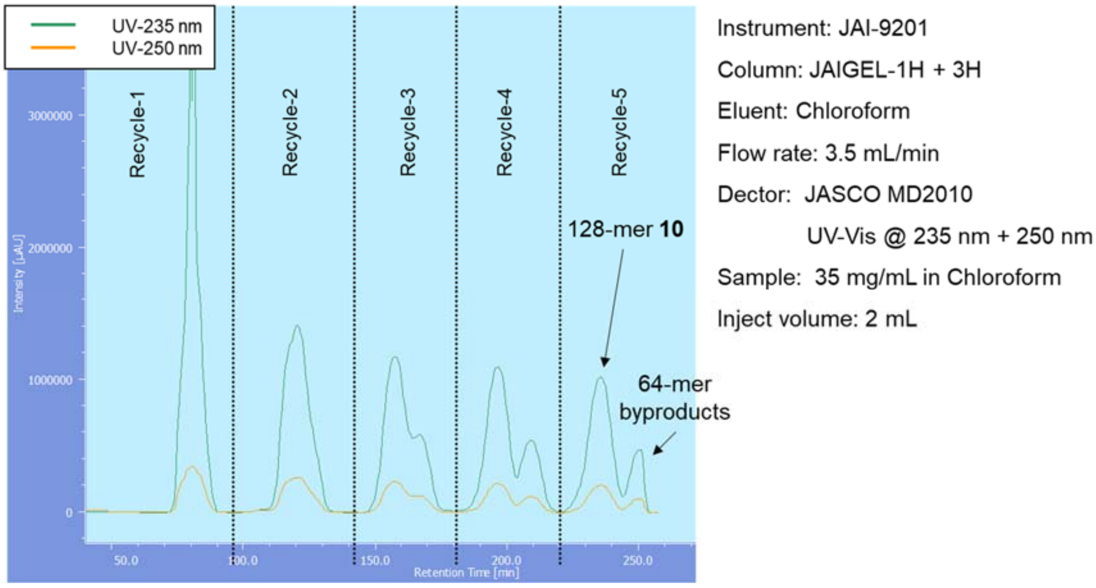

Instrument: JAI-9201

Column: JAIGEL-1H + 3H

Eluent: Chloroform

Flow rate: 3.5 mL/min

Dector: JASCO MD2010

UV-Vis @ 235 nm + 250 nm

Sample: 35 mg/mL in Chloroform

Inject volume: 2 mL

Figure S7. The preparative GPC traces of 128-mer **10** after [64+64] glycosylation.

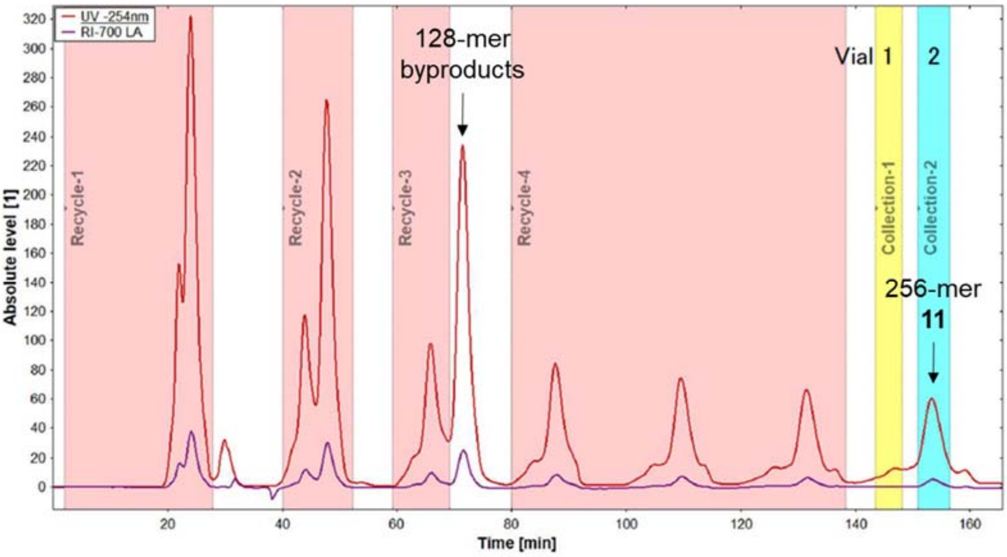

Instrument : LaboACE LC-5060

Column : JAIGEL-4HR + 5HR

Eluent : Chloroform

Flow rate : 10 ml/min

Detector : UV-Vis 4ch LA @ 254 nm and RI-700 LA

Sample : 24 mg/2 ml

Injection Volume : 2.0 ml

Figure S8. The preparative GPC traces of 256-mer **11** after [128+128] glycosylation.

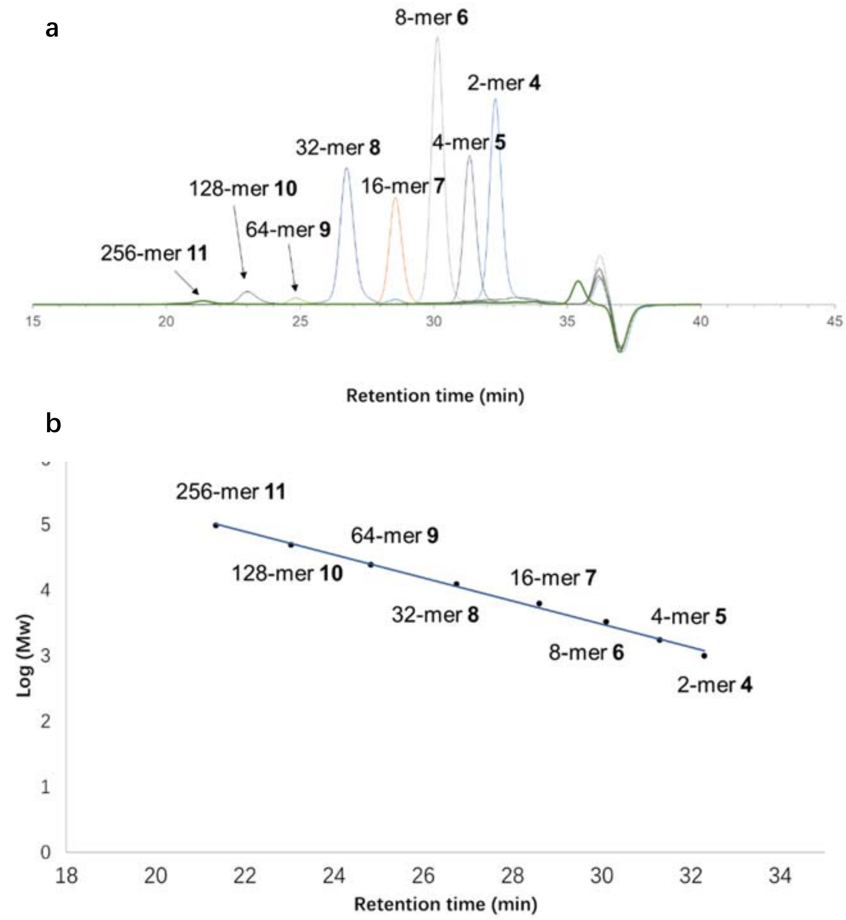

Figure S9. GPC profiles of the fully protected glycans **4-11**. **a**, Overlaid GPC traces of glycans 2-mer **4**, 4-mer **5**, 8-mer **6**, 16-mer **7**, 32-mer **8**, 64-mer **9**, 128-mer **10**, and 256-mer **11**. **b**, The plot of Log(Mw) over retention time. Mw, molecular weight.

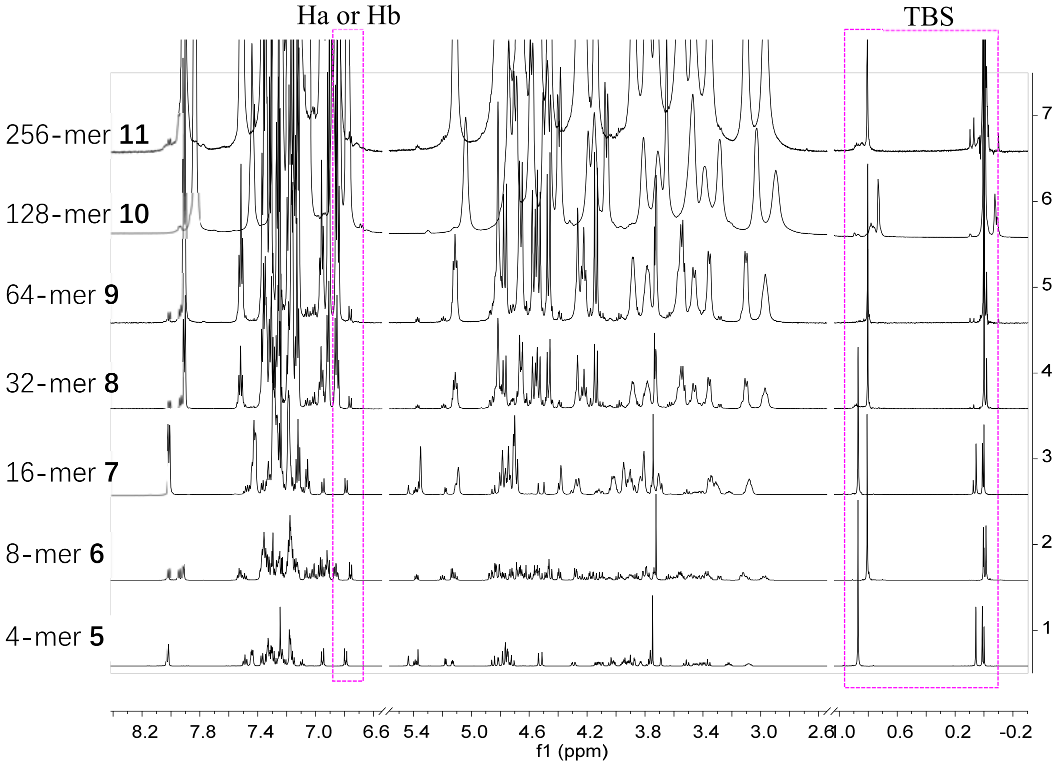

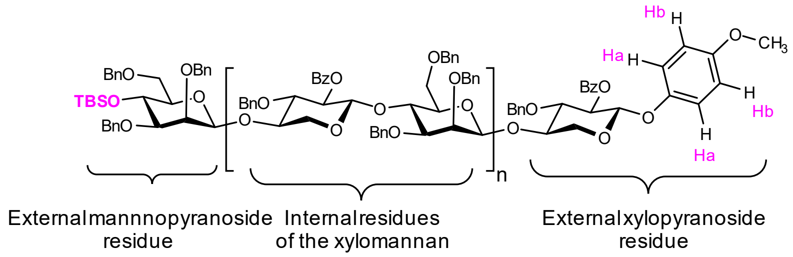

Figure S10. The overlaid ^{1}H NMR spectra of the fully protected H[→4)-*β*-D-Man*p*-(1→4)-*β*-D-Xyl*p*-(1→]$_n$OMP. Marked in the pink dashed box are the resonance peaks of TBS and MP groups. The external mannopyranoside and xylopyranoside residues are merging into baselines as the elongation of glycans.

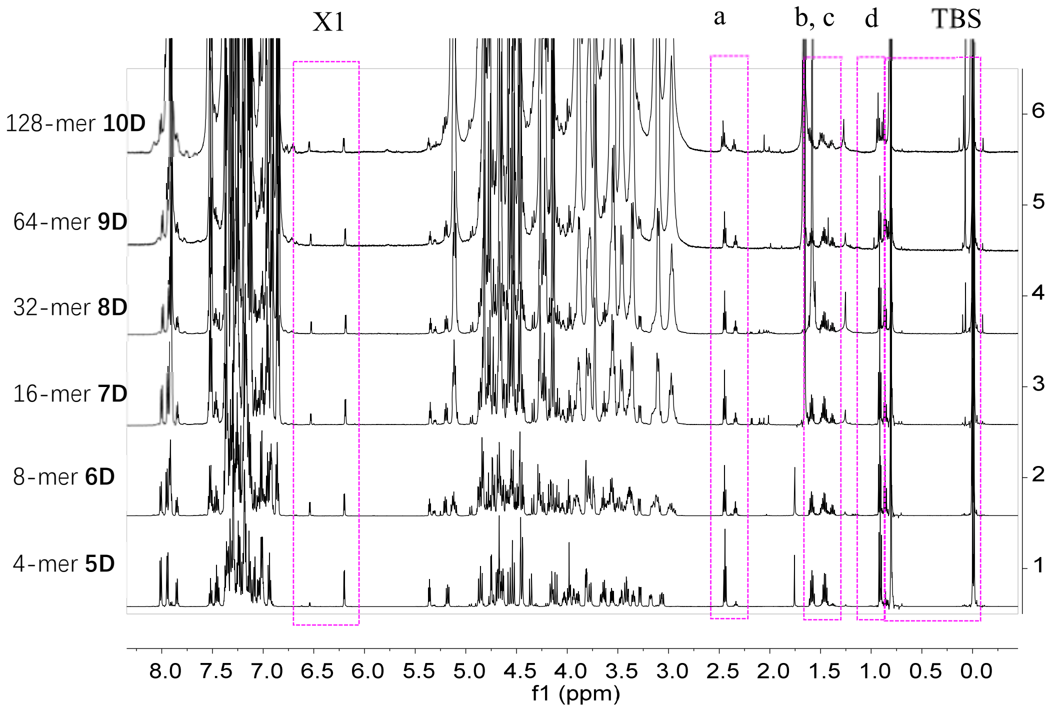

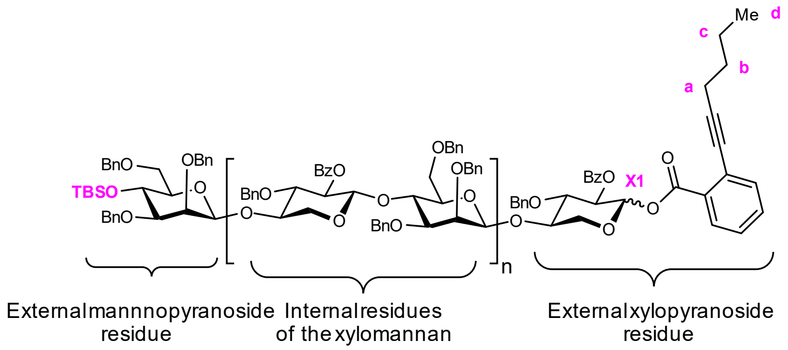

Figure S11. The overlaid ^{1}H NMR spectra of *o*-hexynylbenzoate donors **5D-10D**. Marked in the pink dashed box are the resonance peaks of the TBS group at the nonreducing end, the anomeric proton at the reducing end, and the *n*-butyl group at the leaving group. The external mannopyranoside and xylopyranoside residues are merging into baselines as the elongation of glycans.

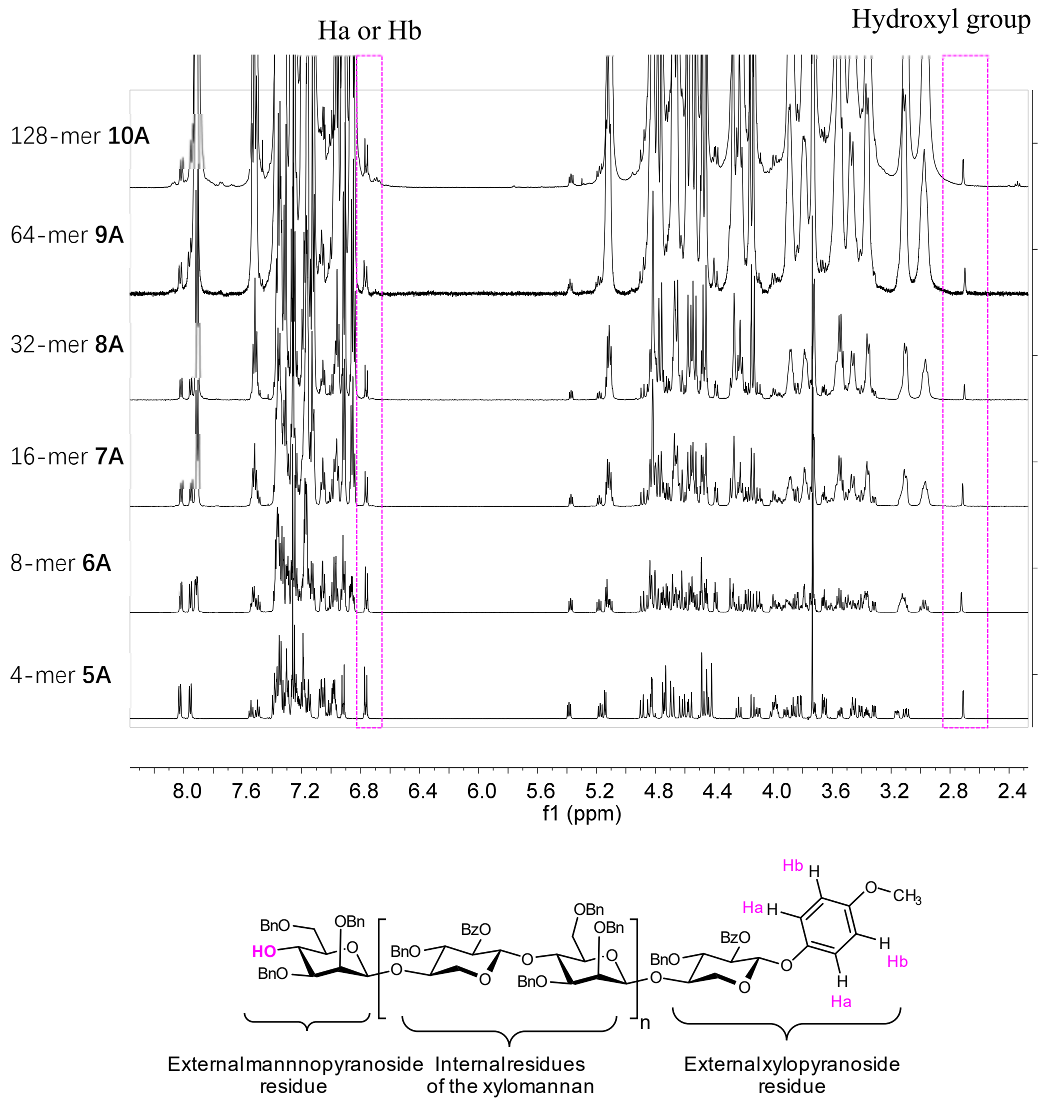

Figure S12. The overlaid ^{1}H NMR spectra of glycosyl acceptors **5A-10A**. Marked in the pink dashed box are the resonance peaks of the hydroxyl group at the nonreducing end and the MP group at the reducing end. The external mannopyranoside and xylopyranoside residues are merging into baselines as the elongation of glycans.

2.5 Preparation of free glycans 4F-9F

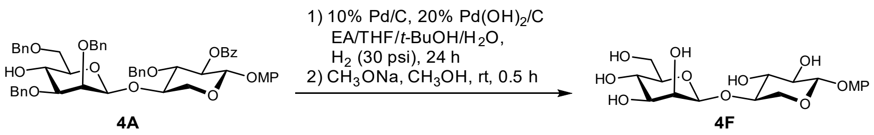

2-Mer **4A** (30 mg) was dissolved in a mixture of EA/THF/*t*-BuOH/H_2O (2/2/2/1, 5 mL) containing 10% Pd/C (30 mg, wetted with 55% H_2O) and 20% $Pd(OH)_2/C$ (30 mg, wetted with 50% H_2O). The mixture was stirred under H_2 atmosphere (30 psi) at 30 °C for 24 hours, and was then filtrated through a pad of Celite. The Celite pad was washed with MeOH/$CHCl_3$ (1:1, 6 mL), MeOH/$CHCl_3$ (2:1, 6 mL), MeOH/$CHCl_3$ (3:1, 4 mL), MeOH (3 mL), and $CHCl_3$ (3 mL) successively. The

combined filtrate was concentrated to afford a solid.
To the solution of the above solid in CH_3OH (5 mL) was added CH_3ONa (15 mg, excess). The resulting solution was stirred at room temperature for 0.5 hour, and was then neutralized with Dowex 50 WX8 (H) resin. The mixture was filtrated through a pad of Celite, and the Celite pad was washed with H_2O. The combined filtrate was concentrated *in vacuo* and purified by gel filtration (Sephadex G-25, H_2O) to give **4F** as a white powder after lyophilization (13 mg, 91% over two steps). Compound **4F** is highly soluble in water (> 10 mg/mL).
^1H NMR (600 MHz, D_2O, 40 °C, δ_{HDO} = 4.60 ppm) δ 7.11–7.05 (m, 2H), 7.00–6.94 (m, 2H), 4.96 (d, J = 7.7 Hz, 1H), 4.77 (d, J = 1.1 Hz, 1H), 4.11 (dd, J = 11.8, 5.4 Hz, 1H), 3.97 (dd, J = 3.2, 1.0 Hz, 1H), 3.94–3.89 (m, 2H), 3.79 (s, 3H), 3.73 (dd, J = 12.3, 6.6 Hz, 1H), 3.69 (t, J = 9.1 Hz, 1H), 3.62 (dd, J = 9.6, 3.2 Hz, 1H), 3.56 (t, J = 9.6 Hz, 1H), 3.56 (dd, J = 9.3, 7.6 Hz, 1H), 3.47 (dd, J = 11.8, 10.2 Hz, 1H), 3.38 (ddd, J = 9.3, 6.6, 2.3 Hz, 1H); ^{13}C NMR (151 MHz, D_2O, 40 °C, δ_{DSS} = 0.0 ppm) δ 157.6, 153.3, 121.1, 117.8, 104.4, 101.1, 79.0, 78.9, 76.4, 75.6, 75.3, 73.5, 69.5, 65.6, 63.7, 58.6; HRMS (ESI) calcd for $C_{18}H_{26}O_{11}Na$ $[M + Na]^+$ 441.1373, found 441.1371.

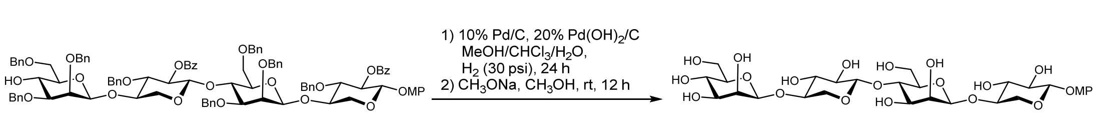

4-Mer **5A** (34 mg) was dissolved in a mixture of $MeOH/CHCl_3/H_2O$ (3/3/0.1, 6.1 mL) containing 10% Pd/C (34 mg, wetted with 55% H_2O) and 20% $Pd(OH)_2/C$ (34 mg, wetted with 50% H_2O). The resulting mixture was stirred under H_2 atmosphere (30 psi) at 30 °C for 24 hours, and was then filtrated through a pad of Celite. The Celite pad was washed with $MeOH/CHCl_3$ (1:1, 6 mL), $MeOH/CHCl_3$ (2:1, 6 mL), $MeOH/CHCl_3$ (3:1, 4 mL), MeOH (3 mL), and $CHCl_3$ (3 mL) successively. The combined filtrate was concentrated to afford a solid.
To a solution of the above solid in CH_3OH (5 mL) was added CH_3ONa (15 mg, excess). The solution was stirred at room temperature for 12 hours, and was then neutralized with Dowex 50 WX8 (H) resin. The resulting mixture was filtrated through a pad of Celite, and the Celite pad was washed with H_2O. The combined filtrate was concentrated under reduced pressure and purified by gel filtration (Sephadex G-25, H_2O) to give **5F** as a white powder after lyophilization (10 mg, 72% over two steps). This compound is highly soluble in water (> 10 mg/mL).
^1H NMR (600 MHz, D_2O, 40 °C, δ_{HDO} = 4.60 ppm) δ 7.10 (d, J = 8.6 Hz, 2H), 6.99 (d, J = 9.0 Hz, 2H), 4.98 (d, J = 7.7 Hz, 1H), 4.82 (s, 1H), 4.78 (s, 1H), 4.43 (d, J = 7.8 Hz, 1H), 4.13 (dt, J = 11.2, 5.0 Hz, 2H), 4.04–3.97 (m, 3H), 3.96–3.91 (m, 2H), 3.90–3.68 (m, 11H), 3.66–3.52 (m, 6H), 3.49 (t, J = 11.0 Hz, 1H), 3.41–3.32 (m, 4H); ^{13}C NMR (151 MHz, D_2O, 40 °C, δ_{DSS} = 0.0 ppm) δ 157.6, 153.4, 121.19, 121.16, 117.9, 105.9, 104.5, 101.1, 101.0, 79.02, 78.97, 78.8, 77.9, 76.6, 76.5, 75.63, 75.60, 75.3, 74.2, 73.5, 73.2, 69.5, 65.7, 65.6, 65.3, 63.8, 63.1, 58.7; HRMS (ESI) calcd for $C_{29}H_{44}O_{20}Na$ $[M + Na]^+$ 735.2324, found 735.2324.

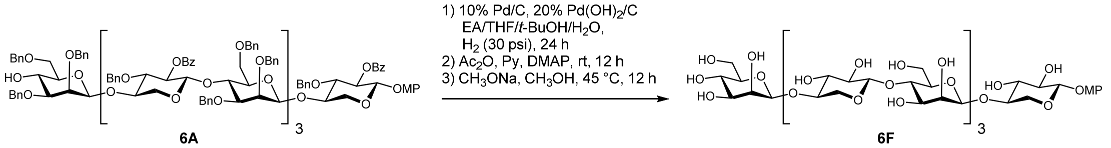

8-Mer **6A** (60 mg) was dissolved in a mixture of EtAc/THF/*t*-BuOH/H_2O (2/2/2/1, 5 mL) containing 10% Pd/C (60 mg, wetted with 55% H_2O) and 20% $Pd(OH)_2/C$ (60 mg, wetted with 50% H_2O). The

resulting mixture was stirred under H_2 atmosphere (30 psi) at 30 °C for 24 hours. The reaction mixture was concentrated directly under reduced pressure and the residue was azeotropically dried with pyridine three times to give a black residue.
To the above black residue were added dry pyridine (4 mL, excess), 4-dimethylaminopyridine (4 mg), and acetic anhydride (2 mL, excess). The reaction mixture was stirred at room temperature for 12 hours, and then methanol (1 mL) was added at 0 °C to quench the excess acetic anhydride. The resulting mixture was filtered through a pad of Celite, and the Celite pad was washed with ethyl acetate three times. The combined filtrate was concentrated to give a yellow residue, which was dissolved in ethyl acetate and then washed with 1N HCl and saturated aqueous $NaHCO_3$ successively. The organic layer was dried over anhydrous Na_2SO_4, concentrated *in vacuo*, and purified by gel permeation chromatography (LH-20, MeOH/CH_2Cl_2 = 1:1) to give a white solid.
To the suspension of the above solid in CH_3OH (5 mL) was added CH_3ONa (15 mg, excess). The suspension was stirred vigorously at 45 °C for 12 hours. The resulting white suspension was neutralized with Dowex 50 WX8 (H) resin, and filtrated through a pad of Celite. The Celite pad was washed with H_2O. The combined filtrate was concentrated to afford a residue, which was purified by gel filtration (Sephadex LH-60, H_2O) to give **6F** as a white powder after lyophilization (22 mg, 89% over two steps). 8-Mer **6F** is moderately soluble in water (> 10 mg/mL).
^{1}H NMR (600 MHz, D_2O, 40 ºC, δ_{HDO} = 4.60 ppm) δ 7.11–7.05 (m, 2H), 6.99–6.94 (m, 2H), 4.96 (d, J = 7.7 Hz, 1H), 4.79 (d, J = 1.0 Hz, 1H), 4.76 (d, J = 1.0 Hz, 2H), 4.75 (d, J = 1.0 Hz, 1H), 4.40 (dd, J = 7.8, 3.1 Hz, 3H), 4.10 (ddd, J = 11.8, 6.6, 5.3 Hz, 4H), 4.02–3.94 (m, 8H), 3.94–3.88 (m, 2H), 3.87–3.81 (m, 4H), 3.81–3.76 (m, 6H), 3.76–3.67 (m, 9H), 3.64–3.45 (m, 11H), 3.39–3.28 (m, 7H); ^{13}C NMR (151 MHz, D_2O, 40 ºC, δ_{DSS} = 0.0 ppm) δ 157.7, 153.4, 121.2, 117.9, 106.0, 104.5, 101.1, 101.02, 100.96, 79.1, 79.0, 78.93, 78.88, 77.92, 77.91, 76.7, 76.6, 76.5, 75.7, 75.4, 74.2, 73.5, 73.2, 69.6, 65.73, 65.69, 65.67, 63.8, 63.1, 58.7; HRMS (ESI) calcd for $C_{51}H_{80}O_{38}Na$ [M + Na]$^+$ 1323.4225, found 1323.4225.

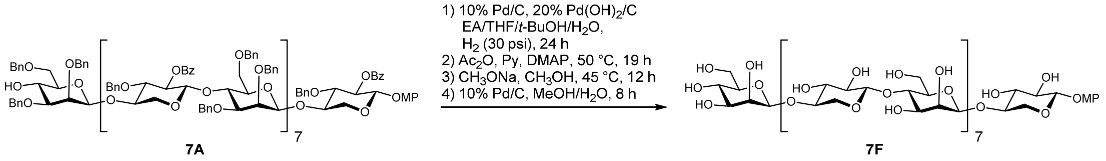

16-Mer **7A** (60 mg) was dissolved in a mixture of EtOAc/THF/*t*-BuOH/H_2O (2/2/2/1, 12 mL) containing 10% Pd/C (90 mg, wetted with 55% H_2O) and 20% $Pd(OH)_2$/C (90 mg, wetted with 50% H_2O). The resulting mixture was stirred under H_2 atmosphere (30 psi) at 30 °C for 24 hours. The reaction mixture was concentrated directly and azeotropically dried with pyridine three times to give a black residue.
To the above residue were added dry pyridine (4 mL, excess), 4-dimethylaminopyridine (4 mg) and acetic anhydride (2 mL, excess). The reaction mixture was stirred at 50 °C for 19 hours, and then methanol (1 mL) was added at 0 °C to quench the excess acetic anhydride. The resulting mixture was filtered through a pad of Celite, and the Celite pad was washed with ethyl acetate three times. The combined filtrate was concentrated to give a yellow residue, which was dissolved in ethyl acetate and then washed with 1N HCl and saturated aqueous $NaHCO_3$ successively. The organic layer was dried over anhydrous Na_2SO_4, concentrated *in vacuo*, and purified by gel permeation chromatography (LH-20, MeOH/CH_2Cl_2 = 1:1) to give a white solid.
To the suspension of the solid above in CH_3OH (5 mL) was added CH_3ONa (15 mg, excess). The

suspension was stirred vigorously at 50 °C for 12 hours. The resulting white suspension was neutralized with Dowex 50 WX8 (H) resin, and was then filtrated through a pad of Celite. The Celite pad was washed with H_2O. The combined filtrate was concentrated to afford a residue, which was purified by gel filtration (Sephadex LH-60, H_2O) to give a white powder after lyophilization (23 mg). A few benzyl groups were remained as detected by ^{1}H NMR analysis.

The above residue was dissolved in a mixture of MeOH/H_2O (4/8, 12 mL) containing 10% Pd/C (18 mg, wetted with 55% H_2O). The resulting mixture was stirred under H_2 atmosphere (30 psi) at 30 °C for 8 hours, and was then filtered through microporous membrane. The filtrate was concentrated *in vacuo* to give **7F** as a white solid after lyophilization (12 mg, 50% over four steps). 16-Mer **7F** shows moderate solubility in water (3.5 mg/mL).

^{1}H NMR (600 MHz, D_2O, 40 °C, δ_{HDO} = 4.60 ppm) δ 7.23–7.16 (m, 2H), 7.12–7.02 (m, 2H), 5.06 (d, J = 7.6 Hz, 1H), 4.89 (s, 1H), 4.87 (s, 6H), 4.85 (s, 1H), 4.54–4.48 (m, 7H), 4.24–4.16 (m, 10H), 4.13–4.04 (m, 16H), 4.04–3.99 (m, 2H), 3.97–3.92 (m, 8H), 3.92–3.87 (m, 11H), 3.87–3.77 (m, 16H), 3.73–3.54 (m, 20H), 3.49–3.40 (m, 17H); ^{13}C NMR (151 MHz, D_2O, 40 °C, δ_{DSS} = 0.0 ppm) δ 157.7, 153.4, 121.2, 117.9, 106.0, 104.5, 101.1, 101.02, 100.96, 79.1, 79.0, 78.93, 78.88, 77.9, 76.65, 76.62, 76.51, 75.7, 75.4, 74.2, 73.5, 73.2, 69.6, 65.7, 63.8, 63.1, 58.7; MALDI FT-ICR MS calcd for $C_{95}H_{152}O_{74}K$ $[M + K]^+$ 2499.802, found 2499.794.

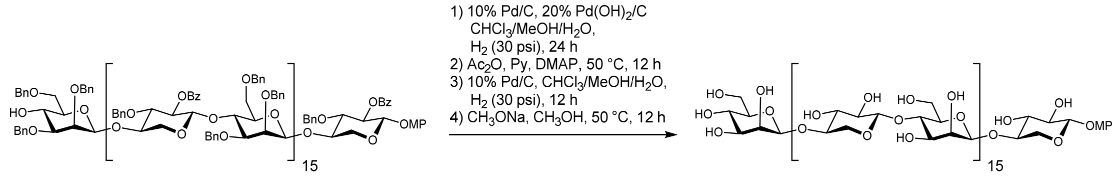

8A **8F**

32-Mer **8A** (30 mg) was dissolved in a mixture of MeOH/$CHCl_3$/H_2O (6/6/0.2, 12.2 mL) containing 10% Pd/C (60 mg, wetted with 55% H_2O) and 20% $Pd(OH)_2$/C (60 mg, wetted with 50% H_2O). The resulting mixture was stirred under an H_2 atmosphere (30 Psi) at 30 °C for 24 hours. The reaction mixture was concentrated directly and then azeotropically dried with pyridine three times to give a black residue.

To the above residue were added dry pyridine (5 mL, excess), 4-dimethylaminopyridine (4 mg), and acetic anhydride (2.5 mL, excess). The mixture was stirred at 50 °C for 12 hours, and then methanol (1 mL) was added at 0 °C to quench the excess acetic anhydride. The resulting mixture was filtered through a pad of Celite, and the Celite pad was washed with ethyl acetate three times. The combined filtrate was concentrated to give a yellow residue, which was dissolved in ethyl acetate and then washed with 1N HCl and saturated aqueous $NaHCO_3$ successively. The organic layer was dried over anhydrous Na_2SO_4, concentrated *in vacuo*, and purified by gel permeation chromatography (LH-20, MeOH/CH_2Cl_2 = 1:1) to give a white solid (20 mg). ^{1}H NMR analysis of the white solid indicated the remaining of a few benzyl groups.

The above solid was dissolved in a mixture of MeOH/$CHCl_3$/H_2O (6/6/0.2, 12.2 mL) containing 10% Pd/C (30 mg, wetted with 55% H_2O). The resulting mixture was stirred under H_2 atmosphere (30 Psi) at 30 °C for 12 hours, and was then filtrated through a pad of Celite. The Celite pad was washed with MeOH/$CHCl_3$ (1:1, 6 mL), MeOH/$CHCl_3$ (2:1, 6 mL), MeOH/$CHCl_3$ (3:1, 4 mL), MeOH (3 mL), $CHCl_3$ (3 mL) successively. The combined filtrate was concentrated to afford a solid (16 mg).

To a suspension of the above solid (16 mg) in CH_3OH (2.5 mL) was added CH_3ONa (10 mg, excess). The resulting mixture was stirred vigorously at 50 °C for 12 hours to give a white slurry. The slurry

was centrifuged at 5000 rpm for 4 minutes, and the supernatant was removed. The white solid thus obtained was further washed multiple times with MeOH to remove CH_3ONa completely. 32-Mer **8F** was obtained as a white solid after lyophilization (7 mg, 60% over four steps).

^{1}H NMR (600 MHz, D_2O, 40 °C, δ_{HDO} = 4.60 ppm) δ 7.07 (d, J = 9.1 Hz, 2H), 6.96 (d, J = 9.2 Hz, 2H), 4.95 (d, J = 7.6 Hz, 1H), 4.80–4.70 (m, 19H), 4.39 (d, J = 7.8 Hz, 15H), 4.12–4.05 (m, 17H), 4.01–3.93 (m, 29H), 3.92–3.86 (m, 2H), 3.86–3.81 (m, 14H), 3.80–3.76 (m, 17H), 3.76–3.67 (m, 35H), 3.62–3.56 (m, 22H), 3.52–3.47 (m, 16H), 3.38–3.33 (m, 22H), 3.32–3.29 (m, 24H); ^{13}C NMR (151 MHz, D_2O, 40 °C, δ_{DSS} = 0.0 ppm) δ 106.0, 101.0, 79.1, 78.9, 77.9, 76.7, 75.7, 74.2, 73.2, 63.1; MALDI FT-ICR MS calcd for $C_{183}H_{296}O_{146}K$ $[M + K]^+$ 4852.563, found 4852.574.

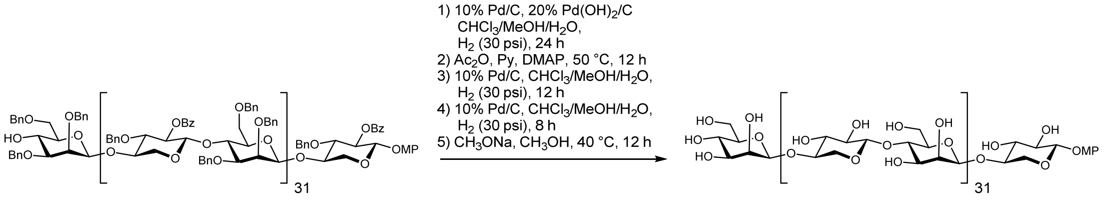

9A **9F**

64-Mer **9A** (60 mg) was dissolved in a mixture of $MeOH/CHCl_3/H_2O$ (10/6/0.2, 16.2 mL) containing 10% Pd/C (120 mg, wetted with 55% H_2O) and 20% $Pd(OH)_2/C$ (120 mg, wetted with 50% H_2O). The mixture was stirred under an H_2 atmosphere (30 Psi) at 30 °C for 24 hours. The reaction mixture was concentrated directly and then azeotropically dried with pyridine three times to give a black residue.

To the above residue were added dry pyridine (5 mL, excess), 4-dimethylaminopyridine (4 mg), and acetic anhydride (2.5 mL, excess). The reaction mixture was stirred at 50 °C for 12 hours, and then methanol (1 mL) was added at 0 °C to quench the excess acetic anhydride. The resulting mixture was filtered through a pad of Celite. The Celite pad was washed with ethyl acetate three times. The combined filtrate was concentrated to give a yellow residue, which was dissolved in ethyl acetate and then washed with 1N HCl and saturated aqueous $NaHCO_3$ successively. The organic layer was dried over anhydrous Na_2SO_4, concentrated *in vacuo*, and purified by gel permeation chromatography (LH-20, $MeOH/CH_2Cl_2$ = 1:1) to give a white solid (56 mg). ^{1}H NMR analysis of the white solid indicated the remaining of a few benzyl groups.

The above white solid (56 mg) was dissolved in a mixture of $MeOH/CHCl_3/H_2O$ (6/3/0.2, 9.2 mL) containing 10% Pd/C (120 mg, wetted with 55% H_2O). The mixture was stirred under H_2 atmosphere (30 Psi) at 30 °C for 12 hours, and was then filtrated through a pad of Celite. The Celite pad was washed with $MeOH/CHCl_3$ (1:1, 6 mL), $MeOH/CHCl_3$ (2:1, 6 mL), $MeOH/CHCl_3$ (3:1, 4 mL), MeOH (3 mL), $CHCl_3$ (3 mL) successively. The combined filtrate was concentrated to afford a solid (21 mg). ^{1}H NMR analysis of the solid indicated the remaining of a few benzyl groups.

The above white solid (21 mg) was dissolved in a mixture of $MeOH/CHCl_3/H_2O$ (6/3/0.1, 9.1 mL) containing 10% Pd/C (42 mg, wetted with 55% H_2O). The mixture was stirred under H_2 atmosphere (30 Psi) at 30 °C for 8 hours, and was then filtrated through a pad of Celite. The Celite pad was washed with $MeOH/CHCl_3$ (1:1, 6 mL), $MeOH/CHCl_3$ (2:1, 6 mL), $MeOH/CHCl_3$ (3:1, 4 mL), MeOH (3 mL), $CHCl_3$ (3 mL) successively. The combined filtrate was concentrated to afford a solid (21 mg). ^{1}H NMR analysis of the solid indicated the complete cleavage of benzyl groups.

To the suspension of the above solid (21 mg) in CH_3OH/ CH_2Cl_2 (2/1, 1 mL) was added CH_3ONa (15 mg, excess). The suspension was stirred vigorously at 40 °C for 12 hours. The resulting white

slurry was centrifuged at 5000 rpm for 4 minutes, and the supernatant was removed. The white solid thus obtained was further washed multiple times with MeOH to remove CH_3ONa completely. 64-Mer **9F** was obtained as a white powder after lyophilization (12 mg, 51% over four steps).

^{1}H NMR (600 MHz, D_2O, 40 °C, δ_{HDO} = 4.60 ppm) δ 4.76 (s, 30H), 4.40 (d, J = 7.7 Hz, 31H), 4.09 (dd, J = 12.0, 5.3 Hz, 36H), 4.03–3.92 (m, 72H), 3.86–3.81 (m, 34H), 3.82–3.75 (m, 33H), 3.74–3.68 (m, 74H), 3.59 (t, J = 9.1 Hz, 36H), 3.52–3.48 (m, 40H), 3.39–3.32 (m, 35H), 3.33–3.28 (m, 38H); ^{13}C NMR (151 MHz, D_2O, 40 °C, δ_{DSS} = 0.0 ppm) δ 103.6, 98.6, 76.7, 76.5, 75.5, 74.2, 73.3, 71.8, 70.8, 63.3, 60.7; MALDI FT-ICR MS calcd for $C_{359}H_{584}O_{290}$ $[M + K]^+$ 9558.084, found 9558.223.

2.6 Spectral comparison of synthetic 16-mer 7F with previously reported xylomannans

Table S1. Comparison of the ^{13}C NMR data of 16-mer **7F** with those of the 8-mer synthesized by Crich et al.[4] and the natural xylomannan reported by Walters et al.[5]

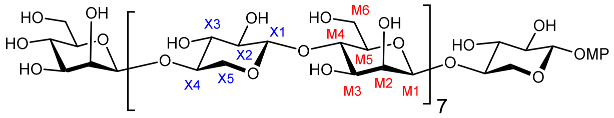

7F

Position	X1	X2	X3	X4	X5	M1	M2	M3	M4	M5	M6
Crich' report (ppm)[4]	103.6	73.3	74.2	76.5	63.2	98.6	70.8	71.8	76.7	75.5	60.7
16-mer **7F** (ppm)	103.3	73.0	73.9	76.2	63.0	98.3	70.5	71.5	76.4	75.2	60.4
Walters' report (ppm)[5]	101.7	72.8	73.8	76.6	63.0	100.2	70.1	71.6	76.6	75.1	60.6
Discrepancy (ppm)	**1.6**	0.2	0.1	-0.4	0.0	**-1.9**	0.4	-0.1	-0.2	0.1	-0.2

Notes: The ^{13}C NMR chemical shifts of the internal xylose and mannose residues for the synthetic 16-mer **7F** are nearly identical to those for the 8-mer prepared by Crich and co-workers, if the resonance of anomeric carbon of internal xyloside residue was calibrated to 103.6 ppm.

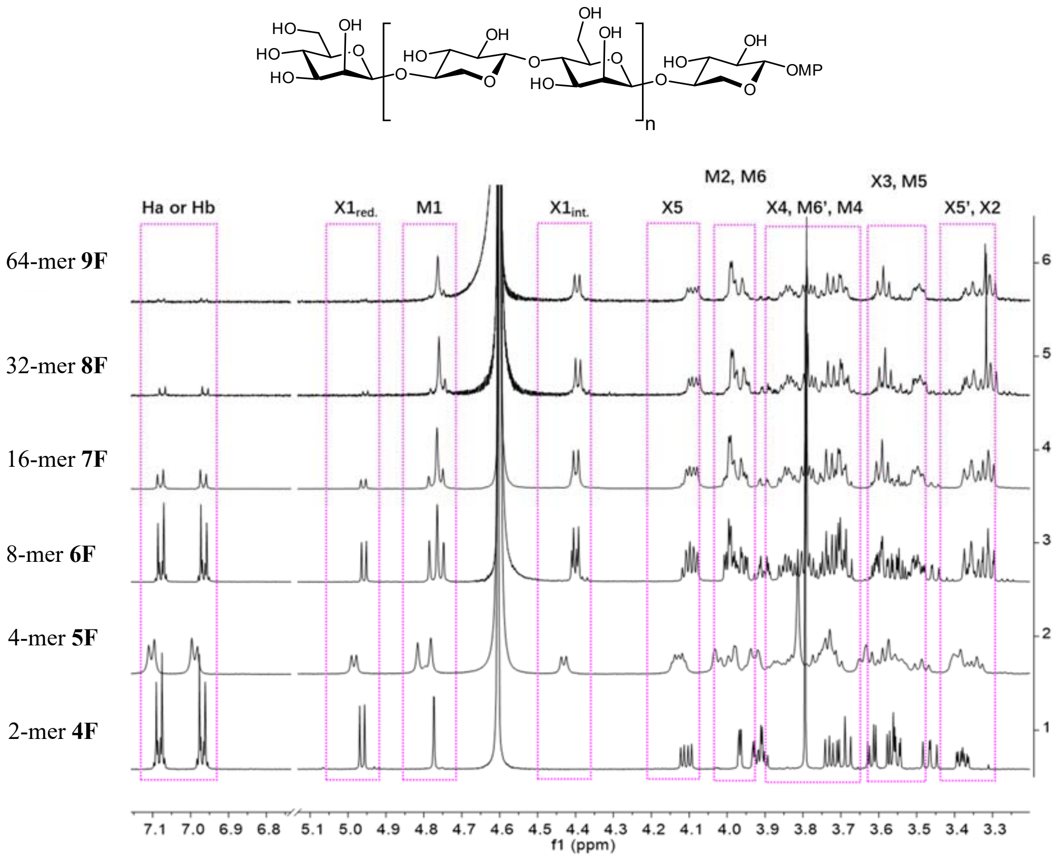

Figure S13. The overlaid ^{1}H NMR spectra of the [→4)-*β*-D-Man*p*-(1→4)-*β*-D-Xyl*p*-(1→]$_n$OMP glycans. Resonance peaks of the *p*-methoxyphenyl residue as well as the external mannopyranoside and xylopyranoside residues are merging into the baselines as the elongation of glycans.

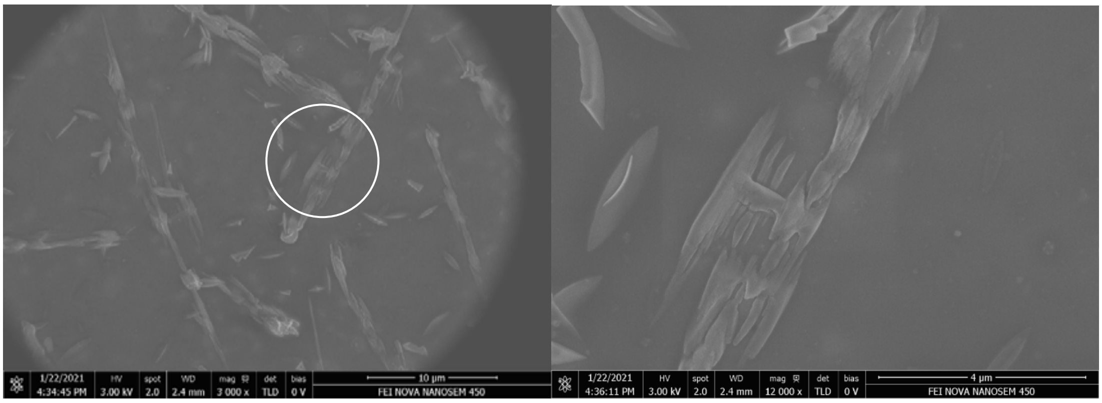

Figure S14. The fibrous aggregate of 16-mer **7F** observed by scanning electron microscopy. The sample was acquired by the solution-drying method.

3. Synthesis of [→3)-β-D-Man*p*-(1→4)-β-D-Xyl*p*-(1→] xylomannan glycan 13F

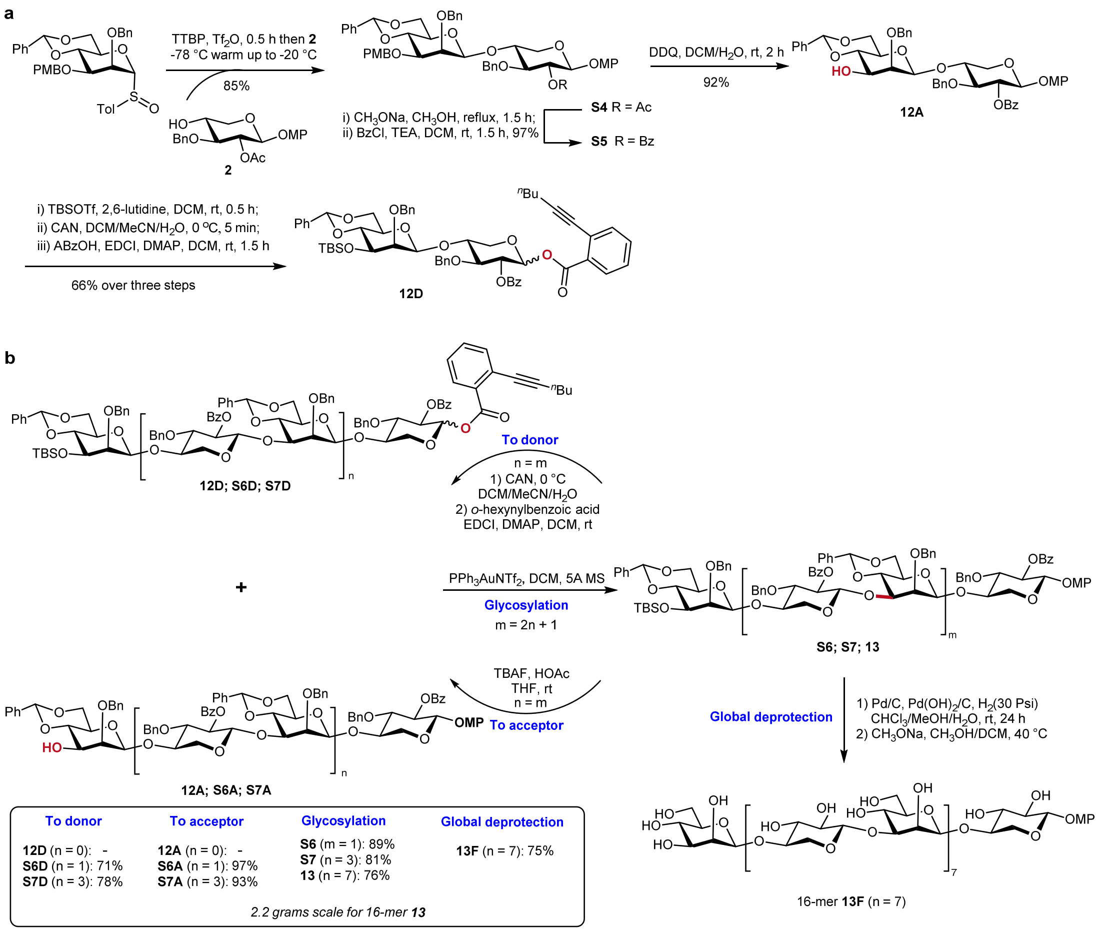

Figure S15. Synthesis of 16-mer **13F**. **a**, Preparation of disaccharide building blocks **12A** and **12D**. **b**, Preparation of 16-mer **13** via iterative exponential glycan growth (IEGG) strategy and the final deprotection to afford free **13F**.

3.1 Preparation of disaccharide building blocks 12A and 12D

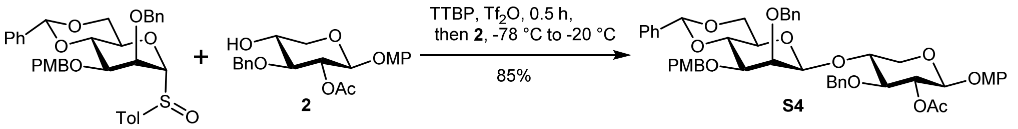

Disaccharide **S4** was prepared via the glycosylation of 4,6-*O*-benzylidene-3-*O*-*p*-methoxybenzyl-2-*O*-benzyl-1-*p*-methylphenylsulfinyl-α-D-mannopyranoside[6] with xylopyranoside **2**. The synthetic procedure was similar to that for the synthesis of disaccharide **3**.

A white solid: $[\alpha]_D^{25}$ = -70.1 (*c* 1.0, $CHCl_3$); ^1H NMR (600 MHz, $CDCl_3$) δ 7.51–7.43 (m, 4H), 7.40–7.24 (m, 11H), 7.23–7.18 (m, 2H), 6.99–6.93 (m, 2H), 6.86–6.79 (m, 4H), 5.58 (s, 1H), 5.14 (dd, *J* = 7.0, 5.4 Hz, 1H), 5.05 (d, *J* = 5.4 Hz, 1H), 4.92 (s, 2H), 4.87 (d, *J* = 11.8 Hz, 1H), 4.72 (d, *J* = 11.8 Hz, 1H), 4.65 (d, *J* = 12.0 Hz, 1H), 4.56 (d, *J* = 1.0 Hz, 1H), 4.55 (d, *J* = 12.0 Hz, 1H), 4.20 (dd, *J* = 10.4, 4.8 Hz, 1H), 4.15 (t, *J* = 9.6 Hz, 1H), 4.11 (dd, *J* = 12.2, 4.0 Hz, 1H), 3.95 (td, *J* = 6.6, 4.0 Hz, 1H), 3.86 (dd, *J* = 3.2, 1.0 Hz, 1H), 3.81–3.73 (m, 8H), 3.56 (dd, *J* = 9.9, 3.2 Hz, 1H), 3.44

(dd, J = 12.2, 6.8 Hz, 1H), 3.24 (ddd, J = 10.0, 9.3, 4.9 Hz, 1H), 1.99 (s, 3H); ^{13}C NMR (151 MHz, $CDCl_3$) δ 169.7, 159.2, 155.3, 150.9, 138.5, 138.4, 137.6, 130.4, 129.2, 128.9, 128.4, 128.3, 128.2, 127.7, 127.6, 126.1, 118.4, 114.6, 113.7, 101.4, 100.9, 99.6, 78.6, 77.4, 77.2, 77.0, 76.8, 76.6, 75.5, 74.8, 73.2, 72.2, 70.3, 68.6, 67.7, 61.5, 55.7, 55.3, 21.0; HRMS (ESI) calcd for $C_{49}H_{52}O_{13}Na$ $[M + Na]^+$ 871.3306, found 871.3294. The anomeric configuration was verified by ^1H-^1H 2D NOESY and coupled ^1H-^{13}C HSQC experiments.

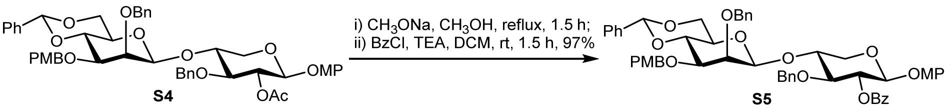

A similar synthetic procedure as that for the preparation of compound **4** was used to give disaccharide **S5** as a white solid. $[\alpha]_D^{25}$ = -53.0 (c 1.0, $CHCl_3$); ^1H NMR (500 MHz, $CDCl_3$) δ 8.08–8.02 (m, 2H), 7.56–7.49 (m, 1H), 7.49–7.35 (m, 5H), 7.38–7.33 (m, 3H), 7.29 (s, 1H), 7.24–7.16 (m, 4H), 7.00–6.93 (m, 2H), 6.87–6.80 (m, 2H), 6.83–6.76 (m, 2H), 5.47 (s, 1H), 5.40 (dd, J = 6.4, 5.1 Hz, 1H), 5.23 (d, J = 5.1 Hz, 1H), 4.90–4.81 (m, 3H), 4.79 (d, J = 11.8 Hz, 1H), 4.65 (d, J = 12.0 Hz, 1H), 4.55 (d, J = 12.0 Hz, 1H), 4.55 (d, J = 1.0 Hz, 1H), 4.19 (dd, J = 12.2, 3.7 Hz, 1H), 4.11 (dd, J = 10.5, 4.8 Hz, 1H), 4.04 (t, J = 9.6 Hz, 1H), 4.01–3.91 (m, 2H), 3.88–3.84 (m, 1H), 3.81 (s, 3H), 3.76 (s, 3H), 3.58–3.52 (m, 1H), 3.56–3.46 (m, 2H), 3.18 (td, J = 9.7, 4.8 Hz, 1H); ^{13}C NMR (126 MHz, $CDCl_3$) δ 165.5, 159.2, 155.3, 150.9, 138.4, 138.1, 137.5, 133.1, 130.3, 130.1, 129.8, 129.2, 128.9, 128.4, 128.3, 128.2, 128.1, 127.9, 127.6, 127.5, 126.0, 118.6, 114.5, 113.7, 101.3, 101.2, 99.6, 78.4, 77.3, 77.0, 76.8, 75.9, 75.0, 73.1, 72.1, 70.6, 68.4, 67.5, 61.5, 55.6, 55.3; HRMS (ESI) calcd for $C_{54}H_{54}O_{13}Na$ $[M + Na]^+$ 933.3462, found 933.3448.

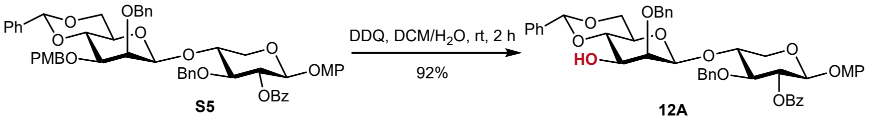

To a solution of compound **S5** (15.6 g, 17.1 mmol) in mixed solvent CH_2Cl_2/H_2O (150/15, 165 mL) was added 2,3-dichloro-5,6-dicyano-p-benzoquinone (DDQ) (5.06 g, 22.2 mmol). The resulting mixture was stirred vigorously at room temperature for 2 hours. The reaction mixture was diluted with CH_2Cl_2 (200 mL), and washed with saturated aqueous $NaHCO_3$ (300 mL) carefully. The organic layer was dried over anhydrous Na_2SO_4, concentrated *in vacuo*, and purified by silica gel chromatography (EtOAc/CH_2Cl_2/petroleum ether, 1:1:3, R_f = 0.2) to give **12A** as a white solid (12.4 g, 92%). $[\alpha]_D^{25}$ = -68.1 (c 1.0, $CHCl_3$); ^1H NMR (600 MHz, $CDCl_3$) δ 8.07–8.02 (m, 2H), 7.51 (tt, J = 7.4, 1.3 Hz, 1H), 7.48–7.42 (m, 2H), 7.38–7.30 (m, 7H), 7.33–7.26 (m, 3H), 7.28 (d, J = 2.0 Hz, 1H), 7.27 (s, 1H), 7.21 (dp, J = 4.8, 2.0 Hz, 3H), 7.01–6.95 (m, 2H), 6.83–6.77 (m, 2H), 5.42 (dd, J = 6.2, 4.8 Hz, 1H), 5.37 (s, 1H), 5.26 (d, J = 4.8 Hz, 1H), 5.01 (d, J = 11.5 Hz, 1H), 4.86 (d, J = 11.7 Hz, 1H), 4.82 (d, J = 11.7 Hz, 1H), 4.64 (d, J = 1.1 Hz, 1H), 4.62 (d, J = 11.5 Hz, 1H), 4.26 (dd, J = 12.3, 3.6 Hz, 1H), 4.12 (dd, J = 10.5, 4.9 Hz, 1H), 4.02 (td, J = 6.0, 3.7 Hz, 1H), 3.97 (t, J = 6.0 Hz, 1H), 3.88 (dd, J = 3.6, 1.0 Hz, 1H), 3.74 (s, 3H), 3.78–3.70 (m, 1H), 3.66 (t, J = 9.5 Hz, 1H), 3.61 (dd, J = 12.3, 6.0 Hz, 1H), 3.43 (t, J = 10.3 Hz, 1H), 3.18 (td, J = 9.7, 4.9 Hz, 1H), 2.44 (d, J = 8.7 Hz, 1H); ^{13}C NMR (151 MHz, $CDCl_3$) δ 165.5, 155.3, 150.9, 138.09, 138.08, 138.01, 137.2, 133.1, 130.0, 129.8, 129.1, 128.5, 128.3, 128.23, 128.20, 128.1, 127.9, 127.8, 127.6, 126.2, 118.5, 114.5, 101.9, 101.0, 99.5, 79.0, 78.9, 77.2, 77.0, 76.8, 76.3, 75.82, 75.76, 73.0, 70.7, 70.4, 68.3,

67.0, 61.1, 55.6; HRMS (ESI) calcd for $C_{46}H_{46}O_{12}Na$ $[M + Na]^+$ 813.2887, found 813.2877.

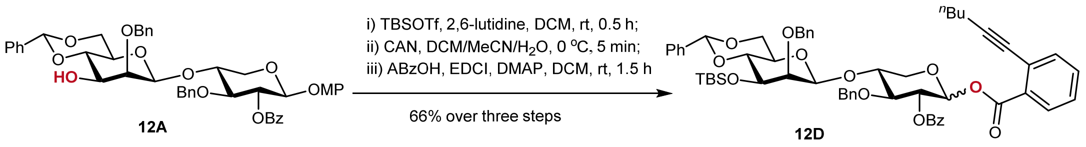

A similar procedure as that for the preparation of compound **4D** was used to provide **12D** as a white foam: α/β = 0.14:1; ^{1}H NMR (600 MHz, $CDCl_3$) δ 8.09–8.06 (m, 0.15H), 8.05–8.01 (m, 0.25H), 7.97–7.92 (m, 1.57H), 7.89 (dd, J = 7.9, 1.4 Hz, 0.84H), 7.72 (dd, J = 8.0, 1.4 Hz, 0.07H), 7.65–7.60 (m, 0.08H), 7.59–7.11 (m, 22H), 6.59 (d, J = 3.7 Hz, 0.78H), 6.57 (d, J = 3.5 Hz, 0.07H), 6.27 (d, J = 4.1 Hz, 0.12H), 5.53 (s, 0.88H), 5.41 (s, 0.13H), 5.40–5.37 (m, 0.12H), 5.35 (dd, J = 9.2, 3.7 Hz, 0.86H), 5.01 (d, J = 11.3 Hz, 0.88H), 4.95 (d, J = 11.5 Hz, 0.88H), 4.85–4.78 (m, 1.15H), 4.77–4.69 (m, 2.14H), 4.27 (dd, J = 12.6, 2.3 Hz, 0.11H), 4.24–4.08 (m, 2.75H), 4.04 (dd, J = 4.2, 2.0 Hz, 0.25H), 3.98 (t, J = 9.4 Hz, 0.89H), 3.91–3.70 (m, 4.89H), 3.46 (t, J = 10.3 Hz, 0.12H), 3.29 (td, J = 9.7, 4.9 Hz, 0.89H), 3.20 (q, J = 5.8, 4.9 Hz, 0.13H), 2.53–2.31 (m, 2H), 1.65–1.37 (m, 4H), 1.04–0.80 (m, 12H), 0.10–0.07 (m, 3H), 0.04–0.02 (m, 3H); ^{13}C NMR (151 MHz, $CDCl_3$) δ 165.5, 164.8, 164.7, 164.1, 163.8, 138.73, 138.68, 138.4, 137.9, 137.5, 134.8, 134.52, 134.48, 133.6, 133.18, 133.16, 132.1, 131.8, 130.8, 130.7, 130.6, 130.3, 130.2, 130.13, 130.08, 129.9, 129.8, 129.6, 129.5, 129.4, 128.9, 128.8, 128.6, 128.34, 128.26, 128.21, 128.08, 128.06, 128.02, 127.99, 127.93, 127.85, 127.71, 127.6, 127.43, 127.36, 127.2, 126.9, 126.2, 125.6, 125.3, 125.2, 101.9, 101.8, 100.4, 100.20, 100.18, 97.1, 97.0, 96.7, 92.5, 90.7, 80.4, 80.1, 79.9, 79.2, 79.08, 79.05, 78.8, 78.7, 78.6, 77.6, 77.2, 77.0, 76.8, 75.94, 75.91, 75.8, 75.5, 75.2, 74.7, 74.6, 74.2, 73.29, 73.25, 73.1, 73.0, 71.4, 68.8, 68.6, 68.4, 67.7, 67.6, 62.0, 61.1, 30.7, 30.7, 30.6, 25.8, 22.09, 22.07, 22.0, 19.6, 19.5, 18.29, 18.28, 13.68, 13.66, 13.64, -4.35, -4.37, -4.8, -5.0; HRMS (ESI) calcd for $C_{58}H_{66}O_{12}Na$ $[M + Na]^+$ 1005.4221, found 1005.4219.

3.2 Synthesis of fully protected 16-mer glycan 13

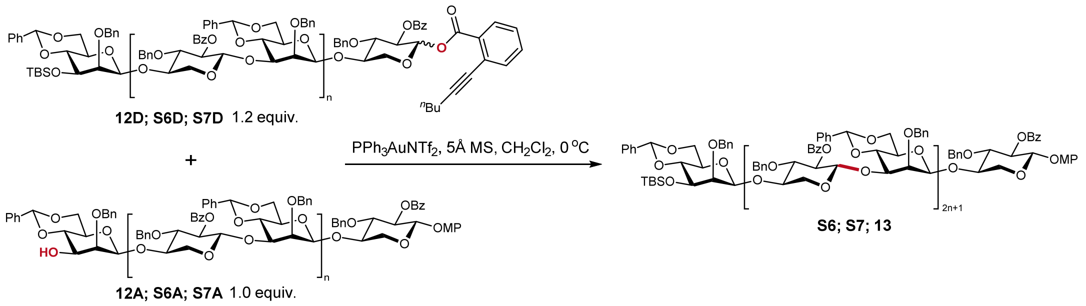

n	Substrates	PPh_3AuNTf_2 (eq.)	DCM/5Å MS	*c*	time	Method for purification	Product
0	**12D** (5.90 g) + **12A** (4.61 g)	431 mg (0.10)	58 mL/10 g	100 mM	1 h	EA/DCM/PE = 1:1:3.5, R_f = 0.6	**S6**, 8.1 g, 89%
1	**S6D** (3.46 g) + **S6A** (2.78 g)	282 mg (0.20)	32 mL/3.2 g	60 mM	2.5 h	EA/DCM/PE = 1:1:3, R_f = 0.6	**S7**, 4.5 g, 81%
3	**S7D** (1.86 g) + **S7A** (1.45 g)	77 mg (0.20)	15 mL/3.0 g	35 mM	14 h	EA/DCM/PE = 1:1:3, R_f = 0.4-0.5 [a]	**13**, 2.2 g, 76%

[a] TLC exhibited an elongated spot.

Figure S16. Preparation of the [→3)-*β*-D-Man*p*-(1→4)-*β*-D-Xyl*p*-(1→]$_n$OMP glycans via the gold (I)-catalyzed [2^n+2^n] glycosylation.

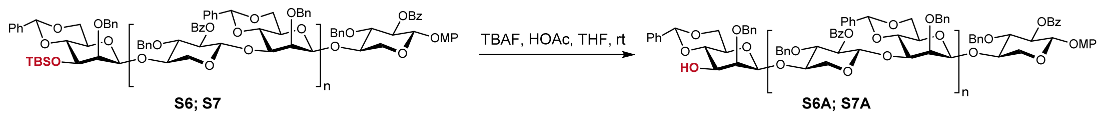

n	Substrates	TBAF (eq.)[a]; HOAc (eq.); THF	*c*	time	Method for purification	Product
1	**S6** (3.28 g)	20.8 mL (10.0); 238 μL (2.0); 21 mL	100 mM	1 h	EA/DCM/PE = 1:1:3, R_f = 0.20	**S6A**, 2.95 g, 97%
3	**S7** (1.74 g)	5.99 mL (10.0); 68.4 μL (2.0); 6 mL	100 mM	1.5 h	EA/DCM/PE = 1:1:2, R_f = 0.50	**S7A**, 1.55 g, 93%

[a] 1.0 M solution in THF.

Figure S17. Removal of the TBS group at the non-reducing end for the preparation of the [→3)-*β*-D-Man*p*-(1→4)-*β*-D-Xyl*p*-(1→]$_n$OMP acceptors.

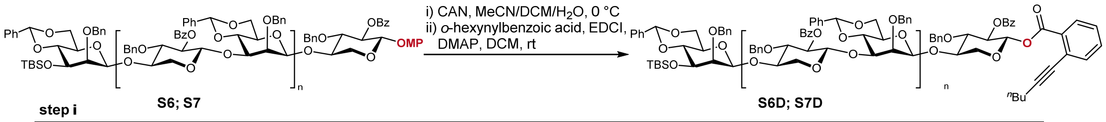

step i

n	Substrates	CAN (eq.); MeCN/DCM/H_2O	*c*	time	Method for purification	Product
1	**S6** (4.77 g)	5.00 (3.0); 50/25/5 mL	40 mM	5 min	EA/DCM/PE = 1:1:2, R_f = 0.4-0.6	used directly
3	**S7** (2.67 g)	2.67 g (3.0); 50/25/5 mL	12 mM	5 min	EA/DCM/PE = 1:1:2, R_f = 0.25-0.45	used directly

step ii

n	ABzOH (eq.); EDCI (eq.); DAMP (eq.); DCM	*c*	Time	Method for purification	Product (over 2 steps)
1	921 mg (1.5); 1.45 g (2.5); 927 mg (2.5), 30 mL	101 mM	2 h	EA/DCM/PE = 1:1:4, R_f = 0.5	**S6D**, 3.53 g, 77%
3	279 mg (1.5); 439 mg (2.5); 280 mg (2.5), 30 mL	31 mM	7 h	EA/DCM/PE = 1:1:3, R_f = 0.70-0.80	**S7D**, 2.13 g, 78%

Figure S18. Removal of the anomeric MP and subsequent condensation with *o*-hexynylbenzoic acid for the preparation of the [→3)-*β*-D-Man*p*-(1→4)-*β*-D-Xyl*p*-(1→]$_n$OMP donors.

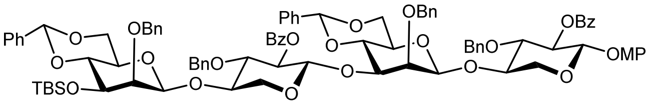

S6

A white foam: $[\alpha]_D^{25}$ = -83.5 (*c* 1.0, $CHCl_3$); ^{1}H NMR (600 MHz, $CDCl_3$) *δ* 8.05–8.00 (m, 4H), 7.52–7.46 (m, 2H), 7.49–7.41 (m, 4H), 7.39–7.34 (m, 2H), 7.36–7.28 (m, 10H), 7.30–7.24 (m, 3H), 7.27–7.24 (m, 1H), 7.24 (s, 2H), 7.24–7.22 (m, 1H), 7.24–7.21 (m, 1H), 7.23–7.18 (m, 1H), 7.20–7.13 (m, 8H), 7.12–7.06 (m, 1H), 6.98–6.92 (m, 2H), 6.82–6.76 (m, 2H), 5.44 (s, 1H), 5.41–5.37 (m, 1H), 5.37 (s, 1H), 5.18 (d, *J* = 5.2 Hz, 1H), 5.15–5.11 (m, 1H), 4.85 (d, *J* = 11.8 Hz, 1H), 4.81 (d, *J* = 3.2 Hz, 1H), 4.78 (s, 1H), 4.76 (s, 2H), 4.77–4.69 (m, 4H), 4.52 (d, *J* = 15.0 Hz, 2H), 4.29 (dd, *J* = 12.5, 3.1 Hz, 1H), 4.16–4.07 (m, 2H), 4.07–3.99 (m, 2H), 3.98–3.88 (m, 4H), 3.87 (d, *J* = 3.1 Hz, 1H), 3.83 (t, *J* = 4.1 Hz, 1H), 3.83–3.73 (m, 5H), 3.71–3.67 (m, 1H), 3.52 (t, *J* = 10.3 Hz, 1H), 3.45 (dd, *J* = 12.2, 6.6 Hz, 1H), 3.44–3.34 (m, 2H), 3.22 (td, *J* = 9.6, 4.8 Hz, 1H), 3.12–3.05 (m, 1H), 0.87 (s, 9H), 0.06 (s, 3H), 0.01 (s, 3H); ^{13}C NMR (151 MHz, $CDCl_3$) *δ* 165.6, 165.5, 155.3, 150.9, 138.8, 138.3, 138.21, 138.15, 137.6, 137.5, 133.09, 133.07, 130.06, 130.0, 129.9, 128.8, 128.7, 128.32, 128.27, 128.25, 128.16, 128.11, 128.05, 128.02, 127.98, 127.86, 127.77, 127.53, 127.46, 127.3, 126.2, 126.0, 118.6, 114.5, 101.7, 101.2, 100.9, 100.8, 99.8, 96.9, 80.3, 78.6, 77.2, 77.0, 76.8, 75.9, 75.7, 75.6, 75.34, 75.29, 75.1, 74.8, 73.2, 72.9, 72.4, 70.7, 69.9, 68.41, 68.37, 67.8, 67.5, 61.4, 59.7, 55.6, 25.8, 18.3, -4.4, -4.8; HRMS (ESI) calcd for $C_{91}H_{98}O_{22}Na$ $[M + Na]^+$ 1593.6217, found 1593.6205.

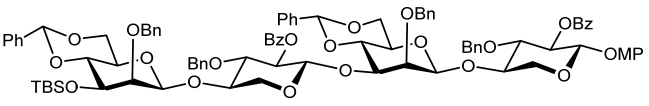

S6A

A white foam: $[\alpha]_D^{25}$ = -107.8 (*c* 1.0, $CHCl_3$); ^{1}H NMR (600 MHz, $CDCl_3$) δ 8.08–8.01 (m, 4H), 7.54–7.46 (m, 2H), 7.49–7.40 (m, 4H), 7.38–7.25 (m, 19H), 7.27–7.19 (m, 5H), 7.21–7.17 (m, 2H), 7.19–7.13 (m, 3H), 7.11–7.05 (m, 1H), 6.98–6.93 (m, 2H), 6.82–6.76 (m, 2H), 5.44 (s, 1H), 5.39 (dd, *J* = 6.6, 5.2 Hz, 1H), 5.30 (s, 1H), 5.19 (d, *J* = 5.2 Hz, 1H), 5.12 (dd, *J* = 4.4, 2.8 Hz, 1H), 4.96 (d, *J* = 11.4 Hz, 1H), 4.86 (d, *J* = 11.8 Hz, 1H), 4.83–4.73 (m, 5H), 4.71 (d, *J* = 11.8 Hz, 1H), 4.57–4.52 (m, 2H), 4.50 (d, *J* = 1.0 Hz, 1H), 4.35 (dd, *J* = 12.5, 2.9 Hz, 1H), 4.17–4.10 (m, 2H), 4.07–4.00 (m, 2H), 4.00–3.94 (m, 2H), 3.94–3.88 (m, 3H), 3.82–3.77 (m, 2H), 3.75 (s, 3H), 3.67 (td, *J* = 9.5, 3.7 Hz, 1H), 3.55 (d, *J* = 9.3 Hz, 1H), 3.52 (d, *J* = 10.3 Hz, 1H), 3.48 (dd, *J* = 12.3, 6.5 Hz, 1H), 3.43 (dd, *J* = 12.4, 4.1 Hz, 1H), 3.28 (t, *J* = 10.3 Hz, 1H), 3.23 (td, *J* = 9.7, 4.8 Hz, 1H), 3.10–3.03 (m, 1H), 2.38 (d, *J* = 9.1 Hz, 1H), -3.88 (s, 1H); ^{13}C NMR (151 MHz, $CDCl_3$) δ 165.6, 165.5, 155.3, 150.9, 138.23, 138.18, 138.15, 138.0, 137.6, 137.1, 133.2, 133.1, 130.1, 130.0, 129.9, 129.8, 129.1, 128.8, 128.4, 128.34, 128.27, 128.26, 128.23, 128.16, 128.08, 128.05, 127.91, 127.86, 127.80, 127.58, 127.57, 127.55, 126.2, 126.0, 118.6, 114.5, 101.9, 101.5, 101.3, 100.9, 99.7, 96.4, 79.0, 78.8, 77.2, 77.0, 76.8, 76.7, 75.9, 75.8, 75.54, 75.47, 75.3, 75.0, 74.2, 73.2, 72.3, 70.7, 70.6, 69.6, 68.4, 68.3, 67.8, 66.8, 61.4, 59.6, 55.6; HRMS (ESI) calcd for $C_{85}H_{84}O_{22}Na$ $[M + Na]^+$ 1479.5352, found 1479.5339.

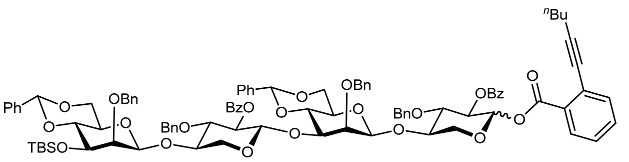

S6D

A white foam (α/β = 1.5:1): ^{1}H NMR (600 MHz, $CDCl_3$) δ 8.10–7.98 (m, 2.86H), 7.96–7.92 (m, 1.16H), 7.89 (td, *J* = 7.9, 1.4 Hz, 0.95H), 7.56 (dd, *J* = 7.8, 1.3 Hz, 0.55H), 7.54–7.12 (m, 40H), 7.11–7.02 (m, 1H), 6.56 (d, *J* = 3.7 Hz, 0.58H), 6.24 (d, *J* = 4.2 Hz, 0.38H), 5.55 (s, 0.59H), 5.40 (s, 0.38H), 5.40–5.36 (m, 1.37H), 5.32 (dd, *J* = 9.2, 3.7 Hz, 0.61H), 5.16 (dd, *J* = 5.2, 3.6 Hz, 0.62H), 5.12 (dd, *J* = 4.8, 3.2 Hz, 0.38H), 4.99 (d, *J* = 11.3 Hz, 0.64H), 4.87–4.67 (m, 8.58H), 4.57 (dd, *J* = 4.0, 0.9 Hz, 0.94H), 4.52 (dd, *J* = 2.9, 1.0 Hz, 1.02H), 4.35–3.64 (m, 16.19H), 3.49–3.34 (m, 2.50H), 3.30 (td, *J* = 9.7, 4.8 Hz, 0.65H), 3.22 (td, *J* = 9.6, 4.8 Hz, 0.42H), 3.13–3.06 (m, 1H), 2.52–2.31 (m, 2H), 1.65–1.58 (m, 0.82H), 1.55–1.36 (m, 3.26H), 0.99–0.81 (m, 12.25H), 0.06 (d, *J* = 2.5 Hz, 3H), 0.01 (d, *J* = 1.7 Hz, 3H); ^{13}C NMR (151 MHz, $CDCl_3$) δ 165.53, 165.51, 165.44, 164.1, 163.8, 138.81, 138.78, 138.34, 138.32, 138.26, 138.15, 138.14, 137.9, 137.56, 137.53, 137.50, 134.8, 134.53, 134.48, 133.20, 133.16, 133.10, 132.2, 132.1, 130.7, 130.6, 130.2, 130.13, 130.10, 130.06, 130.02, 129.89, 129.86, 129.81, 129.79, 129.5, 129.4, 128.82, 128.76, 128.70, 128.60, 128.36, 128.34, 128.31, 128.29, 128.25, 128.22, 128.17, 128.11, 128.08, 128.05, 128.01, 127.98, 127.95, 127.8, 127.7, 127.6, 127.53, 127.47, 127.39, 127.31, 127.30, 127.15, 126.9, 126.2, 126.1, 126.0, 125.6, 125.4, 101.8, 101.7, 101.3, 101.2, 100.80, 100.75, 100.72, 100.5, 97.5, 97.09, 97.07, 96.8, 92.5, 90.6, 80.34, 80.29, 79.1, 79.0, 78.58, 78.56, 77.5, 77.2, 77.0, 76.8, 76.3, 76.2, 76.0, 75.8, 75.71, 75.65, 75.60, 75.58, 75.4, 75.23, 75.19, 75.1, 75.0, 74.8, 74.7, 74.6, 73.1, 73.0, 72.9, 72.5, 72.3, 71.3, 70.3, 69.8, 68.8, 68.5, 68.39, 68.37, 67.9, 67.8, 67.47, 67.45, 61.8, 61.3, 60.1, 59.6, 30.69,

30.66, 30.6, 25.8, 22.09, 22.07, 22.0, 19.58, 19.56, 19.52, 18.3, 13.7, 13.6, -4.4, -4.8; HRMS (ESI) calcd for $C_{97}H_{104}O_{22}Na$ [M + Na]$^+$ 1671.6686, found 1671.6681.

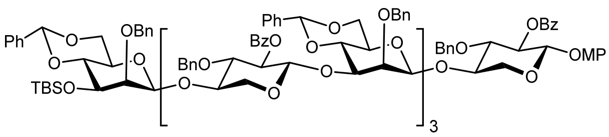

S7

A white foam: $[\alpha]_D^{25}$ = -110.9 (*c* 1.0, $CHCl_3$); ^{1}H NMR (500 MHz, $CDCl_3$) δ 8.08–7.93 (m, 8H), 7.51–7.40 (m, 12H), 7.40–7.01 (m, 60H), 6.98–6.91 (m, 2H), 6.81–6.76 (m, 2H), 5.44 (s, 1H), 5.40–5.34 (m, 4H), 5.18 (d, *J* = 5.2 Hz, 1H), 5.12–5.06 (m, 3H), 4.85 (d, *J* = 11.8 Hz, 1H), 4.82–4.66 (m, 19H), 4.54 (s, 1H), 4.50 (d, *J* = 1.0 Hz, 1H), 4.41–4.38 (m, 2H), 4.34–4.24 (m, 3H), 4.16–4.08 (m, 2H), 4.06–3.99 (m, 4H), 3.97–3.86 (m, 9H), 3.86–3.78 (m, 5H), 3.75 (s, 7H), 3.69–3.67 (m, 1H), 3.52 (t, *J* = 10.3 Hz, 1H), 3.48–3.39 (m, 2H), 3.39–3.29 (m, 5H), 3.25–3.18 (m, 1H), 3.14–3.03 (m, 3H), 0.87 (s, 9H), 0.05 (s, 3H), 0.01 (s, 3H); ^{13}C NMR (126 MHz, $CDCl_3$) δ 165.61, 165.58, 165.56, 165.4, 155.3, 150.9, 138.8, 138.30, 138.28, 138.26, 138.19, 138.16, 138.12, 137.52, 137.46, 133.10, 133.06, 130.10, 130.07, 130.01, 129.85, 129.82, 129.77, 128.82, 128.76, 128.7, 128.40, 128.38, 128.32, 128.26, 128.24, 128.21, 128.15, 128.12, 128.10, 128.04, 128.00, 127.96, 127.85, 127.80, 127.76, 127.53, 127.48, 127.46, 127.3, 126.1, 126.0, 118.5, 114.5, 101.7, 101.4, 101.3, 101.21, 101.17, 101.1, 100.83, 100.80, 99.72, 96.69, 96.4, 96.2, 80.3, 78.5, 77.3, 77.2, 77.0, 76.8, 76.6, 75.9, 75.56, 75.54, 75.47, 75.40, 75.34, 75.26, 75.20, 75.15, 75.0, 74.8, 74.7, 74.5, 74.3, 74.1, 73.2, 72.9, 72.4, 72.24, 72.21, 70.7, 69.8, 69.5, 68.4, 68.3, 67.8, 67.6, 67.4, 61.4, 59.8, 59.5, 59.3, 55.6, 25.8, 18.3, -4.4, -4.9; MALDI FT-ICR MS calcd for $C_{169}H_{174}O_{42}SiK$ [M + K]$^+$ 2942.088, found 2942.079.

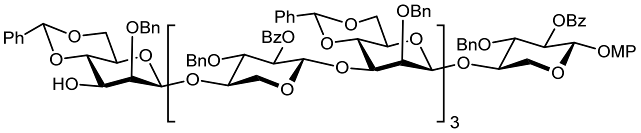

S7A

A white foam: $[\alpha]_D^{25}$ = -130.2 (*c* 1.0, $CHCl_3$); ^{1}H NMR (500 MHz, $CDCl_3$) δ 8.08–7.98 (m, 8H), 7.52–7.47 (m, 2H), 7.47–7.40 (m, 10H), 7.36–7.26 (m, 36H), 7.25–7.17 (m, 14H), 7.16–7.07 (m, 6H), 7.07–7.02 (m, 2H), 6.97–6.92 (m, 2H), 6.83–6.76 (m, 2H), 5.44 (s, 1H), 5.40–5.37 (m, 1H), 5.37–5.36 (m, 2H), 5.28 (s, 1H), 5.18 (d, *J* = 5.3 Hz, 1H), 5.14–5.06 (m, 3H), 4.96 (d, *J* = 11.4 Hz, 1H), 4.88–4.74 (m, 10H), 4.73–4.67 (m, 7H), 4.56–4.47 (m, 4H), 4.42–4.40 (m, 2H), 4.35 (dd, *J* = 12.6, 2.7 Hz, 1H), 4.31–4.24 (m, 2H), 4.16–4.08 (m, 2H), 4.06–3.98 (m, 5H), 3.98–3.89 (m, 7H), 3.88–3.80 (m, 6H), 3.78–3.70 (m, 6H), 3.70–3.64 (m, 1H), 3.54–3.49 (m, 2H), 3.48–3.41 (m, 2H), 3.40–3.30 (m, 4H), 3.28–3.18 (m, 2H), 3.14–3.01 (m, 3H), 2.37 (d, *J* = 9.2 Hz, 1H); ^{13}C NMR (126 MHz, $CDCl_3$) δ 165.61, 165.59, 165.4, 155.3, 150.9, 138.28, 138.26, 138.23, 138.17, 138.13, 138.0, 137.5, 137.1, 133.12, 133.08, 130.11, 130.08, 130.01, 129.90, 129.87, 129.83, 129.78, 129.1, 128.8, 128.43, 128.38, 128.32, 128.28, 128.24, 128.16, 128.13, 128.04, 128.01, 127.93, 127.85, 127.80, 127.58, 127.53, 127.49, 126.2, 126.0, 118.6, 114.5, 101.9, 101.5, 101.4, 101.3, 101.22, 101.19, 101.17, 100.8, 99.7, 96.7, 96.2, 95.9, 78.9, 78.8, 77.3, 77.0, 76.8, 75.9, 75.8, 75.6, 75.5, 75.4, 75.33, 75.26, 75.19, 75.16, 75.13, 74.7, 74.4, 74.1, 73.8, 73.2, 72.4, 72.24, 72.15, 70.7, 70.5, 69.8, 69.4, 69.3, 68.4, 68.3, 68.2, 67.8, 67.6, 66.8, 61.4, 59.8, 59.5, 59.2, 55.6; MALDI FT-ICR MS calcd for $C_{163}H_{160}O_{42}K$ [M + K]$^+$ 2828.002, found 2827.996.

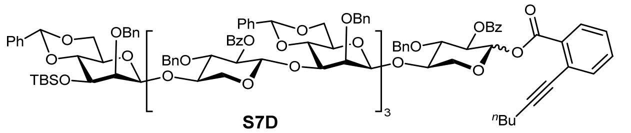

S7D

A white foam (α/β = 1.3:1): ^{1}H NMR (600 MHz, $CDCl_3$) δ 8.05–7.98 (m, 7H), 7.94–7.92 (m, 1H), 7.90–7.87 (m, 1H), 7.57–7.38 (m, 15H), 7.38–7.22 (m, 42H), 7.23–7.08 (m, 21H), 7.07–7.02 (m, 3H), 6.56 (d, J = 3.6 Hz, 0.55H), 6.23 (d, J = 4.2 Hz, 0.43H), 5.54 (s, 0.57H), 5.41–5.34 (m, 4H), 5.32 (dd, J = 9.0, 3.7 Hz, 0.57H), 5.17–5.07 (m, 3H), 4.99 (d, J = 11.3 Hz, 0.55H), 4.87–4.66 (m, 19H), 4.57 (d, J = 5.2 Hz, 1H), 4.50 (s, 1H), 4.42–4.38 (m, 2H), 4.32–3.98 (m, 10H), 3.97–3.88 (m, 7.44H), 3.87–3.78 (m, 5.58H), 3.78–3.66 (m, 8H), 3.50–3.25 (m, 7H), 3.21 (td, J = 9.5, 4.7 Hz, 0.71H), 3.15–3.03 (m, 3H), 2.47 (t, J = 7.2 Hz, 0.88H), 2.36 (t, J = 7.1 Hz, 1.12H), 1.65–1.58 (m, 0.91H), 1.54–1.37 (m, 3.26H), 0.97–0.83 (m, 12H), 0.05 (s, 3H), 0.01 (s, 3H); ^{13}C NMR (151 MHz, $CDCl_3$) δ 165.62, 165.57, 165.5, 165.4, 164.1, 163.8, 138.8, 138.33, 138.30, 138.25, 138.23, 138.20, 138.15, 138.13, 137.8, 137.56, 137.53, 137.49, 134.8, 134.5, 133.20, 133.16, 133.11, 133.06, 132.16, 132.13, 130.7, 130.6, 130.2, 130.10, 130.07, 130.05, 129.89, 129.86, 129.81, 129.79, 129.5, 129.4, 128.82, 128.76, 128.69, 128.39, 128.36, 128.28, 128.25, 128.22, 128.16, 128.13, 128.11, 128.08, 128.04, 128.01, 127.97, 127.95, 127.81, 127.77, 127.69, 127.63, 127.53, 127.49, 127.47, 127.4, 127.3, 127.1, 126.2, 126.04, 126.01, 125.6, 125.4, 101.7, 101.4, 101.34, 101.31, 101.24, 101.22, 101.20, 101.17, 100.8, 100.7, 100.5, 97.3, 97.09, 97.07, 96.6, 96.4, 96.34, 96.30, 92.5, 90.6, 80.3, 79.1, 79.0, 78.5, 77.2, 77.0, 76.8, 76.3, 75.9, 75.8, 75.7, 75.6, 75.51, 75.48, 75.43, 75.41, 75.37, 75.23, 75.21, 75.19, 75.1, 75.0, 74.83, 74.80, 74.76, 74.6, 74.5, 74.4, 74.2, 73.1, 72.9, 72.5, 72.4, 72.3, 72.2, 71.3, 70.3, 69.7, 69.6, 68.8, 68.5, 68.4, 67.9, 67.8, 67.7, 67.6, 67.4, 61.8, 61.3, 60.2, 59.7, 59.6, 59.4, 30.7, 30.6, 25.8, 22.1, 22.0, 19.58, 19.56, 18.3, 13.68, 13.66, 13.63, -4.4, -4.8; MALDI FT-ICR MS calcd for $C_{175}H_{180}O_{42}K$ $[M + K]^+$ 3020.135, found 3020.120.

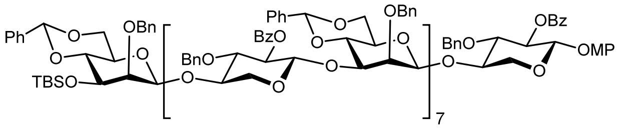

13

A white foam: $[\alpha]_D^{25}$ = -131.3 (c 1.0, $CHCl_3$); ^{1}H NMR (600 MHz, $CDCl_3$) δ 8.05–7.99 (m, 16H), 7.51–7.40 (m, 25H), 7.39–7.31 (m, 6H), 7.34–7.20 (m, 55H), 7.22–7.13 (m, 28H), 7.15–7.08 (m, 13H), 7.08–7.03 (m, 6H), 6.98–6.94 (m, 2H), 6.82–6.76 (m, 2H), 5.43 (s, 1H), 5.40–5.33 (m, 7H), 5.18 (d, J = 5.2 Hz, 1H), 5.13–5.07 (m, 7H), 4.85 (d, J = 11.7 Hz, 1H), 4.82–4.74 (m, 12H), 4.74 (s, 6H), 4.74–4.67 (m, 20H), 4.54 (s, 1H), 4.50 (s, 1H), 4.41–4.36 (m, 6H), 4.33–4.23 (m, 7H), 4.15–4.08 (m, 2H), 4.04–3.99 (m, 7H), 3.97–3.79 (m, 30H), 3.77–3.72 (m, 6H), 3.73–3.67 (m, 6H), 3.51 (t, J = 10.3 Hz, 1H), 3.47–3.39 (m, 2H), 3.38–3.27 (m, 13H), 3.22 (td, J = 9.6, 4.8 Hz, 1H), 3.12–3.04 (m, 7H), 0.87 (s, 9H), 0.05 (s, 3H), 0.01 (s, 3H); ^{13}C NMR (151 MHz, $CDCl_3$) δ 165.61, 165.58, 165.56, 165.4, 155.3, 150.9, 138.8, 138.32, 138.30, 138.28, 138.23, 138.20, 138.15, 137.6, 137.5, 133.1, 133.0, 130.11, 130.07, 130.01, 129.94, 129.88, 129.85, 129.81, 129.3, 128.81, 128.75, 128.69, 128.40, 128.37, 128.31, 128.26, 128.24, 128.21, 128.15, 128.12, 128.10, 128.03, 127.99, 127.96, 127.88, 127.85, 127.80, 127.76, 127.63, 127.52, 127.46, 127.28, 126.15, 126.05, 126.00, 118.6, 115.1, 114.5, 101.7, 101.5, 101.4, 101.3, 101.24, 101.19, 101.16, 100.8, 99.7, 96.7, 96.4, 96.3, 80.3, 78.5, 76.71, 76.66, 76.64, 75.9, 75.64, 75.58, 75.55, 75.52, 75.48, 75.44, 75.3, 75.2, 75.0, 74.82, 74.77, 74.7, 74.5, 74.4, 74.2, 73.2, 72.9, 72.4, 72.25, 72.23, 70.7, 69.8, 69.5, 68.4, 68.3, 67.8, 67.64,

67.61, 67.43, 61.41, 59.8, 59.61, 59.55, 59.4, 55.6, 25.8, -4.4, -4.8; MALDI FT-ICR MS calcd for $C_{325}H_{326}O_{82}SiK$ [M + K]$^+$ 5607.074, found 5607.084.

3.3 Preparation of free 16-mer 13F

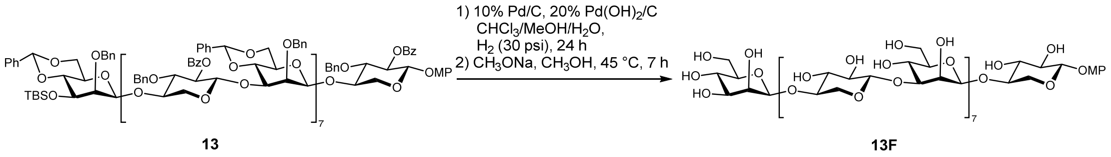

16-Mer **13** (60 mg) was dissolved in a mixture of MeOH/$CHCl_3$/H_2O (3/6/0.2, 9.2 mL) containing 10% Pd/C (90 mg, wetted with 55% H_2O) and 20% $Pd(OH)_2$/C (90 mg, wetted with 50% H_2O). The resulting mixture was stirred under H_2 atmosphere (30 Psi) at 30 °C for 24 hours, and was then filtrated through a pad of Celite. The Celite pad was washed with MeOH/$CHCl_3$ (1:1, 6 mL), MeOH/$CHCl_3$ (2:1, 6 mL), MeOH/$CHCl_3$ (3:1, 4 mL), MeOH (3 mL), and $CHCl_3$ (3 mL) successively. The combined filtrate was concentrated to afford a solid.

To the suspension of the above solid in CH_3OH/CH_2Cl_2 (2.5/1.5, 4 mL) was added CH_3ONa (25 mg, excess). The suspension was stirred vigorously at 45 °C for 7 hours. The resulting white slurry was centrifuged at 5000 rpm for 4 minutes. The supernatant was removed to give a white solid, which was washed multiple times with MeOH to remove the CH_3ONa completely. The white solid was purified by gel filtration (Sephadex G-25, H_2O) to give **13F** as a white powder after lyophilization (20 mg, 75% over two steps).

^{1}H NMR (600 MHz, pH = 7.5, D_2O, 40 °C, δ_{HDO} = 4.80 ppm) δ 7.27 (d, J = 8.6 Hz, 2H), 7.16 (d, J = 8.6 Hz, 2H), 5.15 (d, J = 7.7 Hz, 1H), 4.96 (s, 1H), 4.94 (s, 8H), 4.70 (d, J = 7.6 Hz, 8H), 4.33 (s, 8H), 4.27 (dd, J = 11.7, 5.2 Hz, 9H), 4.11 (d, J = 11.7 Hz, 11H), 4.08–4.03 (m, 8H), 3.98 (d, J = 8.4 Hz, 11H), 3.92 (dd, J = 12.2, 6.5 Hz, 9H), 3.84 (t, J = 9.7 Hz, 9H), 3.80 (t, J = 8.9 Hz, 8H), 3.60–3.55 (m, 20H), 3.53 (d, J = 10.9 Hz, 6H); ^{13}C NMR (151 MHz, pH = 7.5, D_2O, 40 °C, δ_{DSS} = 0.0 ppm) δ 157.6, 153.4, 121.2, 117.9, 104.5, 103.5, 101.1, 100.9, 82.7, 79.1, 78.9, 76.5, 75.6, 75.3, 73.5, 71.0, 69.5, 67.8, 65.6, 63.8, 58.6; MALDI FT-ICR MS calcd for $C_{95}H_{152}O_{74}K$ [M + K]$^+$ 2499.802, found 2499.794.

3.4 Spectral comparison of synthetic 16-mer 13F with the natural xylomannan

Table S2. ^{13}C NMR comparison between the synthetic 16-mer **13F** and the natural xylomannan reported by Walters et al.

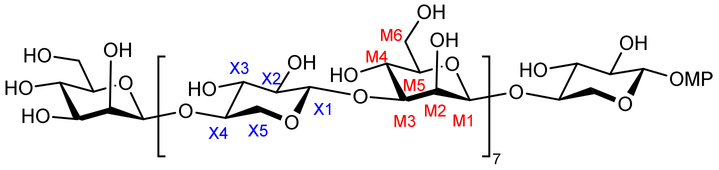

16-mer **13F**

Position	X1	X2	X3	X4	X5	M1	M2	M3	M4	M5	M6
16-mer **13F** (ppm)	101.4	73.2	74.4	77.0	63.5	98.8	68.9	80.6	65.7	76.8	61.7
Walters' report (ppm)[5]	101.7	72.8	73.8	76.6	63.0	100.2	70.1	71.6	76.6	75.1	60.6
Discrepancy (ppm)	-0.3	0.4	0.6	0.4	0.5	**-1.4**	**-1.2**	**9.0**	**-10.9**	**1.7**	**1.1**

4. Synthesis of [→4)-β-L-Manp-(1→4)-β-D-Xylp-(1→] xylomannan glycan 15F

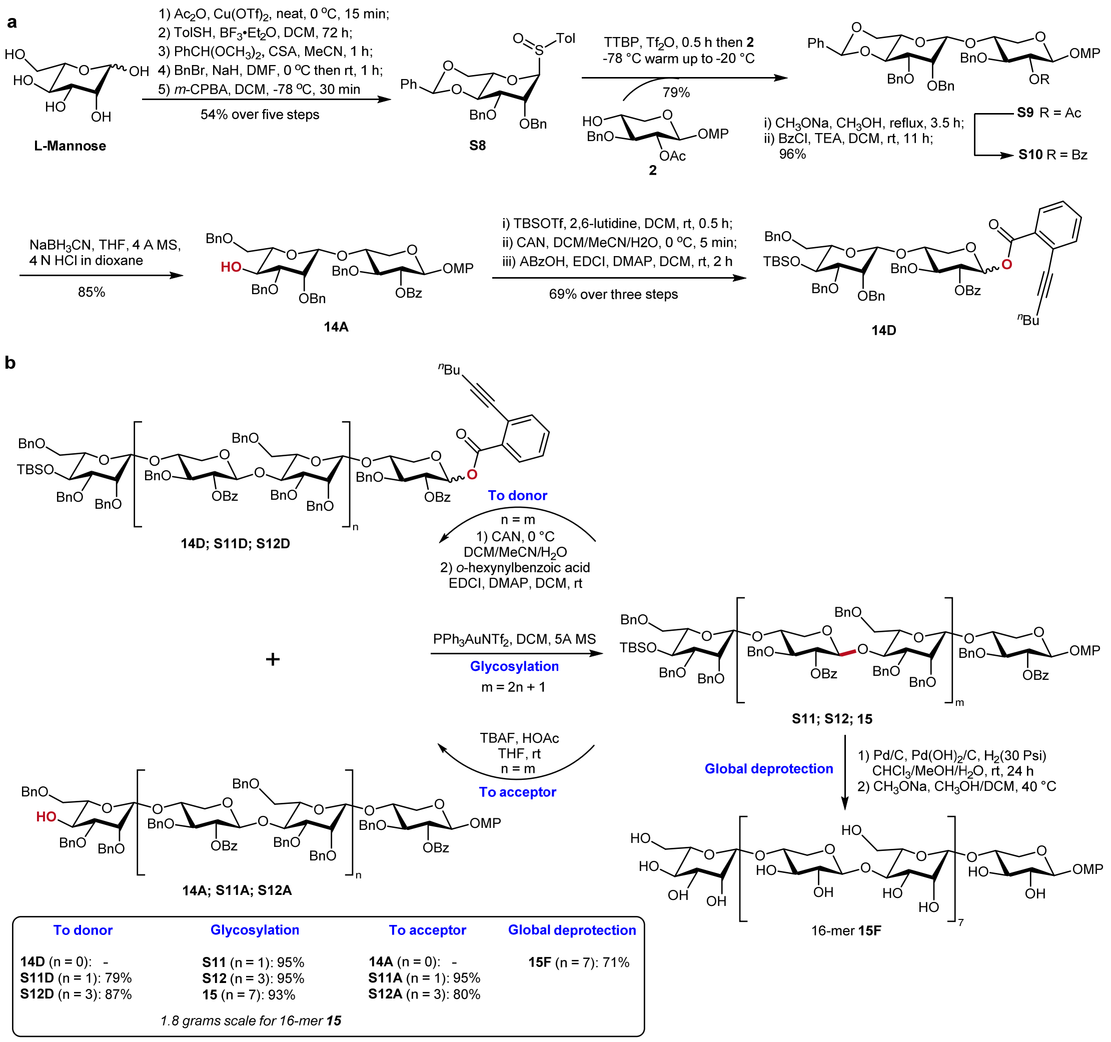

Figure S19. Synthesis of 16-mer **15F**. **a**, Preparation of disaccharide building blocks **14A** and **14D**. **b**, Preparation of 16-mer **15** via iterative exponential glycan growth (IEGG) strategy and the final deprotection to afford free **15F**.

4.1 Preparation of disaccharide building blocks 14A and 14B

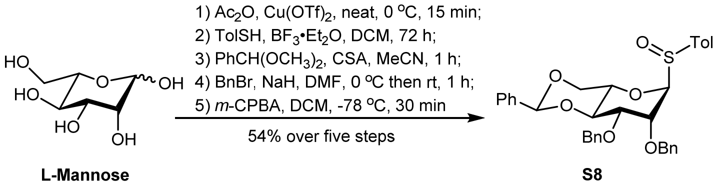

A similar procedure as that for the preparation of compound **1** was used to provide **S8** as a white solid: $[\alpha]_D^{20}$ = 64.0 (*c* 2.0, $CHCl_3$); ^1H NMR (400 MHz, $CDCl_3$) δ 7.50 (dt, J = 7.1, 2.4 Hz, 2H), 7.44–7.35 (m, 5H), 7.38–7.27 (m, 7H), 7.31–7.19 (m, 5H), 5.63 (s, 1H), 4.82 (d, J = 12.0 Hz, 1H),

4.66 (d, J = 12.1 Hz, 1H), 4.59 (s, 2H), 4.47 (d, J = 1.3 Hz, 1H), 4.40 (dd, J = 3.1, 1.4 Hz, 1H), 4.39–4.24 (m, 2H), 4.20 (dd, J = 10.3, 4.8 Hz, 1H), 4.07 (td, J = 9.2, 4.8 Hz, 1H), 3.74 (t, J = 10.1 Hz, 1H), 2.44 (s, 3H); HRMS (ESI) calcd for $C_{34}H_{34}O_6SNa$ $[M + Na]^+$ 593.1974, found 593.1966. The ^{1}H NMR spectroscopic data are identical to those of its enantiomer **1**.

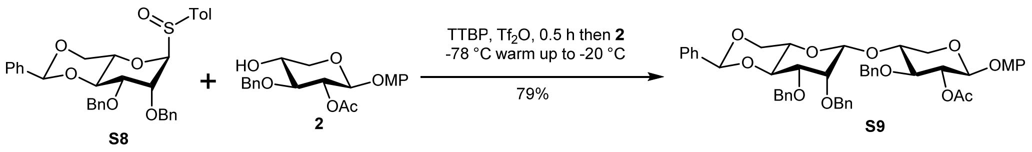

A similar procedure as that for the preparation of compound **3** was used to provide **S9** as a white solid: $[\alpha]_D^{20}$ = 14.7 (c 1.0, $CHCl_3$); ^{1}H NMR (600 MHz, $CDCl_3$) δ 7.56–7.51 (m, 2H), 7.50–7.46 (m, 2H), 7.44–7.25 (m, 12H), 7.22–7.18 (m, 2H), 7.01–6.95 (m, 2H), 6.89–6.83 (m, 2H), 5.63 (s, 1H), 5.20 (dd, J = 9.4, 7.6 Hz, 1H), 4.92 (d, J = 12.4 Hz, 1H), 4.89–4.85 (m, 2H), 4.74 (d, J = 12.3 Hz, 1H), 4.63 (d, J = 11.8 Hz, 1H), 4.59 (d, J = 12.1 Hz, 2H), 4.49 (d, J = 11.8 Hz, 1H), 4.31 (dd, J = 10.4, 4.9 Hz, 1H), 4.26 (dd, J = 11.9, 5.5 Hz, 1H), 4.19 (t, J = 9.6 Hz, 1H), 3.93 (t, J = 10.3 Hz, 1H), 3.88 (ddd, J = 10.0, 8.8, 5.5 Hz, 1H), 3.80 (s, 3H), 3.74 (dd, J = 3.0, 1.0 Hz, 1H), 3.59 (t, J = 9.1 Hz, 1H), 3.48 (dd, J = 9.9, 3.0 Hz, 1H), 3.42 (dd, J = 11.9, 10.0 Hz, 1H), 3.37–3.27 (m, 1H), 2.06 (s, 3H); ^{13}C NMR (151 MHz, $CDCl_3$) δ 169.5, 155.6, 151.1, 138.4, 138.3, 138.1, 137.5, 128.9, 128.7, 128.5, 128.4, 128.22, 128.19, 127.9, 127.74, 127.70, 127.6, 127.5, 126.1, 118.6, 114.6, 102.9, 101.5, 101.0, 82.0, 78.6, 78.3, 78.1, 77.3, 77.1, 76.9, 76.0, 74.9, 74.8, 72.9, 72.8, 68.5, 67.6, 65.1, 55.7, 20.9; HRMS (ESI) calcd for $C_{48}H_{50}O_{12}Na$ $[M + Na]^+$ 841.3200, found 841.3191.

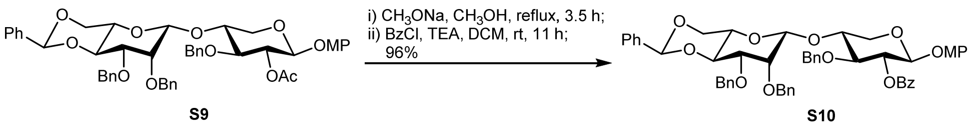

A similar procedure as that for the preparation of compound **4** was used to provide **S10** as a white solid: $[\alpha]_D^{20}$ = 41.4 (c 0.5, $CHCl_3$); ^{1}H NMR (600 MHz, $CDCl_3$) δ 8.08–8.01 (m, 2H), 7.61–7.56 (m, 1H), 7.50–7.47 (m, 2H), 7.47–7.43 (m, 4H), 7.41–7.26 (m, 12H), 7.15–7.10 (m, 3H), 7.09–7.04 (m, 2H), 6.93–6.89 (m, 2H), 6.78–6.74 (m, 2H), 5.59 (s, 1H), 5.43 (dd, J = 9.2, 7.5 Hz, 1H), 4.98 (d, J = 7.5 Hz, 1H), 4.88 (d, J = 12.3 Hz, 1H), 4.83 (d, J = 12.3 Hz, 1H), 4.67 (d, J = 12.3 Hz, 1H), 4.61 (d, J = 11.6 Hz, 1H), 4.58 (d, J = 1.0 Hz, 1H), 4.53 (d, J = 12.3 Hz, 1H), 4.43 (d, J = 11.6 Hz, 1H), 4.31–4.26 (m, 2H), 4.15 (t, J = 9.6 Hz, 1H), 3.96–3.91 (m, 1H), 3.90 (t, J = 10.3 Hz, 1H), 3.74 (s, 3H), 3.75–3.71 (m, 1H), 3.70 (dd, J = 3.1, 0.9 Hz, 1H), 3.46 (dd, J = 12.0, 9.9 Hz, 1H), 3.42 (dd, J = 9.9, 3.0 Hz, 1H), 3.28 (td, J = 9.8, 4.9 Hz, 1H); ^{13}C NMR (151 MHz, $CDCl_3$) δ 165.2, 155.6, 151.1, 138.4, 138.3, 137.8, 137.5, 133.3, 129.8, 129.7, 128.9, 128.6, 128.5, 128.4, 128.19, 128.17, 127.8, 127.68, 127.66, 127.59, 127.54, 126.0, 118.9, 114.5, 102.8, 101.4, 101.3, 81.7, 78.5, 78.3, 77.8, 76.8, 76.0, 74.9, 74.8, 73.3, 72.8, 68.4, 67.6, 65.1, 55.6; HRMS (ESI) calcd for $C_{53}H_{52}O_{12}Na$ $[M + Na]^+$ 903.3356, found 903.3349.

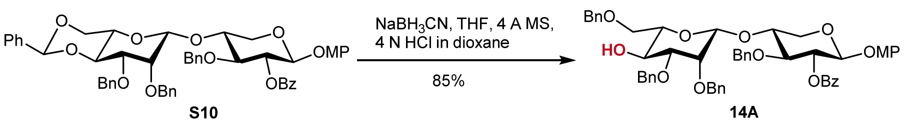

A similar procedure as that for the preparation of compound **4A** was used to provide **14A** as a white

solid: $[\alpha]_D^{20}$ = 78.4 (*c* 1.0, $CHCl_3$); ^{1}H NMR (600 MHz, $CDCl_3$) δ 8.06–8.01 (m, 2H), 7.60–7.54 (m, 1H), 7.45–7.42 (m, 2H), 7.41–7.39 (m, 2H), 7.34–7.25 (m, 13H), 7.17–7.13 (m, 3H), 7.12–7.09 (m, 2H), 6.94–6.87 (m, 2H), 6.79–6.73 (m, 2H), 5.44 (dd, *J* = 9.2, 7.5 Hz, 1H), 4.98 (d, *J* = 7.5 Hz, 1H), 4.88 (d, *J* = 12.5 Hz, 1H), 4.71 (d, *J* = 12.5 Hz, 1H), 4.67 (d, *J* = 11.7 Hz, 1H), 4.61 (d, *J* = 12.0 Hz, 1H), 4.56–4.53 (m, 2H), 4.52 (d, *J* = 0.8 Hz, 1H), 4.44 (d, *J* = 11.8 Hz, 1H), 4.36 (dd, *J* = 12.0, 5.5 Hz, 1H), 4.34 (d, *J* = 11.8 Hz, 1H), 3.97–3.91 (m, 2H), 3.82–3.74 (m, 3H), 3.73 (s, 3H), 3.69–3.67 (m, 1H), 3.46 (dd, *J* = 12.0, 9.8 Hz, 1H), 3.38 (ddd, *J* = 9.6, 5.7, 4.1 Hz, 1H), 3.15 (dd, *J* = 9.4, 2.9 Hz, 1H), 2.63 (d, *J* = 2.0 Hz, 1H); ^{13}C NMR (151 MHz, $CDCl_3$) δ 165.2, 155.5, 151.2, 138.6, 138.0, 137.9, 137.8, 133.2, 129.78, 129.76, 128.51, 128.46, 128.43, 128.31, 128.2, 128.1, 127.9, 127.8, 127.70, 127.69, 127.54, 127.51, 118.9, 114.5, 102.5, 101.2, 81.7, 81.5, 78.1, 74.9, 74.8, 74.2, 73.8, 73.6, 73.2, 71.7, 70.6, 68.3, 65.2, 55.6; HRMS (ESI) calcd for $C_{53}H_{54}O_{12}Na$ $[M + Na]^+$ 905.3513, found 905.3504.

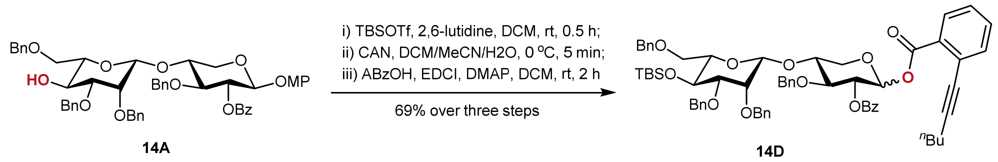

A similar procedure as that for the preparation of compound **4D** was used to provide **14D** as a white foam (α/β = 0.41:1): ^{1}H NMR (600 MHz, $CDCl_3$) δ 8.10–8.05 (m, 0.12H), 7.98–7.87 (m, 3H), 7.58–7.44 (m, 2H), 7.41–7.34 (m, 5H), 7.34–7.26 (m, 10H), 7.25–7.20 (m, 2H), 7.19–7.12 (m, 3H), 7.11–7.07 (m, 2H), 6.65–6.60 (m, 0.29H), 6.03 (d, *J* = 7.3 Hz, 0.71H), 5.47 (dd, *J* = 8.6, 7.3 Hz, 0.67H), 5.40–5.34 (m, 0.30H), 4.87–4.79 (m, 1H), 4.73–4.50 (m, 6H), 4.46–4.30 (m, 3H), 4.28–4.22 (m, 0.28H), 4.07 (dd, *J* = 9.8, 8.7 Hz, 0.29H), 4.00–3.92 (m, 1H), 3.91–3.86 (m, 1H), 3.84–3.78 (m, 1H), 3.78–3.74 (m, 1H), 3.70–3.67 (m, 0.28H), 3.66–3.58 (m, 2H), 3.45–3.31 (m, 1H), 3.21–3.07 (m, 1H), 2.56–2.26 (m, 2H), 1.74–1.32 (m, 4H), 0.95–0.84 (m, 3H), -0.02 (s, 3H), -0.04 (s, 3H); ^{13}C NMR (151 MHz, $CDCl_3$) δ 165.4, 165.2, 164.7, 164.2, 163.8, 138.94, 138.91, 138.89, 138.36, 138.34, 138.23, 137.99, 137.97, 137.92, 134.7, 134.5, 133.5, 133.3, 133.2, 132.2, 132.0, 131.8, 131.0, 130.8, 130.14, 130.08, 129.9, 129.8, 129.64, 129.62, 129.4, 128.6, 128.42, 128.38, 128.36, 128.35, 128.29, 128.24, 128.22, 128.08, 127.99, 127.97, 127.82, 127.78, 127.66, 127.63, 127.60, 127.53, 127.52, 127.48, 127.46, 127.36, 127.34, 127.31, 127.29, 127.2, 127.1, 127.0, 125.7, 125.3, 102.5, 101.9, 97.2, 97.1, 96.7, 93.0, 90.7, 82.7, 82.6, 80.3, 79.3, 79.1, 78.99, 78.97, 78.4, 77.3, 77.2, 77.0, 76.8, 75.3, 75.2, 74.7, 74.64, 74.58, 74.28, 74.24, 74.15, 73.3, 72.1, 72.0, 71.7, 71.45, 71.43, 71.3, 69.72, 69.69, 68.0, 65.7, 63.7, 63.6, 30.7, 30.6, 25.9, 22.1, 22.0, 19.54, 19.49, 18.1, 13.7, 13.6, -3.8, -4.9; HRMS (ESI) calcd for $C_{65}H_{74}O_{12}Na$ $[M + Na]^+$ 1097.4847, found 1097.4839.

4.2 Synthesis of fully protected 16-mer glycan 15

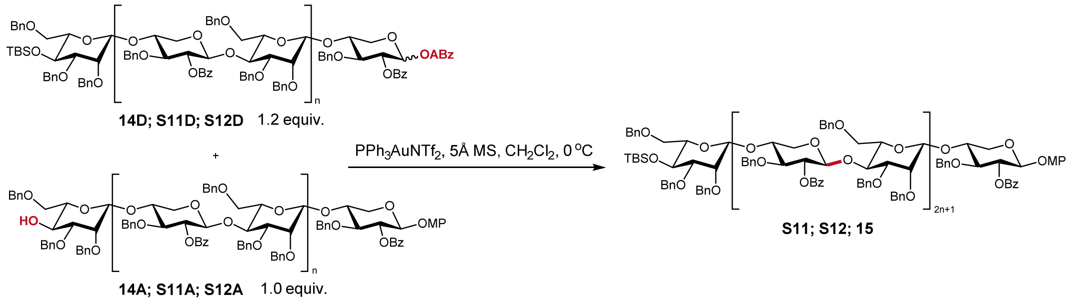

n	Substrates	PPh_3AuNTf_2 (eq.)	DCM/5Å MS	*c*	time	Method for purification	Product
0	**14D** (3.65 g) + **14A** (2.73 g)	229 mg (0.10)	62 mL/6.2 g	50 mM	1 h	EA/DCM/PE = 1:1:4, R_f = 0.4	**S11**, 5.4 g, 95%
1	**S11D** (2.25 g) + **S11A** (1.83 g)	165 mg (0.20)	50 mL/5.0 g	22 mM	1 h	EA/DCM/PE = 1:1:2.5, R_f = 0.6	**S12**, 3.5 g, 95%
3	**S12D** (1.21 g) + **S12A** (0.99 g)	46 mg (0.20)	20 mL/4.0 g	16 mM	2 h	EA/DCM/PE = 1:1:2, R_f = 0.4-0.6 [a]	**15**, 1.8 g, 93%

[a] TLC exhibited an elongated spot.

Figure S20. Preparation of the [→4)-*β*-L-Man*p*-(1→4)-*β*-D-Xyl*p*-(1→]$_n$OMP glycans via the gold (I)-catalyzed [2^n+2^n] glycosylation.

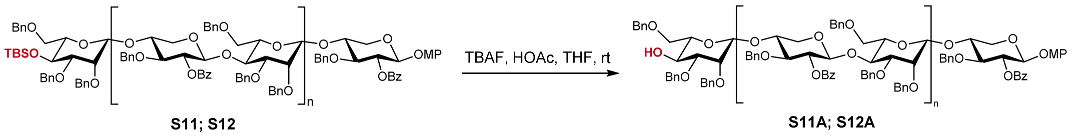

n	Substrates	**TBAF** (eq.)[a]; **HOAc** (eq.); THF	*c*	time	Method for purification	Product
1	**S11** (2.18 g)	12.4 mL (10.0); 142 μL (2.0); 30 mL	41 mM	8 h	EA/DCM/PE = 1:1:2.5, R_f = 0.15	**S11A**, 1.93 g, 95%
3	**S12** (1.42 g)	4.34 mL (10.0); 50 μL (2.0); 20 mL	22 mM	3 h	EA/DCM/PE = 1:1:2, R_f = 0.25	**S12A**, 1.10 g, 80%

[a] 1.0 M solution in THF.

Figure S21. Removal of the TBS group at the non-reducing end for the preparation of the [→4)-*β*-L-Man*p*-(1→4)-*β*-D-Xyl*p*-(1→]$_n$OMP acceptors.

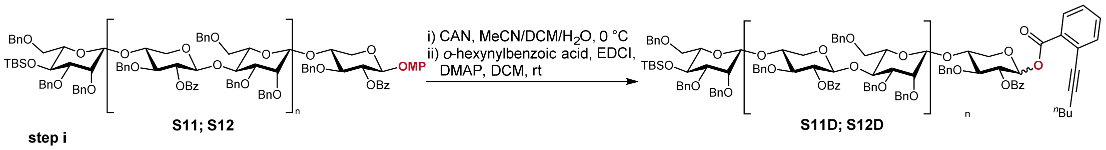

step i

n	Substrates	**CAN** (eq.); **MeCN/DCM/H_2O**	*c*	time	Method for purification	Product
1	**S11** (3.11 g)	2.91 g (3.0); 50/25/5 mL	39 mM	5 min	EA/DCM/PE = 1:1:2.5, R_f = 0.2-0.4	used directly
3	**S12** (1.95 g)	1.31 g (3.0); 50/25/5 mL	7.5 mM	5 min	EA/DCM/PE = 1:1:2, R_f = 0.2-0.5	used directly

step ii

n	**ABzOH** (eq.); **EDCI** (eq.); **DAMP** (eq.); **DCM**	*c*	Time	Method for purification	**Product** (over 2 steps)
1	537 mg (1.5); 846 mg (2.5); 540 mg (2.5), 30 mL	59 mM	2 h	EA/PE = 1:3, R_f = 0.5	**S11D**, 2.57 g, 79%
3	181 mg (1.5); 285 mg (2.5); 182 mg (2.5), 30 mL	20 mM	4 h	EA/DCM/PE = 1:1:2, R_f = 0.70-0.80	**S12D**, 1.73 g, 87%

Figure S22. Removal of the anomeric MP and subsequent condensation with *o*-hexynylbenzoic acid for the preparation of the [→4)-*β*-L-Man*p*-(1→4)-*β*-D-Xyl*p*-(1→]$_n$OMP donors.

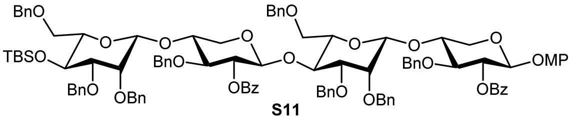

S11

A white foam. $[\alpha]_D^{20}$ = 41.1 (*c* 1.0, $CHCl_3$); ^1H NMR (500 MHz, $CDCl_3$) δ 8.07–8.01 (m, 2H), 7.93–7.79 (m, 2H), 7.63–7.51 (m, 2H), 7.48–7.29 (m, 26H), 7.28–7.12 (m, 10H), 7.11–6.98 (m, 7H), 6.97–6.90 (m, 2H), 6.82–6.77 (m, 2H), 5.45 (dd, *J* = 9.1, 7.4 Hz, 1H), 5.19 (dd, *J* = 9.5, 7.8 Hz, 1H), 5.02 (d, *J* = 7.8 Hz, 1H), 4.98 (d, *J* = 7.5 Hz, 1H), 4.86 (d, *J* = 12.5 Hz, 1H), 4.80 (d, *J* = 12.5 Hz, 1H), 4.71 (dd, *J* = 14.4, 12.5 Hz, 2H), 4.64 (dd, *J* = 13.8, 12.0 Hz, 2H), 4.63–4.54 (m, 3H), 4.57–4.50 (m, 2H), 4.51–4.35 (m, 6H), 4.28 (dd, *J* = 12.0, 5.5 Hz, 1H), 4.19–4.06 (m, 2H), 4.04–3.96 (m, 2H), 3.98–3.89 (m, 2H), 3.90–3.81 (m, 2H), 3.79–3.64 (m, 7H), 3.60 (t, *J* = 9.1 Hz, 1H), 3.52 (d, *J* = 3.0 Hz, 1H), 3.50–3.30 (m, 4H), 3.16 (dd, *J* = 9.1, 2.8 Hz, 1H), 3.12 (dd, *J* = 9.3, 3.0 Hz, 1H), 0.84 (s, 8H), 0.03 (s, 3H), 0.01 (s, 3H); ^{13}C NMR (126 MHz, $CDCl_3$) δ 165.1, 165.0, 155.4, 151.2, 139.0, 138.7, 138.6, 138.4, 138.2, 138.11, 138.06, 138.00, 133.1, 133.0, 130.1, 129.78, 129.74, 129.71, 128.39, 128.38, 128.33, 128.31, 128.26, 128.24, 128.21, 128.18, 128.14, 128.06, 128.03, 127.94, 127.84, 127.78, 127.72, 127.64, 127.56, 127.50, 127.43, 127.37, 127.34, 127.31, 127.29, 126.6, 119.0, 118.9, 114.5, 114.4, 102.3, 102.1, 101.3, 101.2, 99.7, 82.6, 82.5, 82.0, 81.3, 78.7, 78.2, 77.3, 77.2, 77.0, 76.8, 74.9, 74.8, 74.7, 74.5, 74.4, 74.1, 73.9, 73.7, 73.5, 73.3, 73.2, 73.12, 73.09, 71.9, 71.8, 71.34, 71.29, 69.9, 69.7, 68.0, 65.1, 55.58, 55.55, 25.9, 18.1, -0.0, -3.8, -4.9; HRMS (ESI) calcd for $C_{105}H_{114}O_{22}SiNa$ $[M + Na]^+$ 1777.7469, found 1777.7471.

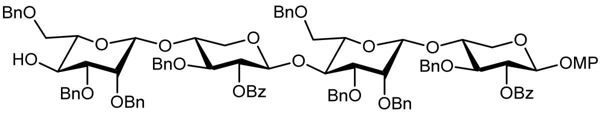

S11A

A white foam. $[\alpha]_D^{20}$ = 59.0 (*c* 0.5, $CHCl_3$); ^1H NMR (500 MHz, $CDCl_3$) δ 8.06–7.97 (m, 2H), 7.89–7.84 (m, 2H), 7.61–7.49 (m, 2H), 7.46–7.38 (m, 4H), 7.36–7.27 (m, 21H), 7.26–7.08 (m, 10H), 7.06–6.94 (m, 7H), 6.92–6.85 (m, 2H), 6.79–6.71 (m, 2H), 5.40 (dd, *J* = 9.2, 7.5 Hz, 1H), 5.15 (dd, *J* = 9.4, 7.8 Hz, 1H), 4.99 (d, *J* = 7.8 Hz, 1H), 4.96–4.92 (m, 1H), 4.86 (d, *J* = 12.6 Hz, 1H), 4.76 (d, *J* = 12.6 Hz, 1H), 4.72 (d, *J* = 12.5 Hz, 1H), 4.68–4.50 (m, 7H), 4.47 (d, *J* = 5.1 Hz, 2H), 4.45–4.37 (m, 4H), 4.35–4.29 (m, 1H), 4.17 (dd, *J* = 11.9, 5.6 Hz, 1H), 4.11 (d, *J* = 12.1 Hz, 1H), 4.05 (d, *J* = 12.1 Hz, 1H), 3.98–3.85 (m, 4H), 3.84–3.66 (m, 9H), 3.64 (d, *J* = 2.9 Hz, 1H), 3.58 (t, *J* = 9.1 Hz, 1H), 3.48 (d, *J* = 3.0 Hz, 1H), 3.44–3.27 (m, 4H), 3.12 (dd, *J* = 9.4, 2.9 Hz, 1H), 3.07 (dd, *J* = 9.3, 3.0 Hz, 1H), 2.64 (s, 1H); ^{13}C NMR (126 MHz, $CDCl_3$) δ 165.2, 165.0, 155.5, 151.2, 139.3, 138.74, 138.65, 138.63, 138.2, 138.09, 138.08, 137.9, 137.8, 133.2, 133.1 130.1, 129.79, 129.75, 128.54, 128.50, 128.47, 128.43, 128.41, 128.38, 128.31, 128.28, 128.20, 128.19, 128.1, 128.0, 127.9, 127.83, 127.81, 127.78, 127.73, 127.70, 127.63, 127.59, 127.56, 127.52, 127.47, 127.43, 127.39, 127.34, 126.7, 119.0, 118.9, 114.5, 114.4, 102.5, 102.2, 101.20, 101.18, 82.6, 82.2, 81.8, 81.6, 81.4, 78.6, 78.3, 77.3, 77.1, 76.8, 74.93, 74.90, 74.8, 74.5, 74.4, 74.1, 73.9, 73.73, 73.71, 73.66, 73.6, 73.2, 73.1, 71.8, 71.7, 71.3, 70.7, 69.9, 68.4, 65.2, 65.1, 55.63, 55.60; HRMS (ESI) calcd for $C_{99}H_{100}O_{22}Na$ $[M + Na]^+$ 1663.6604, found 1664.6603.

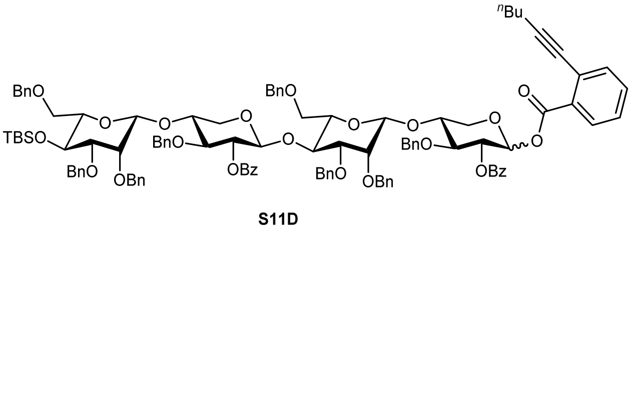

S11D

A white foam (α/β = 0.59:1): ^{1}H NMR (500 MHz, CDCl$_3$) δ 8.11–7.80 (m, 5H), 7.69–7.42 (m, 4H), 7.40–7.25 (m, 25H), 7.24–7.18 (m, 3H), 7.20–7.07 (m, 7H), 7.06–6.93 (m, 6H), 6.64–6.58 (m, 0.37H), 6.06–5.98 (m, 0.63H), 5.43 (dd, *J* = 8.5, 7.1 Hz, 0.61H), 5.32 (dt, *J* = 9.9, 3.5 Hz, 0.35H), 5.22–5.09 (m, 1H), 4.97 (dd, *J* = 7.8, 2.0 Hz, 1H), 4.91–4.29 (m, 17H), 4.25–4.19 (m, 1H), 4.14–4.09 (m, 0.35H), 4.07–3.85 (m, 6H), 3.85–3.74 (m, 3H), 3.70–3.52 (m, 4H), 3.53–3.20 (m, 4H), 3.14–3.08 (m, 1H), 3.10–2.97 (m, 1H), 2.49–2.26 (m, 2H), 1.65–1.30 (m, 4H), 0.97–0.83 (m, 3H), -0.01 (s, 3H), -0.04 (s, 3H); ^{13}C NMR (126 MHz, CDCl$_3$) δ 165.3, 165.1, 165.0, 164.71, 164.66, 164.2, 163.8, 163.6, 139.05, 139.00, 138.95, 138.63, 138.61, 138.56, 138.37, 138.32, 138.26, 138.21, 138.18, 138.12, 138.05, 138.01, 137.94, 137.88, 134.6, 134.5, 133.5, 133.23, 133.18, 133.0, 132.2, 132.0, 131.7, 131.1, 130.8, 130.6, 130.1, 123.0, 129.9, 129.8, 129.74, 129.71, 129.67, 129.4, 129.3, 128.5, 128.38, 128.35, 128.32, 128.30, 128.26, 128.25, 128.21, 128.18, 128.17, 128.09, 128.04, 128.03, 128.01, 127.93, 127.90, 127.84, 127.70, 127.64, 127.61, 127.58, 127.57, 127.50, 127.43, 127.37, 127.34, 127.31, 127.18, 127.09, 127.05, 126.9, 126.62, 126.59, 125.7, 125.3, 125.2, 102.29, 102.26, 102.1, 101.6, 101.2, 97.1, 97.0, 96.7, 92.94, 92.85, 90.7, 82.6, 82.5, 82.2, 82.0, 81.7, 81.6, 80.3, 80.1, 79.4, 79.1, 79.0, 78.9, 78.7, 78.6, 78.2, 77.3, 77.2, 77.0, 76.8, 75.9, 75.1, 75.02, 75.01, 74.9, 74.7, 74.6, 74.5, 74.4, 74.2, 74.1, 73.9, 73.82, 73.76, 73.7, 73.5, 73.40, 73.36, 73.31, 73.29, 73.26, 73.2, 72.1, 72.0, 71.8, 71.7, 71.6, 71.3, 71.22, 71.17, 69.94, 69.89, 69.7, 68.0, 65.5, 65.1, 63.6, 30.7, 30.6, 30.5, 25.9, 22.1, 22.0, 19.53, 19.47, 18.1, 13.71, 13.68, 13.66, 13.62, -0.0, -3.8, -4.91, -4.95; HRMS (ESI) calcd for $C_{111}H_{120}O_{22}SiNa$ [M + Na]$^+$ 1855.7938, found 1855.7942.

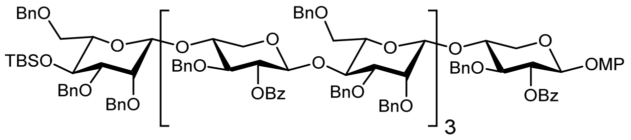

S12

A white foam. $[\alpha]_D^{20}$ = 46.8 (*c* 1.0, CHCl$_3$); ^{1}H NMR (500 MHz, CDCl$_3$) δ 8.05–7.95 (m, 3H), 7.88–7.79 (m, 6H), 7.59–7.04 (m, 86H), 7.03–6.86 (m, 19H), 6.80–6.71 (m, 2H), 5.45–5.36 (m, 1H), 5.18–5.07 (m, 3H), 5.00–4.89 (m, 4H), 4.86–4.28 (m, 35H), 4.25–4.17 (m, 3H), 4.12–3.85 (m, 16H), 3.84–3.59 (m, 16H), 3.58–3.22 (m, 16H), 3.11 (dd, *J* = 9.1, 2.8 Hz, 1H), 3.08–2.99 (m, 3H), 0.80 (s, 9H), -0.01 (s, 3H), -0.04 (s, 3H); ^{13}C NMR (126 MHz, CDCl$_3$) δ 165.2, 165.0, 164.9, 155.4, 151.2, 139.0, 138.7, 138.64, 138.62, 138.5, 138.4, 138.3, 138.2, 138.13, 138.08, 138.02, 133.1, 133.0, 129.79, 129.76, 129.72, 129.70, 128.44, 128.39, 128.33, 128.31, 128.26, 128.25, 128.22, 128.19, 128.14, 128.09, 128.05, 128.03, 127.93, 127.85, 127.65, 127.59, 127.55, 127.50, 127.46, 127.44, 127.41, 127.38, 127.35, 127.33, 127.31, 127.29, 127.27, 127.15, 127.12, 127.07, 126.63, 126.60, 126.58, 119.1, 118.9, 114.5, 114.4, 102.3, 102.14, 102.06, 101.2, 101.1, 82.65, 82.60, 82.55, 82.2, 82.0, 81.3, 78.7, 78.5, 78.3, 77.3, 77.0, 76.8, 75.8, 75.0, 74.9, 74.7, 74.46, 74.44, 74.38, 74.1, 73.9, 73.79, 73.75, 73.54, 73.49, 73.32, 73.26, 73.17, 73.12, 73.10, 71.8, 71.4, 71.27, 71.25, 71.21, 70.0, 69.7, 68.0, 65.1, 65.0, 55.6, 25.9, 18.1, -3.8, -4.9; MALDI FT-ICR MS calcd for $C_{197}H_{206}O_{42}Na$ [M + Na]$^+$ 3294.365, found 3294.362.

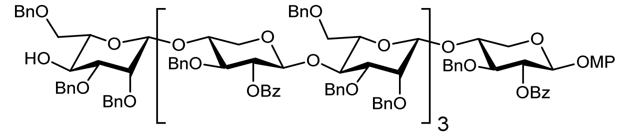

S12A

A white foam. $[\alpha]_D^{20}$ = 44.8 (*c* 1.0, CHCl$_3$); ^{1}H NMR (500 MHz, CDCl$_3$) δ 8.06–7.97 (m, 2H), 7.89–

7.79 (m, 6H), 7.59–7.45 (m, 4H), 7.44–7.38 (m, 4H), 7.37–7.25 (m, 46H), 7.24–7.09 (m, 21H), 7.03–6.99 (m, 6H), 6.99–6.93 (m, 5H), 6.93–6.87 (m, 8H), 6.79–6.70 (m, 2H), 5.40 (dd, *J* = 9.2, 7.5 Hz, 1H), 5.19–5.08 (m, 3H), 4.99 (d, *J* = 7.8 Hz, 1H), 4.97–4.91 (m, 3H), 4.87 (d, *J* = 12.5 Hz, 1H), 4.78–4.70 (m, 4H), 4.68–4.59 (m, 5H), 4.59–4.29 (m, 22H), 4.24–4.15 (m, 3H), 4.12–3.85 (m, 15H), 3.84–3.62 (m, 14H), 3.58 (t, *J* = 9.1 Hz, 1H), 3.54–3.21 (m, 14H), 3.13 (dd, *J* = 9.4, 2.9 Hz, 1H), 3.09–2.97 (m, 3H), 2.65 (s, 1H); ^{13}C NMR (126 MHz, $CDCl_3$) δ 165.1, 165.0, 164.9, 155.4, 151.2, 138.69, 138.66, 138.63, 138.2, 138.13, 138.08, 138.05, 137.84, 137.81, 133.1, 133.0, 132.9, 129.78, 129.75, 129.70, 128.50, 128.47, 128.43, 128.38, 128.36, 128.33, 128.31, 128.26, 128.24, 128.22, 128.19, 128.16, 128.14, 128.09, 128.05, 127.93, 127.86, 127.80, 127.66, 127.62, 127.59, 127.54, 127.46, 127.43, 127.39, 127.37, 127.35, 127.31, 127.29, 127.27, 127.1, 126.63, 126.60, 126.58, 126.54, 119.1, 118.9, 114.5, 114.4, 102.5, 102.13, 102.07, 101.2, 101.1, 82.60, 82.55, 82.20, 82.16, 81.7, 81.3, 78.55, 78.51, 78.48, 78.30, 77.28, 77.0, 76.8, 75.0, 74.89, 74.85, 74.79, 74.47, 74.45, 74.43, 74.1, 73.85, 73.79, 73.74, 73.69, 73.64, 73.56, 73.53, 73.49, 73.19, 73.12, 73.09, 71.8, 71.7, 71.3, 71.2, 70.6, 69.9, 68.4, 65.1, 65.0, 55.6, 55.5; MALDI FT-ICR MS calcd for $C_{191}H_{192}O_{42}Na$ [M + Na]$^+$ 3180.278, found 3180.276.

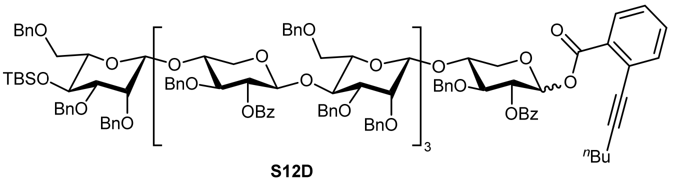

S12D

A white foam (α/β = 0.89:1): ^{1}H NMR (500 MHz, $CDCl_3$) δ 8.09–7.78 (m, 9H), 7.58–7.42 (m, 5H), 7.40–7.25 (m, 54H), 7.24–7.07 (m, 20H), 7.06–6.86 (m, 17H), 6.62–6.59 (m, 0.47H), 6.05–6.00 (m, 0.53H), 5.42 (dd, *J* = 8.4, 7.1 Hz, 0.52H), 5.36–5.28 (m, 0.50H), 5.20–5.05 (m, 3H), 5.02–4.88 (m, 3H), 4.87–4.25 (m, 33H), 4.27–4.16 (m, 4H), 4.13–3.85 (m, 16H), 3.86–3.70 (m, 5H), 3.70–3.19 (m, 20H), 3.11 (dd, *J* = 9.1, 2.8 Hz, 1H), 3.08–2.95 (m, 3H), 2.52–2.25 (m, 2H), 1.64–1.33 (m, 4H), 0.97–0.83 (m, 3H), 0.80 (s, 9H), -0.01 (s, 3H), -0.03 (s, 3H); ^{13}C NMR (126 MHz, $CDCl_3$) δ 165.3, 165.1, 165.0, 164.9, 164.2, 163.8, 139.0, 138.64, 138.62, 138.58, 138.38, 138.34, 138.23, 138.1, 138.0, 137.9, 134.6, 134.5, 133.22, 133.16, 133.0, 132.9, 132.2, 132.0, 131.1, 130.8, 130.6, 130.1, 129.9, 129.8, 129.74, 129.70, 129.39, 129.35, 128.6, 128.4, 128.33, 128.31, 128.29, 128.26, 128.24, 128.22, 128.18, 128.15, 128.08, 128.04, 128.00, 127.8, 127.65, 127.63, 127.60, 127.58, 127.55, 127.49, 127.46, 127.43, 127.38, 127.35, 127.31, 127.28, 127.17, 127.11, 127.05, 126.60, 126.58, 125.65, 125.3, 102.30, 102.26, 102.1, 101.6, 101.2, 101.1, 97.1, 97.0, 92.9, 90.7, 82.65, 82.60, 82.55, 82.18, 82.15, 82.0, 80.1, 79.4, 79.1, 79.0, 78.9, 78.7, 78.5, 78.3, 77.3, 77.2, 77.0, 76.8, 75.1, 75.04, 75.02, 74.99, 74.7, 74.5, 74.4, 74.1, 73.92, 73.80, 73.78, 73.54, 73.49, 73.39, 73.31, 73.26, 73.17, 73.1, 72.0, 71.8, 71.6, 71.34, 71.29, 71.24, 71.21, 71.1, 69.9, 69.7, 68.0, 65.5, 65.1, 65.0, 63.6, 30.7, 30.6, 30.5, 25.9, 22.1, 22.0, 19.53, 19.47, 18.1, 13.72, 13.69, 13.67, 13.62, -3.8, -4.9; MALDI FT-ICR MS calcd for $C_{203}H_{212}O_{42}Na$ [M + Na]$^+$ 3372.411, found 3372.409.

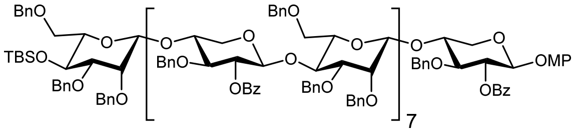

15

A white foam. $[\alpha]_D^{20}$ = 42.1 (*c* 1.0, $CHCl_3$); ^{1}H NMR (500 MHz, $CDCl_3$) δ 8.04–7.97 (m, 2H), 7.87–

7.78 (m, 14H), 7.58–7.46 (m, 9H), 7.44–7.07 (m, 162H), 7.04–6.86 (m, 40H), 6.79–6.73 (m, 2H), 5.40 (dd, J = 9.1, 7.4 Hz, 1H), 5.18–5.07 (m, 7H), 4.99–4.91 (m, 8H), 4.86–4.26 (m, 65H), 4.26–4.17 (m, 8H), 4.14–3.85 (m, 31H), 3.84–3.60 (m, 22H), 3.59–3.20 (m, 33H), 3.11 (dd, J = 9.1, 2.8 Hz, 1H), 3.09–2.97 (m, 7H), 0.80 (s, 9H), -0.01 (s, 3H), -0.04 (s, 3H); ^{13}C NMR (126 MHz, $CDCl_3$) δ 165.2, 165.0, 164.9, 155.4, 151.2, 139.0, 138.69, 138.65, 138.62, 138.4, 138.26, 138.23, 138.15, 138.10, 138.0, 133.1, 132.9, 129.81, 129.76, 129.7, 128.4, 128.33, 128.32, 128.27, 128.22, 128.14, 128.08, 128.0, 127.93, 127.85, 127.65, 127.59, 127.55, 127.50, 127.47, 127.44, 127.40, 127.37, 127.3, 127.1, 126.63, 126.61, 126.58, 118.9, 114.4, 102.3, 102.2, 102.1, 101.2, 101.1, 82.7, 82.2, 82.0, 81.3, 78.7, 78.6, 78.4, 77.3, 77.0, 76.8, 75.0, 74.9, 74.7, 74.44, 74.39, 74.1, 73.9, 73.8, 73.3, 73.2, 73.1, 71.3, 71.24, 71.19, 70.0, 69.7, 68.0, 65.0, 55.6, 25.9, 18.1, -3.8, -4.9; MALDI FT-ICR MS calcd for $C_{381}H_{390}O_{82}Na$ [M + Na]$^+$ 6327.601, found 6327.578.

4.3 Preparation of free 16-mer 15F

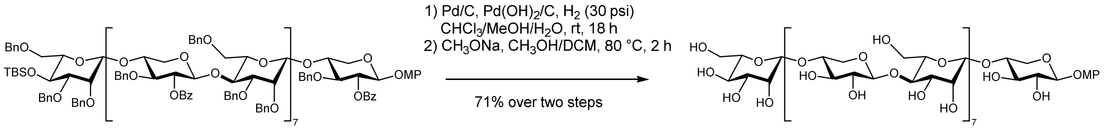

16-Mer **15** (60 mg) was dissolved in a mixture of $MeOH/CHCl_3/H_2O$ (3/6/0.2, 9.2 mL) containing 10% Pd/C (90 mg, wetted with 55% H_2O) and 20% $Pd(OH)_2/C$ (90 mg, wetted with 50% H_2O). The resulting mixture was stirred under H_2 atmosphere (30 Psi) at 30 °C for 18 hours, and was then filtrated through a pad of Celite. The Celite pad was washed with $MeOH/CHCl_3$ (1:1, 6 mL), $MeOH/CHCl_3$ (2:1, 6 mL), $MeOH/CHCl_3$ (3:1, 4 mL), MeOH (3 mL), and $CHCl_3$ (3 mL) successively. The combined filtrate was concentrated to afford a solid (30 mg).

To the suspension of the above solid (30 mg) in CH_3OH/CH_2Cl_2 (2/1, 7.5 mL) was added CH_3ONa (25 mg, excess). The suspension was stirred vigorously at 80 °C for 2 hours to give a white slurry, which was then neutralized with Dowex 50 WX8 (H) resin. The resulting mixture was filtrated through a pad of Celite, and the Celite pad was washed with H_2O. The combined filtrate was concentrated *in vacuo* and purified by gel filtration (Sephadex G-25, H_2O) to give **15F** (17 mg, 71% over two steps) as a white powder after lyophilization: ^{1}H NMR (600 MHz, D_2O, 40 °C, δ_{HDO} = 4.60 ppm) δ 7.10–7.06 (m, 2H), 6.99–6.95 (m, 2H), 4.93 (d, J = 7.7 Hz, 1H), 4.81 (s, 1H), 4.78 (s, 11H), 4.65–4.63 (m, 14H), 4.21–4.10 (m, 10H), 4.06–4.02 (m, 9H), 3.92–3.87 (m, 10H), 3.85–3.81 (m, 8H), 3.81–3.66 (m, 29H), 3.56 (t, J = 9.3 Hz, 9H), 3.44–3.39 (m, 8H), 3.36–3.31 (m, 7H), 3.31–3.27 (m, 7H); ^{13}C NMR (151 MHz, D_2O, 40 °C, δ_{DSS} = 0.0 ppm) δ 157.6, 153.3, 121.1, 117.9, 106.3, 104.4, 103.68, 103.62, 103.59, 80.8, 80.7, 80.6, 78.9, 77.7, 77.28, 77.25, 77.1, 76.2, 75.63, 75.59, 75.53, 75.47, 73.23, 73.15, 73.08, 69.4, 67.3, 67.2, 63.7, 63.5, 58.6; MALDI FT-ICR MS calcd for $C_{95}H_{152}O_{74}Na$ [M + Na]$^+$ 2499.802, found 2499.801.

4.4 Spectral comparison of synthetic 16-mer 15F with the natural xylomannan

Table S3. ^{13}C NMR comparison between the synthetic 16-mer **15F** and the natural xylomannan reported

by Walters et al.

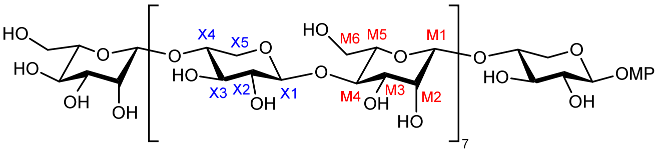

16-mer **15F**

Position	X1	X2	X3	X4	X5	M1	M2	M3	M4	M5	M6
16-mer **15F** (ppm)	103.0	72.9	73.9	77.4	64.0	100.3	69.9	72.2	74.4	74.0	60.2
Walters' report (ppm)[5]	101.7	72.8	73.8	76.6	63.0	100.2	70.1	71.6	76.6	75.1	60.6
Discrepancy (ppm)	**1.3**	0.1	0.1	0.8	**1.0**	0.1	-0.2	0.6	**-2.2**	**-1.1**	-0.4

5. Synthesis of block-wise xylomannan 32-mer 23F

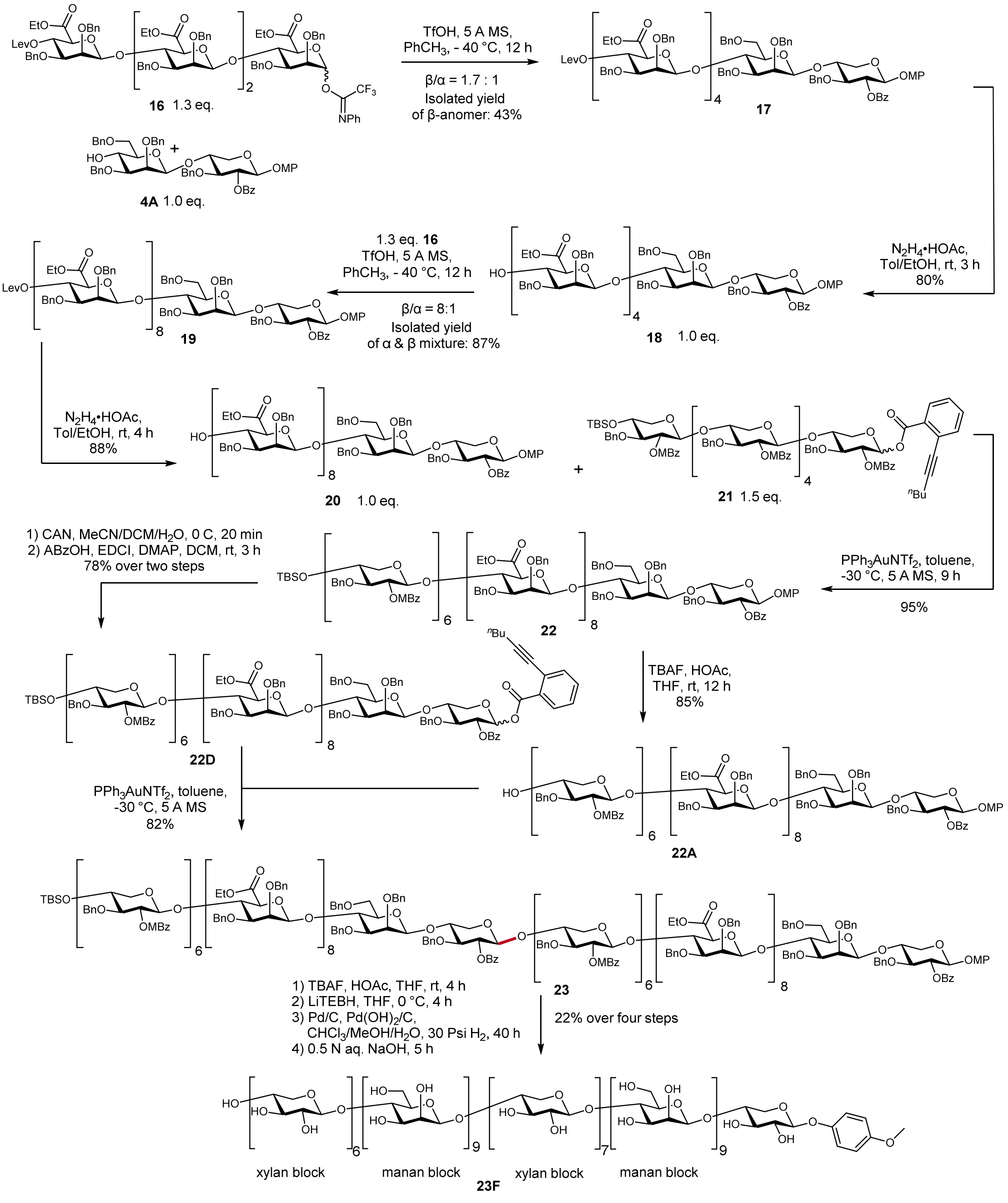

Figure S23. Preparation of the block-wise xylomannan 32-mer **23F**. MBz, *p*-methylbenzoyl.

5.1 Preparation of xylan 6-mer donor 21

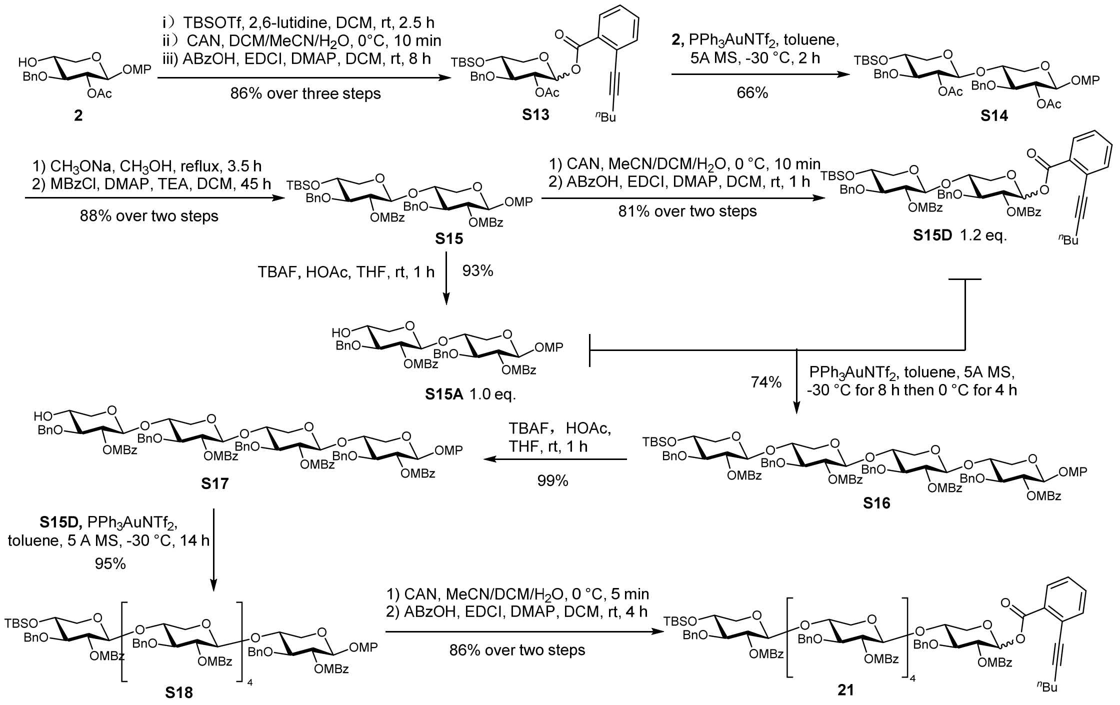

Figure S24. Preparation of xylan 6-mer *o*-hexynylbenzoate donor **21**. MBz, *p*-methylbenzoyl.

Xylopyranoside **2** was converted to glycosyl donor **S13** in 86% yield over three steps. Initial attempts at the coupling of xylopyranoside **2** with **S13** under the catalysis of $Ph_3PAuNTf_2$ afforded disaccharide **S14** as a mixture of anomers.[7,8] Pleasingly, we screened several reaction solvents and found that utilization of toluene led to satisfactory 66% yield and 25:1 β/α selectivity.

In further glycan assembly, we replaced the 2-*O*-acetyl group into 2-*O*-*p*-methylbenzoyl group (MBz) as the neighboring participating group,[9] thus converting disaccharide **S14** to **S15** in 88% yield over two steps. Disaccharide **S15** was transformed to glycosyl donor **S15D** (81%, two steps) and acceptor **S15A** (93%), respectively. Condensation of **S15D** and **S15A** under the action of $Ph_3PAuNTf_2$ in toluene provided tetrasaccharide **S16** in 74% yield.

Removal of the TBS group in **S16** by treatment with TBAF furnished **S17** nearly quantitatively (99%). Glycosylation of **S17** with **S15D** under the catalysis of $Ph_3PAuNTf_2$ in toluene afforded hexasaccharide **S18** in an excellent 95% yield. **S18** was then elaborated to the desired hexasaccharide donor **21** in a good 86% yield over two steps.

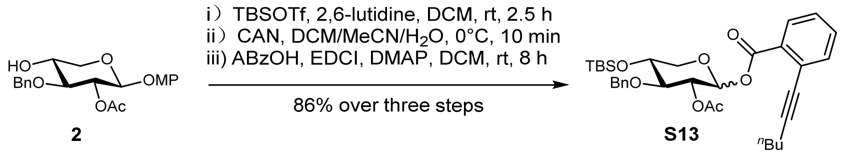

To a solution of **2** (6.00 g, 15.5 mmol), 2,6-lutidine (9.0 mL, 77.3 mmol) in anhydrous CH_2Cl_2 (90 mL) was added TBSOTf (8.90 mL, 38.6 mmol) at 0 °C. The mixture was warmed up to room temperature and kept stirring for 2.5 hours, to which MeOH/Et_3N (15 mL/15 mL) was added. The mixture was washed with 1N HCl, saturated aqueous $NaHCO_3$, and brine successively. The organic layer was dried over anhydrous Na_2SO_4, filtered, and concentrated *in vacuo*. The resulting residue was used for the next step directly.

To a solution of the above residue in CH_2Cl_2/MeCN/H_2O (96 mL / 48 mL /19 mL) was added ammonium ceric nitrate (25.4 g, 46.4 mmol) at 0 °C. The mixture was stirred at 0 °C for 10 minutes, and was then poured into saturated aqueous $NaHCO_3$ to quench the reaction. The aqueous layer was extracted with EtOAc (3 × 150 mL). The combined organic layer was dried over anhydrous Na_2SO_4 and concentrated *in vacuo*. The residue was purified by silica gel chromatography (EtOAc/petroleum ether, 1:2.5, R_f = 0.5) to afford a brown foam, which was used for the next step directly.

To a mixture of the above foam, *ortho*-hexynylbenzoic acid (4.69 g, 23.2 mmol), 1-(3-dimethylaminopropyl)-3-ethylcarbodiimide hydrochloride (EDCI) (7.41 g, 38.6 mmol), and 4-dimethylaminopyridine (4.72 g, 38.6 mmol) was added CH_2Cl_2 (60 mL). The resulting mixture was stirred at room temperature for 8 hours, and was then poured into saturated aqueous $NaHCO_3$. The aqueous layer was extracted with CH_2Cl_2 (2 × 50 mL). The combined organic layer was dried over anhydrous Na_2SO_4 and concentrated *in vacuo*. The resulting residue was purified by silica gel chromatography (EtOAc/petroleum ether, 1:4, R_f = 0.7) to afford **S13** as a white foam (6.66 g, α/β =2.5:1, 86% over three steps). ^{1}H NMR (600 MHz, $CDCl_3$) δ 7.93–7.89 (m, 0.80H), 7.71 (dd, J = 7.9, 1.4 Hz, 0.21H), 7.57 (dd, J = 7.8, 1.3 Hz, 0.50H), 7.52–7.46 (m, 1H), 7.45–7.39 (m, 0.53H), 7.38–7.35 (m, 0.50H), 7.36–7.21 (m, 4.6H), 7.22–7.15 (m, 1H), 6.48 (d, J = 3.7 Hz, 0.50H), 6.35 (d, J = 3.8 Hz, 0.20H), 5.84 (d, J = 7.4 Hz, 0.30H), 5.20 (dd, J = 9.5, 3.8 Hz, 0.21H), 5.16 (dd, J = 8.7, 7.4 Hz, 0.30H), 5.04 (dd, J = 9.5, 3.7 Hz, 0.52H), 4.87–4.80 (m, 1H), 4.77 (d, J = 11.2 Hz, 0.20H), 4.68 (dd, J = 11.6, 9.1 Hz, 0.84H), 3.97 (dd, J = 11.7, 5.0 Hz, 0.32H), 3.92–3.80 (m, 2.32H), 3.77–3.70 (m, 0.73H), 3.67–3.62 (m, 0.21H), 3.57 (t, J = 8.3 Hz, 0.31H), 3.45 (dd, J = 11.7, 9.2 Hz, 0.29H), 2.53 (t, J = 7.1 Hz, 1H), 2.48 (q, J = 7.2 Hz, 1H), 2.14 (s, 0.63H), 1.89 (s, 1.52H), 1.86 (s, 0.91H), 1.67–1.58 (m, 2.09H), 1.54–1.46 (m, 2.07H), 0.97–0.93 (m, 3H), 0.92–0.90 (m, 9H), 0.11–0.08 (m, 6H); ^{13}C NMR (151 MHz, $CDCl_3$) δ 170.0, 169.6, 169.2, 164.7, 164.6, 163.7, 138.5, 138.3, 138.2, 135.0, 134.61, 134.59, 132.3, 132.0, 131.8, 130.8, 130.73, 130.66, 130.1, 129.7, 128.4, 128.28, 128.26, 128.16, 127.83, 127.68, 127.66, 127.63, 127.53, 127.50, 127.42, 127.25, 127.23, 127.0, 125.7, 125.2, 125.0, 97.2, 96.7, 96.6, 93.2, 91.1, 90.1, 81.7, 80.04, 80.00, 79.8, 79.2, 79.1, 75.4, 74.9, 71.9, 71.8, 71.06, 71.04, 70.95, 70.7, 66.6, 65.0, 64.5, 30.8, 30.7, 25.76, 25.74, 22.13, 22.11, 22.09, 20.9, 20.8, 20.6, 19.7, 19.63, 19.58, 18.02, 17.98, 17.96, 13.70, 13.69, -4.63, -4.69, -4.73, -4.75, -4.77; HRMS (ESI) calcd for $C_{33}H_{44}O_7SiNa$ $[M + Na]^+$ 603.2754, found 603.2752.

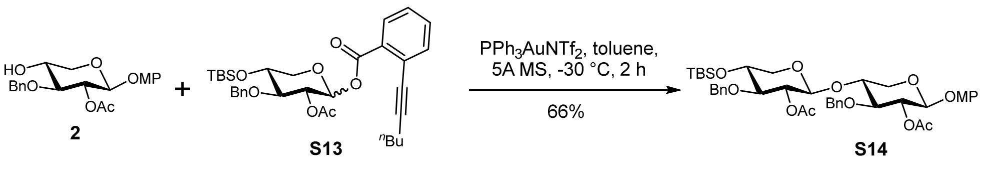

A mixture of xylose donor **S13** (6.65 g, 13.2 mmol) and acceptor **2** (4.28 g, 11.0 mmol) was azeotropically dried with toluene (2 × 30 mL), and then 5Å MS (15 g) and anhydrous toluene (150 mL) were added. The mixture was stirred at room temperature for 15 minutes, and was then cooled to -30 °C, to which $Ph_3PAuNTf_2$ (813 mg, 1.10 mmol) was added. The resulting mixture was stirred at -30 °C for another 2 hours, to which Et_3N (1 mL) was added to quench the reaction. The resulting mixture was filtered through a pad of Celite. The filtrate was concentrated *in vacuo* and purified by silica gel column chromatography (EtOAc/petroleum ether, 1:3, R_f = 0.5) to give **S14** as a white solid (5.56 g, 66%): $[\alpha]_D^{25}$ = -3.7 (c 1.0, $CHCl_3$); ^{1}H NMR (600 MHz, $CDCl_3$) δ 7.36–7.26 (m, 9H), 6.93–6.88 (m, 2H), 6.82–6.78 (m, 2H), 5.10 (dd, J = 8.3, 6.6 Hz, 1H), 4.91–4.83 (m, 3H), 4.81 (d,

J = 11.7 Hz, 1H), 4.68 (d, J = 11.7 Hz, 1H), 4.61 (d, J = 11.7 Hz, 1H), 4.39 (d, J = 7.5 Hz, 1H), 4.01 (dd, J = 11.9, 4.7 Hz, 1H), 3.91 (td, J = 8.1, 4.7 Hz, 1H), 3.85–3.78 (m, 2H), 3.76 (s, 3H), 3.60 (t, J = 7.9 Hz, 1H), 3.40 (dd, J = 9.2, 7.7 Hz, 1H), 3.29 (dd, J = 11.9, 8.4 Hz, 1H), 3.16–3.09 (m, 1H), 1.99 (s, 3H), 1.90 (s, 3H), 0.90 (s, 9H), 0.09 (s, 3H), 0.08 (s, 3H); ^{13}C NMR (151 MHz, $CDCl_3$) δ 169.6, 169.5, 155.4, 151.0, 138.4, 138.3, 128.3, 128.2, 128.0, 127.9, 127.63, 127.55, 118.4, 114.5, 100.7, 100.3, 82.2, 78.5, 76.1, 75.0, 74.0, 72.6, 71.22, 71.16, 66.1, 62.3, 55.6, 25.7, 20.9, 17.9, -4.7, -4.7; HRMS (ESI) calcd for $C_{41}H_{54}O_{12}SiNa$ [M + Na]$^+$ 789.3282, found 789.3281.

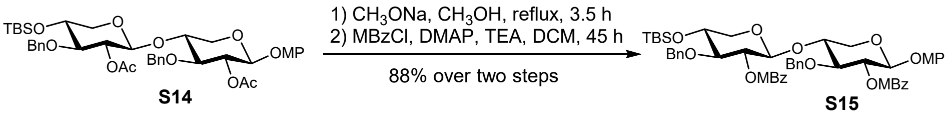

To a solution of **S14** (5.53 g, 7.21 mmol) in CH_3OH (50 mL) was added CH_3ONa (38 mg, 0.72 mmol). The mixture was refluxed for 3.5 hours, and was then concentrated *in vacuo*. The resultant residue was diluted with CH_2Cl_2 (200 mL), and washed with H_2O (2 × 200 mL). The organic layer was dried over anhydrous Na_2SO_4 and concentrated *in vacuo* to give a residue (petroleum ether /EtOAc, 1:2.5, R_f = 0.5), which was used for the next step without further purification.

To the above residue, 4-dimethylaminopyridine (879 mg, 7.20 mmol), and triethylamine (16.0 mL, 115 mmol) in CH_2Cl_2 (50 mL) was added 4-methylbenzoyl chloride (7.61 mL, 57.6 mmol) at 0 °C. The mixture was stirred at 45 °C for 2 hours, and was then quenched with CH_3OH (5 mL) at 0 °C. The resultant mixture was poured into saturated aqueous $NaHCO_3$. The water layer was extracted with CH_2Cl_2 (100 mL × 2). The organic phase was dried over anhydrous Na_2SO_4, concentrated *in vacuo* and purified by silica gel column chromatography (petroleum ether/EtOAc, 4:1, R_f = 0.5) to give **S15** as a white solid (5.81 g, 88% over 2 steps): $[\alpha]_D^{25}$ = -4.9 (c 2.0, $CHCl_3$); ^{1}H NMR (600 MHz, $CDCl_3$) δ 7.91–7.84 (m, 4H), 7.25–7.21 (m, 6H), 7.17–7.14 (m, 3H), 7.15–7.07 (m, 5H), 6.83–6.77 (m, 2H), 6.73–6.66 (m, 2H), 5.34 (dd, J = 8.3, 6.7 Hz, 1H), 5.16 (dd, J = 9.2, 7.5 Hz, 1H), 4.89 (d, J = 6.7 Hz, 1H), 4.82 (d, J = 11.4 Hz, 1H), 4.74 (d, J = 7.1 Hz, 1H), 4.72 (d, J = 7.1 Hz, 1H), 4.62 (d, J = 11.4 Hz, 1H), 4.56 (d, J = 7.6 Hz, 1H), 4.00–3.91 (m, 2H), 3.86–3.76 (m, 2H), 3.76–3.71 (m, 1H), 3.70 (s, 3H), 3.58–3.53 (m, 1H), 3.24–3.12 (m, 2H), 2.43 (s, 3H), 2.40 (s, 3H), 0.90 (s, 9H), 0.08 (s, 3H), 0.07 (s, 3H); ^{13}C NMR (151 MHz, $CDCl_3$) δ 165.24, 165.15, 155.3, 151.0, 143.8, 143.7, 138.1, 137.9, 129.9, 129.7, 129.2, 129.1, 128.2, 128.13, 128.12, 128.08, 127.98, 127.44, 127.41, 127.10, 127.07, 118.6, 114.4, 101.4, 100.5, 81.9, 78.4, 76.7, 74.8, 74.0, 73.1, 71.7, 71.3, 66.2, 62.6, 55.6, 25.7, 21.70, 21.68, 17.9, -4.7, -4.8; HRMS (ESI) calcd for $C_{53}H_{62}O_{12}Na$ [M + Na]$^+$ 941.3908, found 941.3906.

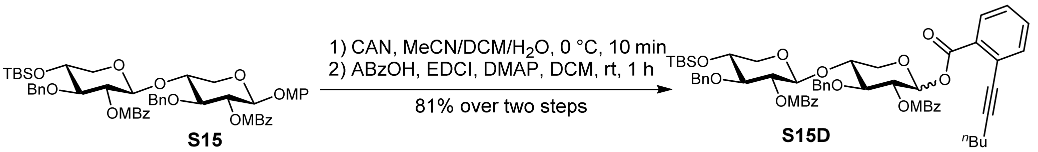

A similar procedure as that for the preparation of compound **4D** was used to provide **S15D** as a white foam (4.05 g, α/β = 1.4:1, 81% over two steps): ^{1}H NMR (500 MHz, $CDCl_3$) δ 7.89–7.80 (m, 4H), 7.77 (d, J = 8.0 Hz, 1H), 7.73 (dd, J = 7.9, 1.3 Hz, 0.55H), 7.49 (dd, J = 7.8, 1.3 Hz, 0.68H), 7.46–7.41 (m, 1H), 7.39–7.33 (m, 0.63H), 7.30–7.07 (m, 19H), 6.49 (d, J = 3.7 Hz, 0.58H), 5.90 (d, J = 6.4 Hz, 0.42H), 5.37 (dd, J = 7.6, 6.4 Hz, 0.56H), 5.26 (dd, J = 9.7, 3.7 Hz, 0.60H), 5.17–5.13 (m, 1H), 4.92 (d, J = 10.9 Hz, 0.71H), 4.82 (d, J = 11.3 Hz, 0.48H), 4.76–4.69 (m, 2H), 4.64–4.59

(m, 2H), 4.57 (d, J = 7.5 Hz, 0.44H), 4.06 (t, J = 9.3 Hz, 1H), 4.03–3.92 (m, 2H), 3.91–3.74 (m, 3H), 3.69 (dd, J = 11.5, 5.6 Hz, 1H), 3.61 (d, J = 11.2 Hz, 1H), 3.59–3.53 (m, 1H), 3.36 (dd, J = 12.0, 8.0 Hz, 0.49H), 3.17 (dd, J = 11.4, 9.4 Hz, 1H), 2.47–2.29 (m, 8H), 1.59–1.35 (m, 4H), 0.94–0.86 (m, 12H), 0.10–0.05 (m, 6H); ^{13}C NMR (600 MHz, $CDCl_3$) δ 165.43, 165.29, 165.23, 165.20, 164.1, 163.7, 143.93, 143.89, 143.87, 143.84, 138.3, 137.94, 137.91, 137.88, 134.7, 134.5, 132.1, 132.0, 130.8, 130.4, 130.3, 129.94, 129.87, 129.82, 129.75, 129.69, 129.62, 129.21, 129.19, 129.05, 128.25, 128.22, 128.13, 128.07, 128.05, 128.02, 127.99, 127.50, 127.47, 127.45, 127.40, 127.13, 127.04, 126.95, 126.8, 126.6, 125.6, 125.2, 101.8, 101.5, 97.07, 97.05, 92.8, 90.6, 81.92, 81.86, 79.3, 79.0, 77.6, 77.53, 77.51, 77.1, 76.2, 75.0, 74.84, 74.77, 74.1, 73.1, 72.9, 71.5, 71.3, 71.2, 70.1, 66.24, 66.19, 63.1, 61.9, 30.7, 30.6, 25.8, 22.1, 22.0, 21.69, 21.65, 19.52, 19.46, 17.95, 17.93, 13.7, -4.65, -4.68, -4.73, -4.77; HRMS (ESI) calcd for $C_{59}H_{68}O_{12}SiNa$ $[M + Na]^+$ 1019.4378, found 1019.4375.

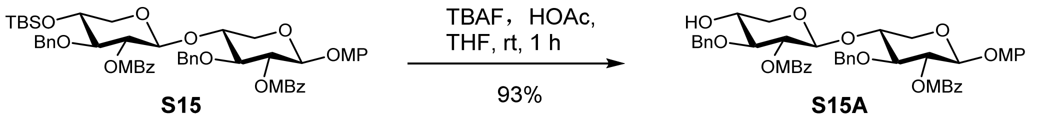

To a solution of compound **S15** (1.10 g, 1.20 mmol) in anhydrous tetrahydrofuran (10 mL) were added acetic acid (68.6 μL, 1.20 mmol) and tetrabutylammonium fluoride (6.00 mL, 1 M in THF) successively. The mixture was stirred at room temperature for 1 hour, and was then poured into saturated aqueous NH_4Cl. The aqueous layer was extracted with EtOAc two times. The combined organic layer was dried over anhydrous $NaSO_4$, filtered, concentrated *in vacuo*, and purified by silica gel column chromatography (EtOAc/petroleum ether, 1:2, R_f = 0.2) to give **S15A** as a white solid (970 mg, 93%): $[\alpha]_D^{25}$ = -39.4 (c 1.0, $CHCl_3$); ^{1}H NMR (600 MHz, $CDCl_3$) δ 7.95–7.92 (m, 2H), 7.91–7.87 (m, 2H), 7.28–7.20 (m, 10H), 7.21–7.11 (m, 5H), 6.85–6.79 (m, 2H), 6.76–6.68 (m, 2H), 5.37 (dd, J = 8.4, 6.8 Hz, 1H), 5.21 (dd, J = 7.5, 5.9 Hz, 1H), 4.93 (d, J = 6.7 Hz, 1H), 4.80 (d, J = 11.3 Hz, 1H), 4.73 (d, J = 11.5 Hz, 1H), 4.70 (d, J = 5.9 Hz, 1H), 4.67 (d, J = 11.2 Hz, 1H), 4.59 (d, J = 11.5 Hz, 1H), 4.08 (dd, J = 11.8, 4.4 Hz, 1H), 4.05–4.00 (m, 2H), 3.82–3.73 (m, 2H), 3.71 (s, 3H), 3.63 (t, J = 7.3 Hz, 1H), 3.32–3.26 (m, 2H), 2.43 (s, 3H), 2.41 (s, 3H), 2.29 (d, J = 5.2 Hz, 1H); ^{13}C NMR (151 MHz, $CDCl_3$) δ 165.3, 165.2, 155.4, 151.1, 144.3, 143.8, 138.0, 137.7, 129.9, 129.7, 129.4, 129.1, 128.5, 128.2, 128.1, 128.04, 128.00, 127.5, 127.1, 126.8, 118.6, 114.4, 100.7, 100.0, 80.4, 78.8, 75.9, 74.2, 73.8, 72.1, 71.9, 68.8, 64.1, 62.5, 55.6, 21.72, 21.68; HRMS (ESI) calcd for $C_{47}H_{48}O_{12}Na$ $[M + Na]^+$ 827.3043, found 827.3045.

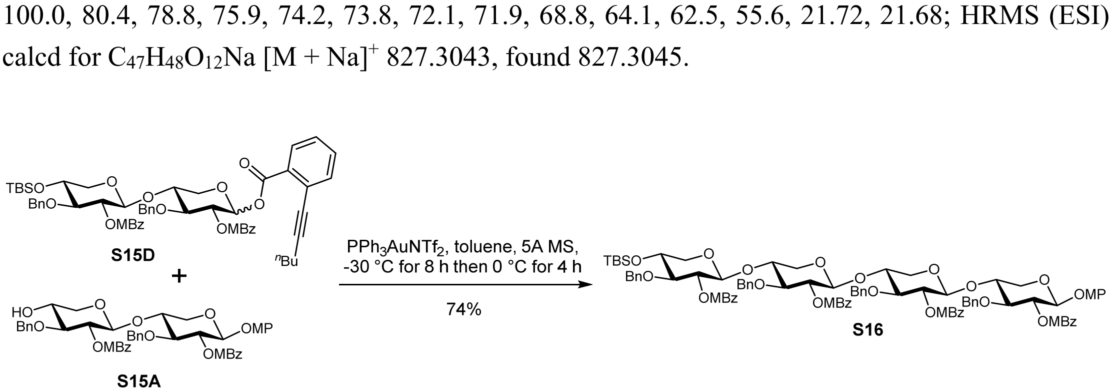

A mixture of disaccharide donor **S15D** (1.38 g, 1.38 mmol) and acceptor **S15A** (928 mg, 1.15 mmol) was azeotropically dried with toluene (2 × 15 mL), and then 5Å MS (1.5 g) and anhydrous toluene (15 mL) were added. The mixture was stirred at room temperature for 15 minutes, and was then cooled to -30 °C, to which $Ph_3PAuNTf_2$ (170 mg, 0.230 mmol) was added. The mixture was stirred

at -30 °C for 8 hours, and was then warmed up to 0 °C. After being stirred at 0 °C for 4 hours, Et_3N (2 mL) was added to quench the reaction. The resulting mixture was filtered through a pad of Celite. The filtrate was concentrated *in vacuo* and purified by silica gel column chromatography (EtOAc/ petroleum ether, 1:2, R_f = 0.7) to give **S16** as a white foam (1.37 g, 74%): $[\alpha]_D^{25}$ = -40.5 (*c* 1.0, $CHCl_3$); ^{1}H NMR (600 MHz, $CDCl_3$) δ 7.92–7.81 (m, 8H), 7.31–7.19 (m, 12H), 7.18–7.04 (m, 19H), 6.81–6.76 (m, 2H), 6.72–6.67 (m, 2H), 5.29 (dd, *J* = 8.5, 6.9 Hz, 1H), 5.18 (dd, *J* = 9.2, 7.6 Hz, 1H), 5.13 (dd, *J* = 8.7, 7.2 Hz, 1H), 5.08 (dd, *J* = 8.8, 7.2 Hz, 1H), 4.84 (s, 1H), 4.82 (d, *J* = 3.8 Hz, 1H), 4.79–4.73 (m, 3H), 4.68 (d, *J* = 11.5 Hz, 1H), 4.67–4.62 (m, 2H), 4.60 (d, *J* = 11.6 Hz, 1H), 4.54 (d, *J* = 7.6 Hz, 1H), 4.40 (dd, *J* = 10.8, 7.2 Hz, 2H), 3.94–3.74 (m, 9H), 3.71 (s, 3H), 3.68–3.53 (m, 4H), 3.22–3.16 (m, 1H), 3.15–3.09 (m, 1H), 3.05–2.99 (m, 1H), 2.98–2.92 (m, 1H), 2.45 (s, 4H), 2.44 (s, 3H), 2.43 (s, 3H), 2.40 (s, 3H), 0.92 (s, 9H), 0.104 (s, 3H), 0.100 (s, 3H); ^{13}C NMR (151 MHz, $CDCl_3$) δ 165.18, 165.15, 165.08, 165.05, 155.3, 151.0, 143.98, 143.96, 143.8, 143.7, 138.03, 137.97, 137.94, 137.92, 129.87, 129.74, 129.72, 129.66, 129.24, 129.14, 129.06, 129.01, 128.3, 128.24, 128.21, 128.15, 128.12, 128.06, 128.04, 128.02, 127.98, 127.45, 127.40, 127.35, 127.33, 127.04, 127.00, 126.91, 118.6, 114.3, 101.2, 100.6, 100.4, 81.9, 78.6, 78.5, 78.3, 76.9, 76.44, 76.36, 74.8, 74.1, 74.0, 73.9, 73.0, 72.4, 72.3, 71.7, 71.3, 66.2, 62.8, 62.7, 62.5, 55.5, 25.8, 21.72, 21.70, 21.66, 17.9, -4.7, -4.8; HRMS (ESI) calcd for $C_{93}H_{102}O_{22}SiNa$ $[M + Na]^+$ 1621.6530, found 1621.6529.

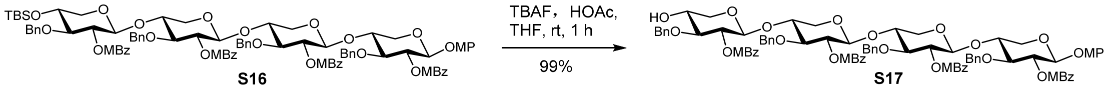

A similar procedure as that for the preparation of compound **S15A** was used to provide **S17** as a white foam (510 mg, 99%): $[\alpha]_D^{25}$ = -48.1 (*c* 1.0, $CHCl_3$); ^{1}H NMR (500 MHz, $CDCl_3$) δ 7.97–7.92 (m, 2H), 7.89–7.80 (m, 6H), 7.35–7.17 (m, 12H), 7.15–7.04 (m, 15H), 6.78–6.74 (m, 2H), 6.69–6.65 (m, 2H), 5.27 (dd, *J* = 8.5, 6.9 Hz, 1H), 5.21 (dd, *J* = 7.7, 6.1 Hz, 1H), 5.13 (dd, *J* = 8.8, 7.2 Hz, 1H), 5.07 (dd, *J* = 8.8, 7.2 Hz, 1H), 4.81 (d, *J* = 6.8 Hz, 1H), 4.79 (d, *J* = 11.3 Hz, 1H), 4.77–4.71 (m, 3H), 4.65–4.59 (m, 5H), 4.58 (d, *J* = 2.4 Hz, 1H), 4.41 (t, *J* = 7.6 Hz, 2H), 4.09 (dd, *J* = 11.8, 4.5 Hz, 1H), 3.97–3.74 (m, 8H), 3.69 (s, 3H), 3.63 (td, *J* = 8.1, 4.4 Hz, 3H), 3.55 (dd, *J* = 9.7, 7.0 Hz, 1H), 3.29 (dd, *J* = 11.8, 8.3 Hz, 1H), 3.14–3.04 (m, 2H), 2.99–2.92 (m, 1H), 2.43 (s, 3H), 2.42 (s, 4H), 2.42 (s, 3H), 2.39 (s, 3H); ^{13}C NMR (126 MHz, $CDCl_3$) δ 165.20, 165.13, 165.09, 165.07, 155.3, 151.0, 144.4, 144.0, 143.8, 143.7, 138.0, 137.9, 137.7, 129.9, 129.8, 129.7, 129.4, 129.3, 129.2, 129.1, 128.5, 128.3, 128.2, 128.09, 128.05, 128.01, 127.5, 127.4, 127.04, 126.98, 126.9, 126.7, 118.6, 114.3, 100.7, 100.60, 100.58, 100.0, 80.5, 78.9, 78.6, 78.3, 76.6, 76.4, 76.1, 74.3, 74.03, 73.95, 73.9, 72.44, 72.36, 72.1, 71.7, 69.5, 68.8, 64.2, 62.74, 62.70, 62.5, 55.6, 53.7, 31.8, 29.3, 21.73, 21.71, 21.67; HRMS (ESI) calcd for $C_{87}H_{88}O_{22}Na$ $[M + Na]^+$ 1507.5665, found 1507.5671.

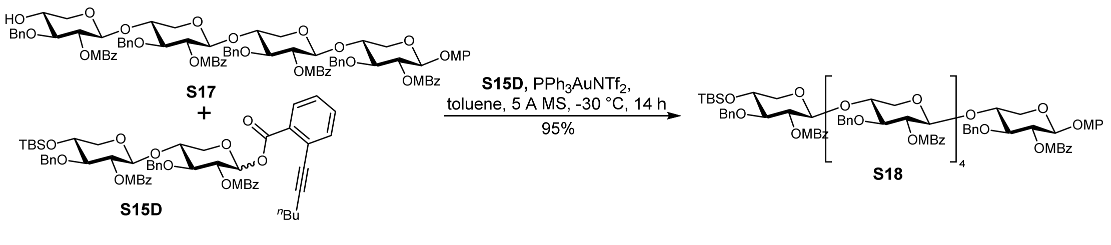

A mixture of disaccharide donor **S15D** (400 mg, 0.401 mmol) and tetrasaccharide acceptor **S17** (497 mg, 0.334 mmol) in a Schlenk flask equipped with a Teflon-coated magnetic stir bar was dried in high vacuum at 40 °C for 3 hours, and then 5Å MS (3.3 g) and anhydrous toluene (33 mL) were added. The mixture was stirred at room temperature for 15 minutes, and was then cooled to 0 °C, to which $Ph_3PAuNTf_2$ (49 mg, 0.0669 mmol) was added. The resulting mixture was stirred at -30 °C for 14 hours, to which Et_3N (100 μL) was added to quench the reaction. The resulting mixture was filtered through a pad of Celite. The filtrate was concentrated *in vacuo* and purified by silica gel column chromatography (EtOAc/petroleum ether, 1:1, R_f = 0.6) to give **S18** as a white foam (720 mg, 95%): $[\alpha]_D^{25}$ = -40.7 (*c* 1.0, $CHCl_3$); ^{1}H NMR (600 MHz, $CDCl_3$) δ 7.88–7.77 (m, 11H), 7.25–7.16 (m, 15H), 7.15–7.00 (m, 28H), 6.77–6.73 (m, 2H), 6.69–6.65 (m, 2H), 5.26 (dd, *J* = 8.5, 6.8 Hz, 1H), 5.14 (dd, *J* = 9.2, 7.6 Hz, 1H), 5.10 (dd, *J* = 8.7, 7.1 Hz, 1H), 5.07–5.00 (m, 3H), 4.81 (s, 1H), 4.80 (d, *J* = 4.6 Hz, 1H), 4.75 (d, *J* = 5.1 Hz, 1H), 4.72 (dd, *J* = 6.7, 3.4 Hz, 2H), 4.71–4.69 (m, 1H), 4.67 (d, *J* = 12.0 Hz, 1H), 4.64 (d, *J* = 9.4 Hz, 1H), 4.61 (s, 1H), 4.59 (d, *J* = 1.8 Hz, 1H), 4.57 (s, 1H), 4.56–4.52 (m, 1H), 4.51 (d, *J* = 7.6 Hz, 1H), 4.37 (t, *J* = 6.7 Hz, 2H), 4.33 (d, *J* = 7.2 Hz, 1H), 4.30 (d, *J* = 7.3 Hz, 1H), 3.92–3.69 (m, 13H), 3.68 (s, 3H), 3.61 (dt, *J* = 9.9, 8.1 Hz, 2H), 3.57–3.48 (m, 4H), 3.16 (td, *J* = 11.1, 3.0 Hz, 1H), 3.09 (tt, *J* = 10.6, 2.5 Hz, 1H), 3.03–2.97 (m, 1H), 2.95–2.86 (m, 3H), 2.45–2.34 (m, 18H), 0.90 (s, 10H), 0.08 (s, 3H), 0.07 (s, 3H); ^{13}C NMR (151 MHz, $CDCl_3$) δ 165.33, 165.30, 165.22, 165.18, 155.4, 151.2, 144.10, 144.08, 143.94, 143.85, 138.19, 138.13, 138.10, 138.07, 138.02, 137.6, 130.01, 129.96, 129.88, 129.84, 129.81, 129.4, 129.28, 129.26, 129.21, 129.18, 129.15, 128.48, 128.45, 128.42, 128.38, 128.36, 128.28, 128.26, 128.22, 128.19, 128.17, 128.15, 128.11, 128.09, 128.04, 127.9, 127.8, 127.62, 127.58, 127.53, 127.48, 127.41, 127.20, 127.17, 127.15, 127.09, 127.07, 127.05, 118.8, 118.7, 114.52, 114.49, 101.3, 100.77, 100.73, 100.68, 100.65, 100.59, 100.3, 82.1, 78.73, 78.65, 78.60, 78.4, 76.8, 76.6, 76.4, 75.0, 74.19, 74.15, 74.10, 74.06, 73.2, 72.50, 72.48, 72.44, 71.9, 71.4, 66.4, 63.0, 62.9, 62.8, 62.6, 55.7, 25.9, 21.83, 21.81, 21.79, 18.1; HRMS (ESI) calcd for $C_{133}H_{142}O_{32}SiNa$ $[M + Na]^+$ 2301.9185, found 2302.9151.

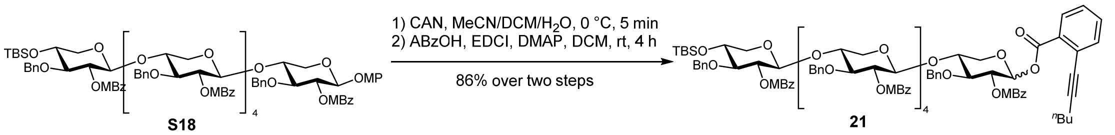

A similar procedure as that for the preparation of compound **4D** was used to provide **21** as a white foam (629 mg, α/β = 0.47:1, 86%): ^{1}H NMR (600 MHz, $CDCl_3$) δ 8.09 (dd, *J* = 7.9, 1.4 Hz, 1H), 7.93–7.65 (m, 16H), 7.54 (dd, *J* = 7.7, 1.3 Hz, 1H), 7.48 (td, *J* = 7.6, 1.4 Hz, 2H), 7.44–7.40 (m, 1H), 7.39–7.32 (m, 2H), 7.25–7.22 (m, 11H), 7.21–6.99 (m, 42H), 6.98–6.89 (m, 0.72H), 6.85–6.80 (m, 0.34H), 6.43 (d, *J* = 3.7 Hz, 0.34H), 5.82 (d, *J* = 6.7 Hz, 0.72H), 5.29 (dd, *J* = 7.9, 6.7 Hz, 0.72H), 5.20–5.09 (m, 3.32H), 5.07–5.01 (m, 3H), 4.80 (d, *J* = 11.4 Hz, 2H), 4.77–4.49 (m, 14H), 4.42 (dd, *J* = 7.3, 5.1 Hz, 0.72H), 4.40–4.36 (m, 2H), 4.32 (dd, *J* = 7.2, 3.6 Hz, 2H), 4.29 (d, *J* = 7.2 Hz, 0.72H), 3.96 (t, *J* = 9.4 Hz, 0.34H), 3.93–3.65 (m, 16H), 3.60 (t, *J* = 8.3 Hz, 2H), 3.58–3.48 (m, 6H), 3.25 (dd, *J* = 12.1, 8.4 Hz, 0.72H), 3.20–3.13 (m, 1H), 3.00 (dd, *J* = 11.9, 9.3 Hz, 1.32H), 2.96–2.88 (m, 4H), 2.51 (t, *J* = 7.1 Hz, 2H), 2.44–2.35 (m, 18H), 1.48–1.35 (m, 4H), 0.96 (t, *J* = 7.3 Hz, 4H), 0.90 (s, 9H), 0.08 (s, 3H), 0.07 (s, 3H); ^{13}C NMR (151 MHz, $CDCl_3$) δ 168.9, 165.93, 165.90, 165.5, 165.4, 165.34, 165.30, 165.24, 165.22, 165.19, 165.16, 164.1, 163.8, 144.3, 144.08, 144.06, 144.01, 143.99, 143.95, 138.5, 138.4, 138.3, 138.2, 138.12, 138.06, 138.0, 137.9, 137.6, 134.8,

134.6, 134.3, 132.5, 132.3, 132.1, 131.5, 130.9, 130.7, 130.6, 130.5, 130.4, 130.3, 130.01, 129.96, 129.93, 129.86, 129.84, 129.80, 129.78, 129.6, 129.36, 129.33, 129.30, 129.27, 129.20, 129.16, 129.13, 128.54, 128.47, 128.44, 128.37, 128.30, 128.27, 128.25, 128.20, 128.18, 128.15, 128.10, 128.06, 127.99, 127.92, 127.89, 127.86, 127.77, 127.62, 127.58, 127.56, 127.52, 127.47, 127.44, 127.40, 127.23, 127.16, 127.13, 127.08, 127.06, 127.04, 126.98, 126.85, 126.76, 126.70, 126.6, 125.7, 125.4, 124.2, 101.3, 101.0, 100.8, 100.7, 100.63, 100.57, 98.5, 97.2, 93.0, 90.6, 82.10, 82.07, 79.3, 79.08, 79.05, 78.72, 78.68, 78.60, 78.56, 78.52, 77.9, 76.8, 76.6, 76.5, 76.1, 75.1, 75.0, 74.9, 74.2, 74.12, 74.05, 73.2, 72.5, 72.43, 72.40, 72.3, 71.44, 71.41, 70.4, 66.4, 63.2, 63.0, 62.89, 62.85, 62.79, 61.9, 30.8, 30.7, 30.6, 25.9, 22.18, 22.16, 22.12, 22.11, 21.89, 21.82, 21.79, 21.76, 21.74, 19.62, 19.58, 19.55, 18.08, 18.06, 13.76, 13.74, -4.5, -4.6; HRMS (ESI) calcd for $C_{139}H_{148}O_{32}SiNa$ $[M + Na]^+$ 2380.9654, found 2380.9572.

5.2 Preparation of fully protected block-wise 32-mer 23

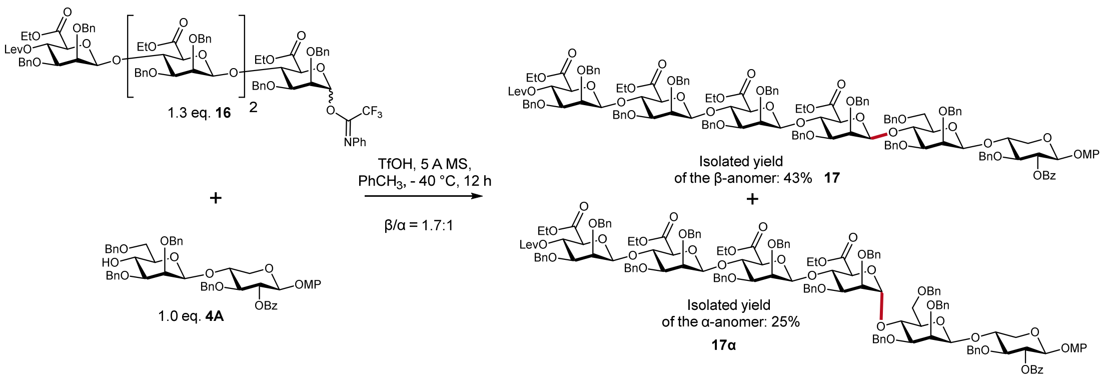

A mixture of tetrasaccharide donor **16**[10] (1.66 g, 0.910 mmol) and disaccharide acceptor **4A** (670 mg, 0.759 mmol) was azeotropically dried with toluene (2 × 10 mL), and then 5 Å molecular sieves (1.9 g) and anhydrous toluene (19 mL) were added under Ar atmosphere. The mixture was stirred at room temperature for 15 minutes, and was then cooled to -40 °C, to which TfOH (7 μL, 0.0758 mmol) was added. The mixture was stirred at -40 °C for 12 hours, to which Et_3N (1 mL) was added to quench the reaction. The resulting mixture was warmed up to room temperature, and was then filtered through a pad of Celite. The filtrate was concentrated *in vacuo* and purified by silica gel column chromatography (EtOAc/CH_2Cl_2/petroleum ether, 1:1:4, α anomer R_f = 0.3, β anomer R_f = 0.25) to give the crude α/β mixed product, which was then purified by silica gel column chromatography (EtOAc/toluene, 1:4, α anomer R_f = 0.2, β anomer R_f = 0.1) to give the pure β-anomer **17** (815 mg, 43%) and the α-anomer **17α** (484 mg, 25%). The anomeric configuration of the β-anomer and the α-anomer was verified by the coupled ^{1}H-^{13}C HSQC experiments.

17: $[\alpha]_D^{25}$ = -47.8 (*c* 1.0, $CHCl_3$); ^{1}H NMR (600 MHz, $CDCl_3$) δ 8.05–8.01 (m, 2H), 7.53–7.48 (m, 1H), 7.38–7.29 (m, 16H), 7.28–7.14 (m, 49H), 7.12–7.09 (m, 2H), 7.10–7.05 (m, 1H), 7.06–7.01 (m, 2H), 6.97–6.91 (m, 2H), 6.81–6.75 (m, 2H), 5.46–5.39 (m, 2H), 5.14 (d, J = 5.8 Hz, 1H), 4.85–4.67 (m, 17H), 4.65–4.61 (m, 4H), 4.55 (d, J = 12.4 Hz, 1H), 4.52–4.45 (m, 5H), 4.43–4.36 (m, 2H), 4.36–4.26 (m, 4H), 4.20–4.13 (m, 3H), 4.10–4.06 (m, 1H), 4.05–4.02 (m, 1H), 4.02–3.84 (m, 8H), 3.84–3.77 (m, 3H), 3.74 (s, 3H), 3.73–3.72 (m, 3H), 3.70 (d, J = 6.4 Hz, 1H), 3.69–3.62 (m, 3H), 3.62–3.59 (m, 1H), 3.56–3.47 (m, 3H), 3.44 (dd, J = 9.2, 3.0 Hz, 1H), 3.42–3.38 (m, 2H), 3.37–3.32 (m, 2H), 2.69–2.62 (m, 2H), 2.51 (t, J = 6.9 Hz, 2H), 2.13 (s, 3H), 1.10 (t, J = 7.1 Hz, 3H), 1.07–1.01 (m, 6H), 0.99 (t, J = 7.2 Hz, 3H); ^{13}C NMR (151 MHz, $CDCl_3$) δ 206.3, 171.42, 171.39, 168.27,

168.25, 168.16, 167.3, 165.4, 155.4, 151.0, 138.91, 138.86, 138.79, 138.74, 138.71, 138.6, 138.29, 138.27, 137.9, 133.1, 130.1, 130.0, 129.9, 129.8, 128.36, 128.33, 128.31, 128.25, 128.20, 128.18, 128.12, 128.09, 128.03, 127.99, 127.87, 127.82, 127.80, 127.76, 127.67, 127.64, 127.58, 127.56, 127.53, 127.49, 127.45, 127.39, 127.36, 127.34, 127.27, 127.24, 127.21, 127.17, 118.6, 114.5, 102.6, 102.52, 102.49, 101.6, 100.1, 99.8, 80.2, 80.0, 79.7, 79.6, 78.6, 77.8, 77.6, 76.1, 75.9, 75.8, 75.7, 75.6, 75.5, 75.3, 74.9, 74.8, 74.63, 74.61, 74.56, 74.48, 74.38, 74.26, 74.20, 74.15, 73.50, 73.45, 73.0, 72.9, 72.8, 72.6, 72.4, 71.5, 71.2, 69.0, 68.9, 61.8, 61.47, 61.42, 61.38, 55.6, 40.8, 37.9, 33.8, 31.9, 29.9, 29.7, 29.5, 28.4, 27.9, 23.8, 20.8, 20.6, 17.5, 17.3, 14.7, 14.1, 14.04, 14.02, 13.96, 13.89, 13.86, 7.9; HRMS (ESI) calcd for $C_{146}H_{156}O_{38}Na$ $[M + Na]^+$ 2541.0206, found 2541.0190.

17α: $[\alpha]_D^{25}$ = -43.1 (*c* 1.0, $CHCl_3$); ^1H NMR (600 MHz, $CDCl_3$) δ 8.11–8.06 (m, 2H), 7.52–7.48 (m, 1H), 7.41–7.06 (m, 72H), 6.97–6.90 (m, 2H), 6.82–6.76 (m, 2H), 5.68 (s, 1H), 5.48–5.40 (m, 2H), 5.20 (d, *J* = 5.3, 4.0 Hz, 1H), 4.85–4.69 (m, 12H), 4.67 (s, 1H), 4.64 (s, 1H), 4.61 (s, 1H), 4.59 (s, 1H), 4.56–4.34 (m, 12H), 4.32 (t, *J* = 9.3 Hz, 1H), 4.27 (d, *J* = 4.1 Hz, 1H), 4.19 (dd, *J* = 12.3, 3.9 Hz, 1H), 4.15–3.92 (m, 11H), 3.92–3.85 (m, 4H), 3.82 (d, *J* = 3.0 Hz, 1H), 3.78 (d, *J* = 3.0 Hz, 1H), 3.76–3.72 (m, 6H), 3.67 (d, *J* = 9.8 Hz, 1H), 3.62–3.50 (m, 4H), 3.50–3.43 (m, 3H), 3.41 (dd, *J* = 9.8, 2.9 Hz, 1H), 2.66 (td, *J* = 6.9, 4.9 Hz, 2H), 2.52 (t, *J* = 6.9 Hz, 2H), 2.14 (s, 3H), 1.11 (t, *J* = 7.2 Hz, 3H), 1.08–1.01 (m, 5H), 0.97 (t, *J* = 7.1 Hz, 3H); ^{13}C NMR (151 MHz, $CDCl_3$) δ 206.3, 171.4, 169.5, 168.3, 168.0, 167.3, 165.5, 155.3, 151.0, 138.9, 138.8, 138.73, 138.68, 138.5, 138.4, 138.1, 137.9, 133.0, 130.1, 130.0, 129.9, 128.5, 128.34, 128.27, 128.24, 128.20, 128.14, 128.12, 128.07, 128.04, 128.01, 127.97, 127.89, 127.84, 127.80, 127.75, 127.66, 127.61, 127.59, 127.54, 127.52, 127.48, 127.45, 127.42, 127.38, 127.35, 127.25, 127.21, 127.19, 118.6, 114.5, 102.5, 102.4, 101.7, 99.8, 98.7, 82.7, 79.6, 79.4, 78.6, 77.6, 76.3, 75.9, 75.8, 75.0, 74.9, 74.8, 74.7, 74.6, 74.4, 74.3, 74.0, 73.52, 73.45, 72.9, 72.7, 72.6, 72.5, 71.7, 71.5, 70.8, 70.6, 68.9, 61.54, 61.50, 61.48, 61.4, 55.6, 37.8, 29.9, 29.7, 27.9, 14.04, 13.99, 13.87, 13.7; HRMS (ESI) calcd for $C_{146}H_{156}O_{38}Na$ $[M + Na]^+$ 2541.0206, found 2541.0161.

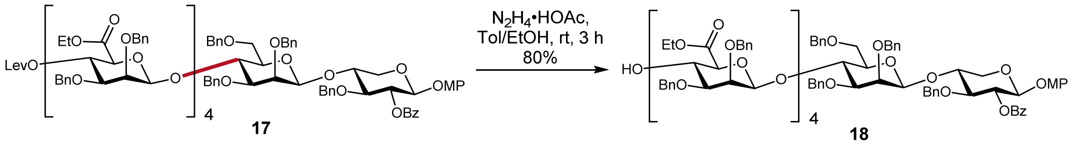

To a solution of hexasaccharide **17** (780 mg, 0.310 mmol) in the mixed solvents of toluene/EtOH (10mL / 20 mL) was added NH_2NH_2·HOAc (285 mg, 3.10 mmol). The resulting mixture was stirred at room temperature for 3 hours, and was then concentrated *in vacuo*. The residue was purified by silica gel column chromatography (EtOAc/toluene, 1:3, R_f = 0.3) to give **18** as a white foam (602 mg, 80%): $[\alpha]_D^{25}$ = -42.6 (*c* 1.0, $CHCl_3$); ^1H NMR (600 MHz, $CDCl_3$) δ 8.04–8.01 (m, 2H), 7.54–7.49 (m, 1H), 7.39–7.14 (m, 61H), 7.12–7.07 (m, 3H), 7.03 (dd, *J* = 8.0, 6.6 Hz, 2H), 6.95–6.91 (m, 2H), 6.83–6.74 (m, 2H), 5.43 (dd, *J* = 7.4, 5.8 Hz, 1H), 5.14 (d, *J* = 5.8 Hz, 1H), 4.85–4.77 (m, 8H), 4.76–4.66 (m, 8H), 4.67–4.60 (m, 5H), 4.57–4.46 (m, 8H), 4.40 (d, *J* = 12.1 Hz, 1H), 4.38–4.29 (m, 3H), 4.28 (d, *J* = 11.8 Hz, 1H), 4.21–4.13 (m, 3H), 4.12–4.03 (m, 3H), 4.02–3.95 (m, 2H), 3.95–3.84 (m, 4H), 3.84–3.79 (m, 3H), 3.78–3.72 (m, 6H), 3.71–3.68 (m, 1H), 3.67 (d, *J* = 2.8 Hz, 1H), 3.64 (d, *J* = 9.3 Hz, 1H), 3.62–3.59 (m, 1H), 3.56 (d, *J* = 9.6 Hz, 1H), 3.55–3.45 (m, 4H), 3.41 (dd, *J* = 9.3, 3.0 Hz, 1H), 3.38–3.32 (m, 2H), 3.29 (dd, *J* = 9.5, 2.9 Hz, 1H), 2.94 (s, 1H), 1.13 (t, *J* = 7.1 Hz, 3H), 1.05–1.02 (m, 6H), 0.99 (t, *J* = 7.1 Hz, 3H); ^{13}C NMR (151 MHz, $CDCl_3$) δ 169.5, 168.27, 168.24, 168.18, 165.4, 155.3, 151.0, 138.90, 138.87, 138.82, 138.78, 138.70, 138.28, 138.26, 138.0,

133.1, 130.0, 129.8, 128.42, 128.39, 128.36, 128.31, 128.29, 128.24, 128.19, 128.12, 128.07, 128.04, 128.03, 127.99, 127.96, 127.92, 127.87, 127.84, 127.80, 127.77, 127.74, 127.71, 127.67, 127.66, 127.58, 127.53, 127.47, 127.41, 127.38, 127.36, 127.28, 127.26, 127.24, 127.22, 127.15, 118.6, 114.5, 102.59, 102.57, 102.54, 101.6, 100.1, 99.8, 80.3, 80.2, 80.0, 79.7, 77.8, 77.3, 76.1, 75.9, 75.8, 75.7, 75.6, 75.5, 75.3, 75.2, 75.0, 74.82, 74.75, 74.72, 74.6, 74.53, 74.50, 74.20, 74.15, 73.5, 73.0, 72.8, 72.7, 72.41, 72.35, 72.31, 71.7, 71.2, 69.2, 68.1, 61.8, 61.6, 61.40, 61.37, 55.6, 14.02, 13.98, 13.96, 13.92; HRMS (ESI) calcd for $C_{141}H_{150}O_{36}Na$ $[M + Na]^+$ 2442.9838, found 2442.9793.

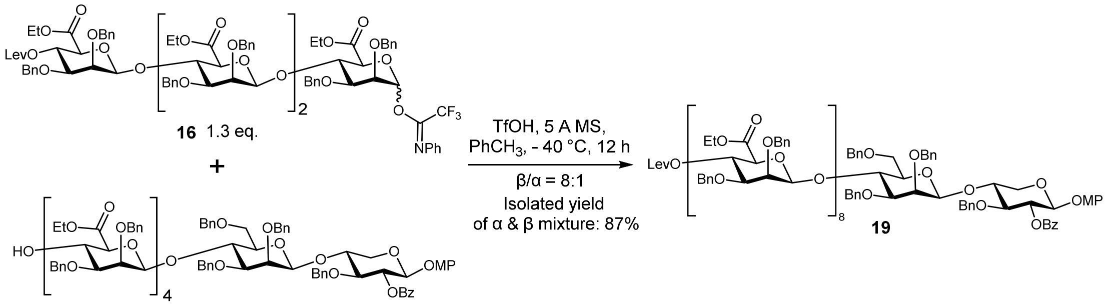

A mixture of tetrasaccharide donor **16**[10] (556 mg, 0.304 mmol) and hexasaccharide acceptor **18** (567 mg, 0.234 mmol) was azeotropically dried with toluene (2 × 10 mL), and then 5 Å molecular sieves (2.4 g) and anhydrous toluene (24 mL) were added under Ar atmosphere. The mixture was stirred at room temperature for 15 minutes, and was then cooled to -40 °C, to which TfOH (4 μL, 0.05 mmol) was added. The resulting mixture was stirred at -40 °C for 12 hours, to which Et_3N (1 mL) was added to quench the reaction. The resulting mixture was warmed up to room temperature, and was then filtered through a pad of Celite. The filtrate was concentrated *in vacuo* and purified by silica gel column chromatography (EtOAc/toluene, 1:3, R_f = 0.2) to give **19** as a mixture of α/β anomers (833 mg, 87%, α/β = 1:8): ^{1}H NMR (600 MHz, CD_3CN) δ 8.04–8.00 (m, 2H), 8.01–7.96 (m, 0.12H), 7.65–7.59 (m, 1H), 7.48 (t, J = 7.9 Hz, 2H), 7.38–7.21 (m, 97H), 7.20–7.17 (m, 5H), 7.12–6.99 (m, 5H), 6.93–6.85 (m, 2H), 6.83–6.76 (m, 2H), 5.48 (s, 0.12H), 5.28 (dd, J = 8.9, 7.3 Hz, 1H), 5.16 (d, J = 7.3 Hz, 1H), 5.12 (t, J = 9.9 Hz, 1H), 4.97 (d, J = 11.6 Hz, 1H), 4.84–4.49 (m, 51H), 4.46 (t, J = 11.3 Hz, 2H), 4.39 (d, J = 12.0 Hz, 1H), 4.19–3.87 (m, 32H), 3.85–3.80 (m, 4H), 3.79–3.71 (m, 14H), 3.71 (s, 3H), 3.66 (dd, J = 13.1, 10.3 Hz, 2H), 3.61 (dd, J = 9.5, 3.1 Hz, 1H), 3.57–3.44 (m, 10H), 3.42 (dd, J = 9.4, 3.0 Hz, 1H), 2.66–2.62 (m, 2H), 2.54–2.37 (m, 2H), 2.08 (s, 3H), 1.10–1.02 (m, 24H); ^{13}C NMR (151 MHz, $CDCl_3$) δ 206.3, 171.4, 168.25, 168.19, 168.16, 167.3, 165.4, 155.3, 151.0, 138.87, 138.85, 138.82, 138.75, 138.71, 138.67, 138.26, 138.24, 137.9, 133.1, 130.0, 129.8, 128.4, 128.32, 128.31, 128.24, 128.19, 128.16, 128.12, 128.08, 128.04, 128.03, 127.99, 127.97, 127.87, 127.83, 127.79, 127.76, 127.67, 127.63, 127.57, 127.53, 127.52, 127.46, 127.43, 127.37, 127.35, 127.31, 127.28, 127.26, 127.24, 127.21, 127.19, 127.16, 118.6, 114.5, 102.59, 102.54, 102.50, 101.6, 100.1, 99.8, 80.2, 80.0, 79.7, 79.6, 78.5, 77.7, 77.6, 76.09, 76.05, 75.8, 75.64, 75.56, 75.47, 75.3, 74.8, 74.70, 74.67, 74.59, 74.55, 74.48, 74.36, 74.19, 74.13, 73.46, 73.44, 73.0, 72.8, 72.7, 72.6, 72.4, 72.3, 71.4, 71.1, 69.2, 68.8, 61.8, 61.5, 61.39, 61.36, 55.6, 37.8, 29.9, 27.9, 14.01, 13.97, 13.95, 13.86; MALDI FT-ICR MS calcd for $C_{234}H_{252}O_{62}Na$ $[M + Na]^+$ 4076.6458, found 4076.6108.

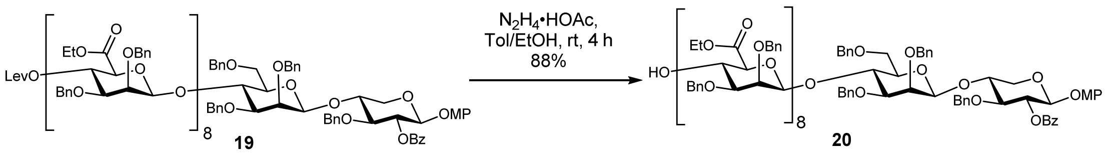

A similar procedure as that for the preparation of compound **18** was used to provide **20** as a white foam (652 mg, 88%): $[\alpha]_D^{25}$ = -50.1 (*c* 1.0, $CHCl_3$); ^1H NMR (600 MHz, CD_3CN) *δ* 8.09–8.00 (m, 2H), 7.55–7.49 (m, 1H), 7.40–7.05 (m, 124H), 7.05–7.00 (m, 2H), 6.96–6.90 (m, 2H), 6.80–6.75 (m, 2H), 5.49 (s, 0.12H), 5.43 (dd, *J* = 7.4, 5.8 Hz, 1H), 5.14 (d, *J* = 5.8 Hz, 1H), 4.85–4.76 (m, 14H), 4.76–4.59 (m, 29H), 4.57–4.46 (m, 13H), 4.44–4.24 (m, 13H), 4.21–4.13 (m, 4H), 4.13–4.03 (m, 4H), 4.03–3.85 (m, 14H), 3.85–3.78 (m, 9H), 3.77–3.65 (m, 18H), 3.64–3.45 (m, 9H), 3.44–3.32 (m, 9H), 3.29 (dd, *J* = 9.5, 2.9 Hz, 1H), 2.94 (s, 1H), 1.13 (t, *J* = 7.2 Hz, 4H), 1.05–0.97 (m, 21H); ^{13}C NMR (151 MHz, $CDCl_3$) *δ* 169.5, 168.26, 168.24, 168.20, 168.17, 165.4, 155.3, 151.0, 138.90, 138.87, 138.82, 138.78, 138.7, 138.28, 138.26, 138.0, 133.1, 130.0, 129.8, 128.42, 128.39, 128.35, 128.31, 128.29, 128.24, 128.18, 128.16, 128.11, 128.06, 128.04, 128.02, 127.99, 127.97, 127.91, 127.87, 127.84, 127.79, 127.76, 127.74, 127.70, 127.67, 127.65, 127.58, 127.52, 127.47, 127.38, 127.36, 127.32, 127.28, 127.25, 127.21, 127.19, 127.14, 118.6, 114.5, 102.6, 102.5, 101.6, 100.1, 99.8, 80.3, 80.2, 80.0, 79.76, 79.72, 79.68, 77.8, 77.18, 77.17, 76.2, 76.1, 75.82, 75.81, 75.7, 75.6, 75.5, 75.3, 75.2, 74.8, 74.7, 74.63, 74.61, 74.5, 74.2, 74.1, 73.5, 73.0, 72.84, 72.76, 72.67, 72.64, 72.58, 72.4, 72.3, 71.7, 71.2, 69.2, 68.1, 14.02, 13.96, 13.92; MALDI FT-ICR MS calcd for $C_{229}H_{246}O_{60}Na$ $[M + Na]^+$ 3978.6091, found 3978.6383.

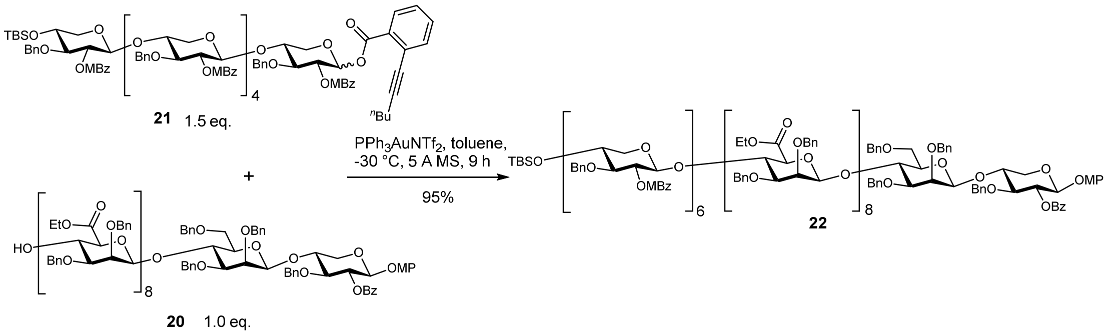

A mixture of glycosyl donor **21** (525 mg, 0.222 mmol) and acceptor **20** (587 mg, 0.148 mmol) was azeotropically dried with toluene (2 × 10 mL), and then 5Å MS (2 g) and anhydrous toluene (20 mL) were added. The mixture was stirred at room temperature for 15 minutes, and was then cooled to -30 °C, to which $Ph_3PAuNTf_2$ (55 mg, 0.0741 mmol) was added. The resulting mixture was stirred at -30 °C for 9 hours and then was slowly warmed up to room temperature, to which Et_3N (1 mL) was added to quench the reaction. The resulting mixture was filtered through a pad of Celite. The filtrate was concentrated *in vacuo* and purified by silica gel column chromatography (EtOAc/petroleum ether, 1:3, R_f= 0.4) to give **22** as a white foam (890 mg, 95%): $[\alpha]_D^{25}$ = -45.6 (*c* 1.0, $CHCl_3$); ^1H NMR (600 MHz, $CDCl_3$) *δ* 8.04–8.01 (m, 2H), 7.87–7.77 (m, 13H), 7.54–7.49 (m, 1H), 7.38–7.35 (m, 2H), 7.34–7.28 (m, 20H), 7.28–7.15 (m, 103H), 7.15–6.99 (m, 48H), 6.96–6.91 (m, 3H), 6.79–6.76 (m, 2H), 5.43 (dd, *J* = 7.4, 5.8 Hz, 1H), 5.17–5.12 (m, 2H), 5.10 (dd, *J* = 8.6, 7.1 Hz, 1H), 5.06–4.98 (m, 3H), 4.96–4.93 (m, 1H), 4.86–4.77 (m, 12H), 4.76–4.43 (m, 56H), 4.42–4.36 (m, 4H), 4.35–4.24 (m, 13H), 4.23–4.13 (m, 3H), 4.11–4.06 (m, 1H), 4.01–3.57 (m, 56H), 3.57–3.43 (m, 10H), 3.42–3.31 (m, 10H), 3.30–3.25 (m, 1H), 3.23–3.13 (m, 2H), 3.00 (dd, *J* = 11.9,

9.3 Hz, 1H), 2.96–2.78 (m, 5H), 2.40 (s, 6H), 2.39 (s, 3H), 2.38 (s, 3H), 2.37 (s, 3H), 2.36 (s, 3H), 1.06–0.93 (m, 21H), 0.91–0.88 (m, 9H), 0.77 (t, *J* = 7.1 Hz, 3H), 0.08–0.07 (m, 6H); ^{13}C NMR (151 MHz, $CDCl_3$) *δ* 168.26, 168.19, 168.17, 168.11, 167.8, 165.4, 165.17, 165.14, 165.09, 165.05, 155.4, 150.98, 144.0, 143.8, 143.4, 138.90, 138.87, 138.77, 138.70, 138.66, 138.28, 138.26, 138.12, 138.06, 138.00, 137.96, 133.1, 130.0, 129.85, 129.80, 129.73, 129.68, 129.23, 129.20, 128.9, 128.3, 128.24, 128.21, 128.19, 128.16, 128.12, 128.09, 128.05, 128.03, 127.97, 127.91, 127.87, 127.79, 127.76, 127.70, 127.67, 127.58, 127.53, 127.49, 127.47, 127.38, 127.34, 127.30, 127.28, 127.23, 127.18, 127.09, 127.05, 126.95, 126.92, 118.6, 114.5, 102.6, 102.5, 102.2, 101.6, 101.2, 100.6, 100.5, 100.2, 100.1, 99.8, 82.0, 80.2, 80.0, 79.71, 79.65, 79.3, 79.14, 79.06, 78.6, 77.8, 76.7, 76.5, 76.2, 75.8, 75.7, 75.6, 75.5, 75.3, 74.8, 74.7, 74.6, 74.5, 74.3, 74.2, 74.14, 74.10, 74.05, 73.99, 73.97, 73.5, 73.1, 73.0, 72.9, 72.8, 72.7, 72.6, 72.5, 72.40, 72.36, 72.31, 72.26, 71.3, 71.2, 69.2, 66.3, 62.8, 62.7, 62.4, 61.8, 61.39, 61.35, 61.30, 61.25, 61.16, 55.6, 25.8, 21.8, 21.69, 21.65, 21.61, 17.9, 14.0, 13.94, 13.90, 13.6, -4.66, -4.74; MALDI FT-ICR MS calcd for $C_{355}H_{380}O_{90}SiNa$ [M + Na]$^+$ 6133.4820, found 6133.3466.

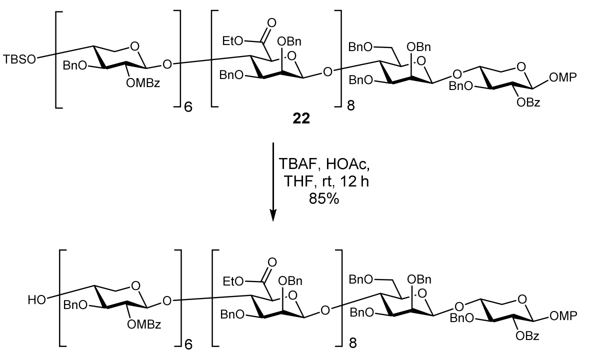

A similar procedure as that for the preparation of compound **S15A** was used to provide **22A** as a white foam (330 mg, 85%):$[\alpha]_D^{25}$ = 52.0 (*c* 2.0, $CHCl_3$); ^{1}H NMR (600 MHz, $CDCl_3$) *δ* 8.03 (d, *J* = 7.7 Hz, 2H), 7.94 (d, *J* = 8.0 Hz, 2H), 7.85 (d, *J* = 8.0 Hz, 2H), 7.83–7.76 (m, 8H), 7.51 (t, *J* = 7.5 Hz, 1H), 7.36 (t, *J* = 7.7 Hz, 2H), 7.33–6.98 (m, 146H), 6.97–6.91 (m, 2H), 6.80–6.75 (m, 2H), 5.43 (dd, *J* = 7.4, 5.8 Hz, 1H), 5.21 (dd, *J* = 7.7, 6.1 Hz, 1H), 5.16–5.11 (m, 2H), 5.08–4.98 (m, 3H), 4.97–4.93 (m, 1H), 4.85–4.12 (m, 80H), 4.12–4.06 (m, 2H), 4.02–3.84 (m, 13H), 3.84–3.57 (m, 38H), 3.56–3.42 (m, 8H), 3.41–3.32 (m, 8H), 3.31–3.26 (m, 2H), 3.20 (dd, *J* = 9.3, 2.9 Hz, 1H), 3.08 (dd, *J* = 11.3, 8.8 Hz, 1H), 2.97–2.79 (m, 4H), 2.43–2.33 (m, 18H), 1.06–0.92 (m, 21H), 0.77 (t, *J* = 7.1 Hz, 3H); ^{13}C NMR (151 MHz, $CDCl_3$) *δ* 168.30, 168.22, 168.20, 168.13, 167.9, 165.8, 165.4, 165.24, 165.17, 165.11, 165.09, 155.4, 151.0, 144.4, 144.03, 144.00, 143.96, 143.8, 143.4, 138.96, 138.92, 138.83, 138.76, 138.70, 138.59, 138.34, 138.30, 138.25, 138.17, 138.04, 138.02, 137.98, 137.8, 137.5, 133.1, 130.0, 129.9, 129.84, 129.76, 129.73, 129.46, 129.29, 129.26, 129.22, 129.1, 128.9, 128.62, 128.57, 128.55, 128.46, 128.44, 128.39, 128.34, 128.29, 128.25, 128.22, 128.19, 128.17, 128.15, 128.13, 128.09, 128.07, 128.02, 128.00, 127.98, 127.94, 127.89, 127.86, 127.83, 127.80, 127.78, 127.75, 127.71, 127.69, 127.62, 127.59, 127.57, 127.53, 127.51, 127.47, 127.42, 127.39, 127.37, 127.34, 127.30, 127.27, 127.24, 127.21, 127.18, 127.12, 127.08, 127.00, 126.96, 126.88, 126.75, 126.72, 118.7, 114.5, 102.62, 102.57, 102.54, 102.51, 102.2, 101.6, 100.6, 100.5, 100.3, 100.2, 100.1, 99.9, 80.6, 80.3, 80.1, 79.78, 79.75, 79.72, 79.3, 79.2, 79.1, 79.0, 78.66, 78.61, 78.59, 77.8, 77.2, 76.73, 76.68, 76.22, 76.17, 75.8, 75.7, 75.61, 75.55, 75.4, 74.84, 74.76, 74.72, 74.67, 74.5, 74.4, 74.3, 74.24, 74.17, 74.14, 74.10, 74.04, 74.01, 73.95, 73.90, 73.5, 73.1,

72.91, 72.87, 72.78, 72.70, 72.68, 72.65, 72.54, 72.50, 72.43, 72.40, 72.38, 72.30, 72.23, 71.2, 69.2, 68.9, 64.3, 62.83, 62.78, 62.69, 62.5, 61.9, 61.42, 61.38, 61.33, 61.28, 61.23, 61.19, 55.7, 21.76, 21.73, 21.69, 21.65, 14.06, 13.99, 13.96, 13.94, 13.6; MALDI FT-ICR MS calcd for $C_{349}H_{366}O_{90}Na$ $[M + Na]^+$ 6019.3955, found 6019.4513.

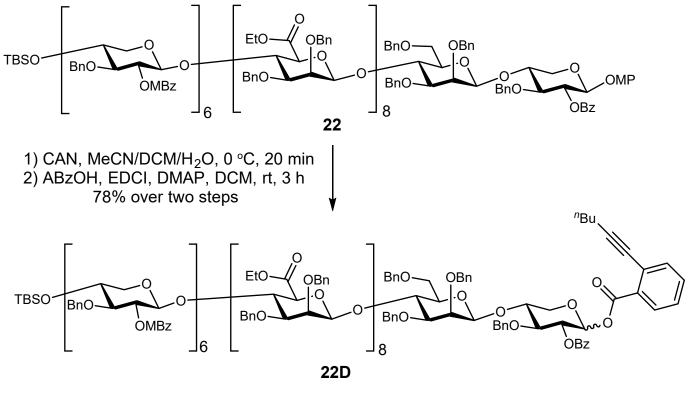

A similar procedure as that for the preparation of compound **4D** was used to provide **22D** as a white foam (373 mg, α/β = 0.67:1, 78%): ^{1}H NMR (600 MHz, $CDCl_3$) δ 8.04–7.98 (m, 1H), 7.95–7.91 (m, 1H), 7.90–7.75 (m, 15H), 7.57–7.45 (m, 2.67H), 7.42–7.38 (m, 1H), 7.38–7.28 (m, 19H), 7.28–7.15 (m, 116H), 7.15–7.09 (m, 20H), 7.09–6.93 (m, 22H), 6.57 (d, J = 3.7 Hz, 0.4H), 6.20 (d, J = 4.8 Hz, 0.6H), 5.43–5.39 (m, 0.6H), 5.36 (dd, J = 9.5, 3.8 Hz, 0.4H), 5.14 (dd, J = 9.2, 7.5 Hz, 1H), 5.10 (dd, J = 8.7, 7.1 Hz, 1H), 5.06–4.97 (m, 3H), 4.94 (dd, J = 9.0, 7.4 Hz, 1H), 4.86–4.77 (m, 9H), 4.77–4.42 (m, 58H), 4.41–4.17 (m, 20H), 4.11 (td, J = 9.2, 2.7 Hz, 1H), 4.08–4.03 (m, 0.6H), 4.02–3.31 (m, 79H), 3.28 (d, J = 9.2 Hz, 1H), 3.22–3.13 (m, 2H), 3.00 (dd, J = 11.9, 9.3 Hz, 1H), 2.95–2.78 (m, 4H), 2.46 (t, J = 7.1 Hz, 1H), 2.42–2.34 (m, 21H), 1.61–1.36 (m, 4H), 1.06–0.86 (m, 39H), 0.79–0.74 (m, 4H), 0.08–0.07 (m, 6H); ^{13}C NMR (151 MHz, $CDCl_3$) δ 168.25, 168.18, 168.16, 168.09, 167.8, 165.5, 165.4, 165.16, 165.12, 165.08, 165.04, 164.2, 163.8, 143.94, 143.91, 143.8, 143.4, 138.92, 138.88, 138.80, 138.77, 138.73, 138.66, 138.63, 138.48, 138.33, 138.30, 138.13, 138.07, 138.06, 138.01, 137.97, 137.95, 137.90, 134.8, 134.5, 133.2, 132.15, 132.12, 130.8, 130.7, 130.3, 130.02, 130.01, 129.89, 129.85, 129.82, 129.73, 129.69, 129.67, 129.51, 129.43, 129.23, 129.22, 129.18, 129.07, 129.04, 128.85, 128.42, 128.38, 128.34, 128.31, 128.29, 128.26, 128.24, 128.21, 128.18, 128.15, 128.12, 128.11, 128.08, 128.05, 128.02, 127.98, 127.96, 127.93, 127.90, 127.83, 127.76, 127.74, 127.71, 127.65, 127.58, 127.56, 127.51, 127.50, 127.47, 127.44, 127.38, 127.35, 127.33, 127.29, 127.25, 127.23, 127.20, 127.17, 127.14, 127.08, 127.04, 127.01, 126.96, 126.93, 126.85, 126.6, 125.6, 125.3, 102.59, 102.53, 102.51, 102.47, 102.2, 101.7, 101.5, 101.4, 101.2, 100.6, 100.49, 100.46, 100.2, 99.8, 99.5, 98.1, 97.1, 97.0, 92.6, 90.7, 82.0, 80.5, 80.09, 80.04, 80.00, 79.74, 79.69, 79.3, 79.20, 79.15, 79.06, 79.04, 78.62, 78.57, 78.54, 77.51, 76.52, 76.4, 76.2, 76.1, 76.0, 75.82, 75.76, 75.7, 75.6, 75.5, 75.4, 74.9, 74.8, 74.7, 74.6, 74.5, 74.3, 74.23, 74.20, 74.14, 74.12, 74.10, 74.05, 73.99, 73.97, 73.5, 73.4, 73.3, 73.08, 73.06, 72.9, 72.83, 72.75, 72.67, 72.64, 72.62, 72.57, 72.5, 72.4, 72.33, 72.31, 72.26, 72.18, 71.4, 71.3, 71.2, 69.3, 69.2, 69.1, 66.3, 62.83, 62.77, 62.65, 62.4, 61.8, 61.5, 61.39, 61.37, 61.34, 61.29, 61.24, 61.15, 30.7, 30.6, 25.8, 22.1, 22.0, 21.69, 21.67, 21.65, 21.61, 19.56, 17.9, 14.04, 14.00, 13.95, 13.92, 13.90, 13.68, 13.64, 13.55, -4.66, -4.73; MALDI FT-ICR MS calcd for $C_{361}H_{386}O_{90}SiNa$ $[M + Na]^+$ 6211.5289, found 6211.6386.

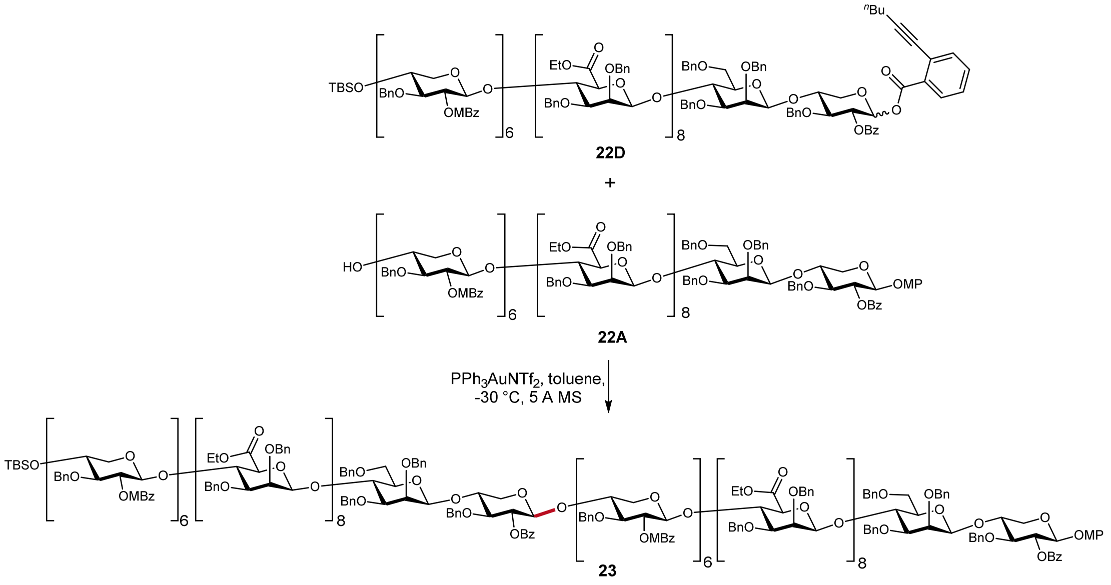

A mixture of glycosyl donor **22D** (347 mg, 0.0560 mmol) and acceptor **22A** (280 mg, 0.0467 mmol) was azeotropically dried with toluene (2 × 10 mL), and then 5Å MS (2 g) and anhydrous toluene (20 mL) were added. The mixture was stirred at room temperature for 15 minutes, and was then cooled to -30 °C, to which $Ph_3PAuNTf_2$ (26 mg, 0.0352 mmol) was added. The resulting mixture was stirred at -30 °C for 7 hours and was then slowly warmed up to room temperature, to which Et_3N (100 μL) was added to quench the reaction. The resulting mixture was filtered through a pad of Celite. The filtrate was concentrated *in vacuo* and by recycle gel permeation chromatography (GPC) to give a colorless foam, which was washed with EtOAc/petroleum ether (1:10, 5 mL) to give **23** as a white solid (458 mg, 82%): $[\alpha]_D^{25}$ = -44.8 (*c* 1.0, $CHCl_3$); ^1H NMR (600 MHz, $CDCl_3$) δ 8.05–7.97 (m, 4H), 7.88–7.76 (m, 25H), 7.55–7.50 (m, 2H), 7.42–7.35 (m, 4H), 7.34–7.28 (m, 32H), 7.28–7.15 (m, 190H), 7.15–7.01 (m, 80H), 6.99–6.91 (m, 5H), 6.83–6.72 (m, 2H), 5.43 (dd, *J* = 7.4, 5.8 Hz, 1H), 5.22–5.08 (m, 5H), 5.08–4.97 (m, 7H), 4.94 (t, *J* = 8.1 Hz, 2H), 4.86–4.07 (m, 152H), 4.06–3.13 (m, 146H), 3.10–2.77 (m, 11H), 2.48–2.32 (m, 36H), 1.07–0.91 (m, 47H), 0.90 (s, 9H), 0.79–0.73 (m, 7H), 0.09–0.07 (m, 6H); ^{13}C NMR (151 MHz, $CDCl_3$) δ 168.24, 168.16, 168.14, 168.08, 167.8, 165.4, 165.3, 165.18, 165.15, 165.11, 165.07, 165.03, 155.3, 151.0, 144.00, 143.95, 143.92, 143.8, 143.4, 139.0, 138.89, 138.85, 138.81, 138.76, 138.70, 138.68, 138.64, 138.62, 138.29, 138.27, 138.24, 138.18, 138.1, 138.03, 138.01, 137.97, 137.93, 133.3, 133.1, 129.9, 129.83, 129.78, 129.76, 129.71, 129.67, 129.3, 129.23, 129.19, 128.8, 128.45, 128.41, 128.34, 128.31, 128.29, 128.24, 128.21, 128.18, 128.15, 128.12, 128.08, 128.04, 128.01, 127.97, 127.95, 127.93, 127.90, 127.85, 127.83, 127.78, 127.75, 127.73, 127.70, 127.66, 127.64, 127.62, 127.57, 127.54, 127.51, 127.47, 127.44, 127.39, 127.36, 127.33, 127.29, 127.26, 127.23, 127.19, 127.16, 127.07, 127.03, 126.99, 126.96, 126.91, 126.87, 126.6, 118.6, 114.5, 102.6, 102.5, 102.1, 101.6, 101.2, 100.7, 100.6, 100.49, 100.46, 100.2, 100.1, 99.8, 99.6, 81.9, 80.2, 80.0, 79.72, 79.68, 79.3, 79.1, 79.0, 78.8, 78.57, 78.53, 78.49, 78.1, 77.7, 77.4, 76.5, 76.12, 76.08, 75.9, 75.7, 75.6, 75.53, 75.47, 75.3, 74.9, 74.81, 74.76, 74.67, 74.6, 74.5, 74.3, 74.2, 74.1, 74.04, 74.03, 73.98, 73.96, 73.4, 73.2, 73.02, 73.00, 72.9, 72.8, 72.7, 72.63, 72.59, 72.5, 72.37, 72.33, 72.29, 72.2, 71.7, 71.3, 71.1, 69.2, 66.2, 62.83, 62.75, 62.4, 62.0, 61.8, 61.38, 61.34, 61.29, 61.24, 61.19, 61.15, 55.6, 25.7, 21.70, 21.68, 21.66, 21.6, 17.9, 14.0, 13.94, 13.91, 13.89, 13.5, -4.68, -4.75; MALDI FT-ICR MS calcd for $C_{697}H_{738}O_{178}SiNa$ $[M + Na]^+$ 12013.8618, found 12013.7792.

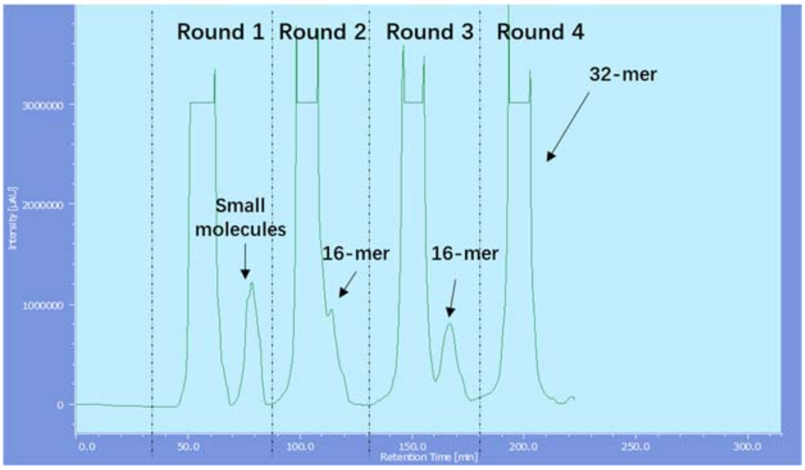

Figure S25. The preparative GPC traces of 32-mer **23** after [16+16] glycosylation.

5.3 Preparation of free block-wise 32-mer 23F

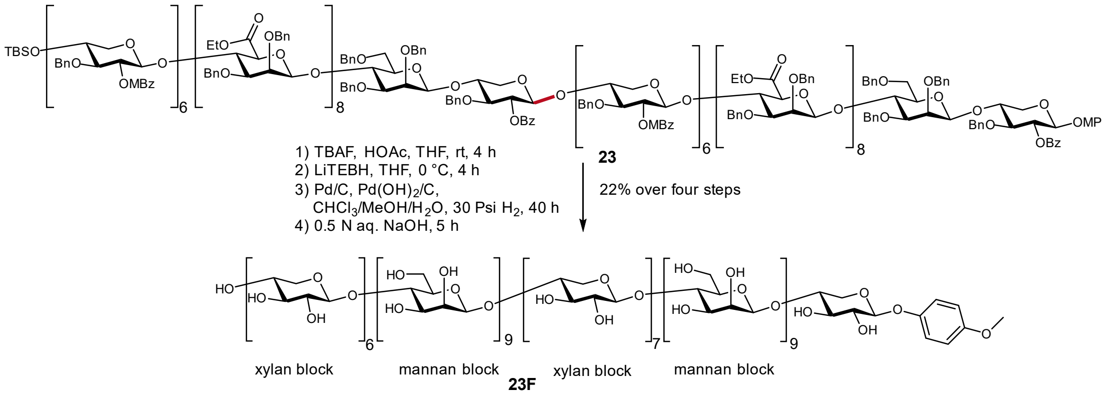

To a solution of compound **23** (217 mg, 0.0181 mmol) in anhydrous tetrahydrofuran (5 mL) were added acetic acid (2 μL, 0.0362 mmol) and tetrabutylammonium fluoride (0.181 mL, 1 M in THF) successively. The mixture was stirred at room temperature for 4 hours, and was then poured into saturated aqueous NH_4Cl. The aqueous layer was extracted with CH_2Cl_2 twice, and the combined organic layer was dried over anhydrous $NaSO_4$, filtered, and concentrated *in vacuo.* The resulting residue was purified by silica gel column chromatography (EtOAc/toluene, 1:3, R_f = 0.2-0.4) to give a colorless solid (130 mg, 60%), which was used directly for the next step without further characterization.

The above product (30 mg, 0.00253 mmol) was azeotropically dried with toluene (2 × 2 mL), and was then dried at vacuum for 2 hours. To the dried compound in anhydrous tetrahydrofuran (2 mL) was added lithium triethylborohydride (1 mL, 1 M in THF) slowly. The mixture was stirred at 0 °C for 4 hours, to which saturated aqueous NH_4Cl (2 mL) was slowly added to quench the reaction. The resulting mixture was poured into saturated aqueous NH_4Cl and the aqueous layer was extracted with EtOAc three times. The combined organic layer was dried over anhydrous Na_2SO_4, concentrated *in vacuo* and purified by gel filtration chromatography (Bio-Beads S-X1, EtOAc) to afford a colorless solid (17 mg).

To a solution of the above colorless solid (17 mg) in a mixed solvents of MeOH/$CHCl_3$/buffer (PBS, pH = 7.0) (2/4/2, 8 mL) were added 10% Pd/C (51 mg, wetted with 55% H_2O) and 20% $Pd(OH)_2$/C

(51 mg, wetted with 50% H_2O). The resulting mixture was stirred under H_2 atmosphere (30 Psi) at 30 °C for 40 hours, and was then filtered through a pad of Celite. The Celite pad was washed with MeOH/$CHCl_3$ (1:1, 6 mL), MeOH (6 mL), MeOH/H_2O (1:1, 6 mL), and H_2O (10 mL) successively. The combined filtrate was concentrated *in vacuo* and purified by gel filtration chromatography (Sephadex G-25, H_2O) to give a colorless solid. ^{1}H NMR analysis of the product indicated the existence of several remaining *p*-methylbenzoyl groups.

As a result, the above solid was treated with aqueous NaOH (0.5 N, 5 mL) at room temperature for 5 hours. The reaction was quenched by adjusting the pH of the reaction mixture to 7.0 with acetic acid. The resulting mixture was purified by gel filtration chromatography (Sephadex G-25, H_2O) to give **23F** as a white solid after lyophilization (4.5 mg, 22% over four steps).

^{1}H NMR (600 MHz, D_2O, 25 °C, δ_{HDO} = 4.79 ppm) δ 7.11–7.04 (m, 2H), 7.00–6.93 (m, 2H), 4.97 (d, J = 7.7 Hz, 1H), 4.73 (s, 16H), 4.53–4.35 (m, 13H), 4.09 (d, J = 11.9 Hz, 26H), 3.89 (d, J = 11.4 Hz, 28H), 3.79 (s, 66H), 3.53 (t, J = 9.2 Hz, 33H), 3.36 (q, J = 11.0, 10.1 Hz, 13H), 3.26 (t, J = 8.5 Hz, 14H); ^{13}C NMR (151 MHz, D_2O, 25 °C, δ_{DSS} = 0.0 ppm) δ 133.8, 131.0, 121.1, 117.8, 106.0, 104.5, 104.4, 102.9, 101.0, 80.5, 79.3, 79.0, 78.3, 77.7, 76.3, 75.4, 74.1, 72.9, 72.6, 71.9, 67.9, 65.7, 63.2, 58.5; MALDI FT-ICR MS calcd for $C_{185}H_{300}O_{148}Na$ $[M + Na]^+$ 4912.5841, found 4912.5379.

5.4 Spectral comparison of synthetic block-wise 32-mer 23F with the natural xylomannan

Table S4. ^{13}C NMR comparison between the synthetic block-wise32-mer **23F** and the natural xylomannan reported by Walters et al.

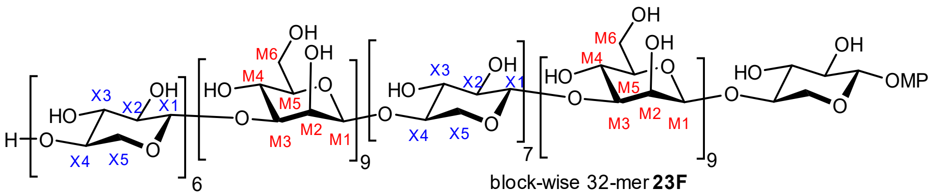

Position	X1	X2	X3	X4	X5	M1	M2	M3	M4	M5	M6
32-mer **23F** (ppm)	101.8	72.8	73.8	76.7	63.1	100.3	70.0	71.5	76.4	75.1	60.6
Walters' report (ppm)[5]	101.7	72.8	73.8	76.6	63.0	100.2	70.1	71.6	76.6	75.1	60.6
Discrepancy (ppm)	0.1	0.0	0.0	0.1	0.1	0.1	-0.1	-0.1	-0.2	0.0	0.0

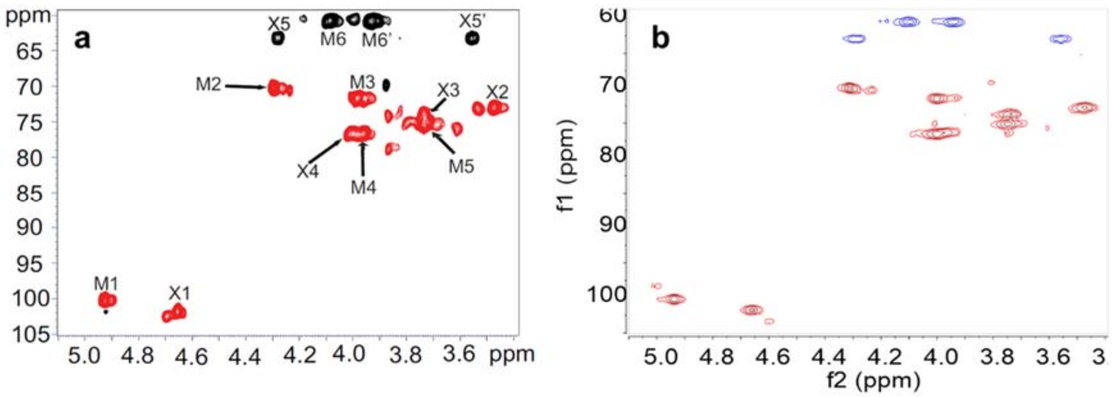

Figure S26. Comparison of the HSQC spectra of the synthetic 32-mer **23F** and the isolated xylomannan[5], where shows no deviations. **a.** HSQC spectrum of the isolated xylomannan[5]. **b.** HSQC spectrum of 32-mer **23F**. Test conditions: 20 mM Na_3PO_4 buffered D_2O, 40 °C.

6. Analysis of the NMR and mass spectroscopic data of synthetic glycans

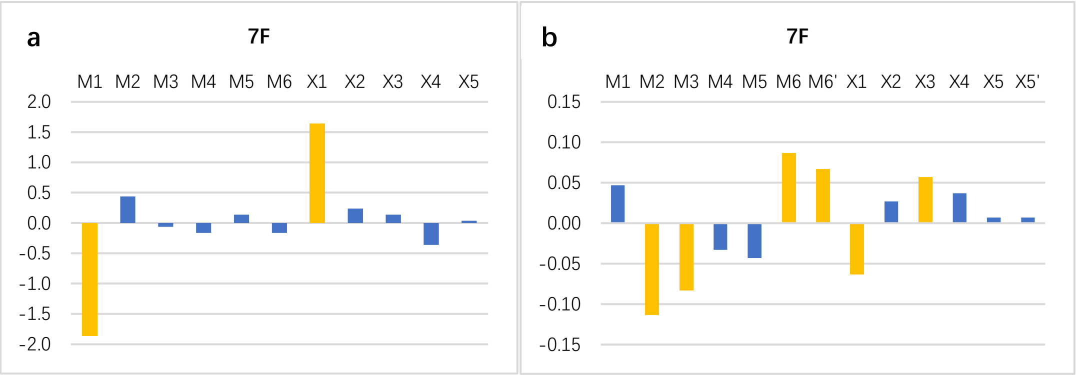

Figure S27. Discrepancies of the NMR spectroscopic data of synthetic 16-mer **7F** from those of the natural xylomannan.[5] **a**, Deviations of the ^{13}C NMR spectroscopic data. **b**, Deviations of the ^{1}H NMR spectroscopic data. Differences larger than 0.5 ppm in the ^{13}C NMR data and differences larger than 0.05 ppm in the ^{1}H NMR data are marked in yellow.

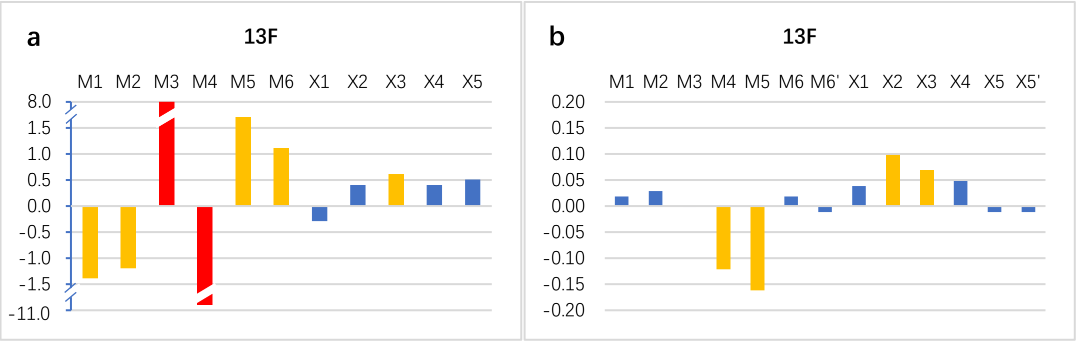

Figure S28. Discrepancies of the NMR spectroscopic data of synthetic 16-mer **13F** from those of the natural xylomannan.[5] **a**, Deviations of the ^{13}C NMR spectroscopic data. **b**, Deviations of the ^{1}H NMR spectroscopic data. Differences larger than 0.5 ppm in the ^{13}C NMR data and differences larger than 0.05 ppm in the ^{1}H NMR data are marked in yellow. Differences larger than 5.0 ppm in the ^{13}C NMR data are marked in red.

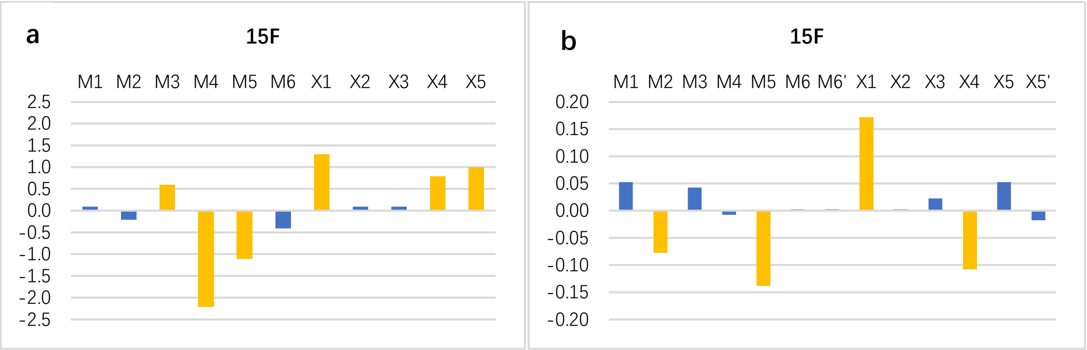

Figure S29. Discrepancies of the NMR spectroscopic data of synthetic 16-mer **15F** from those of the natural xylomannan.[5] **a**, Deviations of the ^{13}C NMR spectroscopic data. **b**, Deviations of the ^{1}H NMR spectroscopic data. Differences larger than 0.5 ppm in the ^{13}C NMR data and differences larger than 0.05 ppm in the ^{1}H NMR data are marked in yellow.

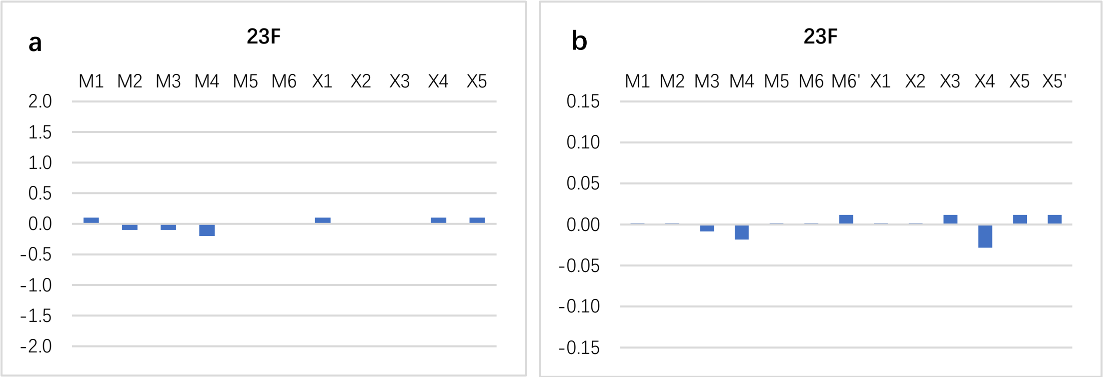

Figure S30. Discrepancies of the NMR spectroscopic data of synthetic 32-mer **23F** from those of the natural xylomannan.[5] **a**, Deviations of the ^{13}C NMR spectroscopic data. **b**, Deviations of the ^{1}H NMR spectroscopic data.

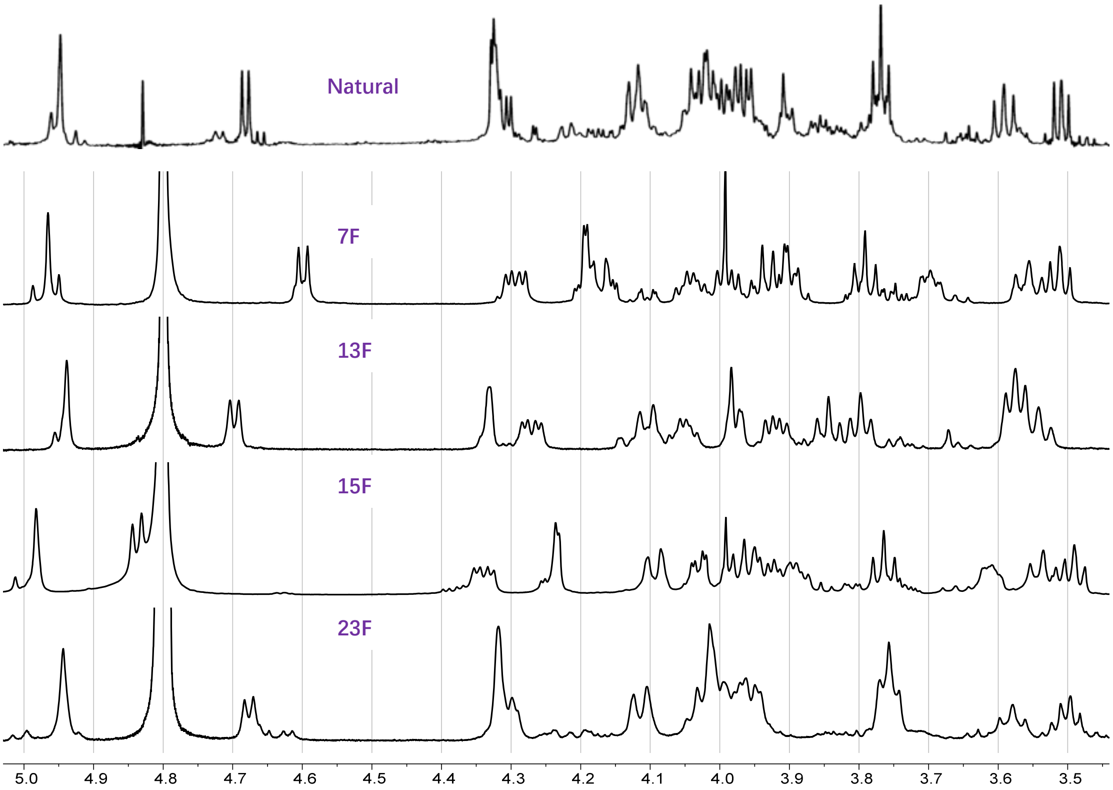

Figure S31. Overlaid ^{1}H NMR spectra of natural xylomannan,[5] and synthetic glycans **7F**, **13F**, **15F** and **23F**.

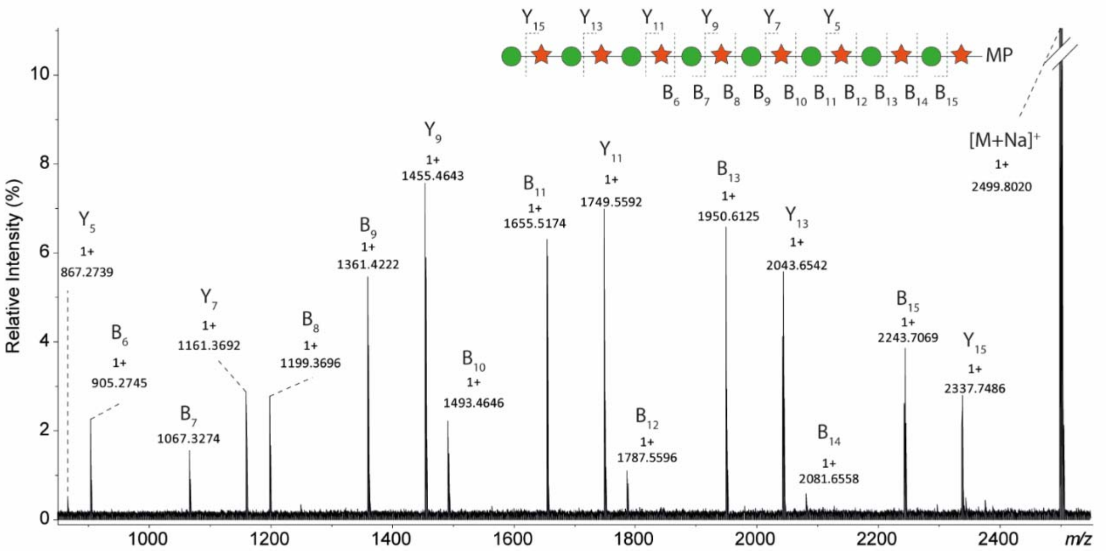

Figure S32. MALDI-CID mass spectrum of 16-mer **7F**. Green circle denotes mannose residue. Orange star denotes xylose residue.

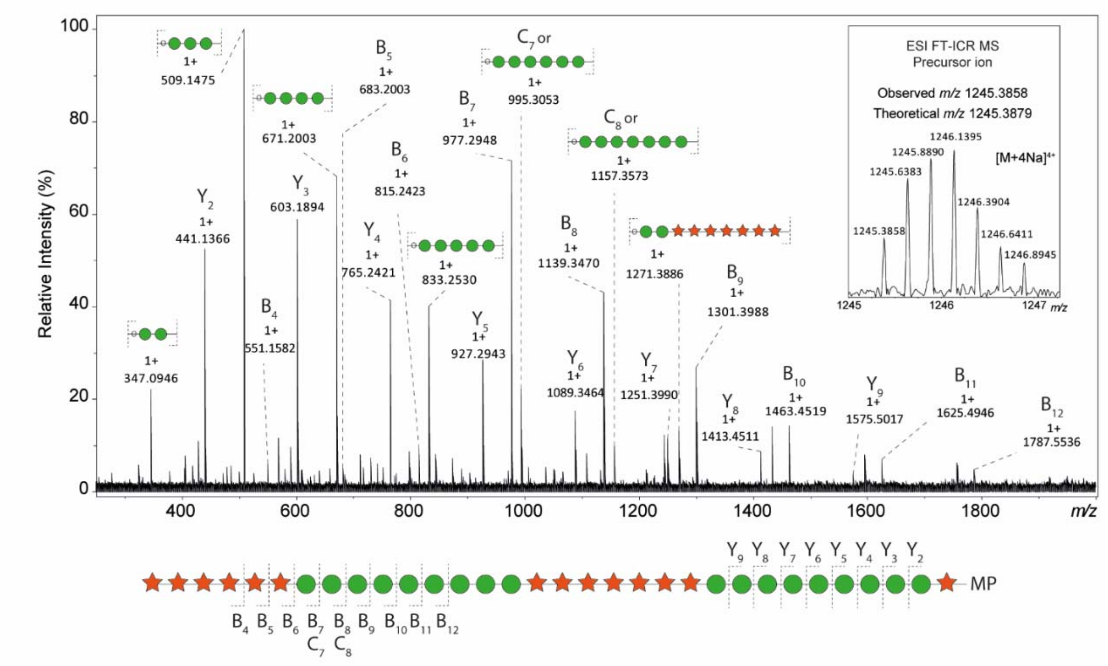

Figure S33. ESI-CID mass spectrum of the block-wise xylomannan **23F**. Green circle denotes mannose residue. Orange star denotes xylose residue.

7. Antifreeze activity measurement

Nanoliter Osmometer Experiments. A reported procedure was followed[11]. Sub-microliter volume of the glycan dispersion in H_2O (10 mg mL^{-1}) was injected using a microsyringe into a temperature-controlled sample holder full of silicone oil. The glycan dispersion immerged in oil was quickly frozen, and was then slowly warmed to the melting temperature. Once a single ice crystal appeared

it was kept for about 20 s and the temperature was recorded as melting temperature (T_m). Then the temperature was slowly decreased to a specific value (T_f), at which ice starts to grow. Supercooling temperature (ΔT) is obtained by subtracting T_f from T_m. This procedure was recorded by an optical CCD.

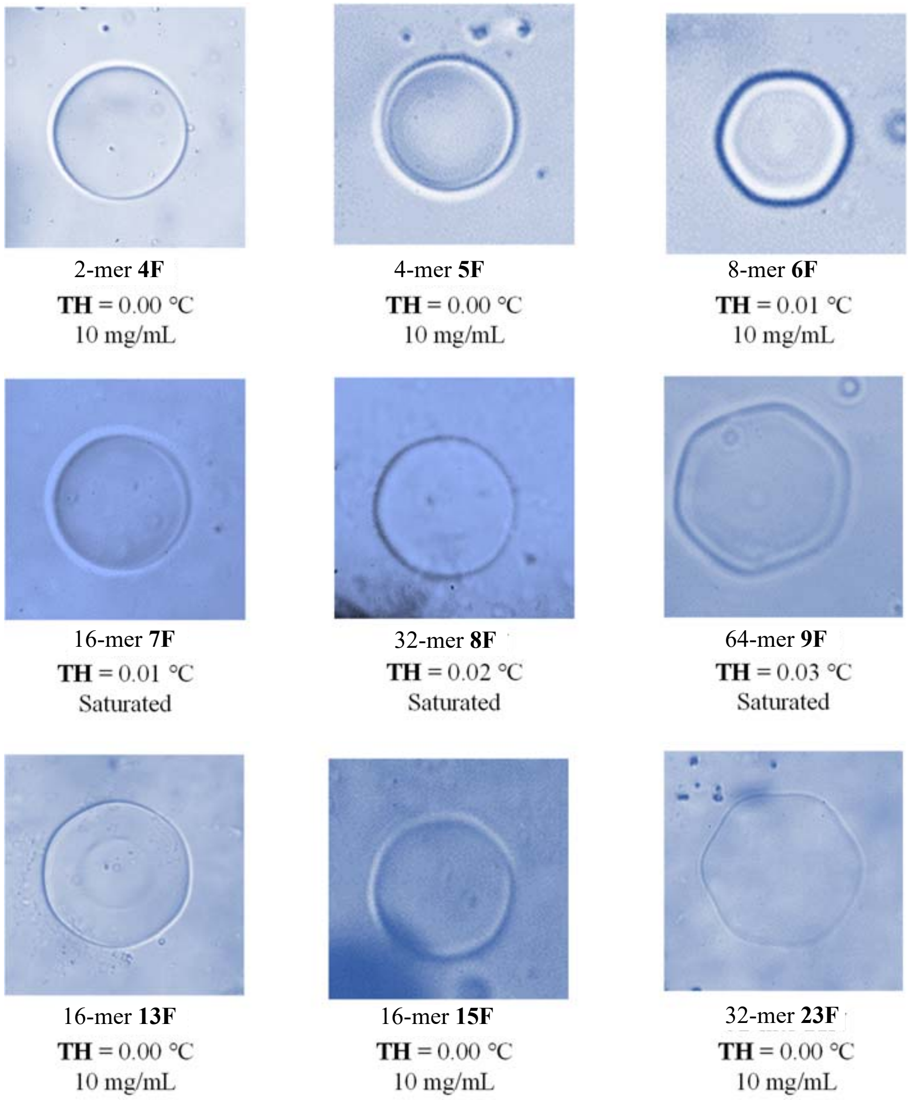

Figure S34. Optical images exhibit the shape of ice crystals with the addition of different xylomannans in pure liquid water. Because the elongated xylomannans (typically exceeding 16-mer) show low water solubility, thermal hysteresis (TH) was measured at their saturated concentrations.

References

1. Worm-Leonhard, K., Larsen, K. & Jensen, K. J. 4,6-*O*-Benzylidene directed β-mannosylation without intermediate triflate formation? Comparison of trichloroacetimidate and DISAL donors in microwave-promoted glycosylations under neutral conditions. *J. Carbohydr. Chem.* **26**, 349-368 (2007).
2. Suhr, R. & Thiem, J. Studies towards the synthesis of the β-D-Xyl-(1→3)-L-Ara disaccharide moiety of OSW-1 from *Ornithogalum saundersiae*. *J. Carbohydr. Chem.* **23**, 261-276 (2006).
3. Liu, X., Wada, R., Boonyarattanakalin, S., Castagner, B. & Seeberger, P. H. Automated synthesis of lipomannan backbone α(1-6) oligomannoside via glycosyl phosphates: glycosyl tricyclic orthoesters revisited. *Chem. Commun.* 3510 (2008).
4. Crich, D. & Rahaman, M. Y. Synthesis and structural verification of the xylomannan antifreeze substance from the freeze-tolerant Alaskan beetle *Upis ceramboides*. *J. Org. Chem.* **76**, 8611-8620 (2011).
5. Walters, K. R., Serianni, A. S., Sformo, T., Barnes, B. M. & Duman, J. G. A nonprotein thermal hysteresis-producing xylomannan antifreeze in the freeze-tolerant Alaskan beetle Upis ceramboides. *Proc. Natl. Acad. Sci. U. S. A.* **106**, 20210-20215 (2009).
6. Ponnapalli, K. K., Ho, Y.-C., Tseng, M.-C., Sekhar Vasamsetti, B. V. & Shie, J.-J. One-pot glycosylation strategy assisted by ion mobility–mass spectrometry analysis toward the synthesis of *N*-linked oligosaccharides. *J. Org. Chem.* **87**, 5339-5357 (2022).
7. Borowiecka, J. & Michalska, M. Novel stereoselective synthesis of glycosyl-*O*-acyl esters via peracetylglycosyl phosphorothioates, -selenoates and -dithioates as glycosyl donors. *Synthesis* **1996**, 858-862 (1996).
8. Sugawara, T. *et al.* Synthesis of ω-(methoxycarbonyl)alkyl and 9-(methoxycarbonyl)-3,6-dioxanonyl glycopyranosides for the preparation of carbohydrate-protein conjugates. *Carbohydr. Res.* **230**, 117-149 (1992).
9. Takeo, K., Ohguchi, Y., Hasegawa, R. & Kitamura, S. Synthesis of (1→4)-*β*-D-xylo-oligosaccharides of dp 4–10 by a blockwise approach. *Carbohydr. Res.* **278**, 301-313 (1995).
10. Pan, D., Zhang, L., Hua, Q. & Yang, Y. Highly convergent synthesis of a β-mannuronic acid alginate hexadecasaccharide. *Org. Biomol. Chem.* **17**, 6174-6177 (2019).
11. Tam, R. Y. *et al.* Solution conformation of *C*-Linked antifreeze glycoprotein analogues and modulation of ice recrystallization. *J. Am. Chem. Soc.* **131**, 15745-15753 (2009).

^{1}H NMR, ^{13}C NMR, 2D NMR and Mass spectra

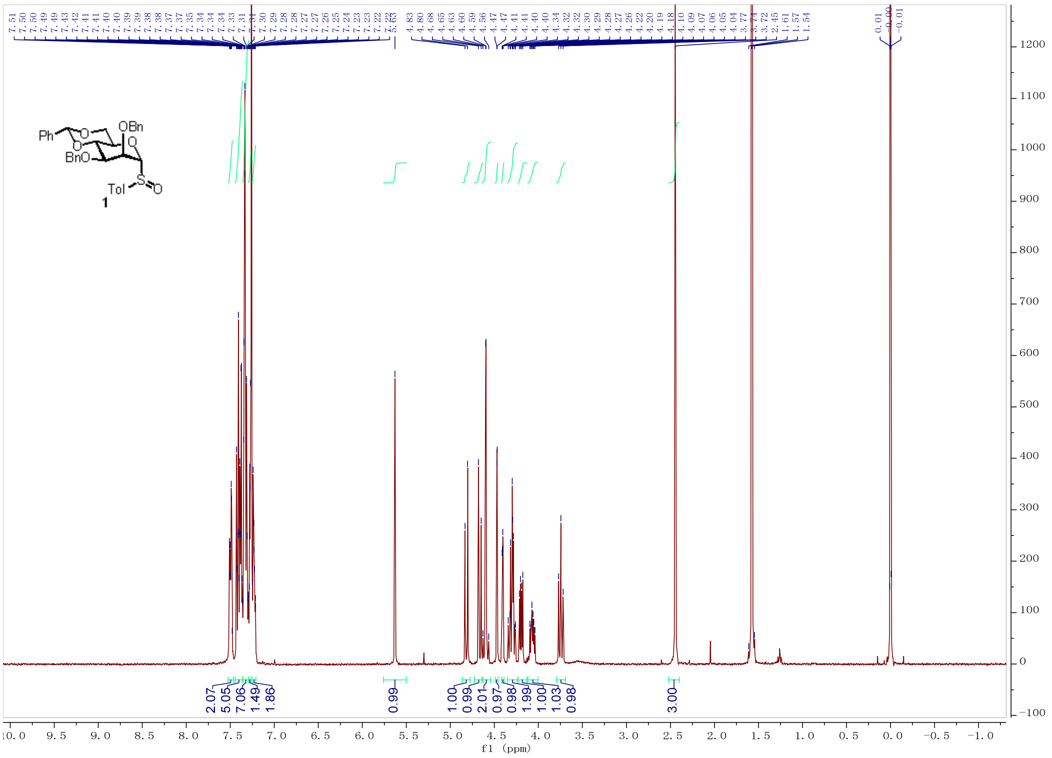

^{1}H NMR spectrum of 1 (400 MHz, $CDCl_3$, 25 °C)

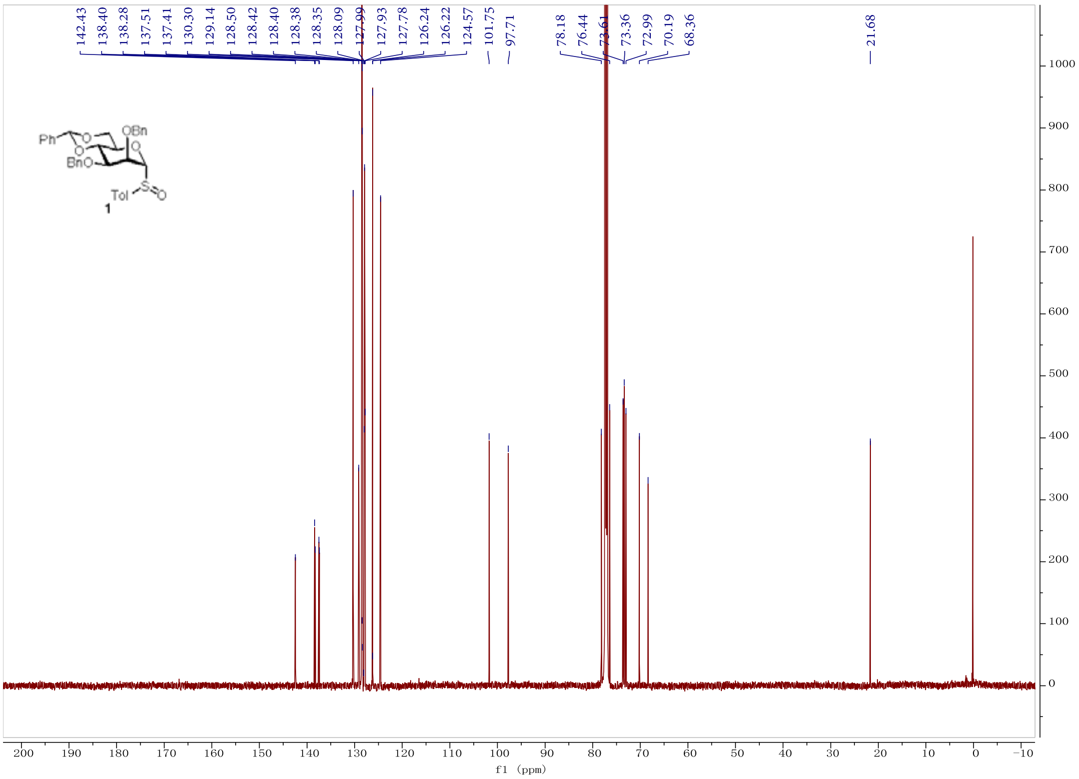

^{13}C NMR spectrum of 1 (151 MHz, $CDCl_3$, 25 °C)

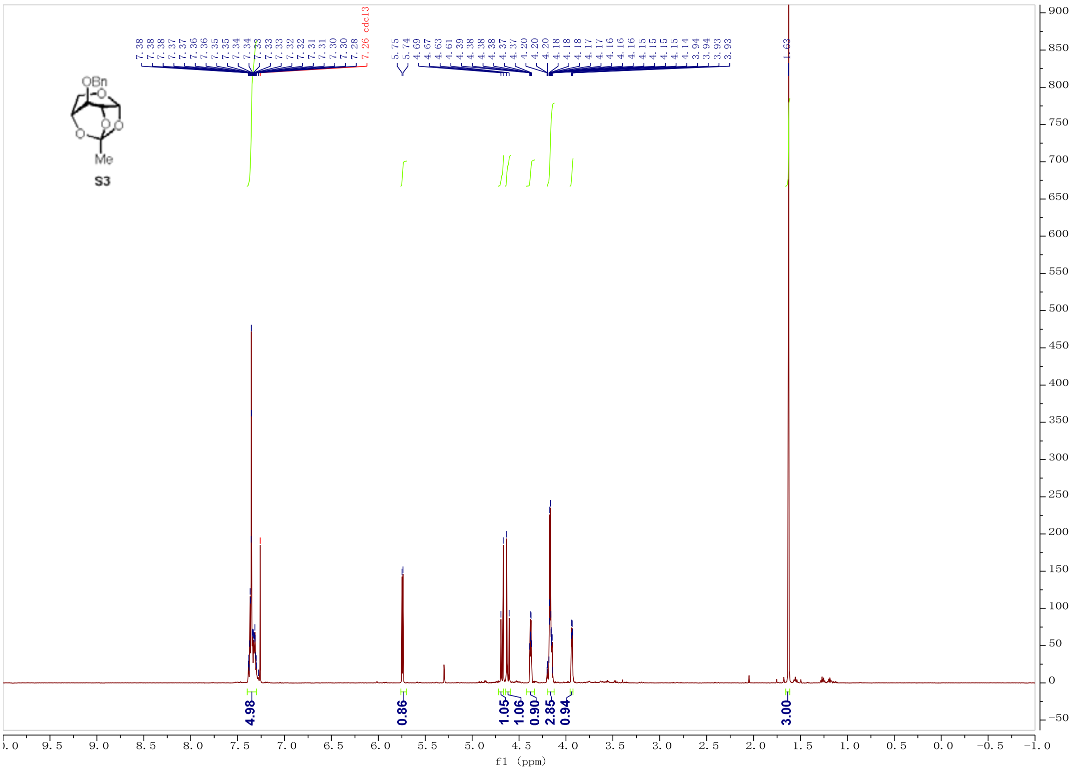

^{1}H NMR spectrum of S3 (500 MHz, $CDCl_3$, 25 °C)

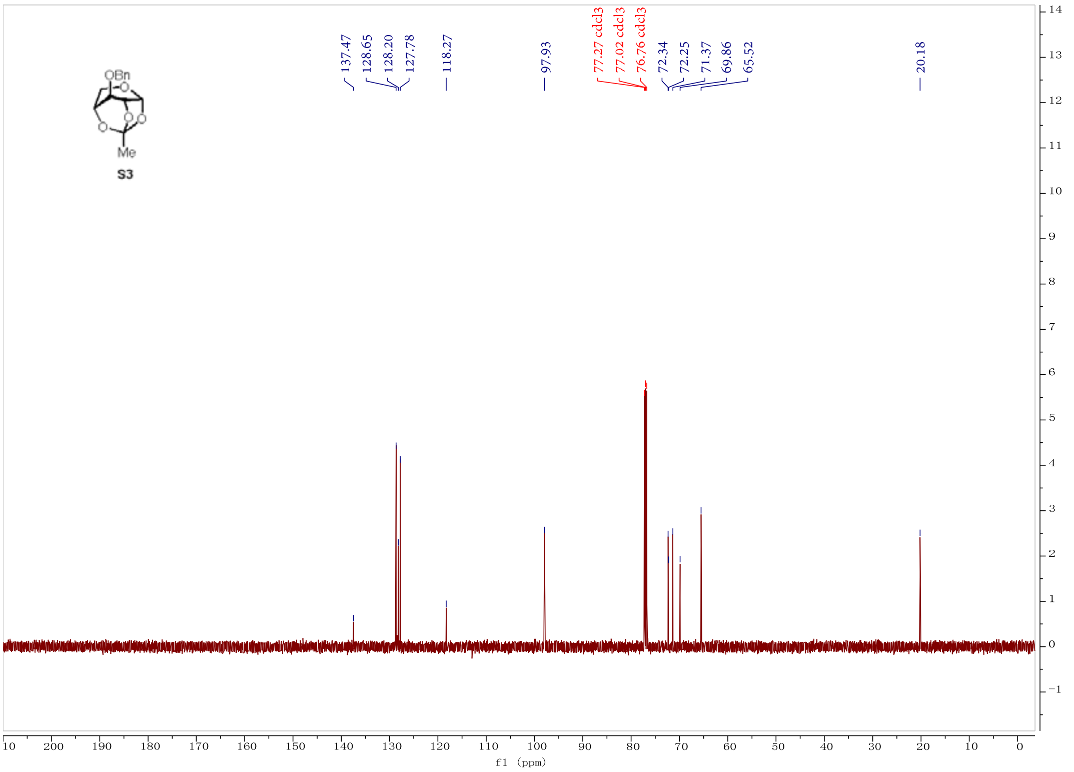

^{13}C NMR spectrum of S3 (126 MHz, $CDCl_3$, 25 °C)

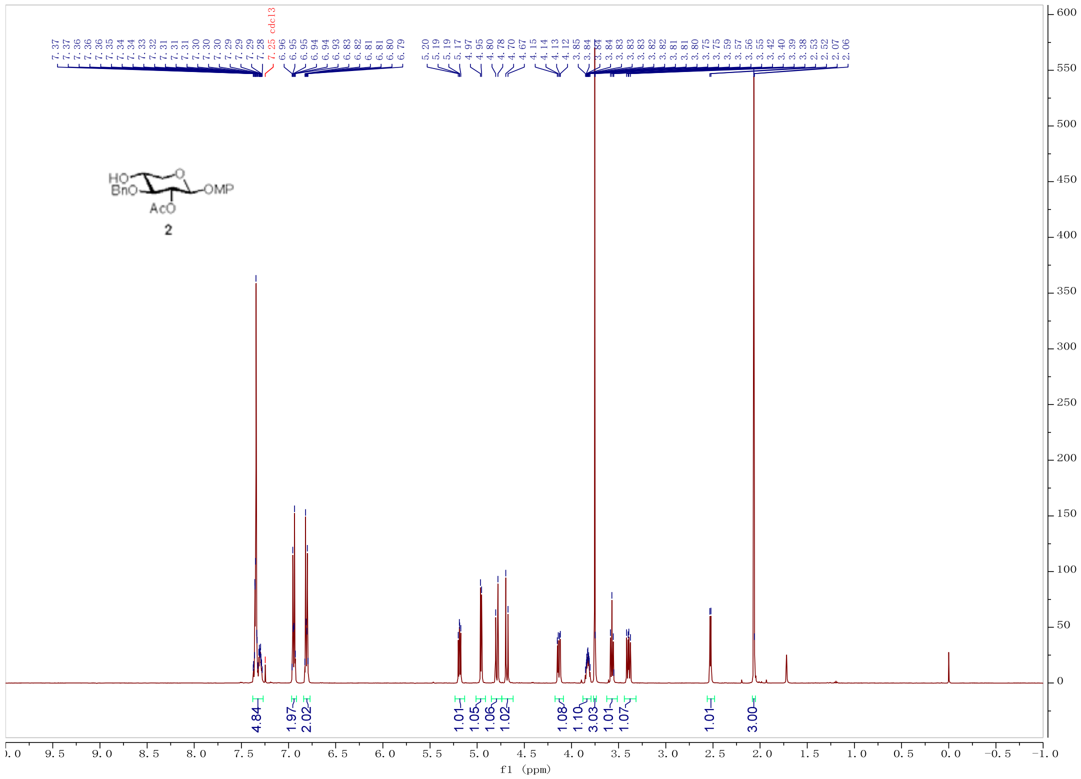

¹H NMR spectrum of 2 (500 MHz, $CDCl_3$, 25 °C)

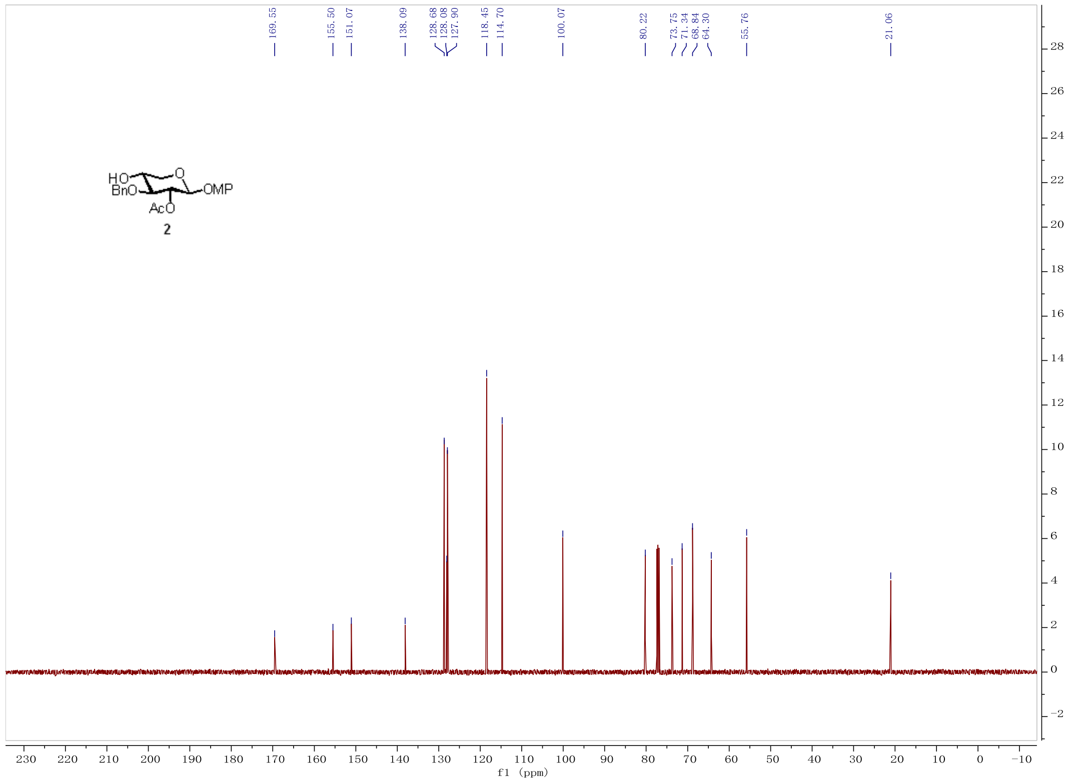

¹³C NMR spectrum of 2 (126 MHz, $CDCl_3$, 25 °C)

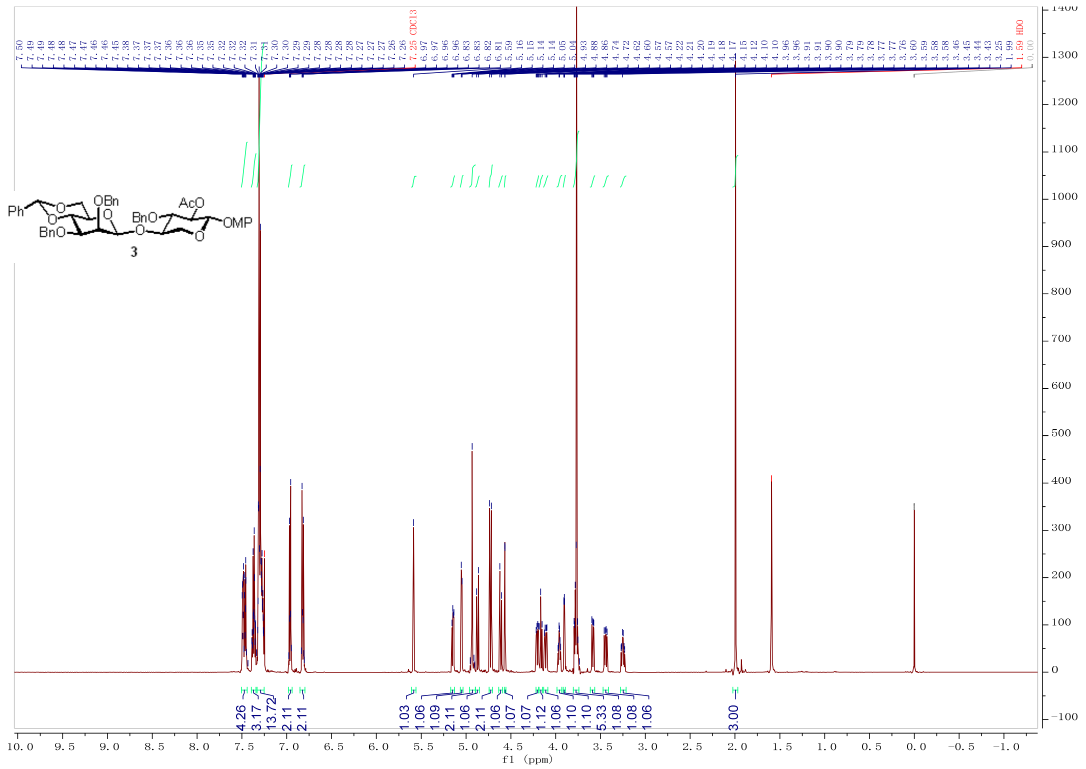

^{1}H NMR spectrum of 3 (600 MHz, $CDCl_3$, 25 °C)

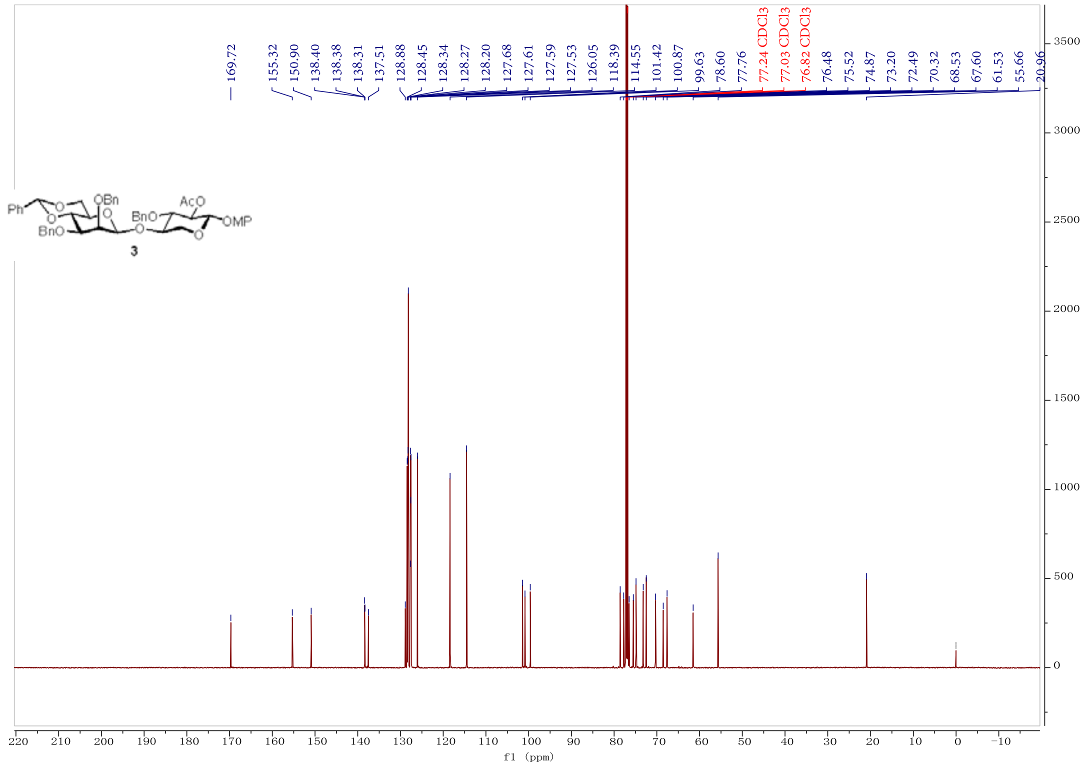

^{13}C NMR spectrum of 3 (151 MHz, $CDCl_3$, 25 °C)

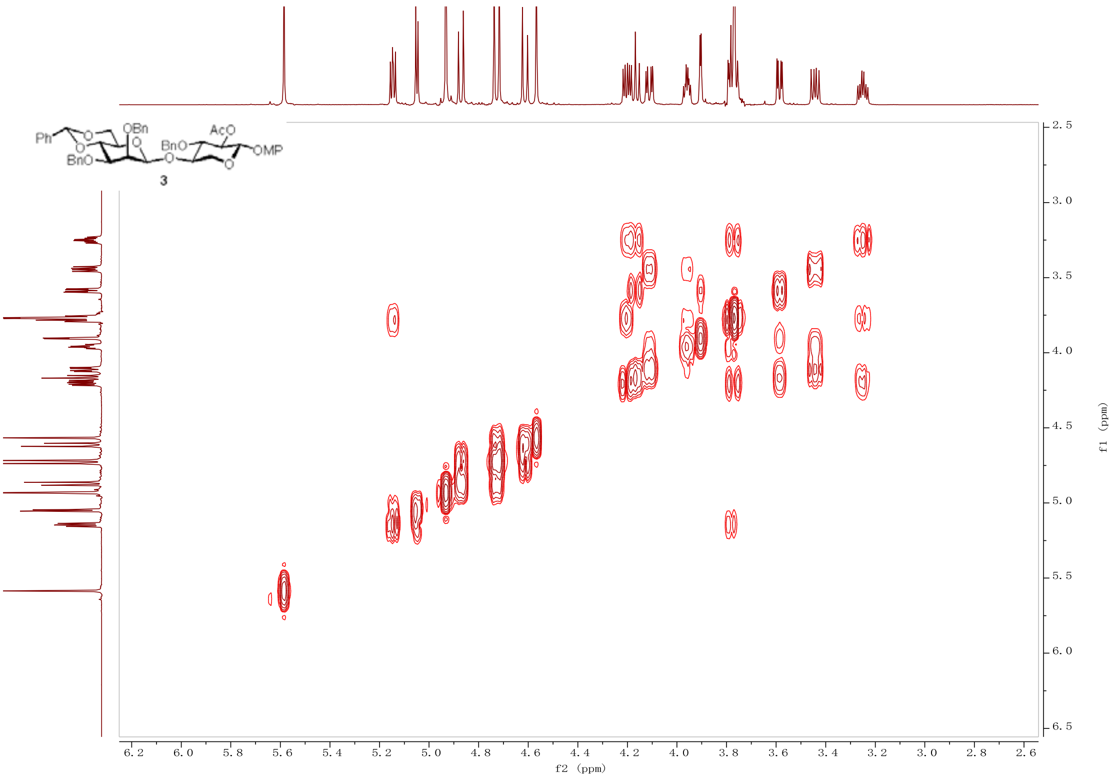

^{1}H-^{1}H gCOSY spectrum of 3 (600 MHz, $CDCl_3$, 25 °C)

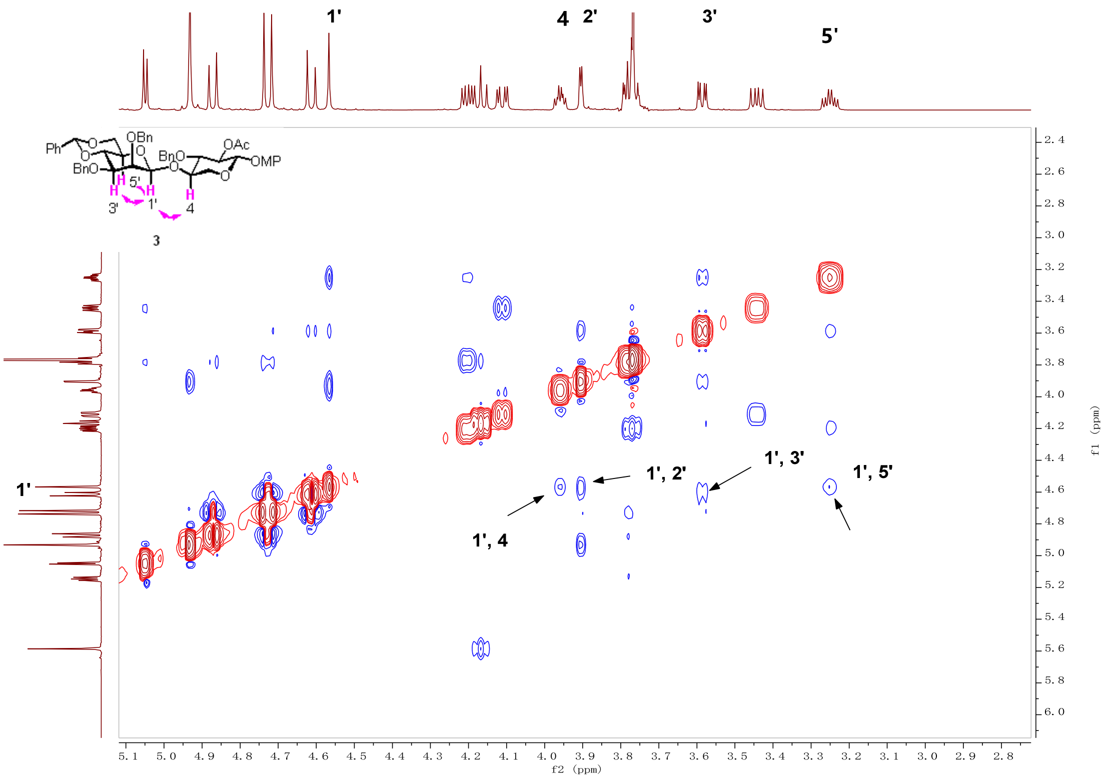

^{1}H-^{1}H NOESY spectrum of 3 (600 MHz, $CDCl_3$, 25 °C)

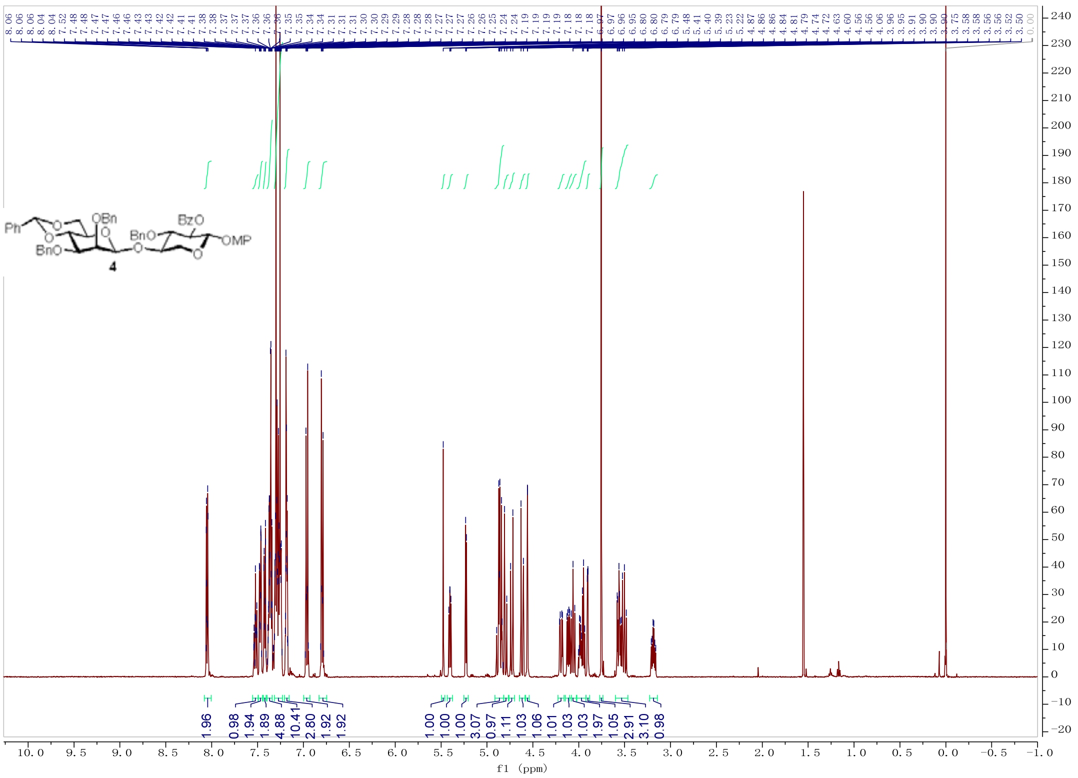

^{1}H NMR spectrum of 4 (500 MHz, $CDCl_3$, 25 °C)

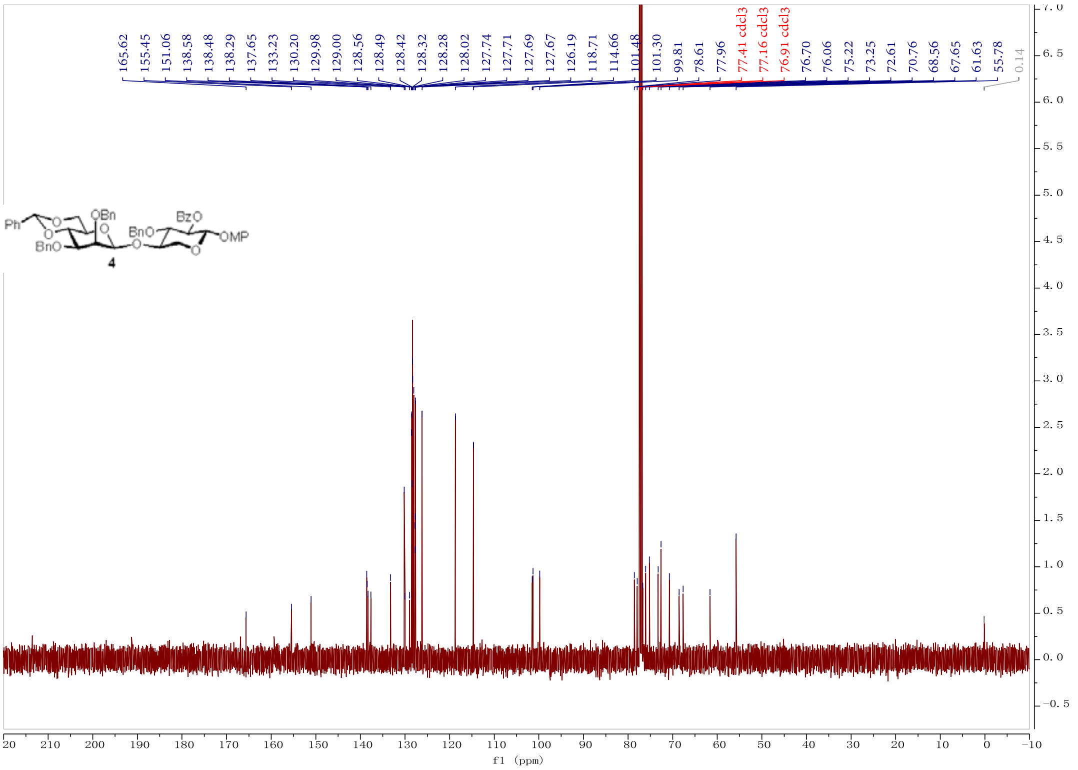

^{13}C NMR spectrum of 4 (126 MHz, $CDCl_3$, 25 °C)

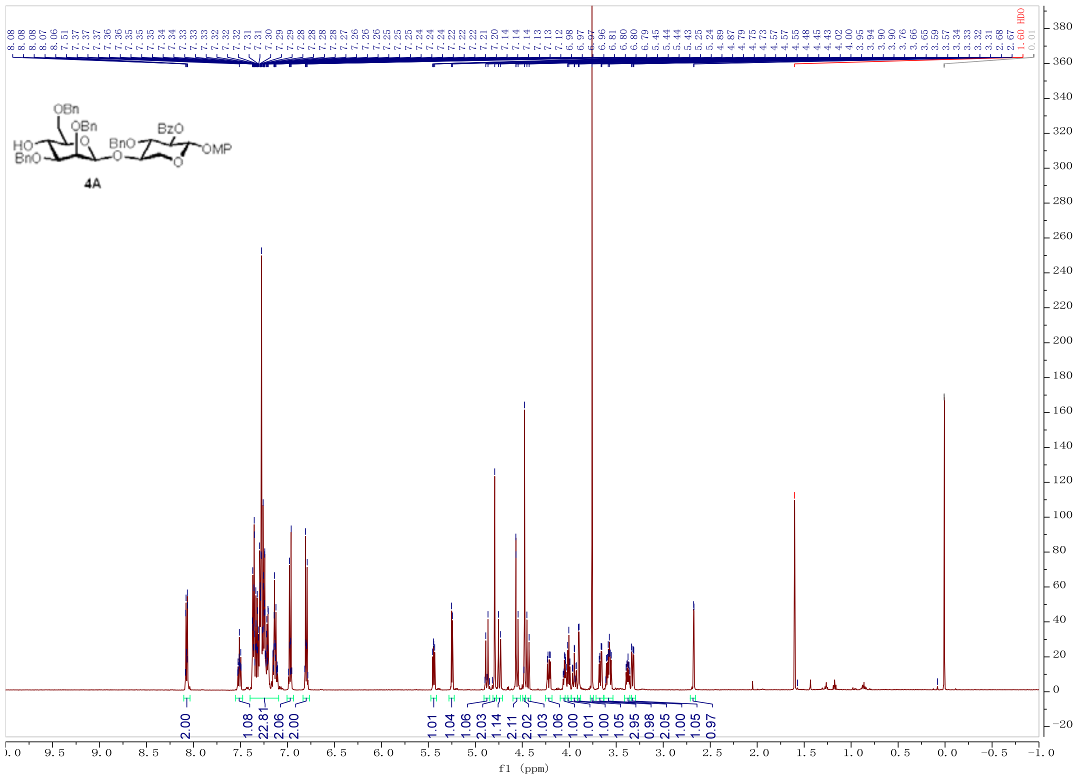

^{1}H NMR spectrum of 4A (500 MHz, $CDCl_3$, 25 °C)

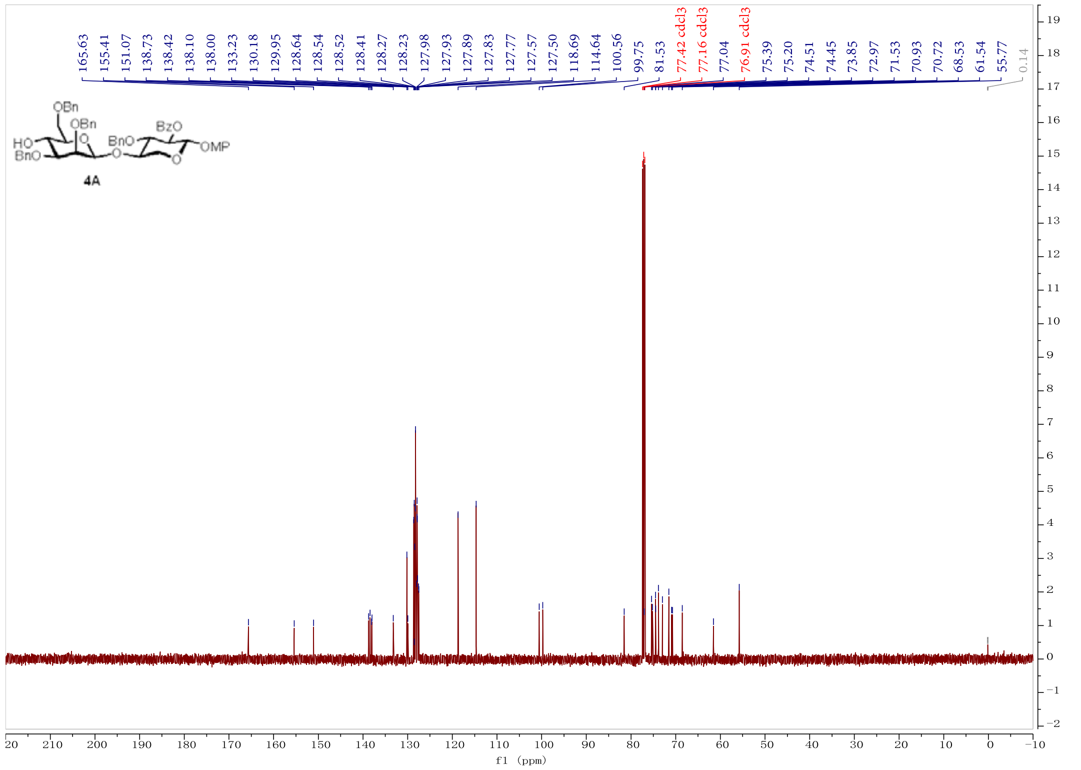

^{13}C NMR spectrum of 4A (126 MHz, $CDCl_3$, 25 °C)

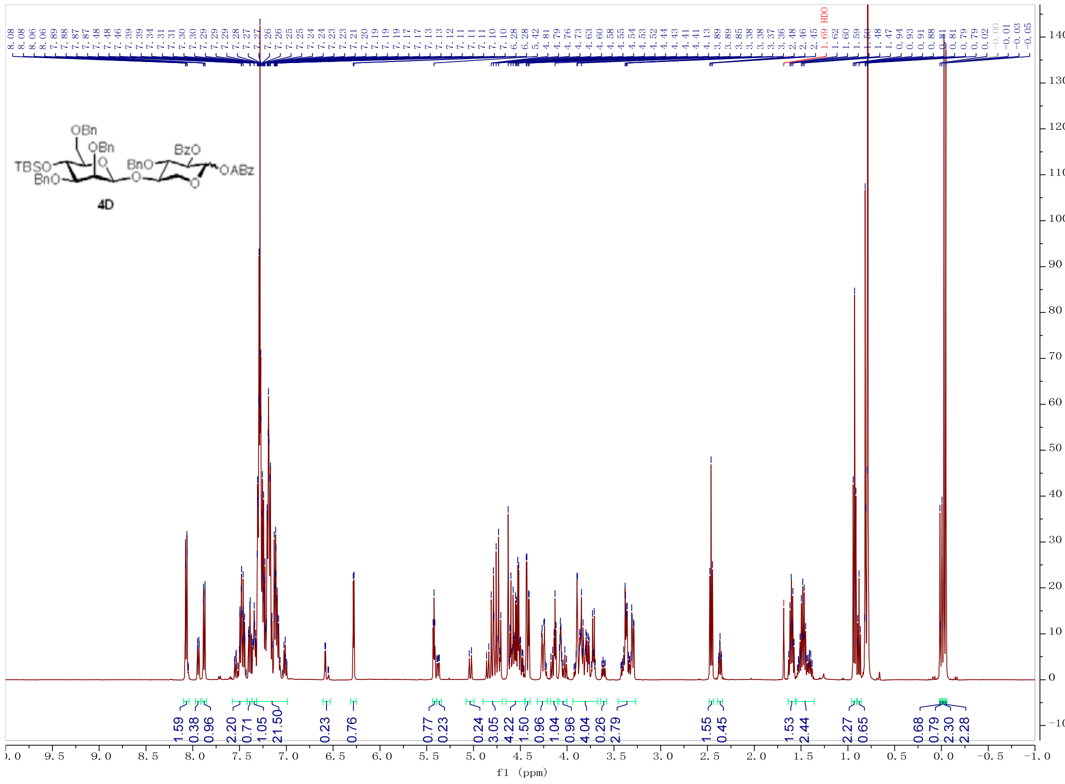

^{1}H NMR spectrum of 4D (500 MHz, $CDCl_3$, 25 °C)

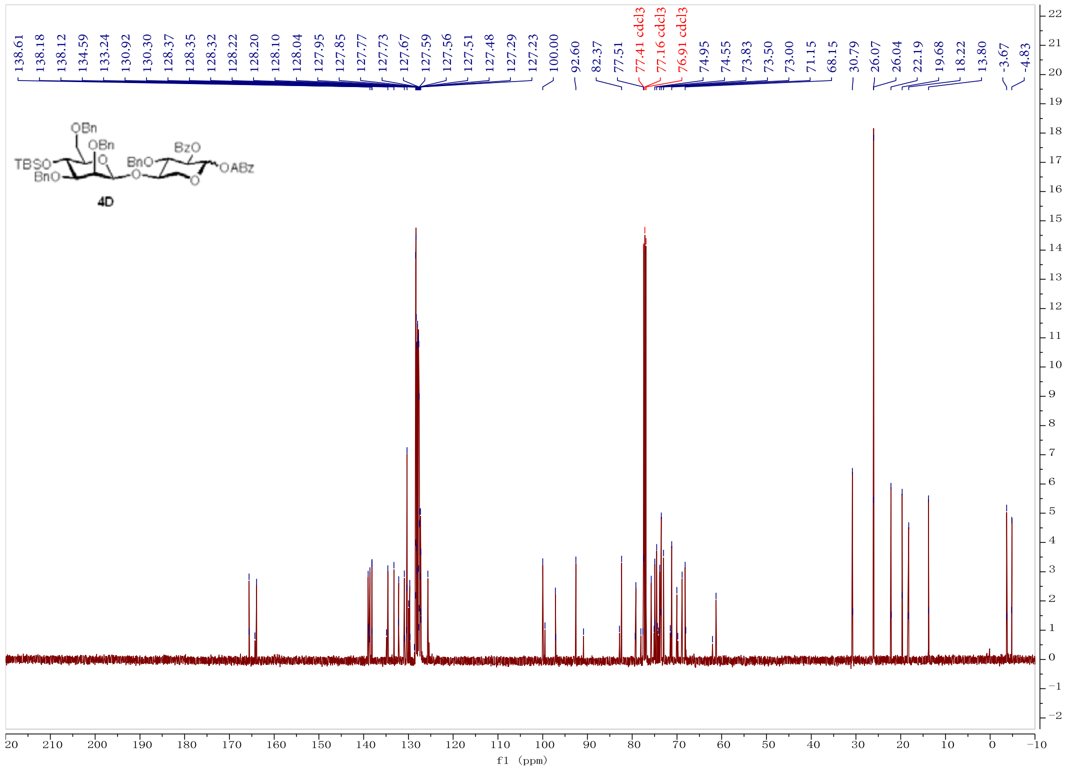

^{13}C NMR spectrum of 4D (126 MHz, $CDCl_3$, 25 °C)

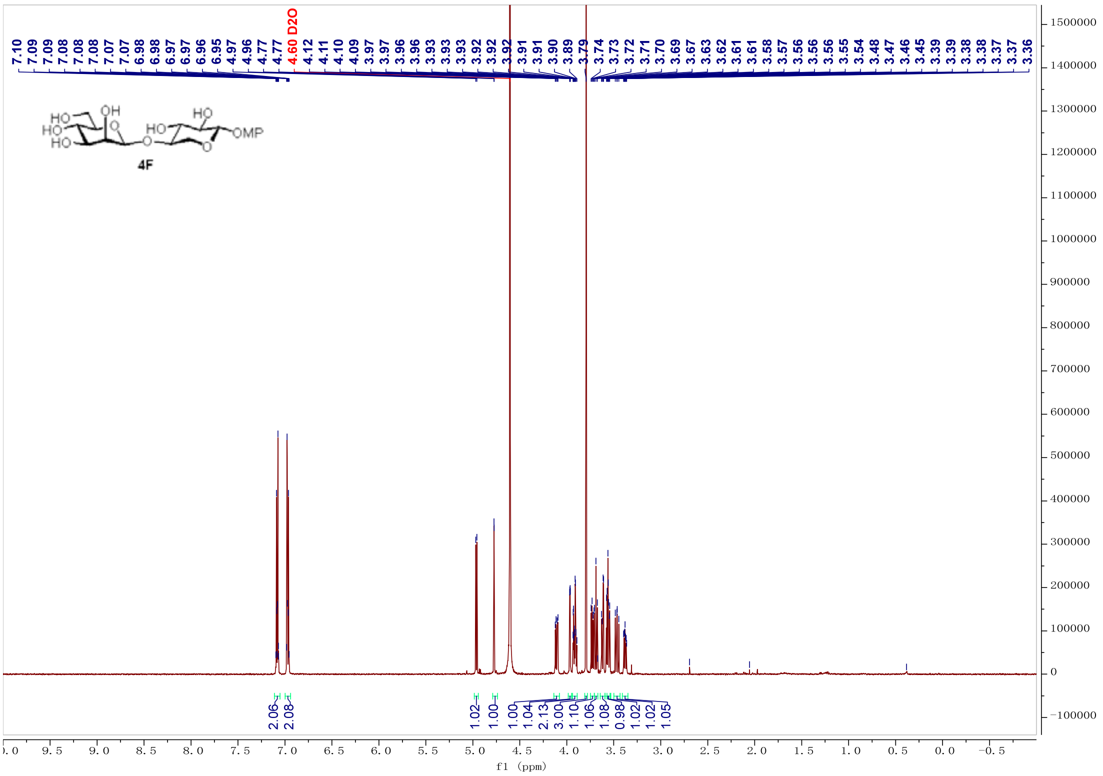

^{1}H NMR spectrum of compound 4F (600 MHz, 20 mM Na_3PO_4 buffered D_2O, 40 °C)

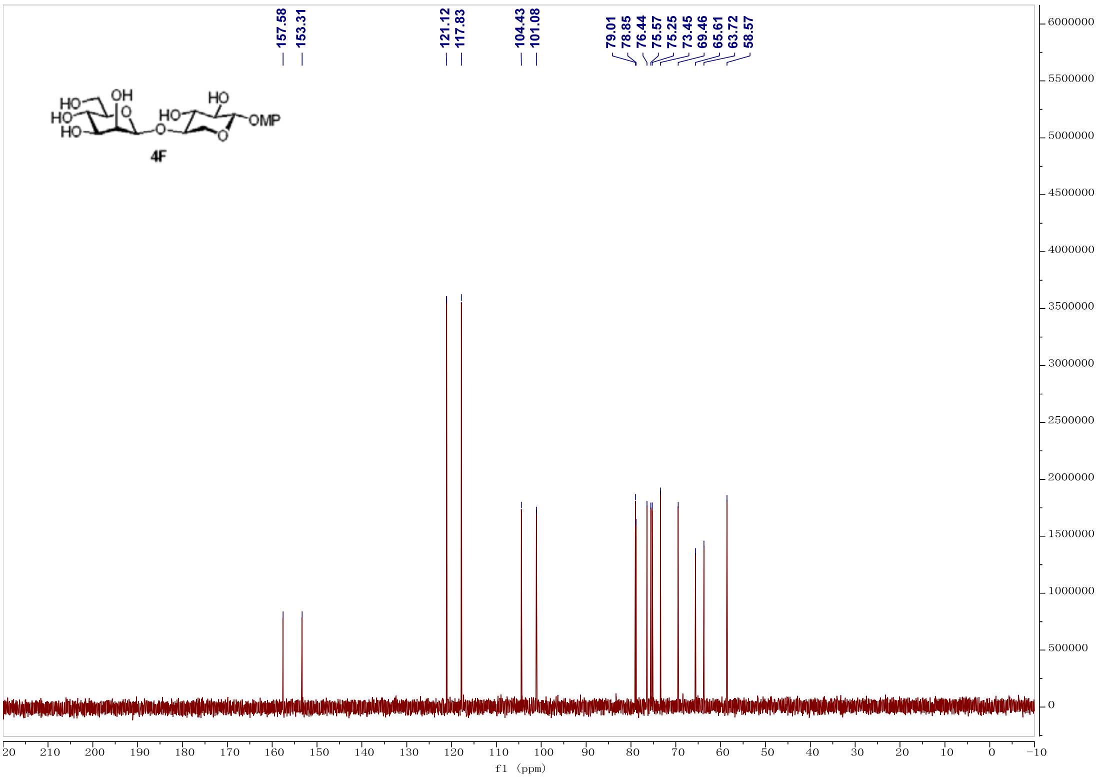

^{13}C NMR spectrum of compound 4F (151 MHz, 20 mM Na_3PO_4 buffered D_2O, 40 °C)

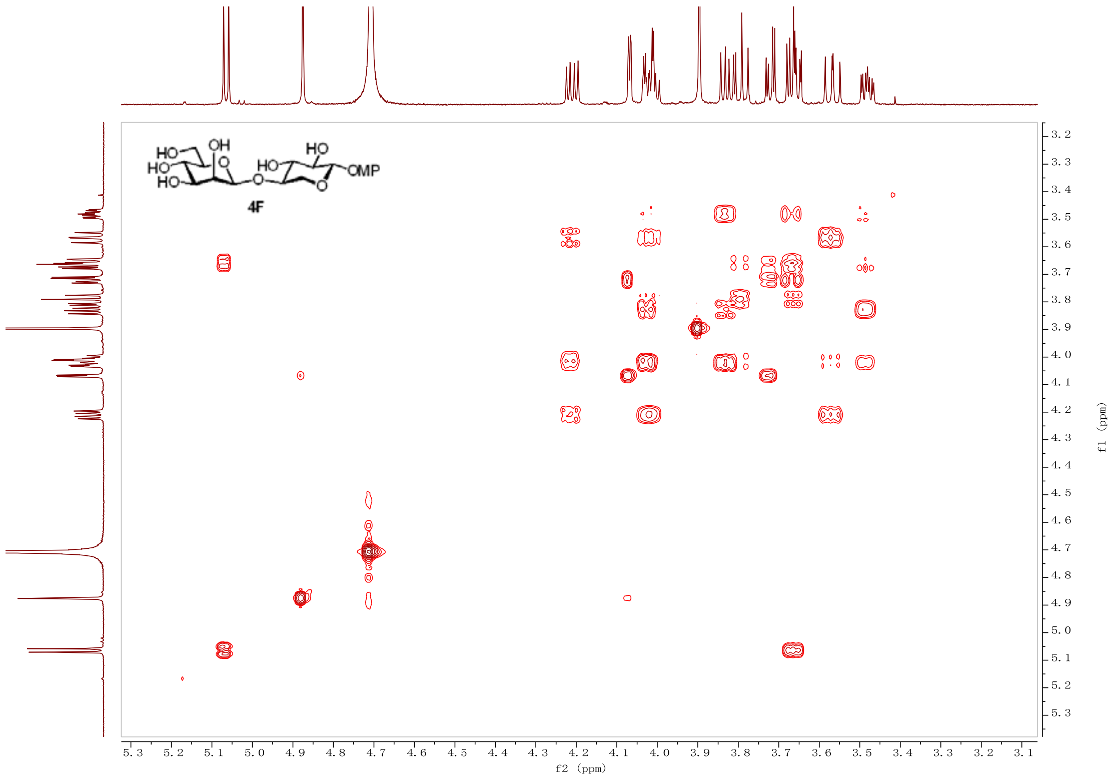

^{1}H-^{1}H gCOSY spectrum of compound 4F (600 MHz, 20 mM Na_3PO_4 buffered D_2O, 40 °C)

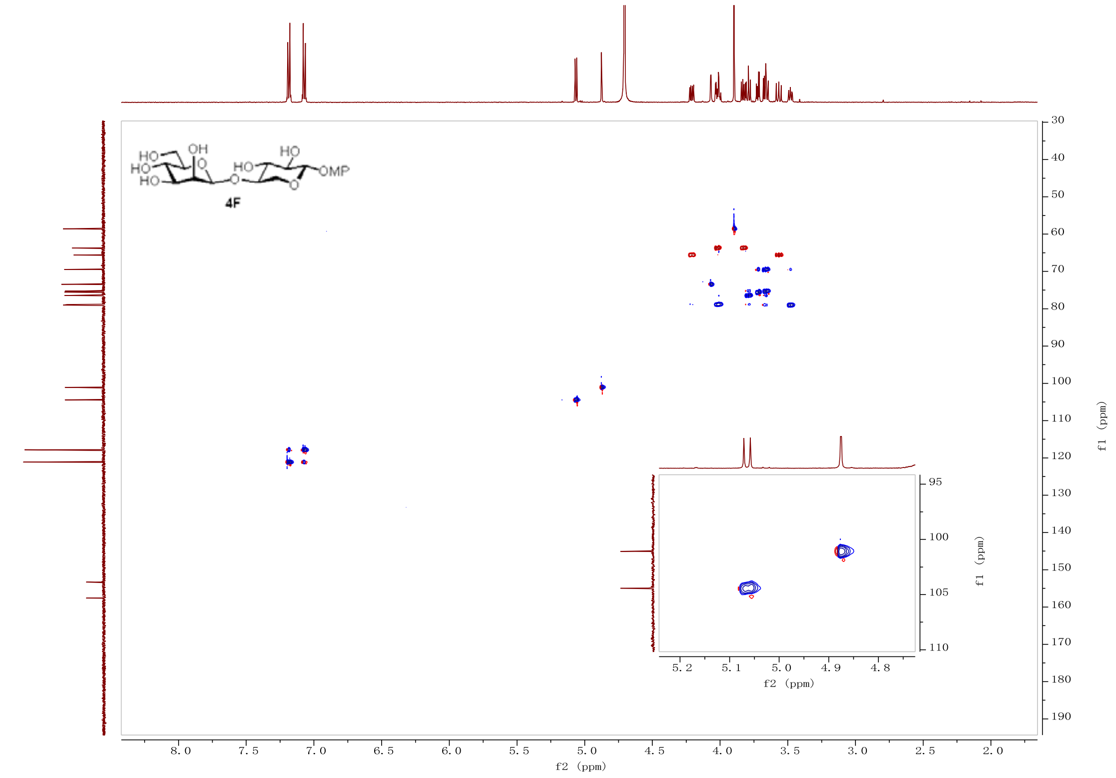

^{1}H-^{13}C HSQC spectrum of compound 4F (600 MHz, 20 mM Na_3PO_4 buffered D_2O, 40 °C)

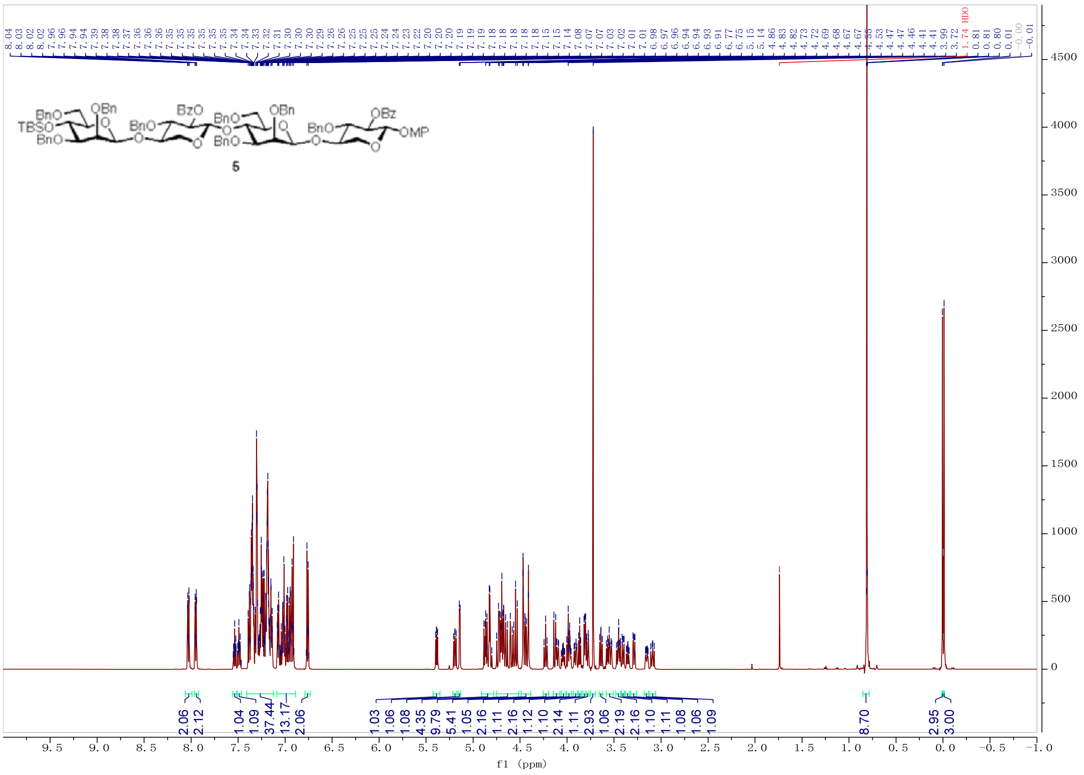

^{1}H NMR spectrum of 5 (600 MHz, $CDCl_3$, 25 °C)

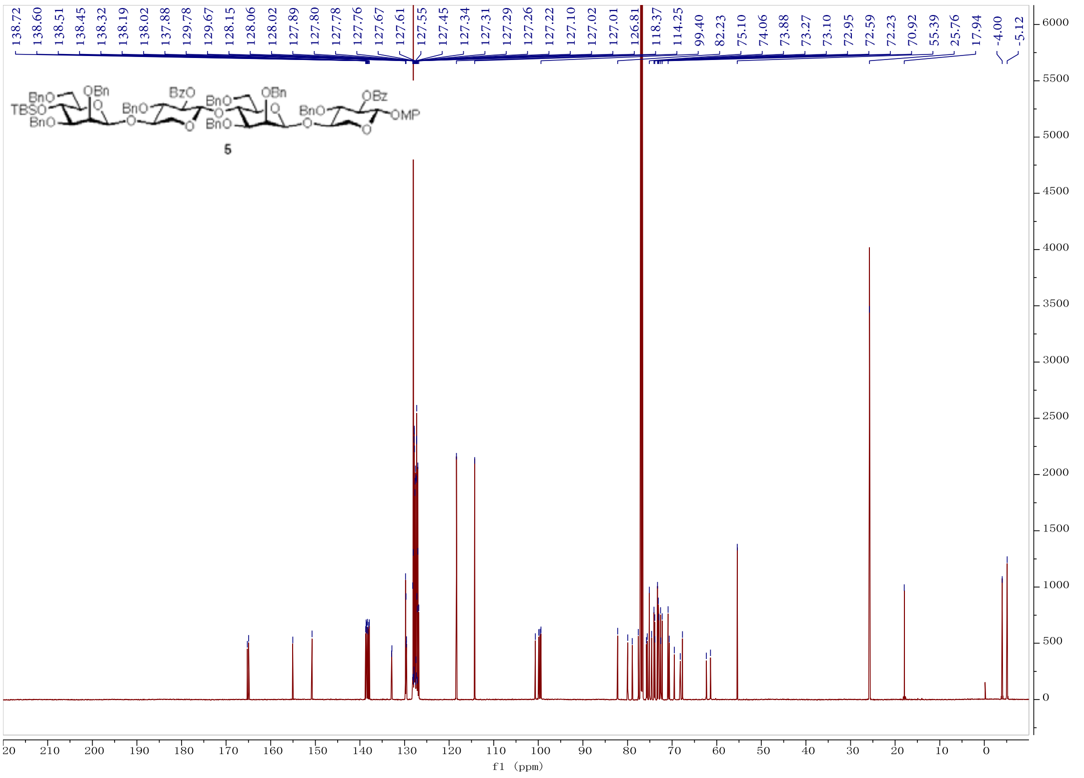

^{13}C NMR spectrum of 5 (151 MHz, $CDCl_3$, 25 °C)

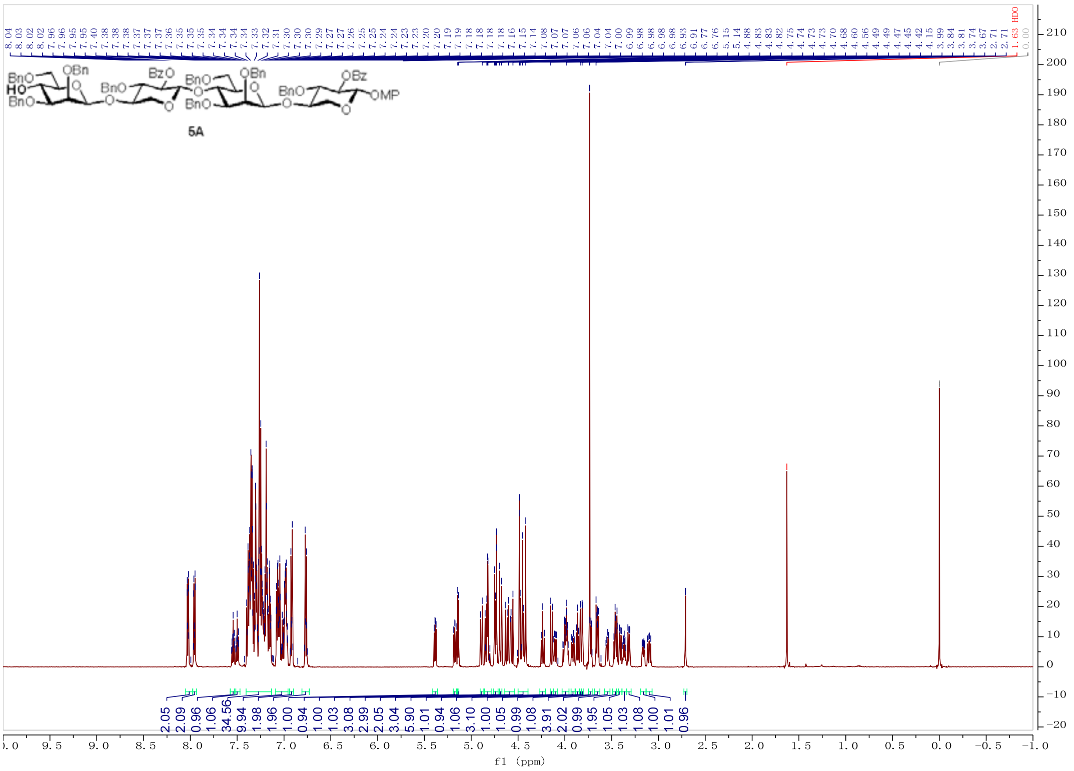

^{1}H NMR spectrum of 5A (600 MHz, $CDCl_3$, 25 °C)

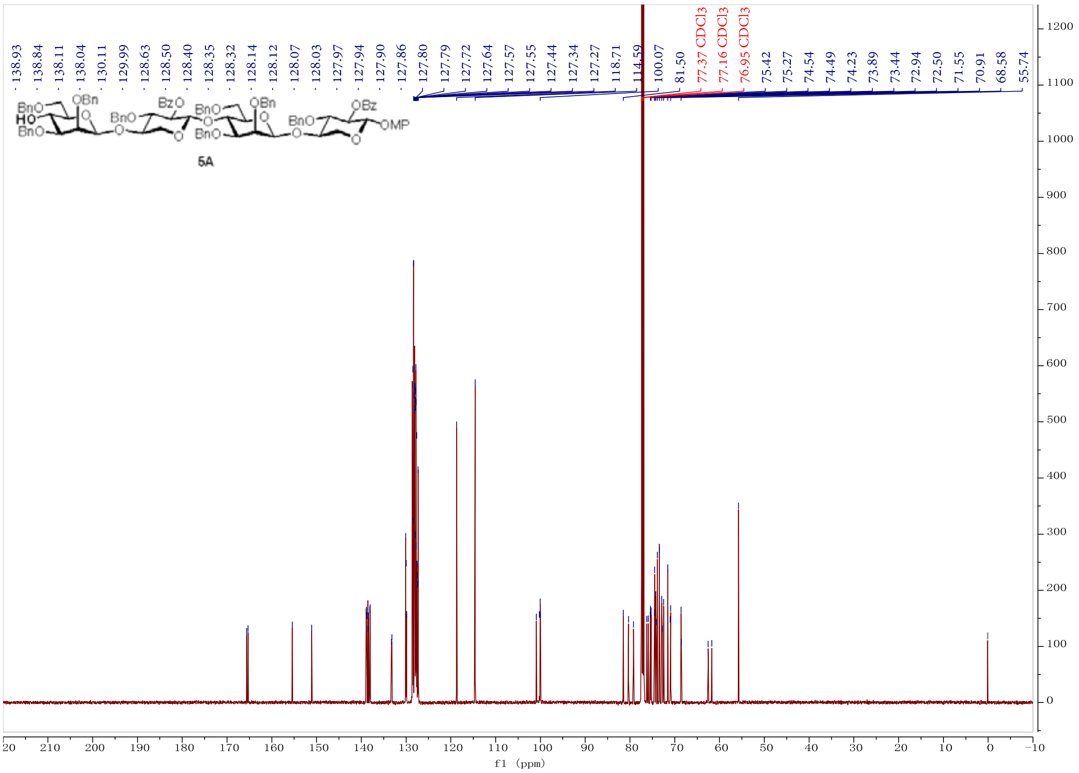

^{13}C NMR spectrum of 5A (151 MHz, $CDCl_3$, 25 °C)

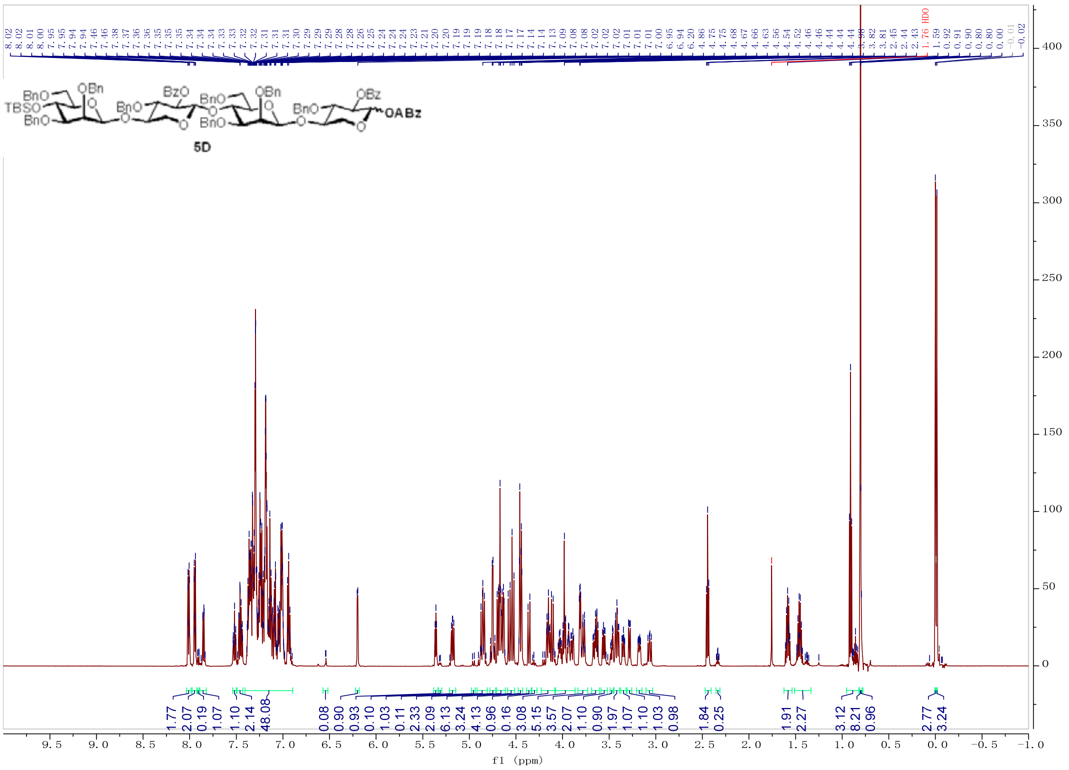

^{1}H NMR spectrum of 5D (600 MHz, $CDCl_3$, 25 °C)

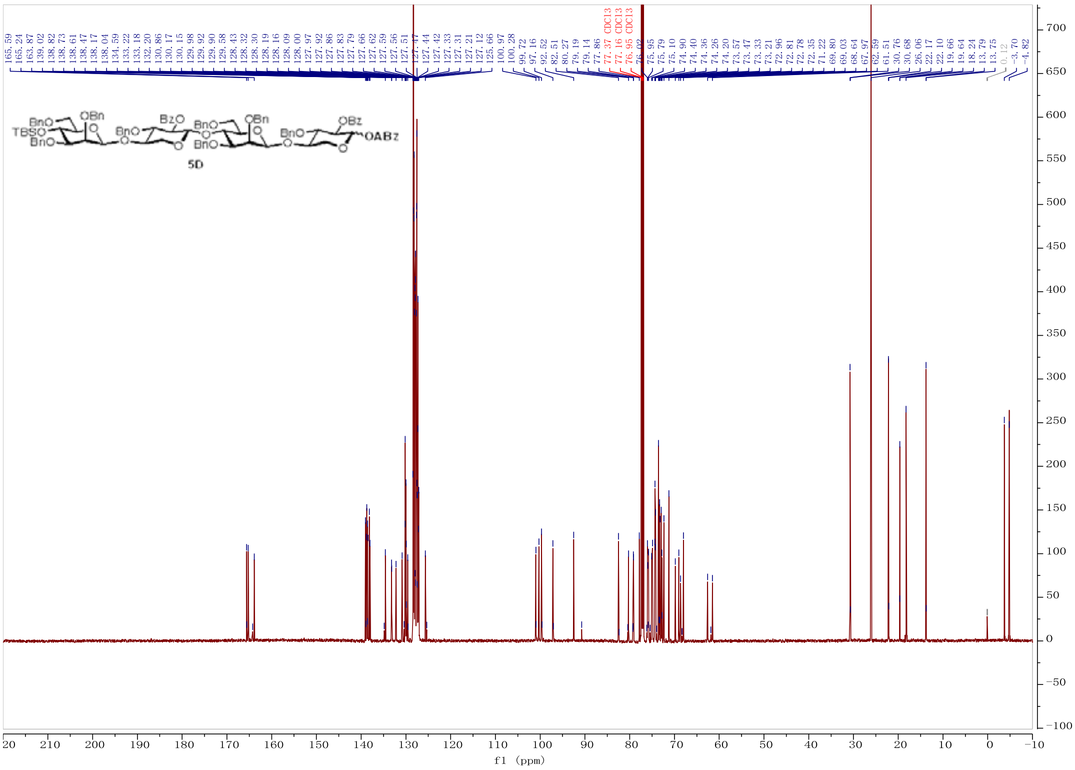

^{13}C NMR spectrum of 5D (151 MHz, $CDCl_3$, 25 °C)

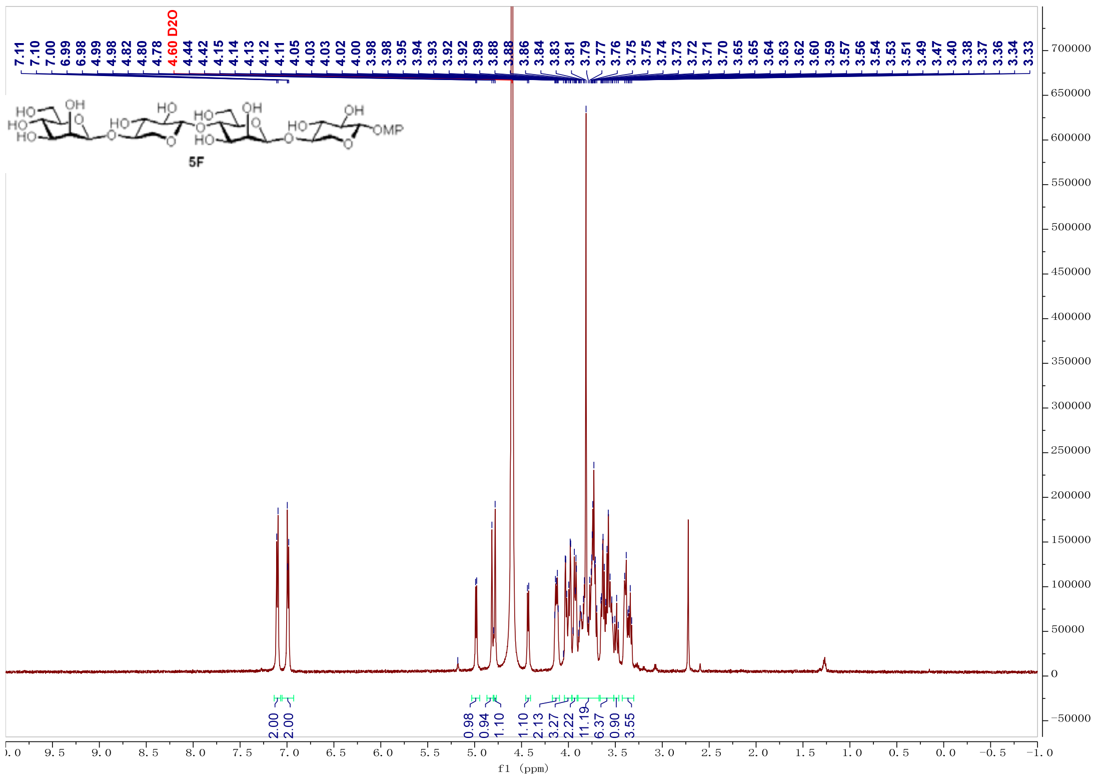

^{1}H NMR spectrum of compound 5F (600 MHz, 20 mM Na_3PO_4 buffered D_2O, 40 °C)

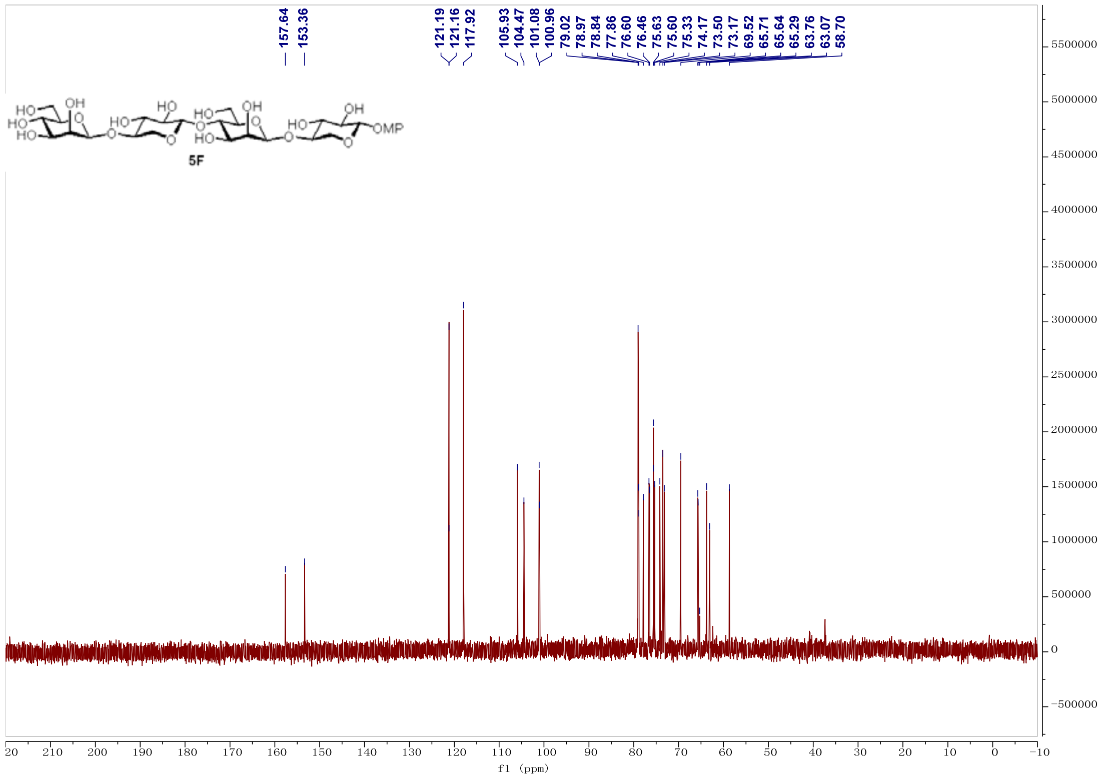

^{13}C NMR spectrum of compound 5F (151 MHz, 20 mM Na_3PO_4 buffered D_2O, 40 °C)

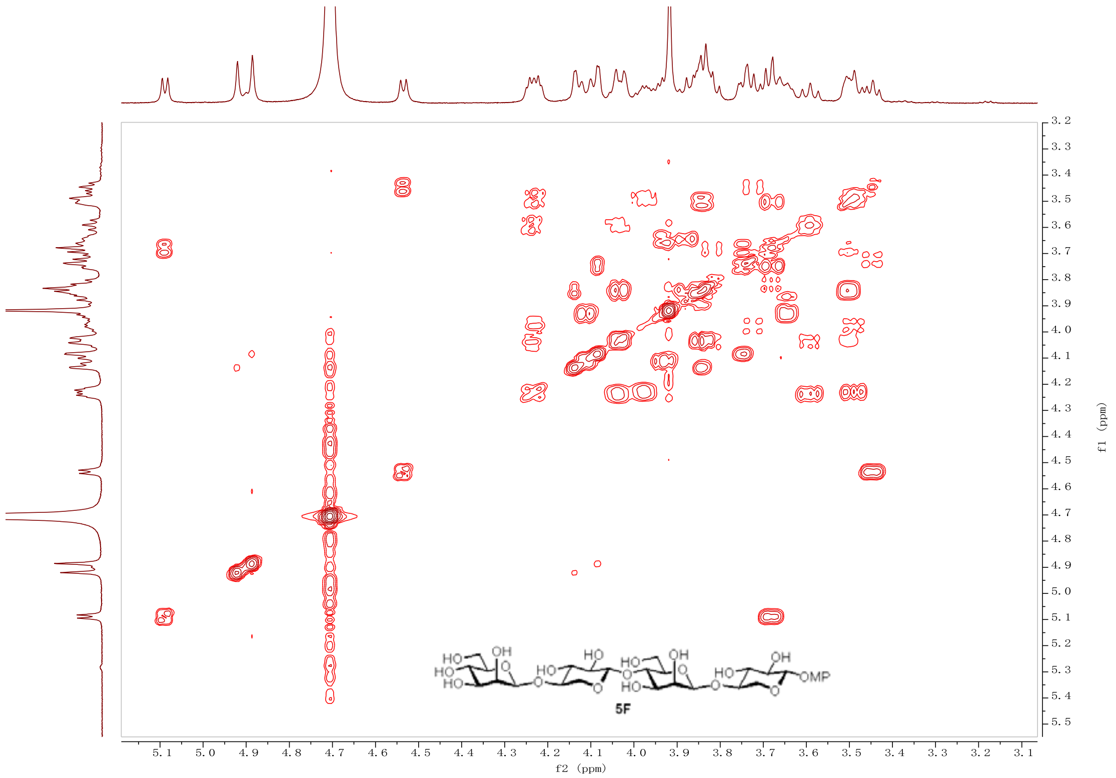

^{1}H-^{1}H gCOSY spectrum of compound 5F (600 MHz, 20 mM Na_3PO_4 buffered D_2O, 40 °C)

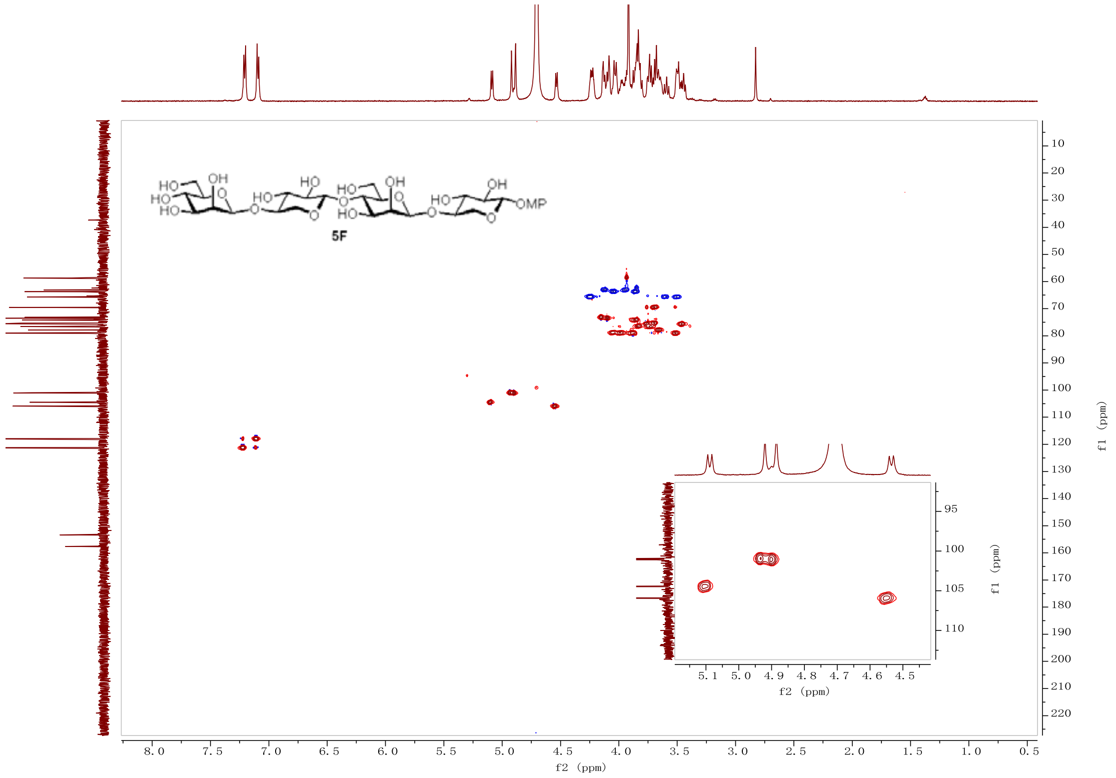

^{1}H-^{13}C HSQC spectrum of compound 5F (600 MHz, 20 mM Na_3PO_4 buffered D_2O, 40 °C)

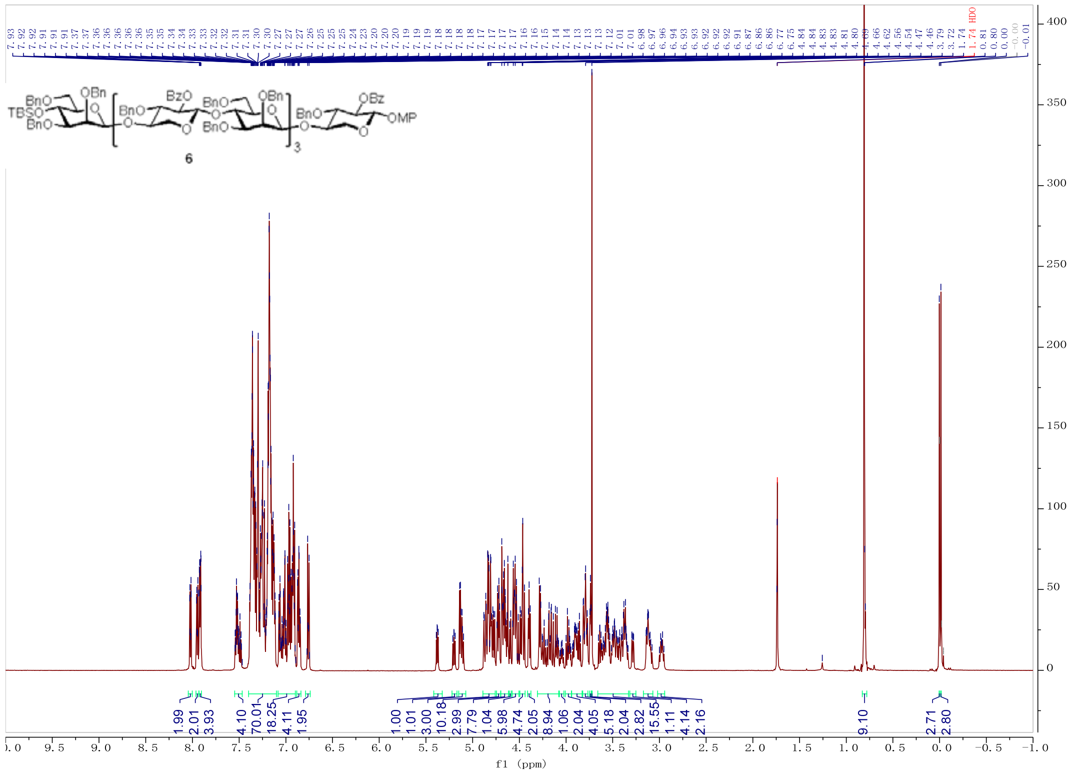

^{1}H NMR spectrum of 6 (600 MHz, $CDCl_3$, 25 °C)

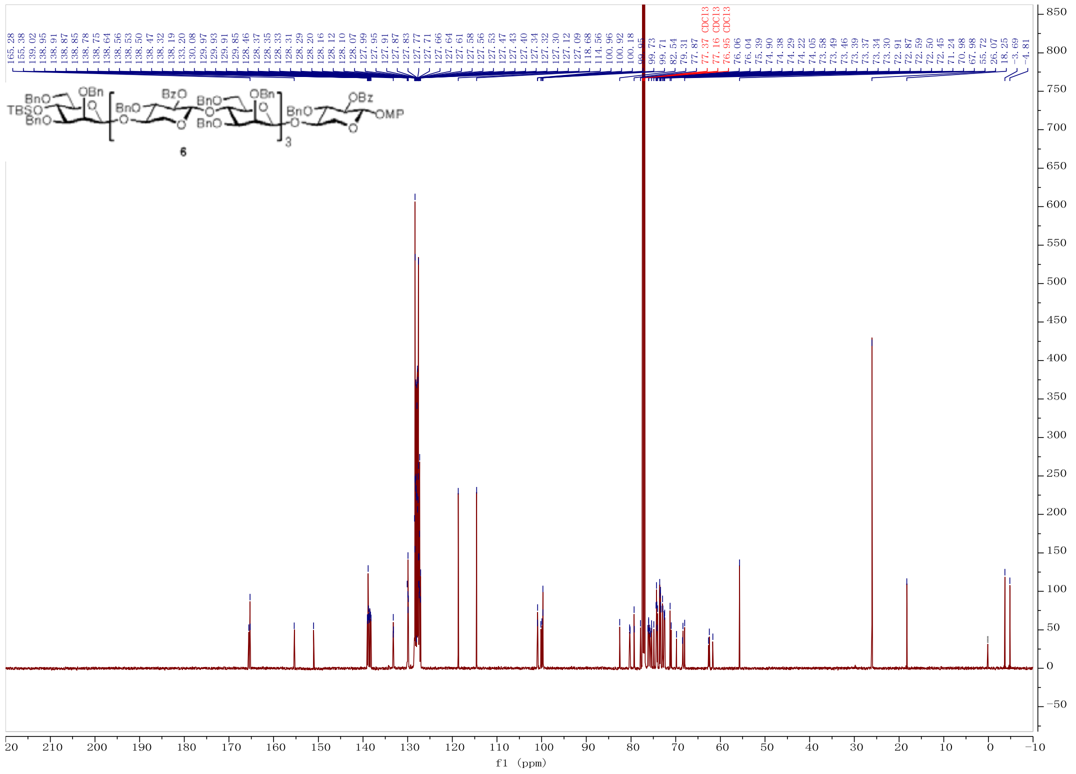

^{13}C NMR spectrum of 6 (151 MHz, $CDCl_3$, 25 °C)

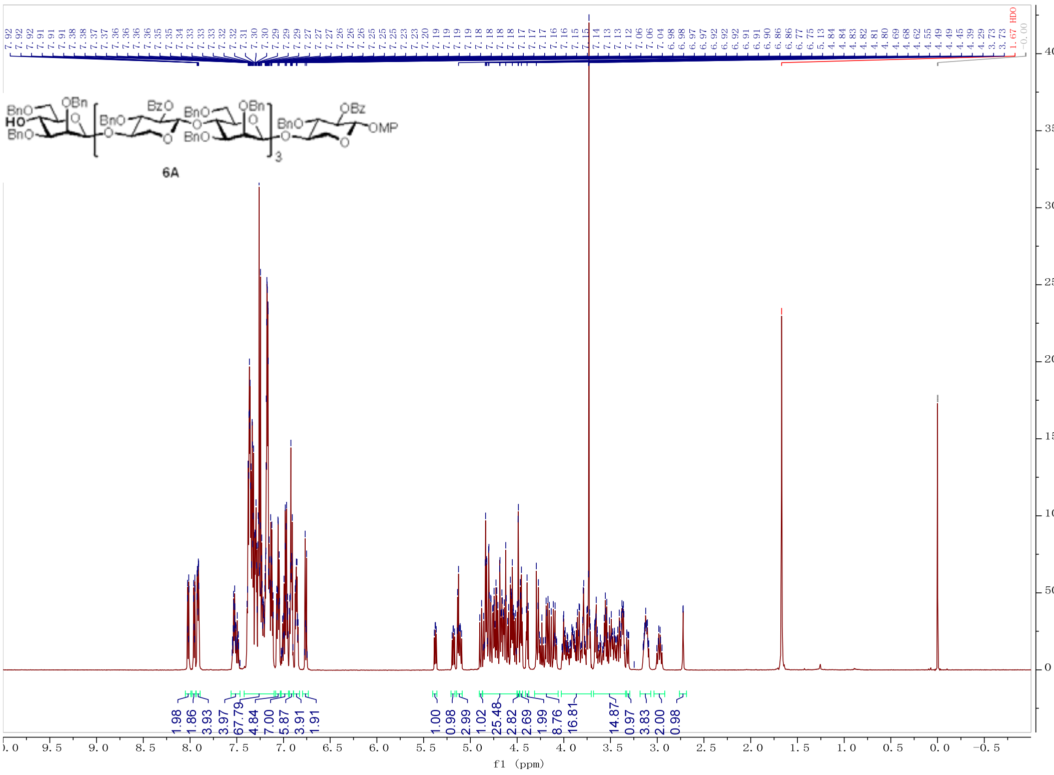

^{1}H NMR spectrum of 6A (600 MHz, $CDCl_3$, 25 °C)

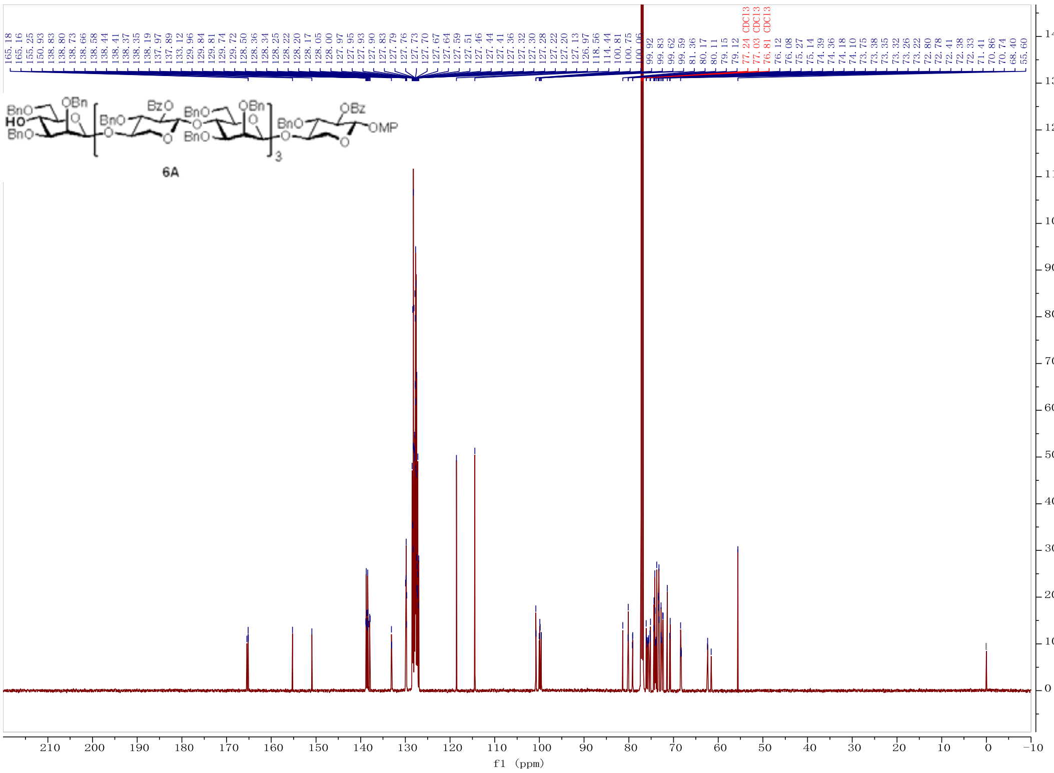

^{13}C NMR spectrum of 6A (151 MHz, $CDCl_3$, 25 °C)

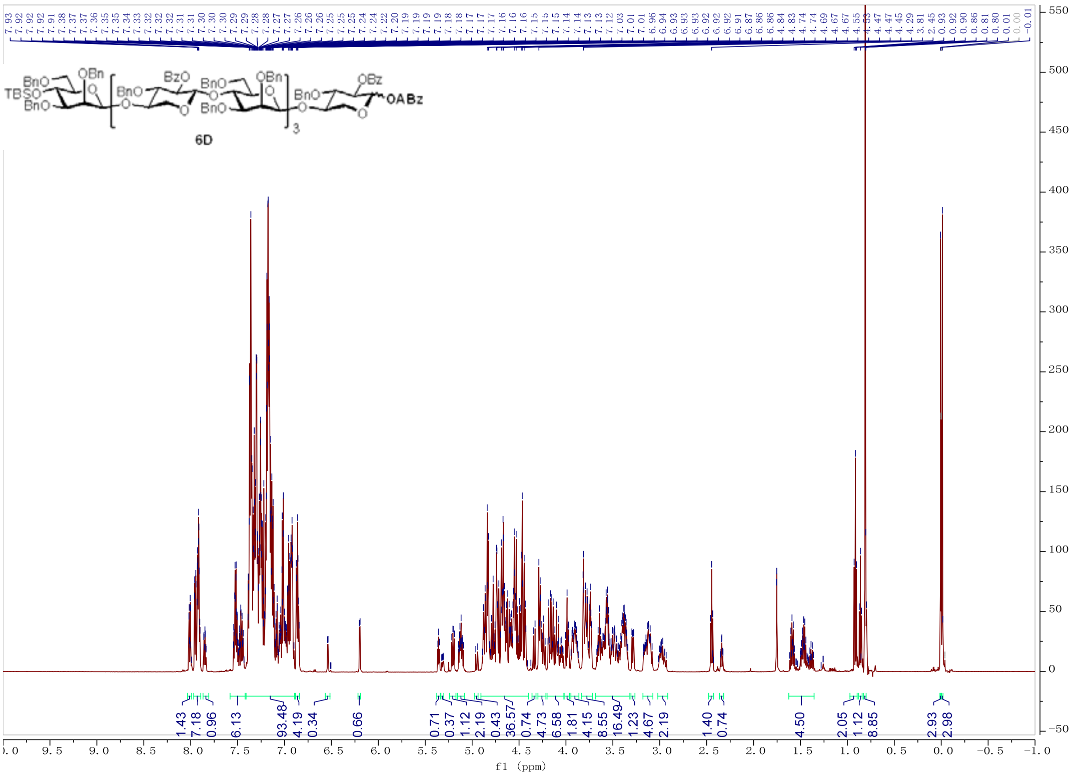

^{1}H NMR spectrum of 6D (600 MHz, $CDCl_3$, 25 °C)

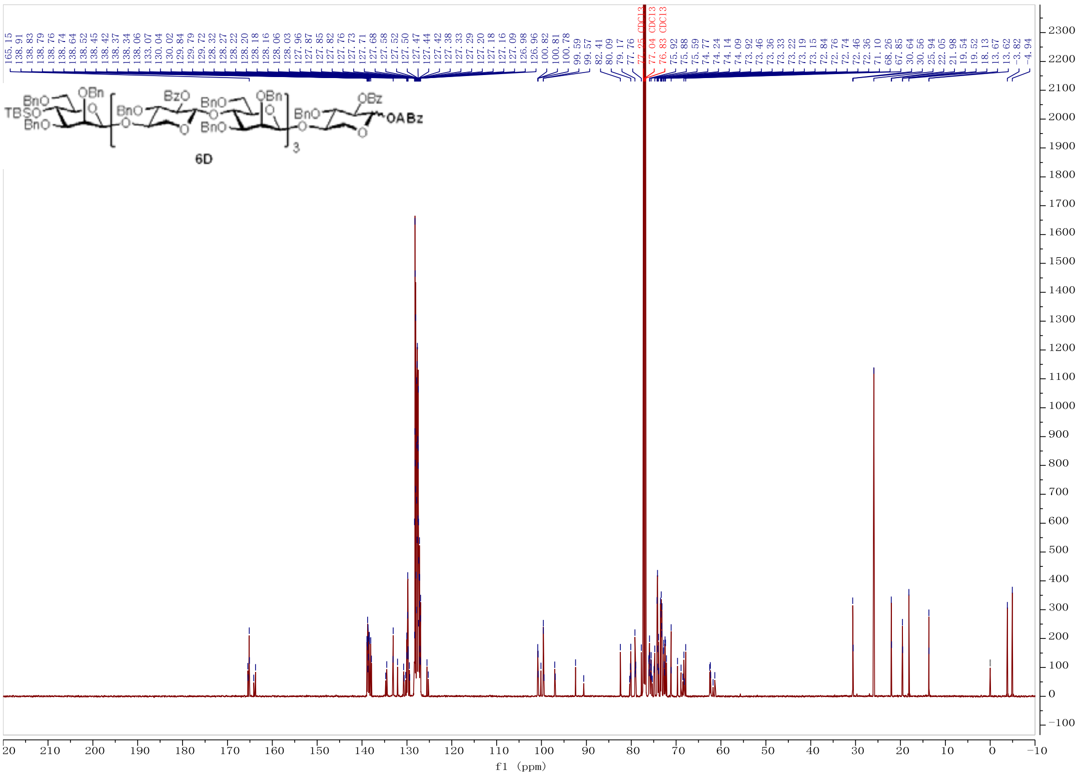

^{13}C NMR spectrum of 6D (151 MHz, $CDCl_3$, 25 °C)

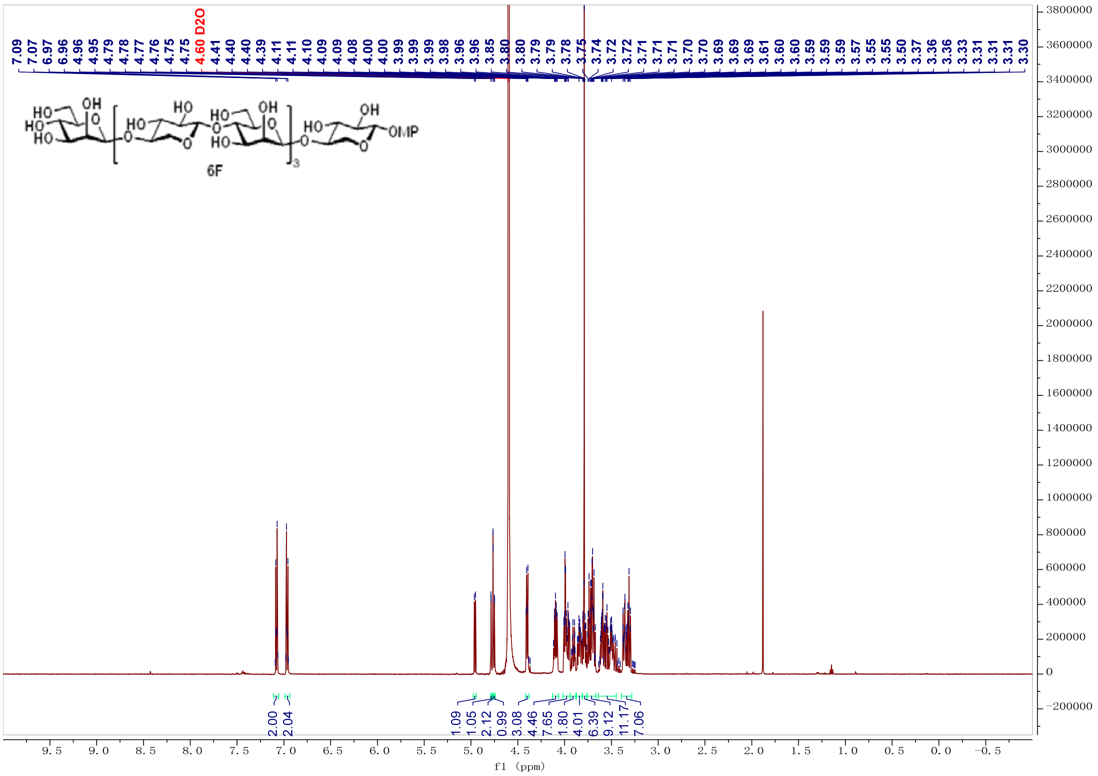

^{1}H NMR spectrum of compound 6F (600 MHz, 20 mM Na_3PO_4 buffered D_2O, 40 °C)

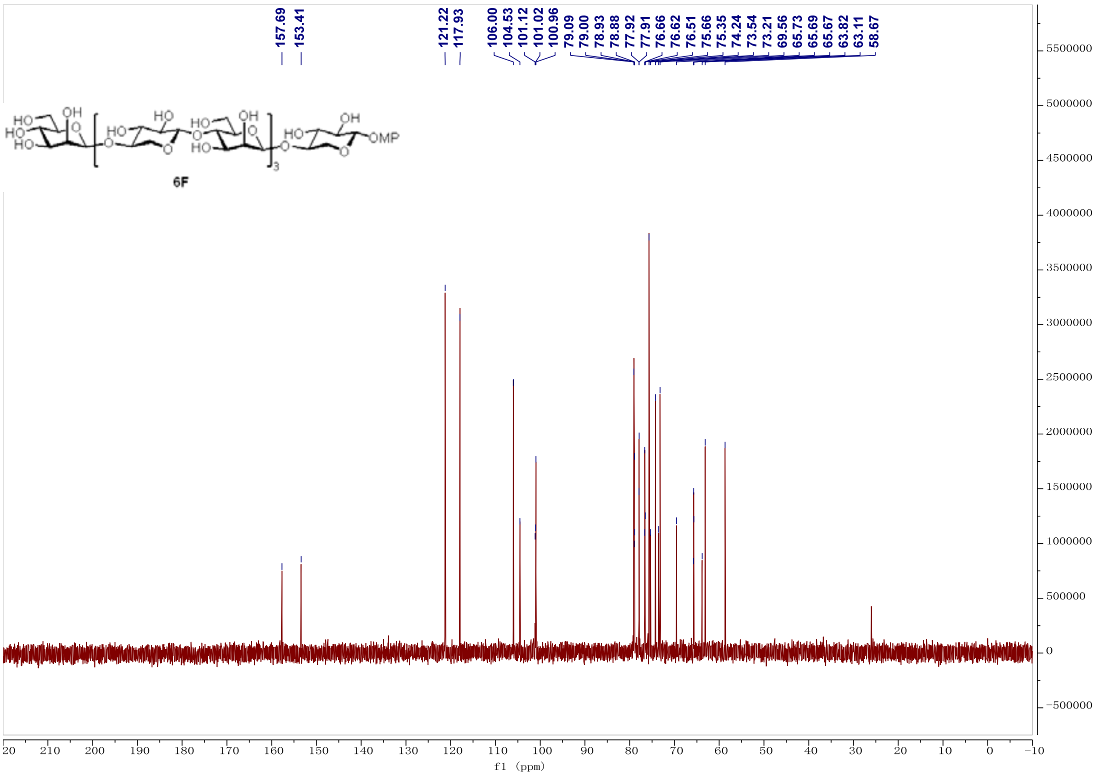

^{13}C NMR spectrum of compound 6F (151 MHz, 20 mM Na_3PO_4 buffered D_2O, 40 °C)

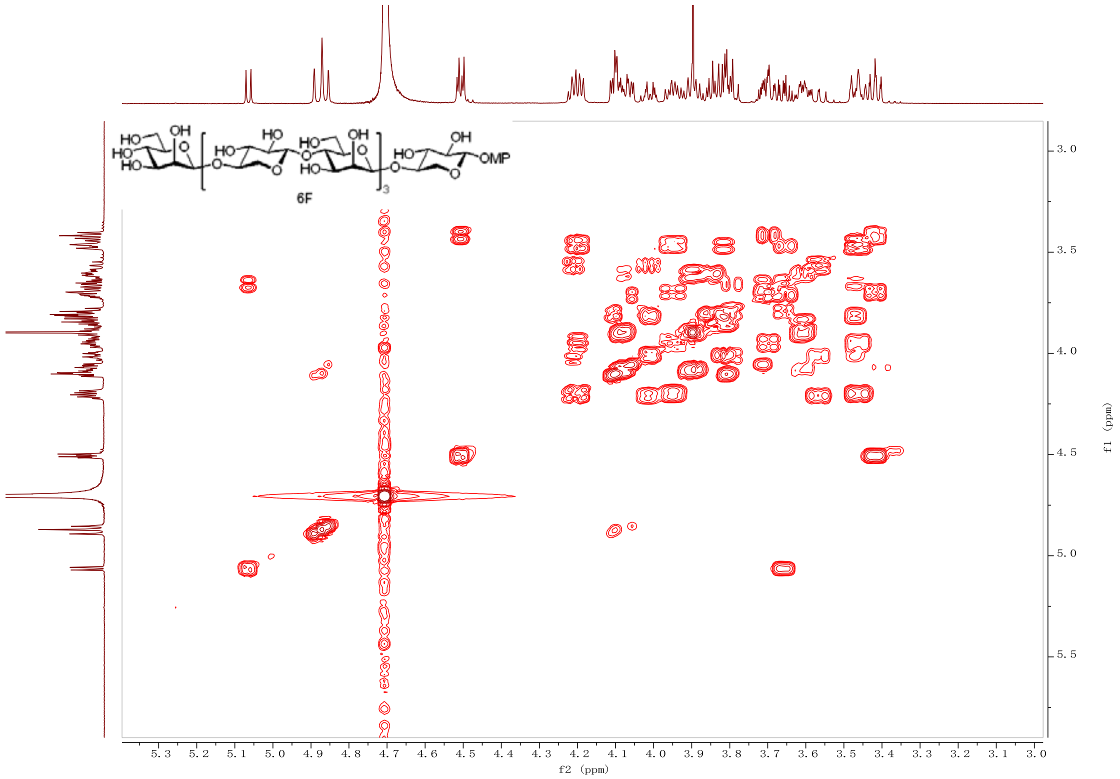

^{1}H-^{1}H gCOSY spectrum of compound 6F (600 MHz, 20 mM Na_3PO_4 buffered D_2O, 40 °C)

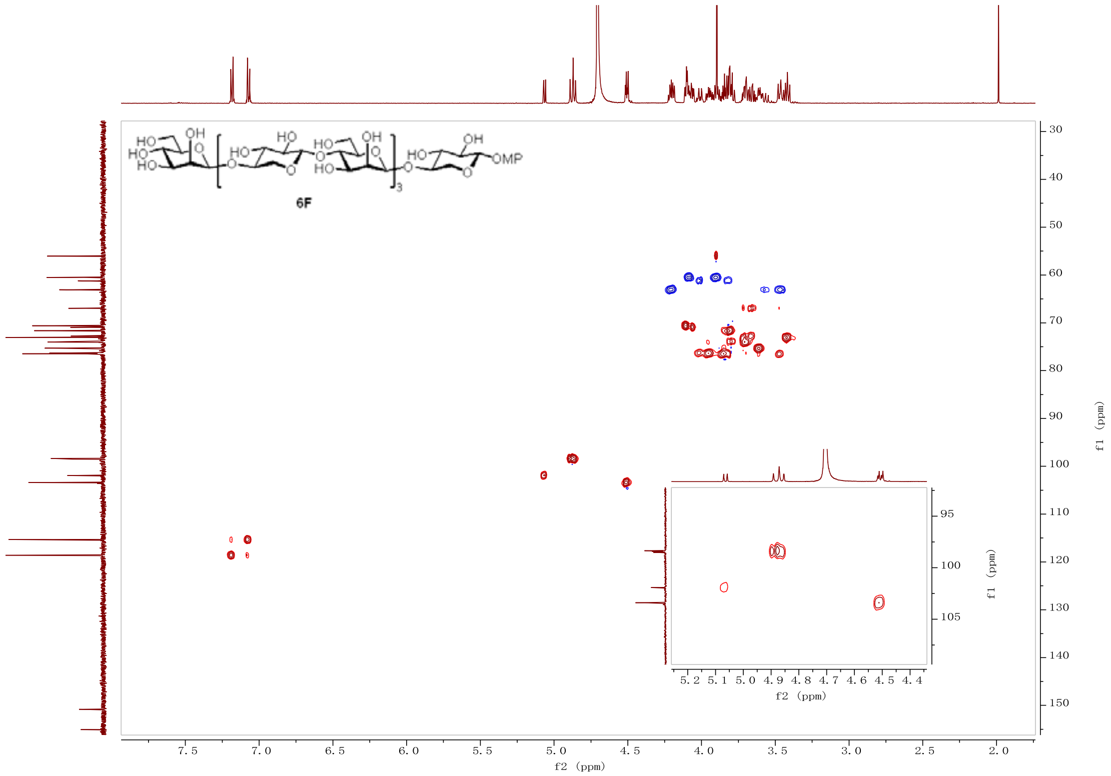

^{1}H-^{13}C HSQC spectrum of compound 6F (600 MHz, 20 mM Na_3PO_4 buffered D_2O, 40 °C)

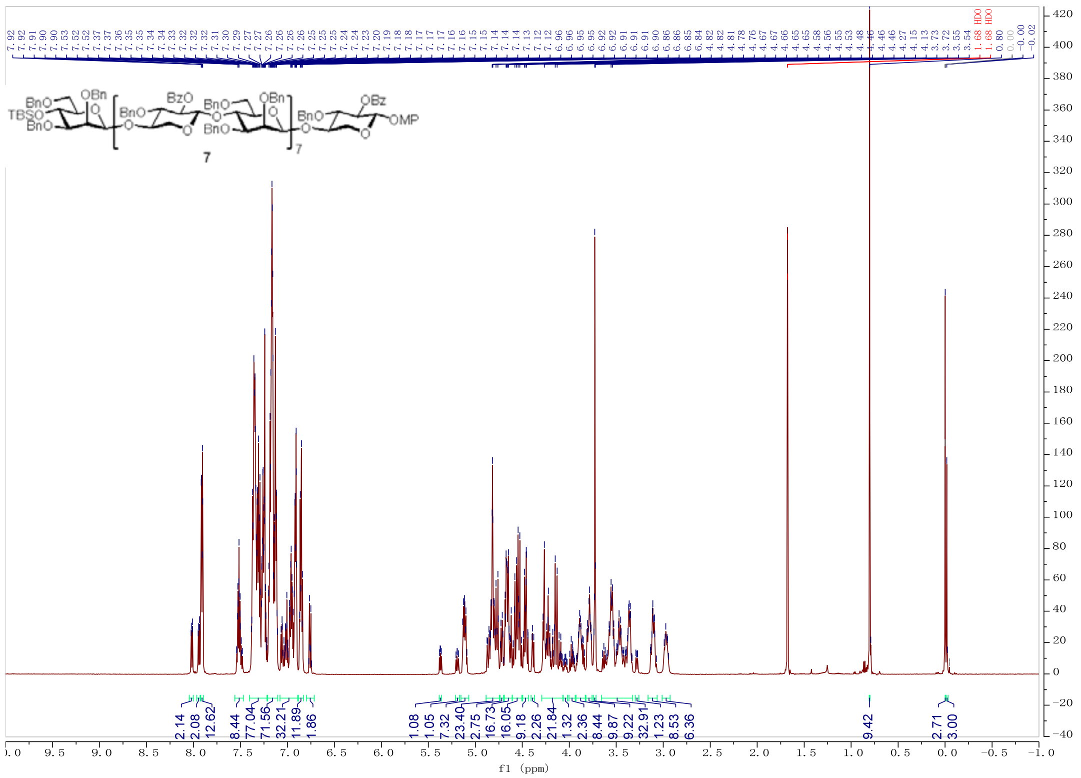

^{1}H NMR spectrum of 7 (600 MHz, $CDCl_3$, 25 °C)

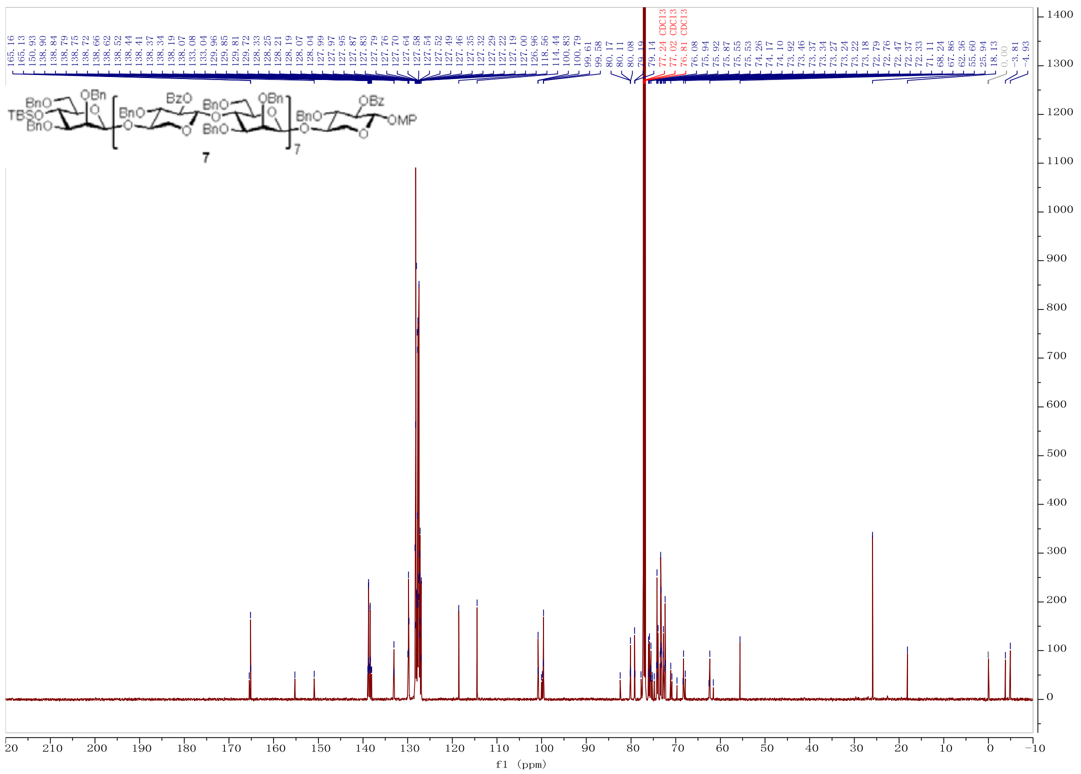

^{13}C NMR spectrum of 7 (151 MHz, $CDCl_3$, 25 °C)

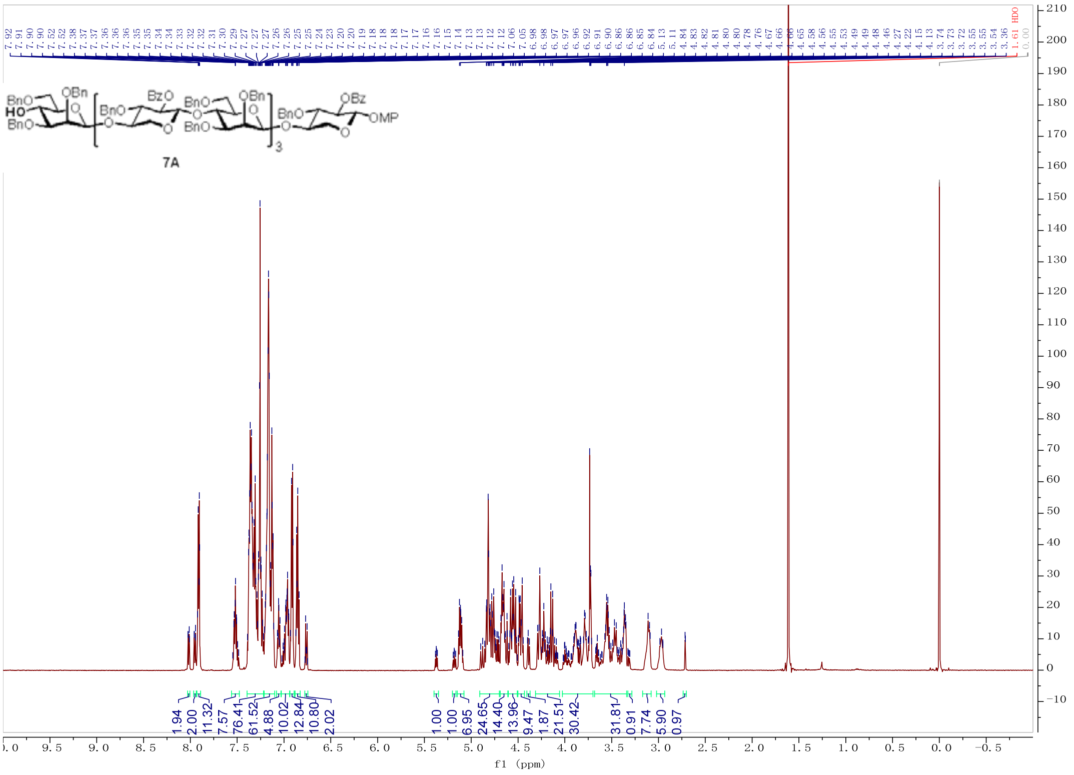

^{1}H NMR spectrum of 7A (600 MHz, $CDCl_3$, 25 °C)

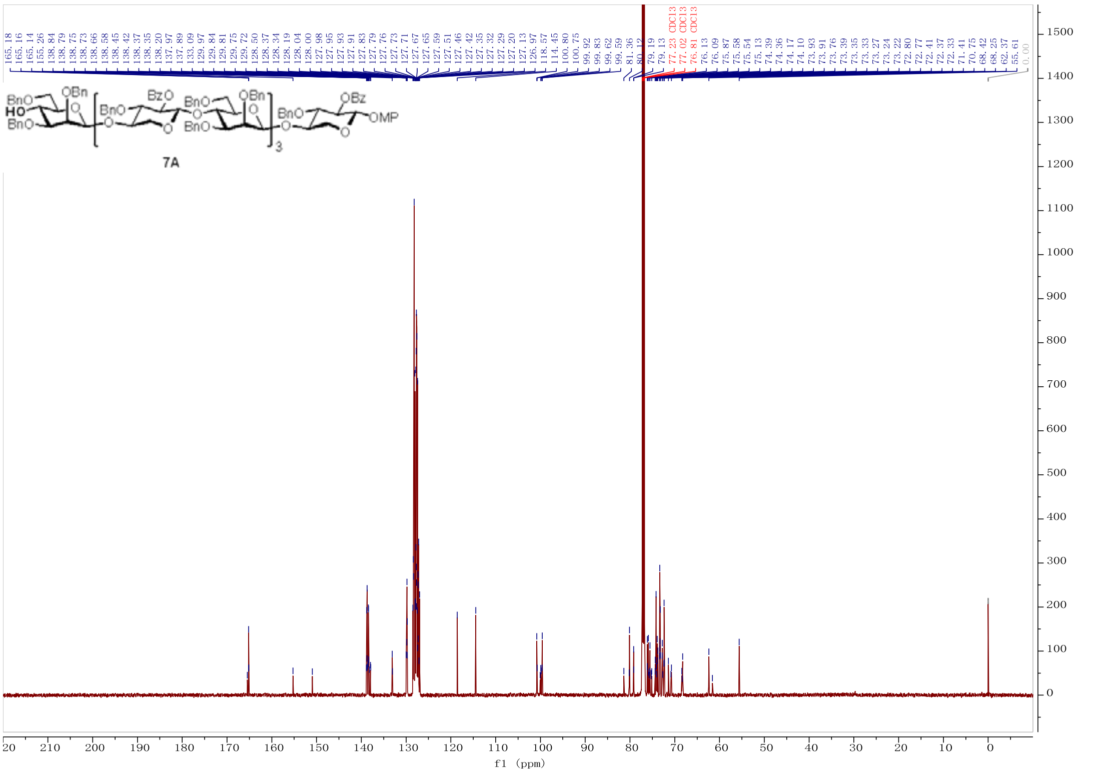

^{13}C NMR spectrum of 7A (151 MHz, $CDCl_3$, 25 °C)

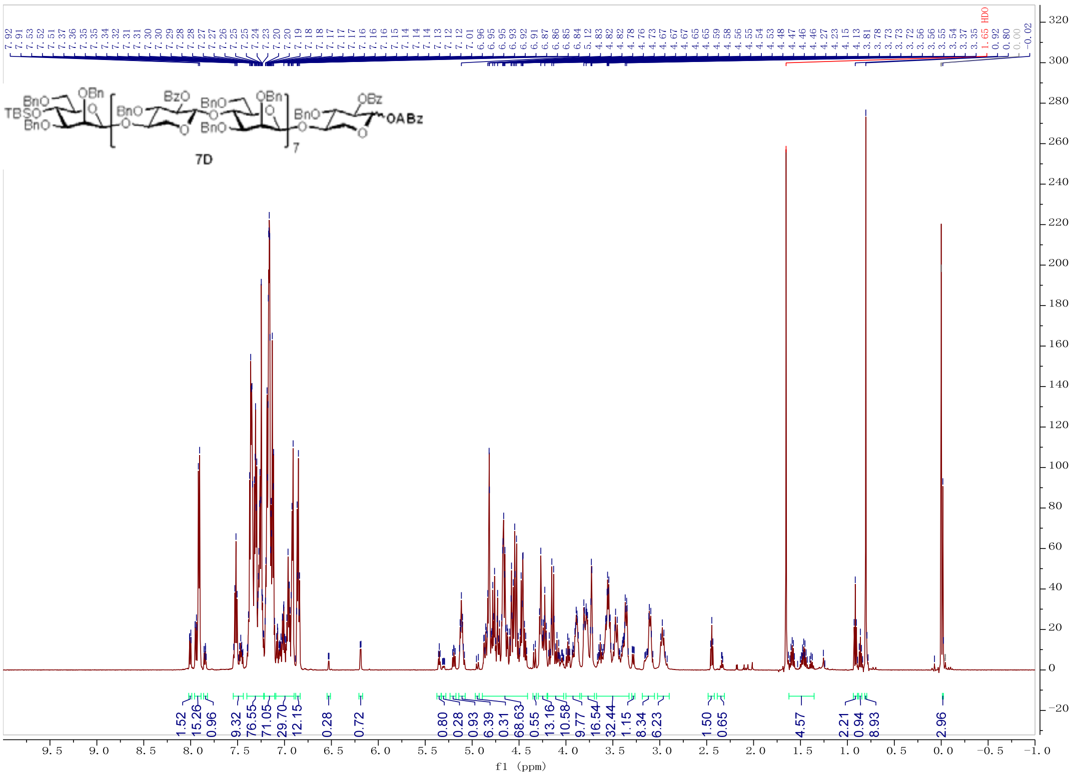

^{1}H NMR spectrum of 7D (600 MHz, $CDCl_3$, 25 °C)

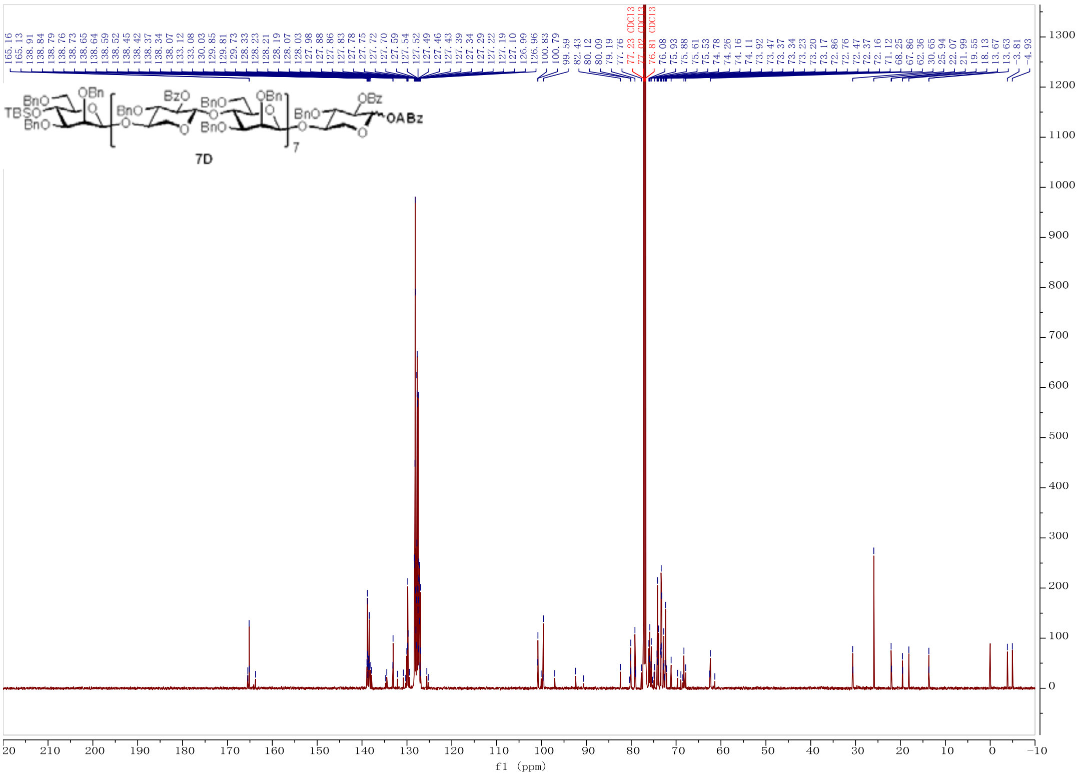

^{13}C NMR spectrum of 7D (151 MHz, $CDCl_3$, 25 °C)

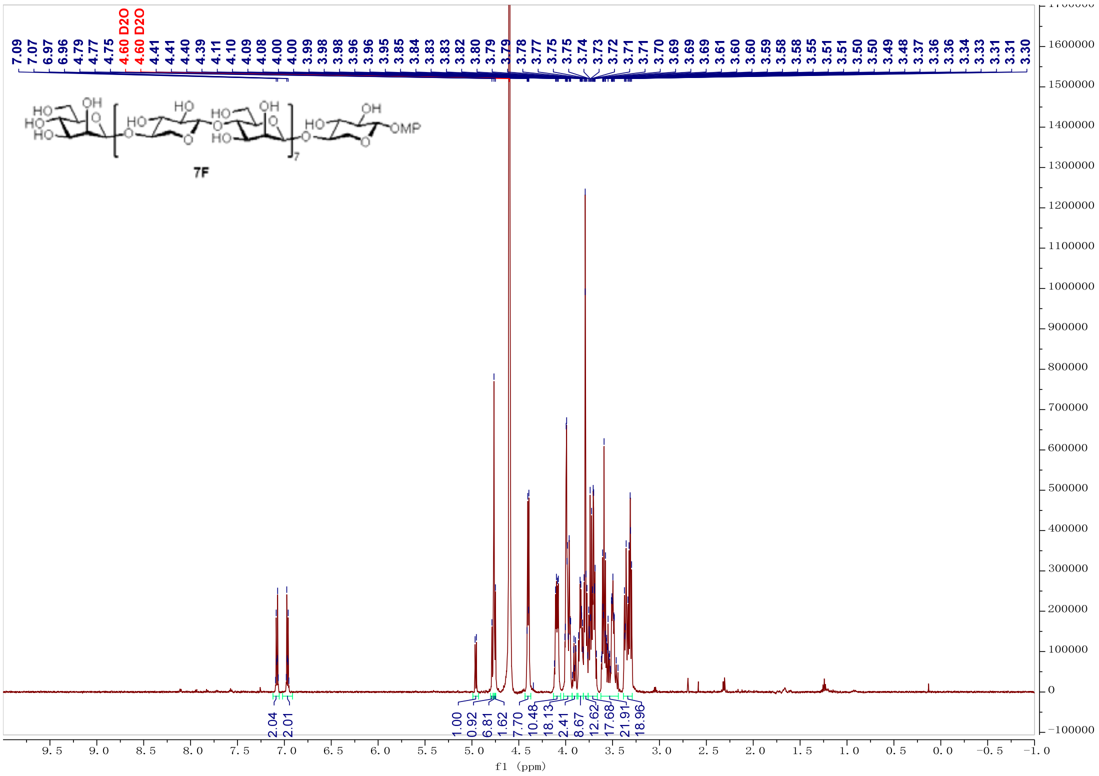

^{1}H NMR spectrum of compound 7F (600 MHz, 20 mM Na_3PO_4 buffered D_2O, 40 °C)

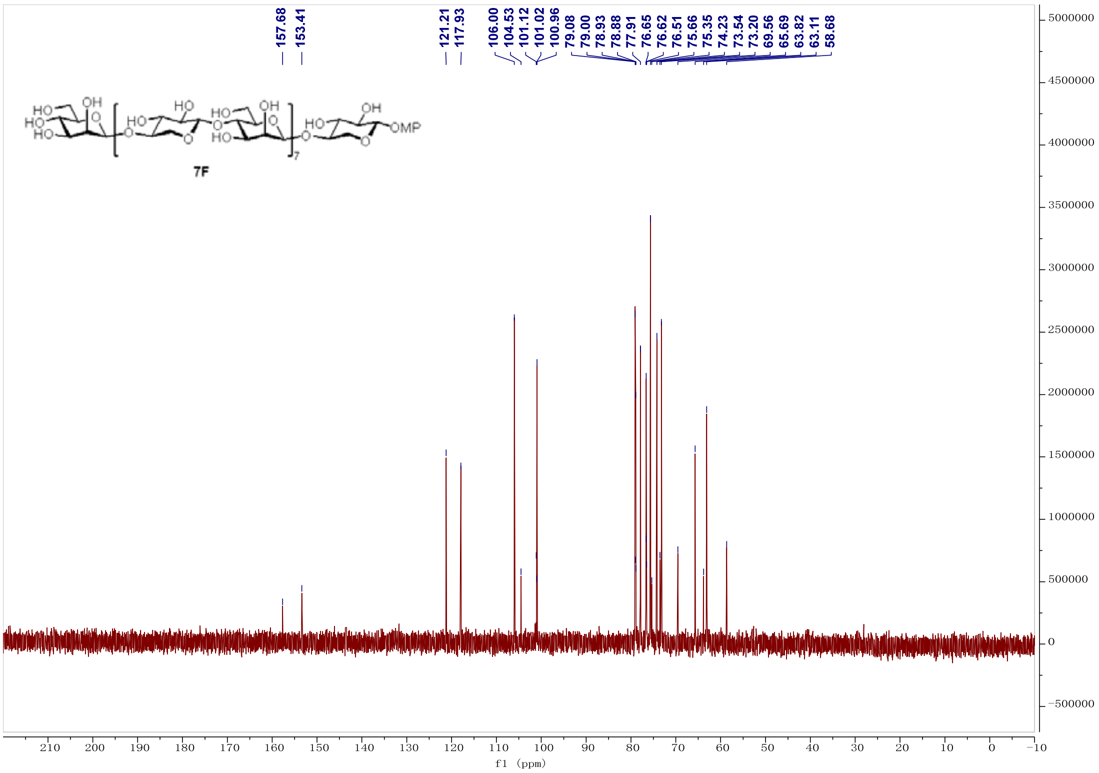

^{13}C NMR spectrum of compound 7F (151 MHz, 20 mM Na_3PO_4 buffered D_2O, 40 °C)

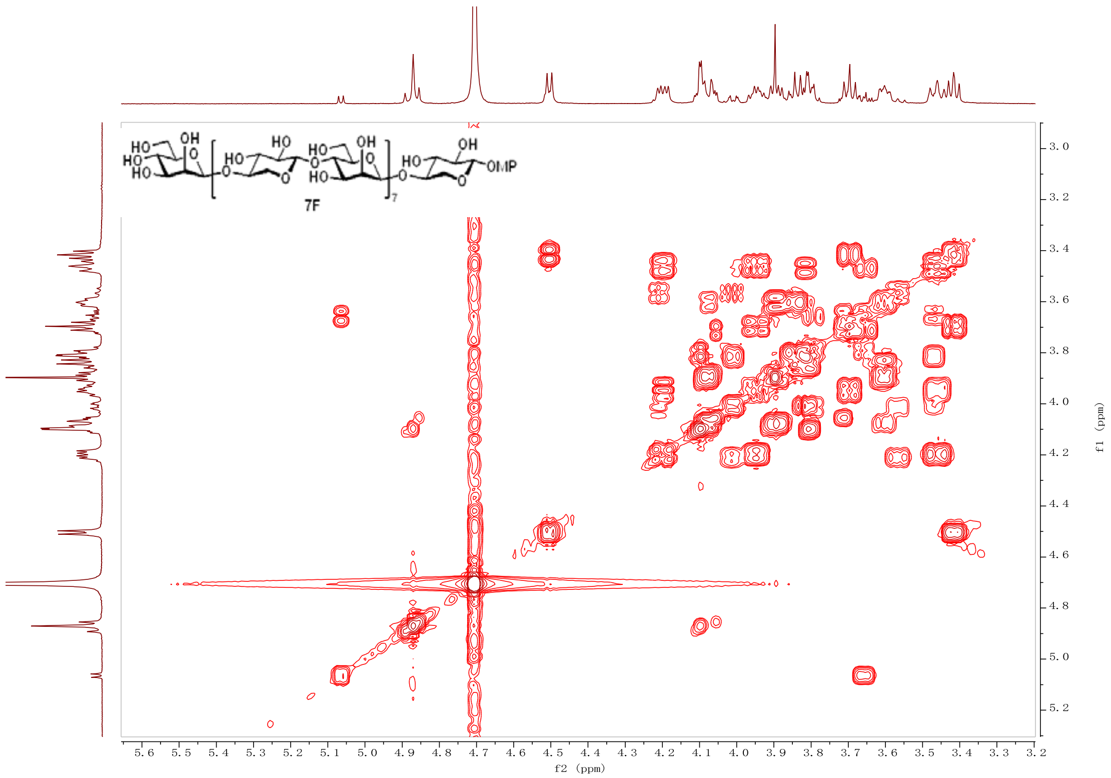

^{1}H-^{1}H gCOSY spectrum of compound 7F (600 MHz, 20 mM Na_3PO_4 buffered D_2O, 40 °C)

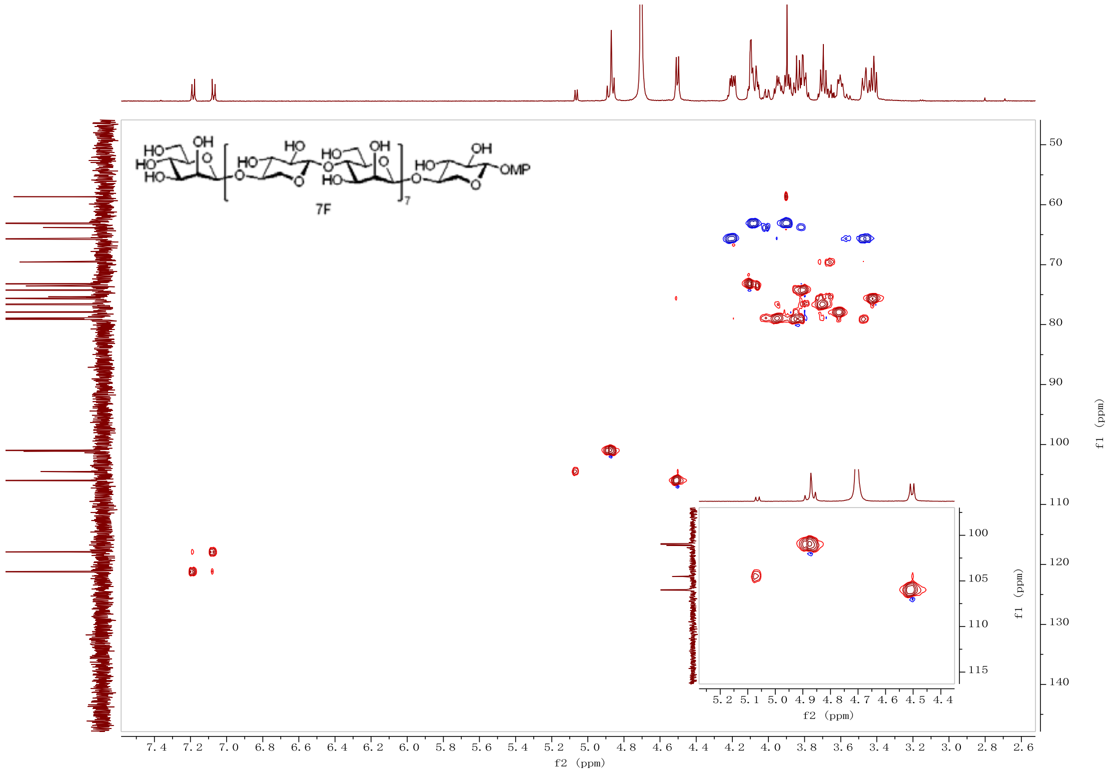

^{1}H-^{13}C HSQC spectrum of compound 7F (600 MHz, 20 mM Na_3PO_4 buffered D_2O, 40 °C)

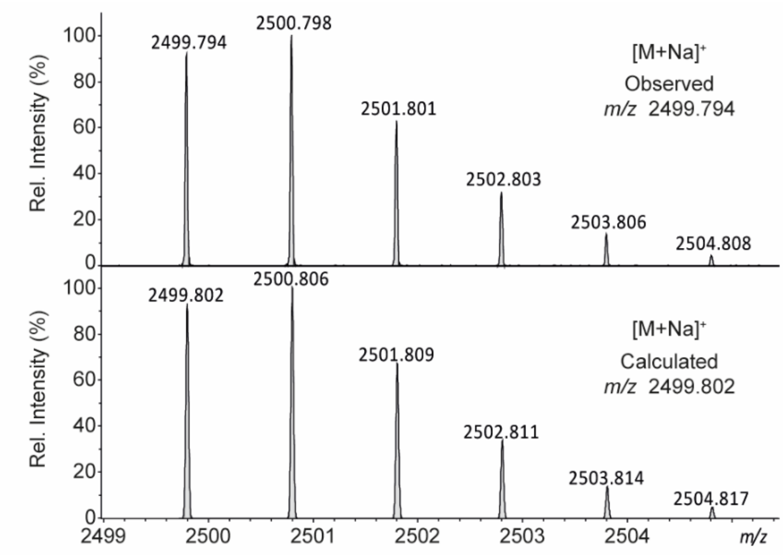

MALDI-FT-ICR MS spectrum of compound 7F

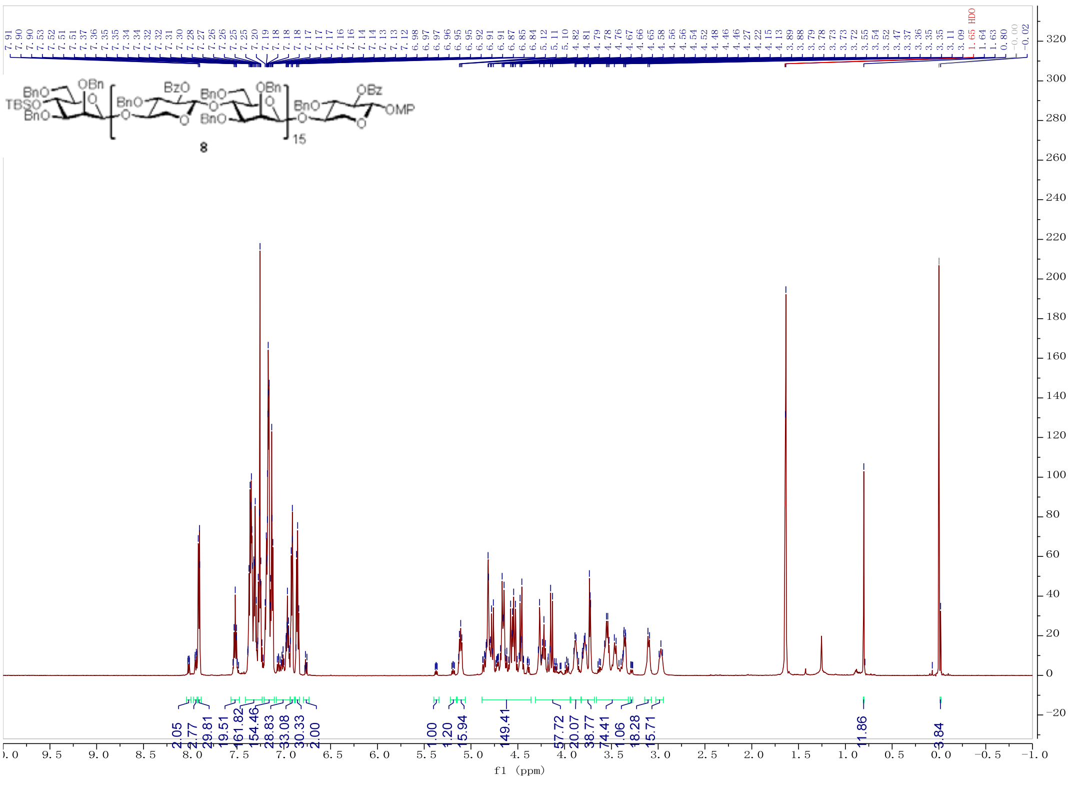

^{1}H NMR spectrum of 8 (600 MHz, $CDCl_3$, 25 °C)

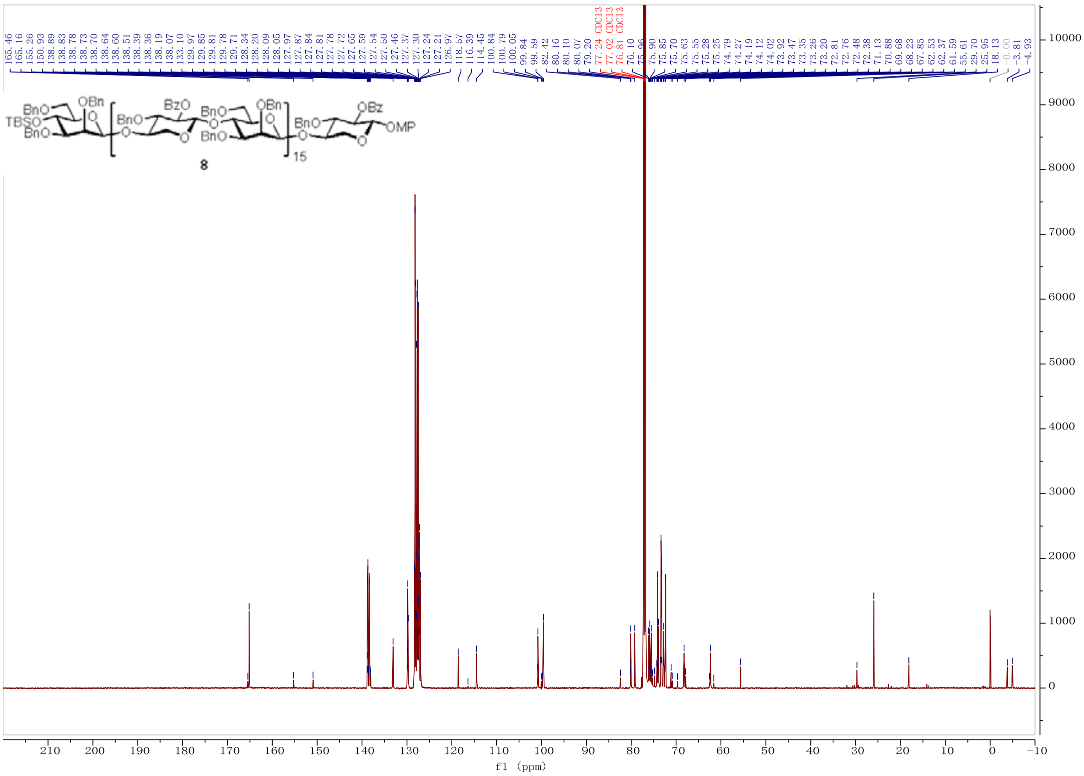

^{13}C NMR spectrum of 8 (151 MHz, $CDCl_3$, 25 °C)

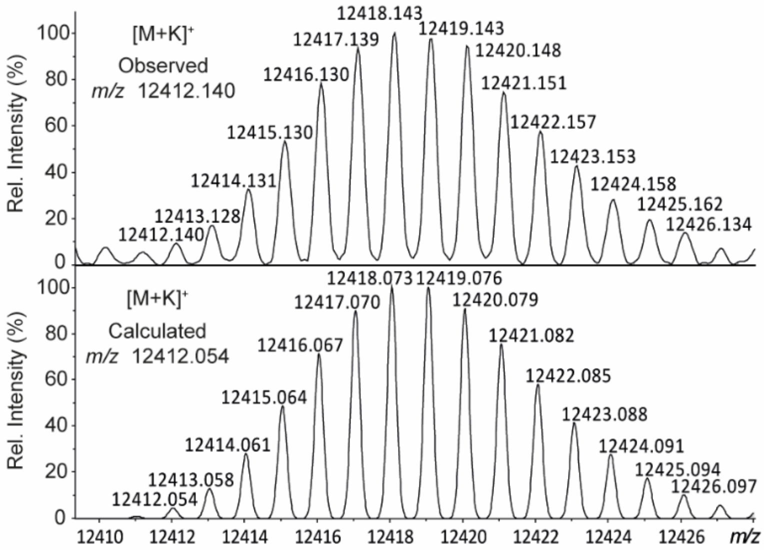

MALDI-FT-ICR MS spectrum of compound 8

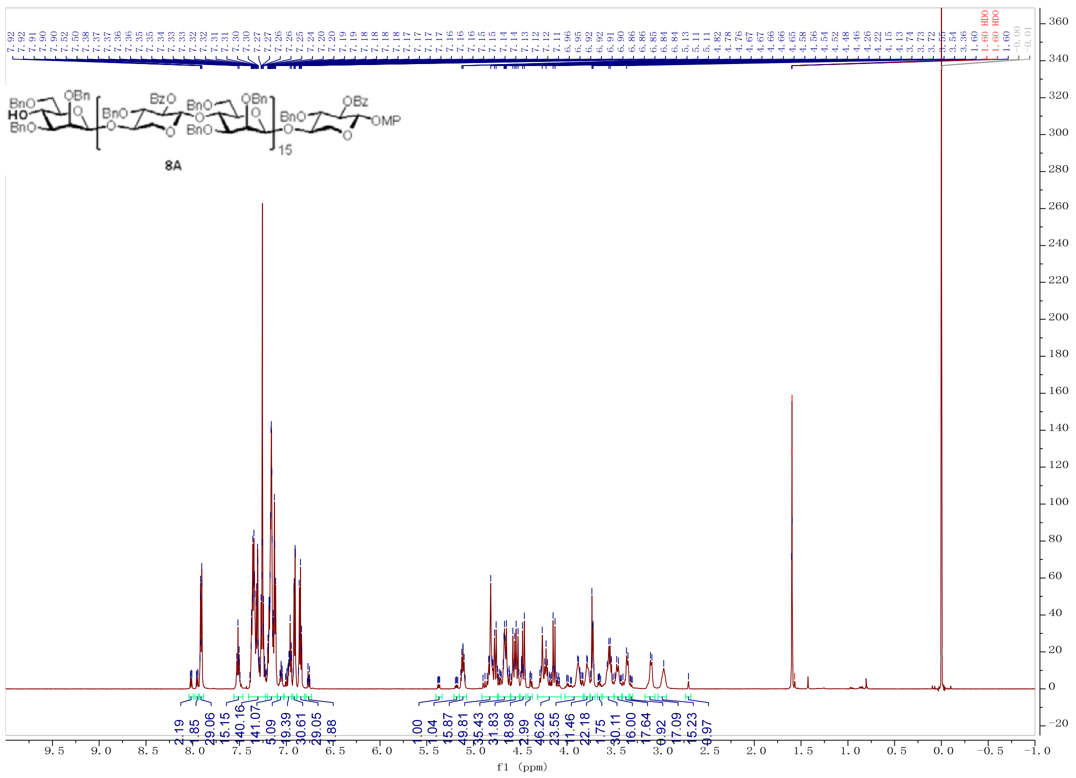

^{1}H NMR spectrum of 8A (600 MHz, $CDCl_3$, 25 °C)

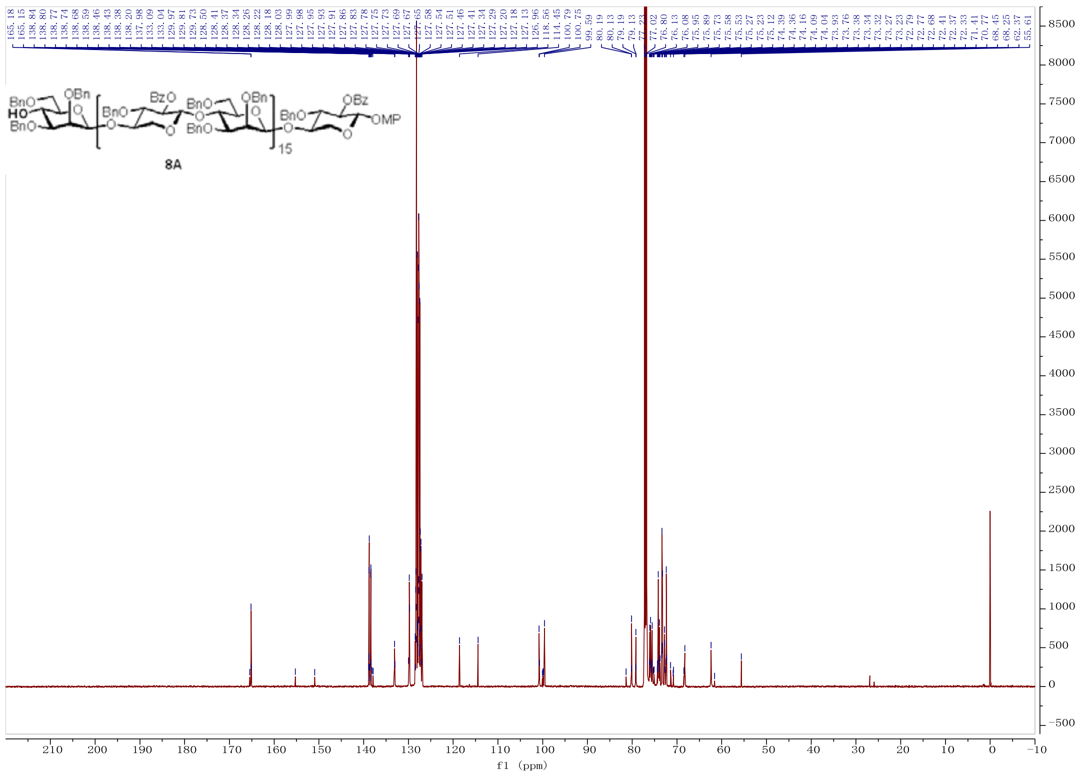

^{13}C NMR spectrum of 8A (151 MHz, $CDCl_3$, 25 °C)

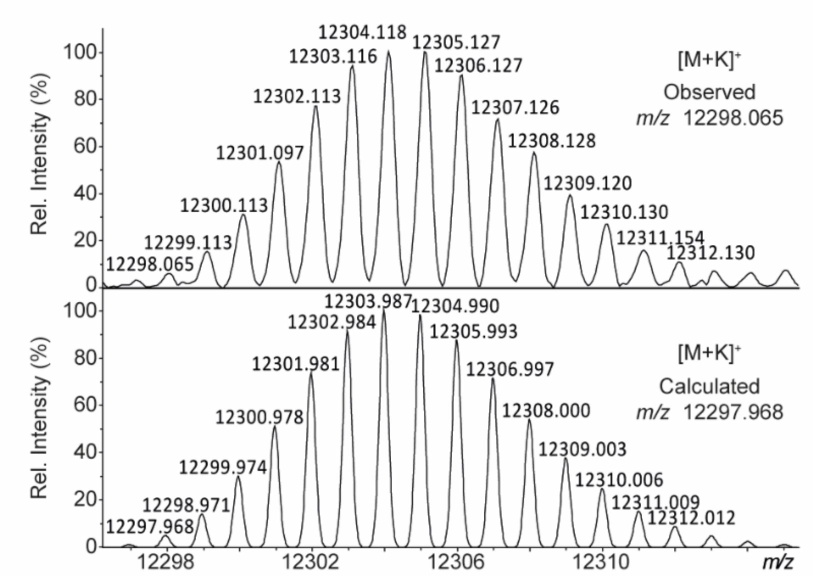

MALDI-FT-ICR MS spectrum of compound 8A

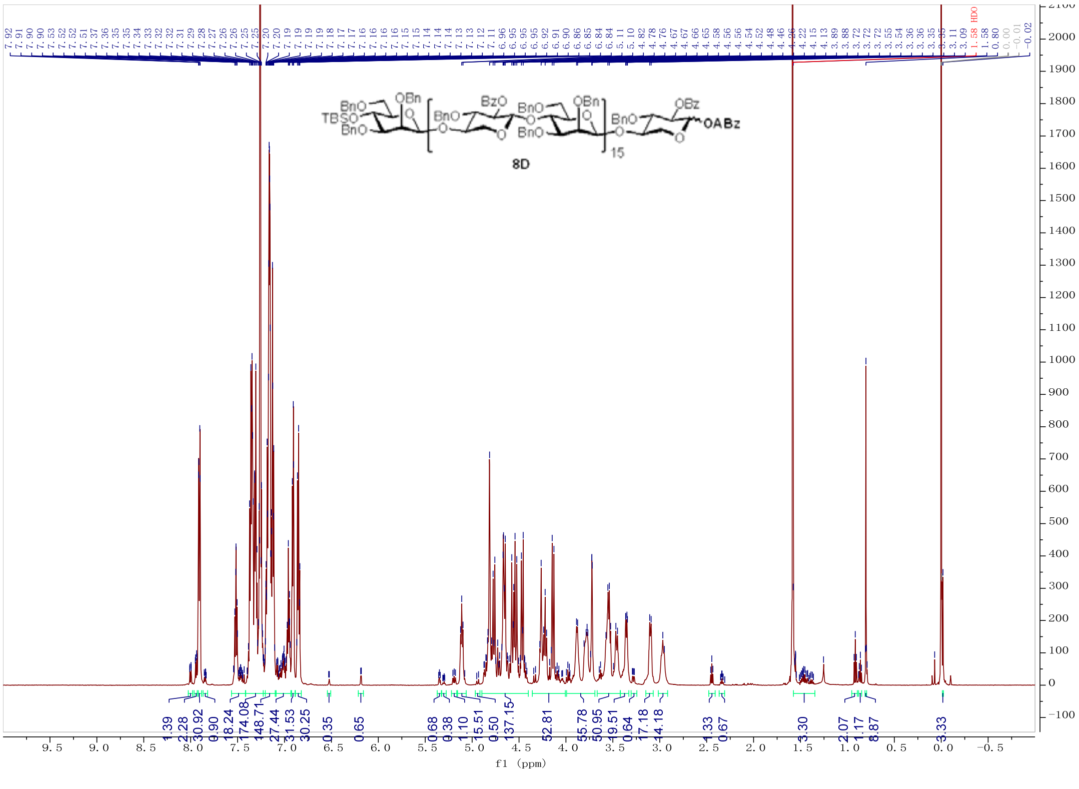

^{1}H NMR spectrum of 8D (600 MHz, $CDCl_3$, 25 °C)

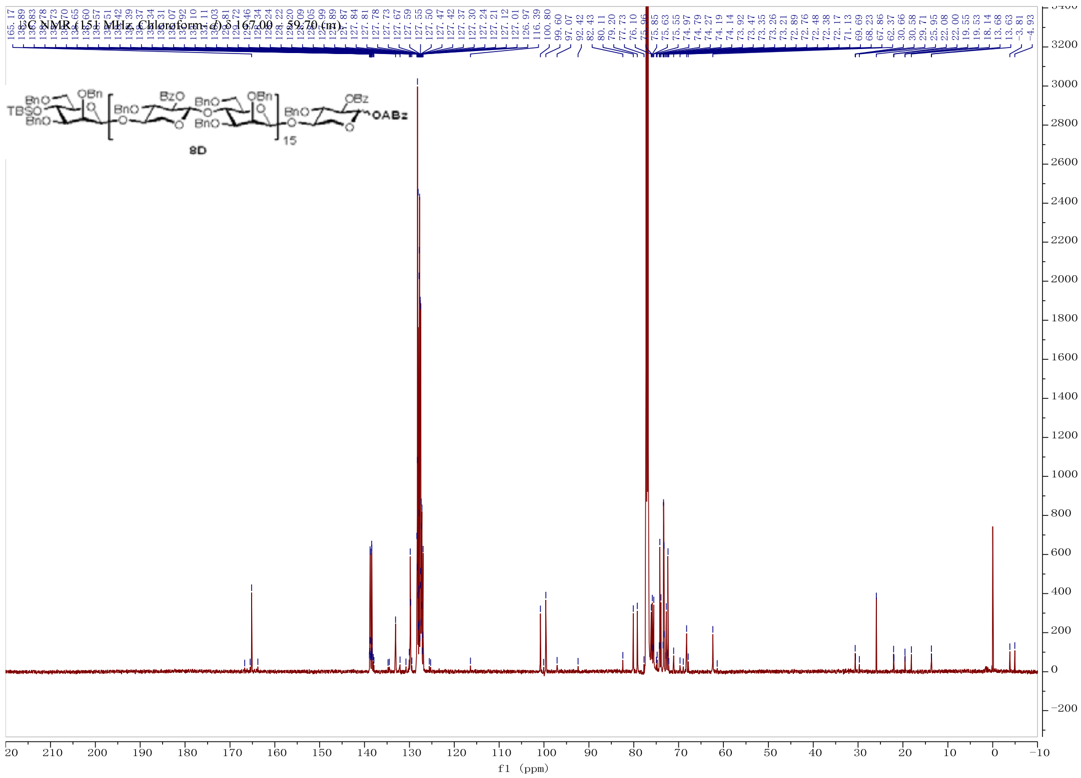

^{13}C NMR spectrum of 8D (151 MHz, $CDCl_3$, 25 °C)

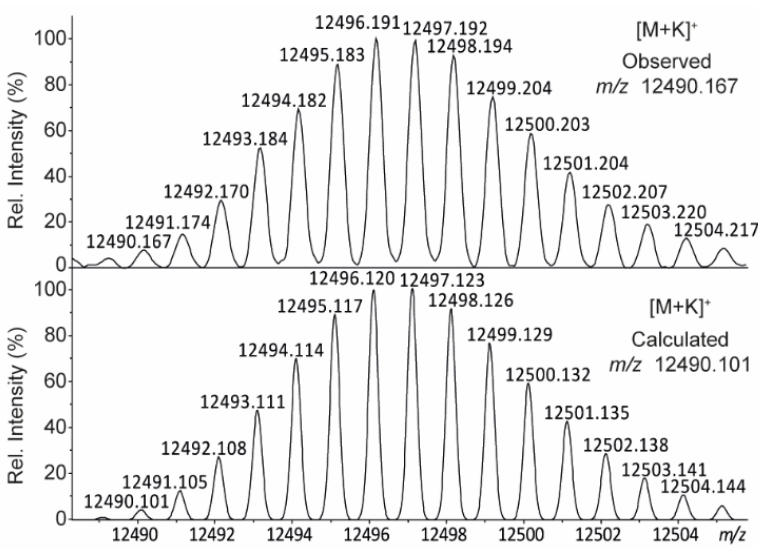

MALDI-FT-ICR MS spectrum of compound 8D

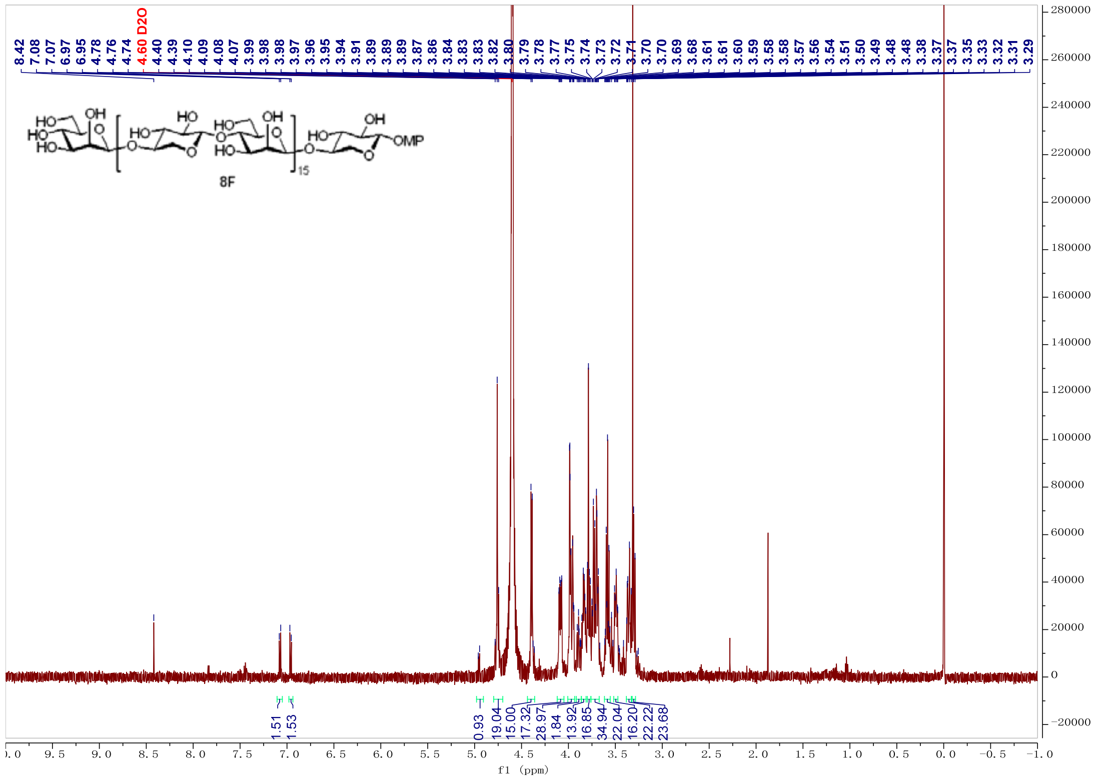

^{1}H NMR spectrum of compound 8F (600 MHz, 20 mM Na_3PO_4 buffered D_2O, 40 °C)

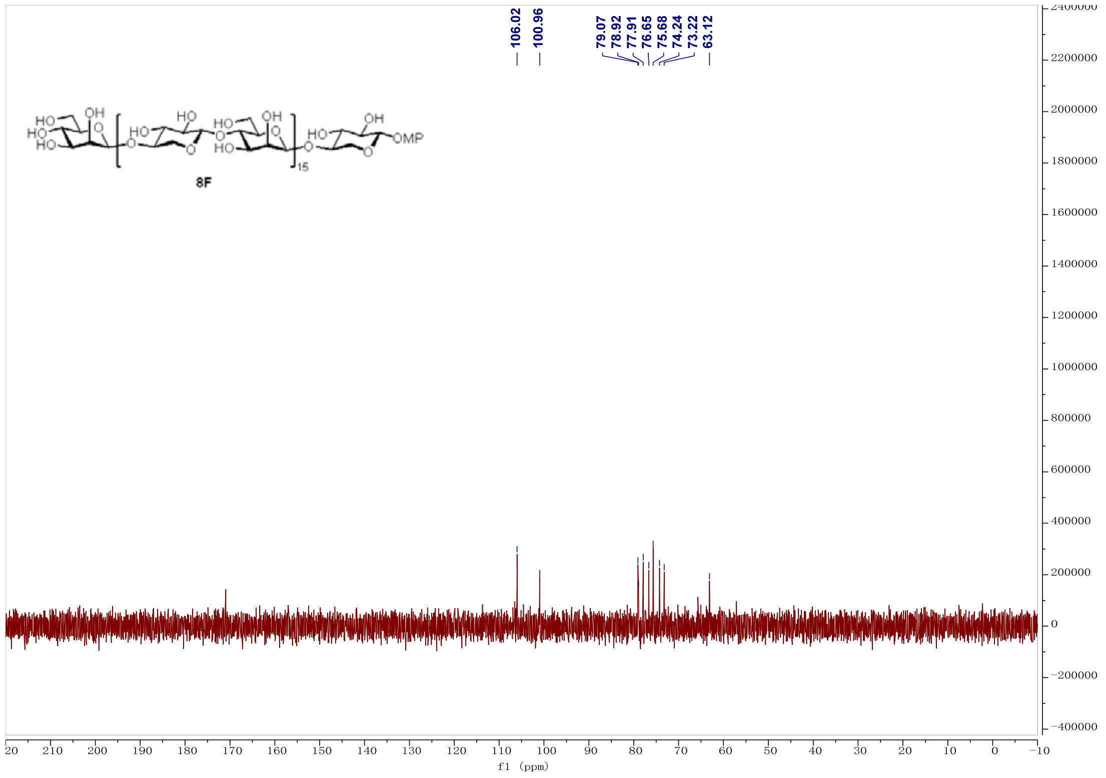

^{13}C NMR spectrum of compound 8F (151 MHz, 20 mM Na_3PO_4 buffered D_2O, 40 °C)

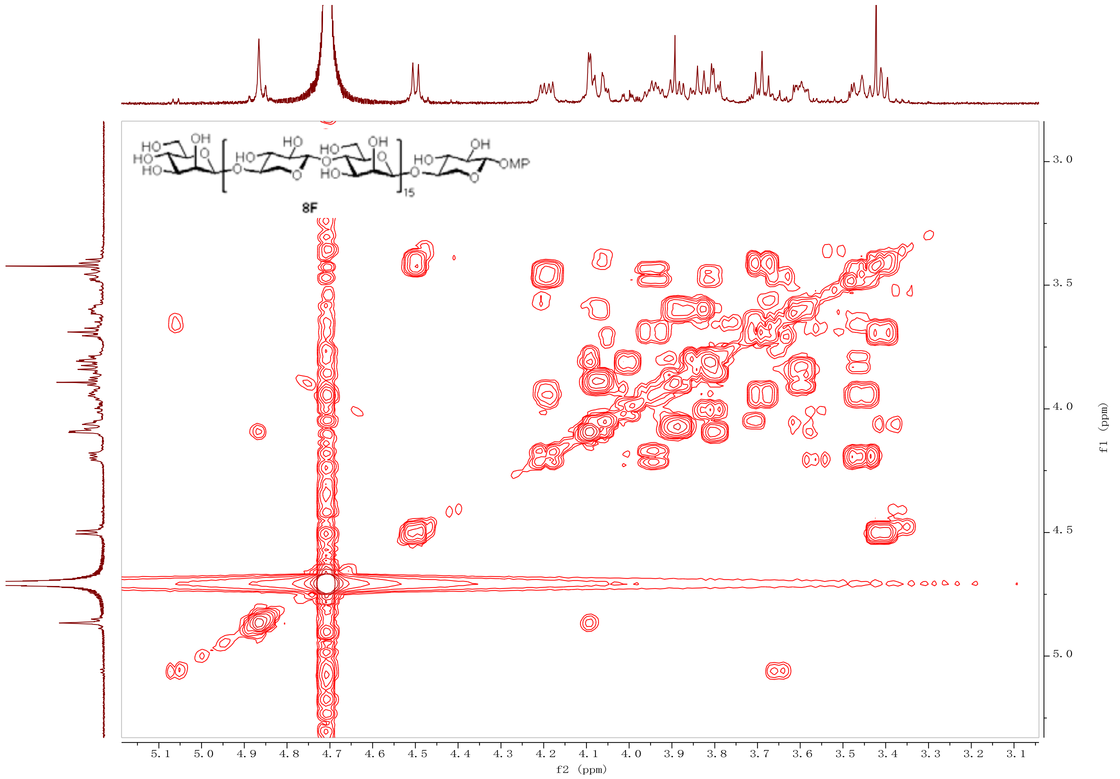

^{1}H-^{1}H gCOSY spectrum of compound 8F (600 MHz, 20 mM Na_3PO_4 buffered D_2O, 40 °C)

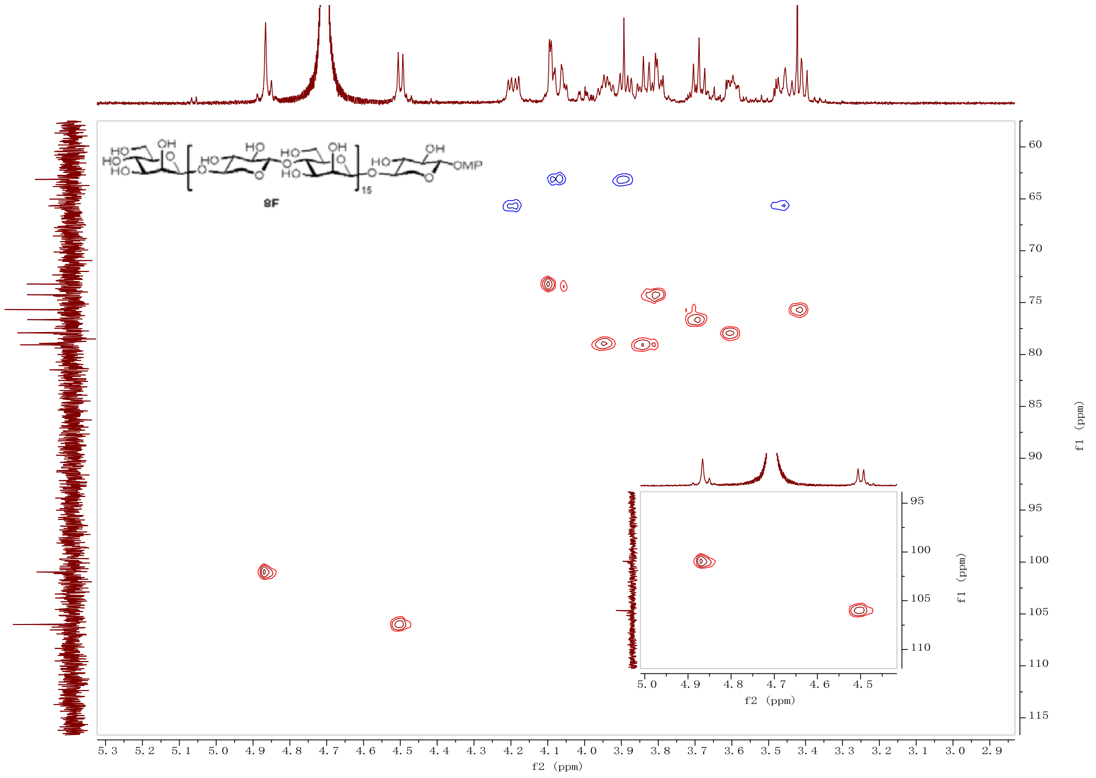

^{1}H-^{13}C HSQC spectrum of compound 8F (600 MHz, 20 mM Na_3PO_4 buffered D_2O, 40 °C)

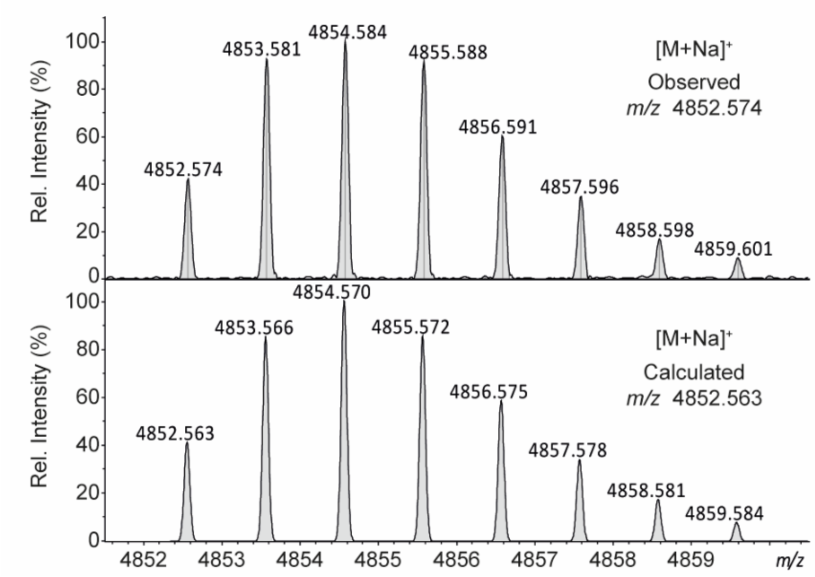

MALDI-FT-ICR MS spectrum of compound 8F

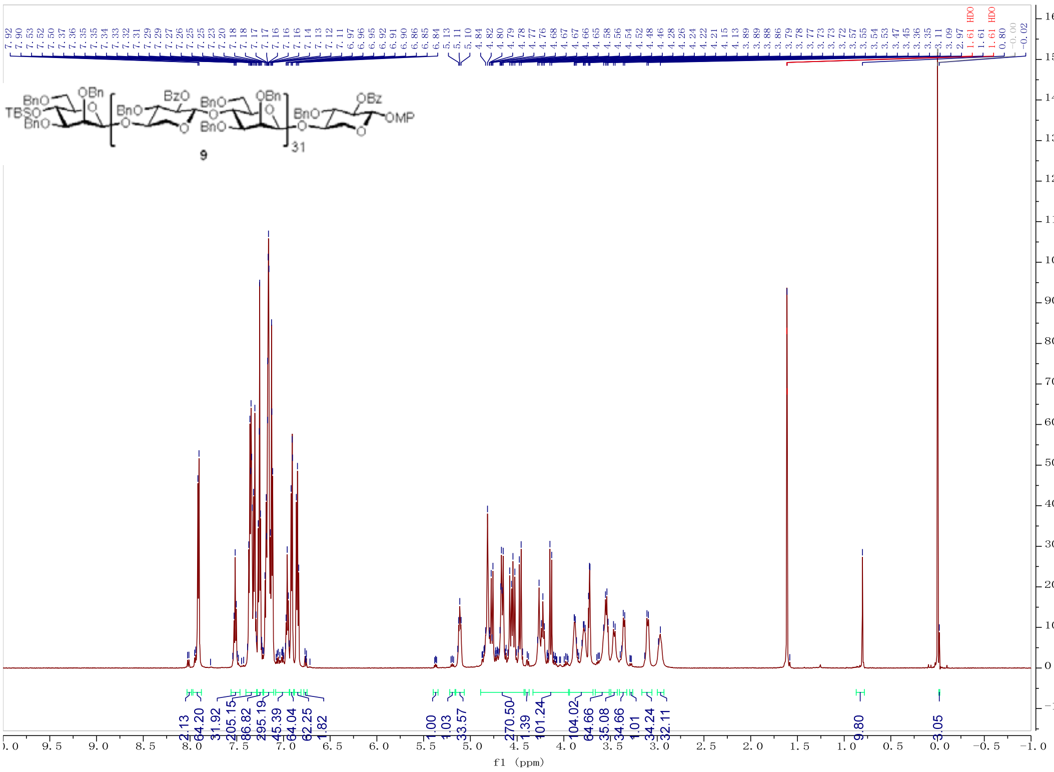

^{1}H NMR spectrum of 9 (600 MHz, $CDCl_3$, 25 °C)

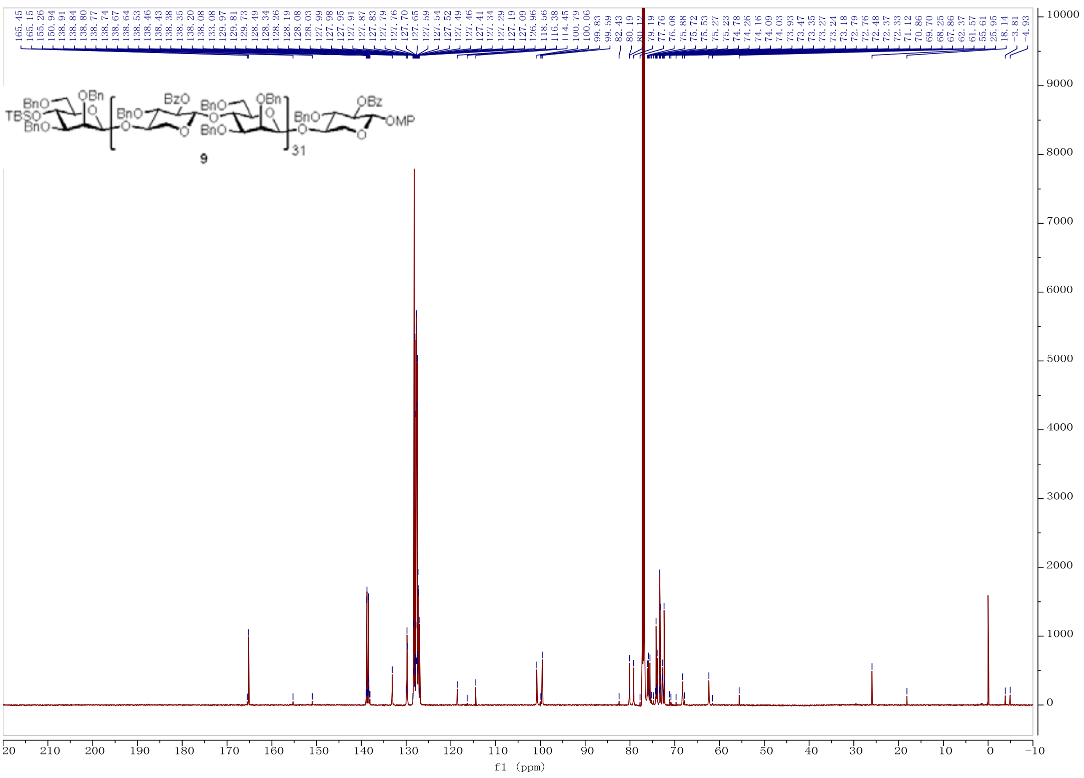

^{13}C NMR spectrum of 9 (151 MHz, $CDCl_3$, 25 °C)

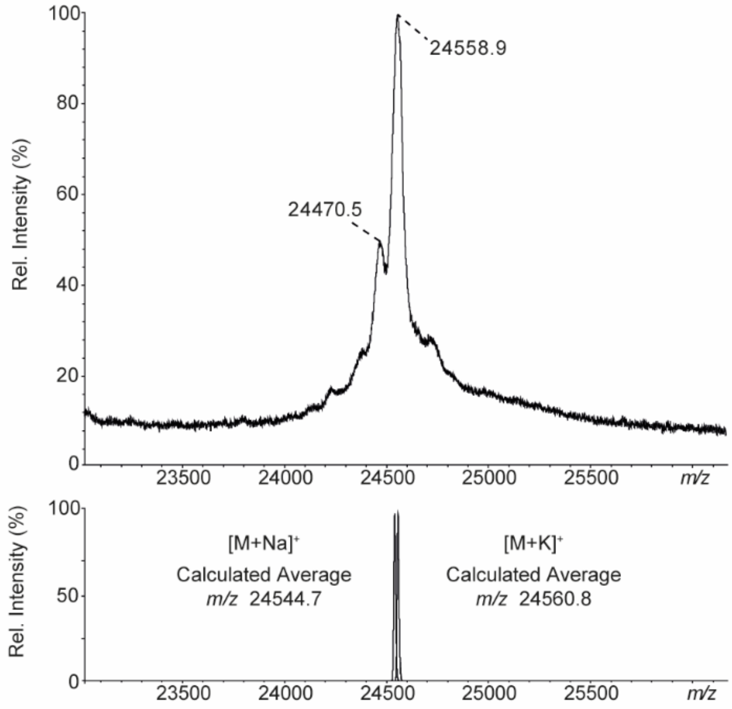

MALDI-FT-ICR MS spectrum of compound 9

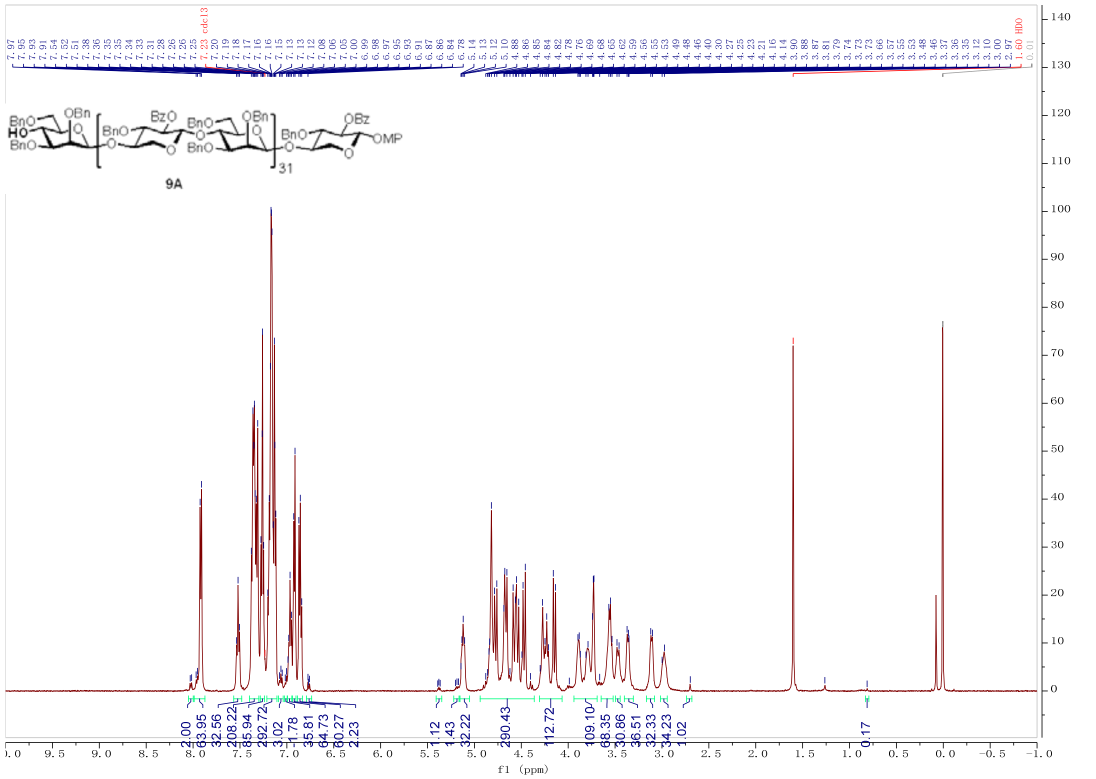

^{1}H NMR spectrum of 9A (600 MHz, $CDCl_3$, 25 °C)

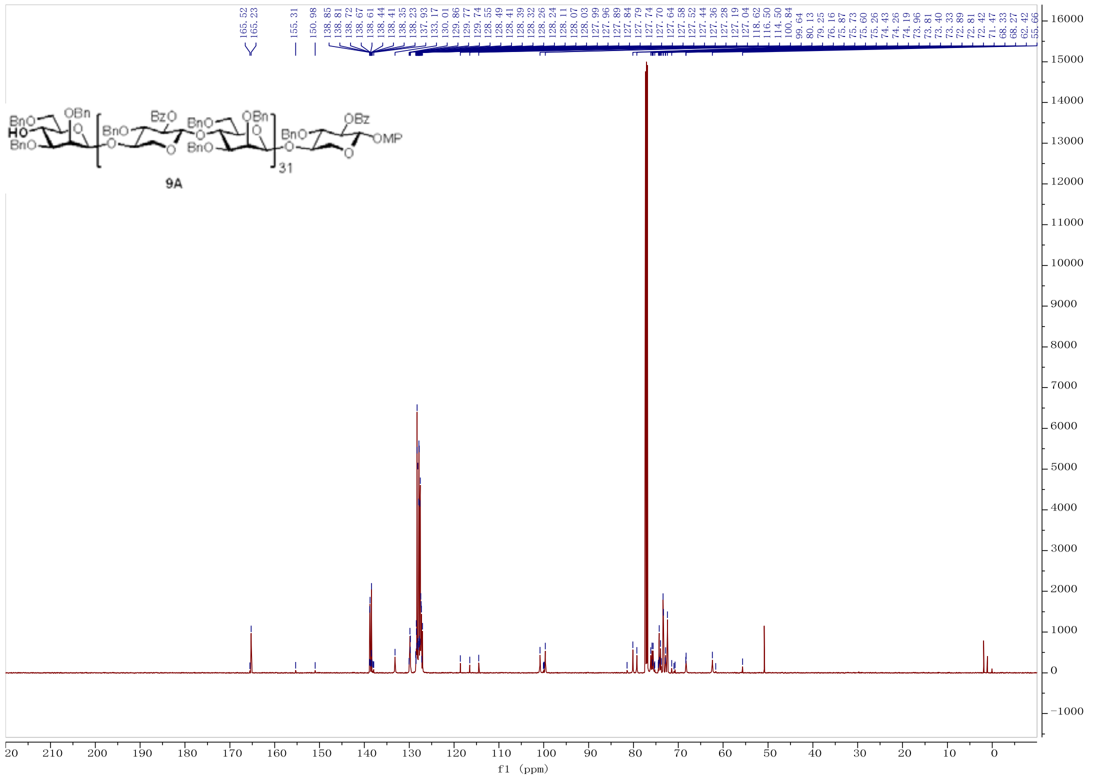

^{13}C NMR spectrum of 9A (151 MHz, $CDCl_3$, 25 °C)

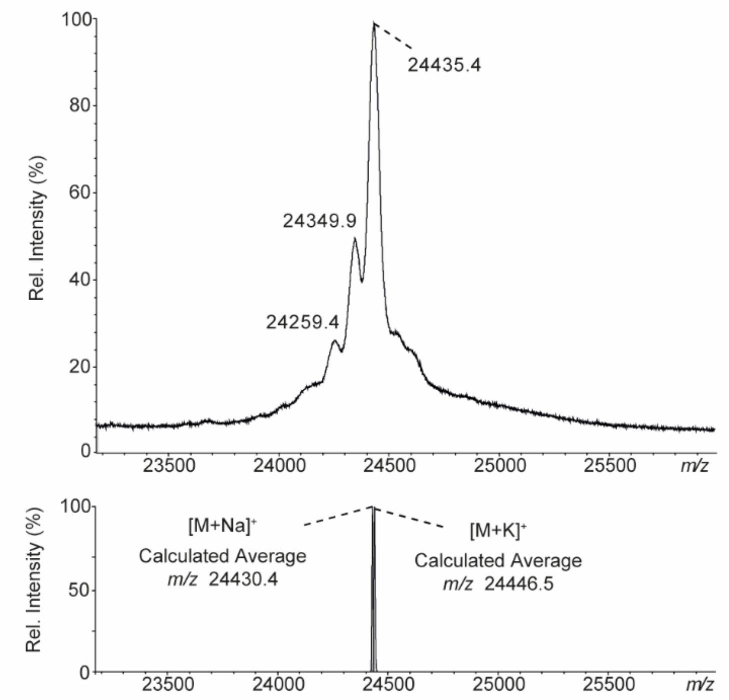

MALDI-FT-ICR MS spectrum of compound 9A

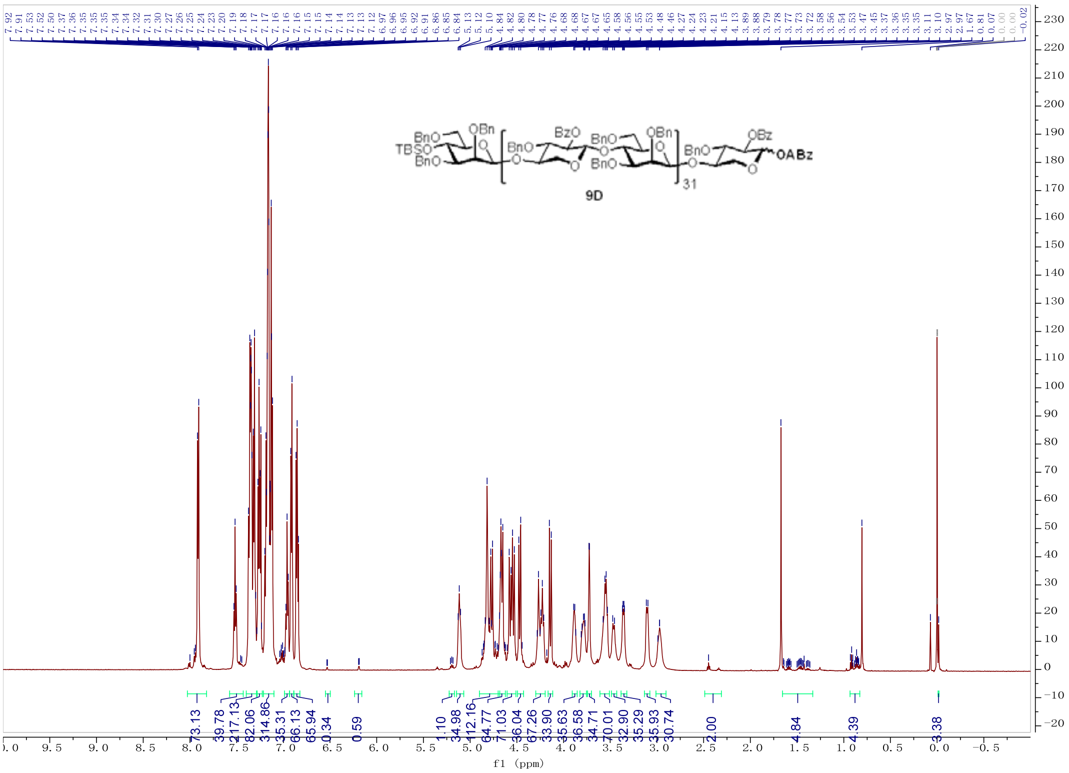

^{1}H NMR spectrum of 9D (600 MHz, $CDCl_3$, 25 °C)

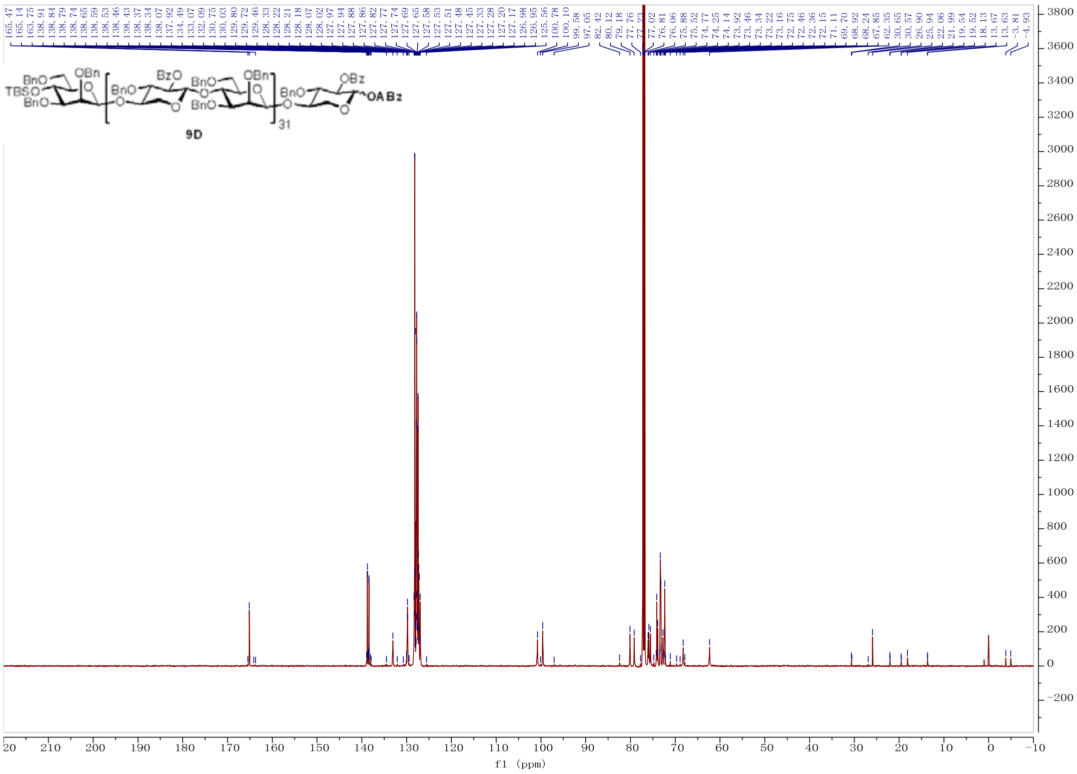

^{13}C NMR spectrum of 9D (151 MHz, $CDCl_3$, 25 °C)

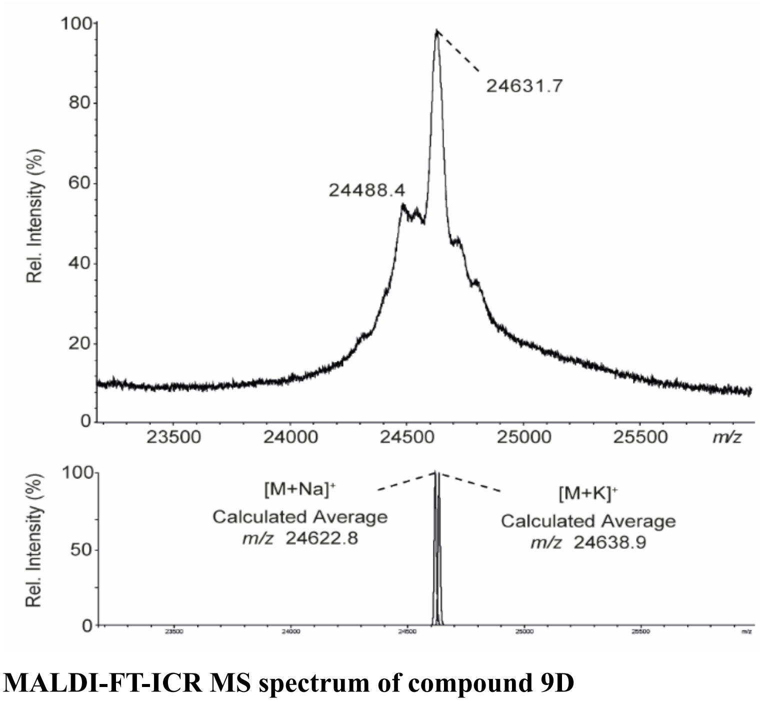

MALDI-FT-ICR MS spectrum of compound 9D

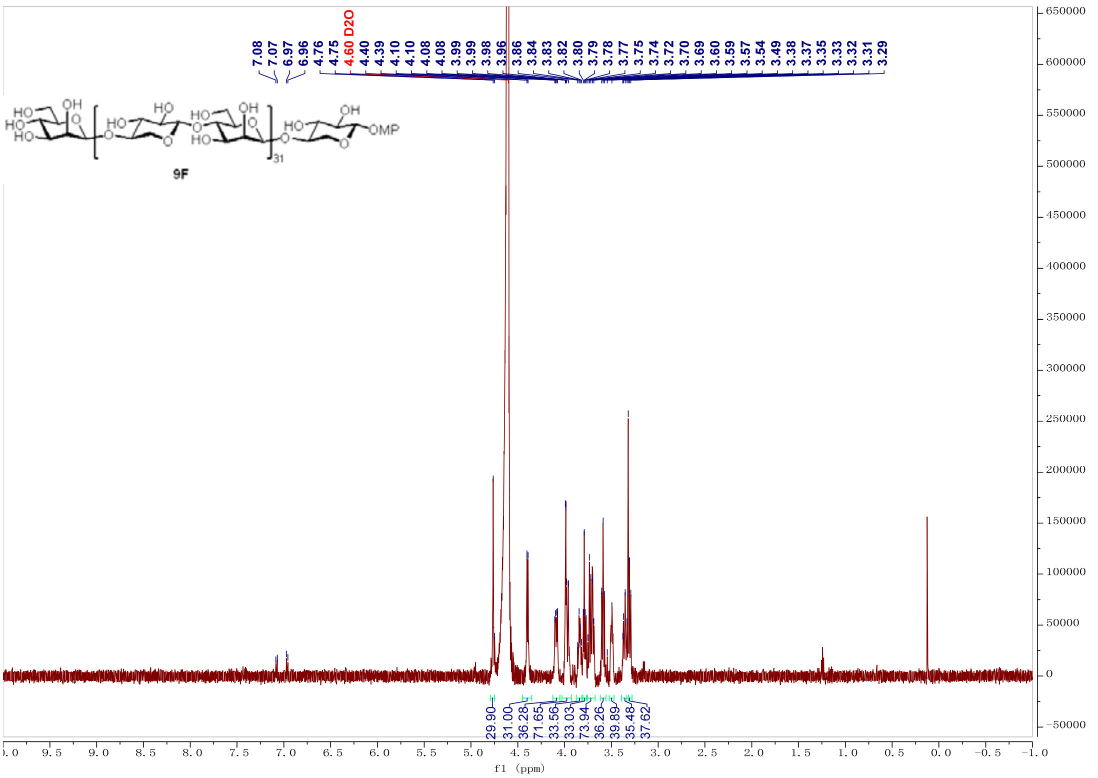

^{1}H NMR spectrum of compound 9F (600 MHz, 20 mM Na_3PO_4 buffered D_2O, 40 °C)

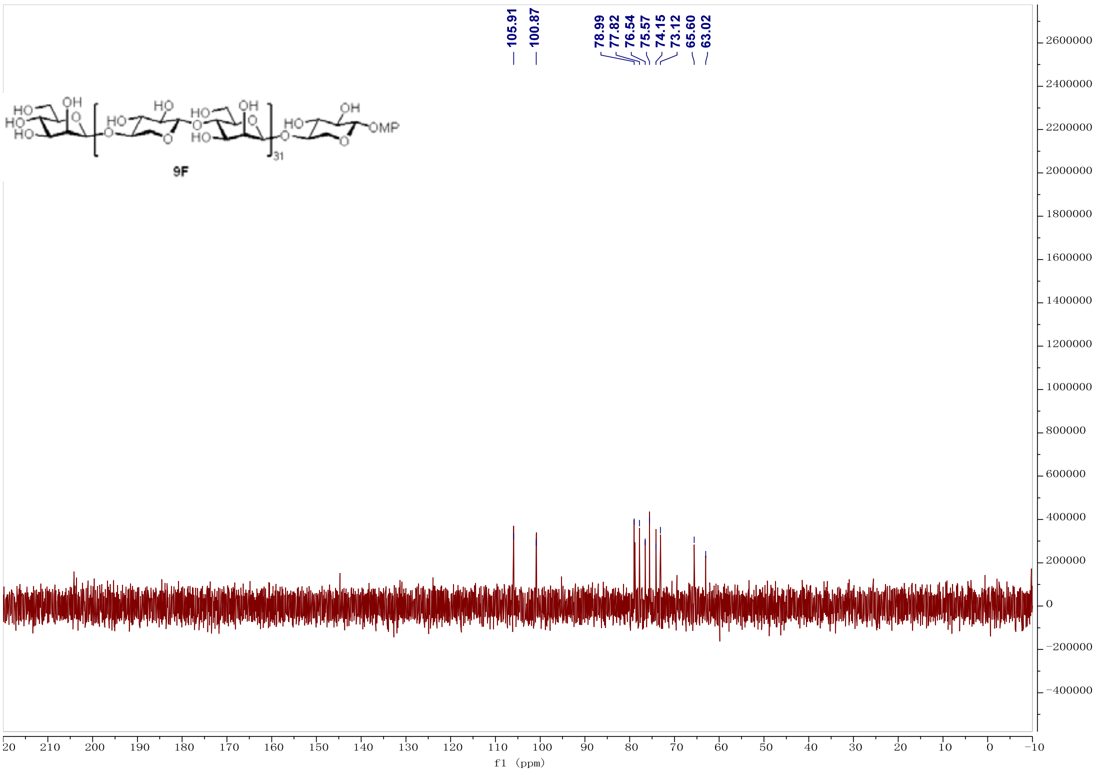

^{13}C NMR spectrum of compound 9F (151 MHz, 20 mM Na_3PO_4 buffered D_2O, 40 °C)

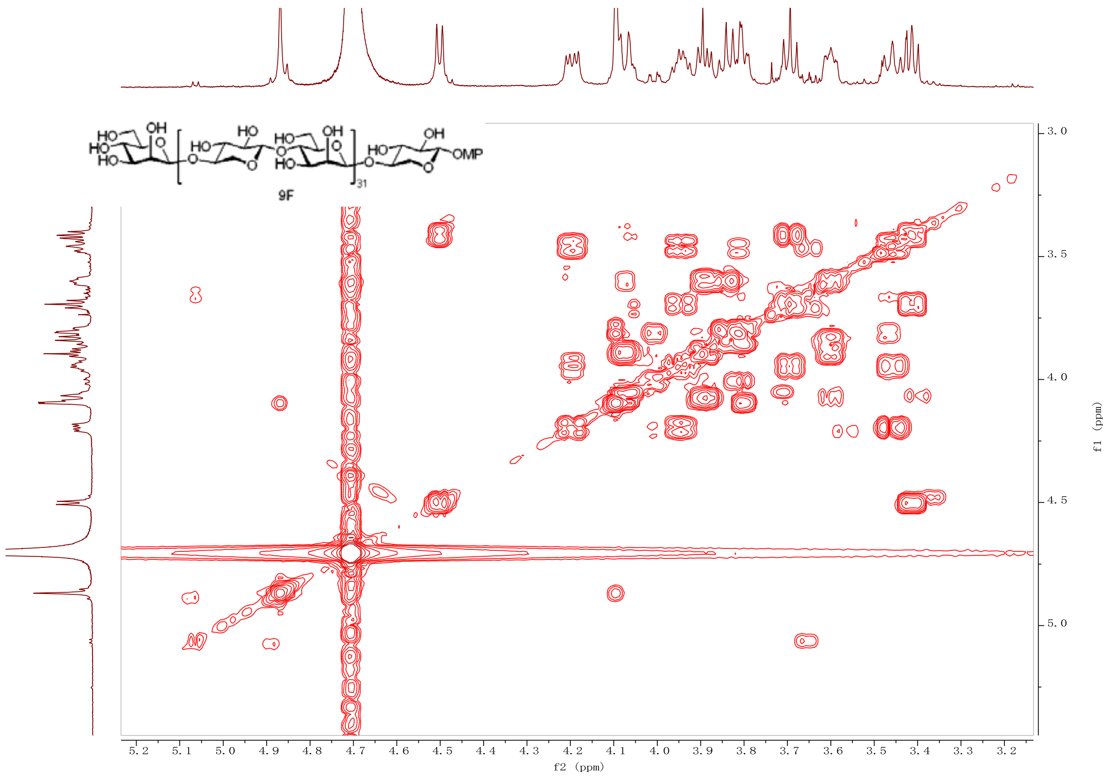

^{1}H-^{1}H gCOSY spectrum of compound 9F (600 MHz, 20 mM Na_3PO_4 buffered D_2O, 40 °C)

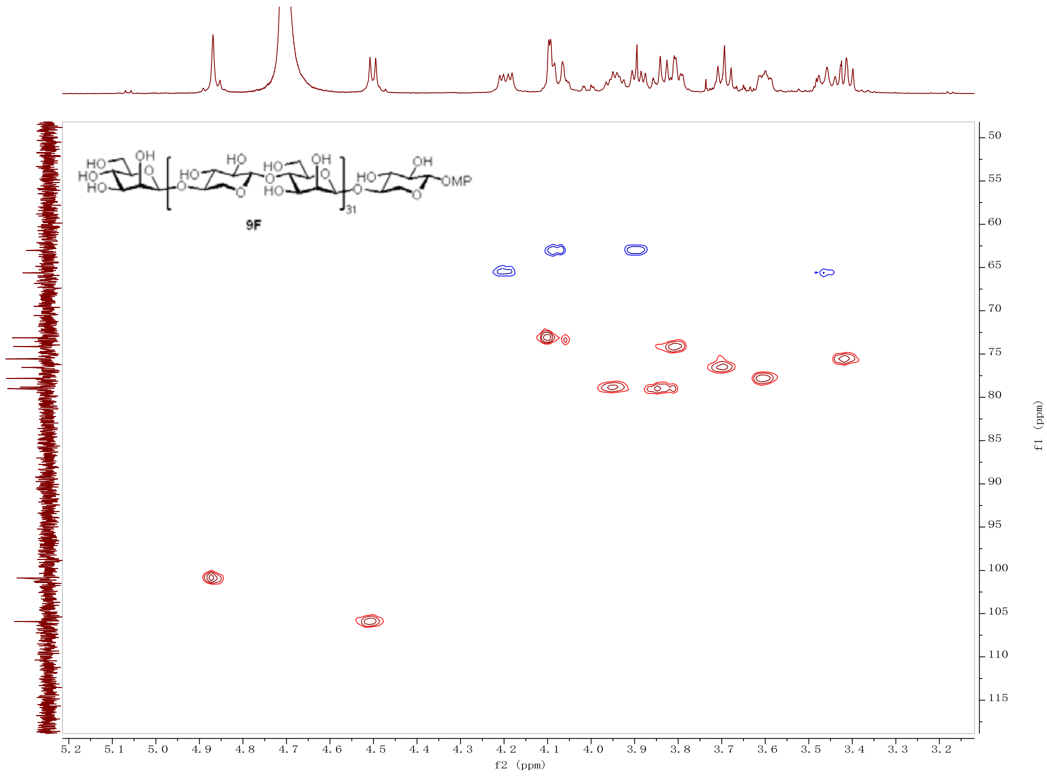

^{1}H-^{13}C HSQC spectrum of compound 9F (600 MHz, 20 mM Na_3PO_4 buffered D_2O, 40 °C)

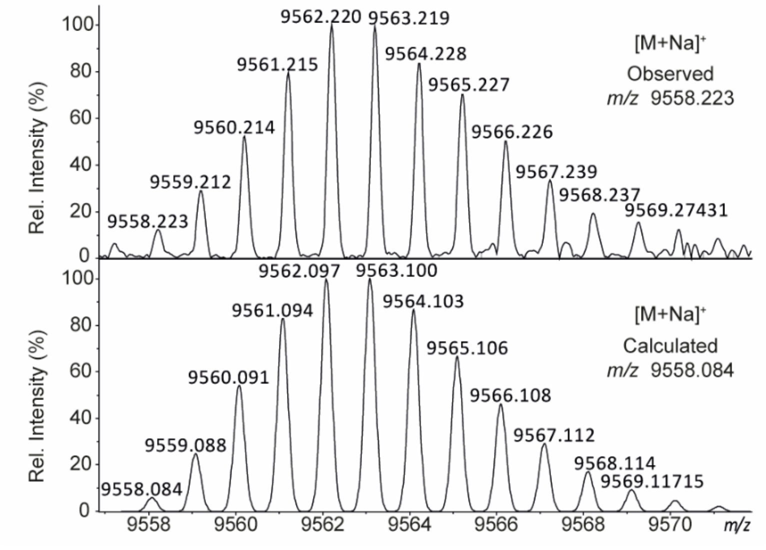

MALDI-FT-ICR MS spectrum of compound 9F

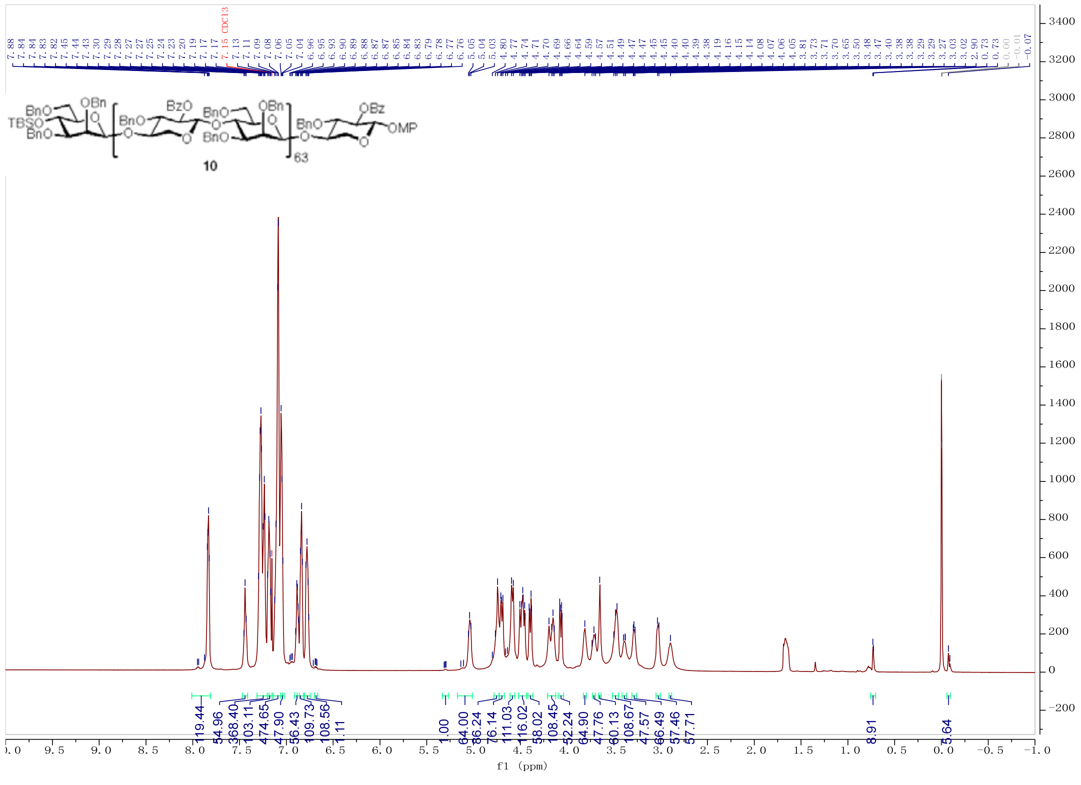

^{1}H NMR spectrum of 10 (600 MHz, $CDCl_3$, 25 °C)

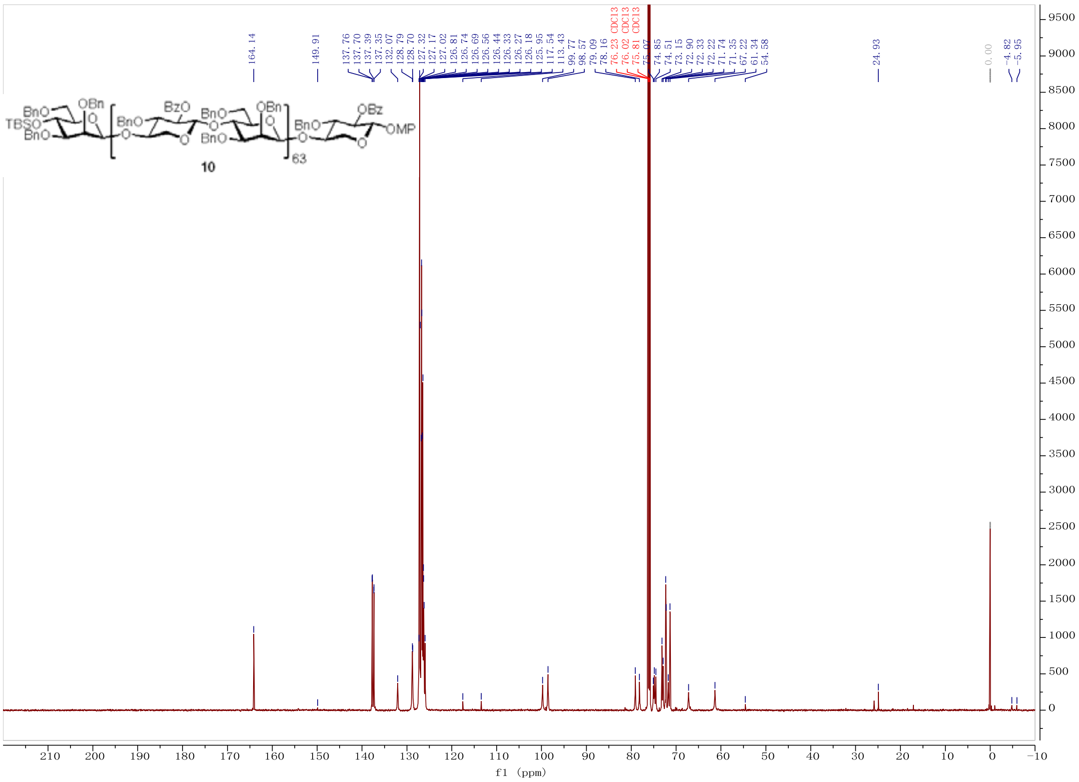

^{13}**C NMR spectrum of 10** (151 MHz, $CDCl_3$, 25 °C)

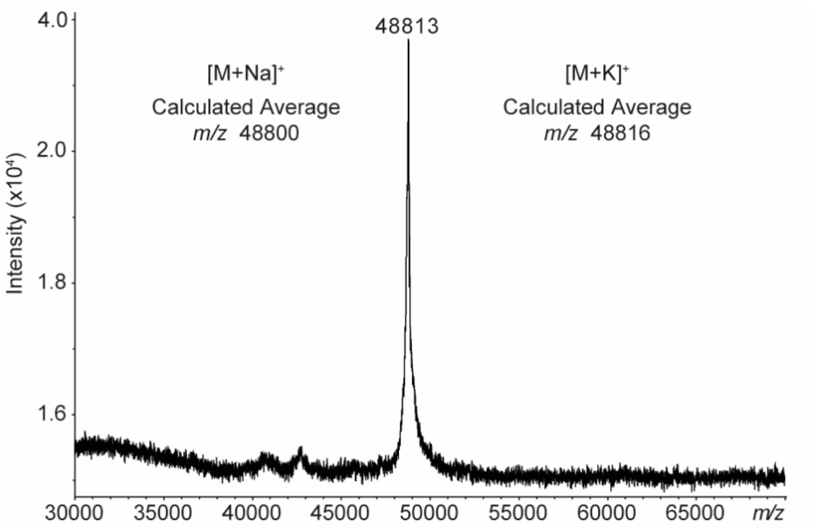

MALDI-FT-ICR MS spectrum of compound 10

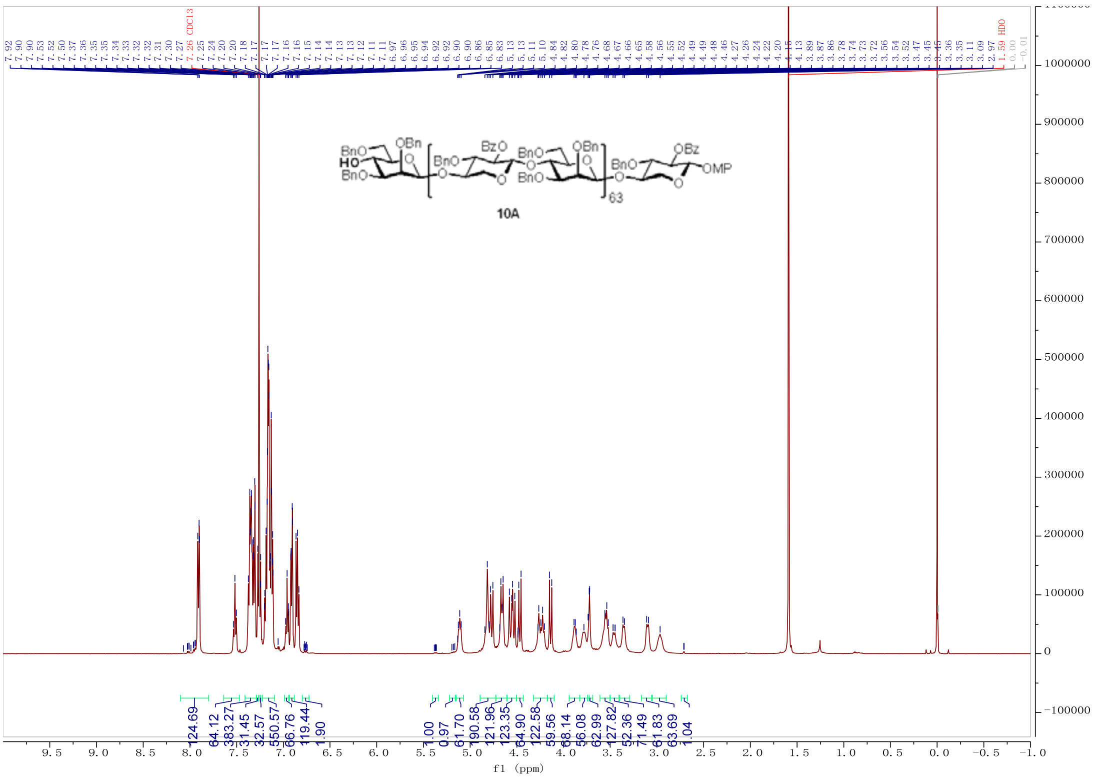

^{1}H NMR spectrum of 10A (600 MHz, $CDCl_3$, 25 °C)

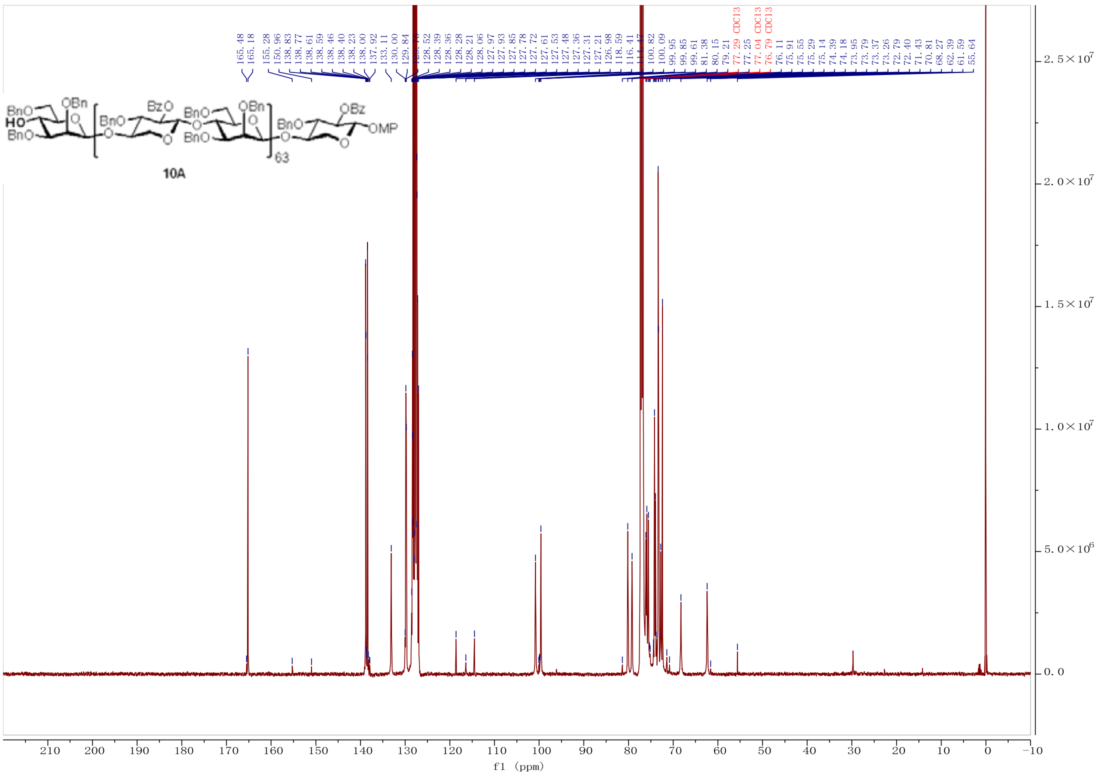

^{13}C NMR spectrum of 10A (151 MHz, $CDCl_3$, 25 °C)

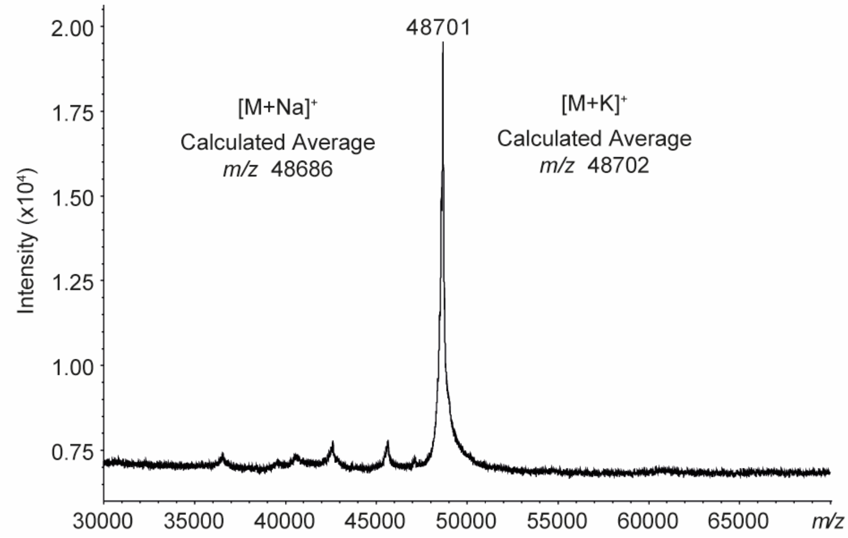

MALDI-FT-ICR MS spectrum of compound 10A

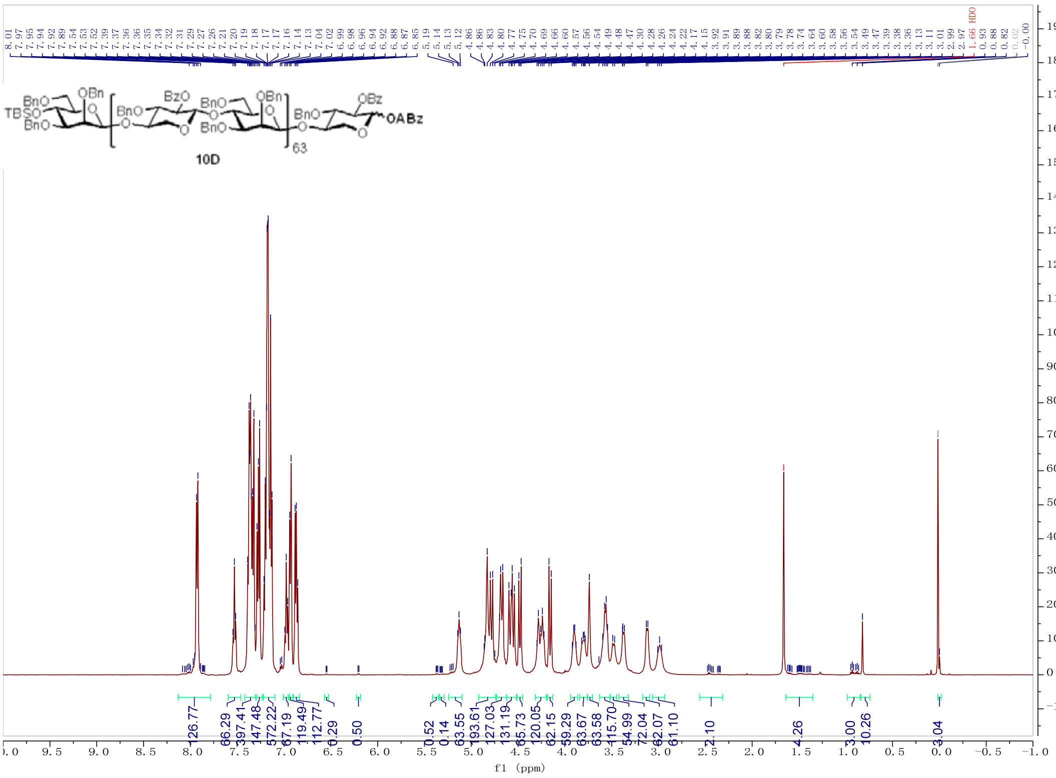

^{1}H NMR spectrum of 10D (600 MHz, $CDCl_3$, 25 °C)

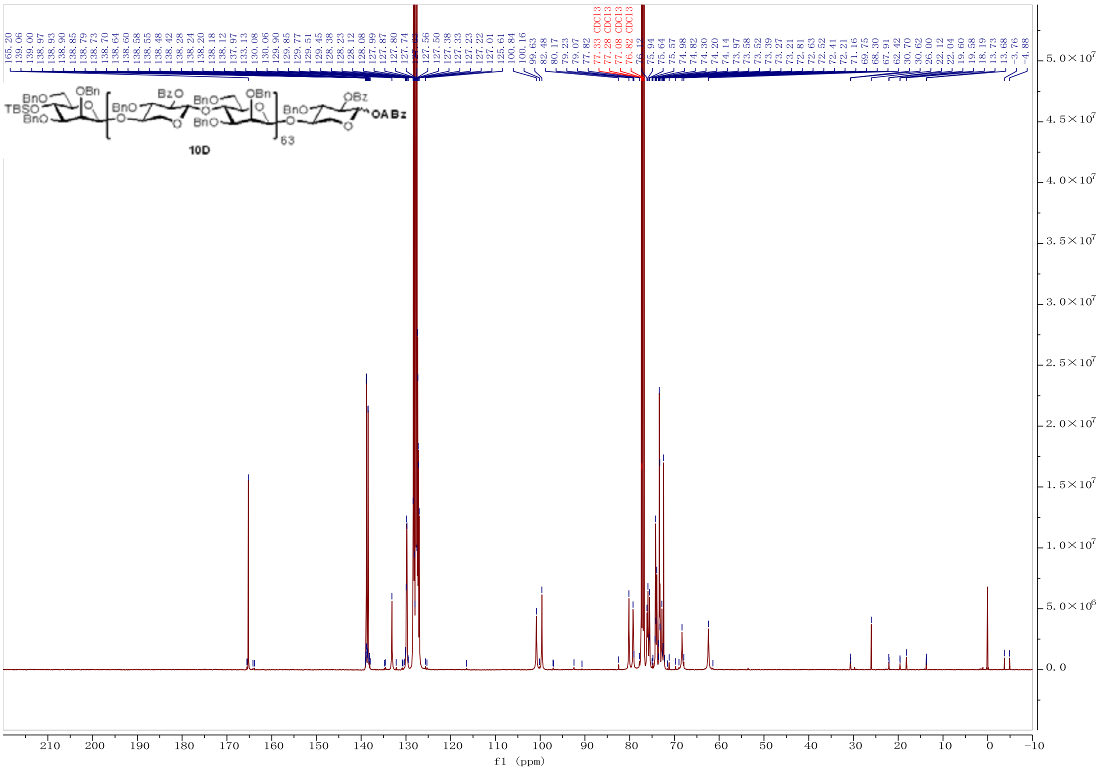

^{13}C NMR spectrum of 10D (151 MHz, $CDCl_3$, 25 °C)

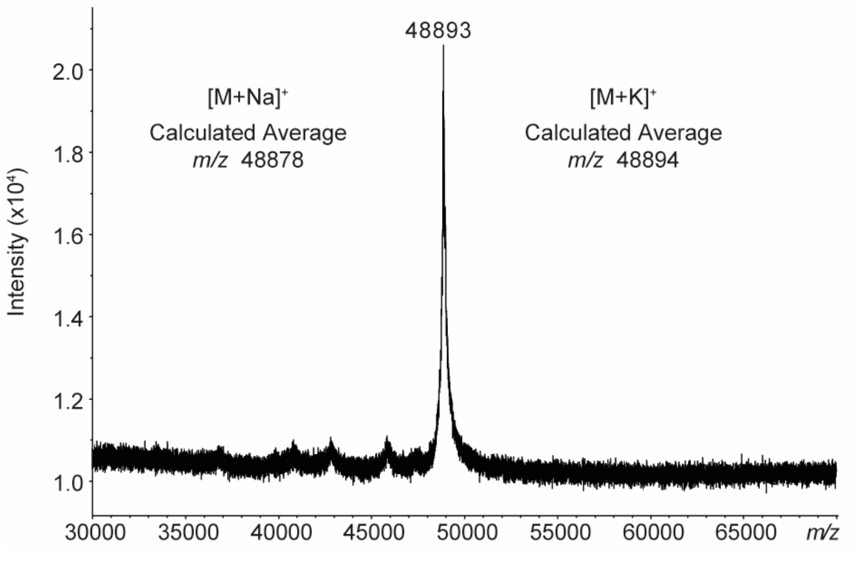

MALDI-FT-ICR MS spectrum of compound 10D

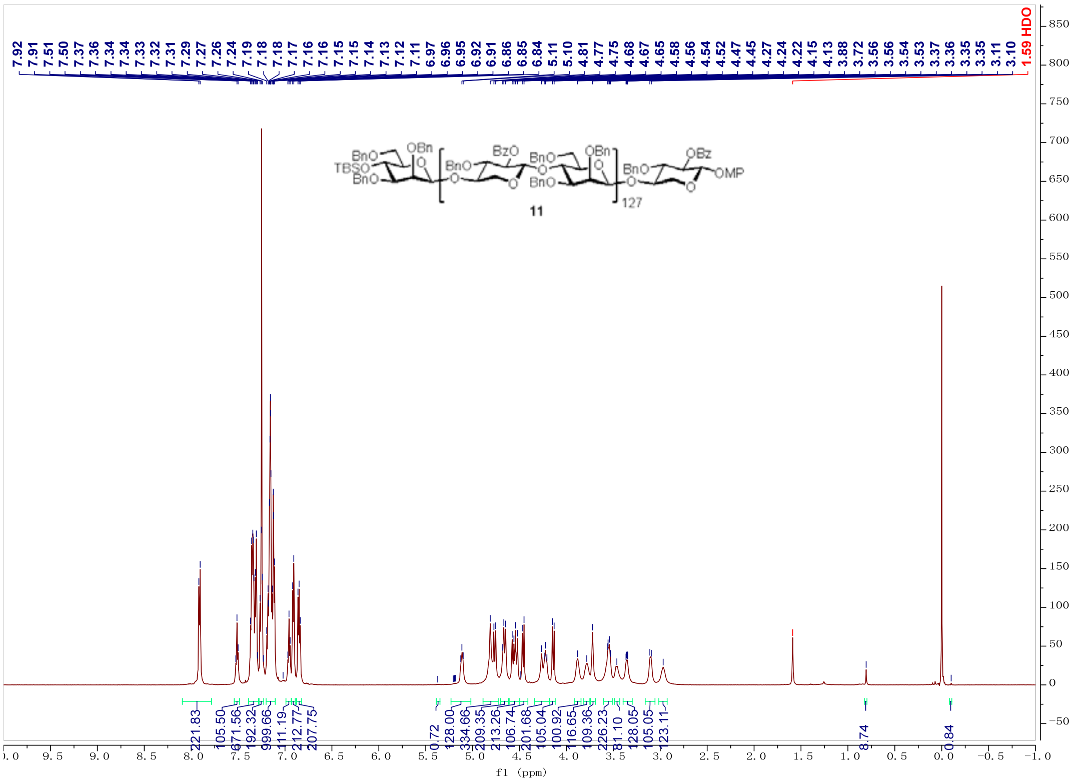

^{1}H NMR spectrum of 11 (600 MHz, $CDCl_3$, 25 °C)

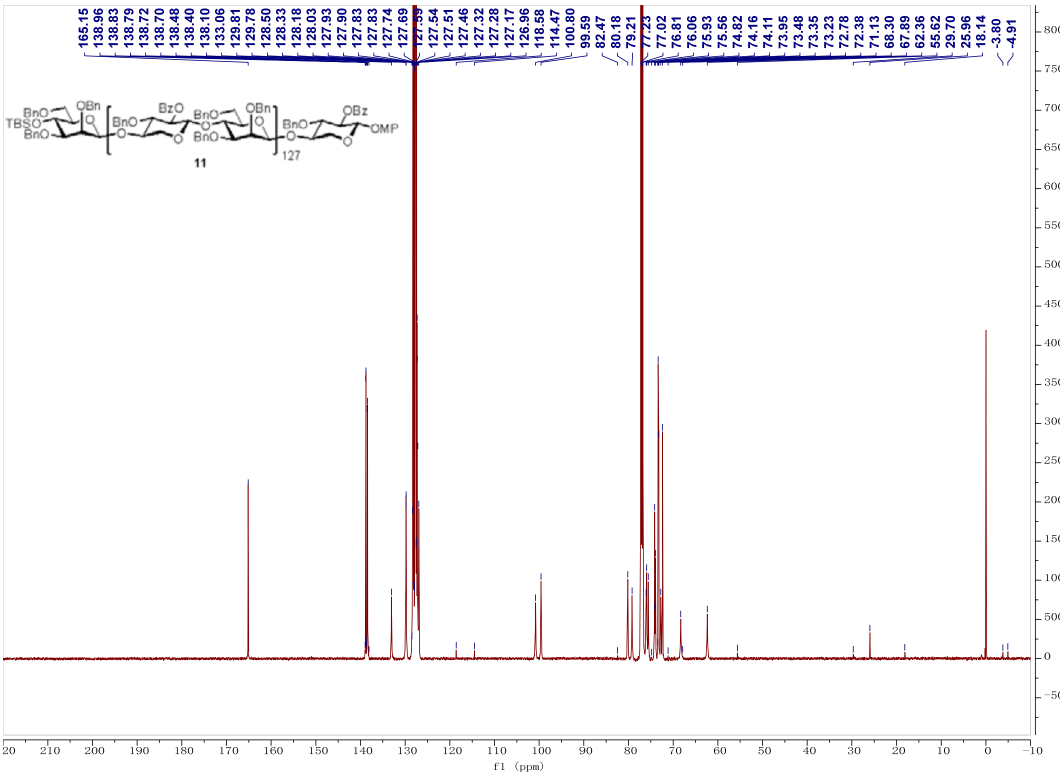

^{13}C NMR spectrum of 10D (151 MHz, $CDCl_3$, 25 °C)

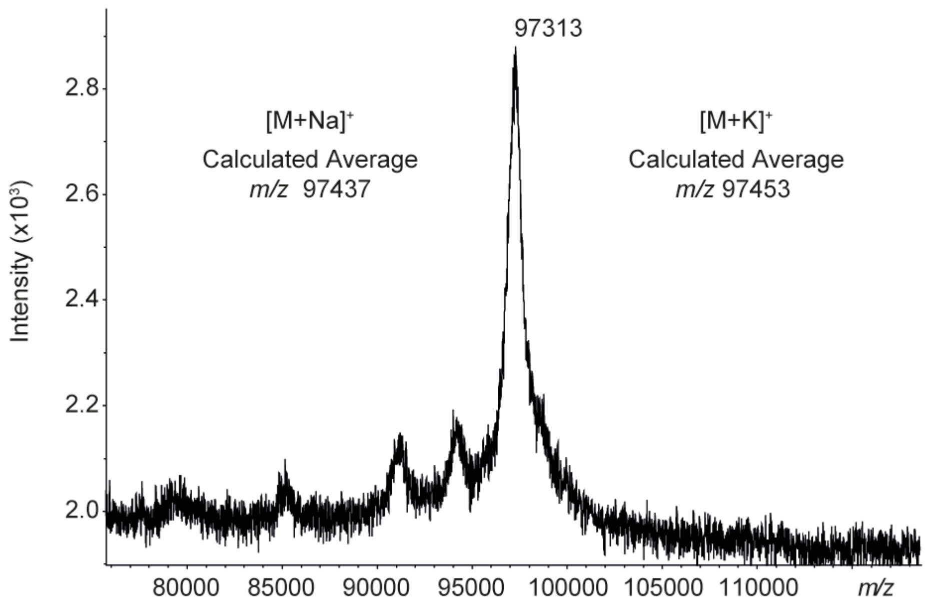

MALDI-FT-ICR MS spectrum of compound 11

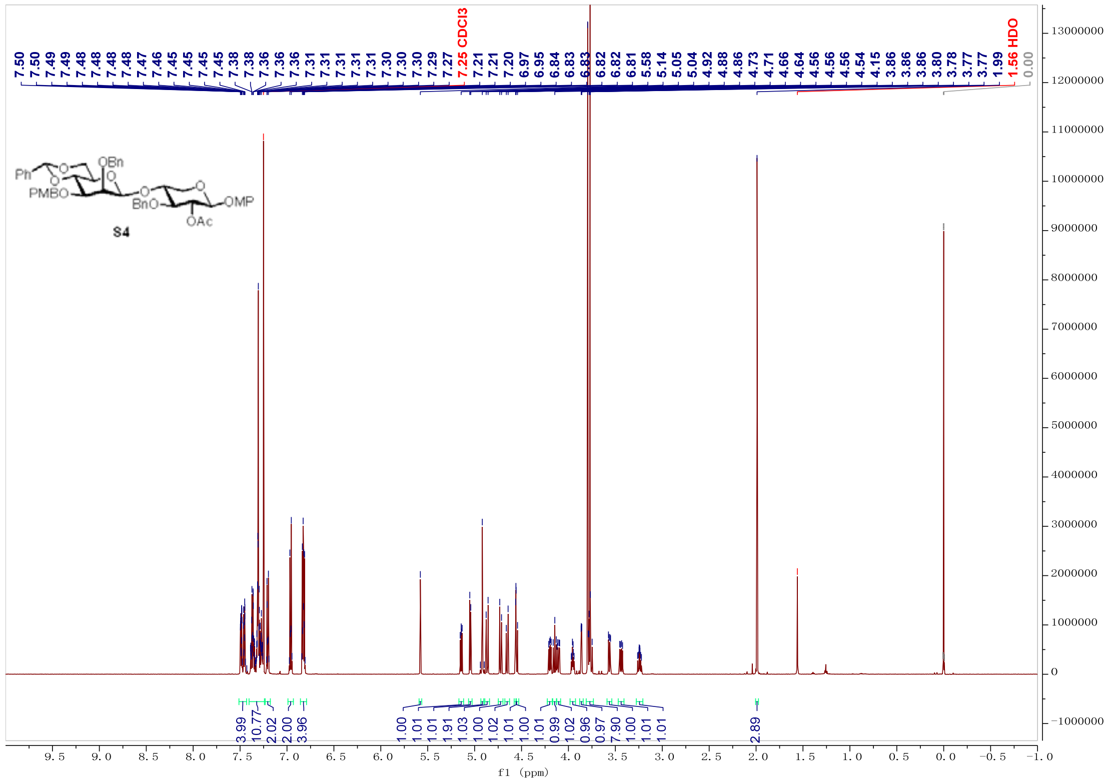

^{1}H NMR spectrum of S4 (600 MHz, $CDCl_3$, 25 °C)

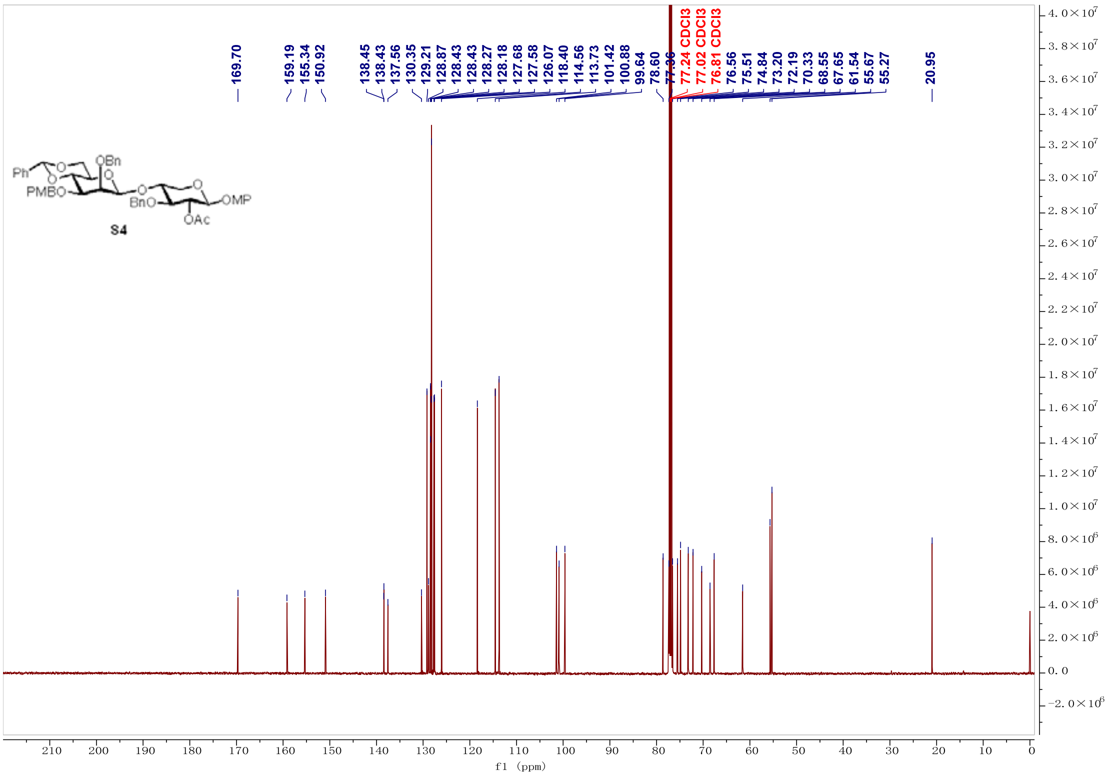

^{13}C NMR spectrum of S4 (151 MHz, $CDCl_3$, 25 °C)

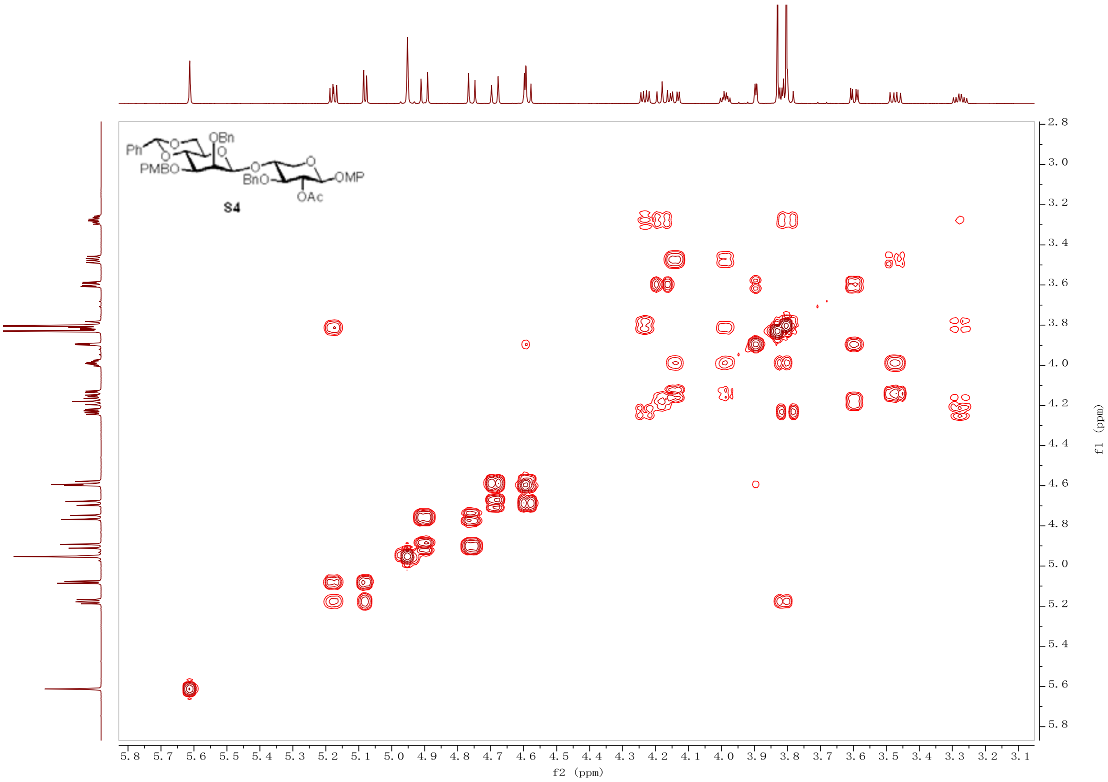

^{1}H-^{1}H gCOSY spectrum of S4 (600 MHz, $CDCl_3$, 25 °C)

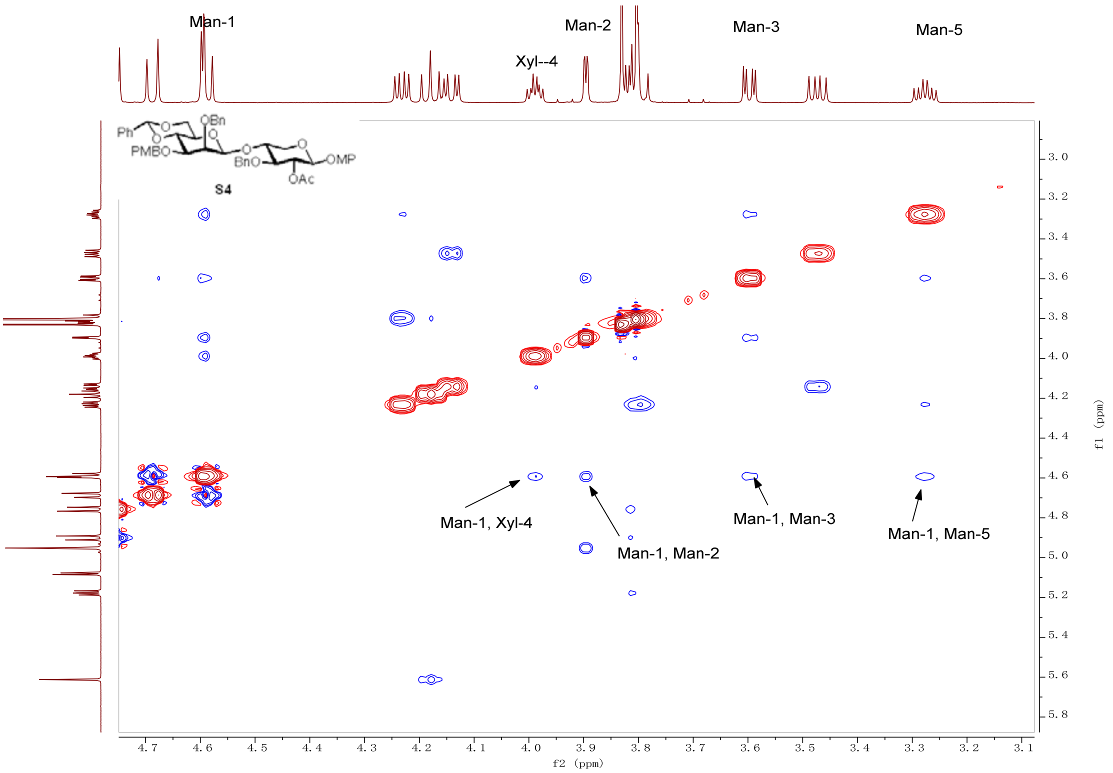

^{1}H-^{1}H NOESY spectrum of S4 (600 MHz, $CDCl_3$, 25 °C)

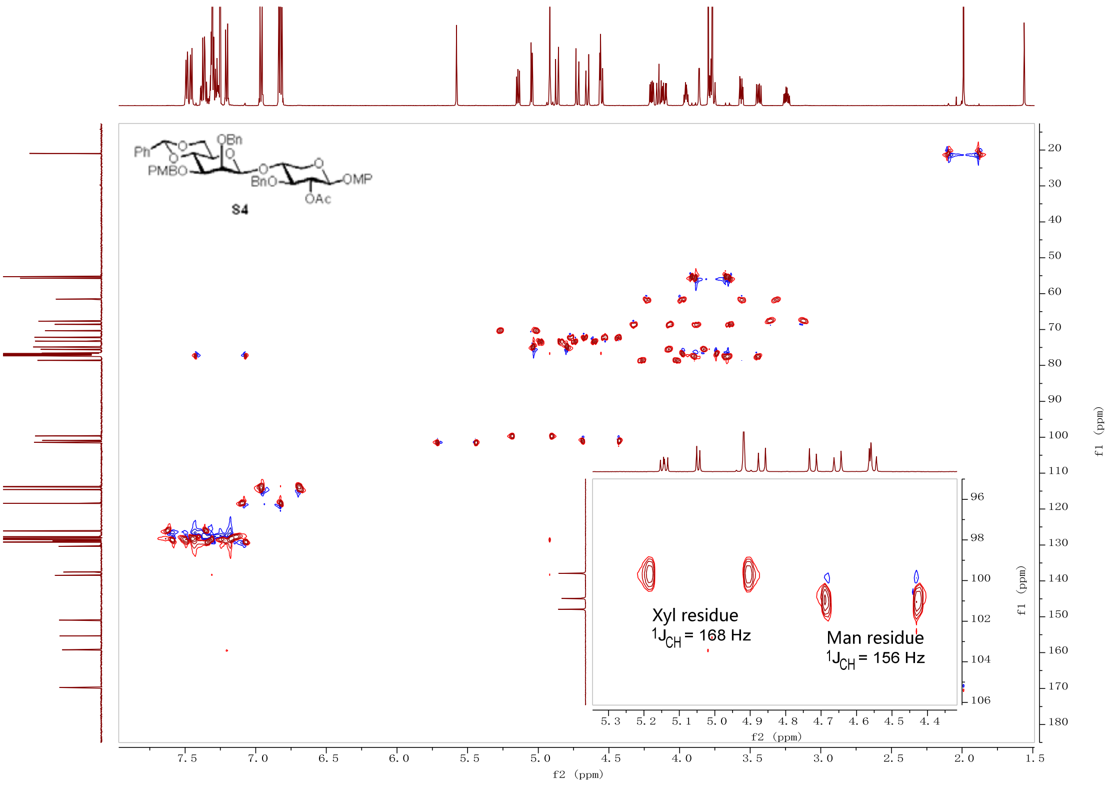

^{1}H-^{13}C coupled-HSQC spectrum of S4 (600 MHz, $CDCl_3$, 25 °C)

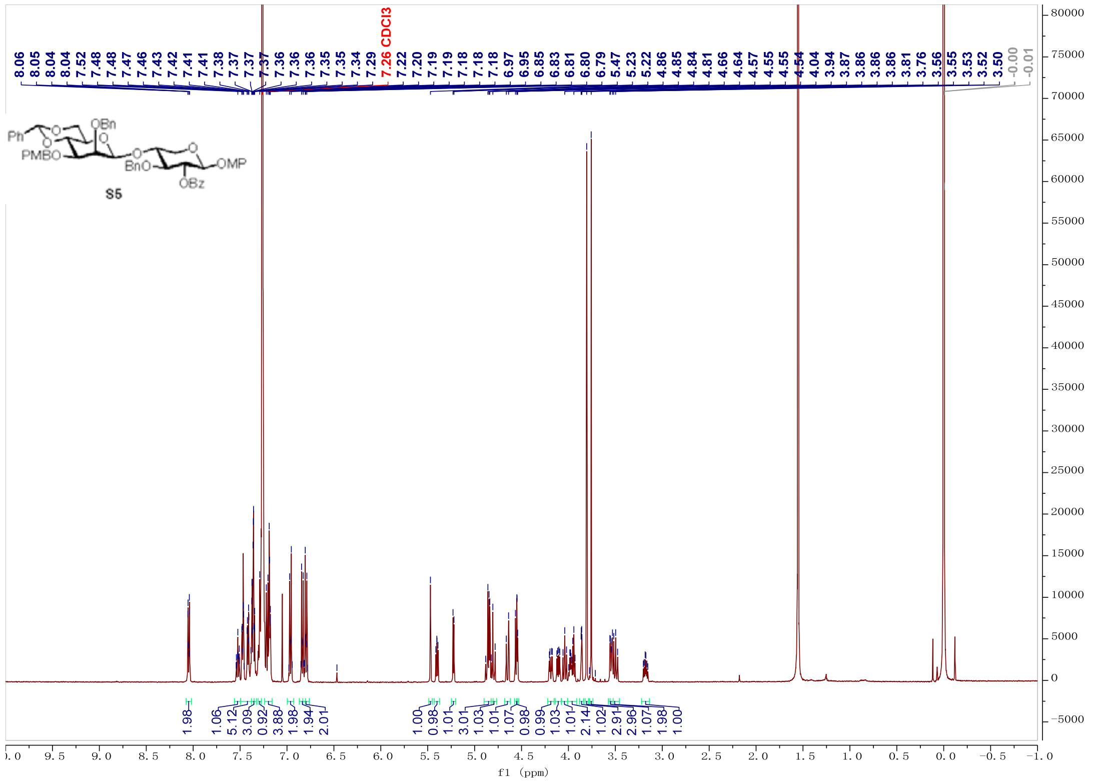

¹H NMR spectrum of S5 (500 MHz, $CDCl_3$, 25 °C)

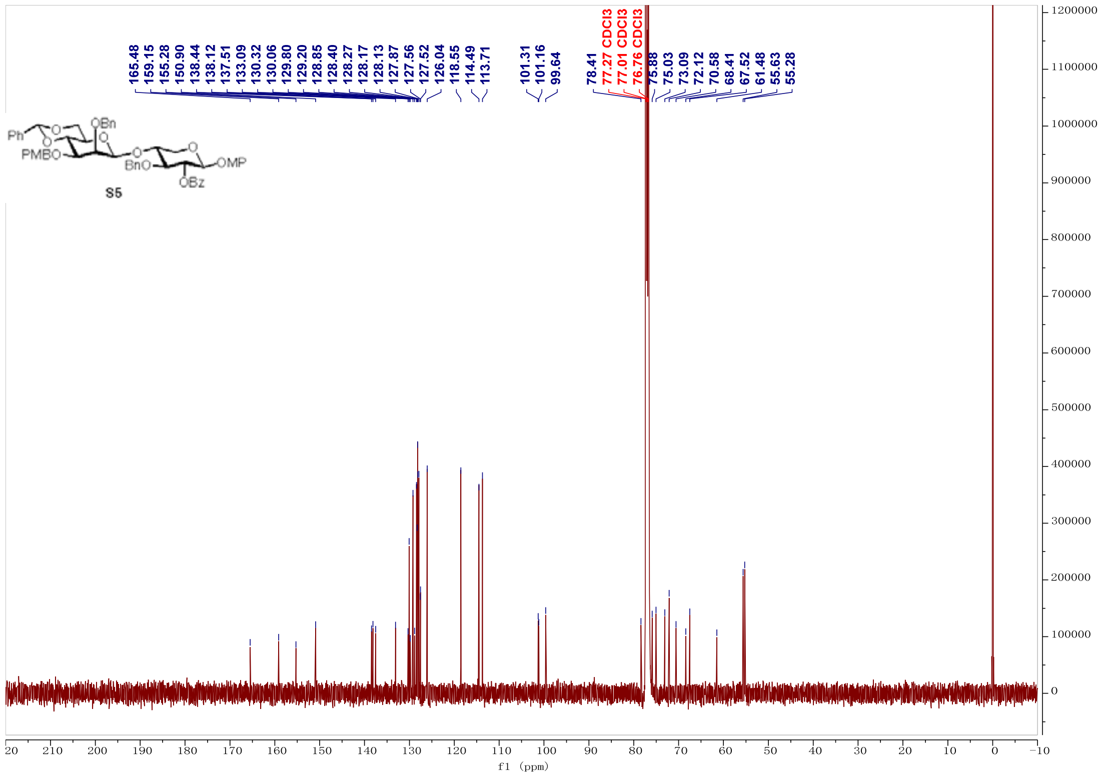

¹³C NMR spectrum of S5 (126 MHz, $CDCl_3$, 25 °C)

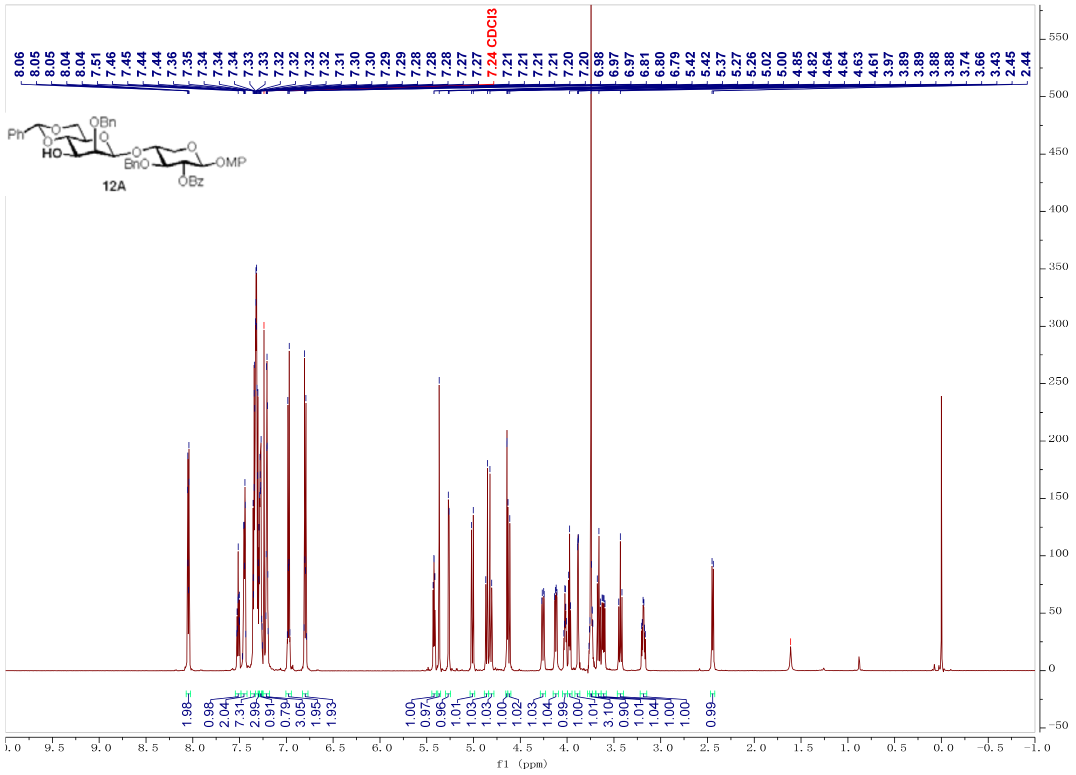

^{1}H NMR spectrum of 12A (600 MHz, $CDCl_3$, 25 °C)

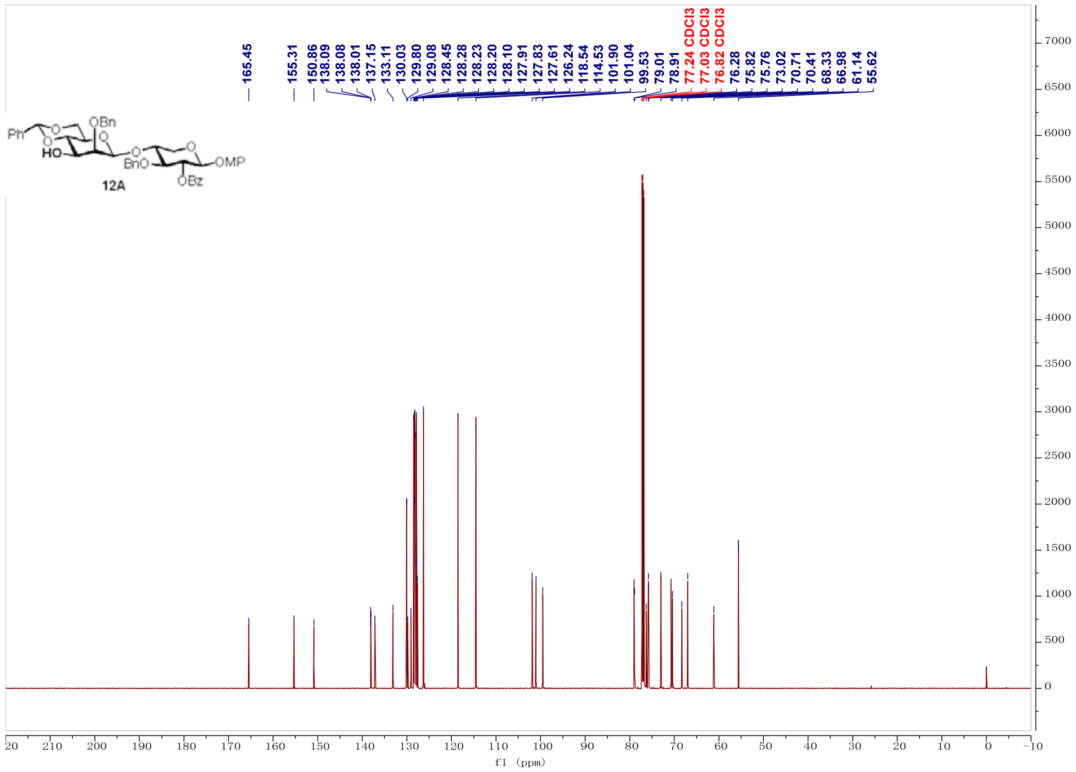

^{13}C NMR spectrum of 12A (151 MHz, $CDCl_3$, 25 °C)

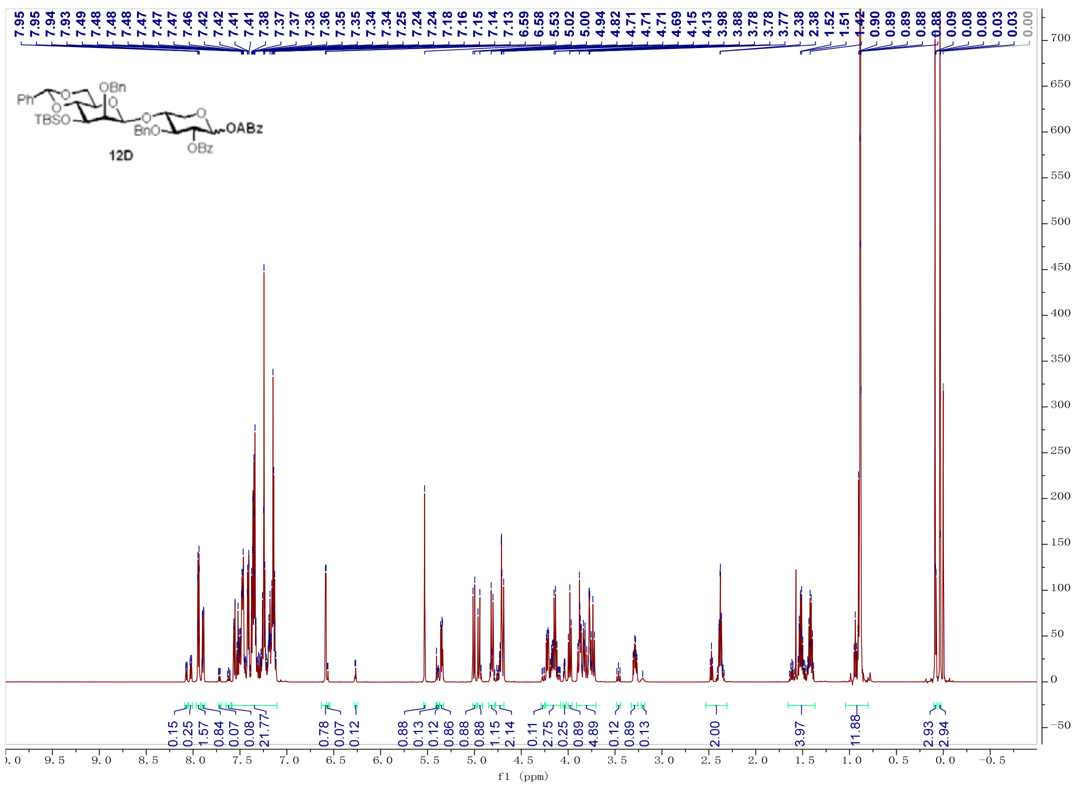

^{1}H NMR spectrum of 12D (600 MHz, $CDCl_3$, 25 °C)

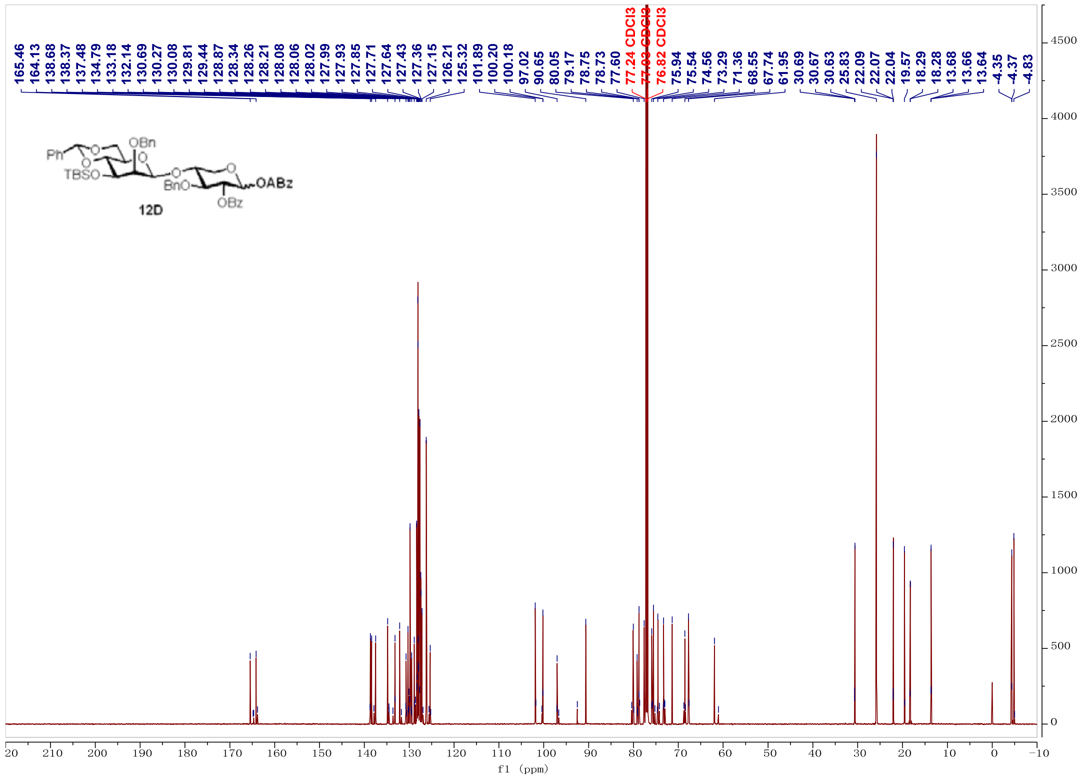

^{13}C NMR spectrum of 12D (151 MHz, $CDCl_3$, 25 °C)

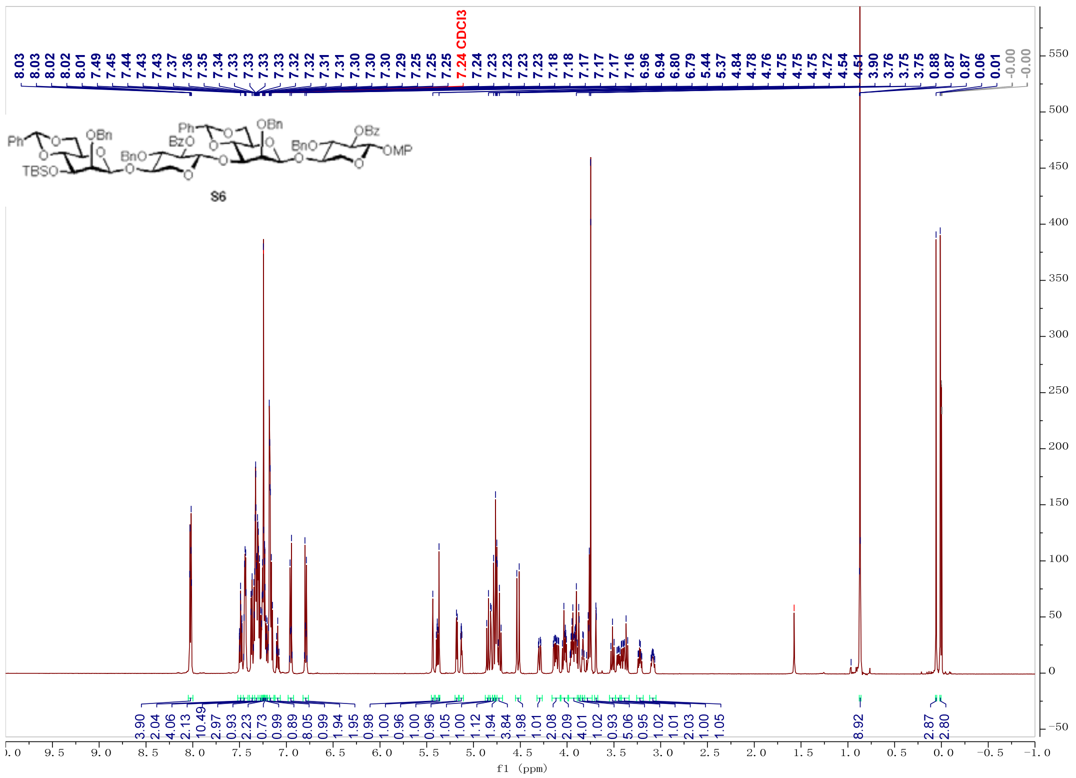

^{1}H NMR spectrum of S6 (600 MHz, $CDCl_3$, 25 °C)

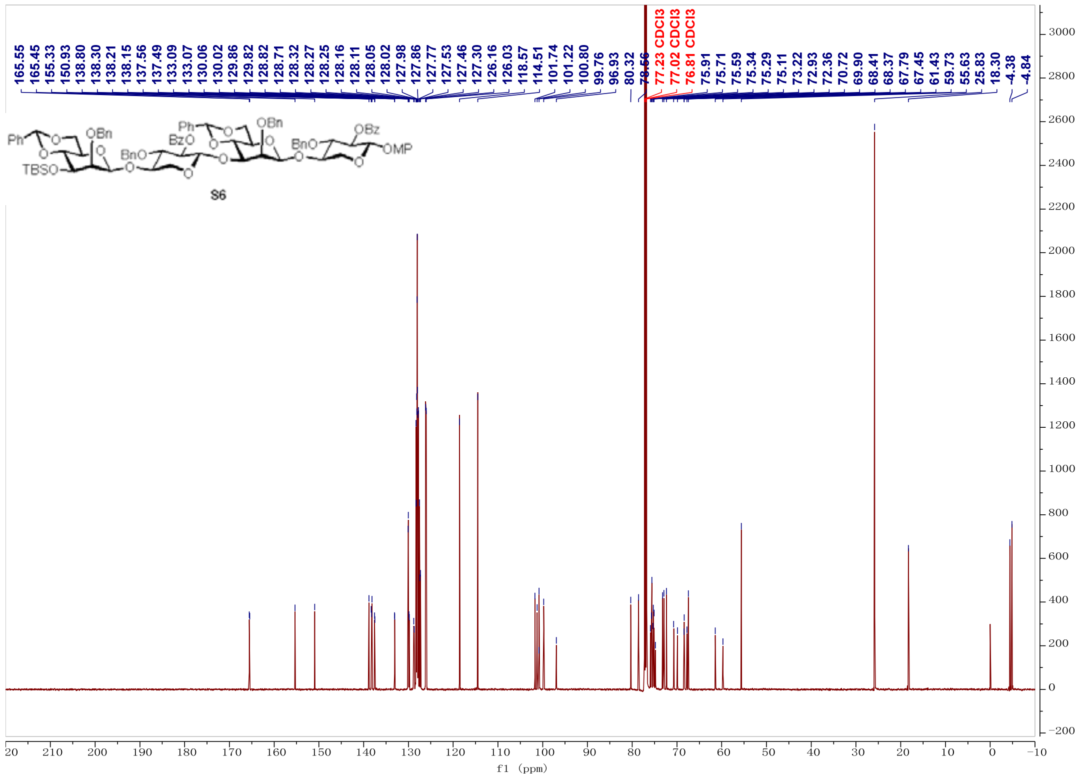

^{13}C NMR spectrum of S6 (151 MHz, $CDCl_3$, 25 °C)

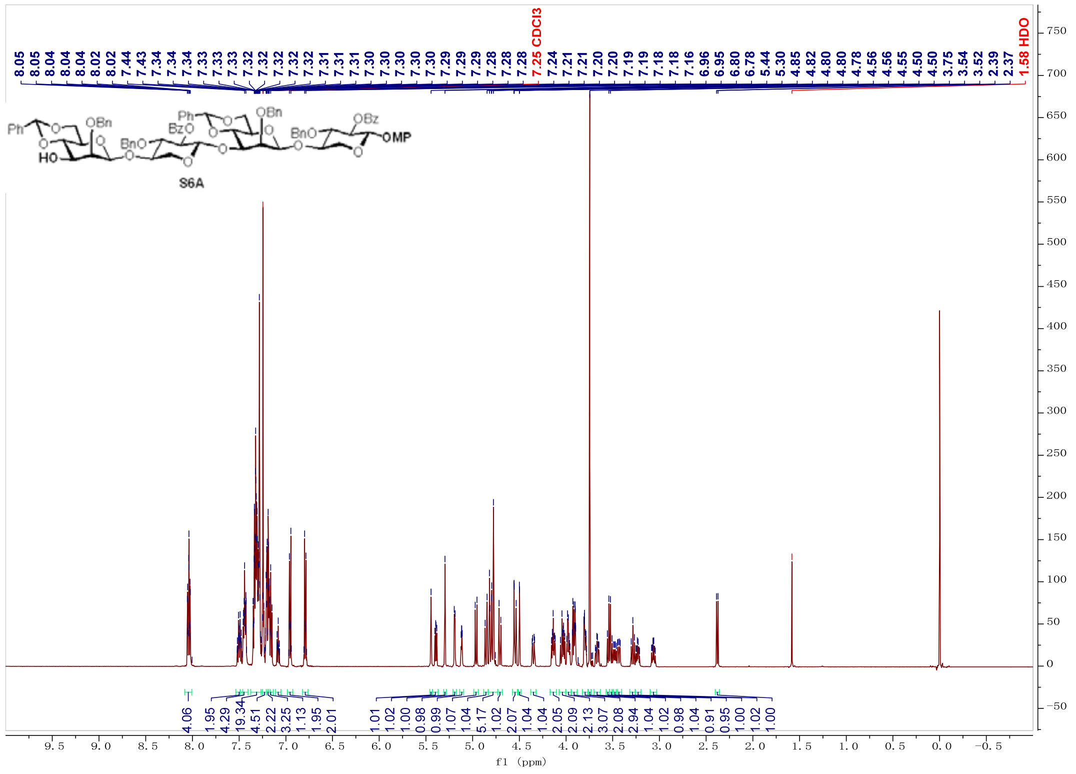

^{1}**H NMR spectrum of S6A** (600 MHz, $CDCl_3$, 25 °C)

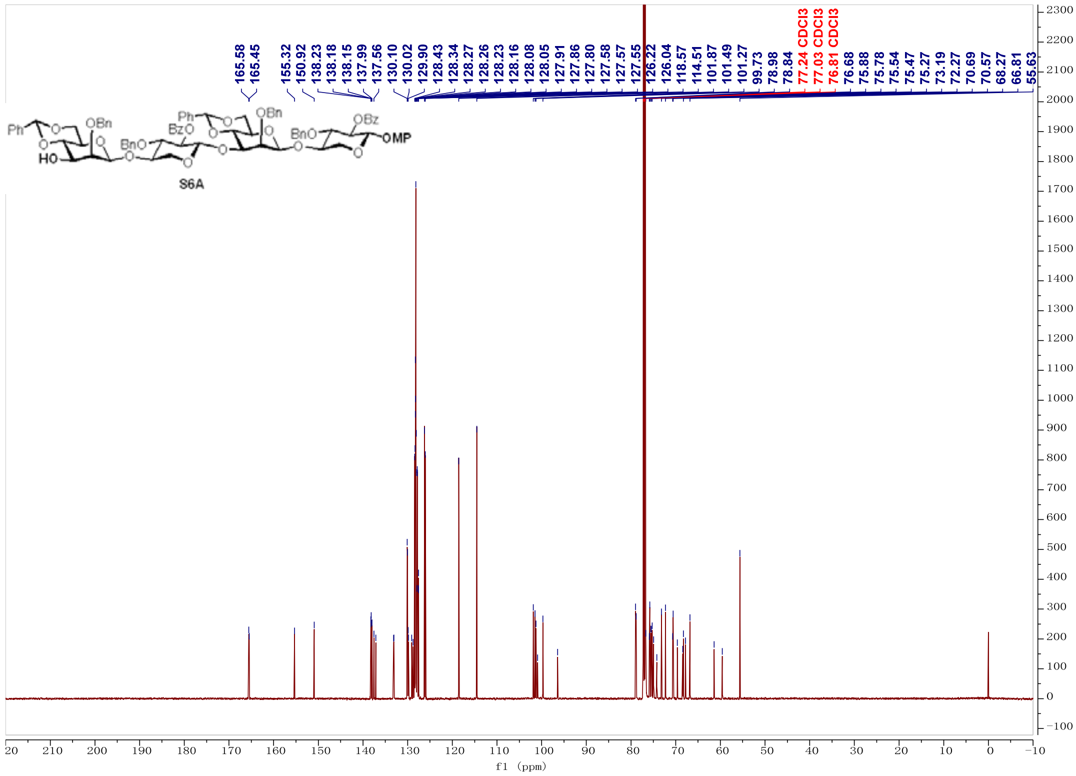

^{13}**C NMR spectrum of S6A** (151 MHz, $CDCl_3$, 25 °C)

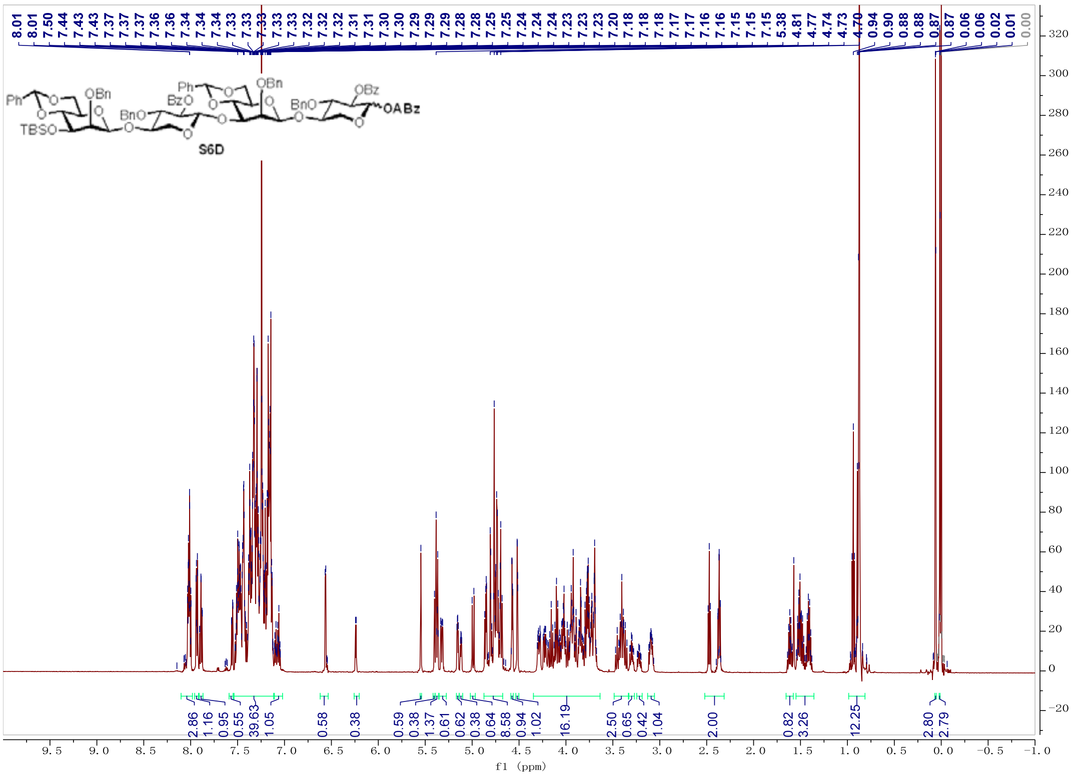

¹H NMR spectrum of S6D (600 MHz, $CDCl_3$, 25 °C)

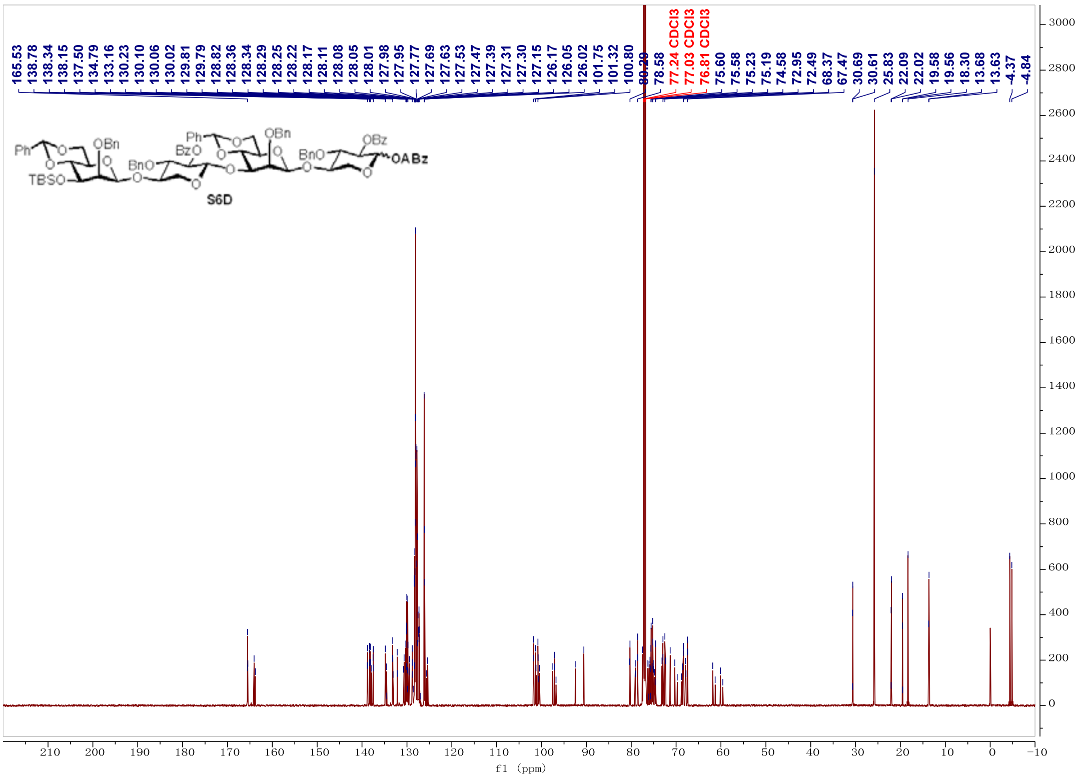

¹³C NMR spectrum of S6D (151 MHz, $CDCl_3$, 25 °C)

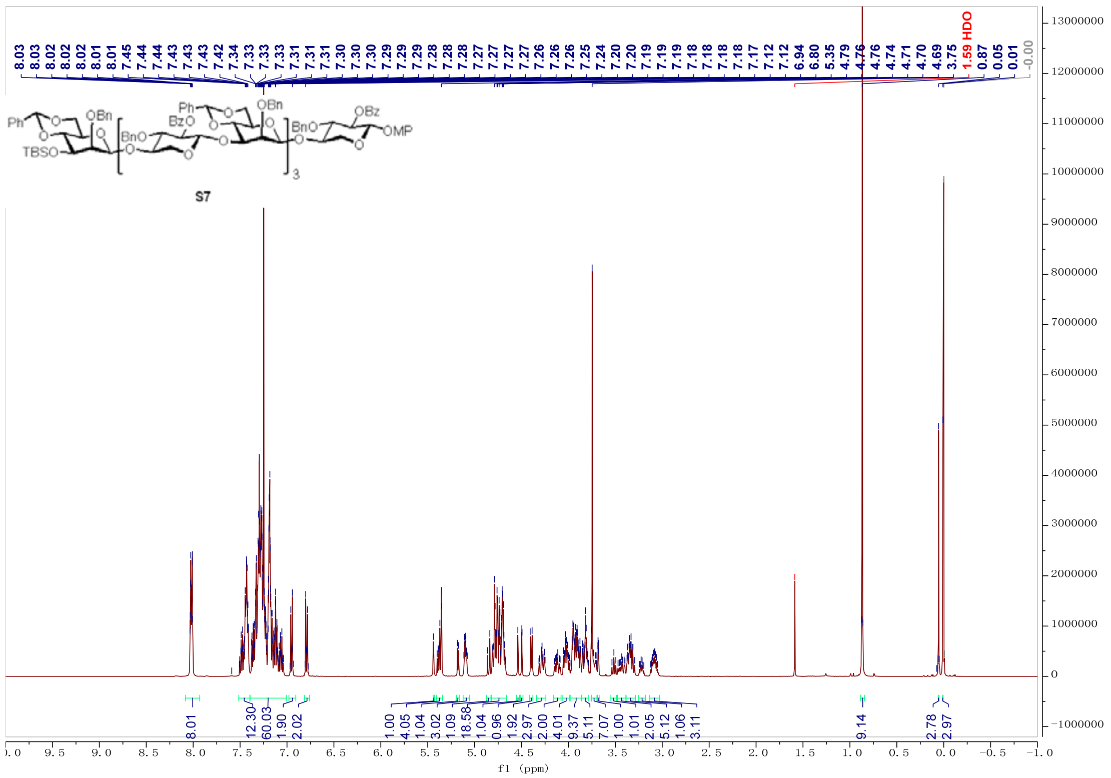

^{1}H NMR spectrum of S7 (500 MHz, $CDCl_3$, 25 °C)

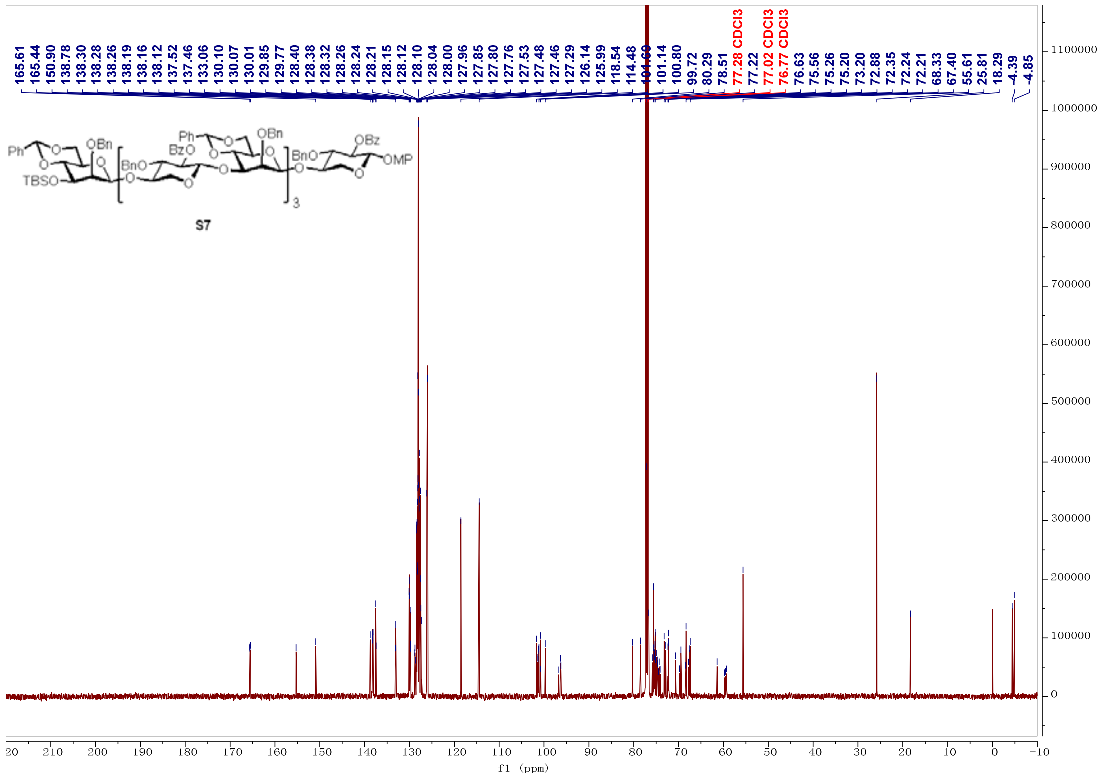

^{13}C NMR spectrum of S7 (126 MHz, $CDCl_3$, 25 °C)

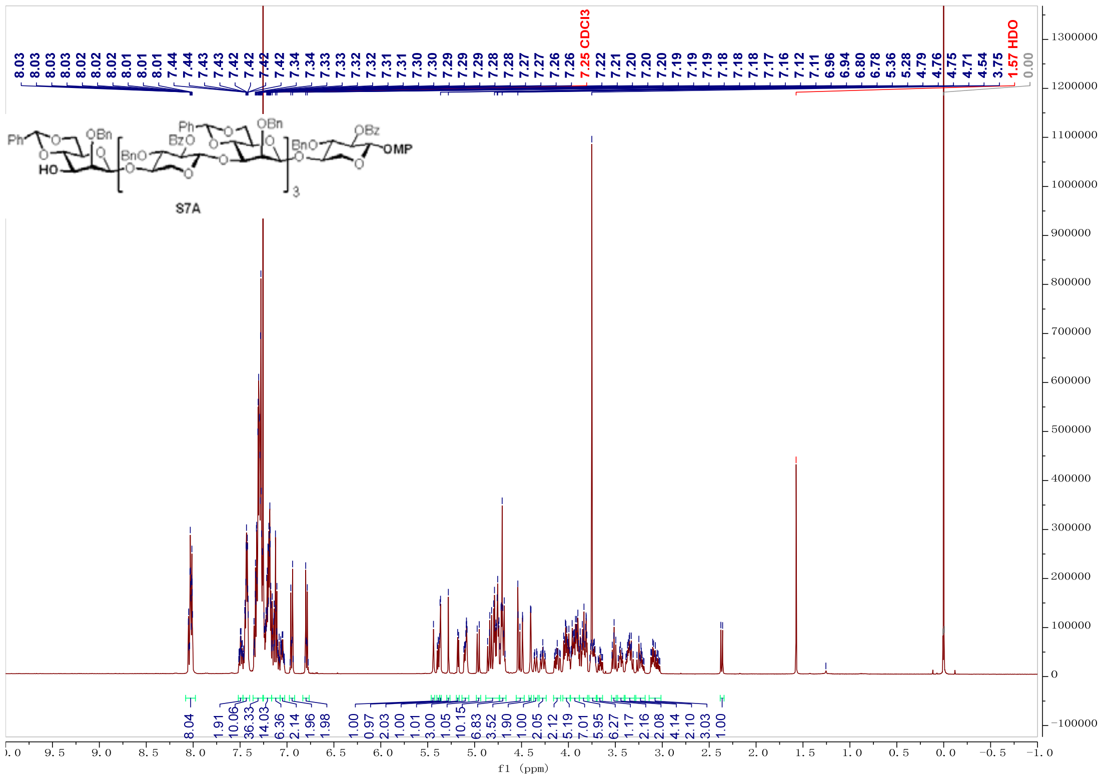

^{1}H NMR spectrum of S7A (500 MHz, $CDCl_3$, 25 °C)

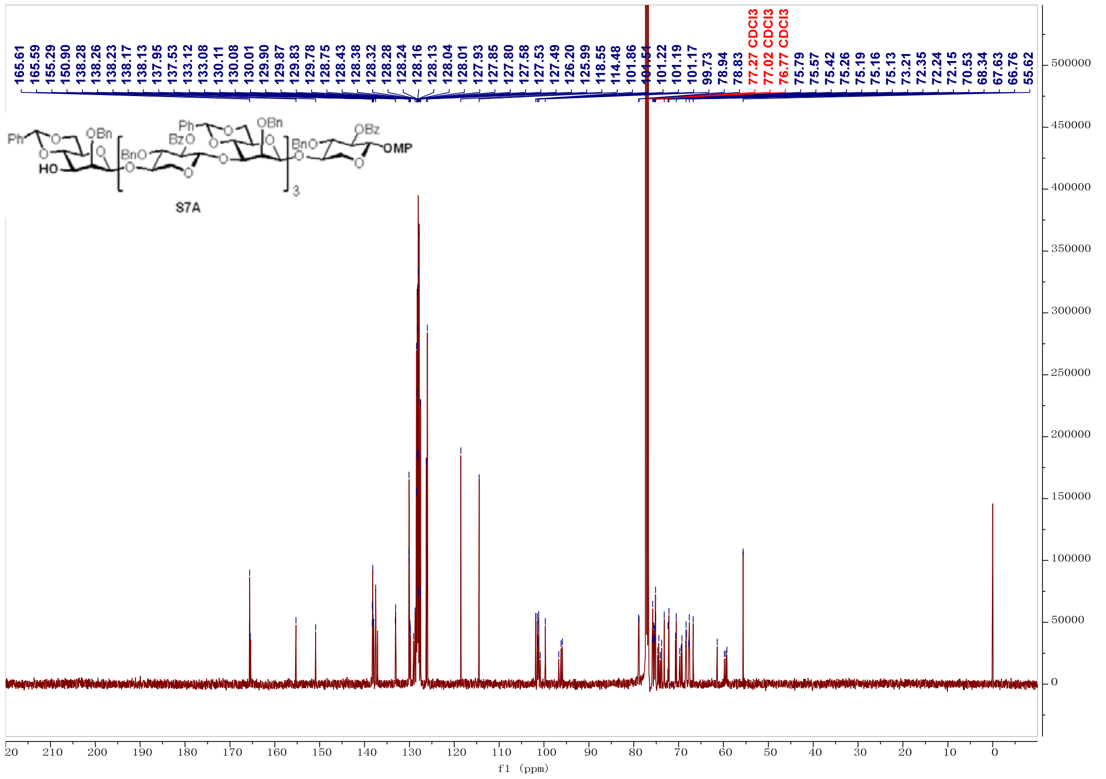

^{13}C NMR spectrum of S7A (126 MHz, $CDCl_3$, 25 °C)

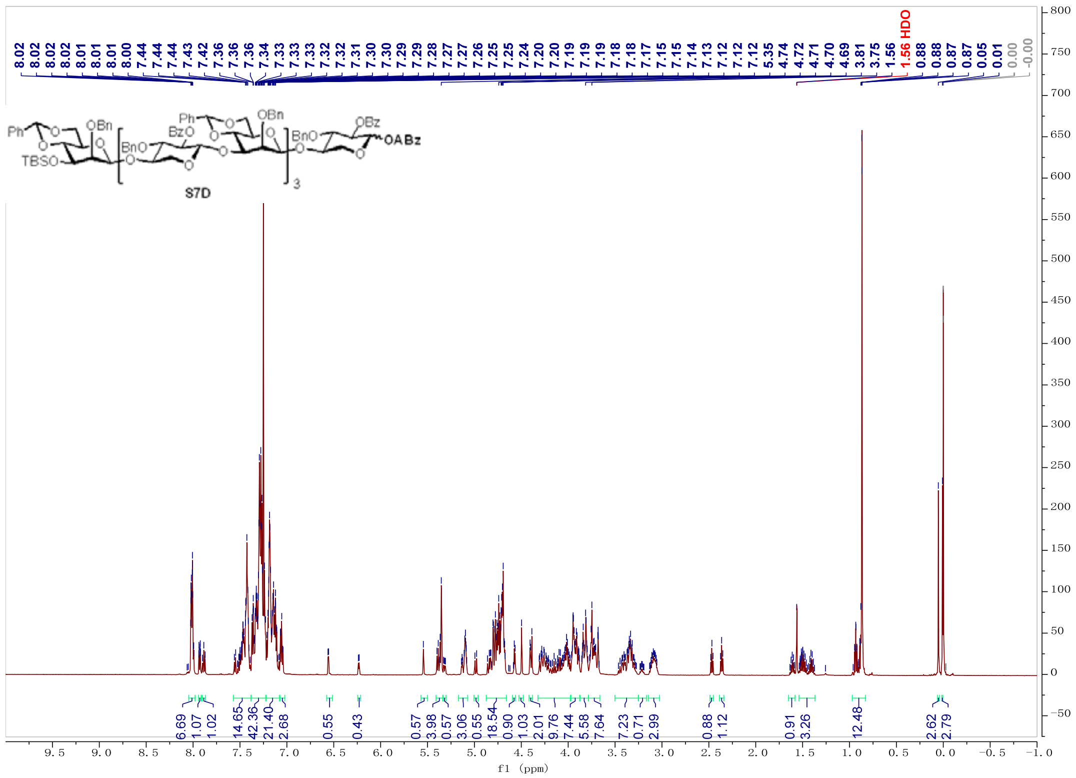

^{1}H NMR spectrum of S7D (600 MHz, $CDCl_3$, 25 °C)

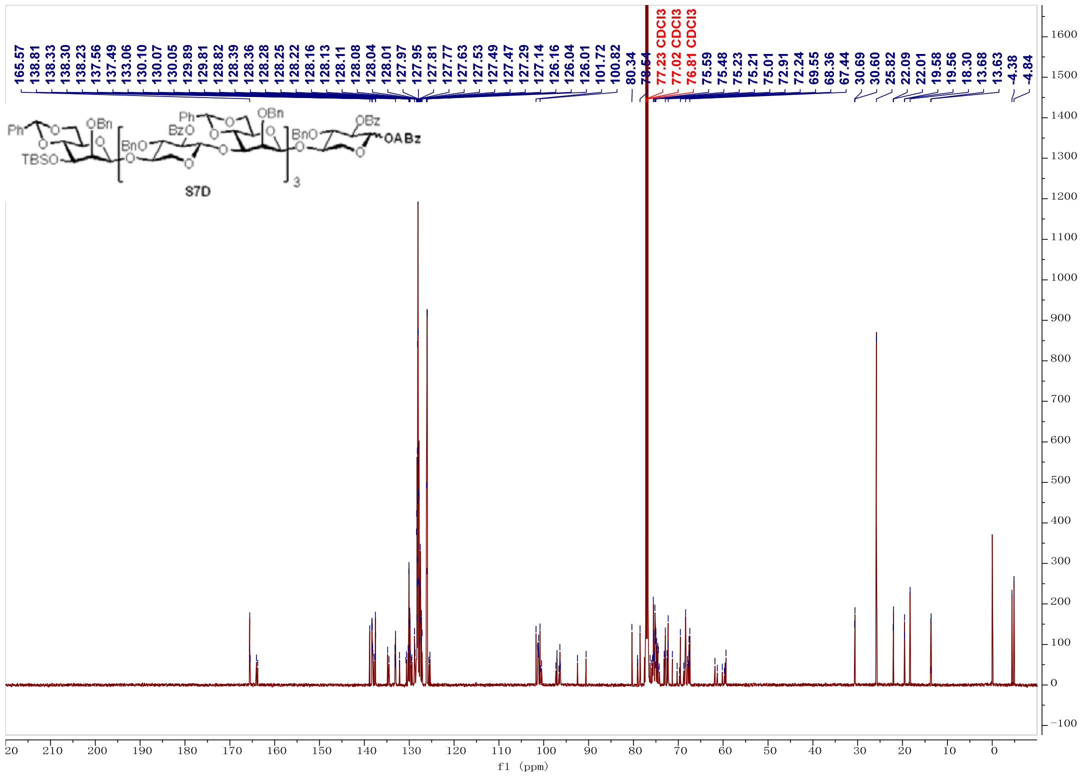

^{13}C NMR spectrum of S7D (151 MHz, $CDCl_3$, 25 °C)

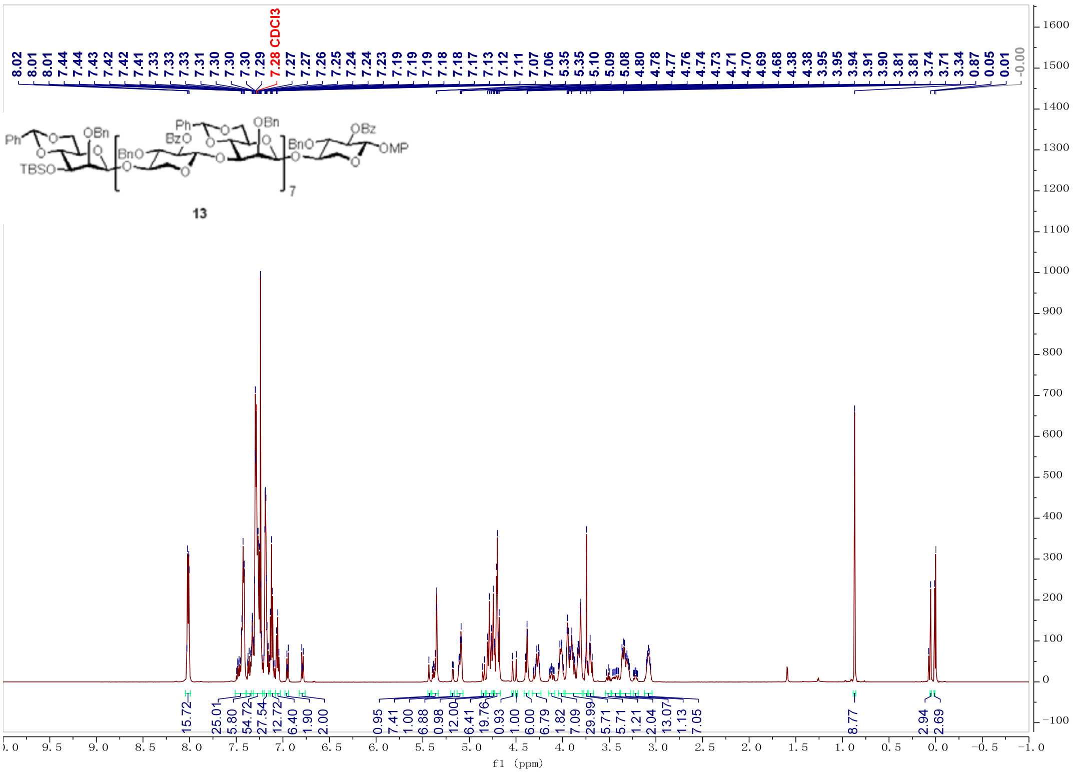

^{1}H NMR spectrum of 13 (600 MHz, $CDCl_3$, 25 °C)

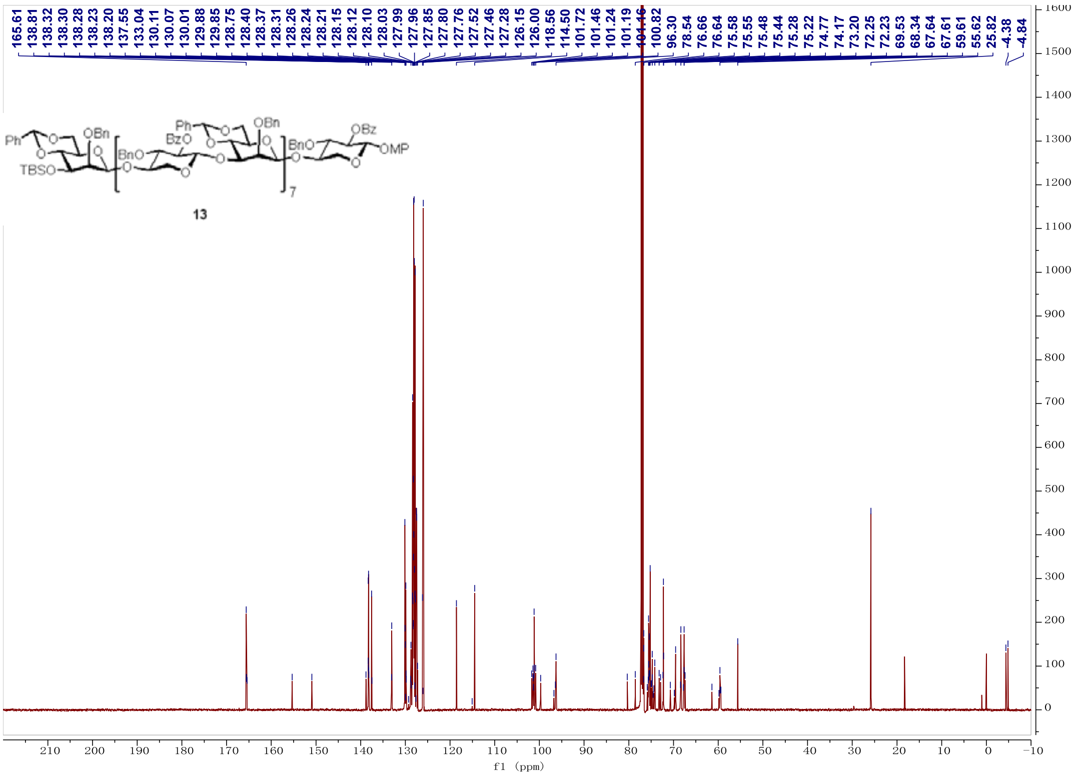

^{13}C NMR spectrum of 13 (151 MHz, $CDCl_3$, 25 °C)

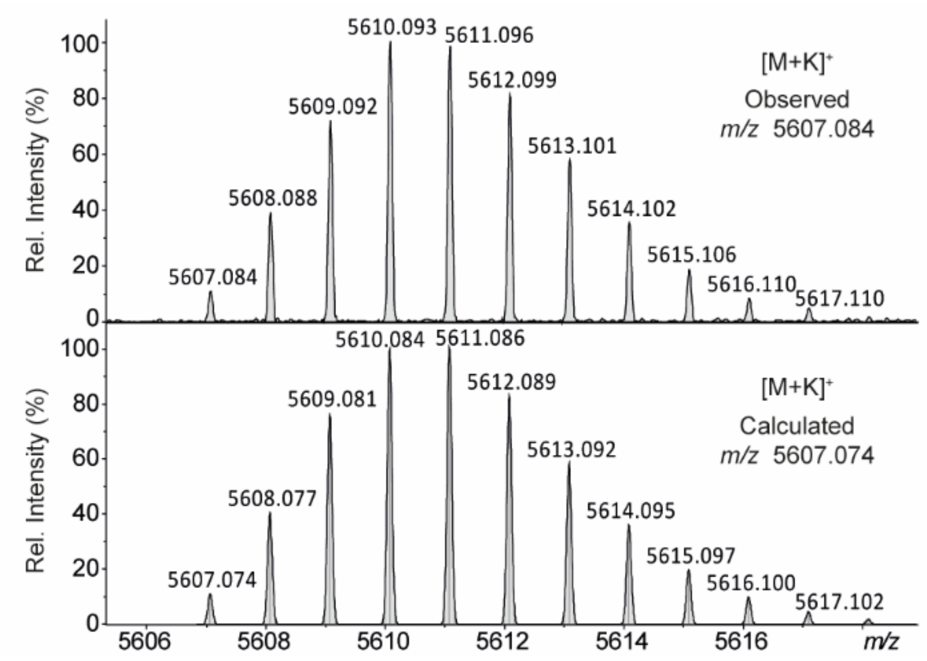

MALDI-FT-ICR MS spectrum of compound 13

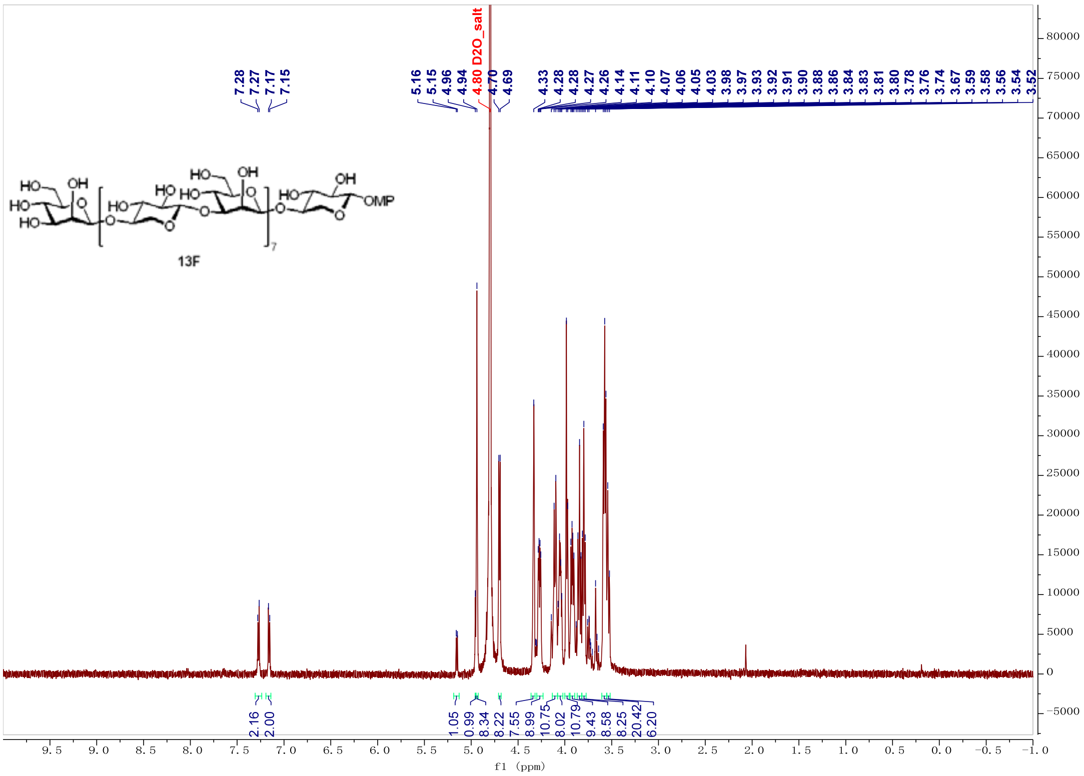

^{1}H NMR spectrum of compound 13F (600 MHz, 20 mM Na_3PO_4 buffered D_2O, 40 °C)

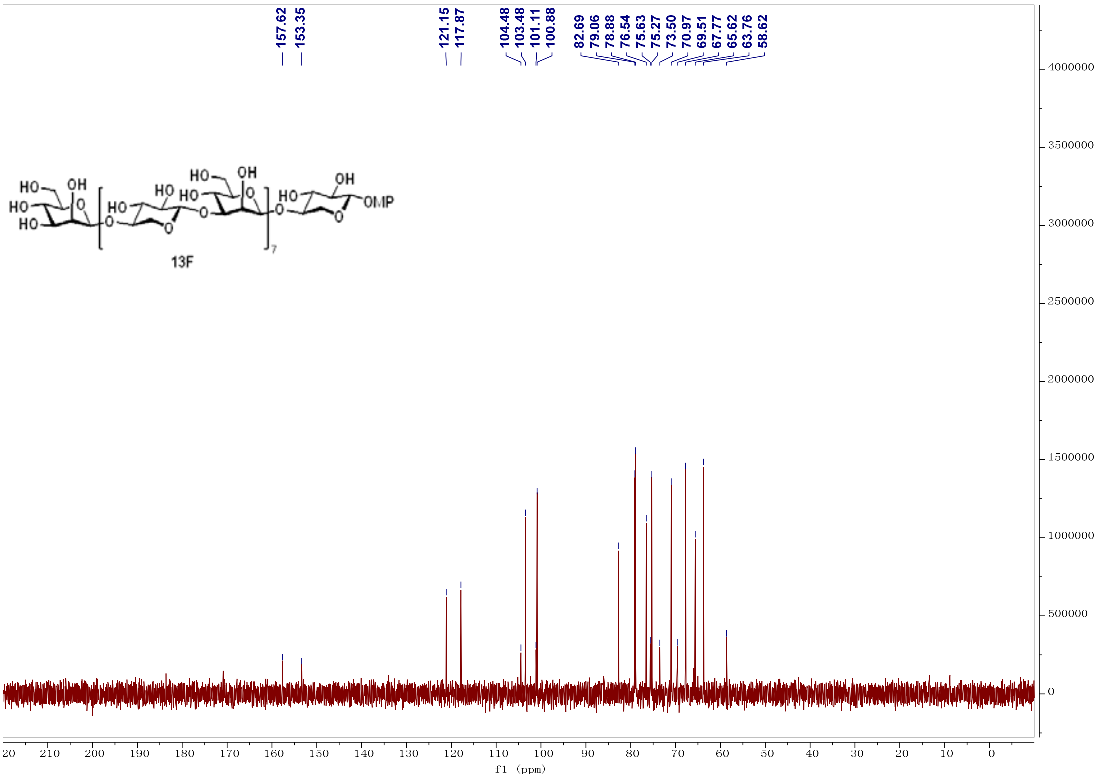

^{13}C NMR spectrum of compound 13F (151 MHz, 20 mM Na_3PO_4 buffered D_2O, 40 °C)

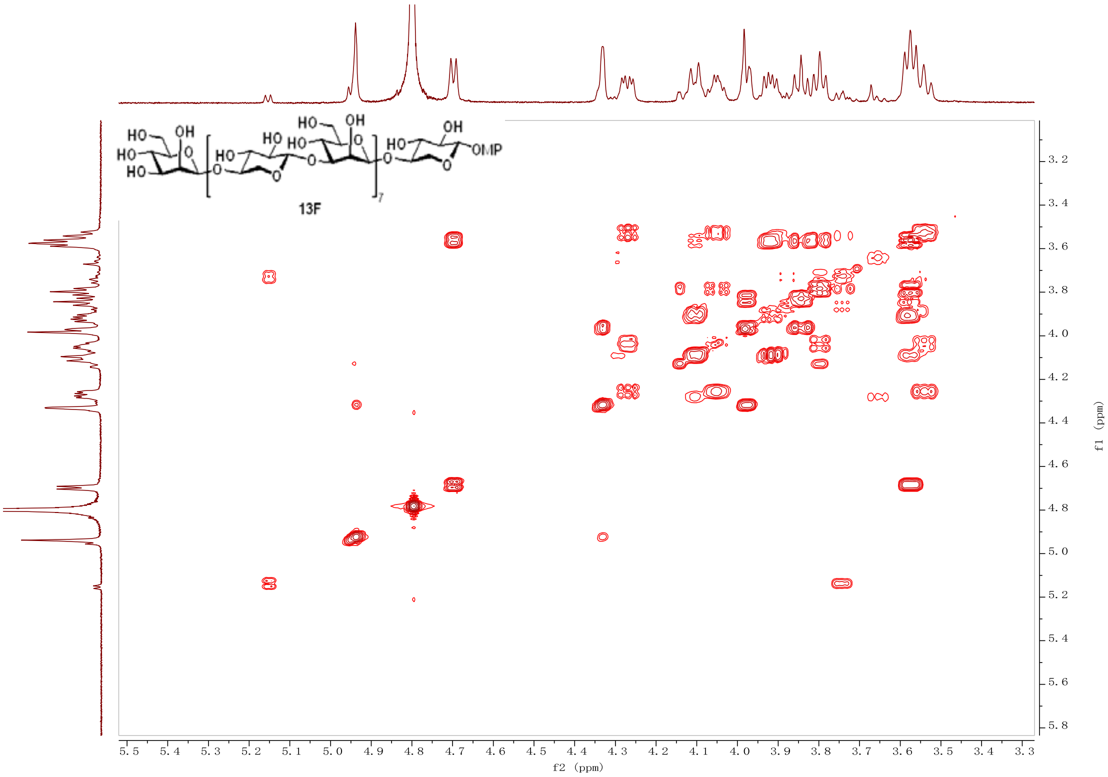

^{1}H-^{1}H gCOSY spectrum of compound 13F (600 MHz, 20 mM Na_3PO_4 buffered D_2O, 40 °C)

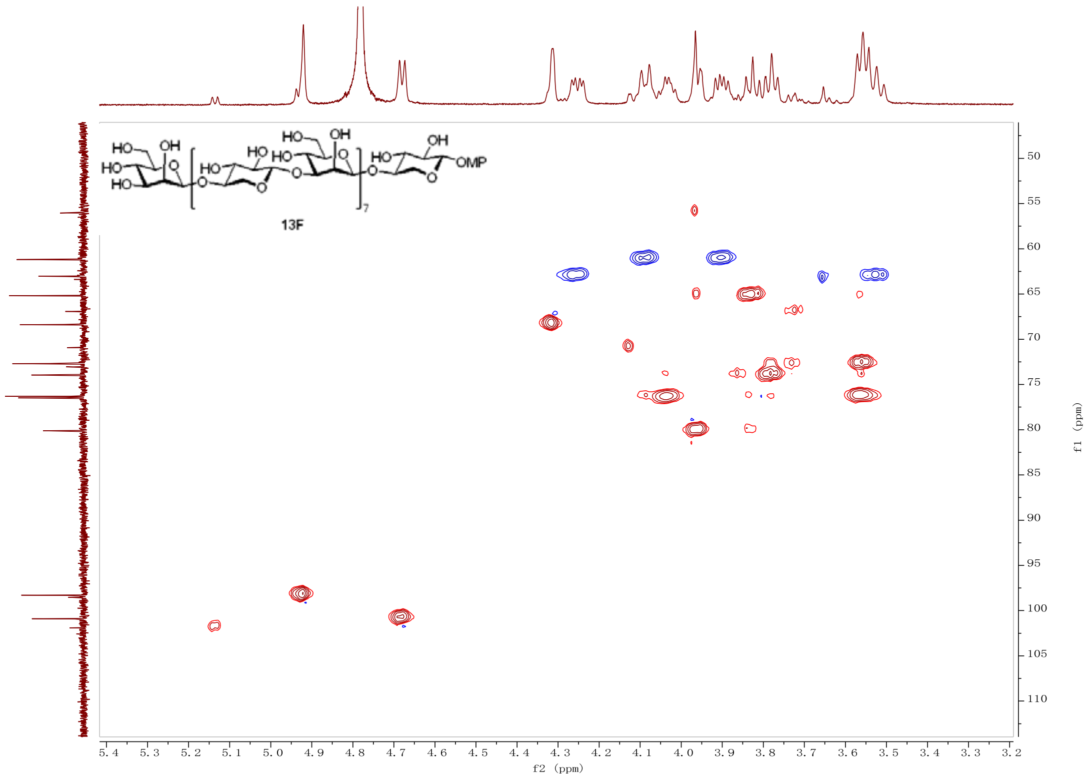

^{1}H-^{13}C HSQC spectrum of compound 13F (600 MHz, 20 mM Na_3PO_4 buffered D_2O, 40 °C)

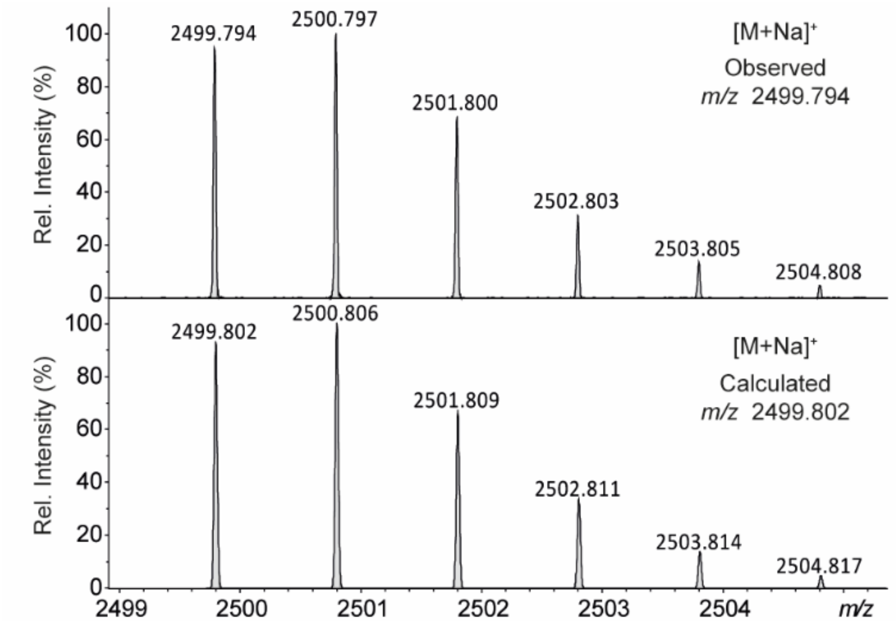

MALDI-FT-ICR MS spectrum of compound 13F

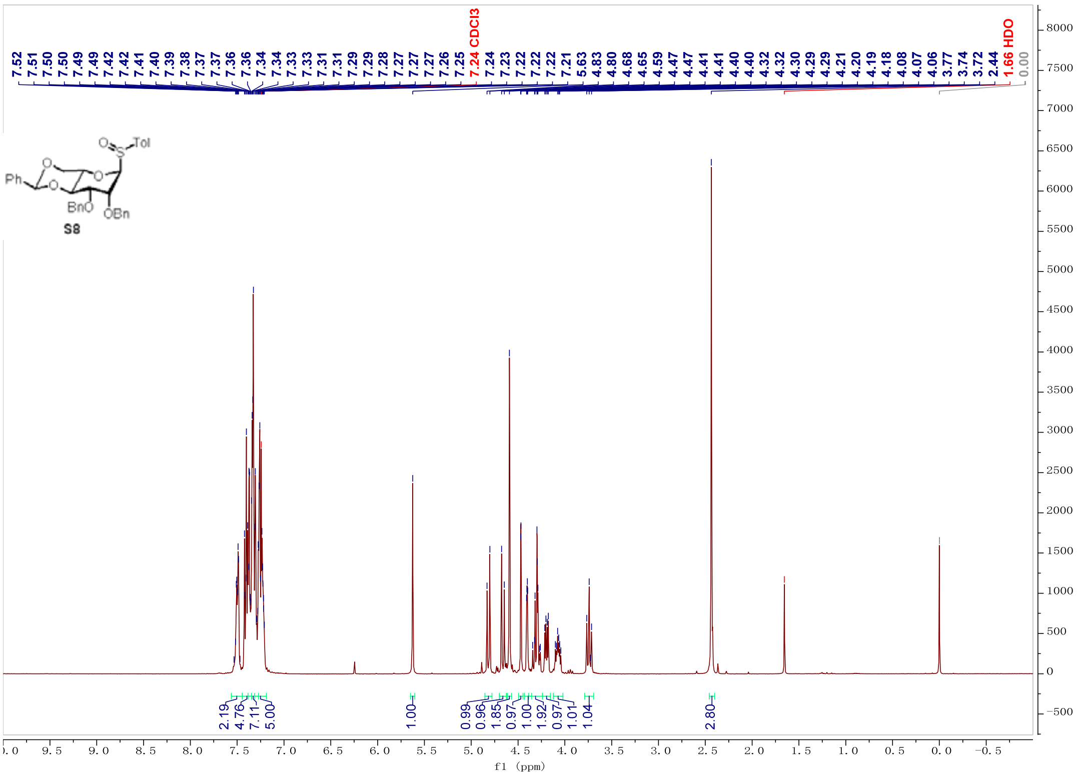

^{1}H NMR spectrum of S8 (400 MHz, $CDCl_3$, 25 °C)

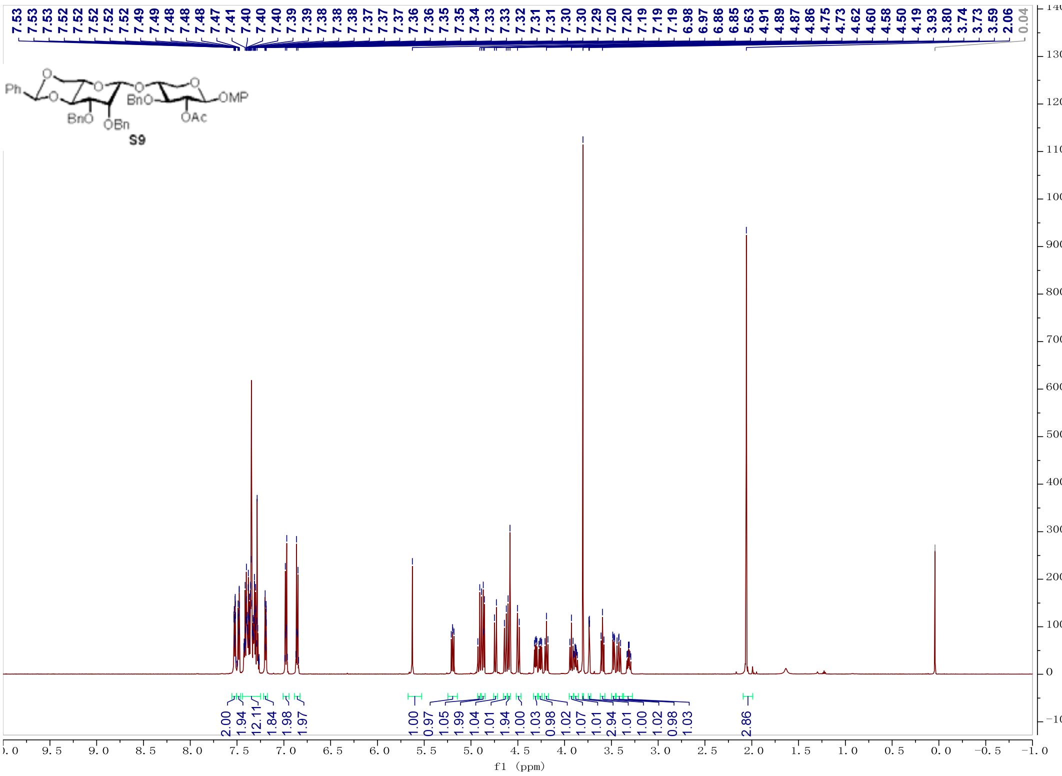

^{1}H NMR spectrum of S9 (600 MHz, $CDCl_3$, 25 °C)

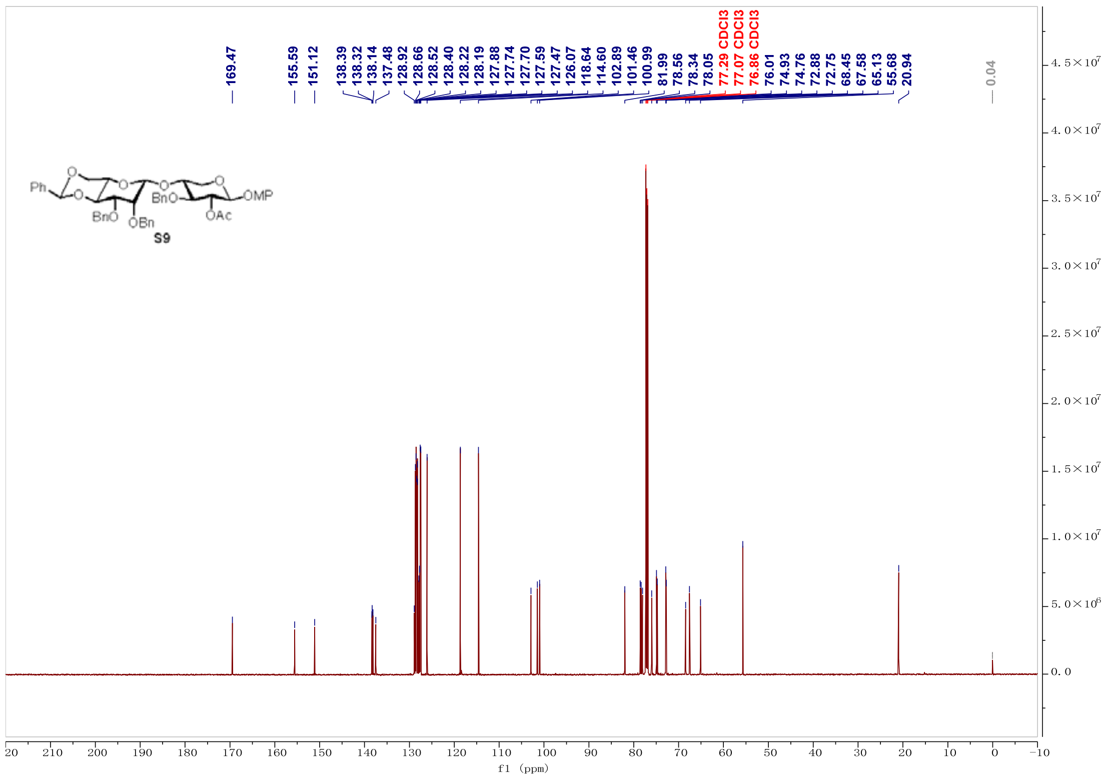

^{13}C NMR spectrum of S9 (151 MHz, $CDCl_3$, 25 °C)

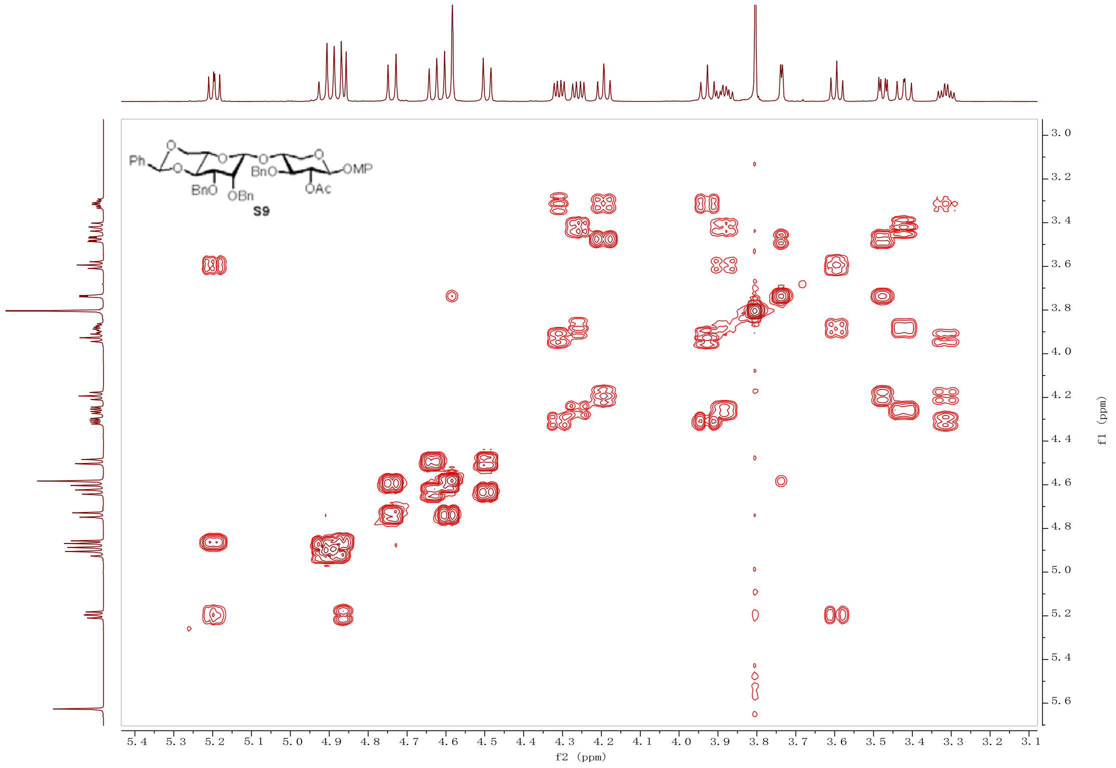

^{1}H-^{1}H gCOSY spectrum of S9 (600 MHz, $CDCl_3$, 25 °C)

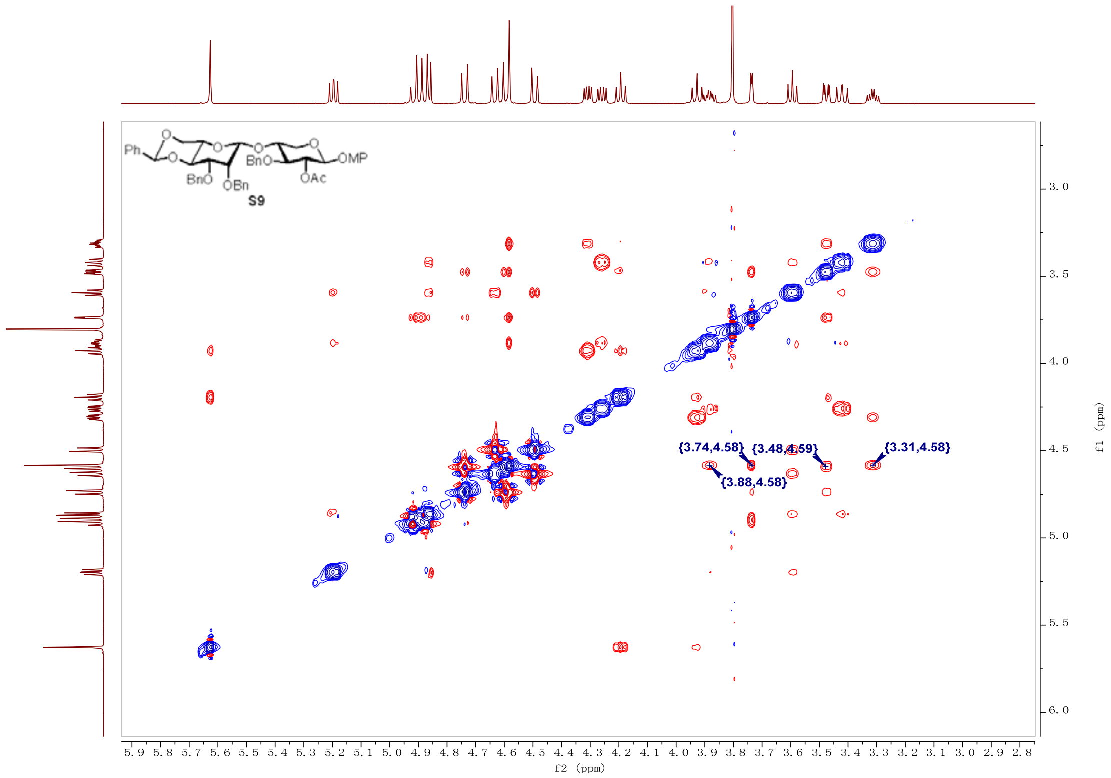

^{1}H-^{1}H NOESY spectrum of S9 (600 MHz, $CDCl_3$, 25 °C)

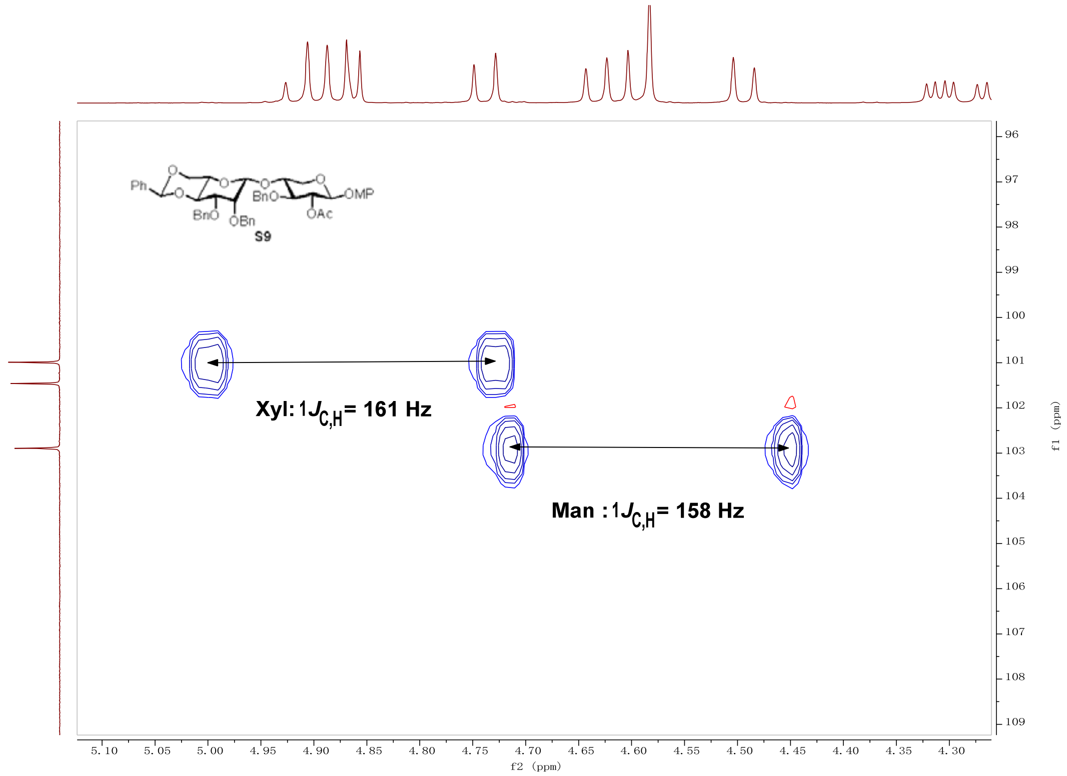

^{1}H-^{13}C coupled-HSQC spectrum of S9 (600 MHz, $CDCl_3$, 25 °C)

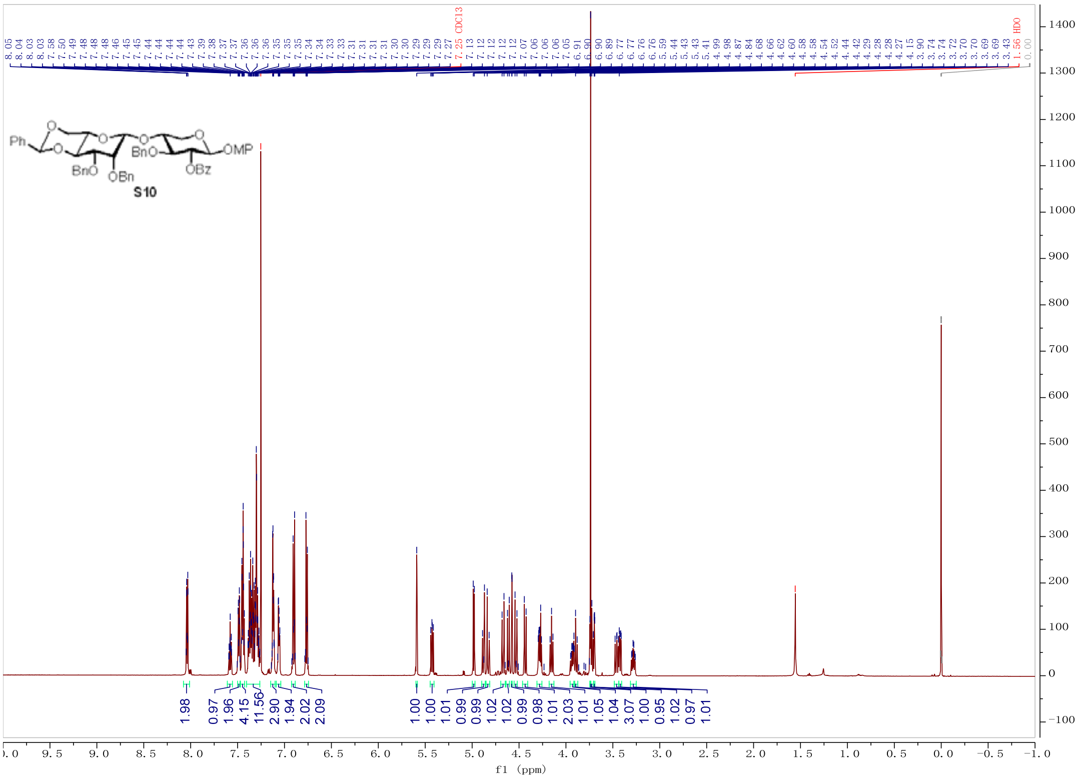

^{1}H NMR spectrum of S10 (600 MHz, $CDCl_3$, 25 °C)

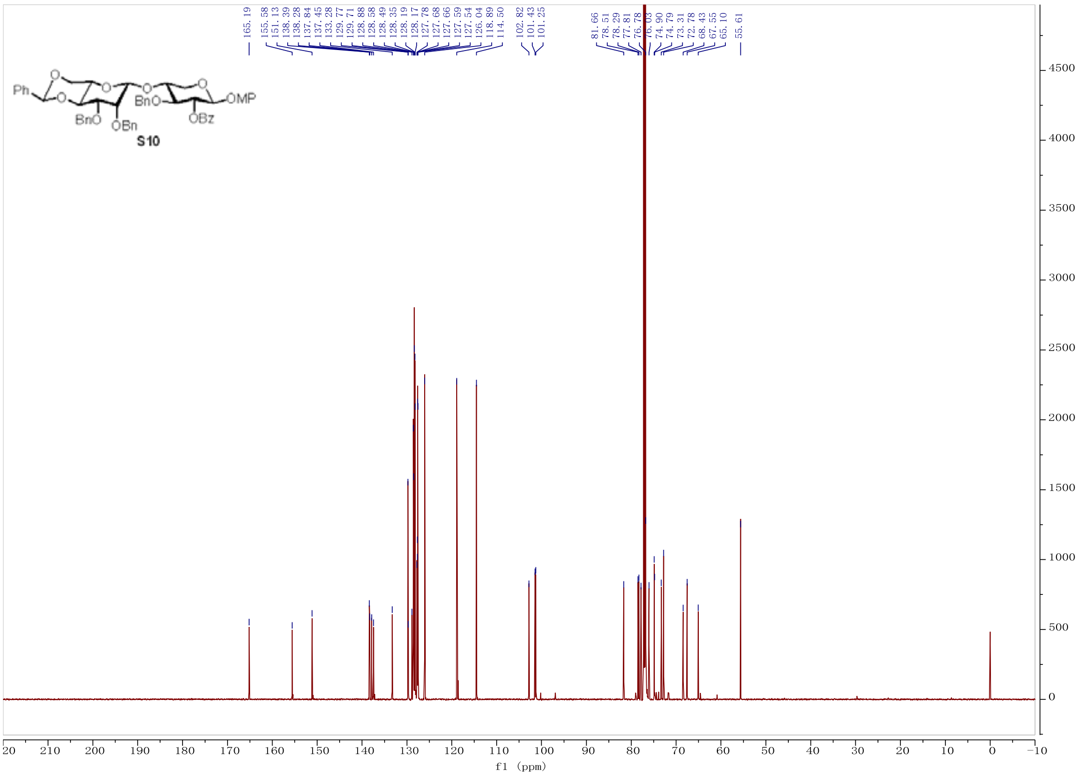

^{13}C NMR spectrum of S10 (151 MHz, $CDCl_3$, 25 °C)

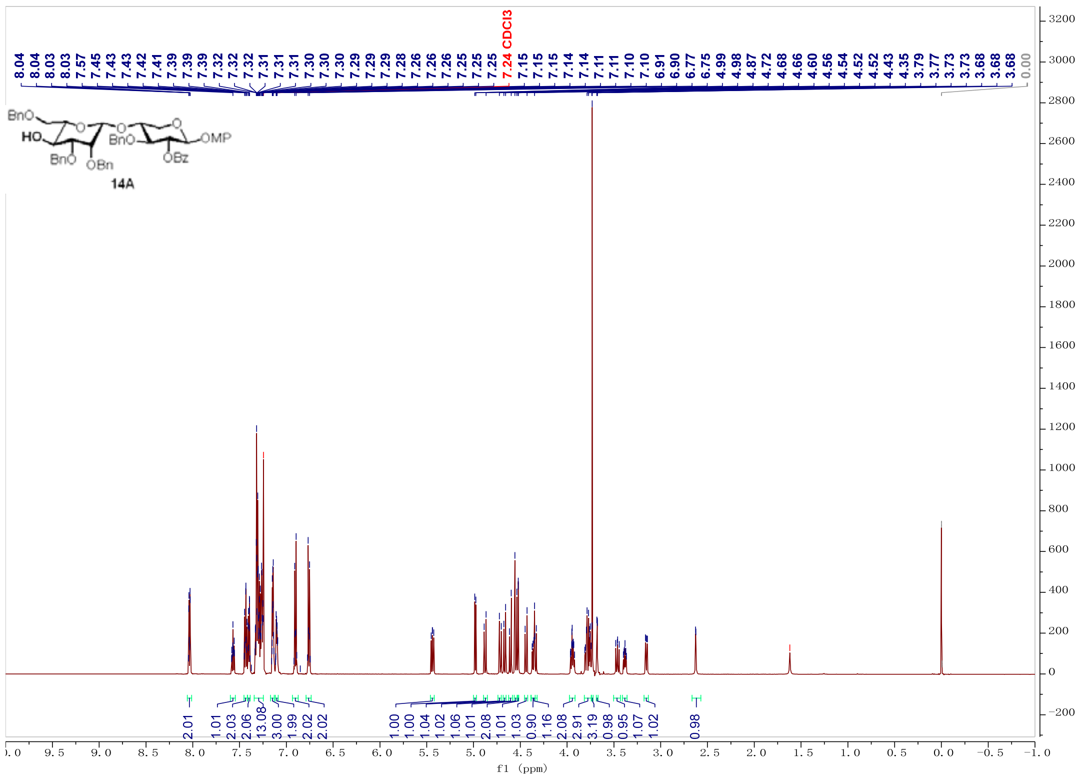

^{1}H NMR spectrum of 14A (600 MHz, $CDCl_3$, 25 °C)

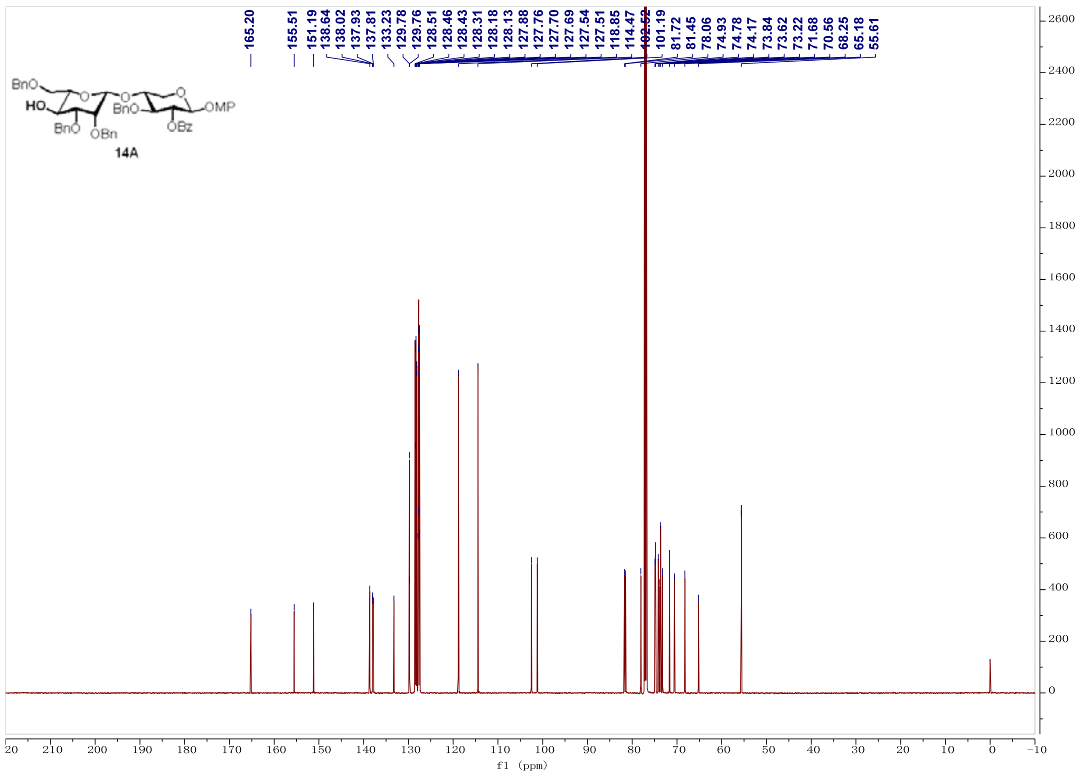

^{13}C NMR spectrum of 14A (151 MHz, $CDCl_3$, 25 °C)

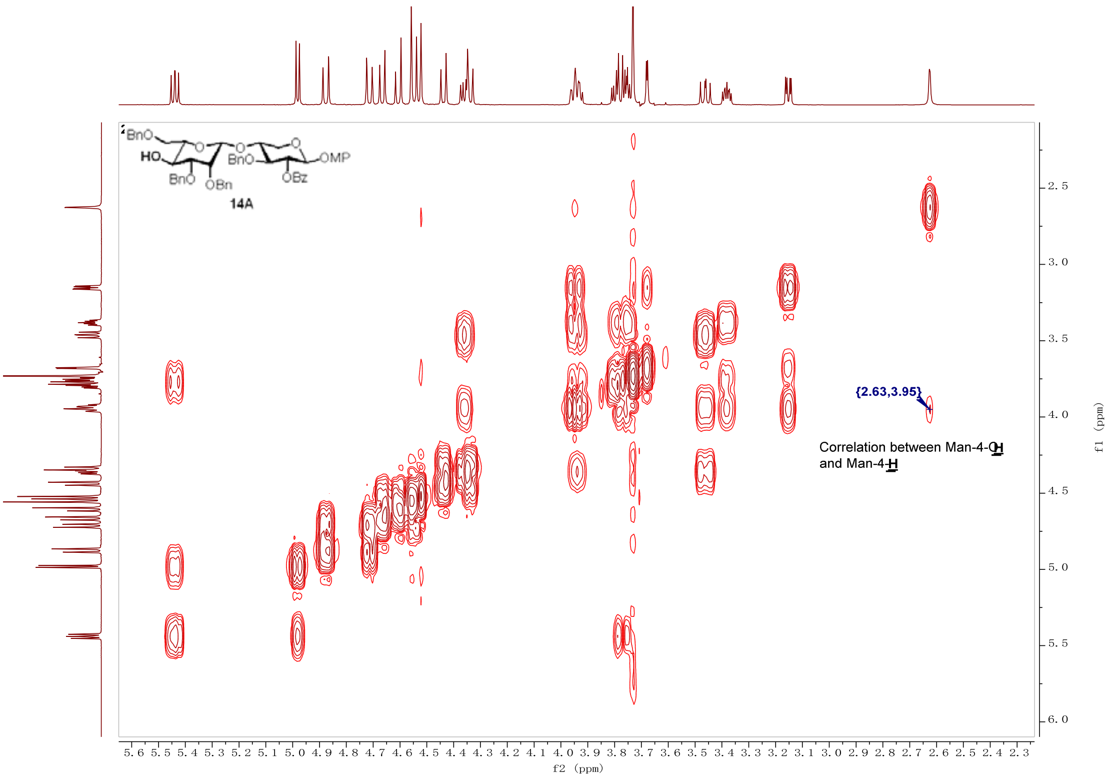

^{1}H-^{1}H gCOSY spectrum of 14A (600 MHz, $CDCl_3$, 25 °C)

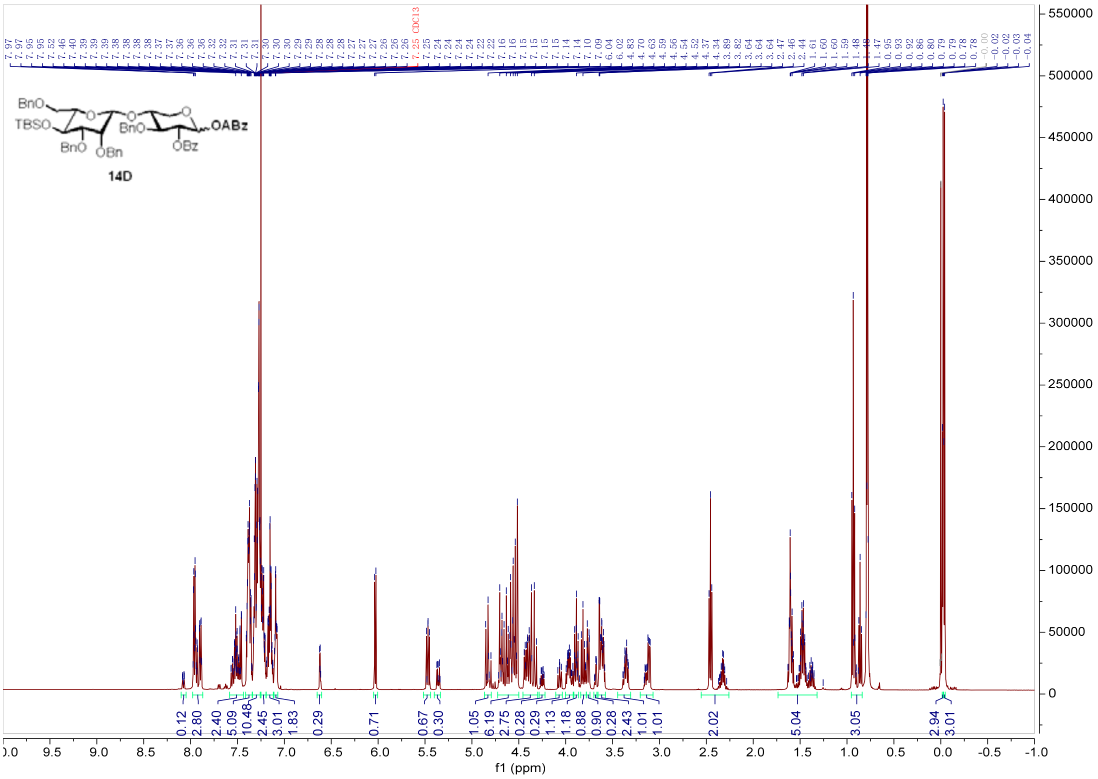

^{1}H NMR spectrum of 14D (500 MHz, $CDCl_3$, 25 °C)

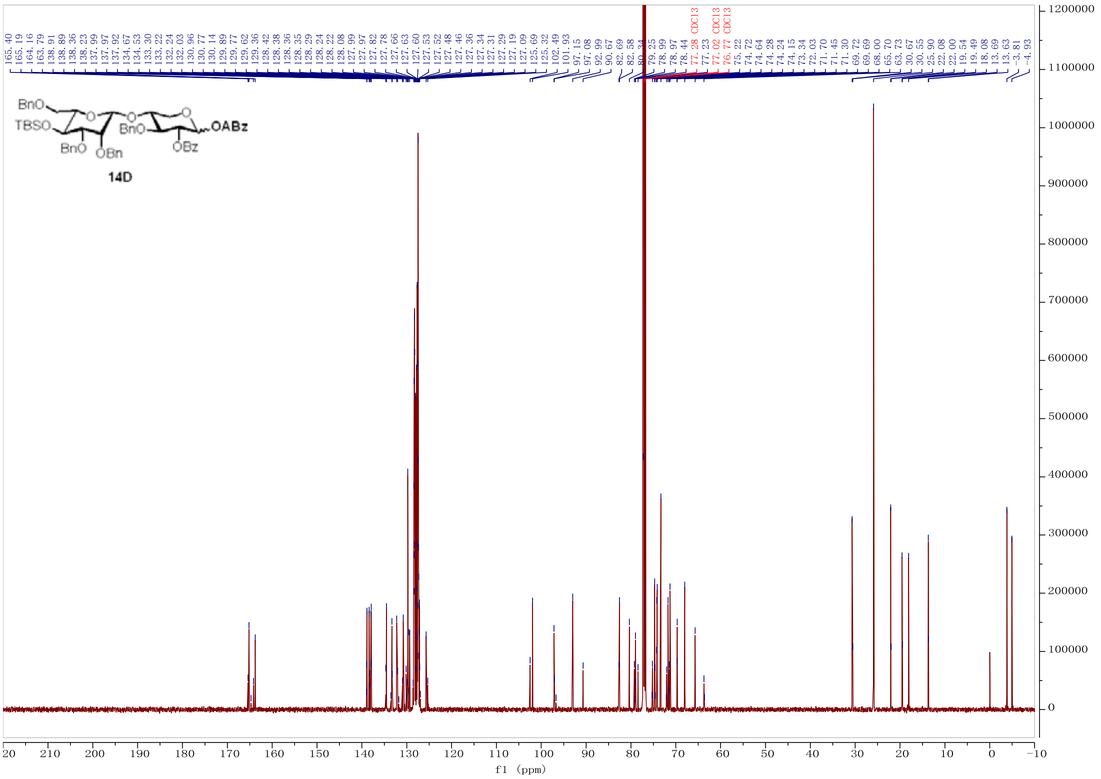

^{13}C NMR spectrum of 14D (126 MHz, $CDCl_3$, 25 °C)

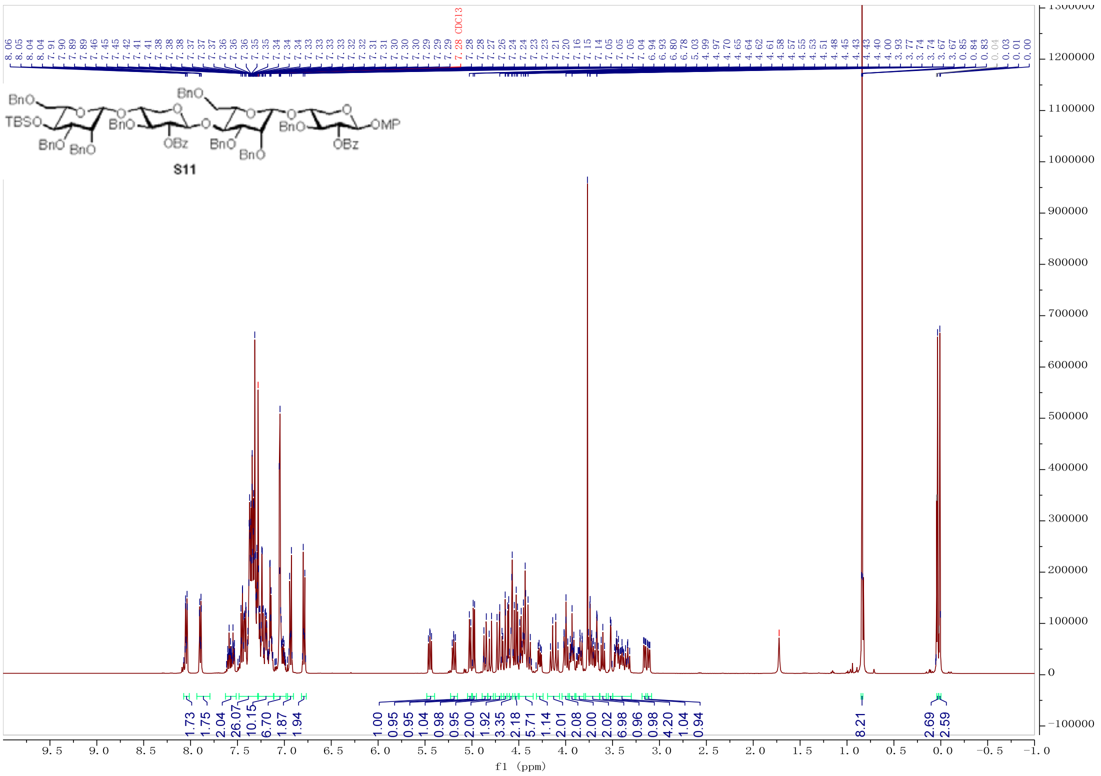

^{1}H NMR spectrum of S11 (500 MHz, $CDCl_3$, 25 °C)

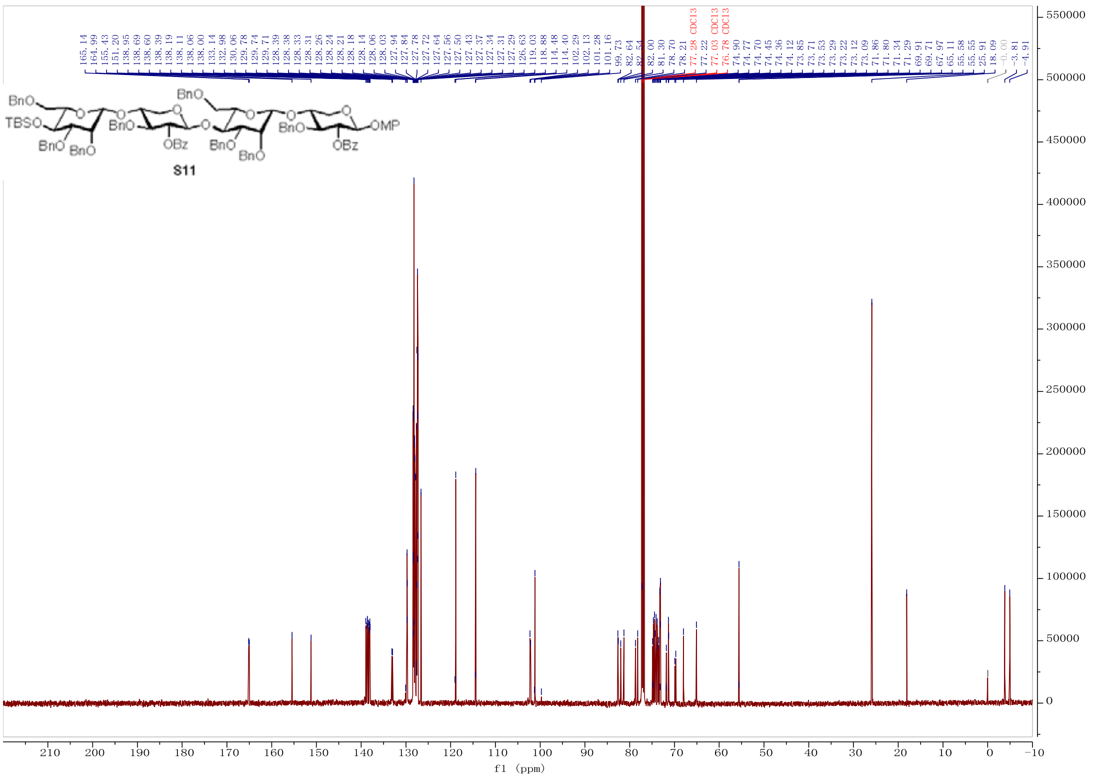

^{13}C NMR spectrum of S11 (126 MHz, $CDCl_3$, 25 °C)

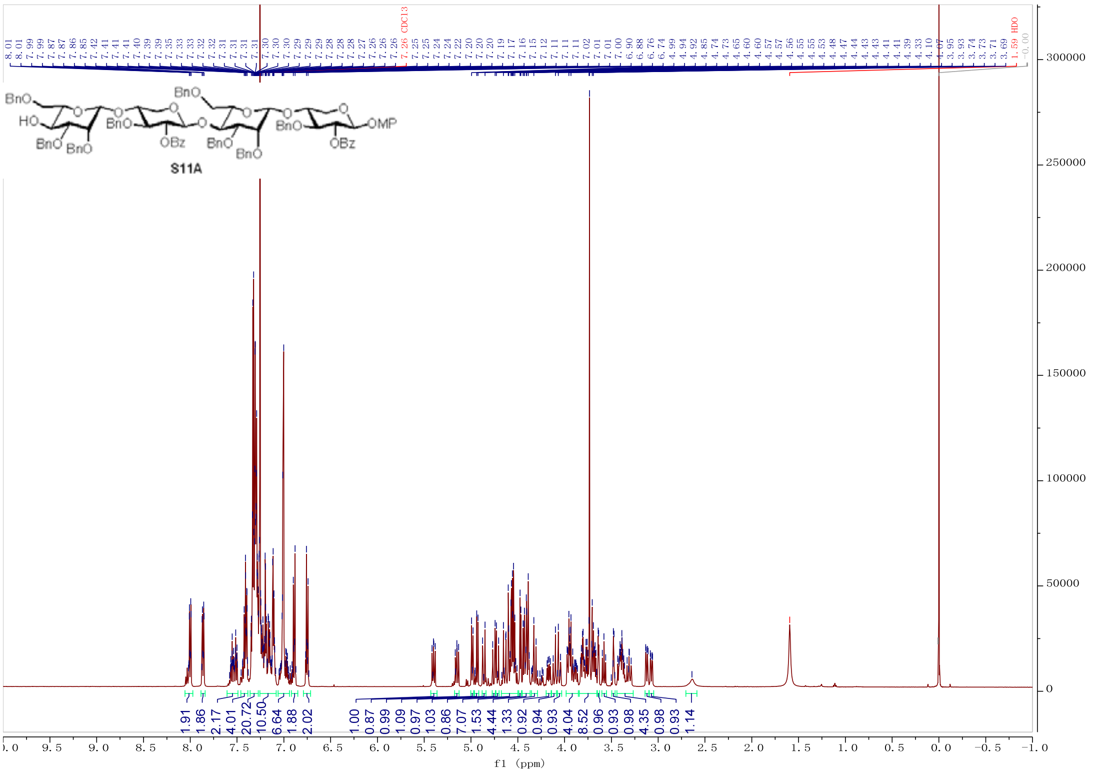

^{1}H NMR spectrum of S11A (500 MHz, $CDCl_3$, 25 °C)

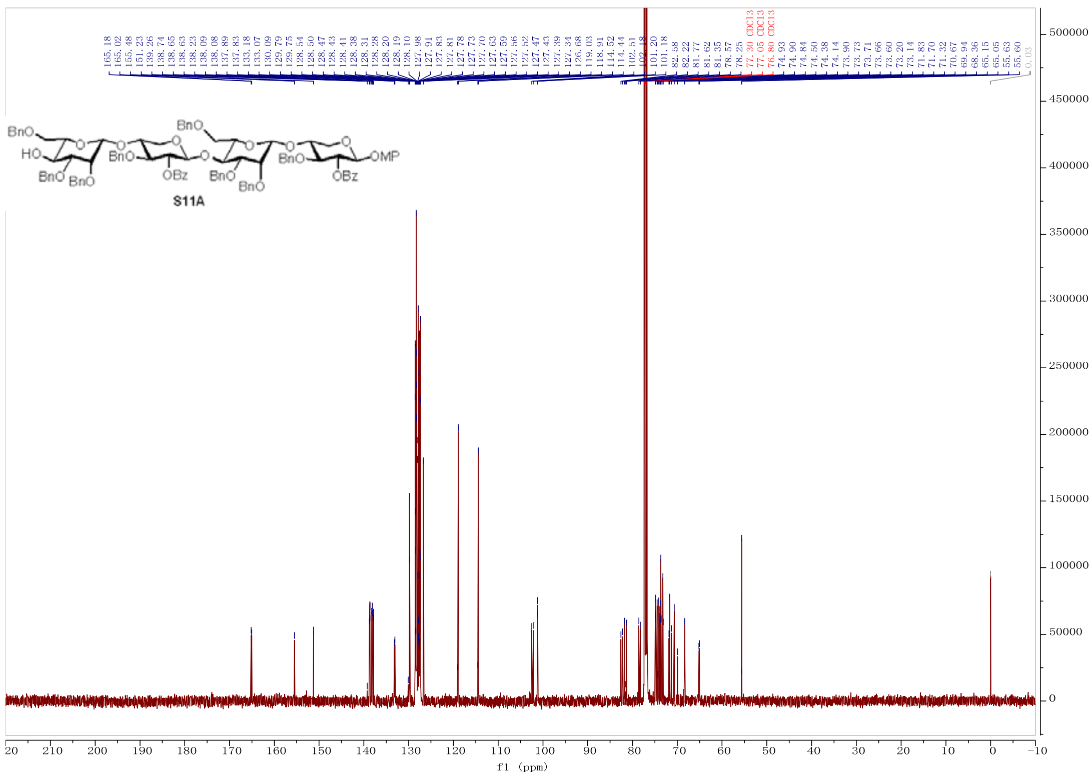

^{13}C NMR spectrum of S11A (126 MHz, $CDCl_3$, 25 °C)

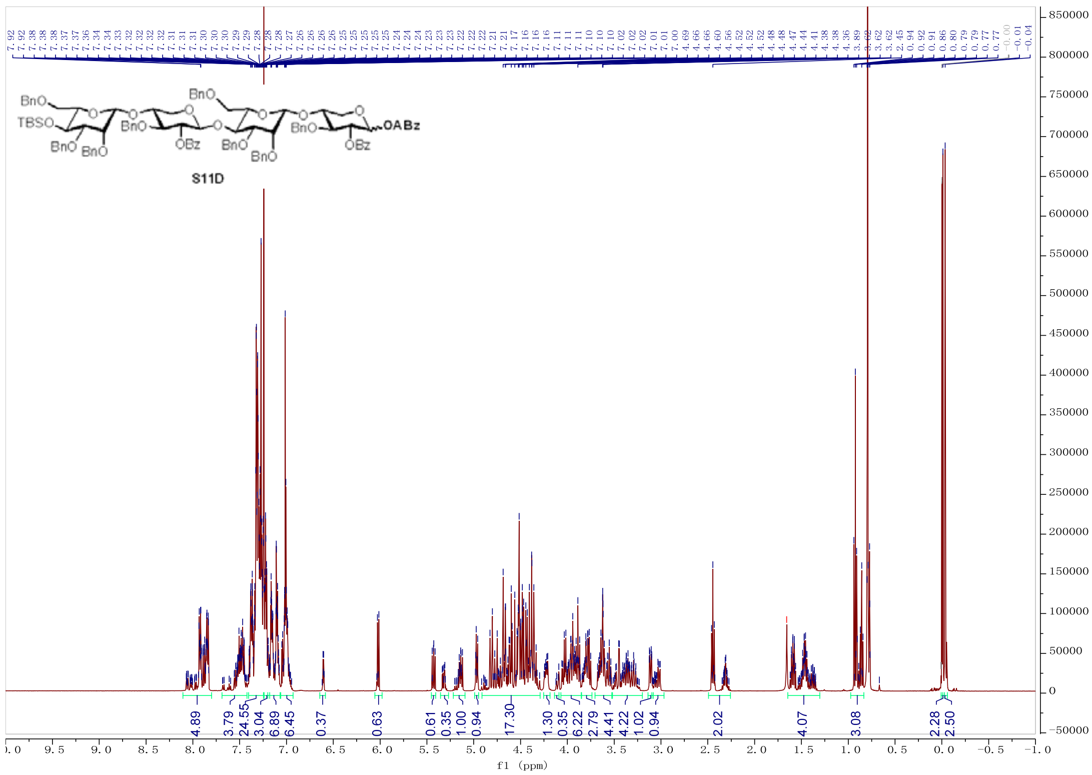

^{1}H NMR spectrum of S11D (500 MHz, $CDCl_3$, 25 °C)

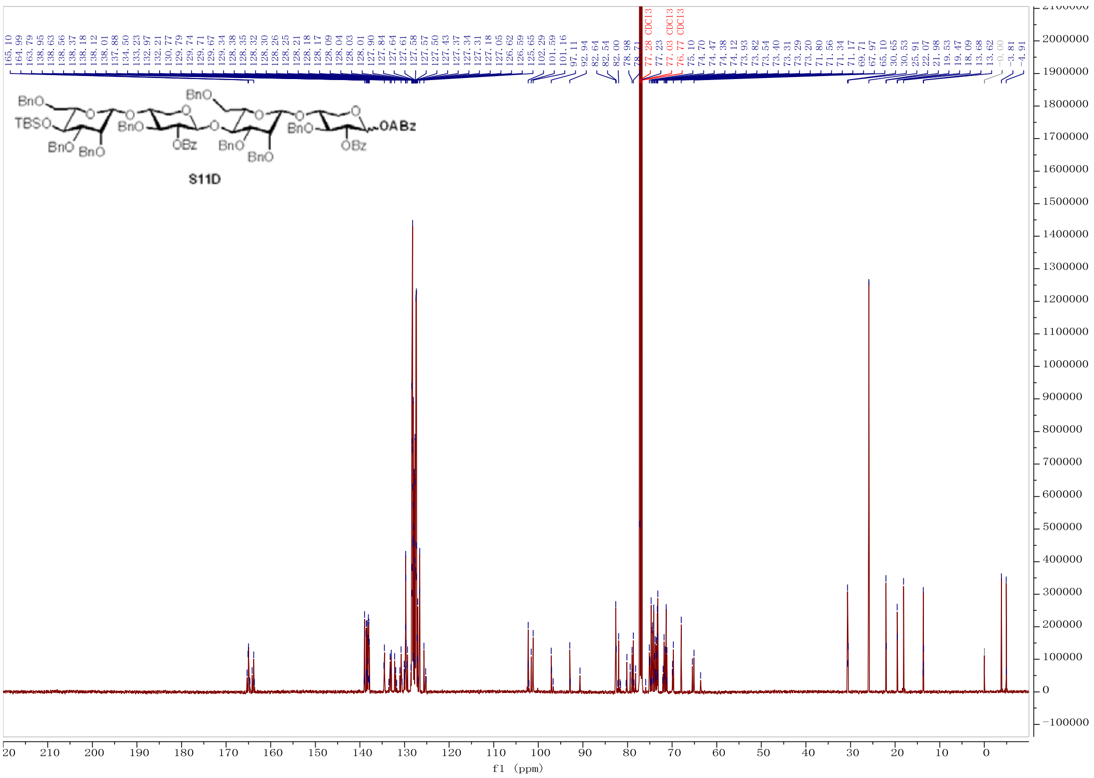

^{13}C NMR spectrum of S11D (126 MHz, $CDCl_3$, 25 °C)

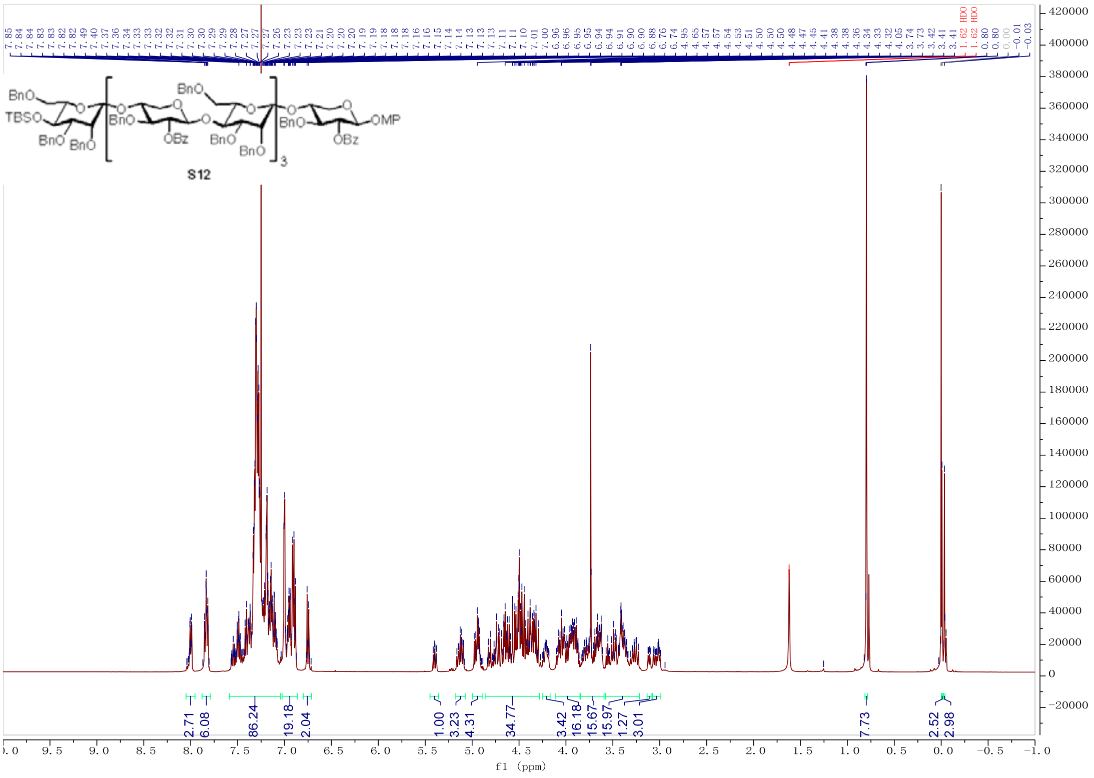

^{1}H NMR spectrum of S12 (500 MHz, $CDCl_3$, 25 °C)

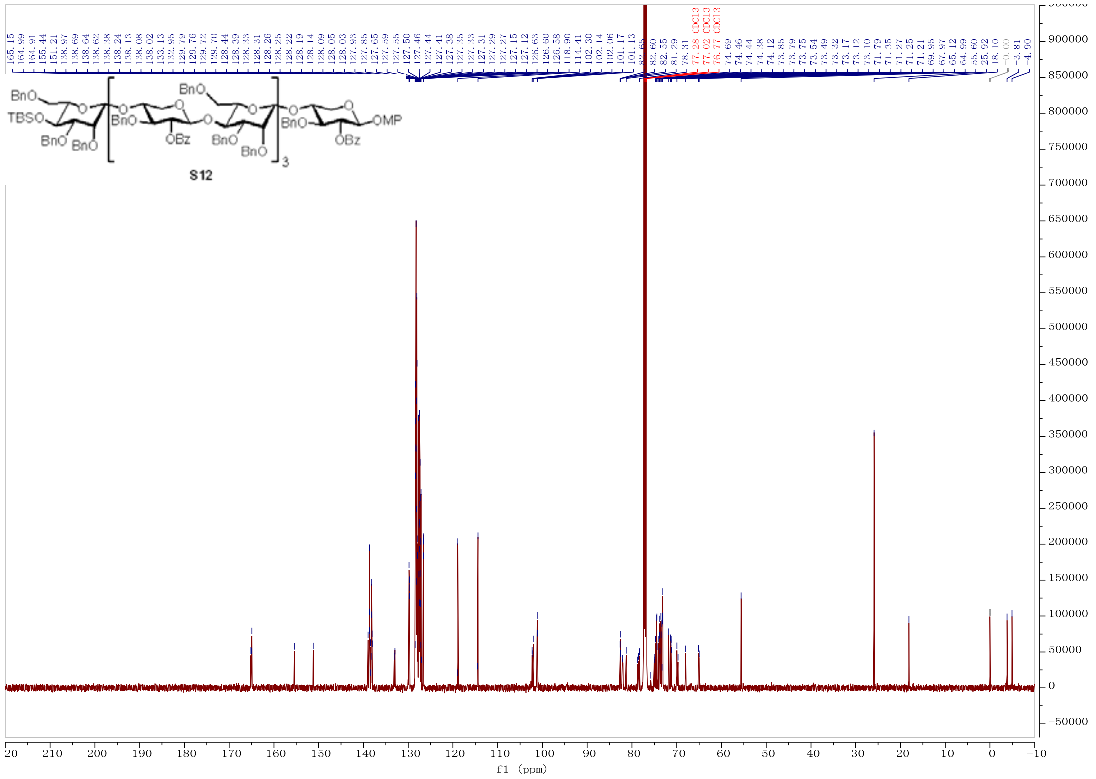

^{13}C NMR spectrum of S12 (126 MHz, $CDCl_3$, 25 °C)

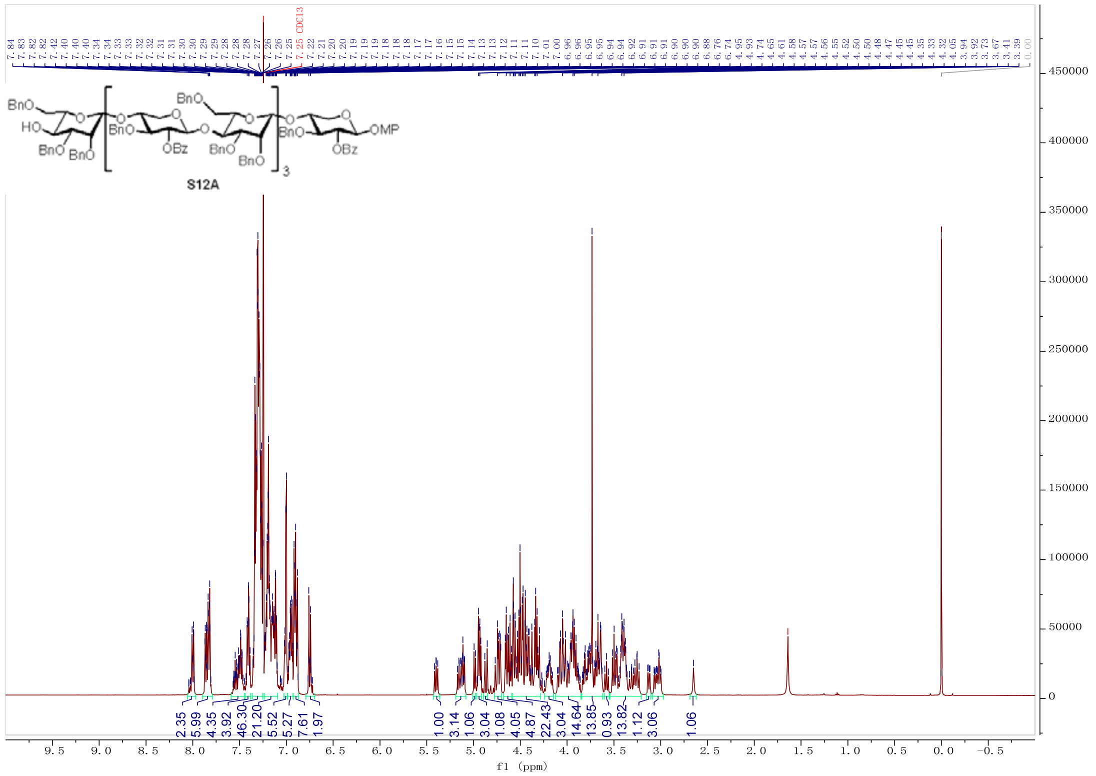

^{1}H NMR spectrum of S12A (500 MHz, $CDCl_3$, 25 °C)

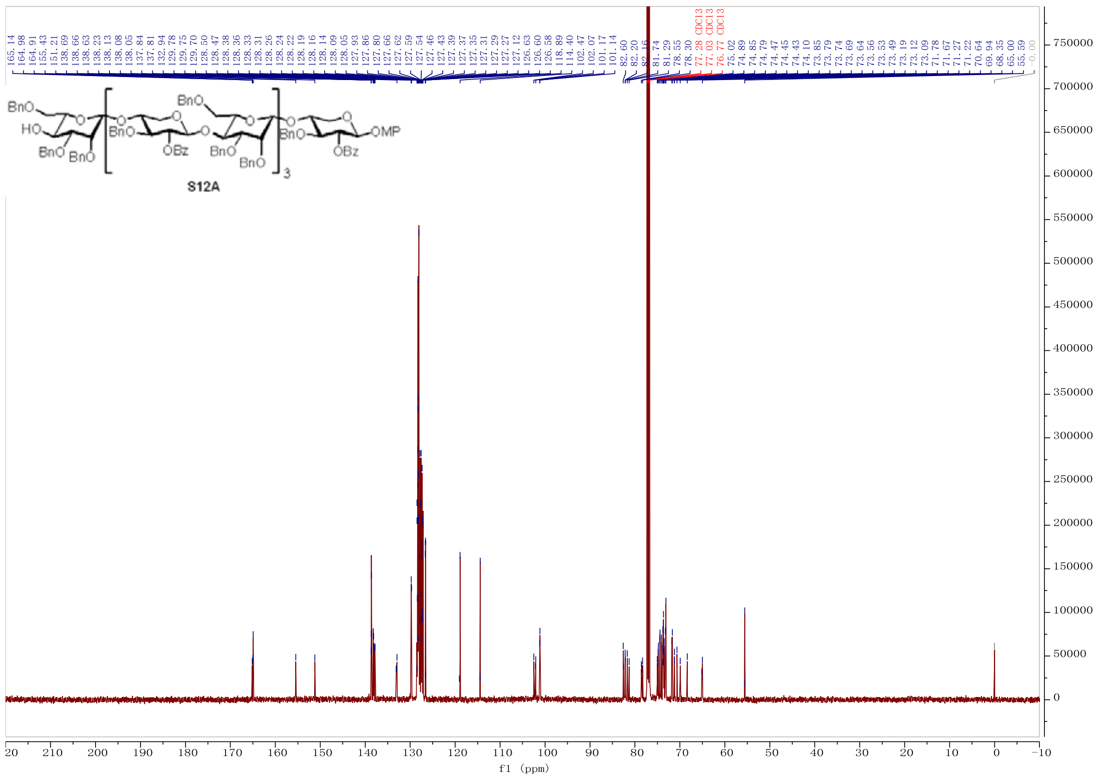

^{13}C NMR spectrum of S12A (126 MHz, $CDCl_3$, 25 °C)

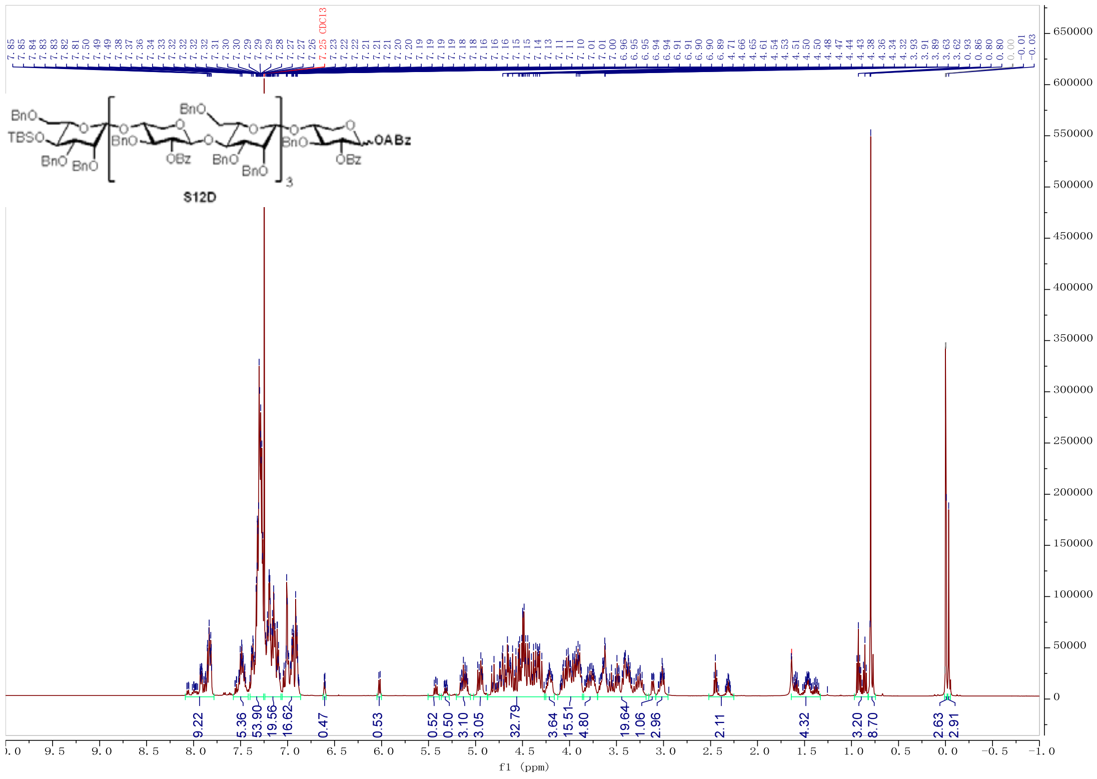

^{1}H NMR spectrum of S12D (500 MHz, $CDCl_3$, 25 °C)

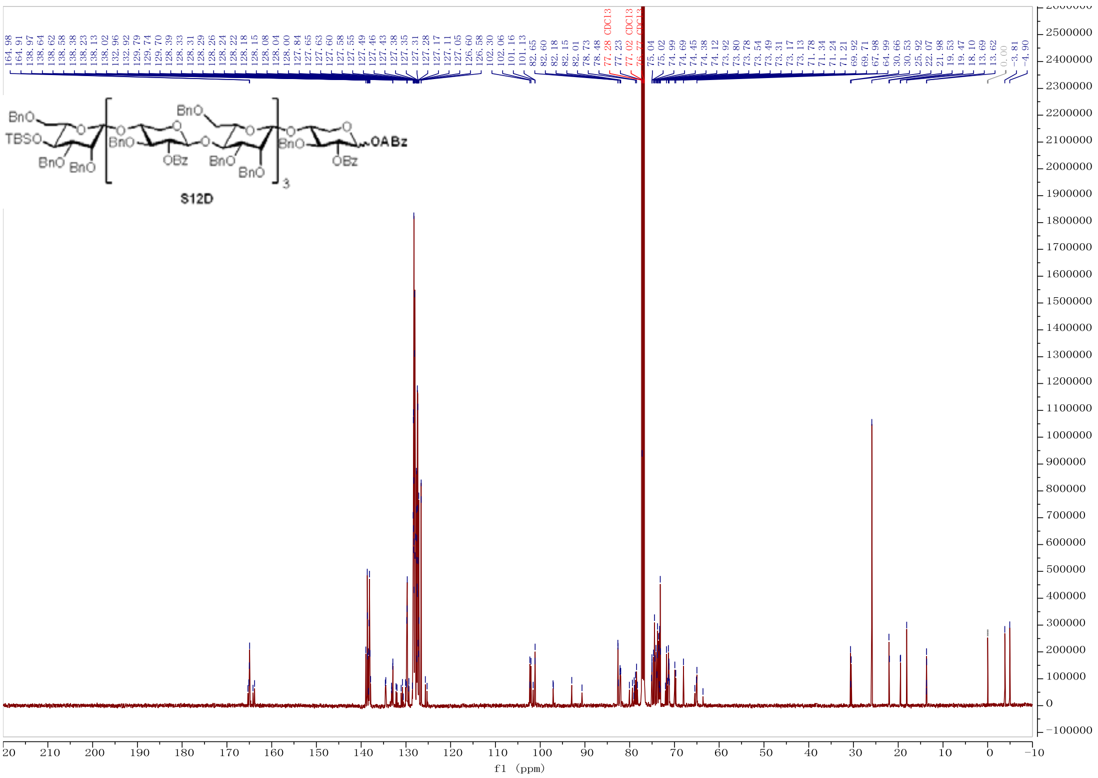

^{13}C NMR spectrum of S12D (126 MHz, $CDCl_3$, 25 °C)

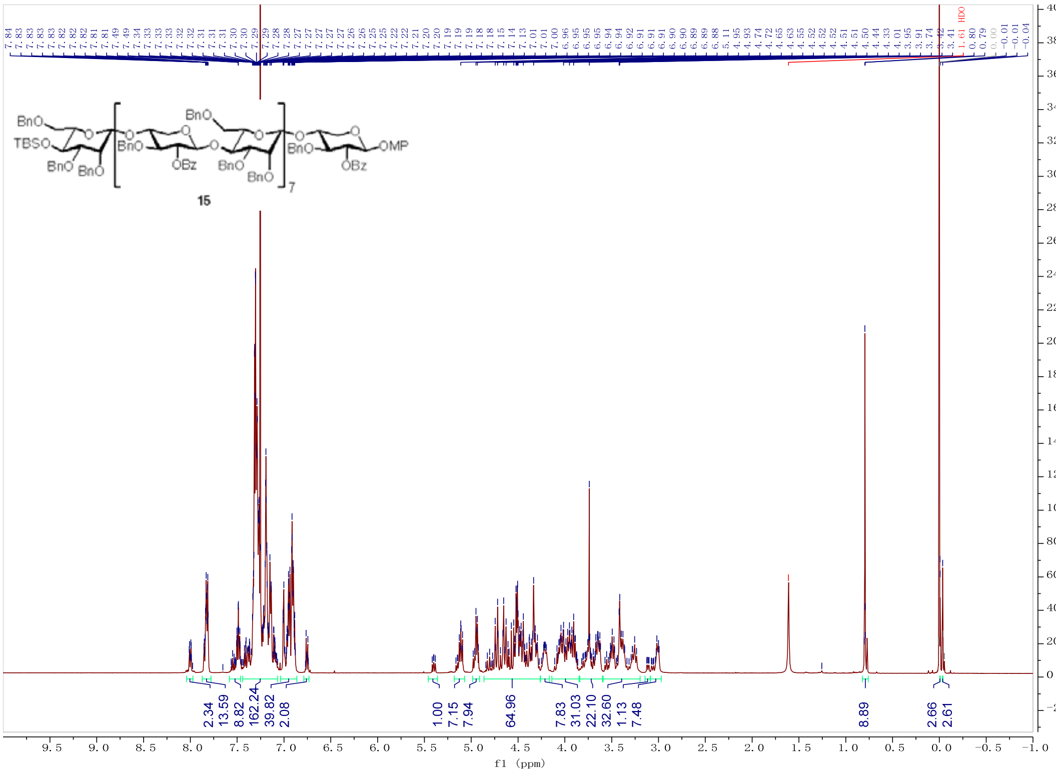

^{1}H NMR spectrum of 15 (500 MHz, $CDCl_3$, 25 °C)

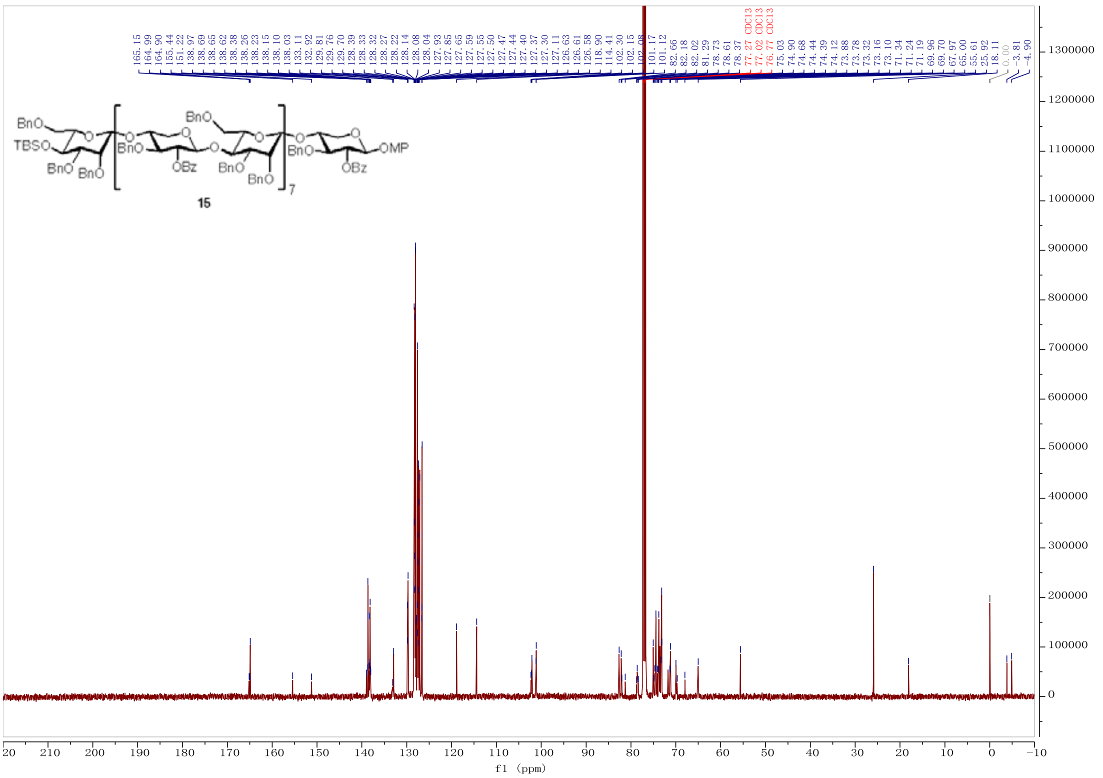

^{13}C NMR spectrum of 15 (126 MHz, $CDCl_3$, 25 °C)

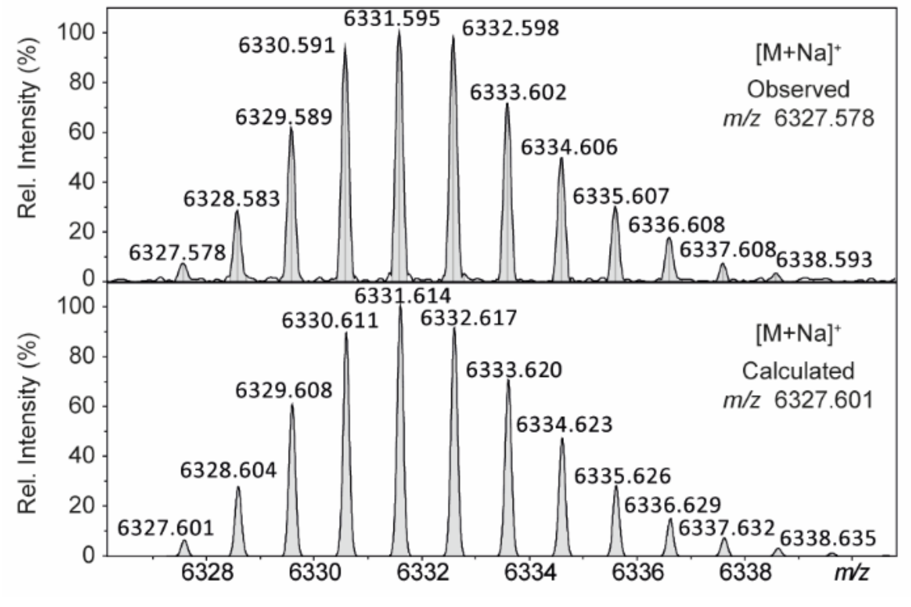

MALDI-FT-ICR MS spectrum of compound 15

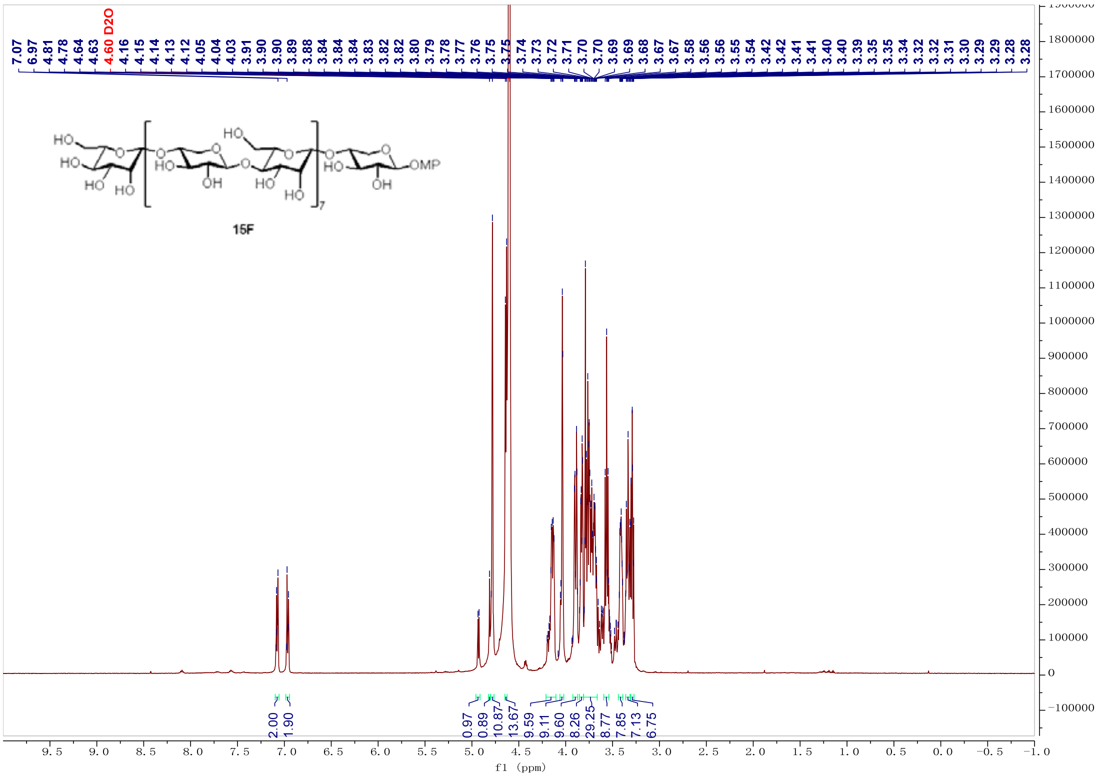

^{1}H NMR spectrum of compound 15F (600 MHz, 20 mM Na_3PO_4 buffered D_2O, 40 °C)

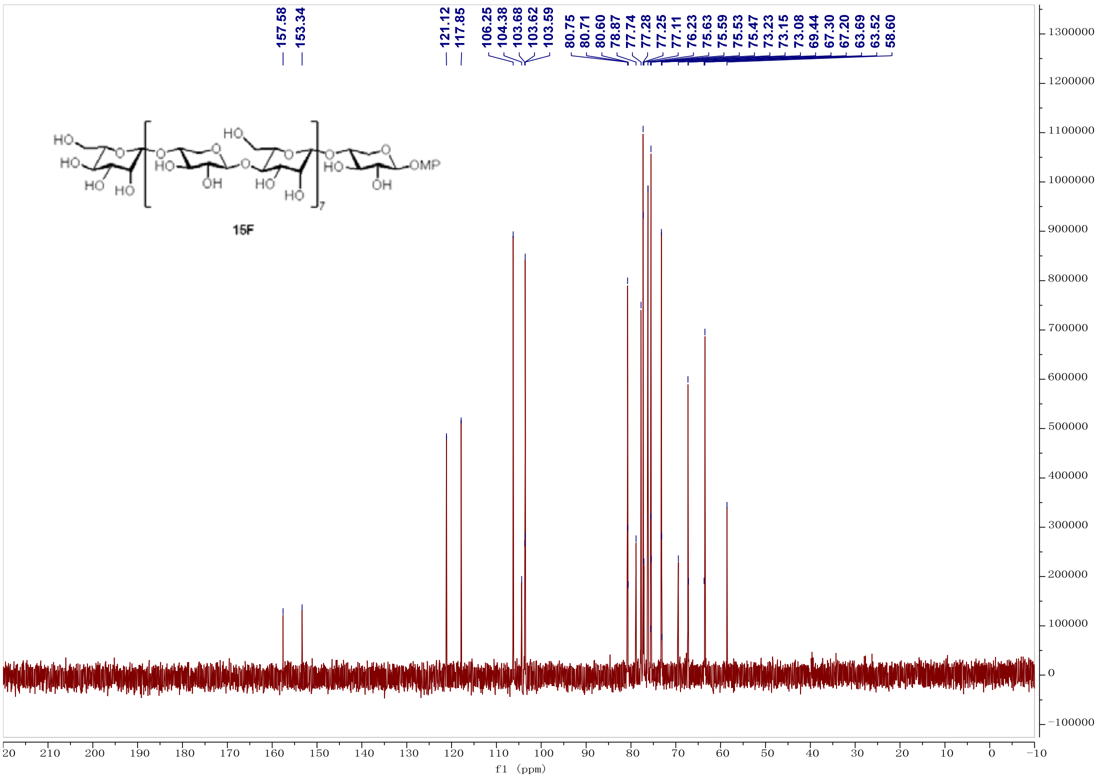

^{13}C NMR spectrum of compound 15F (151 MHz, 20 mM Na_3PO_4 buffered D_2O, 40 °C)

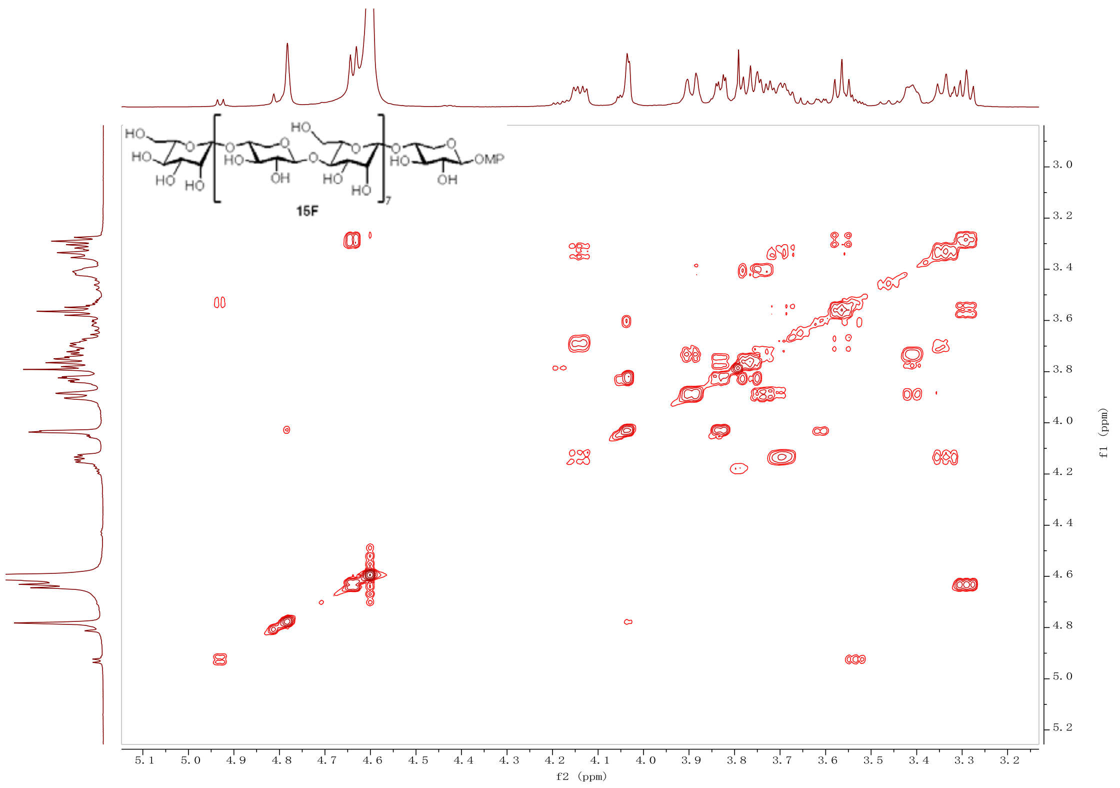

^{1}H-^{1}H gCOSY spectrum of compound 15F (600 MHz, 20 mM Na_3PO_4 buffered D_2O, 40 °C)

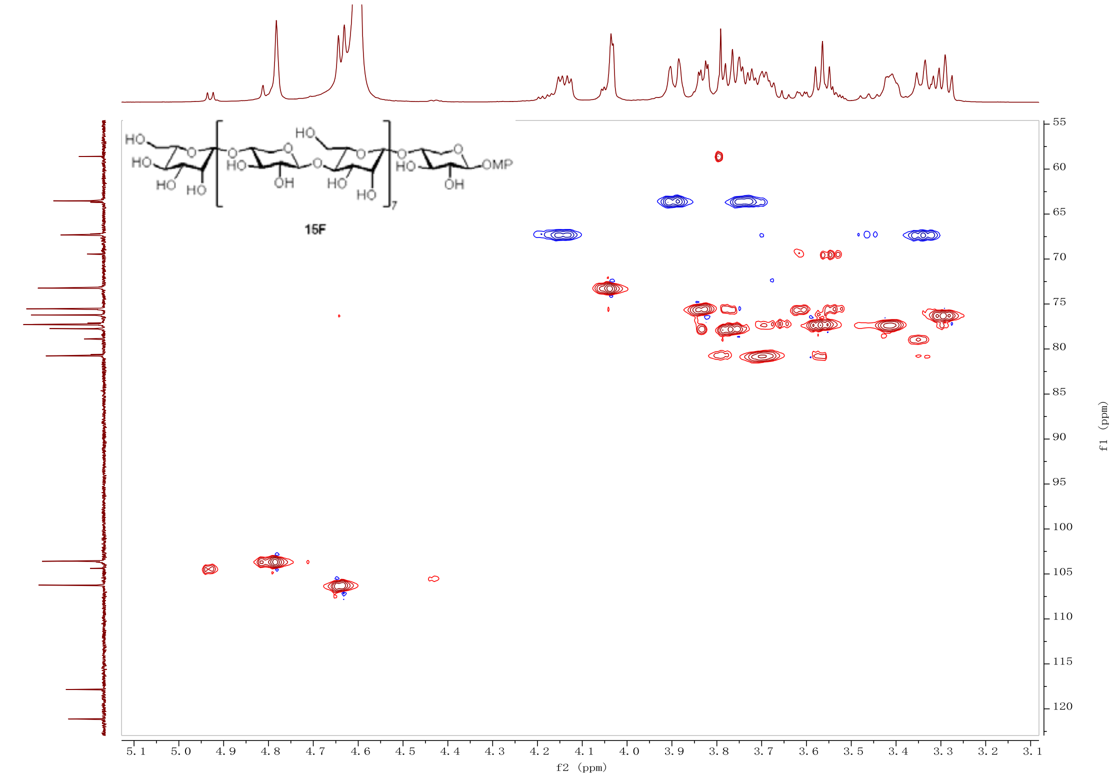

^{1}H-^{13}C HSQC spectrum of compound 15F (600 MHz, 20 mM Na_3PO_4 buffered D_2O, 40 °C)

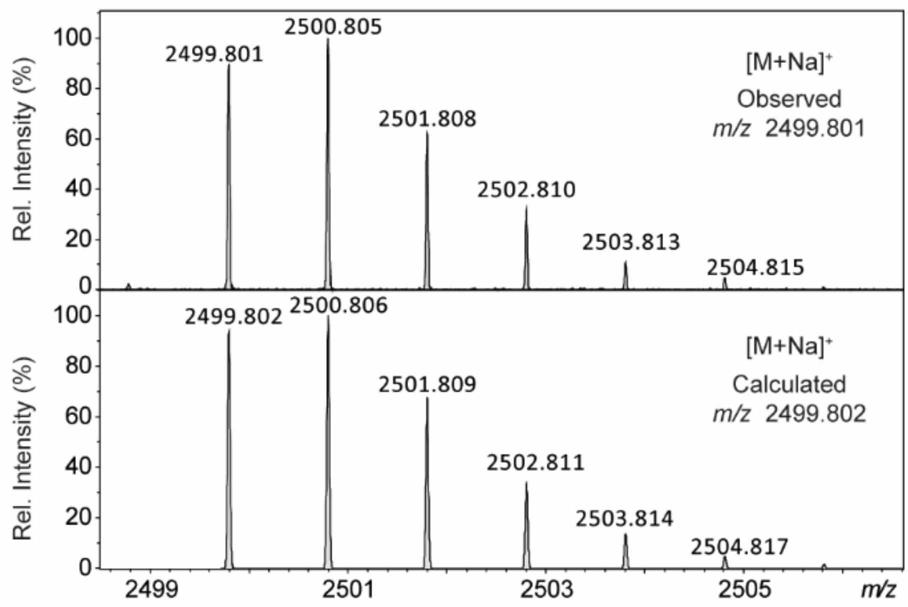

MALDI-FT-ICR MS spectrum of compound 15F

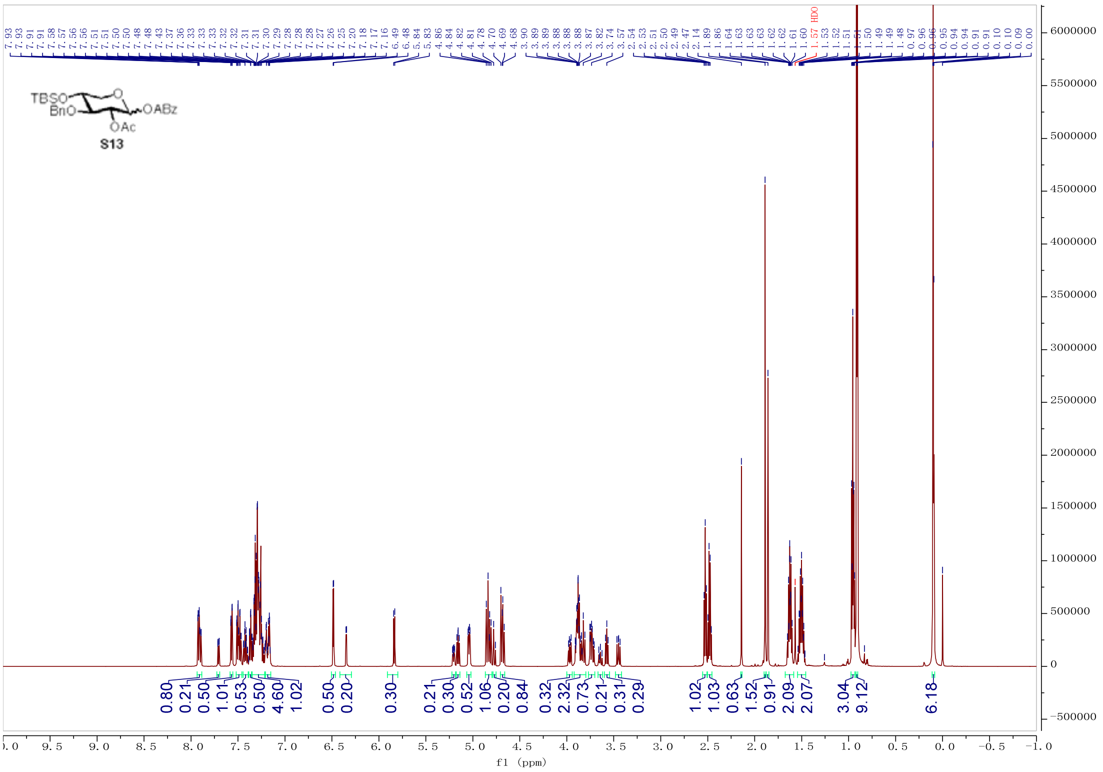

^{1}H NMR spectrum of S13 (600 MHz, $CDCl_3$, 25 °C)

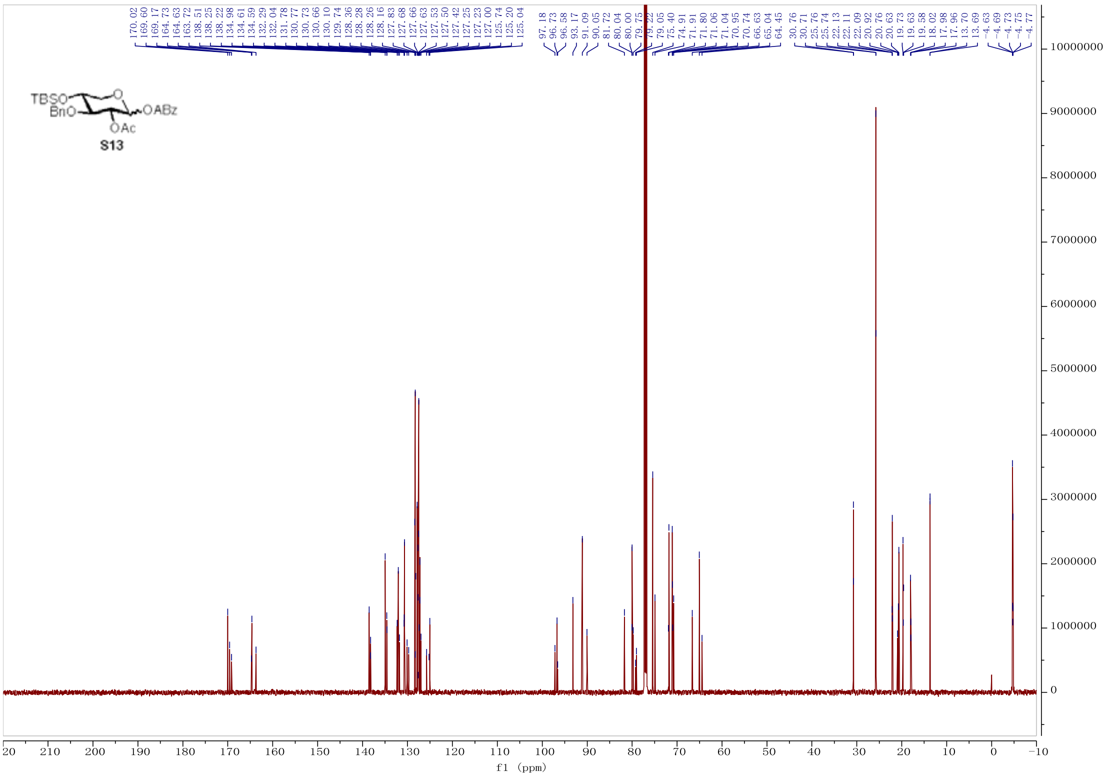

^{13}C NMR spectrum of S13 (151 MHz, $CDCl_3$, 25 °C)

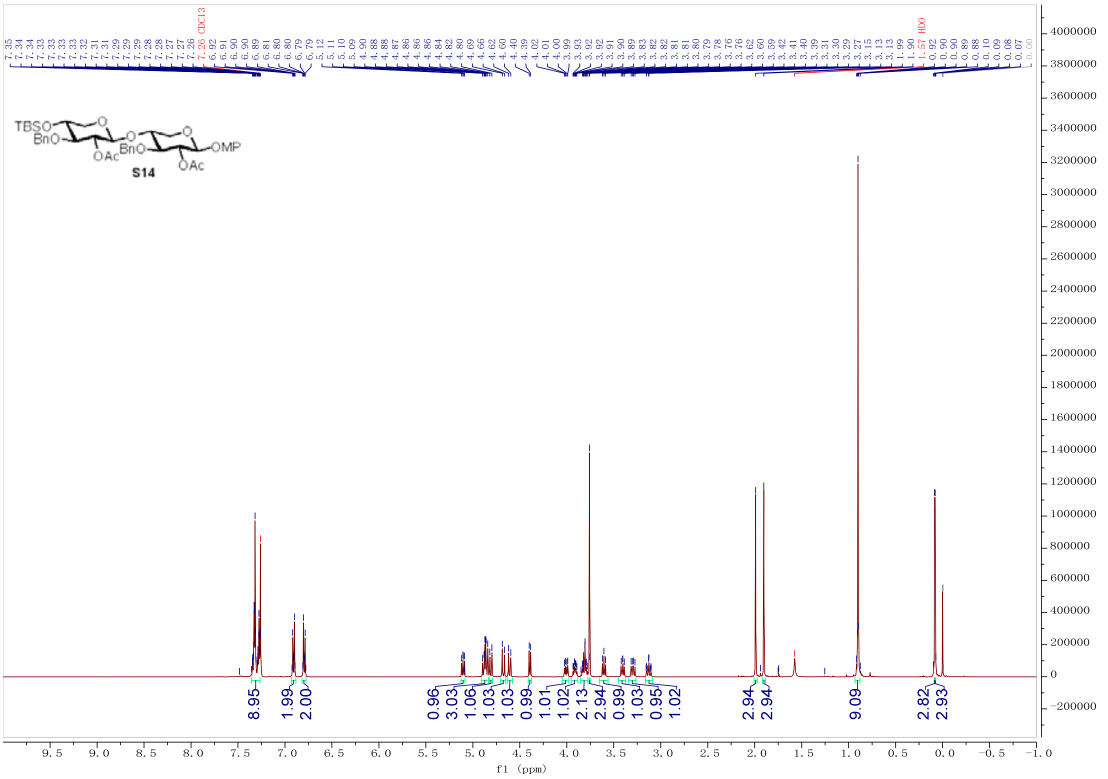

^{1}H NMR spectrum of S14 (500 MHz, $CDCl_3$, 25 °C)

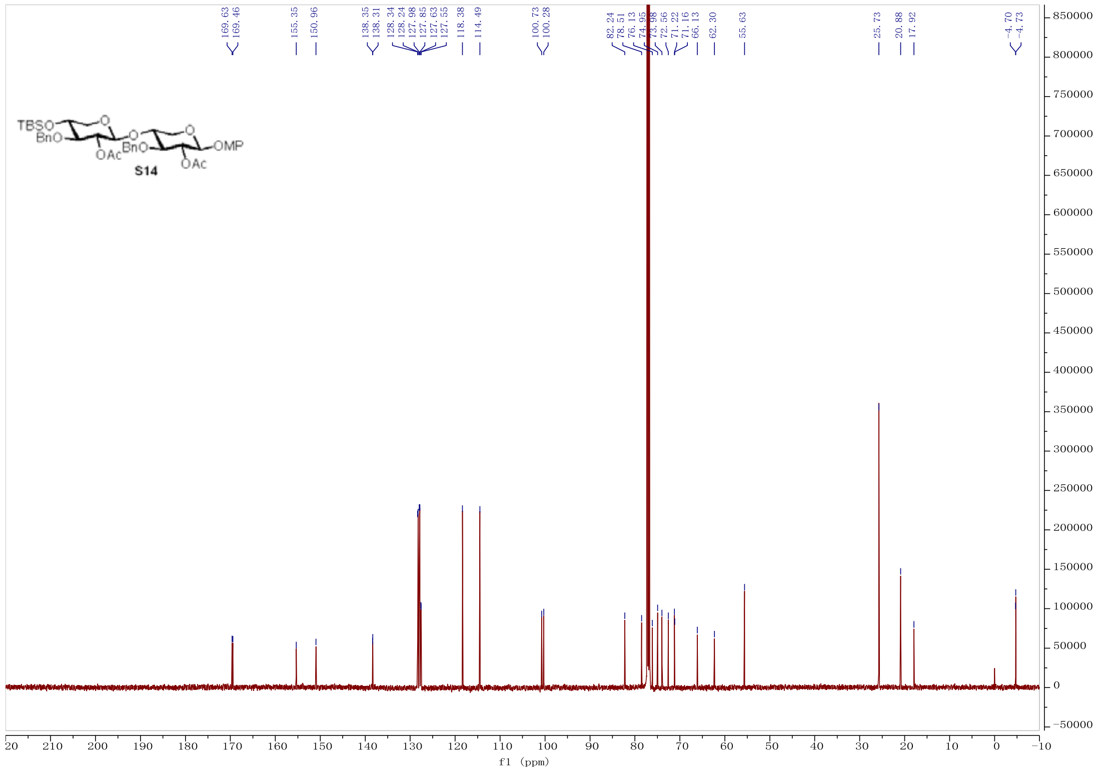

^{13}C NMR spectrum of S14 (126 MHz, $CDCl_3$, 25 °C)

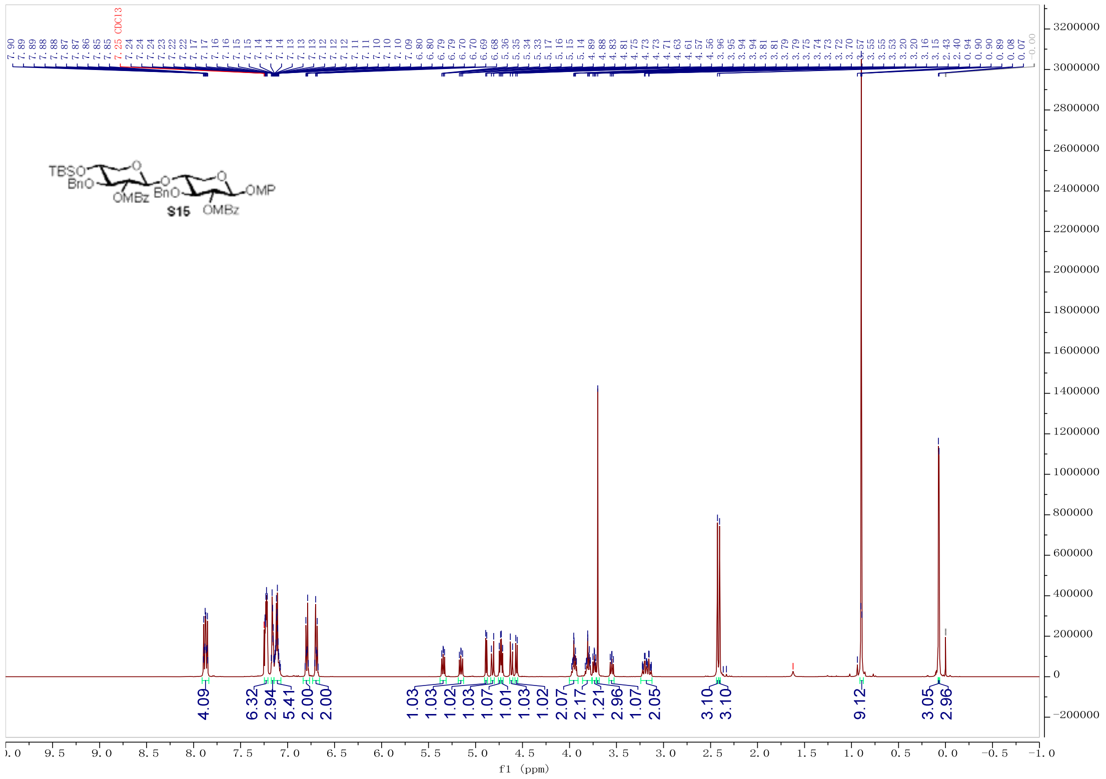

^{1}H NMR spectrum of S15 (500 MHz, $CDCl_3$, 25 °C)

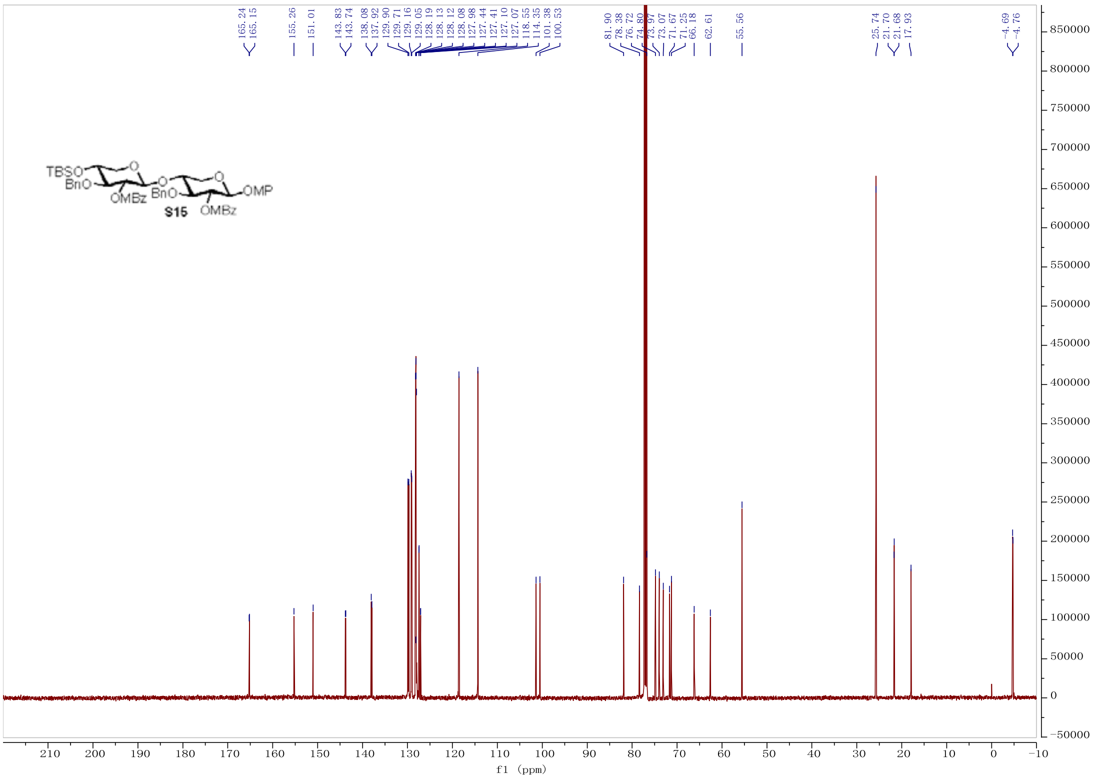

^{13}C NMR spectrum of S15 (126 MHz, $CDCl_3$, 25 °C)

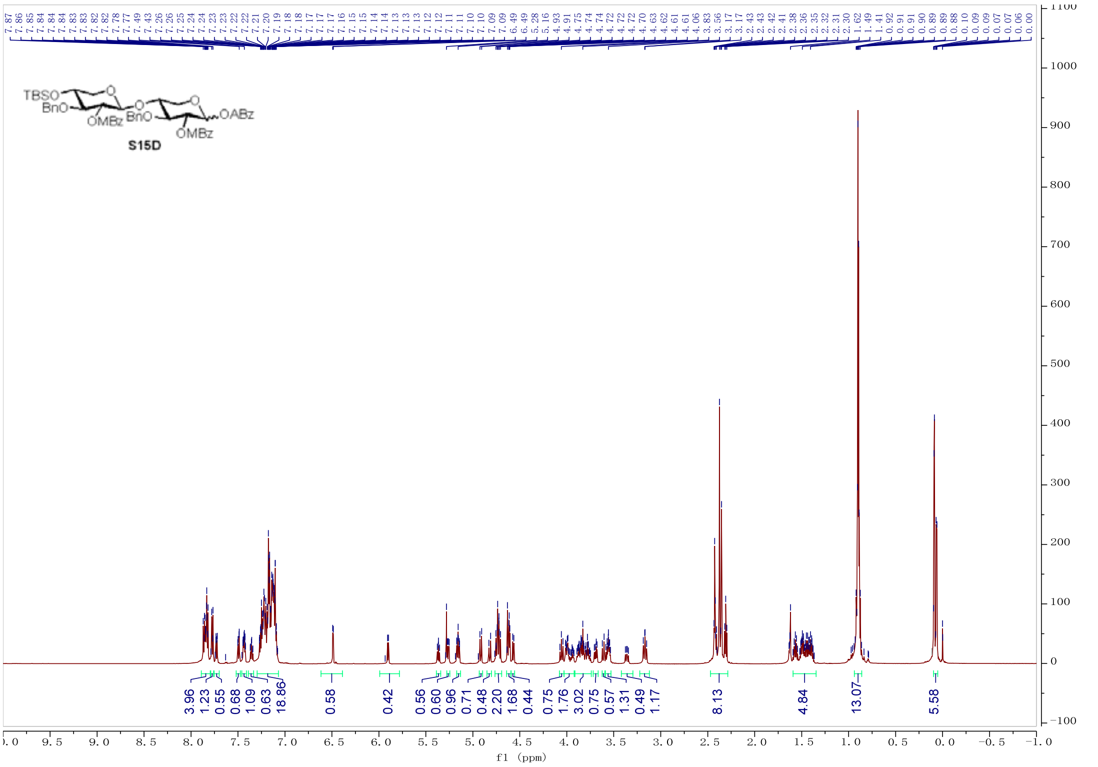

^{1}H NMR spectrum of S15D (600 MHz, $CDCl_3$, 25 °C)

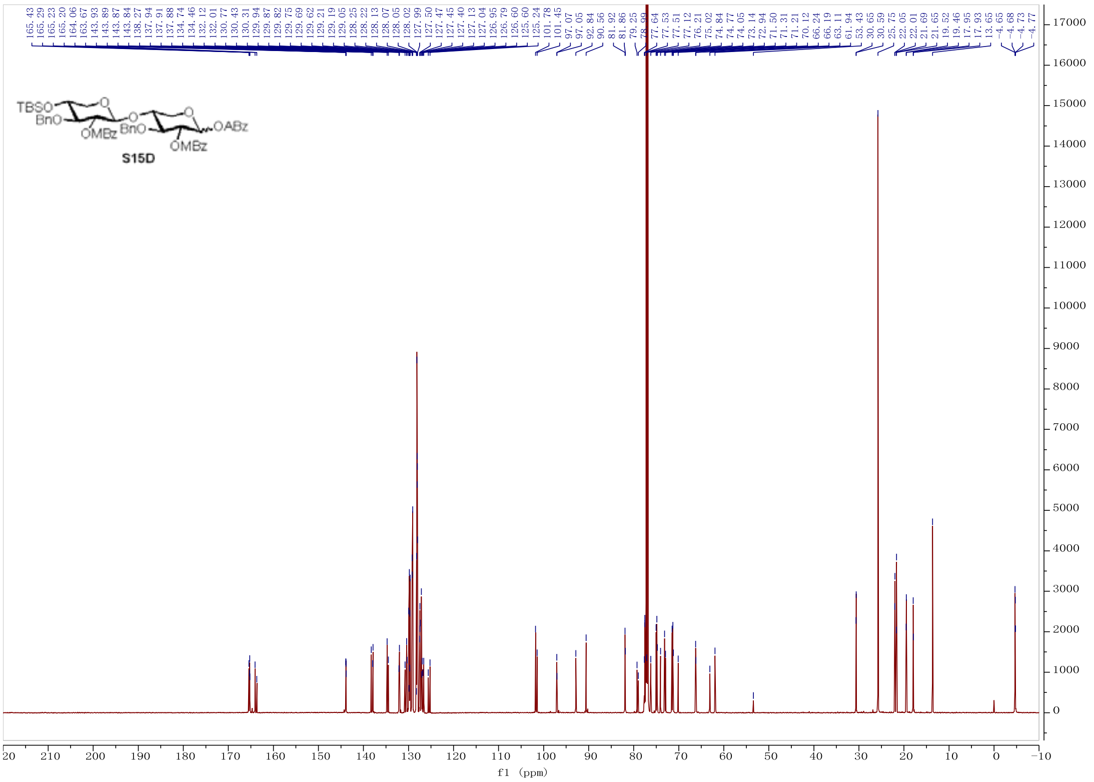

^{13}C NMR spectrum of S15D (151 MHz, $CDCl_3$, 25 °C)

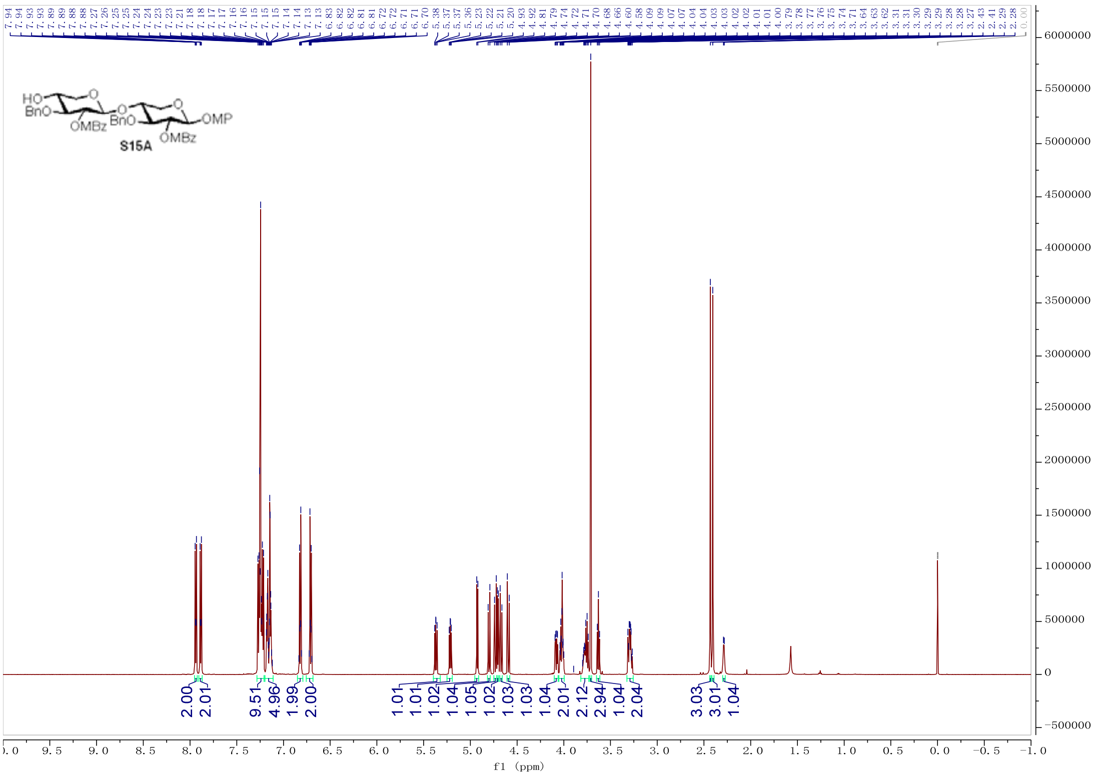

^{1}H NMR spectrum of S15A (600 MHz, $CDCl_3$, 25 °C)

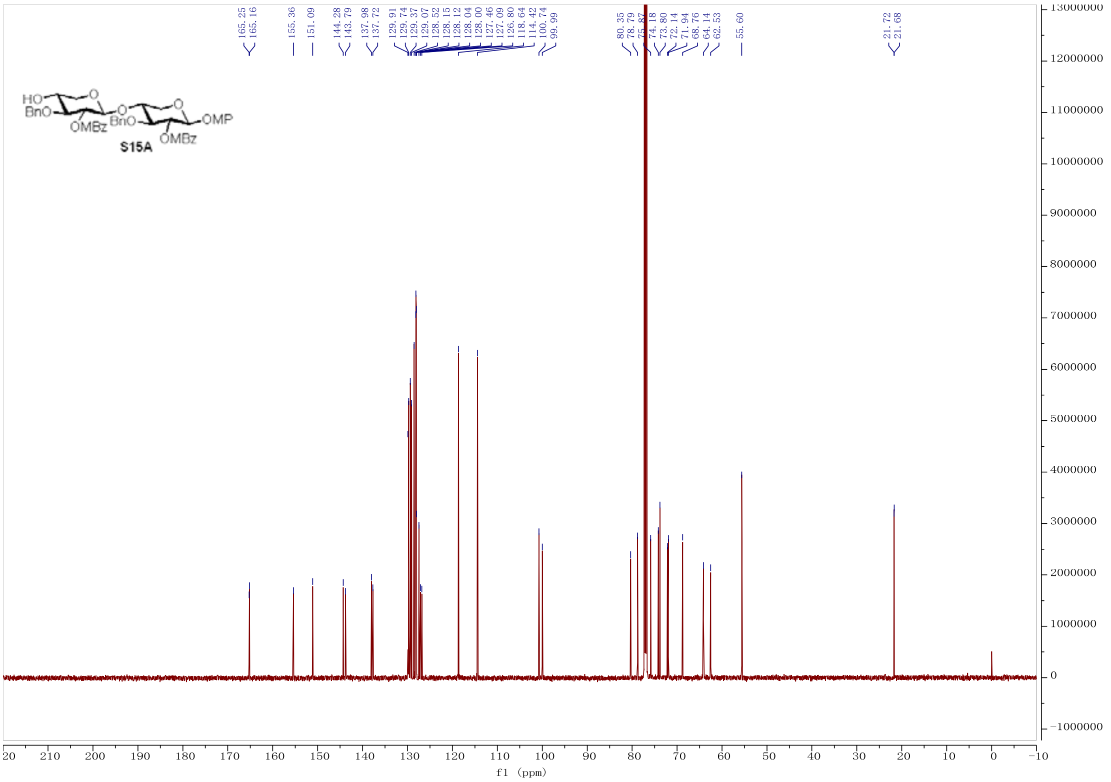

^{13}C NMR spectrum of S15A (151 MHz, $CDCl_3$, 25 °C)

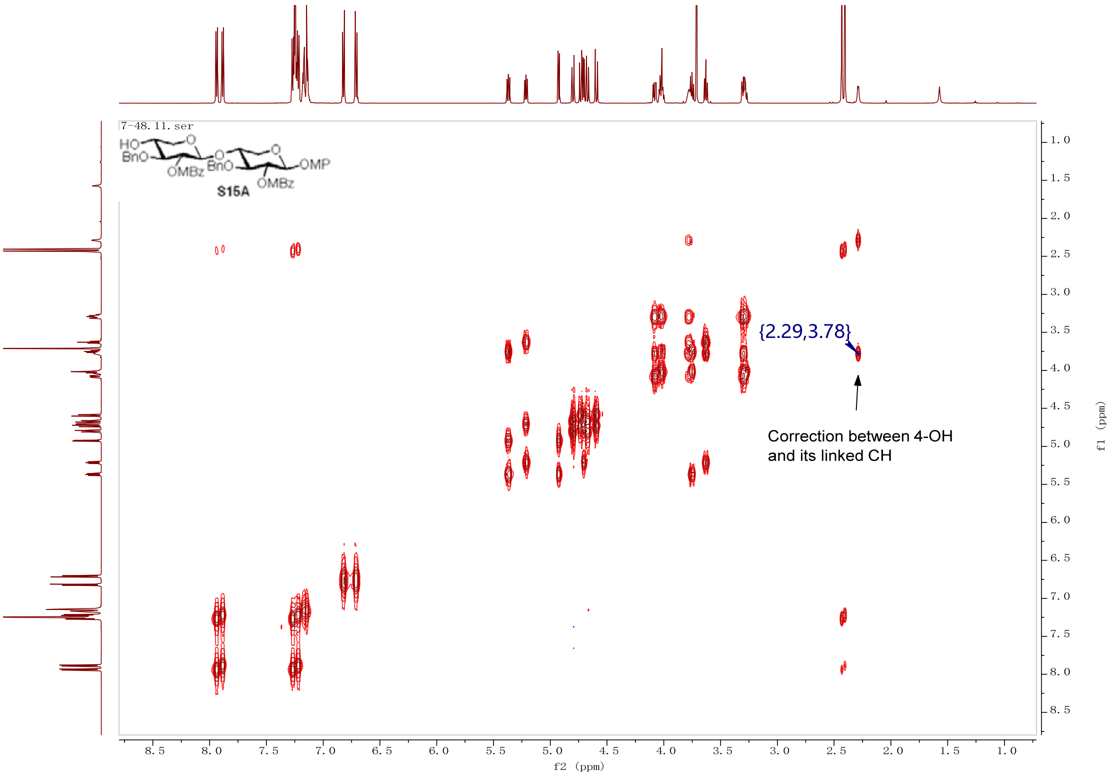

^{1}H-^{1}H gCOSY spectrum of S15A (600 MHz, $CDCl_3$, 25 °C)

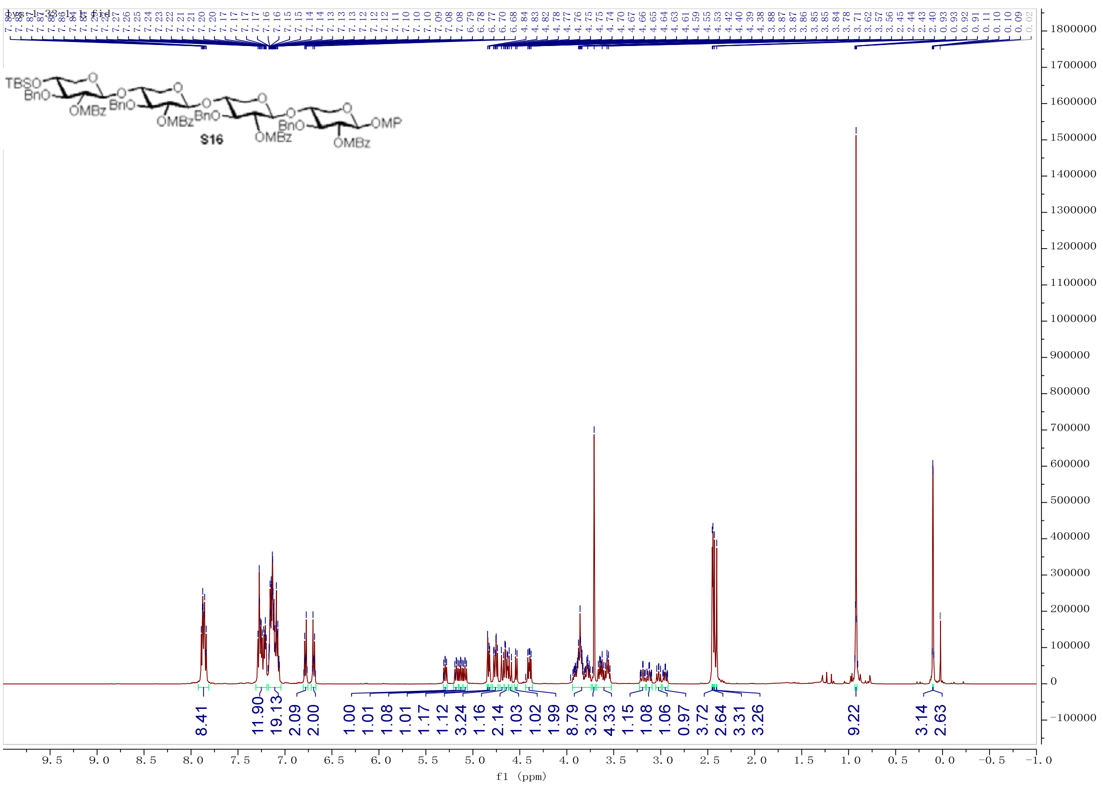

^{1}H NMR spectrum of S16 (500 MHz, $CDCl_3$, 25 °C)

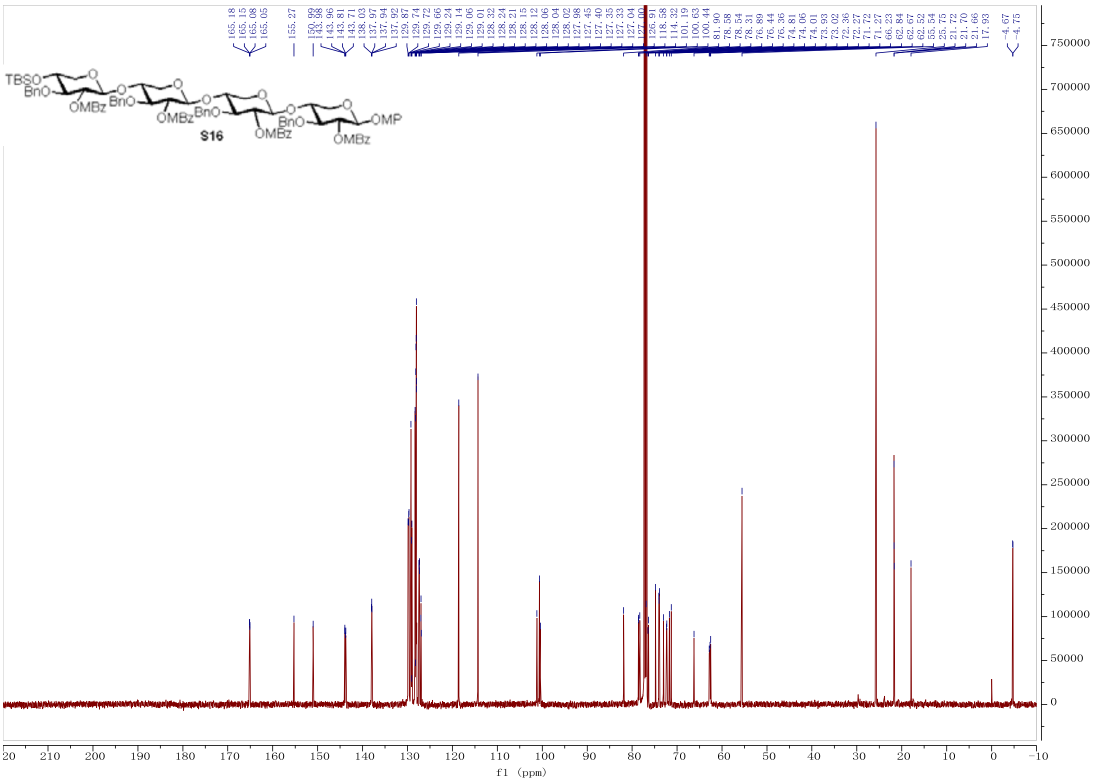

^{13}C NMR spectrum of S16 (126 MHz, $CDCl_3$, 25 °C)

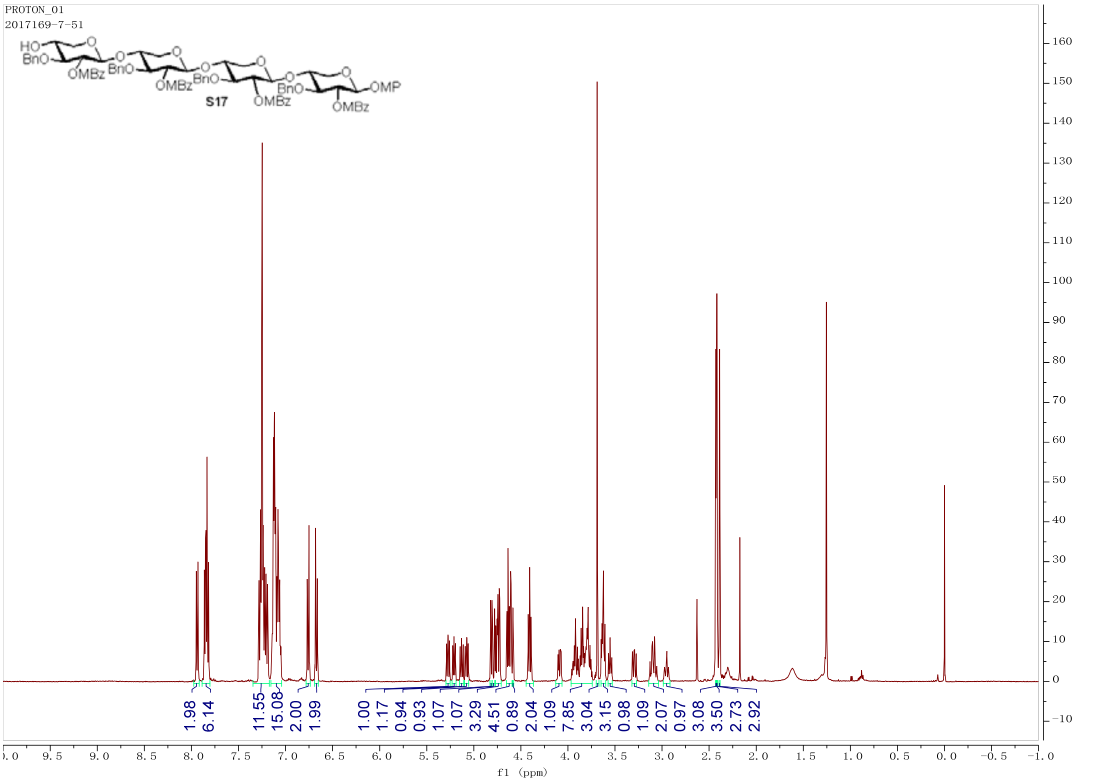

^{1}H NMR spectrum of S17 (500 MHz, $CDCl_3$, 25 °C)

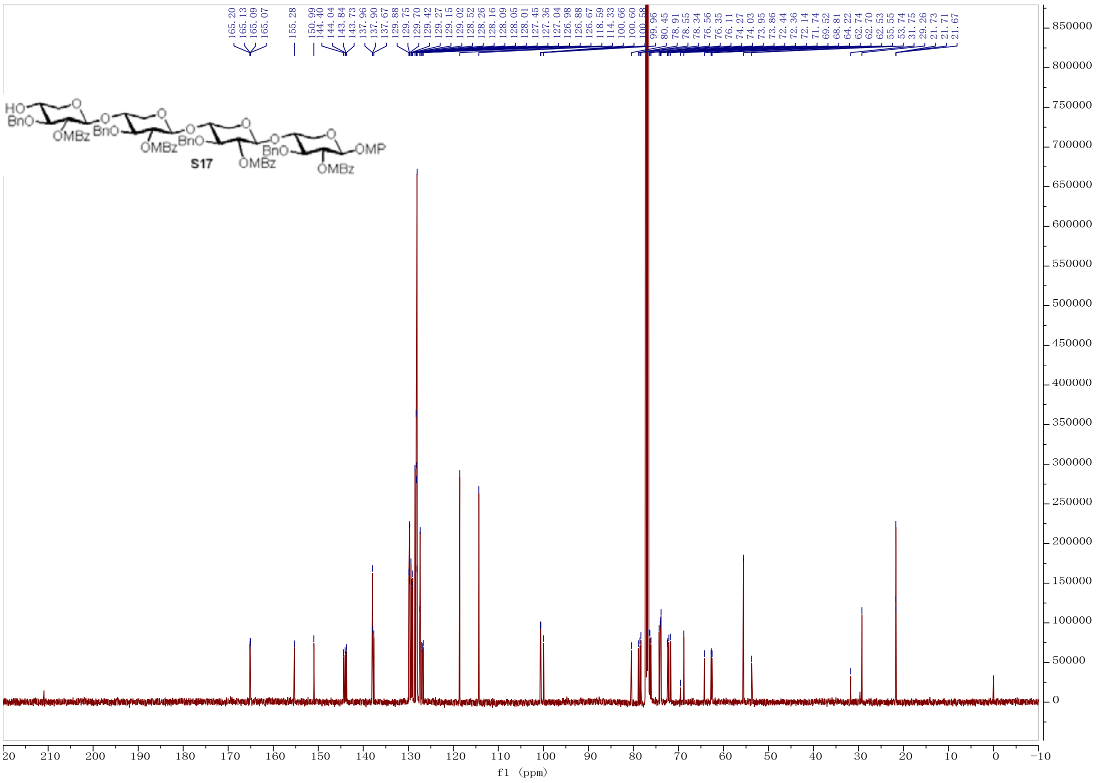

^{13}C NMR spectrum of S17 (126 MHz, $CDCl_3$, 25 °C)

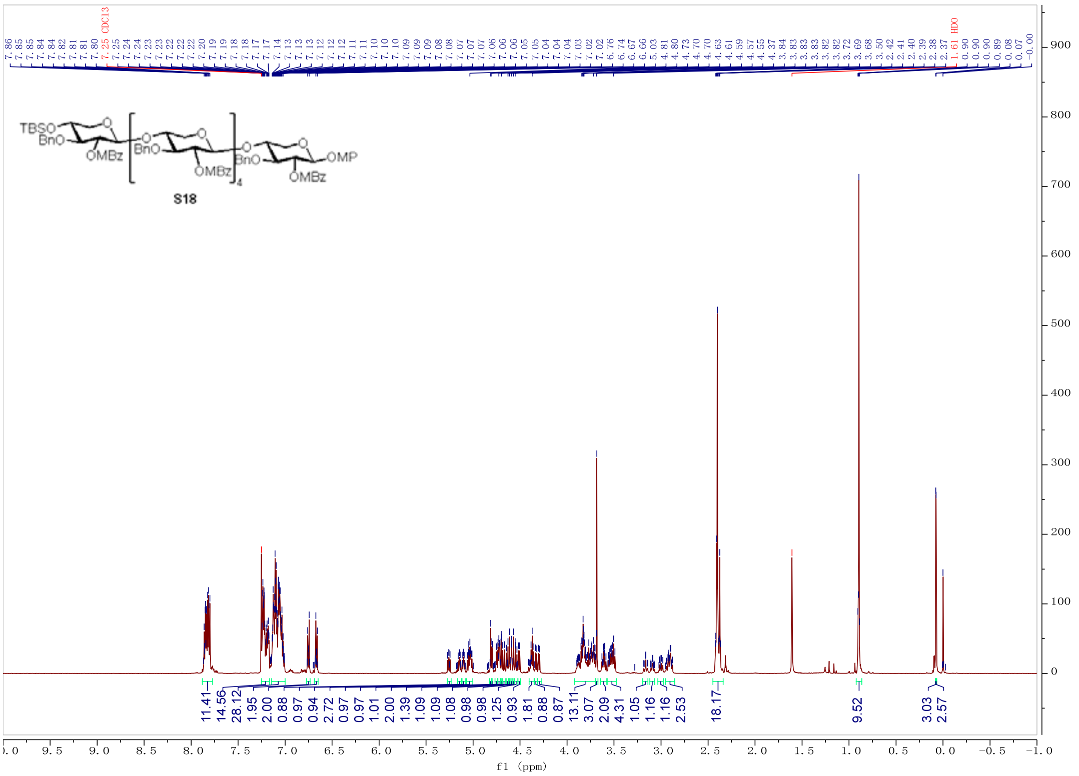

^{1}H NMR spectrum of S18 (600 MHz, $CDCl_3$, 25 °C)

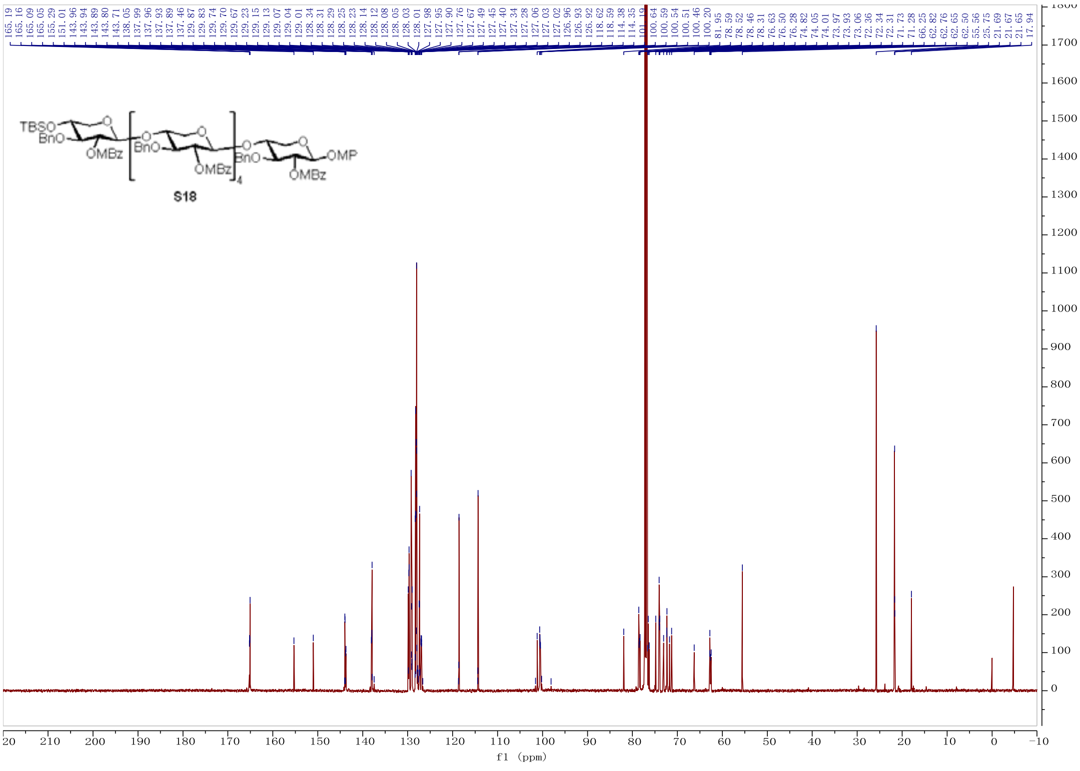

^{13}C NMR spectrum of S18 (151 MHz, $CDCl_3$, 25 °C)

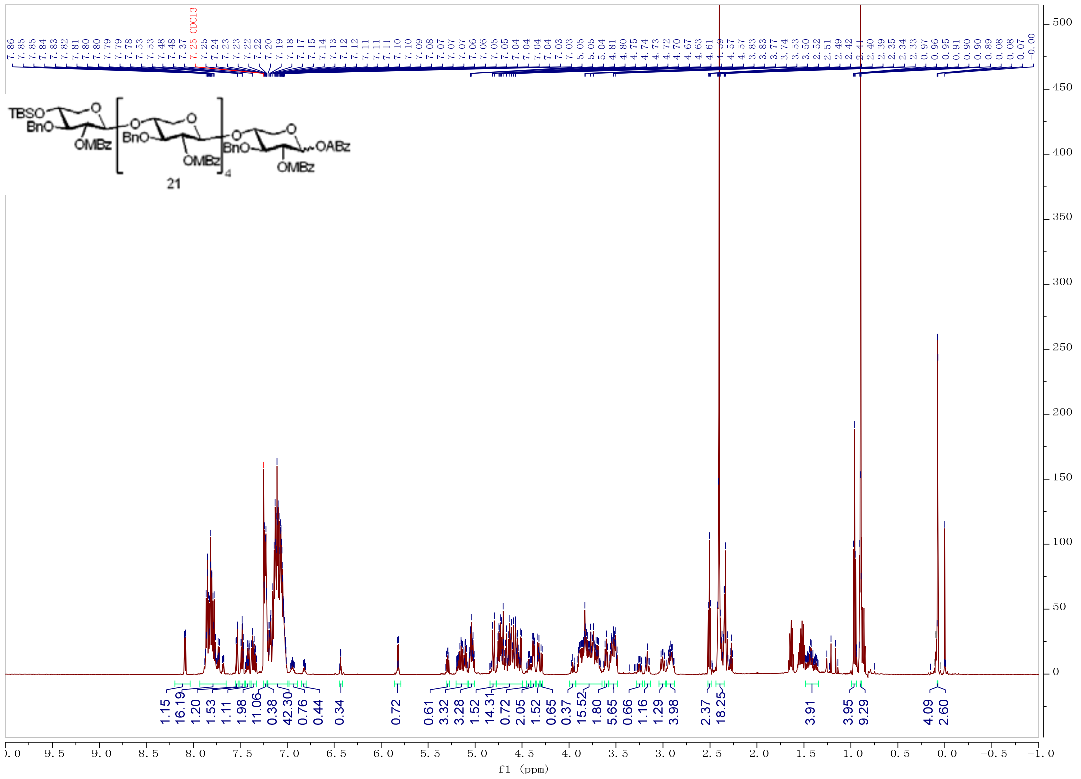

^{1}H NMR spectrum of 21 (600 MHz, $CDCl_3$, 25 °C)

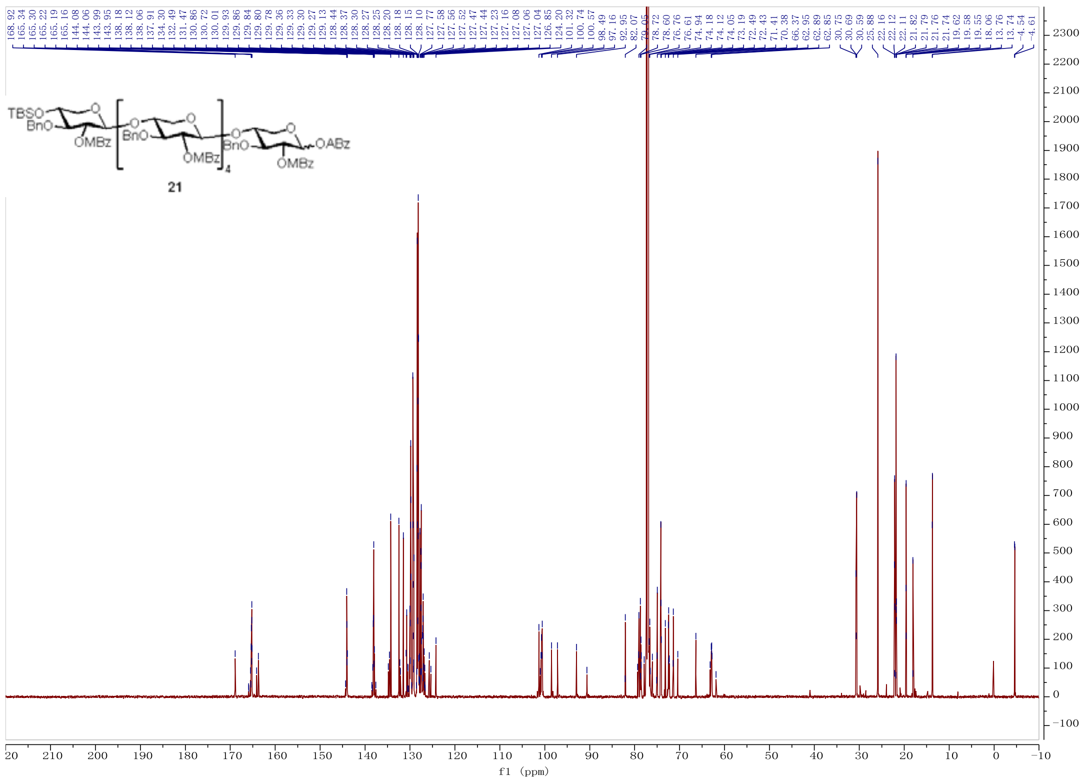

^{13}C NMR spectrum of 21 (151 MHz, $CDCl_3$, 25 °C)

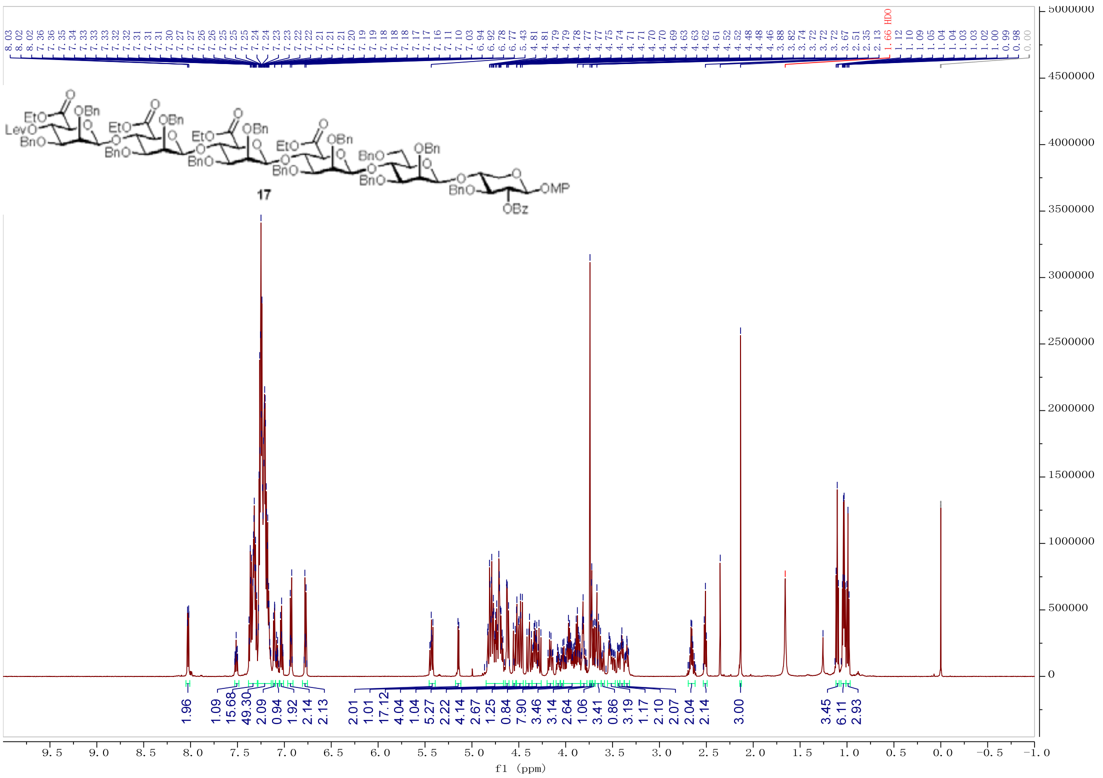

¹H NMR spectrum of 17 (600 MHz, $CDCl_3$, 25 °C)

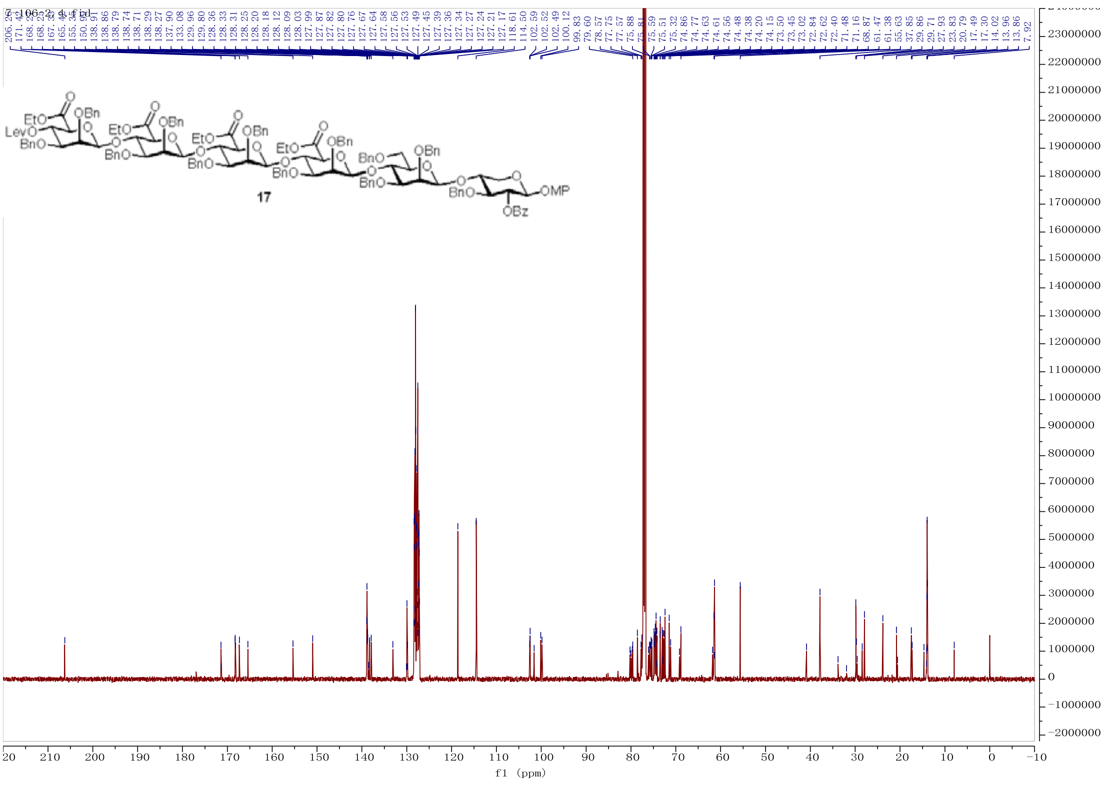

¹³C NMR spectrum of 17 (151 MHz, $CDCl_3$, 25 °C)

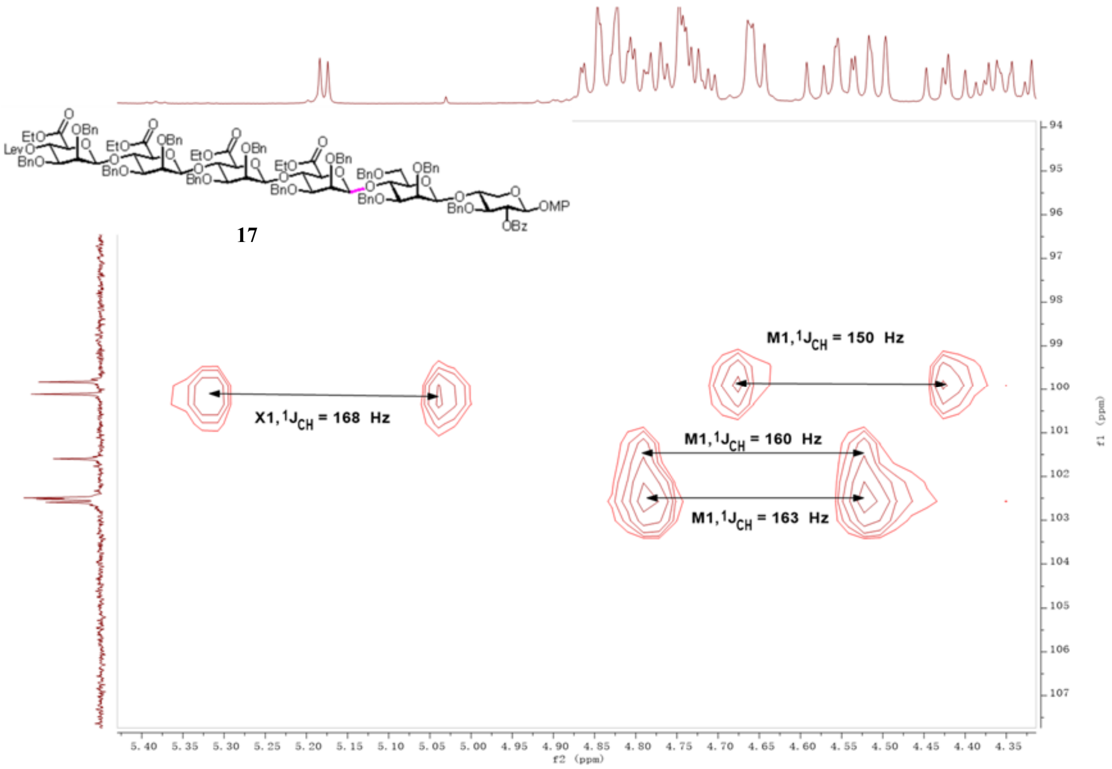

^{1}H-^{13}C coupled HSQC spectrum of 17 (600 MHz, $CDCl_3$, 25 °C)

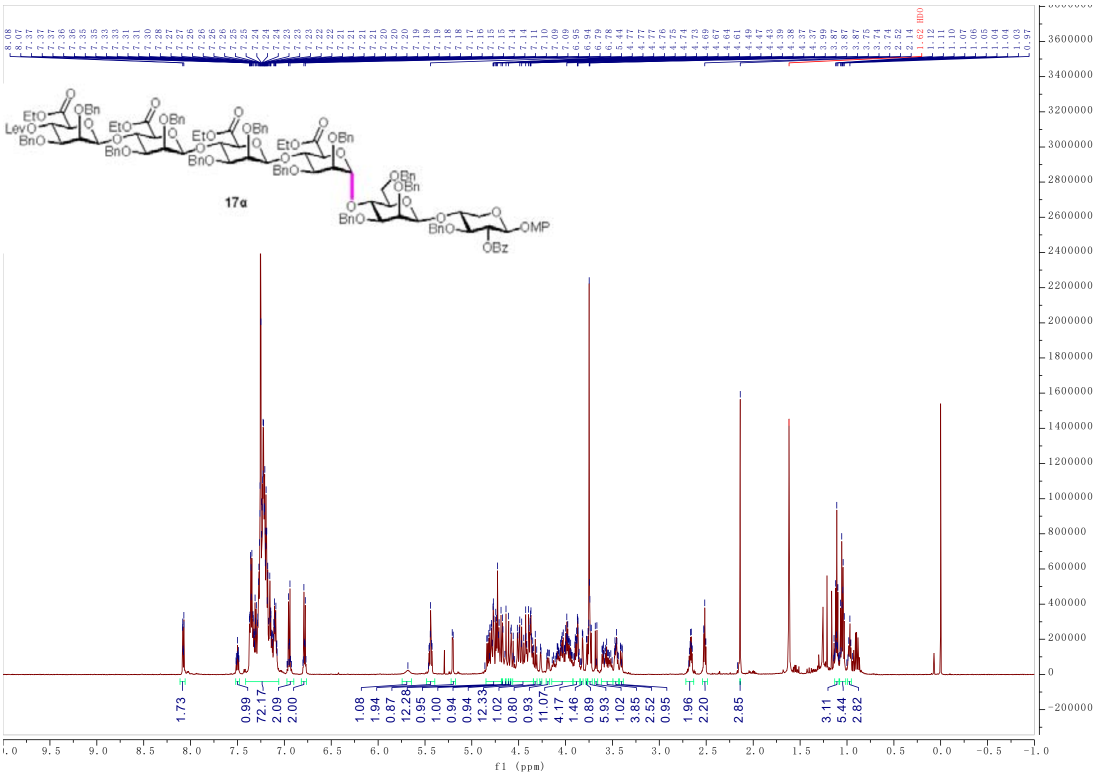

^{1}H NMR spectrum of 17α (600 MHz, $CDCl_3$, 25 °C)

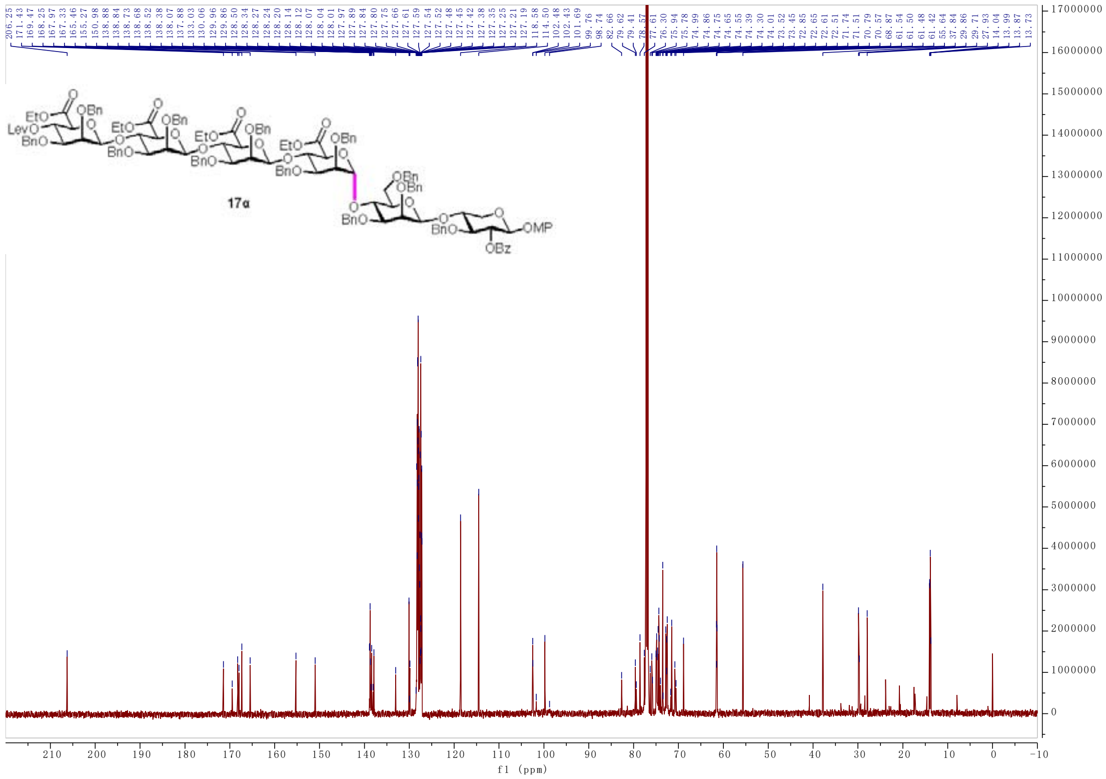

^{13}C NMR spectrum of 17α (151 MHz, $CDCl_3$, 25 °C)

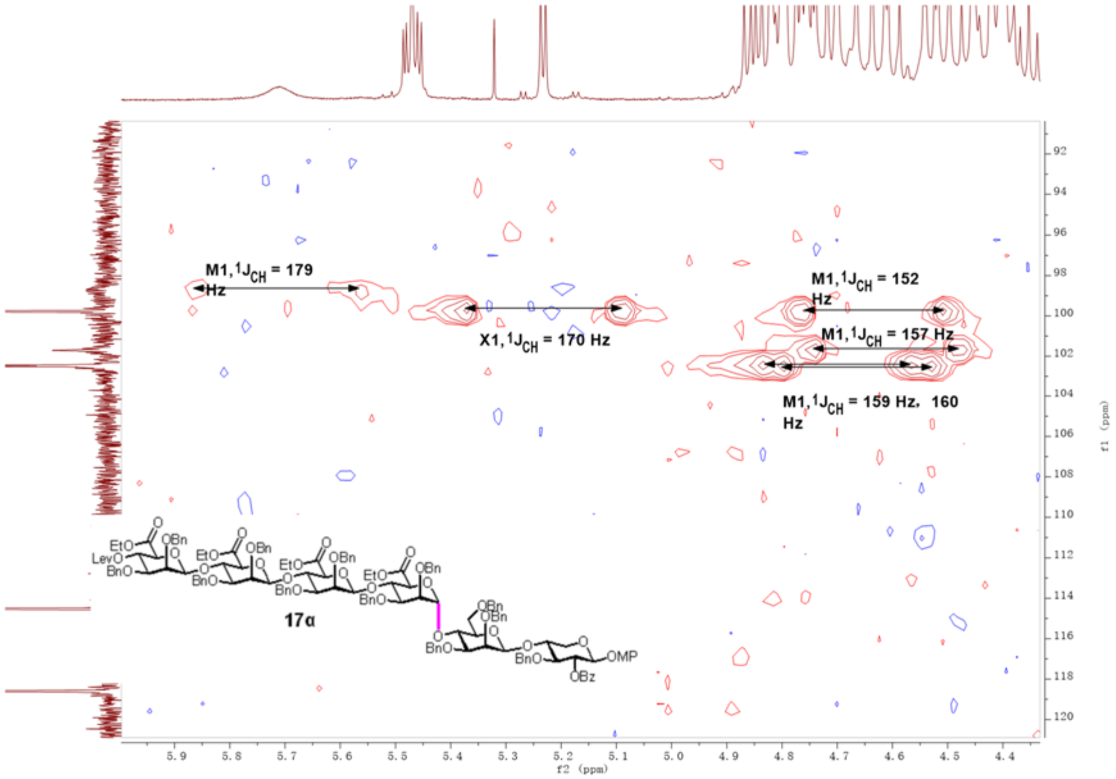

^{1}H-^{13}C coupled HSQC spectrum of 17α (600 MHz, $CDCl_3$, 25 °C)

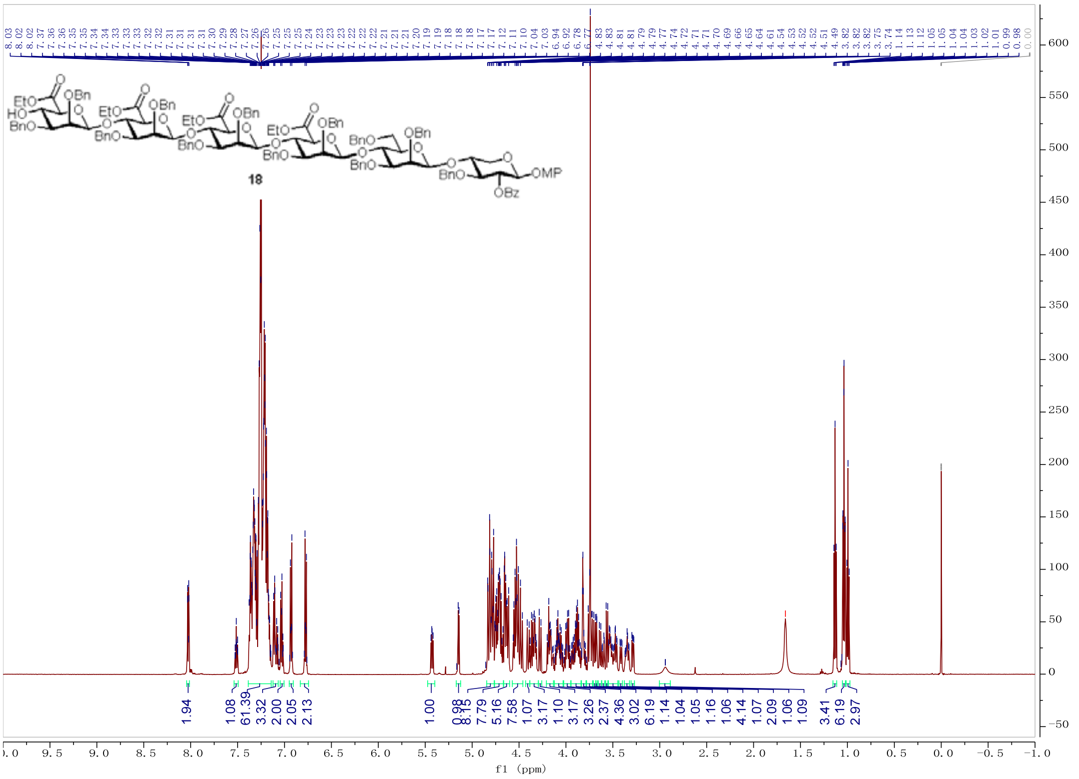

^{1}H NMR spectrum of 18 (600 MHz, $CDCl_3$, 25 °C)

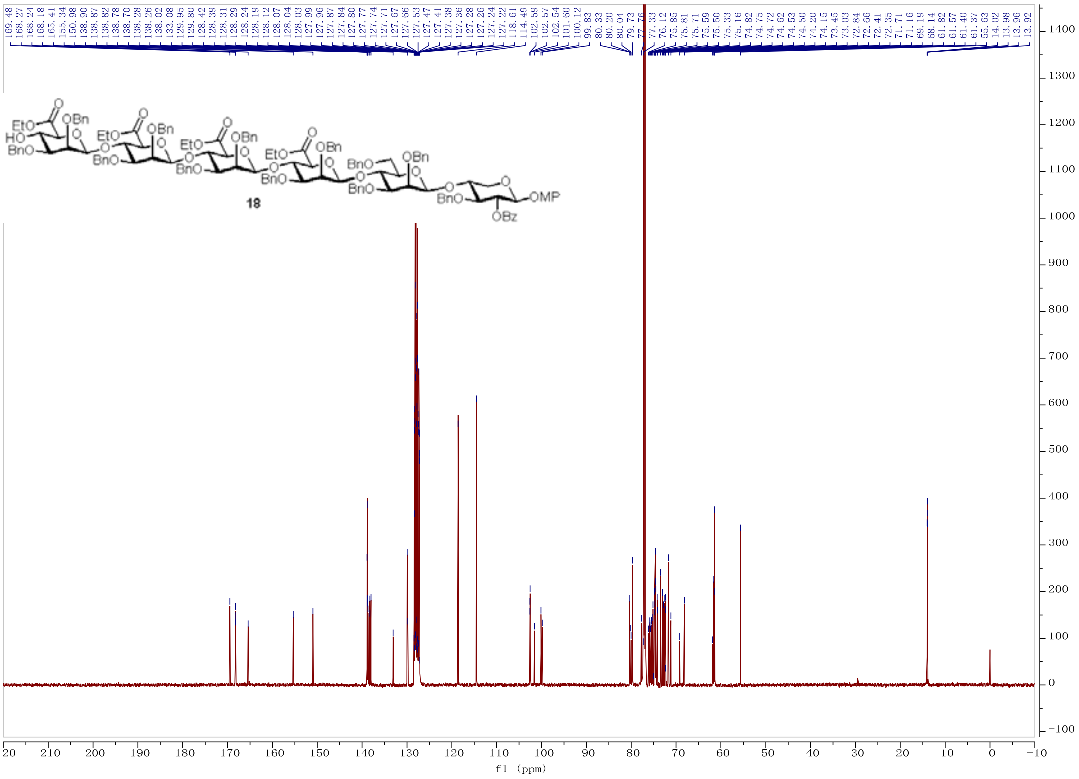

^{13}C NMR spectrum of 18 (151 MHz, $CDCl_3$, 25 °C)

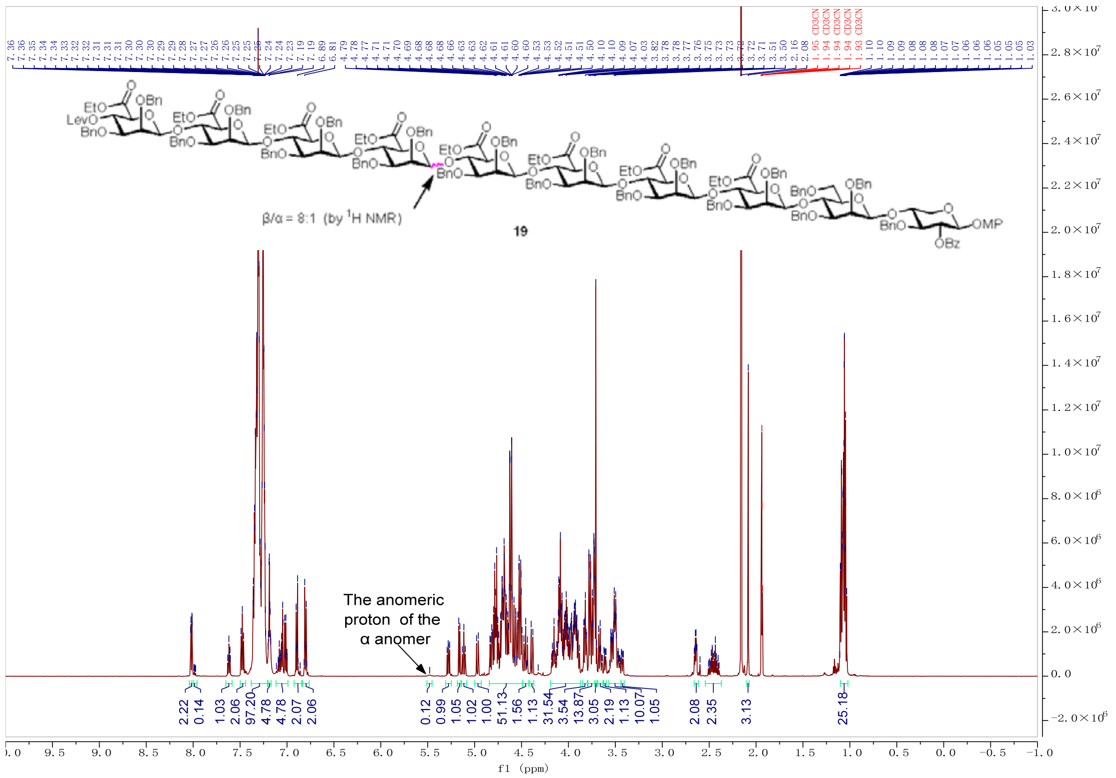

^{1}H NMR spectrum of 19 (600 MHz, $CDCl_3$, 25 °C)

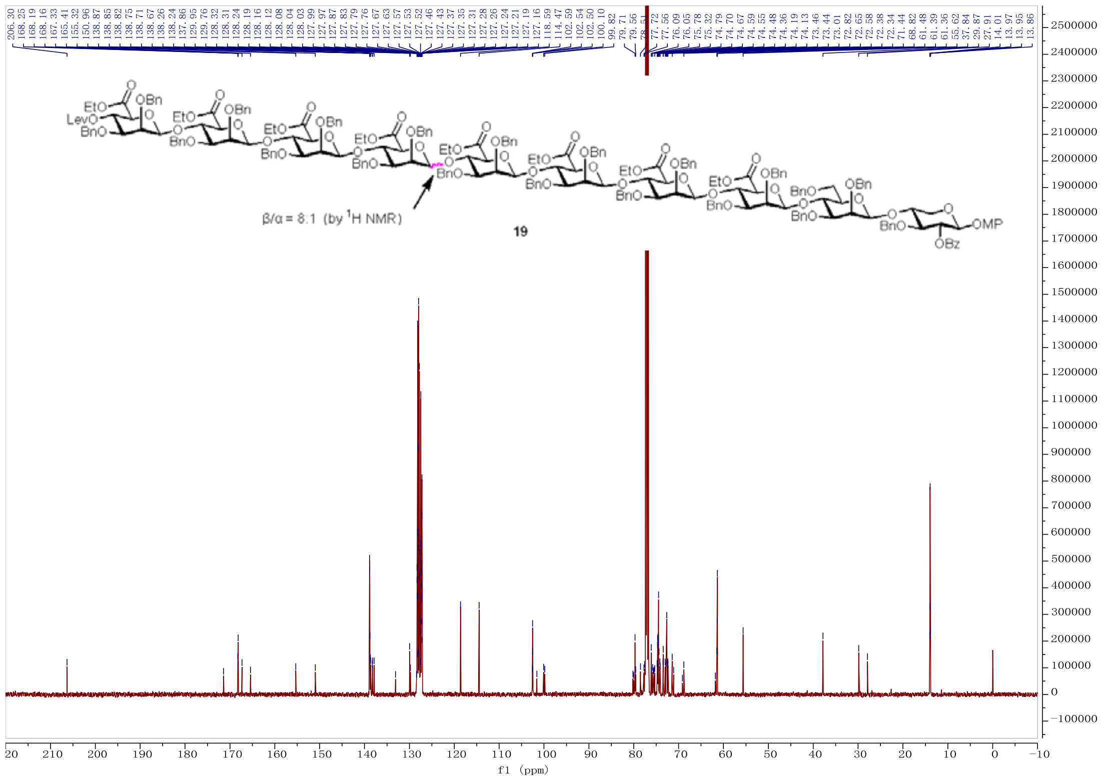

^{13}C NMR spectrum of 19 (151 MHz, $CDCl_3$, 25 °C)

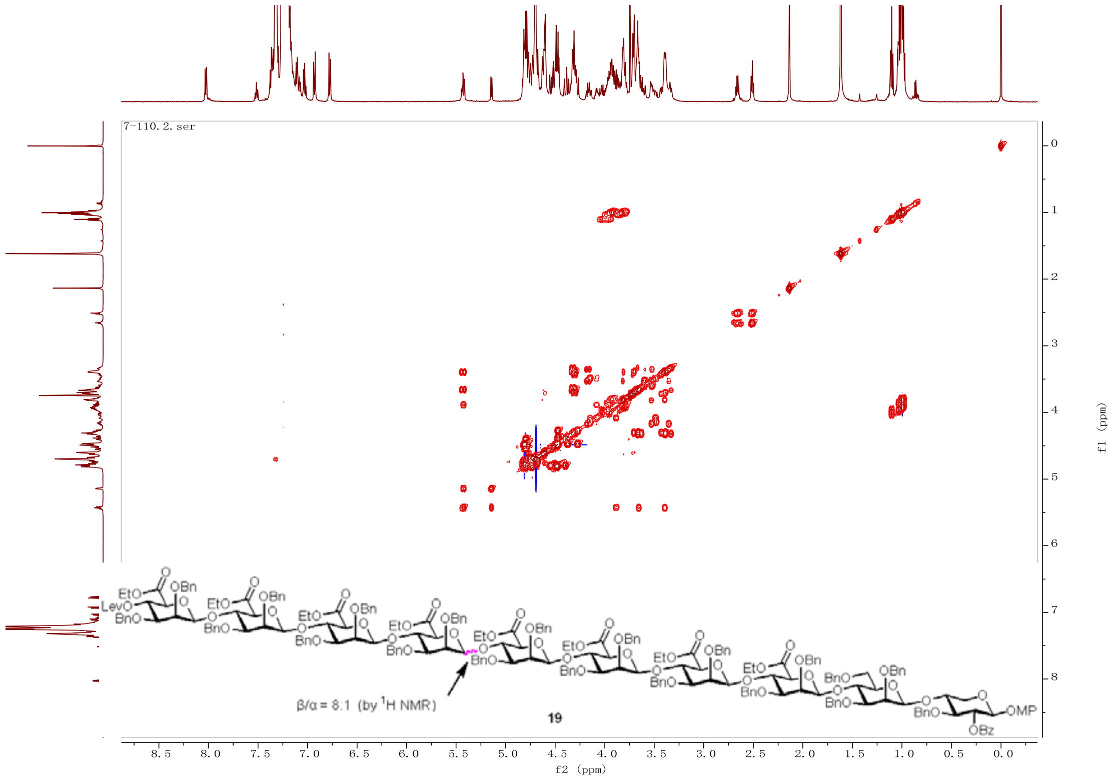

^{1}H-^{1}H gCOSY spectrum of 19 (600 MHz, $CDCl_3$, 25 °C)

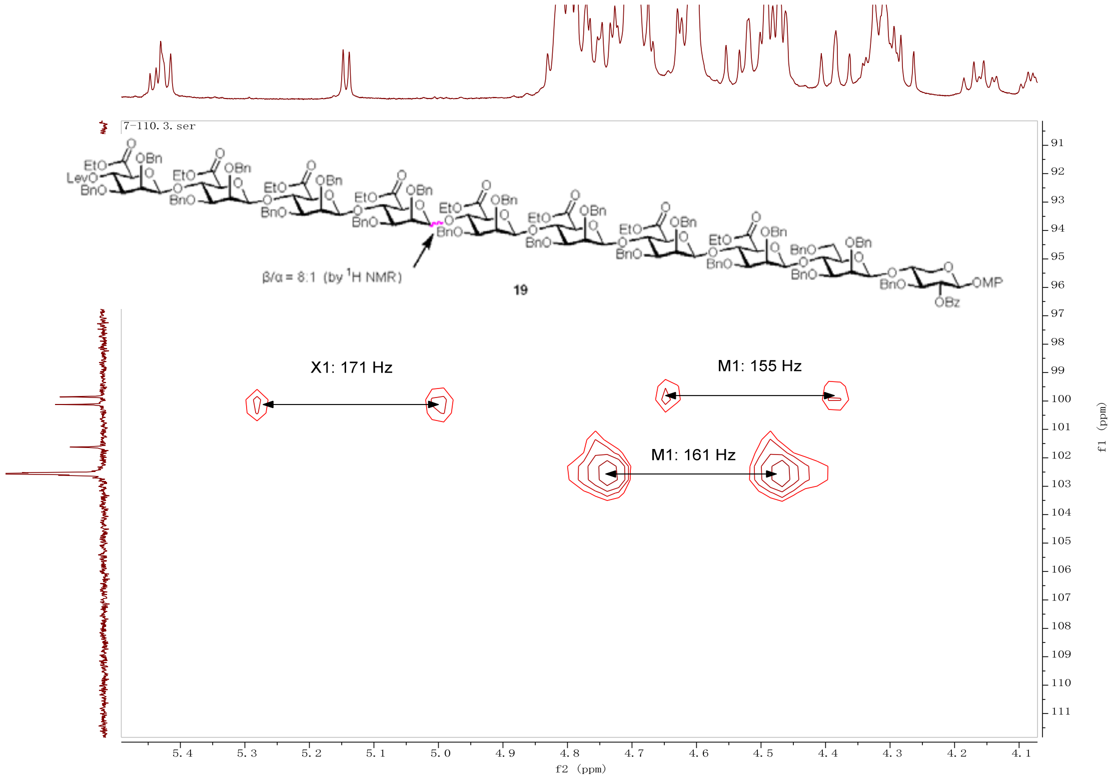

^{1}H-^{13}C coupled HSQC spectrum of 19 (600 MHz, $CDCl_3$, 25 °C)

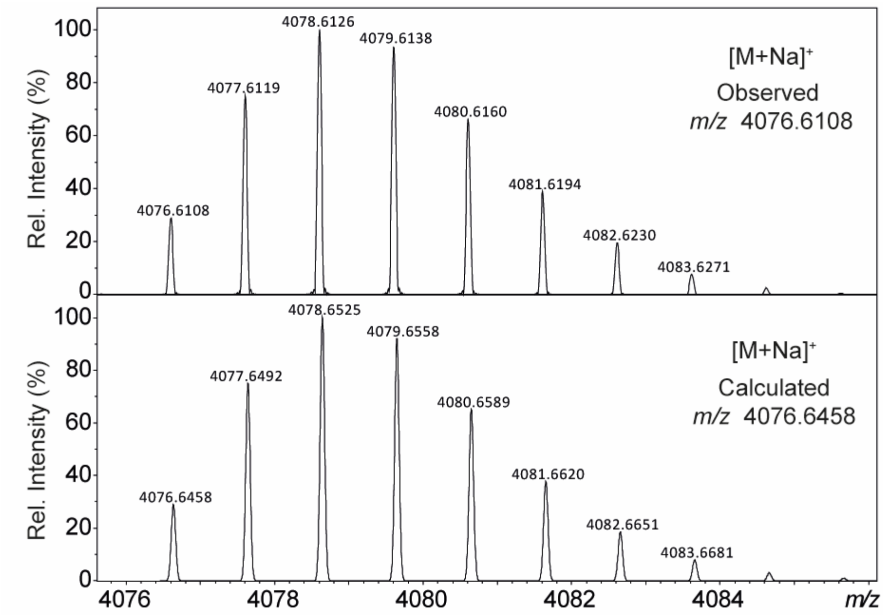

MALDI-FT-ICR MS spectrum of 19

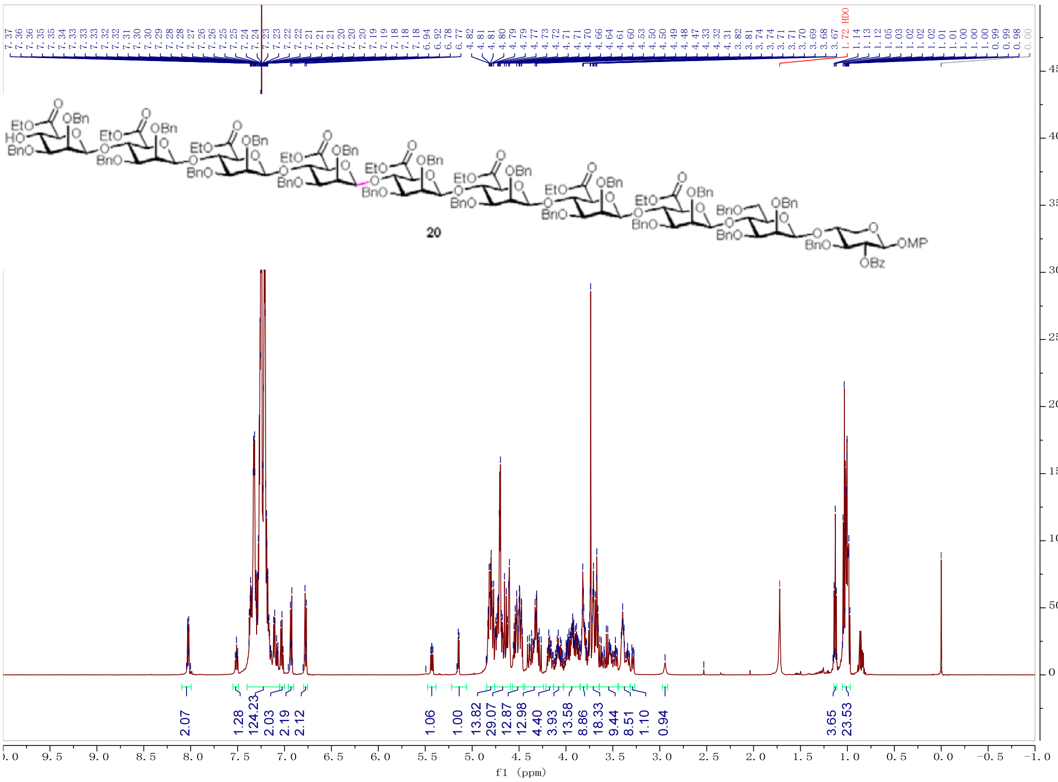

^{1}H NMR spectrum of 20 (600 MHz, $CDCl_3$, 25 °C)

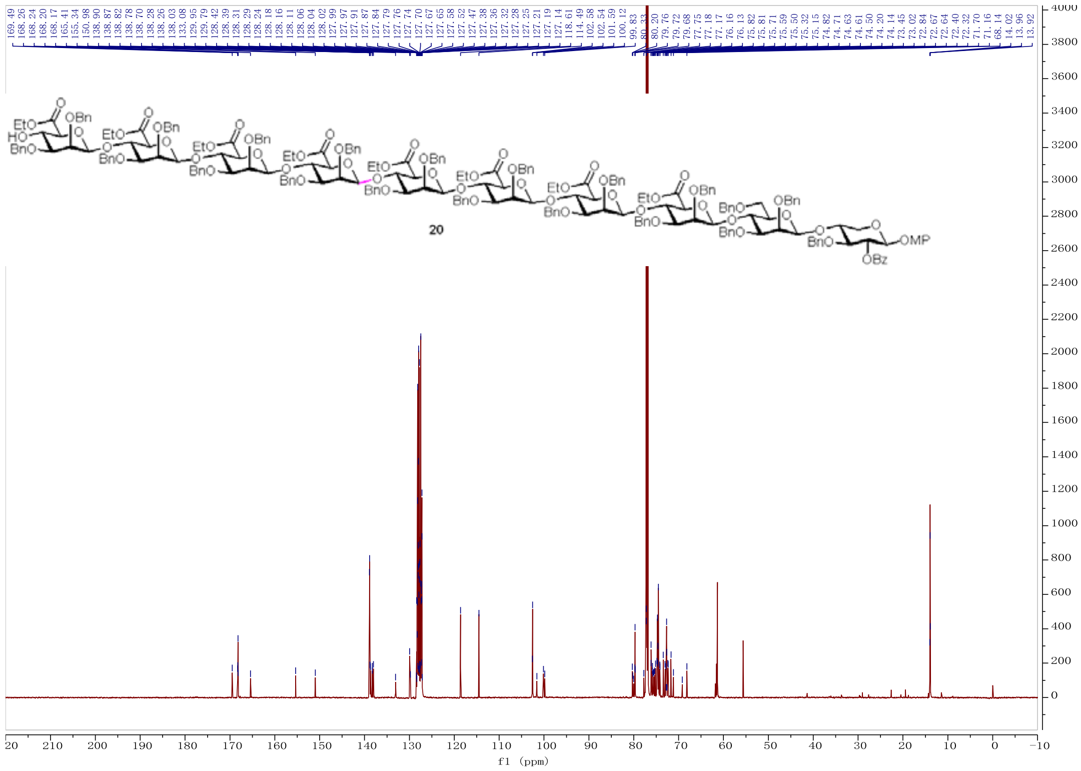

^{13}C NMR spectrum of 20 (151 MHz, $CDCl_3$, 25 °C)

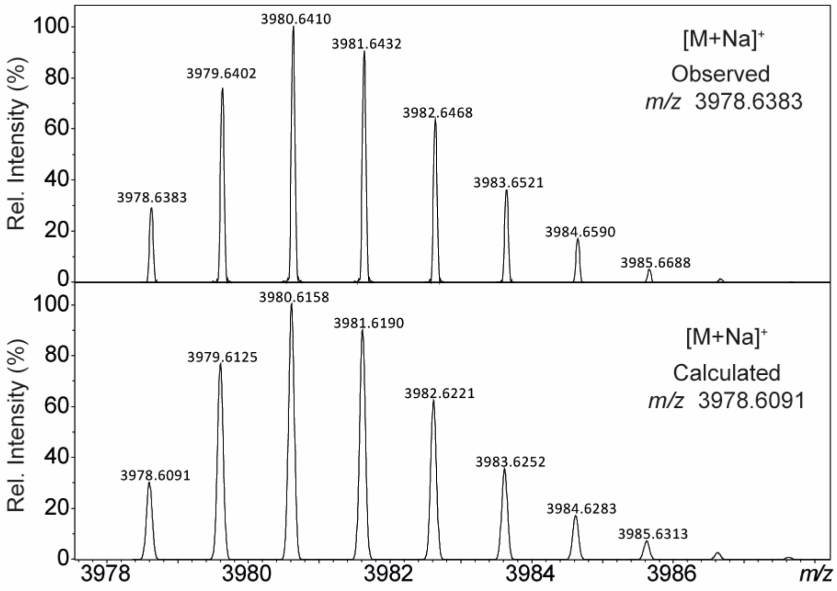

MALDI-FT-ICR MS spectrum of 20

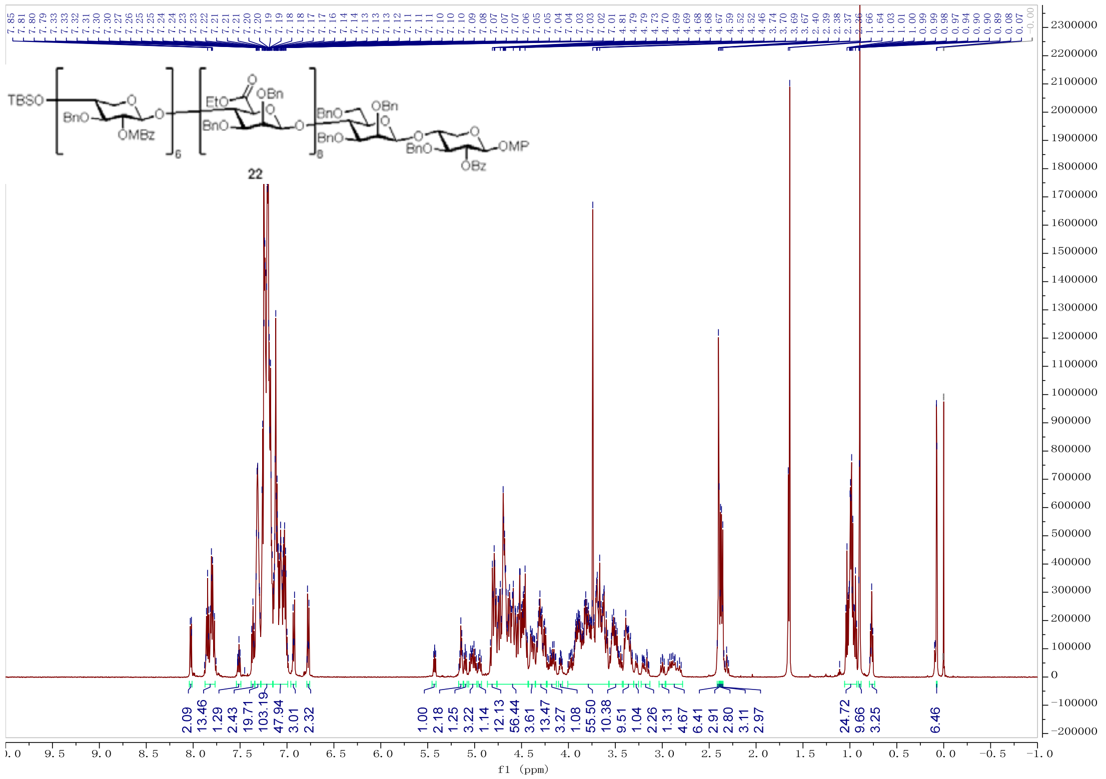

^{1}H NMR spectrum of 22 (600 MHz, $CDCl_3$, 25 °C)

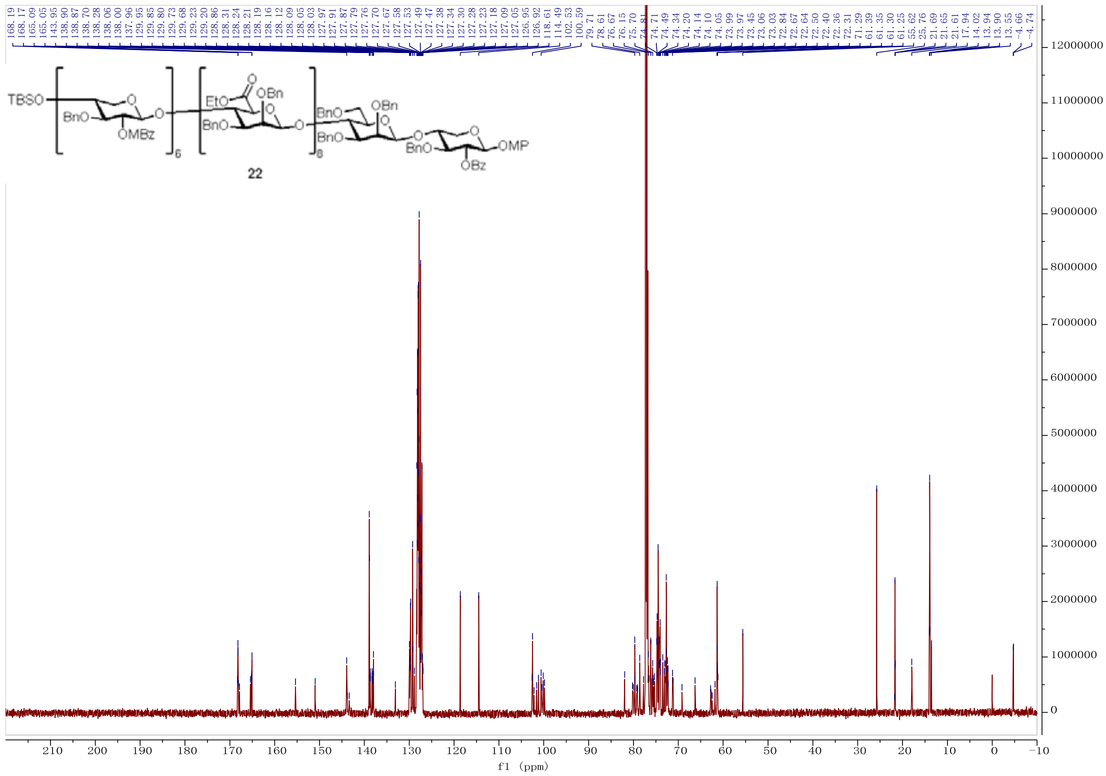

^{13}C NMR spectrum of 22 (151 MHz, $CDCl_3$, 25 °C)

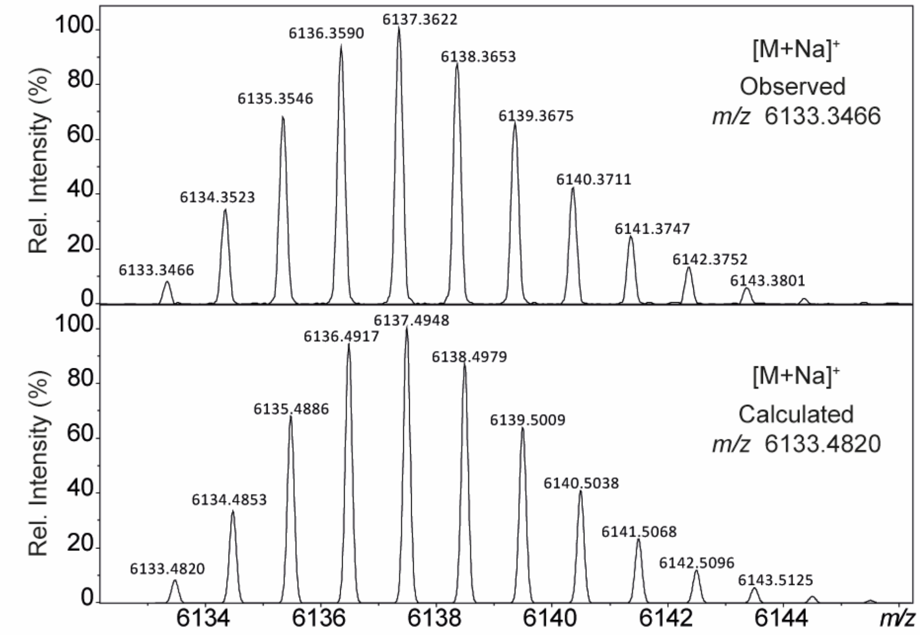

MALDI-FT-ICR MS spectrum of 22

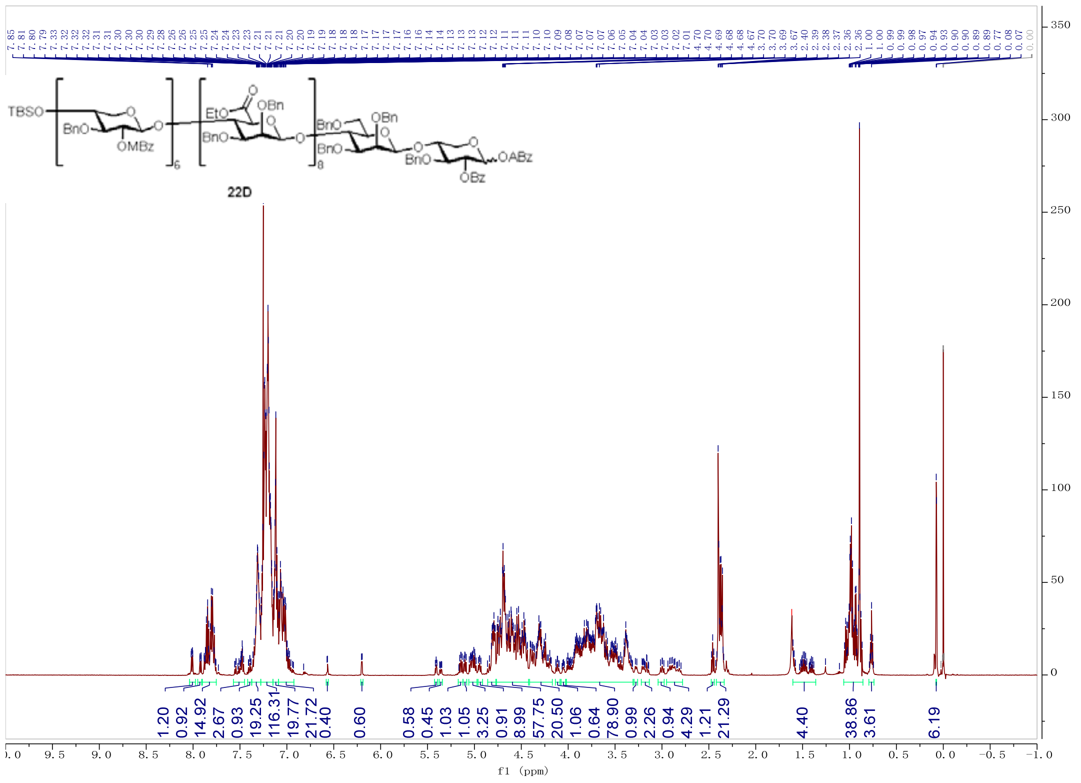

^{1}H NMR spectrum of 22D (600 MHz, $CDCl_3$, 25 °C)

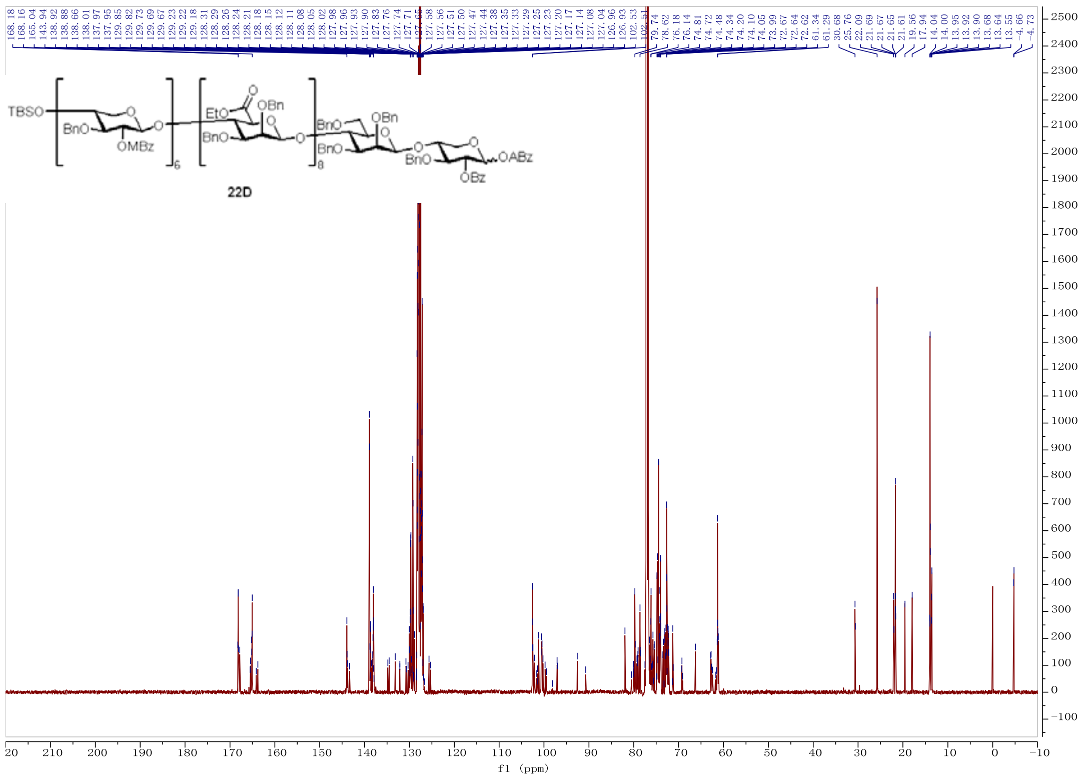

^{13}C NMR spectrum of 22D (151 MHz, $CDCl_3$, 25 °C)

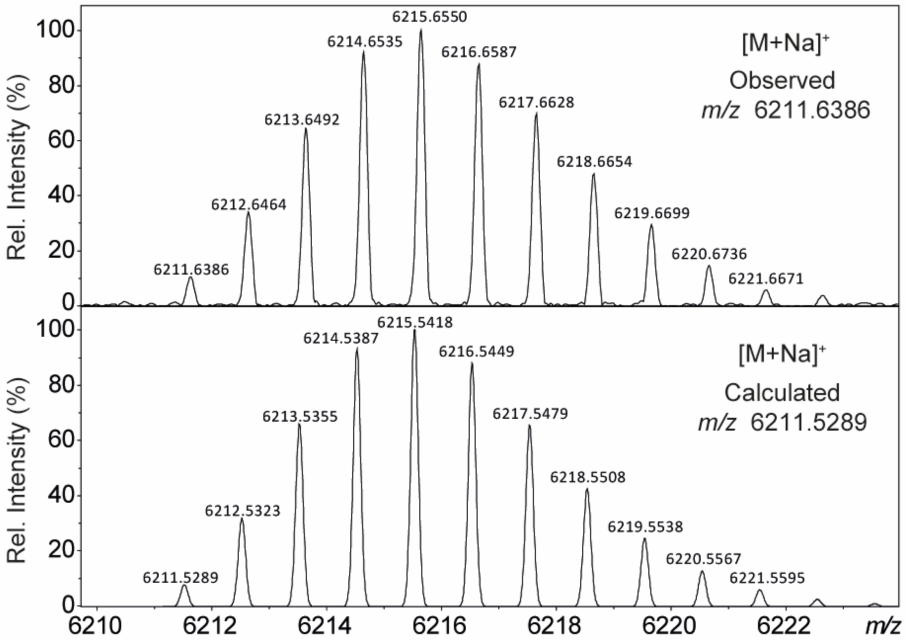

MALDI-FT-ICR MS spectrum of 22D

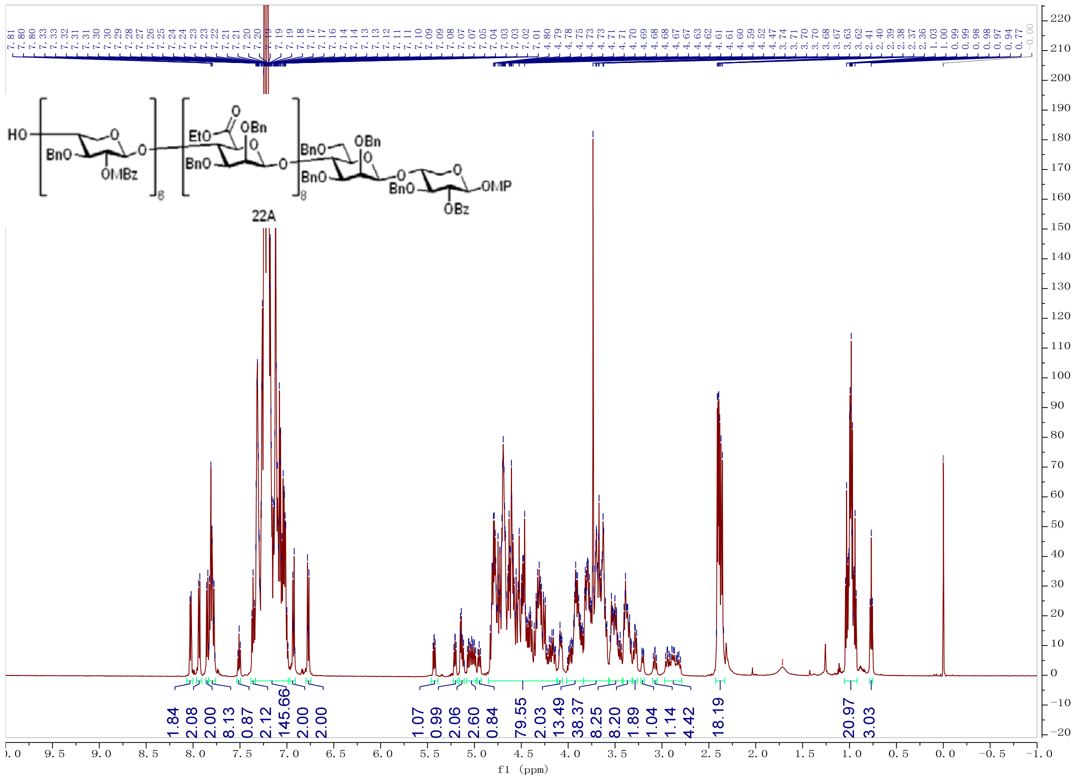

^{1}H NMR spectrum of 22A (600 MHz, $CDCl_3$, 25 °C)

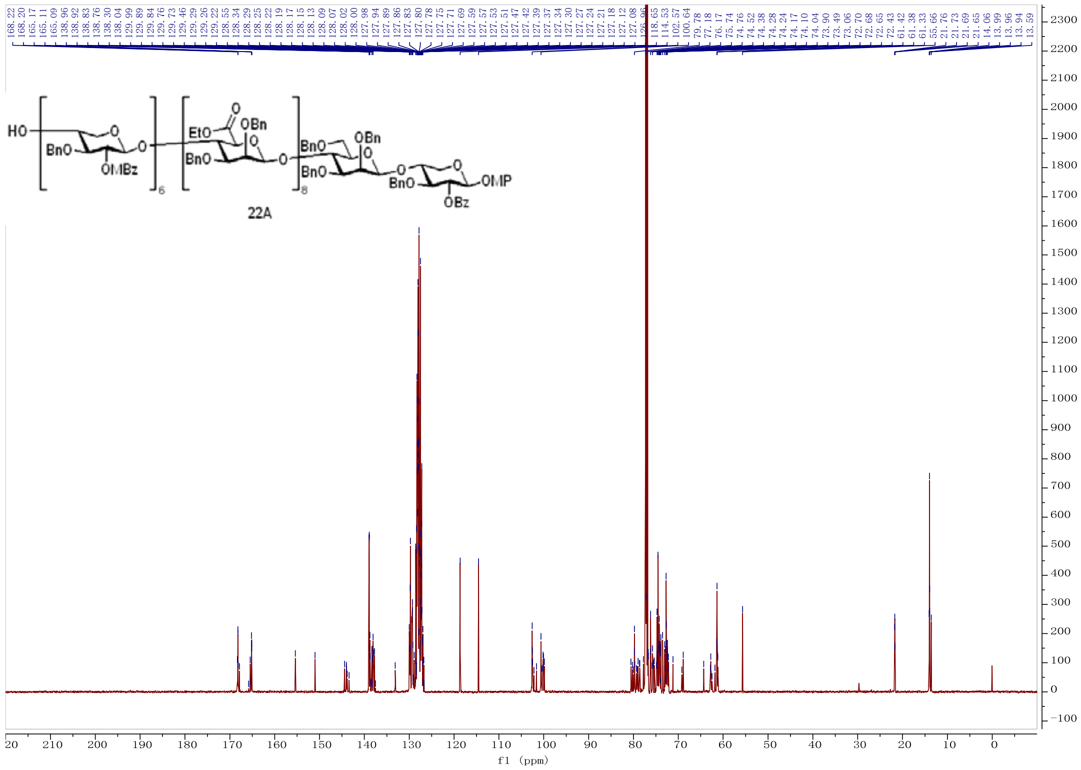

^{13}C NMR spectrum of 22A (151 MHz, $CDCl_3$, 25 °C)

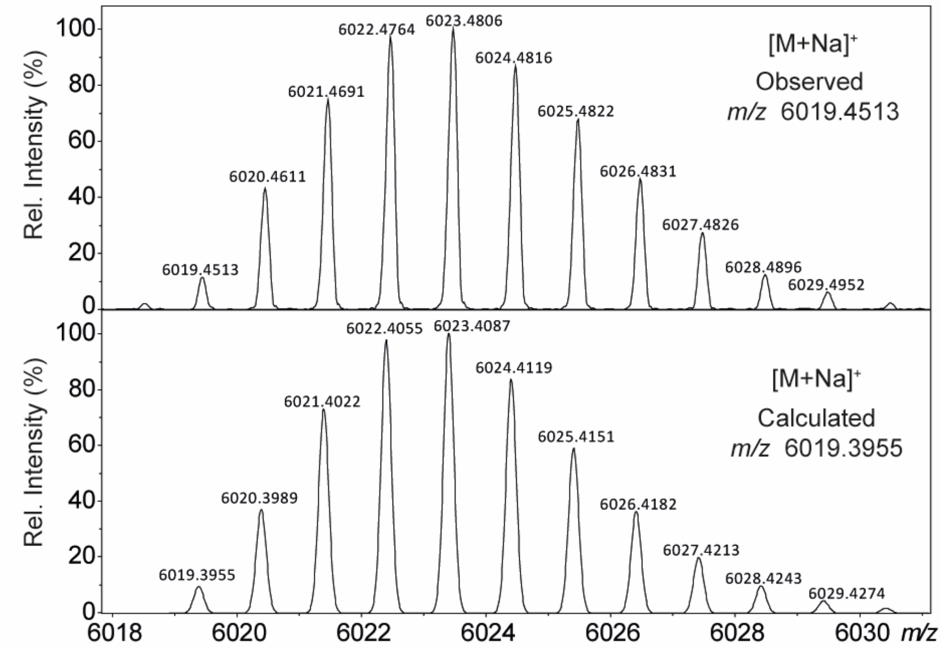

MALDI-FT-ICR MS spectrum of 22A

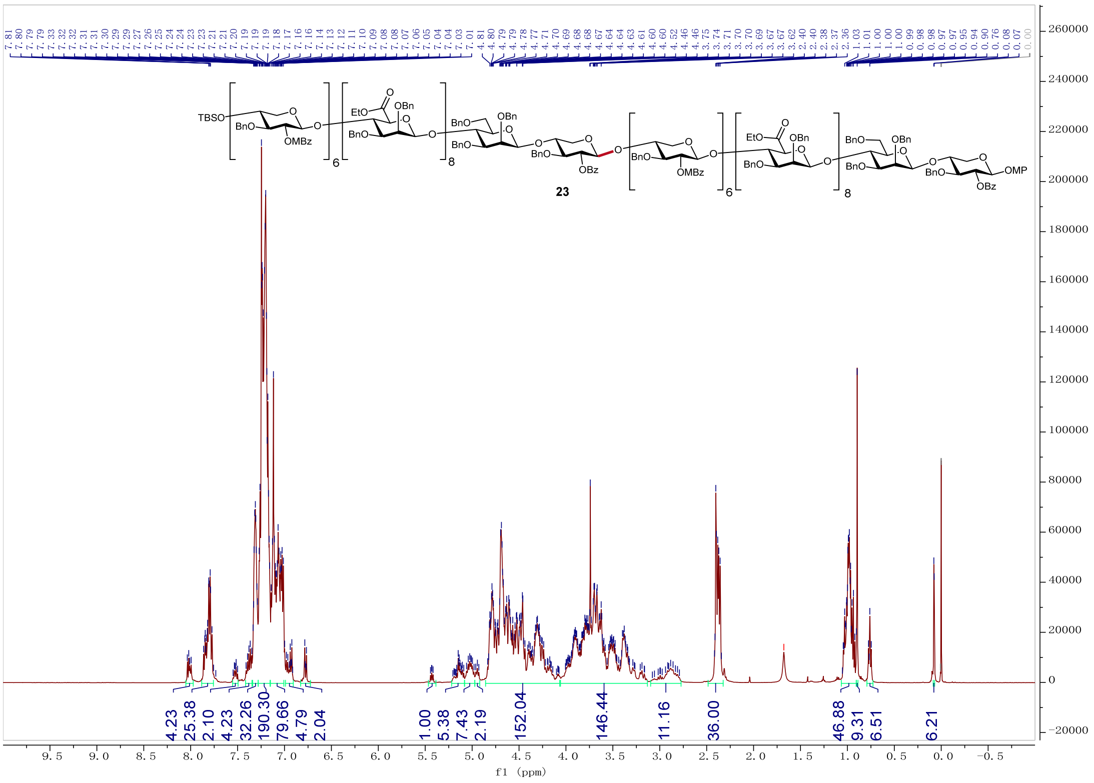

^{1}H NMR spectrum of 23 (600 MHz, $CDCl_3$, 25 °C)

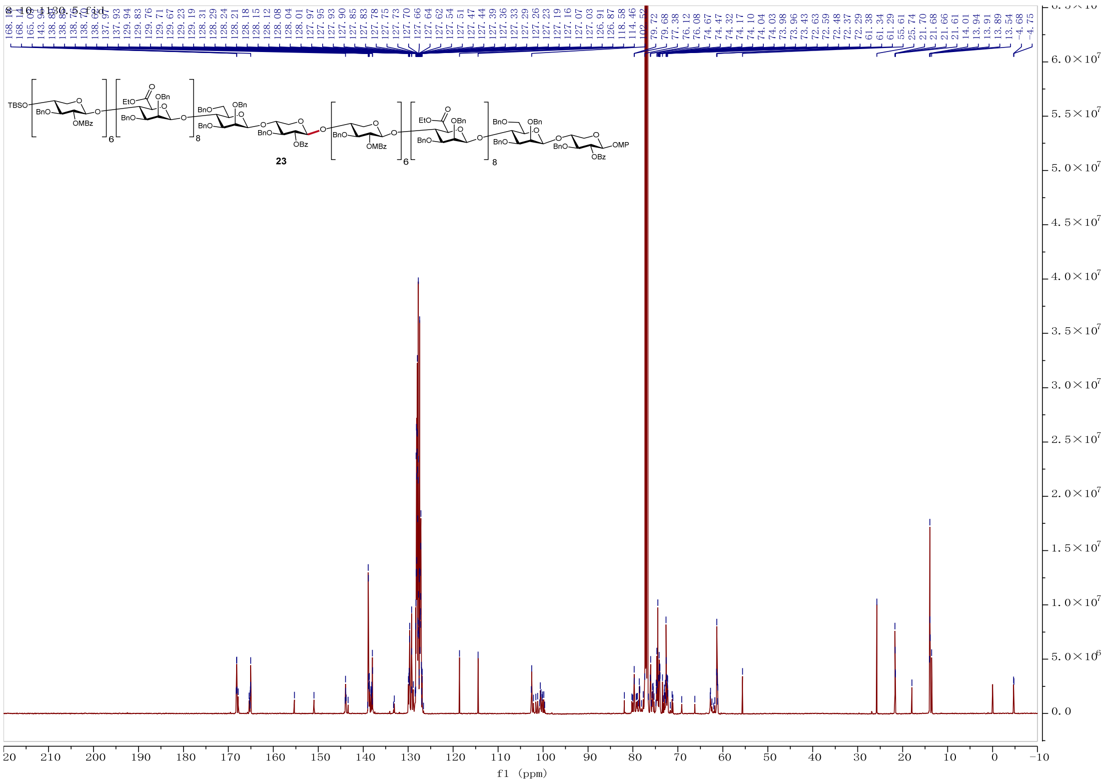

^{13}C NMR spectrum of 23 (151 MHz, $CDCl_3$, 25 °C)

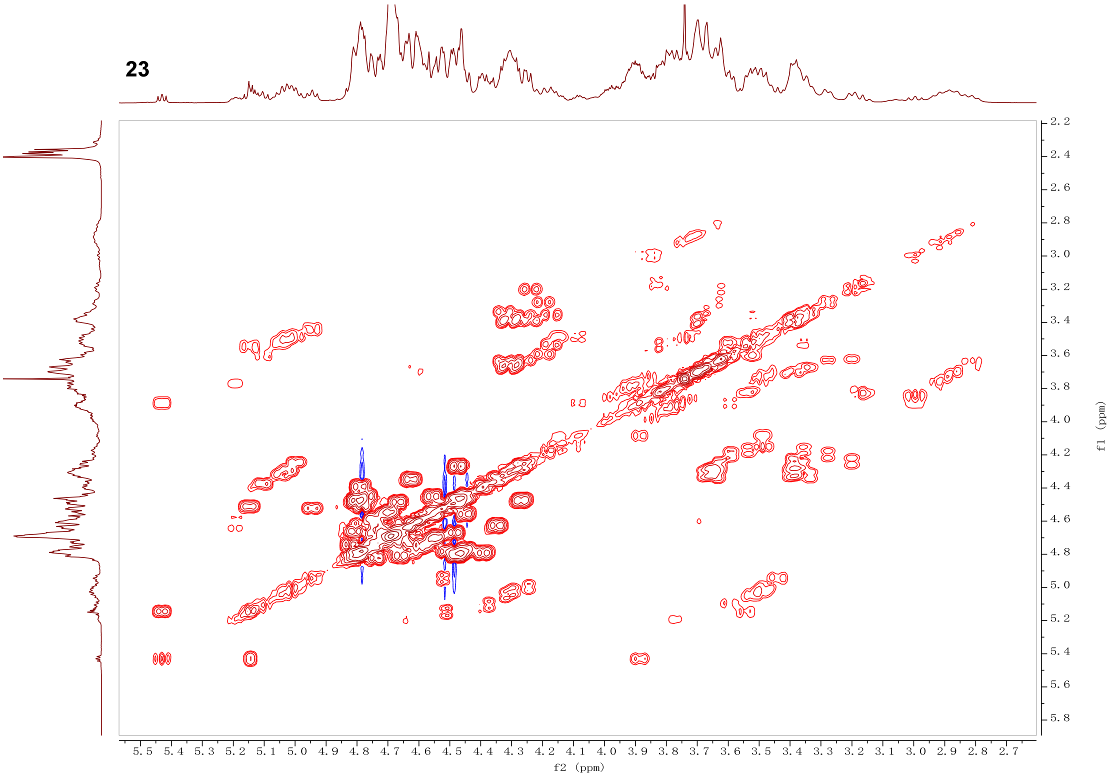

^{1}H-^{1}H gCOSY spectrum of 23 (600 MHz, $CDCl_3$, 25 °C)

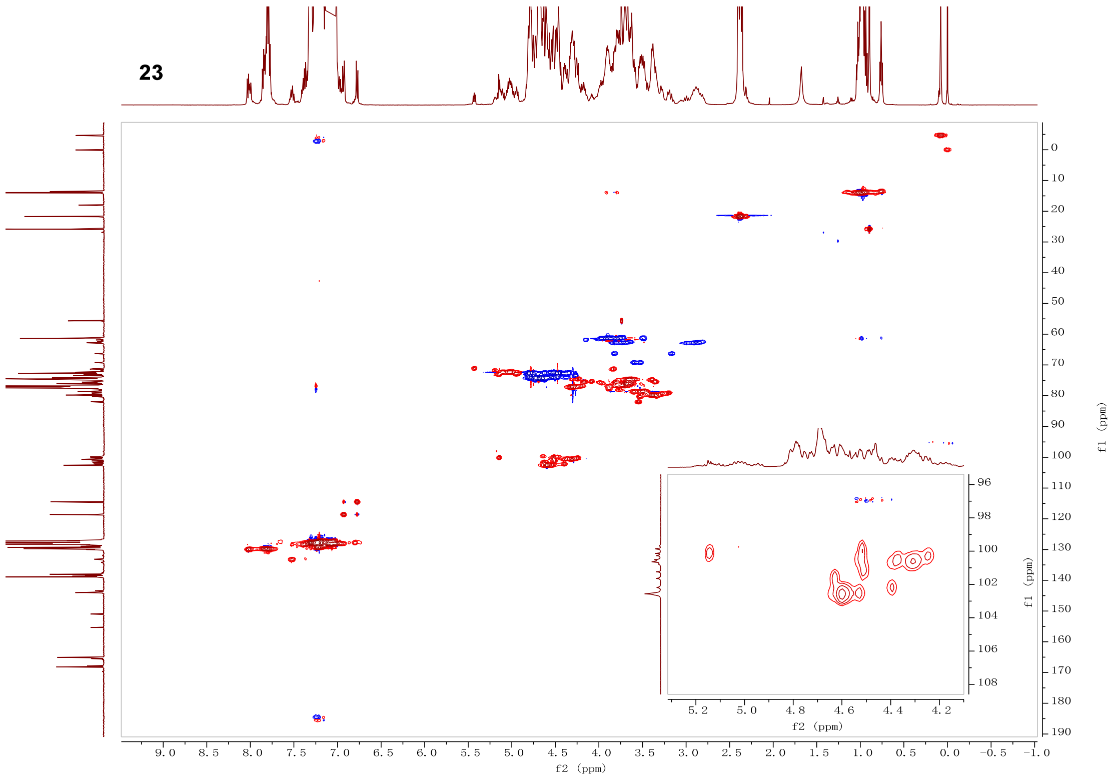

^{1}H-^{13}C HSQC spectrum of 23 (600 MHz, $CDCl_3$, 25 °C)

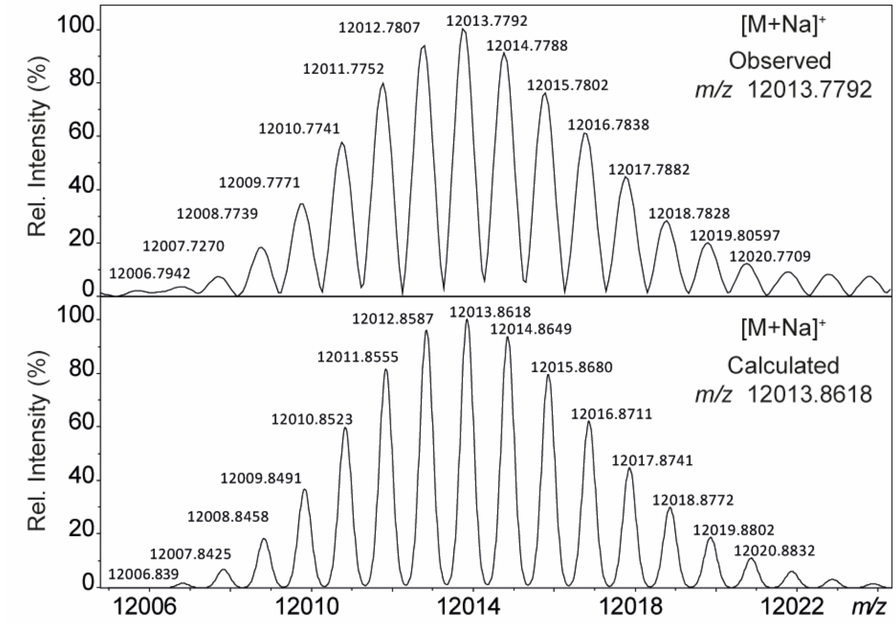

MALDI-FT-ICR MS spectrum of 23

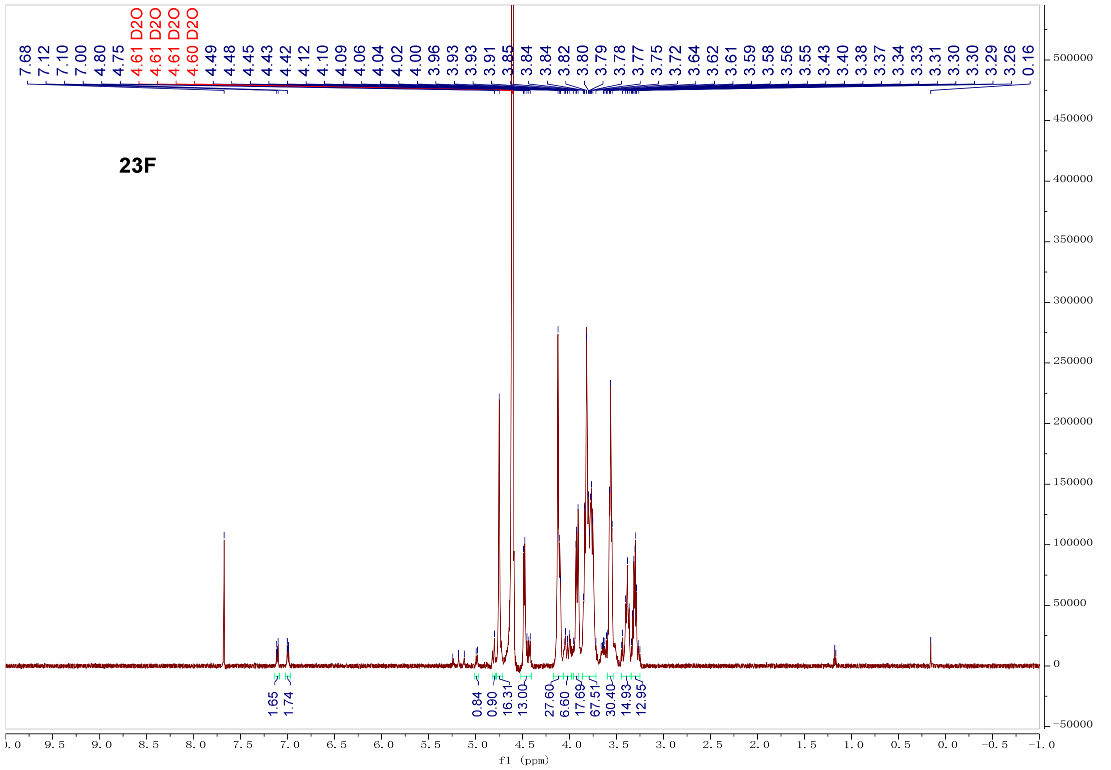

^{1}H NMR spectrum of compound 23F (600 MHz, 20 mM Na_3PO_4 buffered D_2O, 40 °C)

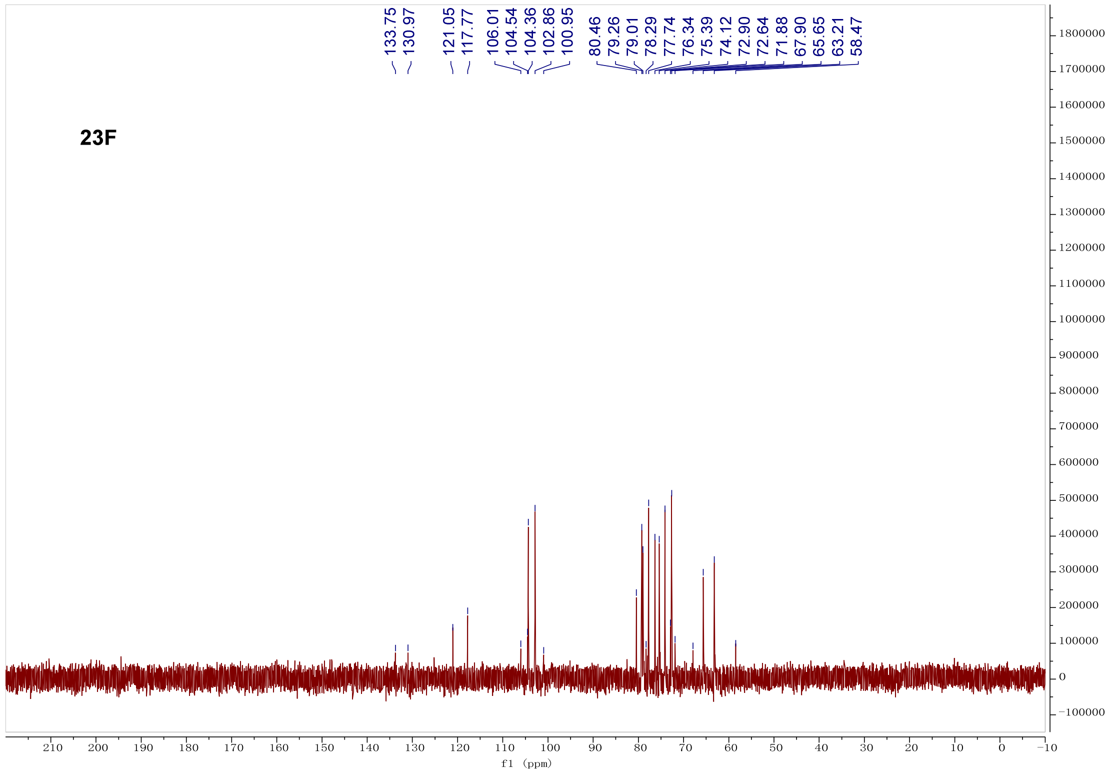

^{13}C NMR spectrum of compound 23F (151 MHz, 20 mM Na_3PO_4 buffered D_2O, 40 °C)

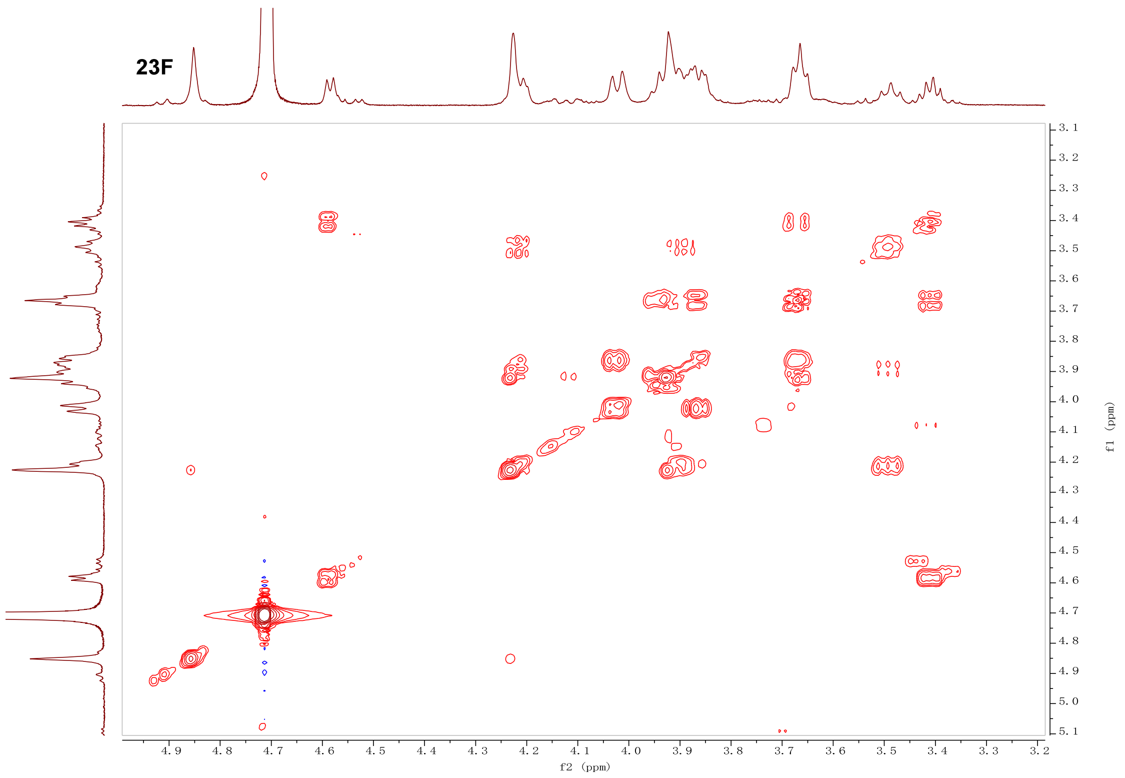

^{1}H-^{1}H gCOSY spectrum of compound 23F (600 MHz, 20 mM Na_3PO_4 buffered D_2O, 40 °C)

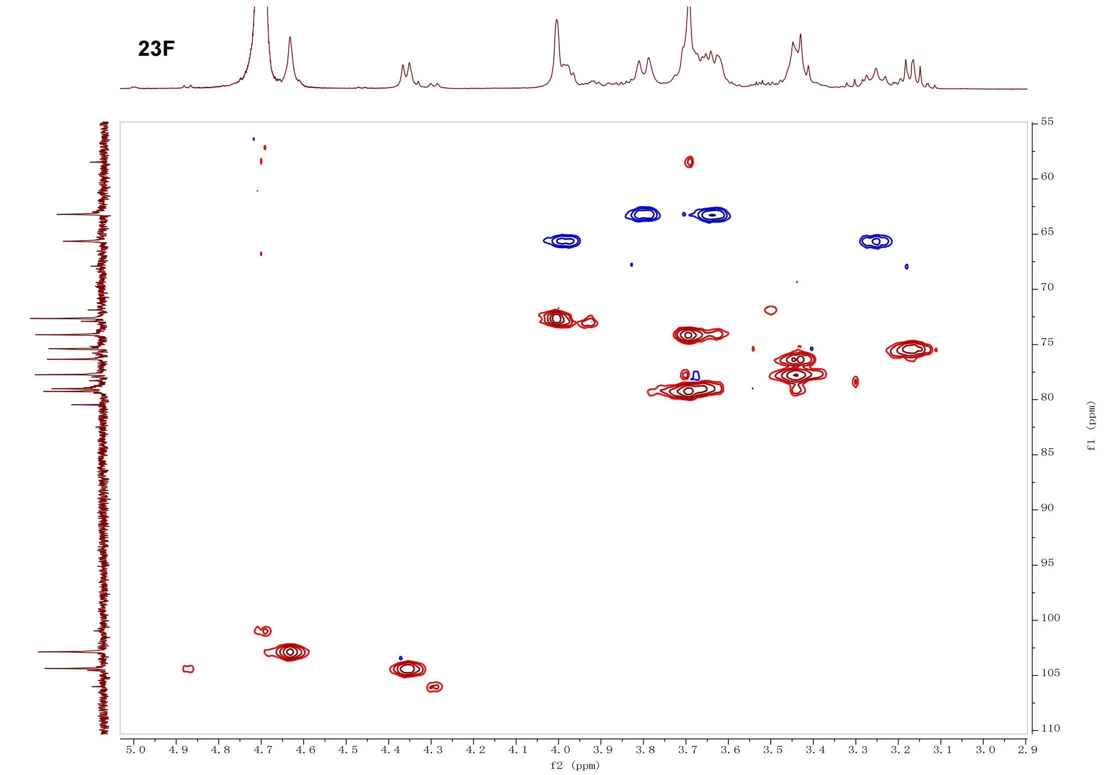

^{1}H-^{13}C HSQC spectrum of compound 23F (600 MHz, 20 mM Na_3PO_4 buffered D_2O, 40 °C)

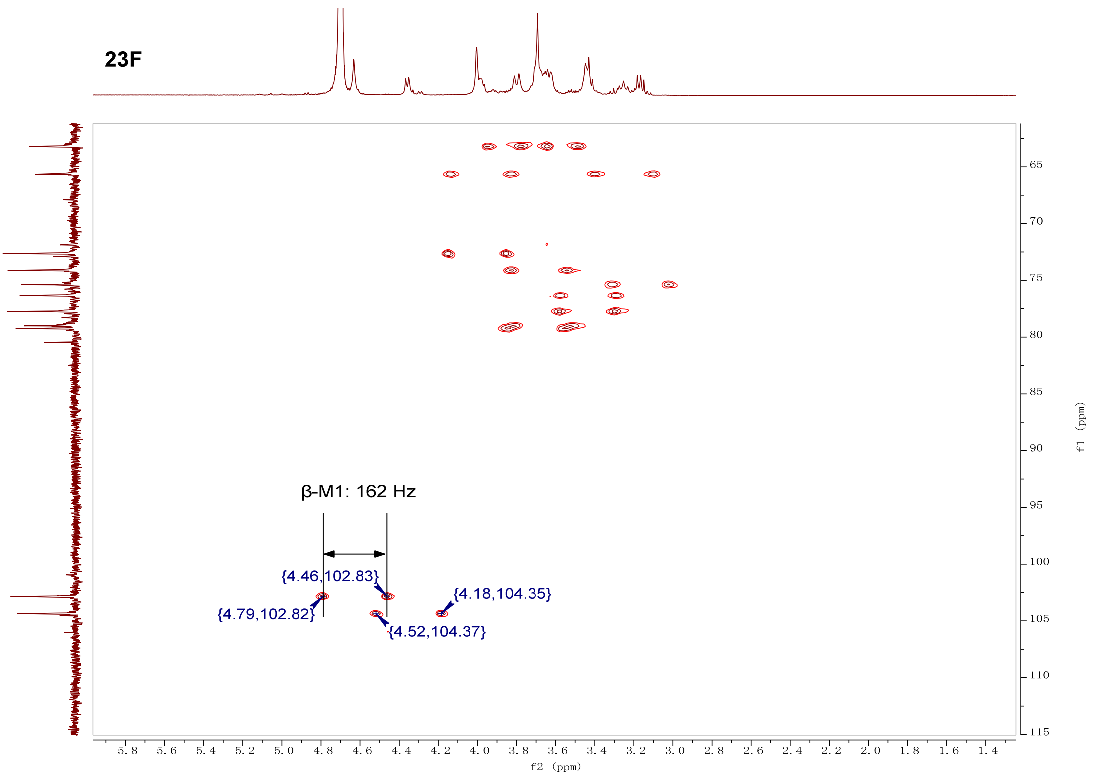

^{1}H-^{13}C coupled HSQC spectrum of compound 23F (600 MHz, 20 mM Na_3PO_4 buffered D_2O, 40 °C)

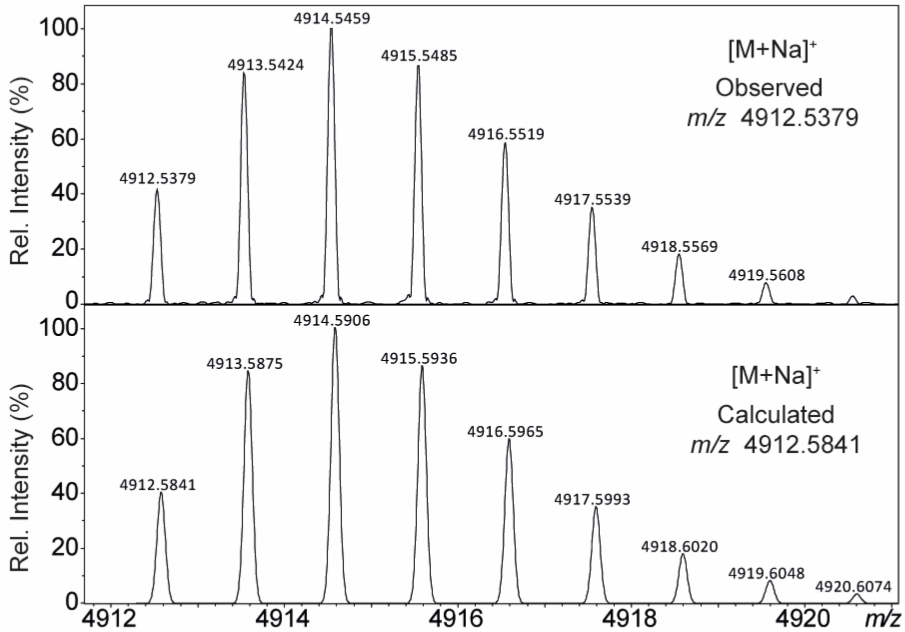

MALDI-FT-ICR MS spectrum of 23F
